# Using Noun Phrases for Navigating Biomedical Literature on Pubmed: How Many Updates Are We Losing Track of?

**DOI:** 10.1371/journal.pone.0024920

**Published:** 2011-09-14

**Authors:** Devabhaktuni Srikrishna, Marc A. Coram

**Affiliations:** 1 authorsurvey.com, San Mateo, California, United States of America; 2 Health Research and Policy, Stanford University, Stanford, California, United States of America; University of Chicago, United States of America

## Abstract

Author-supplied citations are a fraction of the related literature for a paper. The “related citations” on PubMed is typically dozens or hundreds of results long, and does not offer hints why these results are related. Using noun phrases derived from the sentences of the paper, we show it is possible to more transparently navigate to PubMed updates through search terms that can associate a paper with its citations. The algorithm to generate these search terms involved automatically extracting noun phrases from the paper using natural language processing tools, and ranking them by the number of occurrences in the paper compared to the number of occurrences on the web. We define search queries having at least one instance of overlap between the author-supplied citations of the paper and the top 20 search results as citation validated (CV). When the overlapping citations were written by same authors as the paper itself, we define it as CV-S and different authors is defined as CV-D. For a systematic sample of 883 papers on PubMed Central, at least one of the search terms for 86% of the papers is CV-D versus 65% for the top 20 PubMed “related citations.” We hypothesize these quantities computed for the 20 million papers on PubMed to differ within 5% of these percentages. Averaged across all 883 papers, 5 search terms are CV-D, and 10 search terms are CV-S, and 6 unique citations validate these searches. Potentially related literature uncovered by citation-validated searches (either CV-S or CV-D) are on the order of ten per paper – many more if the remaining searches that are not citation-validated are taken into account. The significance and relationship of each search result to the paper can only be vetted and explained by a researcher with knowledge of or interest in that paper.

## Introduction

Today, there is no systematic way to keep track of individual discoveries of the best known related literature on any research topic, especially for the more interdisciplinary or esoteric topics. Search engines like PubMed order results by how recent they are. Google Scholar has mastered search of biomedical literature based on user-supplied keywords and search ranking algorithms. As the research literature expands and opens up to discovery due to the success of pre-print servers and open-access journals, searches on PubMed and the web are returning large numbers of results – the barrier to discovery is the vast size of the corpus and rapid rate of updates of potentially related literature. Over the past 20 years, PubMed has reached nearly 20 million records and has grown annually at a compound rate of ∼4% [1]. That currently works out to approximately 2000 papers per day on average. How can individual researchers expect to keep up even with state of the art search interfaces?

Part of our motivation for this study is to explore a scalable way of not only identifying, but also navigating to potentially related literature to a paper that also incorporates some degree of author verification. With that in mind, we ask how easy is it to recover author supplied citations by searching for them on PubMed? Using ranked noun phrases extracted from papers, we construct searches to observe potentially related literature on PubMed through search results that also contain the citations. In contrast to benchmarks traditionally used in text retrieval, we propose a new method called citation validation, to validate search terms – it applies more generally to any technique for discovery and tracking of related literature on PubMed.

Author-supplied citations for PubMed papers form a citation graph [2], whose nodes are the citing and cited papers (on PubMed) or web links (not necessarily part of Pubmed). In general, the citation graph represents a valuable, though small fraction of the entire body of literature relevant to readers of a paper. But often readers want to identify other related literature. For example, the “related citations” feature of PubMed is derived from text-analysis of papers (See “Computation of Related Citations.” <http://www.ncbi.nlm.nih.gov/books/NBK3827/#pubmedhelp.Computation_of_Related_Citati>), and for each paper on PubMed provides a single ranked list of typically several dozen PubMed papers that may be related. For each word or term in each paper, a numeric weight is computed based on the number of times the word occurs in the paper and the number of papers that the term occurs in within PubMed. These term-weights are used to find the most similar pairs of papers by computing the dot product of the vector of weights. Clicks on the “related citations” link comprise a fifth of all user sessions on PubMed [3] indicating it is often utilized by researchers. Besides PubMed's “related citations,” several alternate approaches exist for discovering new and related work from the text of research papers such as [4].

We define the related literature to consist of any documents helpful to readers. For any scientific or technical paper these may include,

1. Non-obvious connections: If there is a relationship between two papers that is useful, non-obvious, and not recorded in the citation graph such as new technology that may be applicable.
2. Summaries: A video, review, or discussion of a paper on a blog.
3. Newer research: Newer, yet relevant, research results published after the paper was published.
4. Foundational: Supporting references, foundational work, or a tutorial that would be useful or helpful to readers of a paper.
5. Terminology Variants: When terminology changes in newer research, keyword searches may not reveal related material. Scientific notation can be inconsistent, author-dependent, or change over time due to new discoveries where the new terminology is not incorporated in older references. For example, references involving Mendel's laws, genetics, and DNA.
6. Competition: Any knowledge challenging anything in a research article including counterexamples, counterpoints, and competitive research. Some researchers may not cite their competitors or simply be unaware of related or contradictory papers. Reviewers may not always correct them.
7. Closed-access: Not everyone may be aware of the contents of a closed-access paper – this includes most articles on PubMed which give public access to author-supplied title/abstract – not the full text.

Related literature is defined by what is meaningful to readers of each paper, and each reader may have their own opinion while informed readers may agree on some smaller subset. PubMed's “related citations” is neither a complete list of all related literature, nor are all items in the list necessarily part of the related literature.

### How much related literature is on PubMed compared to author-supplied citations?

It is not obvious how to precisely answer this question since the relevant connections may be undiscovered [5]. In this paper we are aiming to take a first step at characterizing and quantifying the difference between related literature and the citation graph by using noun phrases from papers on PubMed as search terms to uncover potentially related literature.

Prior studies have offered some examples and anecdotal data.

1. In the late 1980s and 1990s, the field of “literature-based discovery” (LBD) demonstrated that undiscovered edges in medical research literature not only exist, but can form the basis of new medical remedies [6]. The original LBD technique is to search for undiscovered transitive relations among research papers – if it is known that A inhibits B and that B causes C but the relation between A and C is as yet unpublished, then A may be a new cure for C – and to present a short list to a human reviewer, who can vet the validity of a small or manageable number of machine-surfaced and pre-filtered candidate connections. For example, using LBD it was discovered that fish oil is a treatment option for Raynaud's disease [7]
2. A study of the medical research literature has found that supporting and contradictory evidence arrives in waves: papers in medical research have been contradicted once they become highly cited in the literature [8].
3. As online publication has accelerated, a study of 34 million research articles published in Science [9] indicates that both the average number of citations and the diversity of citations may be decreasing as journals go online. One interpretation is that ease of access to web search interfaces is driving greater similarity and consensus in the citations chosen by researchers, compared to the increased diversity or randomness that may have resulted from independent library research in the past when the journal papers were not so easily search-able. (A possible solution may be to offer an option to randomize the ranking of search results on a search engine like PubMed.).

### How can we uncover and expose related literature updates?

When applied to PubMed and the web, search terms derived or extracted from the natural language processing and text analysis of papers (see [10], [11], [12], [13], [14], [15]) are one way to discover literature that is potentially related to a paper. Terminology variants [16] are another way to discover literature in search engines. Although Medical Subject Heading (MeSH) terms are not assigned for many papers on PubMed, when available they are meant to help searchers on PubMed identify similar topics regardless of the actual term variation used in the biomedical literature on PubMed (See <http://www.nlm.nih.gov/mesh/>). They are manually curated terms assigned to papers on PubMed that map term variations and synonyms onto the same term to uncover research referencing the same topic. Since the emergence of the web, web search has also become one of the most widely accepted interfaces for discovery of related literature by researchers. Search engines have mapped related terms in an automatic manner based on search log data ([17], [18]), however sufficiently rare terms used by researchers in new papers may not be present in the logs in sufficient quantities simply because they were not used often enough in search engines therefore may not be picked up.

If the number of related literature updates in a list is too large, then evaluating these candidates becomes too time-consuming. In this paper we propose to restricting attention to the top 20 candidates in any given list, and any candidates after the top 20 are effectively considered a new list. We could have chosen a larger or smaller number, but top twenty results has been shown to be typical of usage on PubMed [19] as “Over 80% of the clicks for abstract views occurred on one of the top 20 citations returned in the result set.”

### How do we benchmark literature discovery and tracking techniques if the scope and nature of the related literature corpus is unknown a priori?

The field of text-retrieval has focused on test queries and expected responses for validating retrieval of information – based on well-defined test datasets (corpora) (http://trec.nist.gov/data.html) for different “tracks” ranging across chemical, enterprise, legal, blogs, web, etc. These incorporate well-known benchmark queries and datasets that are designed for being reproducible, which may not reflect the entire spectrum of relationships that people find to be be useful. To overcome the limitations of relying on analysis of previously characterized texts by continually incorporating new/relevant inputs, modern web search engines incorporated “relevance” based on hyperlinks established in the world wide web, analogous to citations (see “The PageRank Citation Ranking: Bringing Order to the Web” http://ilpubs.stanford.edu:8090/422/1/1999-66.pdf). For related literature, we propose a hybrid between text retrieval and citation ranking to establish the validity of any new technique to generate a list of related literature that are derived from analysis of the paper's text (i.e. any technique not directly incorporating its citations into the list): verify the relevance of any given related literature technique with the presence of author-supplied citations in the list of related citations. If a list of papers generated initially without knowledge of the author-supplied citations passes that test, it can be an indication that some items in the list that are not already cited by the authors may also be relevant as related literature. Conversely, if any given technique to generate related literature rarely or never retrieved any of the author-supplied citations from PubMed that can call it into question.

With that motivation, we make two definitions for an automatically generated list of N related citation candidates for a given paper on PubMed,

1. Overlap: we define there to be “overlap” if the candidate list contains at least one of the citations provided by the authors of the paper, and the number of articles in common to be the “overlapping” set. Articles in the list that are not in the overlapping set are in the “non-overlapping” set.
2. Citation-validated: If the related citation list contains at least one citation from the paper then we define it and its non-overlapping results to be “citation-validated” or CV for short. The validating citations may be by any of the same author as the paper (CV-S) or an entirely different set of authors (CV-D) than the authors of the paper itself.

### How do we interpret the significance of these definitions?

For any given paper, there will likely be many possible lists of related articles that are of primary importance for discovering and tracking related literature, and whose top 20 results do not contain any of the author-supplied citations (no overlap) or are otherwise not CV-S or CV-D. However when a list produced by text-analysis is CV-D or CV-S, it serves as positive indicator of the relevance of non-overlapping items in the list. As a test, it is possible CV-S may simply be caused by the authors' unique use of terminology, whereas CV-D further indicates the overlap in citations is not specific to the authors of the paper.

PubMed's “related citations” is a well known text-analysis technique that is implicitly validated through continued use on PubMed (in 20% of user sessions as noted earlier), and as is often CV-D as well. For “related citations” on PubMed, we show below that for a sample of 883 papers from PubMed, 65% of the top N = 20 PubMed “related citations” sets are CV-D, and the remaining 35% are not. Since “related citations” is a well known technique, this is one way to calibrate our expectations of citation validation.

It is possible that citations may appear in search results by chance which would cast doubt on the usefulness of citation validation as definition. To examine this issue, we ask how likely is a random search with 20 search results from PubMed going to contain at least one citation from the paper, and therefore be CV-D just by chance rather than on its own merits? In general, the top 20 search results of a search term are a small sliver of the Pubmed corpus of N = 18 million citations (See “Yearly Citation Totals from 2010 MEDLINE/PubMed Baseline: 18,502,916 Citations Found” <http://www.nlm.nih.gov/bsd/licensee/2010_stats/2010_Totals.html>). If we can assume for the purposes of evaluating the usefulness of search terms that the top 20 search results for a randomly chosen search term can be modeled as 20 independent document retrievals from the corpus, then for a paper with less than C = 100 citations on Pubmed the probability that a random search results in at least one of these citations in the top 20 search results (i.e. citation validated) is equal to 1−(1−C/N)∧20 by elementary probability. This is approximately equal to 20 C/N<0.02% – that is less than 1 in 5000 searches. So if a search term contains a citation in its top 20 results, that is a statistically improbable outcome which suggests it may be relevant to finding other related papers.

### Why are noun phrases useful to help navigate related literature on PubMed?

To investigate the difference between author-supplied citations and related literature, we use ranked noun phrases (which are composed of sequences of adjectives and/or nouns) and single words extracted from the sentences of a paper (which often end up being nouns and adjectives as well). In contrast, PubMed “related citations” only considers single words and MeSH terms if available which may be multiple words, but not other multi-word sequences or phrases from the sentences in general which may omit several key phrases used by authors.

Noun phrase extraction from sentences has been described in detail elsewhere – for example see Chapter 5 and Table 5.2 of [20] for an explanation of the principles and alternate methods of automated noun phrase extraction. They are well understood technically and universally applicable across all disciplines. Nouns, adjectives, and noun phrases in the paper's text that reflect the subject matter discussed by the author can be useful as search terms on PubMed and the web to discover and keep track of related literature written by other authors. To illustrate what noun phrases are, we include a passage from the paper, Differential expression of anterior gradient gene AGR2 in prostate cancer which has a PubMed ID of 21144054. The underlined words below are examples of noun phrases that were automatically identified as described in the Methods section below:

“AGR2 has been implicated in cancer pathogenesis and has been found to be up-regulated in multiple human cancers, including breast, lung, and prostate. Our study has shown that AGR2 is higher in prostate cancer cells compared to non-malignant prostatic epithelial cells at the transcript and protein levels.”

Due to their ability to describe the subject matter of sentence text without need for any prior knowledge or context, automatically extracted nouns, adjectives, and noun phrases have often been used for discovery of information in medical literature ([21], [22] and also Bennett NA, He Q, Powell K, Schatz BR. Extracting Noun Phrases for all of MEDLINE <http://www.canis.uiuc.edu/archive/papers/AMIAPaper1.html>) and other applications [20]. Co-occurences of different MeSH terms in papers were studied in [23]. The feasibility of using automatically extracted MeSH terms was studied in [24], using noun phrases to assign MeSH terms for papers was studied in [25], and reproduction of manually assigned MeSH terms using automatic methods in [26], and use of natural language processing to complement MeSH terms in [27]. Automatic assignment of MeSH terms for patient medical records was studied in [28] and by using noun phrases in [22]. A technique called TF-IDF was applied to individual tokens to find related literature in [29] and to noun phrases in [30] to automate ontology generation. Amazon.com uses statistically improbable phrases to create search terms for new books (See Statistically Improbable Phrases, <http://www.amazon.com/gp/search-inside/sipshelp.html>). Researchers in social networking areas have investigated the ability to predict social connections from information about the individuals [31].

To compare related literature and citations, we test whether we can use text-analysis techniques to rank noun phrases and identify search terms among these noun phrases whose PubMed searches are CV-D. The universe of searches that can be generated from terms and concepts in the paper can produce a large number of results drawn from trillions of links on the web – only a small fraction of these are likely to be relevant. Therefore we create search terms based on noun phrases for each paper chosen from the nouns, adjectives, and noun phrases in the sentences in the paper, and also rank them based on how frequently they appear in the paper and how infrequently they appear on the web – similar to TF-IDF [10]. This approach is similar in spirit to the approach that relies on how frequently the term occurs in the corpus versus on Medline (instead of the web) as described in equation (1) of [32].

### How can we estimate the size of related literature on PubMed relative to the citation graph?

Unlike PubMed's “related citations” which is a single ranked list – the ranked noun phrases show that there are many different searches for each paper, some of which are also citation-validated. The ranked noun phrases provide one constructive and systematic approach to navigating related literature. If all search results in these searches are valid related literature, then the related literature would be an order of magnitude larger than citation graph. The abundance of noun phrases that we found ranked alongside the phrases we chose also point to potentially vast undiscovered related literature from both PubMed and the web. For example, seven citations included in this paper were discovered by the authors using the ranked noun phrases extracted from earlier drafts of the text as search terms on PubMed and the web. In general, the non-overlapping search results can only be vetted by an interested researcher who knows how to recognize and explain relationships – i.e. by connecting the dots.

### Organization of the paper

The rest of the paper is organized as follows. First we describe our methodology for uncovering potentially related literature on Pubmed based on ranked noun phrases as search terms. For papers on Pubmed whose full-text is available from PubMed Central (PMC), we create search terms for each paper chosen from the nouns, adjectives, and noun phrases in the sentences in the paper, those that tend to occur most frequently in the paper and less frequently on the web. Next, we compare PubMed and web search results using these search terms to citations included by authors of the paper that are available directly from PubMed Central. We find that there is overlap, i.e. reproduction of some of the author-supplied citations. Secondly we discover that in some cases the overlap includes copies of the original paper or papers by the same author, and in other cases papers by different authors cited in the original paper (CV-D). Finally, we provide an estimate to answer the open-question of how many of the non-overlapping search results that are relevant to readers that have not been captured by author-supplied citations?

## Methods

### Nouns, adjectives, and noun phrases from papers as search terms

We test whether ranked noun phrases can expose and discover potentially related literature on a sufficiently representative and recent sample of the medical literature. The medical literature is vast and described by over 26,000 Medical Subject Heading (MeSH) terms ([33] and also see “Fact Sheet, Medical Subject Headings” <http://www.nlm.nih.gov/pubs/factsheets/mesh.html>). PubMed Central makes the text of open-access papers and the Pubmed links to the author-supplied citations available online (http://www.ncbi.nlm.nih.gov/pmc/). PubMed Central contains nearly 2 million open-access articles from several hundred journals, most of which are cross-listed on Pubmed (See “What is the connection between PubMed Central and PubMed?” http://www.ncbi.nlm.nih.gov/pmc/about/faq.html#q8).

PubMed Central IDs are not sequential and therefore not amenable to random sampling of recent literature, and a list of most recent papers was not otherwise available. To select a representative sample from the recent research literature on PubMed Central, during the week of April 18, 2011 we searched for “research,” then ordered the results by publication date, and took the top 1000 search results. In order to perform the citation validation test, we filtered out the papers with less than 5 citations which left us with 883 papers. For each of these papers on PubMed Central, we retrieved its citations from PubMed Central, PubMed's “related citation” list, and selected search terms from each paper to run queries on Pubmed abstracts and titles using the “Entrez Utilities Entrez Programming Utilities” <http://eutils.ncbi.nlm.nih.gov/>). For example the paper, Differential expression of anterior gradient gene AGR2 in prostate cancer has a PubMed Central identifier of PMC3009682. There were 46 author-supplied citations on PubMed, and the top 20 PubMed related citations included 4 overlapping citations by different authors. Searches on all search terms are run in quotes for phrase matching such as “anterior gradient” versus simply anterior gradient.

To extract noun phrases to use as search terms for each of the papers on PubMed Central, we run a software program written in Python based NLTK (http://www.nltk.org/) to automatically extract nouns, adjectives, and noun phrases from each sentence in the paper. NLTK is described in [11]. The theory of noun phrase extraction has been described in [22] and [20], and noun phrase extraction is available in commercial software packages such as Attivio (See <http://www.attivio.com/active-intelligence/aie-features/aie-language-processing.html>) and Inxight (See <http://www.inxightfedsys.com/pdfs/LinguistX_FinalWeb.pdf>).

Starting with the text of a paper, the steps to extract noun phrases comprise separate software modules for,

1. extracting the author's written text of the paper from the native format (PDF, HTML, etc)
2. splitting the text into well-formed sentences (sentence tokenization) and words (word tokenization) which involves correctly recognizing punctuation and word boundaries (See “Package tokenize” <http://nltk.googlecode.com/svn/trunk/doc/api/nltk.tokenize-module.html>)
3. identifying the part of speech of each word in each sentence using a part of speech tagger such as the Brill tagger. (See “Module brill” <http://nltk.googlecode.com/svn/trunk/doc/api/nltk.tag.brill-module.html>). Note that part-of-speech tagging may sometimes involve classification errors such as mistagging a noun for a verb, etc. The tagging accuracy is typically in the range of 90–97% (see “Tagging Accuracy” on pages 371–373 of [20]) but depends heavily on the corpus and tagger.
4. selecting single word nouns (N), adjectives (A), and multi-word noun phrases using patterns such as AN, NN, etc. For example “blue sky” and “house boat.” Note the tagging errors introduced in the previous step may carry over to induce mis-identifcation of noun phrases in this step, which is why we may occasionally observe phrases that are not exactly noun phrases appearing below.

For a paper, there are typically hundreds if not thousands of noun phrases depending on its length, and all combinations of these phrases are not possible to search for. To select the most representative terms, we rank them based on the number of occurrences of the term in the paper itself (document count) and inversely to the number of occurrences of that same term on the web (web count) obtained using the Yahoo-BOSS API (http://developer.yahoo.com/search/boss/). The greater this ratio the more significant the phrase is likely to be. An alternative to web counts would be to use the same counts obtained from the PubMed corpus, although the rate of search queries is limited by PubMed making it harder to utilize these counts compared to Yahoo-BOSS which permitted several searches per second. In contrast, PubMed “related citations” incorporates the count of the term within PubMed instead of the web.

**Figure 1:**
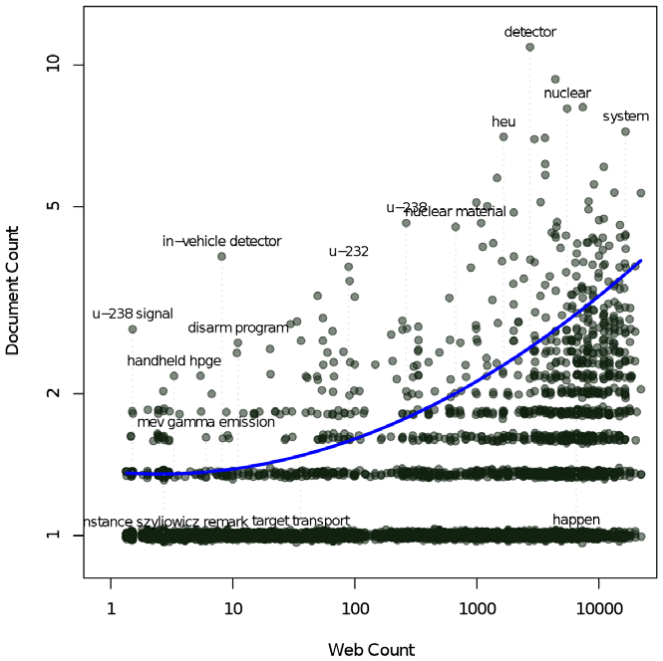
Document and web counts for phrases appearing in an example paper about nuclear detection technologies. The axes are on a log-scale; a quantile regression curve runs through the scatterplot. Phrases that most representative of the document's contents are along the upper boundary; they have high document count for their web count. Less relevant phrases fall below.

To illustrate the concept of document and web counts, Figure 1 shows a plot of these counts for an example paper on detection of highly enriched uranium [34] written by one of the authors – with each dot representing one phrase. Each data point on the plot shows the document count and web count on the x-axis and y-axis respectively on a log-scale. Using the log-scale, phrases that represent the document's subject tend to stand out based on a large document count and smaller web count, such as “in-vehicle detectors,” “nuclear material,” “u-232” and “u-283 signal.” The vast majority of phrases tend to be at the bottom with document count equal to 1. As the web count gets larger, it takes a larger document count for a phrase to be more representative of the paper's subject matter.

After computing the ordered pairs of the document count and web count for each extracted phrase, the phrases need to be ranked in order to find the ones that best reflect the subject matter of the paper. We use the procedure described below based on regression. The document count and web count are converted to logarithms and a curve is fitted to the ordered pairs using quantile regression [35] as illustrated in Figure 1 with the blue line. Alternate variations of this curve fit are feasible such as a linear fit or quadratic fit. For each phrase, the numeric difference between the document count of the phrase and the value of the regression function (fitted curve) evaluated at the web count for that phrase is used to rank order the phrases. For example if the regression is y = mx+c and the document count is y', then the difference is y'-mx-c. We empirically observe that the more positive the difference between the document count and the value of the regression, the more of an outlier the phrase is relative to other phrases with similar web counts, and therefore the greater its relevance to the subject matter expressed in the document. In practice, we have found that the ranking algorithm described produces results comparable to the well-known TF-IDF algorithm [10] which computes a score using each ordered pair without the need for regression. The TF-IDF score is proportional to the document count and inversely proportional to the logarithm of the web count.

**Figure 2:**
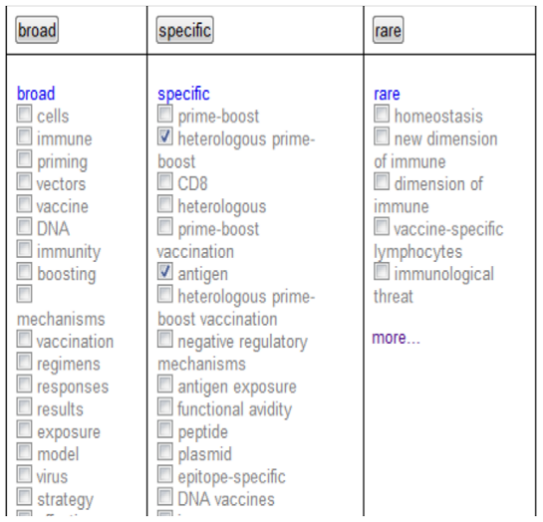
The ranking of noun phrases for the paper, “Programmed cell death-1 (PD-1) at the heart of heterologous prime-boost vaccines and regulation of CD8+ T cell immunity.” We rank them based on the number of occurrences of the term in the paper itself (document count) and inversely to the number of occurrences of that same term on the web (web count). To visually aid in phrase selection the ranked phrases are further split into three columns while maintaining the ranking in each column: those which occur on the web more than 10 million times (broad), those which occur between 100 and 10 million times (specific), and those which occur less than 100 times (rare).

Figure 2 shows the ranking of phrases we used for the paper, “Programmed cell death-1 (PD-1) at the heart of heterologous prime-boost vaccines and regulation of CD8+ T cell immunity.” To visually aid in phrase selection the ranked phrases are further split into three columns while maintaining the ranking in each column: those which occur on the web more than 10 million times (broad), those which occur between 100 and 10 million times (specific), and those which occur less than 100 times (rare). In the figure, representing our test interface, we interactively select phrases (shown as a tick) to invoke a PubMed search with those phrases and ‘more’ simply opens up more ranked phrases further down in the ranking.

Each combination of phrases results in a valid search that reveals different information about what is present in the Pubmed corpus. Many combinations of the ranked phrases can be readily produced for each paper. For the search terms we derive from each paper, we compared the top 20 search results to the author-supplied citations for that paper. We defined an “overlap” if a search result was from Pubmed and its respective ID matched or reproduced any of the author-supplied citations obtained in the paper itself. The author supplied citations are obtained directly from the HTML of the paper itself, and automatically compared to the search results for each search run to measure overlap.

To select search terms automatically, we can select combinations of phrases from the set of all possible combinations of the top ranked phrases. In general for N top ranked terms, the number of combinations of k terms (N choose k) grows approximately as N∧k. Even for a computer, to execute this many searches would be prohibitive as N grows beyond several dozen with fixed k = 2 or 3. We try the top 20 ranked “specific” and “rare” phrases (k = 1) to generate search terms and discover citation validated search terms. For example, we show the automatic search terms for the paper mentioned above whose ID is PMC3009682 and title is “Differential expression of anterior gradient gene AGR2 in prostate cancer.” The citation validated search terms that were automatically generated are listed below, followed by the number of results on PubMed (ranging from 3 to 127 results) and a list of PubMed IDs in square brackets that were the author supplied citations appearing in the search results. “D” indicates they are by different authors, and “S” indicates if they are by authors of the paper itself.

- “laevis cement gland” (4 results) D [‘10095068’, ‘9790916’]
- “Xenopus laevis cement gland” (4 results) D [‘10095068’, ‘9790916’]
- “AGR2 promotes cell” (7 results) D [‘20048076’, ‘18199544’]
- “AGR2” (70 results) D [‘20945500’]
- “XAG-2” (9 results) D [‘15834940’, ‘14967811’, ‘10095068’, ‘9790916’, ‘9533957’]
- “AGR2 expression” (15 results) D [‘20048076’, ‘18681322’, ‘18199544’, ‘17457305’, ‘17455144’, ‘16551856’]
- “Xenopus laevis cement” (5 results) D [‘10095068’, ‘9790916’]
- “gene XAG-2” (3 results) D [‘9790916’, ‘9533957’]
- “hAG-2” (6 results) D [‘12592373’, ‘9790916’]
- “cement gland gene XAG-2” (4 results) D [‘10095068’, ‘9790916’, ‘9533957’]
- “gland gene XAG-2” (5 results) D [‘15834940’, ‘10095068’, ‘9790916’, ‘9533957’]
- “PIN lesions” (127 results) D [‘20945500’]
- “laevis cement gland gene” (70 results) D [‘15867376’]
- “levels of AGR2” (17 results) D [‘20945500’, ‘20048076’, ‘18973922’, ‘17694278’, ‘17457305’],
- “laevis cement” (118 results) D [‘15867376’]
- “lower levels of AGR2” (3 results) S [‘21144054’]

To choose one example from this list, the search term “AGR2 expression” shows 15 results on PubMed with six of the results being author supplied citations. Is there potentially related literature among any of the remaining nine search results (copied below) that are not cited, and if so what is the relation? In each case only an interested researcher can determine their relevance to the paper as related literature.

1. The human adenocarcinoma-associated gene, AGR2, induces expression of amphiregulin through hippo pathway co-activator YAP1 activation. Dong A, Gupta A, Pai RK, Tun M, Lowe AW. J Biol Chem. 2011 Mar 26; http://www.ncbi.nlm.nih.gov/pubmed/21454516/
2. Differential expression of the anterior gradient protein-2 is a conserved feature during morphogenesis and carcinogenesis of the biliary tree. Lepreux S, Bioulac-Sage P, Chevet E. Liver Int. 2011 Mar;31(3):322–8 http://www.ncbi.nlm.nih.gov/pubmed/21281432/
3. The pro-metastatic protein anterior gradient-2 predicts poor prognosis in tamoxifen-treated breast cancers. Hrstka R, Nenutil R, Fourtouna A, Maslon MM, Naughton C, Langdon S, Murray E, Larionov A, Petrakova K, Muller P, Dixon MJ, Hupp TR, Vojtesek B. Oncogene. 2010 Aug 26;29(34):4838–47 http://www.ncbi.nlm.nih.gov/pubmed/20531310/
4. Anterior gradient-2 plays a critical role in breast cancer cell growth and survival by modulating cyclin D1, estrogen receptor-alpha and survivin. Vanderlaag KE, Hudak S, Bald L, Fayadat-Dilman L, Sathe M, Grein J, Janatpour MJ. Breast Cancer Res. 2010;12(3):R32 http://www.ncbi.nlm.nih.gov/pubmed/20525379/
5. Disruption of Paneth and goblet cell homeostasis and increased endoplasmic reticulum stress in Agr2−/− mice. Zhao F, Edwards R, Dizon D, Afrasiabi K, Mastroianni JR, Geyfman M, Ouellette AJ, Andersen B, Lipkin SM. Dev Biol. 2010 Feb 15;338(2):270–9 http://www.ncbi.nlm.nih.gov/pubmed/20025862/
6. Identification of candidate biomarkers of therapeutic response to docetaxel by proteomic profiling. Zhao L, Lee BY, Brown DA, Molloy MP, Marx GM, Pavlakis N, Boyer MJ, Stockler MR, Kaplan W, Breit SN, Sutherland RL, Henshall SM, Horvath LG. Cancer Res. 2009 Oct 1;69(19):7696–703 http://www.ncbi.nlm.nih.gov/pubmed/19773444/
7. Anterior gradient 2 is expressed and secreted during the development of pancreatic cancer and promotes cancer cell survival. Ramachandran V, Arumugam T, Wang H, Logsdon CD. Cancer Res. 2008 Oct 1;68(19):7811–8 http://www.ncbi.nlm.nih.gov/pubmed/18829536/
8. Sequence and expression of Drosophila Antigen 5-related 2, a new member of the CAP gene family. Megraw T, Kaufman TC, Kovalick GE. Gene. 1998 Nov 19;222(2):297–304 http://www.ncbi.nlm.nih.gov/pubmed/9831665/

## Results

The data for each of the 883 papers was recorded in supplementary information tables

- Table S1 contains the measurements for PubMed's “related citations”
- Table S2 contains the measurements for citation validated searches.

The results are summarized in “Table 1: PubMed “related citations” versus ranked noun phrases.” For a sample of 883 papers, search terms for 86% (98%) of the papers were validated by citations written by different authors than the paper (or the same authors) versus an equivalent of 65% (99%) for the top 20 PubMed “related citations” – higher indicates greater validation by citations.

**Table 1:** PubMed “related citations” versus ranked noun phrases.

Measurements for 883 papers	PubMed “related citations”	Using search terms generated from ranked noun phrases
Papers validated by citations from different authors (CV-D)	65% (61–68% at 95% confidence)	86% (83–88% at 95% confidence)
Number of search terms that are CV-D – mean and standard deviation across papers	n/a	5.1 (+/−4.5 standard deviation)
Papers validated by citations from the original authors (CV-S)	99% (98–100% at 95% confidence)	98% (96–99% at 95% confidence)
Number of search terms that are CV-S (mean and standard deviation across papers)	n/a	10.3 (+/−6.1 standard deviation)
Unique validating citations (mean and standard deviation across papers)	1.7 (+/−1.9)	5.7 (+/−3.7 standard deviation)
Papers with at least one CV-S or CV-D search term with less than 5 search results (excluding validating citations)	n/a	64% (60–67% at 95% confidence)
Papers with at least one CV-S or CV-D search term with less than 20 search results (excluding validating citations)	n/a	92% (90–94% at 95% confidence)
Papers with at least one CV-S or CV-D search term with more than 20 search results (excluding validating citations)	n/a	98% (96–99% at 95% confidence)

On average across all 883 papers, out of a maximum of 40 possible (20 specific+20 rare), 15 search terms per paper were citation validated and 5 search terms were validated by citations written by different authors, versus 10 by the same authors as the paper itself. For the search terms, on average 6 unique citations validated the searches versus 1.7 citations for PubMed “related citations.” The number of search results per search varies widely: 64% of these papers have at least one search term with under 5 search results on PubMed, 92% with at least one under 20 search results, and 98% with at least one over 20.

**Figure 3:**
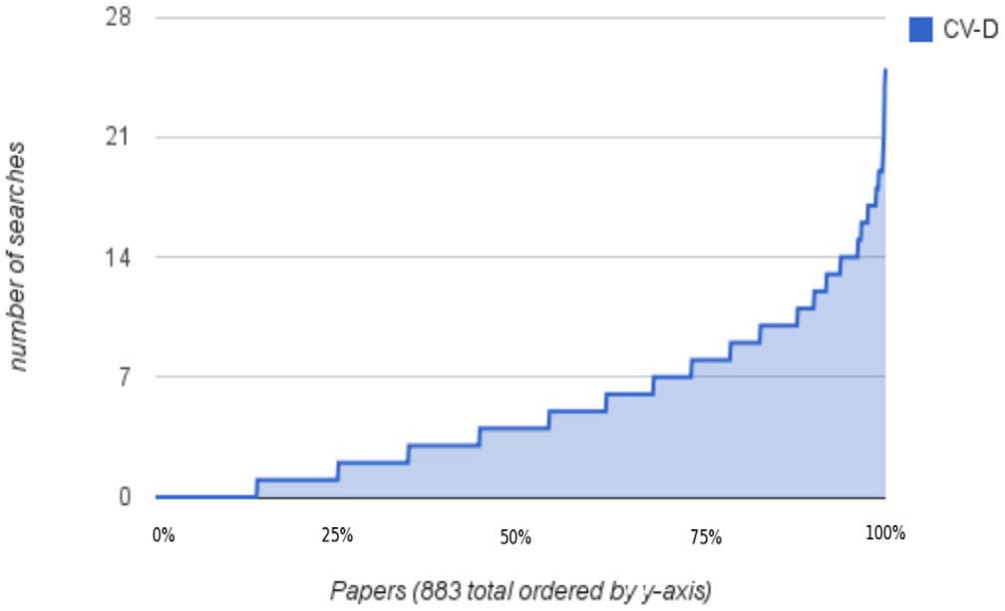
Searches validated by citations by different authors. For 883 papers, this figure shows the number of PubMed search terms per paper which are validated by citations from different authors (CV-D), which ranges from 0 to well over 20 in some cases out of a maximum possible of 40 (20 from each of the specific and rare lists). The search results which are not author-supplied citations in these CV-D searches can in turn be used to suggest related literature for the paper.

**Figure 4:**
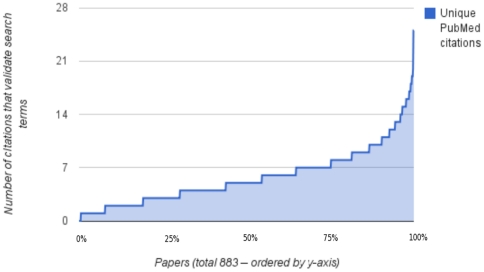
Citations that validate the search terms for each paper. For 883 papers, this figure shows the total number of different citations from the paper that validate all the search terms for each paper. Each paper may have multiple search terms whose top 20 search results are validated by citations. This number ranges from 0 to well over 20 citations across the papers.

**Figure 5:**
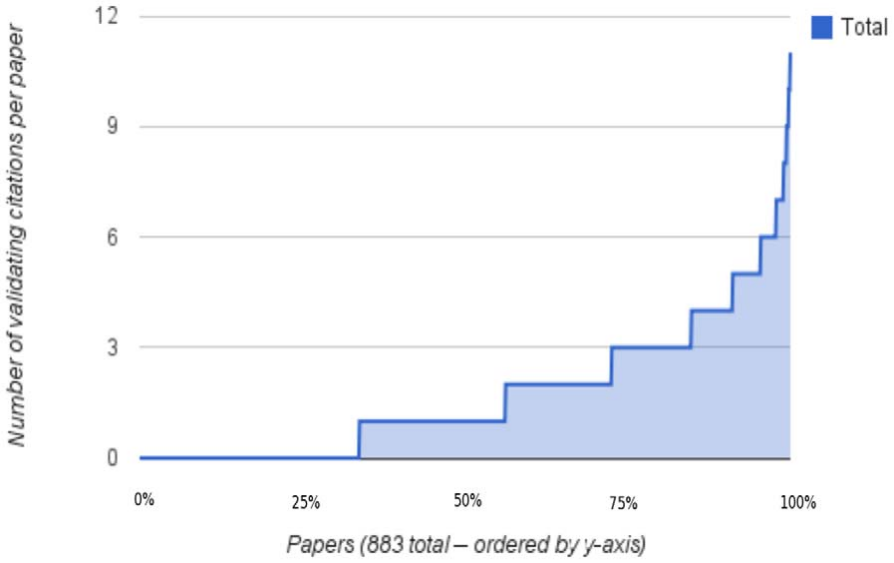
Validating citations per paper for PubMed's “related citations”. For 883 papers, this figure shows the total number of different citations from the paper that validate the top 20 “related citations” from PubMed for each paper. This number ranges from 0 to well over 20 citations across the papers. This number ranges from 0 to well over 10 citations across the papers.

**Figure 6:**
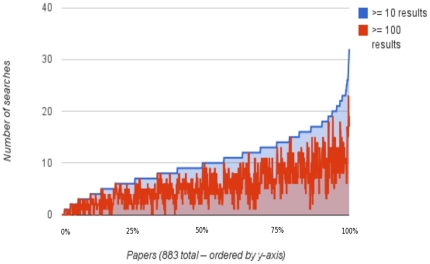
Non-overlapping search results. For 883 papers, the figure shows how many different search terms have more than 10 and more than 100 search results. Each of the search results that are not already citations may be potentially related literature, whose relationship is indicated via the search term.

Figure 3 shows the distribution of number of search terms validated by citations from different authors – CV-D. The top half of this distribution offers many more searches than the average of 5, and well over 15 in many cases. Figure 4 shows the distribution of number of validating citations for each paper. Given the choice of search terms, the number can easily exceed 10 different citations per paper. Figure 5 is the same as Figure 4 except for PubMed's related citations. This shows that the number of validating citations per paper was much lower for “related articles” than for the search terms, both on average and maximum. Figure 6 illustrates the number of search terms for each paper whose non-overlapping search results greater than 10 and 100 respectively. For most papers there were several searches with both over ten and 100 search results, indicating a potentially vast related literature that may not have been reviewed by readers of the paper. On the examples that the authors are familiar with, we have verified the relevance of some of these search results. Without nuanced review by experts, it is hard to make a statement about their relevance across the entire sample of 883 papers and generalize to PubMed.

While the non-overlapping search results for Pubmed may be suggestive, we could not make a more definite statement about their relevance without verification by someone informed about the topic. In the authors' personal tests with papers they are familiar with, we have observed the non-overlapping results often to be relevant including drafts of this paper as well as the text of [34]. Citation validation further confirms the relevance. Computers can aid in suggesting relevant searches for a paper, only informed researchers can ultimately determine the relevance of a paper as related literature.

## Discussion

We investigated if nouns, adjectives, and noun phrases that are part of the terminology used by authors in their papers are useful as search terms to discover and keep track of related literature written by other authors. We tested whether the top 20 search results contain some of the citations in the paper, not only by the same authors (CV-S) but by different sets of authors (CV-D). If the search terms were not relevant, we would not expect to see any of the author-supplied citations in the top 20 search results.

Starting with a systematic sample of the most recent 883 papers with more than 5 author-supplied citations on Pubmed Central obtained by searching for “research,” we were able to reproduce author-supplied citations using hand-selected search terms in 86% of the cases (95% confidence interval is 83%–88%) using citations written by different authors (CV-D), or 98% (95% confidence interval is 96%–99%) if we include the same authors.

Can we generalize these percentages to the PubMed corpus which on the order of 20 million papers? If in turn recent papers on PubMed Central are representative of the entire PubMed Central and PubMed corpora across time, then we can generalize these results to within 5% on PubMed – which we could verify if we were to get access to their full-text and citations. It's possible, that older papers may have slightly different properties than more recent papers. For example, there may be more related work accumulated over time, or that citations in very old papers may not appear as ranked higher by searches because PubMed search results are ordered by date. Other competing factors may be at play as well.

The reproduced citations help validate the relevance of the nouns, adjectives, and noun phrases as multiple options for search terms for related literature on Pubmed. Since there are multiple citation-validated searches with 5–20 search results for most papers, the potentially related literature uncovered through citation-validated searches is on the order of ten citations per paper – likely many more when the search terms that are not citation-validated are also considered. We cannot state with certainty that the literature is definitely “related” or not without expert review on a case by case basis, however the existence of citation validated search terms is a strong indication.

Anecdotally, we have verified in multiple instances hat the phrases generated by the methods described above work well with authors' own papers and searches to uncover related literature. In the future, we plan to further verify the approach of using citation-validated search terms by surveying authors about the relevance of related literature generated using this approach.

It may be possible to generate several more CV-D searches that complement the noun phrases we used and improve on the number of validating citations papers in the bottom half of the distribution of Figures 3 and 4 in at least two different ways:

1. by applying alternate natural language techniques beyond noun phrases such as term variations, synonyms, and search terms related through search logs.
2. by selecting for noun phrases that appear in both the paper and its citations.

The lower level of citation validation in Figure 5 for PubMed “related citations” compared to the search terms does not prove that the “related citations” are any less or more related to the paper than the search terms. Typically the “related citations” are hundreds of results long. If we considered more than 20 “related citations” and search results, we might observe more overlap with the paper's citations. However the navigational advantage of the search terms is they provide a phrase connecting the list of results to the paper, versus PubMed “related citations” which provides no indication for why any of the results are related.

MeSH terms were not available for many papers we sampled. When hand-curated MeSH terms are not available for an article, using automatically generated search phrases can be a useful substitute or fall-back to facilitate the discovery of related literature.

PubMed “related citations” were available in 100% of the cases. The reason why they are related (terms in common, etc) is not clearly part of the display of each related article. PubMed's “related citations” are presented as a ranked list with no specific explanation for each result in the list, whereas the search term that brings up potentially related literature explains more about how and why its search results may be related to the paper. Instead of one list, our results point to the existence of many potentially undiscovered lists of related literature, one for each ranked noun phrase, that in aggregate can be an alternative to PubMed's “related citations” annotated by the search terms and their validating citations. The non-overlapping search results are several times the size of the overlapping set. While the non-overlapping, CV-D results for Pubmed are suggestive, we cannot make a definite statement about the relevance of any of the non-overlapping search results without verification and explanation by informed researchers in the field who understand the given paper and can assess the relevance of any result.

Although computers can aid in suggesting searches that might be relevant to a paper, only informed researchers – not necessarily the authors themselves – can ultimately determine if potentially related literature discovered using noun phrase search terms, PubMed “related citations,” or other techniques deserves to be called related literature. As researchers we can collaborate to uncover and navigate related literature – especially connections that would not otherwise be obvious – by sharing related work, explaining their relationships, and exposing the search terms used to discover them. This includes newer research, summaries, background and foundational work, terminology variants, competitive research, and links to closed-access publications.

One framework representative of many characteristics that can enable collaboration is the Citation Typing Ontology (CiTO) [36]. In one of its many features, CiTO calls on authors [37] to replace ordinary hyperlinks with a “typed” hyperlink,

paper A (http://A)
  - summarizes
  - contradicts
  - agrees with
  - cites
  - etc
paper B (http://B)

Labels like the ones proposed in CiTO may be useful for describing literature relations by researchers reading a paper, in addition to authors. They need not be fixed, and can be expanded to including other types such as “summarizes,” “terminology variant,” “foundational,” etc. as needed by researchers.

Some sharing of related literature for research topics goes on in small research groups using email and in person communication. As researchers, we also find out about related work through colleagues and friends, citation management software, search engines, MeSH terms and related article searches on PubMed, blogs, social networks, Wikipedia, etc. Results of individual research using services such as PubMed, citation indices, and library resources often become inaccessible as researchers may file away related literature relationships or forget about them. Ongoing efforts of researchers to identify important related research articles do not translate directly to helping other researchers working across the world due to lack of a well-known place to save and access them permanently. For example, PubMed “interact” included a feature to enable researchers to add related articles to PubMed [38] although it does not appear to enable collaboration by making these additions publicly visible to all others.

To navigate and and keep track of related literature updates, the next generation of search engines needs to go beyond “related citations” to help navigate the connections that individual researchers discover while reading and understanding papers. This will accelerate the dissemination of research knowledge, to broaden the exposure of researchers to literature in subject areas outside of their expertise, expose new researchers to milestone papers, and eliminate the inefficient cycle of discovery and rediscovery of related literature.

## Supporting Information

Table S1 **Measurements for PubMed's “related citations”.** This table lists each of the 883 papers by Pubmed Central identifier with PubMed “related citations” that are also author-supplied citations (CV-D and CV-S). The data in this table was used in Figure 5.
(PDF)

Table S2 **Measurements for citation validated searches.** This table lists each of the 883 papers by Pubmed Central identifier with citation-validated search terms (CV-D and CV-S). The data in this table was used in Figures 3, 4, and 6.
(PDF)
