# Supplementary material for: Using Noun Phrases for Navigating Biomedical Literature on Pubmed: How Many Updates Are We Losing Track of?
Source: PLoS One. 2011 Sep 14;6(9):e24920. doi: 10.1371/journal.pone.0024920 (PMC3173492; doi:10.1371/journal.pone.0024920)
Supplement: Table S1 — Measurements for PubMed's “related citations”. This table lists each of the 883 papers by Pubmed Central identifier with PubMed “related citations” that are also author-supplied citations (CV-D and CV-S). The data in this table was used in Figure 5. (PDF) [file pone.0024920.s001.pdf]

PMC ID	CV-D	CV-D > 0	CV-S	CV-S > 0		Total	PubMed citations
PMC3072	2	FALSE	1	FALSE	301	2	D ['18573856', '16621883'], S ['21474616']
PMC3077	5	FALSE	1	FALSE	7	5	D ['17050571', '20360059', '19958517', '16982707', '12386112'], S ['15905486']
PMC3063	1	FALSE	2	FALSE		1	D ['15836924'], S ['10794718', '21237154']
PMC3062	2	FALSE	4	FALSE		2	D ['8596916', '16919418'], S ['20339857', '21315377', '17443334', '19727777']
PMC3077	0	TRUE	2	FALSE		0	S ['19429891', '21493661']
PMC3078	0	TRUE	2	FALSE		0	S ['14707273', '21487065']
PMC3077	0	TRUE	1	FALSE		0	S ['21499448']
PMC3078	1	FALSE	1	FALSE		1	D ['16984658'], S ['21486880']
PMC3073	1	FALSE	3	FALSE		1	D ['18318825'], S ['17501764', '21494570', '16255762']
PMC3073	0	TRUE	1	FALSE		0	S ['21494569']
PMC3073	5	FALSE	1	FALSE		5	D ['11692306', '14600521', '17578788', '18025882', '18090299'], S ['21494566']
PMC3073	3	FALSE	3	FALSE		3	D ['17600128', '12574396', '16500656'], S ['21494565', '18665903', '10898501']
PMC3073	4	FALSE	3	FALSE		4	D ['16868023', '19299562', '16533945', '16533944'], S ['21494564', '16030258', '19124
PMC3073	0	TRUE	1	FALSE		0	S ['21494563']
PMC3073	0	TRUE	1	FALSE		0	S ['21494562']
PMC3073	0	TRUE	1	FALSE		0	S ['21494561']
PMC3073	3	FALSE	1	FALSE		3	D ['3060351', '15451554', '3060354'], S ['21494558']
PMC3073	1	FALSE	1	FALSE		1	D ['10070366'], S ['21494557']
PMC3073	3	FALSE	3	FALSE		3	D ['9467005', '11241493', '7842019'], S ['10739752', '21494555', '8981948']
PMC3073	3	FALSE	3	FALSE		3	D ['9467005', '11241493', '7842019'], S ['10739752', '21494555', '8981948']
PMC3073	4	FALSE	1	FALSE		4	D ['19325882', '15302899', '21179567', '16819969'], S ['21494554']
PMC3073	4	FALSE	1	FALSE		4	D ['19917133', '18501983', '19837076', '16202143'], S ['21494551']
PMC3073	7	FALSE	3	FALSE		7	D ['19234154', '17675517', '19553541', '19841166', '17407196', '15210768', '11359817 ['10510079', '11207266', '21494550']
PMC3073	2	FALSE	5	FALSE		2	D ['17227772', '16339923'], S ['16337959', '19098112', '17208994', '16970929', '21494
PMC3073	3	FALSE	1	FALSE		3	D ['17166185', '18160750', '19345831'], S ['21494548']
PMC3073	0	TRUE	3	FALSE		0	S ['21494547', '17885684', '19237507']
PMC3073	2	FALSE	1	FALSE		2	D ['17292629', '20435024'], S ['21494606']
PMC3073	3	FALSE	1	FALSE		3	D ['18765899', '20954199', '15621241'], S ['21494605']
PMC3073	1	FALSE	1	FALSE		1	D ['18410405'], S ['21494603']
PMC3073	3	FALSE	1	FALSE		3	D ['20231244', '18344283', '20931334'], S ['21494602']
PMC3073	2	FALSE	2	FALSE		2	D ['11741199', '9988500'], S ['21494601', '18652651']
PMC3073	1	FALSE	1	FALSE		1	D ['19925809'], S ['21494600']
PMC3073	0	TRUE	2	FALSE		0	S ['21494598', '18784466']
PMC3073	0	TRUE	1	FALSE		0	S ['21494597']
PMC3073	0	TRUE	1	FALSE		0	S ['21494596']
PMC3073	4	FALSE	5	FALSE		4	D ['17986185', '11972783', '15686567', '10231493'], S ['9419339', '16313620', '214945 '11930012', '18723629']
PMC3073	2	FALSE	1	FALSE		2	D ['16684787', '20079853'], S ['21494594']
PMC3073	0	TRUE	3	FALSE		0	S ['21494593', '12403771', '17939115']
PMC3073	1	FALSE	2	FALSE		1	D ['16980375'], S ['21494592', '16262900']

608']
'], S
549']
95',

PMC3073	0	TRUE	2	FALSE		0	S ['21494591', '20038636']
PMC3057	5	FALSE	9	FALSE		5	D ['17488792', '20203099', '19318730', '11038323', '17656604'], S ['21078366', '17694<br>'19439508', '16684822', '17200175', '18987494', '17940071', '19246354', '20595937']
PMC3060	1	FALSE	1	FALSE		1	D ['19850388'], S ['21291964']
PMC3072	0	TRUE	2	FALSE		0	S ['21478484', '18996890']
PMC3073	6	FALSE	1	FALSE		6	D ['12611913', '9325351', '7751937', '17086072', '19118109', '19232534'], S ['2149457
PMC3073	0	TRUE	1	FALSE		0	S ['21494577']
PMC3073	1	FALSE	1	FALSE		1	D ['8239177'], S ['21494635']
PMC3073	3	FALSE	3	FALSE		3	D ['20403166', '20492644', '18923008'], S ['18367574', '21494634', '20231882']
PMC3072	1	FALSE	5	FALSE		1	D ['11015814'], S ['21184587', '19923875', '21494633', '19124689', '18040986']
PMC3072	2	FALSE	1	FALSE		2	D ['17824427', '17960243'], S ['21494629']
PMC3072	2	FALSE	8	FALSE		2	D ['15719223', '11978855'], S ['12867266', '18096321', '19913195', '15936001', '11744<br>'20028362', '20021565', '21494628']
PMC3072	3	FALSE	1	FALSE		3	D ['17919705', '12676609', '17142054'], S ['21494627']
PMC3072	6	FALSE	1	FALSE		6	D ['7853416', '10980121', '7696855', '12488479', '18202187', '11044214'], S ['2149462
PMC3072	1	FALSE	1	FALSE		1	D ['8860885'], S ['21494624']
PMC3072	0	TRUE	3	FALSE		0	S ['11964379', '21494623', '20099144']
PMC3072	0	TRUE	1	FALSE		0	S ['21494620']
PMC3072	4	FALSE	2	FALSE		4	D ['10439069', '17541801', '15201052', '20007325'], S ['19946376', '21494619']
PMC3072	1	FALSE	3	FALSE		1	D ['19890055'], S ['14597100', '11559811', '21494618']
PMC3072	2	FALSE	1	FALSE		2	D ['20875187', '8761578'], S ['21494617']
PMC3072	2	FALSE	2	FALSE		2	D ['20459604', '16132956'], S ['19477545', '21494616']
PMC3072	2	FALSE	4	FALSE		2	D ['14981738', '12422305'], S ['19164207', '15880395', '19745023', '21494615']
PMC3072	6	FALSE	1	FALSE		6	D ['20208569', '15221014', '17723183', '18322270', '18283316', '16266997'], S ['21494
PMC3072	3	FALSE	2	FALSE		3	D ['7578849', '8454115', '8904930'], S ['18184926', '21494613']
PMC3072	1	FALSE	2	FALSE		1	D ['15764667'], S ['20532636', '21494612']
PMC3072	1	FALSE	2	FALSE		1	D ['10434004'], S ['21494611', '20686703']
PMC3072	0	TRUE	1	FALSE		0	S ['21494607']
PMC3072	6	FALSE	1	FALSE		6	D ['17276347', '16989804', '4038667', '19076067', '16782018', '16452714'], S ['214946
PMC3072	7	FALSE	1	FALSE		7	D ['18583159', '16762568', '8087187', '15579383', '15288059', '19597161', '12066683']<br>['21494664']
PMC3072	0	TRUE	1	FALSE		0	S ['21494662']
PMC3072	0	TRUE	2	FALSE		0	S ['21494660', '20572128']
PMC3072	3	FALSE	1	FALSE		3	D ['18825432', '18056739', '17938370'], S ['21494659']
PMC3072	3	FALSE	2	FALSE		3	D ['18818769', '17881571', '20562860'], S ['18216267', '21494658']
PMC3072	3	FALSE	1	FALSE		3	D ['17363583', '20052266', '20544847'], S ['21494657']
PMC3072	3	FALSE	1	FALSE		3	D ['19171079', '17349105', '16887199'], S ['21494656']
PMC3072	3	FALSE	1	FALSE		3	D ['11169610', '19268668', '18466326'], S ['21382016']
PMC3072	0	TRUE	1	FALSE		0	S ['21382015']
PMC3072	1	FALSE	4	FALSE		1	D ['19471109'], S ['17883393', '19776077', '21395555', '16219035']
PMC3078	2	FALSE	1	FALSE		2	D ['20336144', '18160057'], S ['21473762']
PMC3072	0	TRUE	2	FALSE		0	S ['21474525', '18483352']

559',
8']
078',
6']
614']
65']
, S

PMC3072	0	TRUE	1	FALSE		0	S ['21474517']
PMC3072	3	FALSE	1	FALSE		3	D ['17965431', '16246909', '16556315'], S ['21474551']
PMC3076	0	TRUE	1	FALSE		0	S ['21494431']
PMC3072	1	FALSE	2	FALSE		1	D ['19879142'], S ['9335503', '21490725']
PMC3072	3	FALSE	1	FALSE		3	D ['18614027', '15721797', '11466447'], S ['21490724']
PMC3072	0	TRUE	3	FALSE		0	S ['21490722', '17029556', '19283073']
PMC3072	2	FALSE	2	FALSE		2	D ['18632555', '16315107'], S ['21490719', '18675489']
PMC3072	5	FALSE	2	FALSE		5	D ['16236758', '12783845', '18923150', '17353360', '11773003'], S ['21490958', '19464
PMC3072	5	FALSE	2	FALSE		5	D ['19164517', '19160498', '12077604', '15175387', '15746429'], S ['21490957', '16926
PMC3072	2	FALSE	3	FALSE		2	D ['11230149', '11046137'], S ['18845848', '21490951', '18828915']
PMC3072	2	FALSE	2	FALSE		2	D ['12514735', '12239239'], S ['21490950', '19297529']
PMC3072	6	FALSE	4	FALSE		6	D ['18597214', '20550665', '18633108', '19670153', '20509872', '19862325'], S ['19892 '21490949', '18372903', '17463248']
PMC3072	1	FALSE	5	FALSE		1	D ['17597762'], S ['17660198', '21490948', '19109897', '18497825', '20519324']
PMC3071	1	FALSE	2	FALSE		1	D ['19961320'], S ['17616786', '21490707']
PMC3072	4	FALSE	1	FALSE		4	D ['18492804', '15237602', '18563898', '21174459'], S ['21490938']
PMC3072	3	FALSE	1	FALSE		3	D ['17535961', '17919192', '19689793'], S ['21490936']
PMC3072	1	FALSE	1	FALSE		1	D ['20038657'], S ['21490935']
PMC3072	3	FALSE	1	FALSE		3	D ['18849966', '18849965', '19000829'], S ['21490934']
PMC3072	0	TRUE	3	FALSE		0	S ['16527978', '21490933', '19694873']
PMC3072	2	FALSE	3	FALSE		2	D ['11356022', '19956716'], S ['17689523', '20177058', '21490931']
PMC3072	10	FALSE	1	FALSE		10	D ['18408712', '18408711', '16402857', '17167414', '18625400', '18499458', '20357107 '19943954', '20047983', '19660933'], S ['21490930']
PMC3072	2	FALSE	2	FALSE		2	D ['16195470', '10641664'], S ['21490928', '11008824']
PMC3072	6	FALSE	1	FALSE		6	D ['20064449', '15790681', '20049877', '20064450', '17360565', '19995950'], S ['21490
PMC3072	2	FALSE	1	FALSE		2	D ['16827013', '19294922'], S ['21490926']
PMC3072	3	FALSE	2	FALSE		3	D ['19540952', '17050041', '19453396'], S ['21490925', '19543385']
PMC3072	3	FALSE	1	FALSE		3	D ['14530446', '19050014', '9649623'], S ['21490924']
PMC3072	2	FALSE	1	FALSE		2	D ['16894146', '16301659'], S ['21490923']
PMC3072	0	TRUE	1	FALSE		0	S ['21490922']
PMC3072	2	FALSE	1	FALSE		2	D ['16367772', '12101039'], S ['21490921']
PMC3072	1	FALSE	1	FALSE		1	D ['20558037'], S ['21490920']
PMC3072	0	TRUE	2	FALSE		0	S ['16631614', '21490919']
PMC3072	1	FALSE	2	FALSE		1	D ['20531985'], S ['18208976', '21490918']
PMC3072	3	FALSE	4	FALSE		3	D ['18463244', '15009682', '19535604'], S ['15156148', '21490976', '18726914', '19846
PMC3072	3	FALSE	3	FALSE		3	D ['9861006', '10358158', '14764585'], S ['21490975', '19074136', '15238603']
PMC3072	0	TRUE	1	FALSE		0	S ['21490974']
PMC3072	0	TRUE	6	FALSE		0	S ['21490972', '14752840', '21143369', '15095302', '20162609', '11336472']
PMC3072	1	FALSE	1	FALSE		1	D ['19029190'], S ['21490971']
PMC3072	2	FALSE	1	FALSE		2	D ['17640524', '12451133'], S ['21490970']
PMC3072	2	FALSE	1	FALSE		2	D ['14871364', '11475052'], S ['21490969']
PMC3072	2	FALSE	1	FALSE		2	D ['20167929', '18723424'], S ['21490968']

282']
268']
838',
',
927']
715']

PMC3072	8	FALSE	4	FALSE		8	D ['17638703', '18387180', '19765815', '16195288', '19765816', '16827699', '17975412 '19675675'], S ['21490967', '20808923', '18628828', '20559558']
PMC3072	2	FALSE	1	FALSE		2	D ['8288645', '1782212'], S ['21490965']
PMC3072	3	FALSE	2	FALSE		3	D ['16116445', '11726774', '16487726'], S ['21490964', '18671736']
PMC3072	0	TRUE	2	FALSE		0	S ['21490961', '19106307']
PMC3072	3	FALSE	6	FALSE		3	D ['11553577', '19332804', '11101562'], S ['19897651', '21490962', '16309456', '16008 '17768241', '15339271']
PMC3072	0	TRUE	2	FALSE		0	S ['17409151', '21490960']
PMC3072	1	FALSE	5	FALSE		1	D ['7877171'], S ['17157854', '17583712', '21490955', '16527366', '17962986']
PMC3072	2	FALSE	6	FALSE		2	D ['19284284', '18843368'], S ['15051281', '21490952', '19383118', '18171245', '18377 '19635025']
PMC3072	2	FALSE	1	FALSE		2	D ['19696603', '18029063'], S ['21483641']
PMC3072	0	TRUE	1	FALSE		0	S ['21483640']
PMC3072	0	TRUE	1	FALSE		0	S ['21483639']
PMC3072	3	FALSE	1	FALSE		3	D ['19520795', '20530476', '18535017'], S ['21483638']
PMC3072	6	FALSE	1	FALSE		6	D ['17962861', '16444375', '18958550', '10715786', '18613384', '18507090'], S ['21483
PMC3072	1	FALSE	2	FALSE		1	D ['17091077'], S ['20061857', '21483636']
PMC3072	1	FALSE	1	FALSE		1	D ['10350287'], S ['21483635']
PMC3072	5	FALSE	1	FALSE		5	D ['17786515', '17905027', '17968803', '17185093', '18635172'], S ['21483634']
PMC3072	5	FALSE	1	FALSE		5	D ['20512988', '15368448', '17724809', '20034047', '19318971'], S ['21483633']
PMC3072	1	FALSE	2	FALSE		1	D ['19931544'], S ['17287895', '21483632']
PMC3072	0	TRUE	1	FALSE		0	S ['21483631']
PMC3072	2	FALSE	1	FALSE		2	D ['17466600', '19032991'], S ['21483630']
PMC3072	0	TRUE	1	FALSE		0	S ['21483629']
PMC3072	2	FALSE	1	FALSE		2	D ['20590905', '11923602'], S ['21483627']
PMC3072	1	FALSE	3	FALSE		1	D ['16378172'], S ['19557549', '20132083', '21483625']
PMC3072	2	FALSE	2	FALSE		2	D ['19913991', '19343435'], S ['19198959', '21483624']
PMC3071	3	FALSE	3	FALSE		3	D ['14757604', '9240805', '15659966'], S ['14974006', '21471175', '15064208']
PMC3071	2	FALSE	1	FALSE		2	D ['19455553', '17060337'], S ['21471172']
PMC3077	2	FALSE	1	FALSE		2	D ['12381428', '11710080'], S ['21499449']
PMC3071	0	TRUE	2	FALSE		0	S ['19602527', '21494644']
PMC3071	4	FALSE	1	FALSE		4	D ['11012890', '2200803', '14964452', '10652025'], S ['21494642']
PMC3071	3	FALSE	3	FALSE		3	D ['8023443', '8354429', '19757909'], S ['19003978', '21494641', '20573592']
PMC3071	0	TRUE	3	FALSE		0	S ['17712427', '21494640', '17478450']
PMC3071	5	FALSE	6	FALSE		5	D ['20687218', '18619854', '8220092', '11075874', '19861519'], S ['21494639', '158684 '7612803', '17388667', '8850273', '15225833']
PMC3071	0	TRUE	1	FALSE		0	S ['21494638']
PMC3071	5	FALSE	4	FALSE		5	D ['18182054', '18258746', '20659558', '20130188', '19625511'], S ['19741132', '21248 '16269541', '21494637']
PMC3071	1	FALSE	3	FALSE		1	D ['15855434'], S ['16110325', '21494695', '20479889']
PMC3071	2	FALSE	1	FALSE		2	D ['9861414', '16466034'], S ['21494694']
PMC3071	0	TRUE	1	FALSE		0	S ['21494690']

',
583',
652',
637']
50',
115',

PMC3071[illegible]	2	FALSE	1	FALSE		2	D ['2296494', '15685092'], S ['21494689']
PMC3071[illegible]	0	TRUE	1	FALSE		0	S ['21494688']
PMC3071[illegible]	4	FALSE	2	FALSE		4	D ['17182609', '15071506', '16601139', '20018847'], S ['15297438', '21494687']
PMC3071[illegible]	2	FALSE	1	FALSE		2	D ['9593773', '12475386'], S ['21494686']
PMC3071[illegible]	1	FALSE	3	FALSE		1	D ['10558986'], S ['20179896', '18030355', '21494685']
PMC3071[illegible]	0	TRUE	1	FALSE		0	S ['21494684']
PMC3071[illegible]	0	TRUE	1	FALSE		0	S ['21494683']
PMC3071[illegible]	2	FALSE	6	FALSE		2	D ['12683538', '11005299'], S ['19146596', '11005298', '21494682', '16674582', '11298[illegible] '19451640']
PMC3071[illegible]	4	FALSE	2	FALSE		4	D ['18043717', '16416399', '18773084', '16847467'], S ['21494681', '18304320']
PMC3071[illegible]	2	FALSE	2	FALSE		2	D ['17204159', '20181713'], S ['21494680', '17005661']
PMC3071[illegible]	4	FALSE	1	FALSE		4	D ['10328804', '19515244', '9155244', '12969481'], S ['21494678']
PMC3071[illegible]	3	FALSE	1	FALSE		3	D ['2700895', '7802700', '20442937'], S ['21494677']
PMC3071[illegible]	0	TRUE	1	FALSE		0	S ['21494676']
PMC3071[illegible]	7	FALSE	2	FALSE		7	D ['16850313', '16964521', '20018713', '16027975', '12657298', '18430767', '18254594 ['21494675', '19334756']
PMC3071[illegible]	3	FALSE	1	FALSE		3	D ['10558881', '19592617', '17047689'], S ['21494673']
PMC3071[illegible]	4	FALSE	1	FALSE		4	D ['9549491', '19667222', '18825432', '17938370'], S ['21494672']
PMC3071[illegible]	8	FALSE	3	FALSE		8	D ['15167938', '16118348', '12560917', '14576477', '16990345', '12640028', '18190713 '15452161'], S ['18845559', '21494671', '18953412']
PMC3071[illegible]	7	FALSE	1	FALSE		7	D ['20594299', '20716359', '19835577', '20194752', '21084477', '20185493', '18684813 ['21494670']
PMC3071[illegible]	5	FALSE	1	FALSE		5	D ['20537539', '17148320', '17698452', '18682357', '19452178'], S ['21494669']
PMC3071[illegible]	7	FALSE	1	FALSE		7	D ['18024482', '15613952', '15802397', '12533125', '16322894', '19226540', '11438414 ['21494667']
PMC3071[illegible]	2	FALSE	2	FALSE		2	D ['11352728', '19164292'], S ['21494666', '19874007']
PMC3071[illegible]	2	FALSE	5	FALSE		2	D ['19132981', '20351212'], S ['17581935', '17391377', '19558526', '21494333', '17949[illegible]
PMC3071[illegible]	4	FALSE	1	FALSE		4	D ['14683701', '14980566', '15325353', '15929647'], S ['21494331']
PMC3071[illegible]	1	FALSE	1	FALSE		1	D ['15479708'], S ['21494330']
PMC3071[illegible]	3	FALSE	1	FALSE		3	D ['17051813', '12685072', '12739854'], S ['21494329']
PMC3071[illegible]	2	FALSE	1	FALSE		2	D ['15784743', '18554145'], S ['21494328']
PMC3071[illegible]	5	FALSE	1	FALSE		5	D ['18995938', '17051813', '18063010', '12739854', '12685072'], S ['21494326']
PMC3062[illegible]	3	FALSE	2	FALSE		3	D ['17020799', '17567794', '19070450'], S ['21460019', '18039797']
PMC3062[illegible]	4	FALSE	1	FALSE		4	D ['12766836', '12954562', '8589132', '11357153'], S ['21460018']
PMC3062[illegible]	3	FALSE	1	FALSE		3	D ['9182474', '8291822', '12390596'], S ['21460003']
PMC3078[illegible]	0	TRUE	2	FALSE		0	S ['18653731', '21466698']
PMC3078[illegible]	0	TRUE	1	FALSE		0	S ['21466699']
PMC3078[illegible]	4	FALSE	1	FALSE		4	D ['20081562', '20417153', '11722799', '1831751'], S ['21466665']
PMC3071[illegible]	0	TRUE	1	FALSE		0	S ['21467101']
PMC3071[illegible]	4	FALSE	1	FALSE		4	D ['19152681', '16227797', '15817527', '15161896'], S ['21467104']
PMC3071[illegible]	0	TRUE	3	FALSE		0	S ['18479744', '21422131', '16875901']
PMC3071[illegible]	1	FALSE	1	FALSE		1	D ['19366494'], S ['21422129']

977',
'], S
',
'], S
'], S
441']

PMC3071	1	FALSE	1	FALSE		1	D ['11869618'], S ['21422138']
PMC3071	0	TRUE	1	FALSE		0	S ['21402681']
PMC3071	4	FALSE	2	FALSE		4	D ['10511607', '12500665', '16478900', '16926275'], S ['18469240', '21398246']
PMC3071	1	FALSE	2	FALSE		1	D ['16106005'], S ['19157653', '21398248']
PMC3071	4	FALSE	1	FALSE		4	D ['17984482', '18381180', '18707986', '17923591'], S ['21422125']
PMC3073	1	FALSE	1	FALSE		1	D ['15531427'], S ['21489219']
PMC3073	0	TRUE	1	FALSE		0	S ['21489218']
PMC3073	1	FALSE	1	FALSE		1	D ['18656277'], S ['21489217']
PMC3073	0	TRUE	1	FALSE		0	S ['21489214']
PMC3073	1	FALSE	1	FALSE		1	D ['17980649'], S ['21489213']
PMC3073	0	TRUE	1	FALSE		0	S ['21489212']
PMC3073	0	TRUE	1	FALSE		0	S ['21489211']
PMC3073	1	FALSE	1	FALSE		1	D ['17119190'], S ['21489210']
PMC3073	0	TRUE	1	FALSE		0	S ['21489209']
PMC3073	0	TRUE	1	FALSE		0	S ['21489208']
PMC3073	0	TRUE	3	FALSE		0	S ['21489207', '17854706', '16909291']
PMC3074	2	FALSE	4	FALSE		2	D ['12657662', '12815026'], S ['9623887', '18248614', '7472524', '8815906']
PMC3074	1	FALSE	4	FALSE		1	D ['15063186'], S ['17912743', '18331887', '19790262', '21344401']
PMC3074	2	FALSE	2	FALSE		2	D ['17937229', '9183698'], S ['19459217', '20603194']
PMC3069	0	TRUE	7	FALSE		0	S ['9864188', '21467263', '17517651', '17616592', '16820471', '17277211', '18689513']
PMC3071	2	FALSE	2	FALSE		2	D ['16278657', '17967812'], S ['21483721', '7519623']
PMC3071	2	FALSE	1	FALSE		2	D ['9560155', '15860578'], S ['21483719']
PMC3071	0	TRUE	2	FALSE		0	S ['18728776', '21483714']
PMC3071	1	FALSE	2	FALSE		1	D ['21031138'], S ['20377886', '21483718']
PMC3071	0	TRUE	1	FALSE		0	S ['21483716']
PMC3071	0	TRUE	1	FALSE		0	S ['21483715']
PMC3071	0	TRUE	5	FALSE		0	S ['19627591', '19627594', '19627588', '15194254', '21483712']
PMC3071	1	FALSE	4	FALSE		1	D ['9851613'], S ['11295173', '9553063', '20338198', '21483720']
PMC3071	0	TRUE	2	FALSE		0	S ['18644955', '21483717']
PMC3071	2	FALSE	2	FALSE		2	D ['15019339', '16973662'], S ['16170117', '21483713']
PMC3071	0	TRUE	9	FALSE		0	S ['18296439', '19443417', '18573308', '18160404', '13678633', '18848453', '19383516 '14754899', '21483711']
PMC3071	0	TRUE	5	FALSE		0	S ['17870072', '19423396', '17134654', '20532163', '21483710']
PMC3071	0	TRUE	4	FALSE		0	S ['18981509', '16022790', '9817521', '21483709']
PMC3071	3	FALSE	2	FALSE		3	D ['9601102', '10884615', '15504914'], S ['18419774', '21483708']
PMC3071	2	FALSE	2	FALSE		2	D ['19034521', '20479152'], S ['16787988', '21490708']
PMC3071	2	FALSE	2	FALSE		2	D ['15638826', '11821707'], S ['20739939', '21483652']
PMC3071	3	FALSE	1	FALSE		3	D ['20606252', '17545546', '18521084'], S ['21483692']
PMC3071	1	FALSE	2	FALSE		1	D ['10206504'], S ['21483690', '18641849']
PMC3071	1	FALSE	2	FALSE		1	D ['19208751'], S ['18832708', '21483689']
PMC3071	6	FALSE	4	FALSE		6	D ['11753363', '12069726', '19888332', '16049021', '16510898', '19015153'], S ['21483 '18814329', '16939645', '16275786']

',
688',

PMC3071	0	TRUE	2	FALSE		0	S ['21483687', '12134017']
PMC3071	1	FALSE	4	FALSE		1	D ['11282899'], S ['8635268', '21483686', '11181072', '12769644']
PMC3071	2	FALSE	1	FALSE		2	D ['11179319', '10586030'], S ['21483684']
PMC3071	3	FALSE	2	FALSE		3	D ['18495166', '10827961', '12779708'], S ['21483682', '19270753']
PMC3071	0	TRUE	1	FALSE		0	S ['21483681']
PMC3071	2	FALSE	2	FALSE		2	D ['19289061', '16024039'], S ['21483680', '19413984']
PMC3071	1	FALSE	2	FALSE		1	D ['18775941'], S ['21483679', '16052453']
PMC3071	0	TRUE	1	FALSE		0	S ['21483678']
PMC3071	1	FALSE	2	FALSE		1	D ['20195468'], S ['21483677', '19887665']
PMC3071	2	FALSE	1	FALSE		2	D ['19952710', '12370434'], S ['21483676']
PMC3071	4	FALSE	1	FALSE		4	D ['17108166', '16672644', '17314293', '16630540'], S ['21483675']
PMC3071	0	TRUE	1	FALSE		0	S ['21483674']
PMC3071	3	FALSE	4	FALSE		3	D ['11463864', '11389194', '12830383'], S ['14686897', '21483673', '17250763', '14686
PMC3071	0	TRUE	2	FALSE		0	S ['19299735', '21483672']
PMC3071	1	FALSE	1	FALSE		1	D ['19539325'], S ['21483670']
PMC3071	1	FALSE	1	FALSE		1	D ['16887991'], S ['21483669']
PMC3071	2	FALSE	1	FALSE		2	D ['12941799', '17096326'], S ['21483668']
PMC3071	0	TRUE	1	FALSE		0	S ['21483666']
PMC3071	1	FALSE	2	FALSE		1	D ['20562860'], S ['21483665', '17410175']
PMC3071	5	FALSE	1	FALSE		5	D ['15753212', '17174297', '16414040', '9585507', '12070087'], S ['21483663']
PMC3073	1	FALSE	1	FALSE		1	D ['16841214'], S ['21463506']
PMC3078	0	TRUE	1	FALSE		0	S ['21463527']
PMC3076	0	TRUE	5	FALSE		0	S ['15491494', '19435491', '18405345', '21463520', '15960852']
PMC3078	0	TRUE	1	FALSE		0	S ['21463503']
PMC3078	1	FALSE	1	FALSE		1	D ['16329458'], S ['21463499']
PMC3070	0	TRUE	1	FALSE		0	S ['21464103']
PMC3076	0	TRUE	2	FALSE		0	S ['16256688', '21463509']
PMC3073	3	FALSE	2	FALSE		3	D ['18236013', '20852629', '19441077'], S ['17551265', '15953824']
PMC3074	6	FALSE	3	FALSE		6	D ['16148235', '16738217', '18184790', '17715194', '19692618', '8865200'], S ['162514<br>'17499115', '15858048']
PMC3071	0	TRUE	2	FALSE		0	S ['21475643', '17065850']
PMC3071	0	TRUE	2	FALSE		0	S ['21475642', '19907386']
PMC3070	4	FALSE	3	FALSE		4	D ['20347483', '16912954', '16905784', '18427206'], S ['14565604', '21483703', '19617
PMC3070	2	FALSE	2	FALSE		2	D ['12960521', '17638204'], S ['21483701', '18941376']
PMC3070	1	FALSE	5	FALSE		1	D ['10456100'], S ['16088143', '21483699', '20381625', '15146191', '17924521']
PMC3070	1	FALSE	2	FALSE		1	D ['11967025'], S ['17005917', '21483698']
PMC3070	0	TRUE	1	FALSE		0	S ['21483696']
PMC3070	3	FALSE	3	FALSE		3	D ['17919499', '19360310', '9160173'], S ['21314952', '20830292', '21483694']
PMC3070	2	FALSE	3	FALSE		2	D ['18497855', '2485170'], S ['21483693', '17502004', '3068854']
PMC3070	2	FALSE	2	FALSE		2	D ['16127548', '17306256'], S ['21483751', '20457672']
PMC3070	0	TRUE	3	FALSE		0	S ['21483750', '15592573', '19342373']
PMC3070	0	TRUE	1	FALSE		0	S ['21483749']

896']
52',
847']

PMC3070	5	FALSE	1	FALSE		5	D ['16461792', '18946062', '18484169', '18275280', '12393425'], S ['21483748']
PMC3070	0	TRUE	1	FALSE		0	S ['21483747']
PMC3070	3	FALSE	1	FALSE		3	D ['18593687', '16562712', '19387262'], S ['21483746']
PMC3070	3	FALSE	3	FALSE		3	D ['18433818', '4396320', '19852440'], S ['19267653', '21483745', '19505494']
PMC3070	2	FALSE	1	FALSE		2	D ['18974850', '17156863'], S ['21483743']
PMC3070	3	FALSE	1	FALSE		3	D ['17251437', '8880363', '10976075'], S ['21483742']
PMC3070	4	FALSE	2	FALSE		4	D ['17356813', '16315345', '18481517', '20380198'], S ['18388286', '21483739']
PMC3070	0	TRUE	1	FALSE		0	S ['21483738']
PMC3070	0	TRUE	2	FALSE		0	S ['19412814', '21483737']
PMC3070	5	FALSE	2	FALSE		5	D ['17572678', '16421948', '18342006', '15307186', '17355967'], S ['20586411', '21483
PMC3070	0	TRUE	2	FALSE		0	S ['19292914', '21483735']
PMC3070	3	FALSE	2	FALSE		3	D ['19148282', '20220755', '10747069'], S ['18493072', '21483733']
PMC3070	0	TRUE	1	FALSE		0	S ['21483732']
PMC3070	0	TRUE	0	TRUE		1	related
PMC3076	0	TRUE	1	FALSE		0	S ['21457580']
PMC3076	1	FALSE	1	FALSE		1	D ['9583627'], S ['21457578']
PMC3076	0	TRUE	1	FALSE		0	S ['21457573']
PMC3076	1	FALSE	2	FALSE		1	D ['8890877'], S ['21457572', '16239364']
PMC3029	1	FALSE	2	FALSE		1	D ['17295071'], S ['20043200', '19526346']
PMC3064	0	TRUE	1	FALSE		0	S ['21271397']
PMC3062	1	FALSE	3	FALSE		1	D ['16461857'], S ['16461859', '18239198', '20808140']
PMC3071	1	FALSE	1	FALSE		1	D ['12236358'], S ['21396180']
PMC3075	0	TRUE	4	FALSE		0	S ['17868874', '17502506', '21482873', '19901214']
PMC3075	0	TRUE	1	FALSE		0	S ['21464381']
PMC3074	0	TRUE	1	FALSE		0	S ['21464384']
PMC3064	0	TRUE	3	FALSE		0	S ['21264696', '19412324', '16262768']
PMC3074	0	TRUE	2	FALSE		0	S ['18355457', '21428953']
PMC3062	3	FALSE	1	FALSE		3	D ['12644457', '12058033', '7542741'], S ['21223946']
PMC3060	1	FALSE	2	FALSE		1	D ['12444595'], S ['20566712', '21256213']
PMC3078	2	FALSE	3	FALSE		2	D ['18077351', '19804402'], S ['17065595', '18521886', '21457536']
PMC3078	6	FALSE	1	FALSE		6	D ['19539330', '19196738', '18046447', '17908970', '20082279', '16960146'], S ['21457
PMC3069	0	TRUE	2	FALSE		0	S ['21459975', '17978324']
PMC3066	0	TRUE	1	FALSE		0	S ['21450778']
PMC3066	0	TRUE	1	FALSE		0	S ['21450775']
PMC3066	0	TRUE	1	FALSE		0	S ['21450772']
PMC3066	0	TRUE	1	FALSE		0	S ['21450770']
PMC3066	0	TRUE	1	FALSE		0	S ['21450771']
PMC3066	0	TRUE	1	FALSE		0	S ['21450773']
PMC3066	0	TRUE	1	FALSE		0	S ['21450762']
PMC3066	0	TRUE	1	FALSE		0	S ['21450765']
PMC3066	0	TRUE	1	FALSE		0	S ['21450777']
PMC3066	1	FALSE	1	FALSE		1	D ['16326792'], S ['21450767']

736']
555']

PMC3066	0	TRUE	1	FALSE		0	S ['21450768']
PMC3066	0	TRUE	1	FALSE		0	S ['21450763']
PMC3066	0	TRUE	1	FALSE		0	S ['21450781']
PMC3066	0	TRUE	1	FALSE		0	S ['21450764']
PMC3064	2	FALSE	2	FALSE		2	D ['18684092', '11127194'], S ['15869917', '21130909']
PMC3078	3	FALSE	0	TRUE		4	D ['17846358', '18439564', '3337705']
PMC3072	3	FALSE	1	FALSE		3	D ['20865114', '2772556', '9377907'], S ['21487452']
PMC3062	1	FALSE	2	FALSE		1	D ['16032561'], S ['21427397', '19375159']
PMC3077	0	TRUE	2	FALSE		0	S ['21491347', '19953428']
PMC3076	1	FALSE	1	FALSE		1	D ['17133048'], S ['21499568']
PMC3072	0	TRUE	2	FALSE		0	S ['21432689', '18453390']
PMC3068	1	FALSE	1	FALSE		1	D ['20085595'], S ['21104347']
PMC3073	0	TRUE	3	FALSE		0	S ['21462011', '16828126', '17292628']
PMC3078	1	FALSE	1	FALSE		1	D ['19157967'], S ['21453552']
PMC3075	1	FALSE	1	FALSE		1	D ['15646591'], S ['21494394']
PMC3075	4	FALSE	1	FALSE		4	D ['12008807', '8730271', '12214948', '12806324'], S ['21494382']
PMC3075	0	TRUE	1	FALSE		0	S ['21494387']
PMC3075	0	TRUE	2	FALSE		0	S ['19089131', '21494379']
PMC3076	0	TRUE	1	FALSE		0	S ['21383624']
PMC3058	3	FALSE	2	FALSE		3	D ['12101186', '17761949', '11038323'], S ['18984668', '21163858']
PMC3058	2	FALSE	2	FALSE		2	D ['18482975', '15371448'], S ['15946935', '21163859']
PMC3049	2	FALSE	2	FALSE		2	D ['16581043', '20175987'], S ['20921387', '21236334']
PMC3071	0	TRUE	1	FALSE		0	S ['19074189']
PMC3072	0	TRUE	2	FALSE		0	S ['20001687', '21441982']
PMC3072	6	FALSE	2	FALSE		6	D ['10684894', '8783227', '7996176', '10664098', '12813147', '17717695'], S ['1922557 '20685378']
PMC3069	0	TRUE	7	FALSE		0	S ['15322489', '9926235', '20971819', '16224207', '19390308', '21454882', '2069139']
PMC3069	1	FALSE	2	FALSE		1	D ['20562328'], S ['18695255', '21454879']
PMC3069	0	TRUE	2	FALSE		0	S ['21131035', '21454878']
PMC3069	0	TRUE	1	FALSE		0	S ['21454877']
PMC3058	2	FALSE	2	FALSE		2	D ['16499767', '18585930'], S ['21391248', '18557780']
PMC3056	1	FALSE	2	FALSE		1	D ['17628434'], S ['21391254', '17329470']
PMC3077	1	FALSE	1	FALSE		1	D ['18808340'], S ['21460483']
PMC3076	1	FALSE	1	FALSE		1	D ['16514623'], S ['21494428']
PMC3075	4	FALSE	3	FALSE		4	D ['19815430', '12835208', '18710985', '18180339'], S ['18092513', '21343825', '18335
PMC3071	0	TRUE	3	FALSE		0	S ['11604438', '11440328', '21462026']
PMC3059	0	TRUE	1	FALSE		0	S ['21455479']
PMC3059	3	FALSE	1	FALSE		3	D ['17489233', '7692896', '16556310'], S ['21455480']
PMC3059	0	TRUE	2	FALSE		0	S ['21455478', '17513294']
PMC3059	0	TRUE	1	FALSE		0	S ['21455475']
PMC3059	2	FALSE	1	FALSE		2	D ['20570211', '19781984'], S ['21455476']
PMC3059	2	FALSE	1	FALSE		2	D ['15665377', '18318008'], S ['21455477']

5',
479']

PMC3049	2	FALSE	6	FALSE		2	D ['18971464', '16226840'], S ['18380666', '18805468', '16977615', '21280048', '20082 '18977221']
PMC2896	1	FALSE	5	FALSE		1	D ['17278499'], S ['20204448', '19201355', '18072498', '17659237', '19147902']
PMC3071	0	TRUE	4	FALSE		0	S ['21355664', '20000928', '18344871', '15058346']
PMC3076	5	FALSE	2	FALSE		5	D ['12815052', '12663439', '11756186', '12468433', '8946930'], S ['14982881', '214535
PMC3053	0	TRUE	1	FALSE		0	S ['21346625']
PMC2905	1	FALSE	1	FALSE		1	D ['10934175'], S ['20131000']
PMC2889	0	TRUE	1	FALSE		0	S ['19621260']
PMC3069	2	FALSE	3	FALSE		2	D ['19789228', '19137309'], S ['19703886', '17449772', '21448939']
PMC3072	0	TRUE	1	FALSE		0	S ['21479113']
PMC3066	2	FALSE	2	FALSE		2	D ['15858627', '8607606'], S ['21464855', '7599584']
PMC3066	0	TRUE	1	FALSE		0	S ['21464848']
PMC3066	0	TRUE	1	FALSE		0	S ['21464851']
PMC3066	0	TRUE	1	FALSE		0	S ['21464850']
PMC3066	0	TRUE	1	FALSE		0	S ['21464856']
PMC3066	0	TRUE	1	FALSE		0	S ['21464847']
PMC3043	0	TRUE	1	FALSE		0	S ['19888926']
PMC2919	0	TRUE	3	FALSE		0	S ['20331502', '19388096', '18479942']
PMC3071	0	TRUE	1	FALSE		0	S ['21170589']
PMC3072	2	FALSE	1	FALSE		2	D ['14605224', '10318685'], S ['21462389']
PMC3072	2	FALSE	2	FALSE		2	D ['20047795', '19783279'], S ['20439132', '21462388']
PMC3072	4	FALSE	1	FALSE		4	D ['8455762', '15269466', '11360374', '2362678'], S ['21462385']
PMC3072	1	FALSE	1	FALSE		1	D ['15623763'], S ['21462384']
PMC3072	4	FALSE	1	FALSE		4	D ['18506009', '14752429', '20106358', '12830056'], S ['21462382']
PMC3072	1	FALSE	1	FALSE		1	D ['19439972'], S ['21462381']
PMC3072	0	TRUE	1	FALSE		0	S ['21462380']
PMC3036	0	TRUE	1	FALSE		0	S ['20521133']
PMC3074	4	FALSE	4	FALSE		4	D ['10762551', '18608113', '11753822', '17607593'], S ['20689816', '21468018', '10570 '20231434']
PMC3071	1	FALSE	4	FALSE		1	D ['17561354'], S ['18780893', '21472143', '20453000', '20385987']
PMC3071	3	FALSE	4	FALSE		3	D ['20165849', '17283153', '18931344'], S ['19628770', '18813353', '19010845', '21472
PMC3071	0	TRUE	3	FALSE		0	S ['17597103', '21472137', '18516298']
PMC3071	1	FALSE	3	FALSE		1	D ['17873866'], S ['19637935', '16914580', '21472135']
PMC3071	1	FALSE	3	FALSE		1	D ['9168822'], S ['18570887', '21472134', '12414638']
PMC3070	4	FALSE	1	FALSE		4	D ['19261965', '19381789', '18816391', '12386115'], S ['21464862']
PMC3070	0	TRUE	1	FALSE		0	S ['21464859']
PMC3069	2	FALSE	1	FALSE		2	D ['18321973', '15452216'], S ['21483780']
PMC3069	1	FALSE	2	FALSE		1	D ['17872499'], S ['19786631', '21483779']
PMC3069	2	FALSE	2	FALSE		2	D ['19079604', '18632867'], S ['18842738', '21483777']
PMC3069	3	FALSE	1	FALSE		3	D ['19197294', '18674538', '18272812'], S ['21483776']
PMC3069	3	FALSE	6	FALSE		3	D ['18660541', '18419568', '17017055'], S ['16521028', '19780814', '16599933', '18177 '21483775', '17236413']

980',
45']
183',
138']
673',

PMC3069	0	TRUE	1	FALSE		0	S ['21483774']
PMC3069	1	FALSE	1	FALSE		1	D ['19201924'], S ['21483773']
PMC3069	3	FALSE	2	FALSE		3	D ['12152752', '12152754', '11833798'], S ['19244487', '21483772']
PMC3069	4	FALSE	1	FALSE		4	D ['18487485', '20610667', '17030881', '16145200'], S ['21483771']
PMC3069	1	FALSE	1	FALSE		1	D ['17148580'], S ['21483770']
PMC3069	0	TRUE	1	FALSE		0	S ['21483768']
PMC3069	3	FALSE	1	FALSE		3	D ['20362598', '15473975', '16249098'], S ['21483767']
PMC3069	1	FALSE	3	FALSE		1	D ['19428797'], S ['18523805', '9932958', '21483766']
PMC3069	2	FALSE	5	FALSE		2	D ['9108044', '11864991'], S ['16214870', '19002657', '19751659', '21483765', '196587
PMC3069	0	TRUE	1	FALSE		0	S ['21483764']
PMC3069	0	TRUE	5	FALSE		0	S ['20885892', '19724685', '17304503', '21461174', '17332344']
PMC3069	5	FALSE	1	FALSE		5	D ['11326271', '18754879', '19047140', '15373779', '17579625'], S ['21461173']
PMC3069	0	TRUE	2	FALSE		0	S ['14695158', '21461171']
PMC3069	1	FALSE	1	FALSE		1	D ['20133981'], S ['21461170']
PMC3069	3	FALSE	1	FALSE		3	D ['19501013', '18483355', '19680292'], S ['21461169']
PMC3068	3	FALSE	1	FALSE		3	D ['16260745', '21418605', '19422698'], S ['21453557']
PMC3073	1	FALSE	3	FALSE		1	D ['17563079'], S ['20303322', '17068267', '21453486']
PMC3078	3	FALSE	1	FALSE		3	D ['11454499', '18402681', '16224305'], S ['21453534']
PMC3078	0	TRUE	2	FALSE		0	S ['21308978', '21453505']
PMC3078	1	FALSE	3	FALSE		1	D ['11959894'], S ['20300640', '18271962', '21453500']
PMC3076	2	FALSE	1	FALSE		2	D ['16996320', '19678944'], S ['21453543']
PMC3078	2	FALSE	1	FALSE		2	D ['1384037', '17766423'], S ['21453489']
PMC3076	0	TRUE	1	FALSE		0	S ['21453488']
PMC3078	9	FALSE	1	FALSE		9	D ['18806915', '10673775', '14966478', '15118354', '18628678', '10893498', '18546120 '11324940', '15108187'], S ['21453464']
PMC3078	0	TRUE	1	FALSE		0	S ['21453478']
PMC3078	0	TRUE	2	FALSE		0	S ['19703317', '21453540']
PMC3078	2	FALSE	1	FALSE		2	D ['20137283', '18775527'], S ['21453468']
PMC3078	4	FALSE	1	FALSE		4	D ['18203906', '17079542', '16452367', '18029494'], S ['21453491']
PMC3076	0	TRUE	2	FALSE		0	S ['18655723', '21453509']
PMC3078	1	FALSE	1	FALSE		1	D ['17535433'], S ['21453498']
PMC3076	0	TRUE	1	FALSE		0	S ['21453518']
PMC3069	1	FALSE	2	FALSE		1	D ['19297565'], S ['15548438', '21454449']
PMC3068	4	FALSE	1	FALSE		4	D ['10232312', '16652060', '15555666', '16631881'], S ['21454456']
PMC3077	1	FALSE	2	FALSE		1	D ['10490955'], S ['16314481', '21453480']
PMC3071	1	FALSE	1	FALSE		1	D ['15617052'], S ['15579550']
PMC3071	1	FALSE	3	FALSE		1	D ['18088284'], S ['16180627', '9427330', '17507572']
PMC3071	0	TRUE	0	TRUE		1	related
PMC3071	3	FALSE	0	TRUE		4	D ['16139182', '18562506', '17086910']
PMC3071	1	FALSE	1	FALSE		1	D ['18072813'], S ['19154620']
PMC3071	0	TRUE	2	FALSE		0	S ['18197970', '18536710']
PMC3078	0	TRUE	1	FALSE		0	S ['21453516']

34']
',

PMC3076[illegible]	4	FALSE	1	FALSE		4	D ['16026154', '2513246', '2592352', '3094925'], S ['21494434']
PMC3070[illegible]	0	TRUE	1	FALSE		0	S ['21468299']
PMC3076[illegible]	2	FALSE	0	TRUE		3	D ['9241955', '15189314']
PMC3076[illegible]	0	TRUE	0	TRUE		1	related
PMC3077[illegible]	0	TRUE	1	FALSE		0	S ['21453502']
PMC3077[illegible]	1	FALSE	1	FALSE		1	D ['10589442'], S ['21453474']
PMC3077[illegible]	0	TRUE	1	FALSE		0	S ['21453520']
PMC3074[illegible]	0	TRUE	2	FALSE		0	S ['19046466', '21450112']
PMC3076[illegible]	0	TRUE	1	FALSE		0	S ['21453513']
PMC3078[illegible]	0	TRUE	4	FALSE		0	S ['19917088', '18549498', '21453521', '17897610']
PMC3076[illegible]	2	FALSE	1	FALSE		2	D ['18234078', '8702037'], S ['21453493']
PMC3076[illegible]	1	FALSE	1	FALSE		1	D ['19835832'], S ['21453465']
PMC3068[illegible]	2	FALSE	2	FALSE		2	D ['17588527', '12840017'], S ['21483483', '20041200']
PMC3068[illegible]	3	FALSE	2	FALSE		3	D ['7579351', '18681784', '16955993'], S ['19629158', '21483481']
PMC3068[illegible]	4	FALSE	4	FALSE		4	D ['20057383', '20540110', '19116616', '18793442'], S ['19888215', '21483480', '18974[illegible] '18192387']
PMC3068[illegible]	0	TRUE	1	FALSE		0	S ['21483479']
PMC3068[illegible]	0	TRUE	1	FALSE		0	S ['21483478']
PMC3068[illegible]	1	FALSE	2	FALSE		1	D ['17728389'], S ['14724638', '21483475']
PMC3069	3	FALSE	2	FALSE		3	D ['15882589', '14555699', '17237362'], S ['21483759', '17573799']
PMC3069	6	FALSE	2	FALSE		6	D ['18502872', '16081414', '15994307', '16477007', '18227161', '16923812'], S ['21483[illegible] '19888998']
PMC3069	0	TRUE	4	FALSE		0	S ['16432214', '21483755', '18714371', '20080630']
PMC3069	8	FALSE	1	FALSE		8	D ['19379692', '15180994', '17898715', '19508735', '16728974', '17189264', '17166833[illegible] '12060701'], S ['21483810']
PMC3069	3	FALSE	4	FALSE		3	D ['18582455', '14660434', '12913077'], S ['21483806', '15630097', '20661450', '19101[illegible]
PMC3069	1	FALSE	1	FALSE		1	D ['15824739'], S ['21483804']
PMC3069	1	FALSE	4	FALSE		1	D ['15340061'], S ['19584093', '21483803', '18695251', '19622630']
PMC3069[illegible]	5	FALSE	3	FALSE		5	D ['16617099', '17519251', '17877705', '20233950', '21098730'], S ['21483796', '15923[illegible] '15649364']
PMC3070[illegible]	3	FALSE	1	FALSE		3	D ['9446686', '10801492', '18796334'], S ['21494323']
PMC3069	5	FALSE	3	FALSE		5	D ['16293257', '11006398', '17631297', '19875120', '11006387'], S ['21483808', '20655[illegible] '9596544']
PMC3069	6	FALSE	1	FALSE		6	D ['14707081', '9710218', '11994471', '11398966', '19605564', '10803845'], S ['214838[illegible]
PMC3069	2	FALSE	2	FALSE		2	D ['15669099', '1883934'], S ['21483802', '19435897']
PMC3069	2	FALSE	3	FALSE		2	D ['10342804', '20551234'], S ['17098771', '21483799', '20975481']
PMC3069	4	FALSE	1	FALSE		4	D ['20306336', '11238038', '15031206', '16485041'], S ['21483798']
PMC3069[illegible]	4	FALSE	6	FALSE		4	D ['19420254', '19515925', '21106844', '15737744'], S ['21483795', '18060047', '14557[illegible] '16723543', '18953410', '16510140']
PMC3069[illegible]	0	TRUE	3	FALSE		0	S ['21483793', '20950866', '20974178']
PMC3069[illegible]	2	FALSE	1	FALSE		2	D ['19195863', '18184721'], S ['21483792']
PMC3069[illegible]	3	FALSE	3	FALSE		3	D ['14742438', '19833968', '18728219'], S ['19179447', '21483791', '17525164']
PMC3069[illegible]	0	TRUE	1	FALSE		0	S ['21483790']

823',
756',
',
574']
346',
049',
05']
550',

PMC3069	4	FALSE	3	FALSE			4	D ['14592837', '15772070', '15128841', '11390463'], S ['12847216', '21483789', '17391
PMC3069	1	FALSE	1	FALSE			1	D ['18985733'], S ['21483788']
PMC3069	4	FALSE	1	FALSE			4	D ['15238511', '16547114', '20157005', '19607838'], S ['21483787']
PMC3069	5	FALSE	3	FALSE			5	D ['17713478', '19114593', '18716661', '19451218', '19451217'], S ['17928865', '19415 '21483786']
PMC3069	10	FALSE	1	FALSE			10	D ['12418106', '8429558', '15657931', '16008354', '7756993', '15700296', '17073732', '16373476', '10091661', '11724549'], S ['21483785']
PMC3069	0	TRUE	2	FALSE			0	S ['19555716', '21483784']
PMC3069	1	FALSE	5	FALSE			1	D ['17457855'], S ['16051695', '12813409', '17389524', '21483783', '15223820']
PMC3069	1	FALSE	2	FALSE			1	D ['19197370'], S ['21483842', '18353938']
PMC3069	2	FALSE	2	FALSE			2	D ['17499321', '18003806'], S ['21483841', '17933898']
PMC3069	0	TRUE	3	FALSE			0	S ['15313920', '19351852', '21483840']
PMC3069	4	FALSE	1	FALSE			4	D ['19929911', '12067237', '12716437', '19008301'], S ['21483838']
PMC3069	3	FALSE	2	FALSE			3	D ['15318244', '17875220', '17060371'], S ['21483837', '15753313']
PMC3069	1	FALSE	2	FALSE			1	D ['15965720'], S ['21483836', '18784755']
PMC3069	0	TRUE	1	FALSE			0	S ['21483835']
PMC3069	3	FALSE	1	FALSE			3	D ['16428731', '19635914', '16237097'], S ['21483834']
PMC3069	1	FALSE	1	FALSE			1	D ['20237237'], S ['21483833']
PMC3069	3	FALSE	3	FALSE			3	D ['11782443', '18829553', '20308427'], S ['21483830', '20562908', '15150106']
PMC3069	4	FALSE	1	FALSE			4	D ['15072950', '11741928', '15572053', '16432067'], S ['21483829']
PMC3069	2	FALSE	7	FALSE			2	D ['19297619', '18174328'], S ['15853923', '15716067', '19929378', '21483827', '15772 '18793404', '16298405']
PMC3069	2	FALSE	6	FALSE			2	D ['12843287', '16272153'], S ['10627585', '21483826', '19892736', '17581956', '20505 '19906877']
PMC3069	3	FALSE	2	FALSE			3	D ['11161881', '10204967', '12445835'], S ['21483825', '15368600']
PMC3069	9	FALSE	1	FALSE			9	D ['19847796', '21073609', '19567675', '18634034', '20640596', '19293314', '18521189 '20188135', '20229273'], S ['21483822']
PMC3069	2	FALSE	1	FALSE			2	D ['11900494', '18171218'], S ['21483819']
PMC3069	2	FALSE	1	FALSE			2	D ['19962344', '19255111'], S ['21483816']
PMC3069	3	FALSE	2	FALSE			3	D ['19436712', '10196297', '11483779'], S ['21483815', '20098712']
PMC3069	1	FALSE	3	FALSE			1	D ['16407275'], S ['20121115', '17389385', '21483814']
PMC3069	1	FALSE	1	FALSE			1	D ['16086548'], S ['21483813']
PMC3069	0	TRUE	1	FALSE			0	S ['21483872']
PMC3069	5	FALSE	2	FALSE			5	D ['15613352', '20525690', '17267498', '20231447', '19640992'], S ['18762167', '21483
PMC3069	1	FALSE	1	FALSE			1	D ['17478621'], S ['21483870']
PMC3069	1	FALSE	6	FALSE			1	D ['14552658'], S ['10373585', '11734009', '16044462', '21483869', '20639539', '14982
PMC3069	2	FALSE	3	FALSE			2	D ['18485817', '15176975'], S ['21483868', '20008106', '12656194']
PMC3069	3	FALSE	1	FALSE			3	D ['17289102', '2052593', '9188607'], S ['21483867']
PMC3069	0	TRUE	1	FALSE			0	S ['21483866']
PMC3069	1	FALSE	1	FALSE			1	D ['21151554'], S ['21483865']
PMC3069	1	FALSE	3	FALSE			1	D ['10510507'], S ['17723051', '20682754', '21483863']
PMC3069	3	FALSE	4	FALSE			3	D ['11877283', '12594280', '12874260'], S ['19923446', '15599405', '14607913', '21483

124']
678',
314',
084',
',
871']
955']
862']

PMC3069	2	FALSE	2	FALSE		2	D ['19271242', '16450177'], S ['18850082', '21483861']
PMC3069	5	FALSE	2	FALSE		5	D ['18086664', '19501188', '16170208', '18547394', '18662741'], S ['21483860', '14563
PMC3069	0	TRUE	1	FALSE		0	S ['21483859']
PMC3069	3	FALSE	1	FALSE		3	D ['16854439', '16214404', '20051235'], S ['21483856']
PMC3069	0	TRUE	1	FALSE		0	S ['21483855']
PMC3069	1	FALSE	1	FALSE		1	D ['20371449'], S ['21483853']
PMC3069	2	FALSE	3	FALSE		2	D ['11158751', '8294241'], S ['17383964', '15726184', '21483852']
PMC3069	1	FALSE	3	FALSE		1	D ['18493601'], S ['20618960', '21483851', '11353636']
PMC3069	0	TRUE	2	FALSE		0	S ['18946507', '21483848']
PMC3069	1	FALSE	1	FALSE		1	D ['18167339'], S ['21483847']
PMC3069	0	TRUE	1	FALSE		0	S ['21483846']
PMC3069	1	FALSE	3	FALSE		1	D ['19084217'], S ['19673882', '21483845', '20095037']
PMC3069	4	FALSE	1	FALSE		4	D ['11245843', '18417697', '15953754', '12930483'], S ['21483844']
PMC3069	2	FALSE	1	FALSE		2	D ['9878612', '15078970'], S ['21483843']
PMC3069	2	FALSE	1	FALSE		2	D ['10568647', '18347349'], S ['21483492']
PMC3069	2	FALSE	1	FALSE		2	D ['16951642', '16856117'], S ['21483491']
PMC3068	1	FALSE	2	FALSE		1	D ['15249685'], S ['15249690', '21483490']
PMC3068	7	FALSE	6	FALSE		7	D ['17311420', '19476346', '15123599', '20704566', '19650874', '16115873', '20731382 ['14504267', '18815274', '18158333', '17167418', '16543415', '21483489']
PMC3068	2	FALSE	4	FALSE		2	D ['17914356', '18621946'], S ['16361528', '21483488', '14999409', '19888819']
PMC3068	3	FALSE	3	FALSE		3	D ['17559302', '14981505', '18433450'], S ['15784612', '21483487', '12350375']
PMC3068	4	FALSE	1	FALSE		4	D ['19279100', '18689690', '12239322', '20613878'], S ['21483486']
PMC3068	4	FALSE	6	FALSE		4	D ['18054063', '12596895', '19114283', '16909114'], S ['21483485', '11878911', '16873 '10559321', '15950254', '11698564']
PMC3068	0	TRUE	5	FALSE		0	S ['11069647', '16360685', '20672876', '21483484', '21199566']
PMC3077	0	TRUE	1	FALSE		0	S ['21453454']
PMC3072	1	FALSE	2	FALSE		1	D ['19911855'], S ['21490947', '19351876']
PMC3072	2	FALSE	1	FALSE		2	D ['8165688', '16564423'], S ['21490946']
PMC3072	0	TRUE	1	FALSE		0	S ['21490945']
PMC3072	4	FALSE	1	FALSE		4	D ['18547134', '12809956', '20385971', '15291376'], S ['21490943']
PMC3077	5	FALSE	2	FALSE		5	D ['16123451', '17005678', '11867539', '15572445', '18024889'], S ['21453528', '20631
PMC3072	5	FALSE	1	FALSE		5	D ['12515388', '9882318', '17067712', '12388732', '18077729'], S ['21450110']
PMC3073	0	TRUE	1	FALSE		0	S ['21450092']
PMC3072	3	FALSE	1	FALSE		3	D ['18677371', '20459289', '19079481'], S ['21483623']
PMC3072	1	FALSE	1	FALSE		1	D ['19546980'], S ['21483622']
PMC3078	0	TRUE	1	FALSE		0	S ['21450071']
PMC3078	1	FALSE	2	FALSE		1	D ['19752750'], S ['17005721', '21447194']
PMC3078	1	FALSE	1	FALSE		1	D ['19135160'], S ['21450094']
PMC3072	2	FALSE	1	FALSE		2	D ['15888674', '17145706'], S ['21450055']
PMC3078	0	TRUE	1	FALSE		0	S ['21450063']
PMC3072	1	FALSE	2	FALSE		1	D ['21450083'], S ['21450084', '19622552']
PMC3073	0	TRUE	1	FALSE		0	S ['21447199']

578']
'], S
259',
086']

PMC3078	4	FALSE	2	FALSE		4	D ['11466298', '8253682', '12142431', '19060154'], S ['21450087', '15882425']
PMC3078	0	TRUE	1	FALSE		0	S ['21450100']
PMC3072	5	FALSE	1	FALSE		5	D ['20491547', '12715790', '15178207', '19367054', '19321945'], S ['21447190']
PMC3076	1	FALSE	1	FALSE		1	D ['16475227'], S ['21447191']
PMC3074	1	FALSE	2	FALSE		1	D ['7782118'], S ['21112930', '21450074']
PMC3078	0	TRUE	1	FALSE		0	S ['21450078']
PMC3078	1	FALSE	1	FALSE		1	D ['19292570'], S ['21447192']
PMC3076	0	TRUE	1	FALSE		0	S ['21450064']
PMC3072	1	FALSE	2	FALSE		1	D ['19119129'], S ['19631310', '21450060']
PMC3076	0	TRUE	1	FALSE		0	S ['21447195']
PMC3068	0	TRUE	1	FALSE		0	S ['21490698']
PMC3074	0	TRUE	1	FALSE		0	S ['21450068']
PMC3072	7	FALSE	1	FALSE		7	D ['17219075', '17539970', '2163747', '19788622', '20554399', '18835104', '15660700'] ['21450103']
PMC3077	3	FALSE	2	FALSE		3	D ['12835928', '18192281', '1459432'], S ['21454301', '19531560']
PMC3072	2	FALSE	3	FALSE		2	D ['16950987', '19619581'], S ['16002379', '21450073', '1437425']
PMC3068	0	TRUE	3	FALSE		0	S ['21490699', '18279338', '14987760']
PMC3071	1	FALSE	0	TRUE		2	D ['12750416']
PMC3070	4	FALSE	2	FALSE		4	D ['14637092', '11245693', '16611826', '17626215'], S ['18522595', '21490706']
PMC3070	4	FALSE	2	FALSE		4	D ['16154280', '12077211', '16061489', '19087170'], S ['21483725', '19535591']
PMC3073	3	FALSE	1	FALSE		3	D ['19076107', '20053237', '20157472'], S ['21450053']
PMC3078	0	TRUE	1	FALSE		0	S ['21450069']
PMC3078	2	FALSE	1	FALSE		2	D ['17299461', '15159240'], S ['21450065']
PMC3076	4	FALSE	1	FALSE		4	D ['17294314', '17043749', '18155624', '15906112'], S ['21494429']
PMC3070	1	FALSE	1	FALSE		1	D ['12516954'], S ['21468286']
PMC3070	9	FALSE	1	FALSE		9	D ['15958488', '15611339', '12972502', '8530366', '11085999', '12475947', '15085953', '8983014', '8175808'], S ['21468280']
PMC3070	1	FALSE	1	FALSE		1	D ['17901231'], S ['21468279']
PMC3070	0	TRUE	2	FALSE		0	S ['21468282', '17565725']
PMC3070	2	FALSE	1	FALSE		2	D ['14665957', '17320698'], S ['21468281']
PMC3073	0	TRUE	4	FALSE		0	S ['16928538', '14667639', '21450095', '19698860']
PMC3072	0	TRUE	1	FALSE		0	S ['21450096']
PMC3076	2	FALSE	1	FALSE		2	D ['1469016', '11808992'], S ['21450058']
PMC3074	0	TRUE	1	FALSE		0	S ['21447188']
PMC3077	5	FALSE	1	FALSE		5	D ['11557112', '10208430', '11555598', '11606373', '17657792'], S ['21450056']
PMC3076	0	TRUE	2	FALSE		0	S ['16421945', '21450101']
PMC3071	2	FALSE	2	FALSE		2	D ['17602036', '15976197'], S ['21490820', '19203992']
PMC3078	1	FALSE	1	FALSE		1	D ['18855536'], S ['21450082']
PMC3078	3	FALSE	1	FALSE		3	D ['11222636', '9608533', '10774729'], S ['21450093']
PMC3073	0	TRUE	2	FALSE		0	S ['18187350', '21447193']
PMC3068	2	FALSE	2	FALSE		2	D ['17627274', '17363249'], S ['19425122', '21483474']
PMC3068	2	FALSE	1	FALSE		2	D ['11829360', '17904581'], S ['21479137']

, S

PMC3068	1	FALSE	2	FALSE		1	D ['2694282'], S ['12603494', '21479136']
PMC3068	4	FALSE	2	FALSE		4	D ['20578089', '19842214', '20552648', '19252504'], S ['21479135', '20529923']
PMC3068	0	TRUE	6	FALSE		0	S ['17322097', '16778135', '21479193', '18574047', '18515970', '18706288']
PMC3068	2	FALSE	3	FALSE		2	D ['17185322', '14973296'], S ['19422819', '20682360', '21479192']
PMC3068	1	FALSE	3	FALSE		1	D ['19528256'], S ['18827405', '16550932', '21479190']
PMC3068	2	FALSE	1	FALSE		2	D ['18560572', '18577081'], S ['21479188']
PMC3068	2	FALSE	1	FALSE		2	D ['8918855', '16029119'], S ['21479187']
PMC3068	1	FALSE	2	FALSE		1	D ['16827008'], S ['18686583', '21479185']
PMC3068	2	FALSE	1	FALSE		2	D ['19075246', '19588176'], S ['21479184']
PMC3068	1	FALSE	1	FALSE		1	D ['15612297'], S ['21479183']
PMC3068	0	TRUE	2	FALSE		0	S ['20209167', '21479182']
PMC3068	3	FALSE	1	FALSE		3	D ['17700634', '21187961', '19943892'], S ['21479181']
PMC3068	4	FALSE	3	FALSE		4	D ['20380293', '18470566', '2519691', '19496429'], S ['20032111', '20828381', '214791[illegible]
PMC3068	1	FALSE	1	FALSE		1	D ['20072610'], S ['21479179']
PMC3068	3	FALSE	5	FALSE		3	D ['18978042', '12773183', '12180915'], S ['11007775', '15554971', '18931290', '21479[illegible] '12218047']
PMC3068	0	TRUE	2	FALSE		0	S ['18459962', '21479177']
PMC3068	0	TRUE	2	FALSE		0	S ['18804554', '21479175']
PMC3068	3	FALSE	1	FALSE		3	D ['19234208', '12939333', '17911633'], S ['21479174']
PMC3068	3	FALSE	1	FALSE		3	D ['9634232', '19443505', '11747570'], S ['21479173']
PMC3068	0	TRUE	1	FALSE		0	S ['21479172']
PMC3068	0	TRUE	1	FALSE		0	S ['21479170']
PMC3068	1	FALSE	1	FALSE		1	D ['19234297'], S ['21479169']
PMC3068	0	TRUE	1	FALSE		0	S ['21479168']
PMC3068	1	FALSE	2	FALSE		1	D ['16775236'], S ['12237133', '21479165']
PMC3068	1	FALSE	1	FALSE		1	D ['16013437'], S ['21479164']
PMC3068	0	TRUE	2	FALSE		0	S ['21479223', '19737795']
PMC3068	2	FALSE	2	FALSE		2	D ['16610953', '18596870'], S ['16049284', '21479222']
PMC3068	2	FALSE	1	FALSE		2	D ['19525919', '18728010'], S ['21479221']
PMC3068	0	TRUE	5	FALSE		0	S ['19047102', '14520474', '21479220', '12085212', '17313687']
PMC3068	0	TRUE	1	FALSE		0	S ['21479219']
PMC3068	1	FALSE	3	FALSE		1	D ['3944607'], S ['21479218', '20488197', '20101094']
PMC3068	0	TRUE	2	FALSE		0	S ['17264230', '21479217']
PMC3068	4	FALSE	1	FALSE		4	D ['18255067', '19084530', '18638479', '17295295'], S ['21479215']
PMC3068	0	TRUE	2	FALSE		0	S ['21479214', '20015316']
PMC3068	1	FALSE	1	FALSE		1	D ['18788805'], S ['21479212']
PMC3068	1	FALSE	2	FALSE		1	D ['15891831'], S ['21479211', '17584225']
PMC3068	0	TRUE	1	FALSE		0	S ['21479209']
PMC3068	3	FALSE	1	FALSE		3	D ['20098712', '9737583', '20147404'], S ['21479208']
PMC3068	3	FALSE	1	FALSE		3	D ['20458013', '18363904', '17076270'], S ['21479207']
PMC3068	0	TRUE	1	FALSE		0	S ['21479206']
PMC3068	0	TRUE	1	FALSE		0	S ['21479205']

80']

178',

PMC3068	2	FALSE	2	FALSE		2	D ['9609863', '12668541'], S ['21479204', '16483330']
PMC3068	3	FALSE	1	FALSE		3	D ['11561031', '15539719', '12874394'], S ['21479202']
PMC3070	1	FALSE	1	FALSE		1	D ['8413003'], S ['21464881']
PMC3072	2	FALSE	1	FALSE		2	D ['20595148', '21112201'], S ['21450080']
PMC3077	1	FALSE	1	FALSE		1	D ['12208907'], S ['21447197']
PMC3076	0	TRUE	1	FALSE		0	S ['21450089']
PMC3074	5	FALSE	1	FALSE		5	D ['8790886', '20952706', '16096706', '9201400', '18459299'], S ['21447189']
PMC3061	1	FALSE	1	FALSE		1	D ['8389699'], S ['21262516']
PMC3072	0	TRUE	1	FALSE		0	S ['21483621']
PMC3072	3	FALSE	1	FALSE		3	D ['19724557', '15945142', '16544620'], S ['21483620']
PMC3072	0	TRUE	1	FALSE		0	S ['21447148']
PMC3076	0	TRUE	1	FALSE		0	S ['21447161']
PMC3078	4	FALSE	2	FALSE		4	D ['1843763', '3424671', '17015333', '21170644'], S ['21447186', '18638376']
PMC3078	1	FALSE	1	FALSE		1	D ['10830668'], S ['21443806']
PMC3072	0	TRUE	1	FALSE		0	S ['21447185']
PMC3073	8	FALSE	1	FALSE		8	D ['15181822', '16377674', '16438215', '18164184', '20452372', '19470506', '8031045', '17035499'], S ['21443804']
PMC3073	0	TRUE	1	FALSE		0	S ['21447184']
PMC3073	3	FALSE	2	FALSE		3	D ['19527296', '18539794', '16553727'], S ['19309013', '21447177']
PMC3072	0	TRUE	4	FALSE		0	S ['20673790', '19429049', '20458732', '21447183']
PMC3076	5	FALSE	2	FALSE		5	D ['18596111', '17426772', '18419811', '12582493', '18997115'], S ['17721507', '21447
PMC3074	1	FALSE	1	FALSE		1	D ['19784801'], S ['21447169']
PMC3073	0	TRUE	1	FALSE		0	S ['21443805']
PMC3073	1	FALSE	1	FALSE		1	D ['15004046'], S ['21447159']
PMC3073	2	FALSE	1	FALSE		2	D ['19125201', '19585168'], S ['21447175']
PMC3072	0	TRUE	1	FALSE		0	S ['21443808']
PMC3070	2	FALSE	1	FALSE		2	D ['19426475', '18957448'], S ['21447597']
PMC3076	0	TRUE	4	FALSE		0	S ['10843339', '19875153', '19152957', '21446726']
PMC3070	3	FALSE	3	FALSE		3	D ['17475789', '15727536', '14507977'], S ['14657165', '21483781', '10414988']
PMC3070	1	FALSE	4	FALSE		1	D ['9463358'], S ['15978015', '20123022', '21192085', '21483723']
PMC3070	1	FALSE	4	FALSE		1	D ['18987196'], S ['20589098', '16120665', '19350384', '21483724']
PMC3078	0	TRUE	5	FALSE		0	S ['17081744', '20972628', '21447160', '8433390', '20231085']
PMC3078	5	FALSE	2	FALSE		5	D ['18255271', '11982448', '12648735', '16883147', '12920409'], S ['16697151', '21447
PMC3072	0	TRUE	1	FALSE		0	S ['21447181']
PMC3076	0	TRUE	2	FALSE		0	S ['21494430', '17390543']
PMC3077	2	FALSE	1	FALSE		2	D ['19911780', '20455532'], S ['21446660']
PMC3073	1	FALSE	1	FALSE		1	D ['17218783'], S ['21447176']
PMC3078	0	TRUE	1	FALSE		0	S ['21447168']
PMC3076	0	TRUE	1	FALSE		0	S ['21447163']
PMC3075	0	TRUE	3	FALSE		0	S ['18199533', '16061638', '21447180']
PMC3072	0	TRUE	1	FALSE		0	S ['21447152']
PMC3073	0	TRUE	2	FALSE		0	S ['14681431', '21447150']

172']

155']

PMC3066	1	FALSE	1	FALSE		1	D ['15252059'], S ['21468305']
PMC3066	1	FALSE	2	FALSE		1	D ['12682067'], S ['21468359', '16505382']
PMC3066	0	TRUE	1	FALSE		0	S ['21468304']
PMC3066	1	FALSE	1	FALSE		1	D ['19730269'], S ['21468300']
PMC3066	0	TRUE	1	FALSE		0	S ['21468317']
PMC3066	2	FALSE	6	FALSE		2	D ['18831801', '12133654'], S ['17574093', '15119969', '15228717', '19805362', '21468 '15993957']
PMC3066	2	FALSE	1	FALSE		2	D ['7536937', '19108895'], S ['21468314']
PMC3066	2	FALSE	3	FALSE		2	D ['19263854', '16045462'], S ['18291003', '21468313', '16735334']
PMC3066	0	TRUE	1	FALSE		0	S ['21468312']
PMC3066	3	FALSE	3	FALSE		3	D ['18827884', '17134654', '18638509'], S ['16447162', '16269422', '21468311']
PMC3066	1	FALSE	3	FALSE		1	D ['19627614'], S ['21468310', '20868280', '18797648']
PMC3066	2	FALSE	2	FALSE		2	D ['18430913', '19308251'], S ['21468309', '18365031']
PMC3066	0	TRUE	2	FALSE		0	S ['17467515', '21468308']
PMC3066	0	TRUE	1	FALSE		0	S ['21468307']
PMC3066	4	FALSE	3	FALSE		4	D ['19492944', '19815894', '12125863', '20158331'], S ['18613555', '19068139', '21468
PMC3066	0	TRUE	7	FALSE		0	S ['12611817', '11839534', '16857758', '19503810', '21479249', '19234101', '11587983
PMC3066	3	FALSE	2	FALSE		3	D ['20508213', '17912342', '18815611'], S ['19930588', '21479248']
PMC3066	7	FALSE	1	FALSE		7	D ['18583342', '15962216', '19903258', '15271936', '9767584', '18039770', '20487288'] ['21479247']
PMC3066	1	FALSE	1	FALSE		1	D ['16373502'], S ['21479246']
PMC3066	0	TRUE	2	FALSE		0	S ['18678671', '21479245']
PMC3066	0	TRUE	1	FALSE		0	S ['21479244']
PMC3066	2	FALSE	2	FALSE		2	D ['19074218', '11495617'], S ['19364999', '21479243']
PMC3066	0	TRUE	2	FALSE		0	S ['8663154', '21479242']
PMC3066	2	FALSE	1	FALSE		2	D ['20371808', '16860741'], S ['21479241']
PMC3066	1	FALSE	2	FALSE		1	D ['11191259'], S ['17715249', '21479240']
PMC3066	0	TRUE	2	FALSE		0	S ['14629343', '21483472']
PMC3066	4	FALSE	4	FALSE		4	D ['15135522', '16014945', '15254251', '15016896'], S ['19826476', '12022227', '14615 '21479239']
PMC3066	3	FALSE	2	FALSE		3	D ['16528714', '16454359', '20645046'], S ['21031546', '21479238']
PMC3066	2	FALSE	1	FALSE		2	D ['18593687', '18486165'], S ['21479236']
PMC3066	2	FALSE	1	FALSE		2	D ['19119230', '18079392'], S ['21479235']
PMC3066	2	FALSE	2	FALSE		2	D ['11523567', '12484572'], S ['8834542', '21479234']
PMC3066	5	FALSE	1	FALSE		5	D ['19036818', '19179289', '19214216', '19461879', '19474106'], S ['21479233']
PMC3066	1	FALSE	1	FALSE		1	D ['15537460'], S ['21479232']
PMC3066	1	FALSE	2	FALSE		1	D ['14633289'], S ['19038057', '21479231']
PMC3066	1	FALSE	1	FALSE		1	D ['12950387'], S ['21479230']
PMC3066	1	FALSE	1	FALSE		1	D ['18032004'], S ['21479229']
PMC3066	5	FALSE	3	FALSE		5	D ['19676102', '11689490', '18043721', '17568014', '17923635'], S ['18449188', '20740 '21479228']
PMC3066	3	FALSE	1	FALSE		3	D ['8565856', '15753038', '9611173'], S ['21479227']

315',
306']
']
, S
487',
007',

PMC3066	2	FALSE	2	FALSE		2	D ['8530391', '10229682'], S ['16406194', '21479226']
PMC3066	4	FALSE	1	FALSE		4	D ['17006666', '20348245', '20299475', '20133650'], S ['21479224']
PMC3066	3	FALSE	2	FALSE		3	D ['12234808', '14656708', '11303020'], S ['20053925', '21479273']
PMC3066	7	FALSE	1	FALSE		7	D ['18087008', '20107721', '19002339', '17157411', '17052489', '17553036', '19528427<br>['21479272']
PMC3066	2	FALSE	3	FALSE		2	D ['19005073', '17045251'], S ['12193747', '10748238', '21479271']
PMC3066	2	FALSE	7	FALSE		2	D ['19754158', '20081836'], S ['17323929', '17481360', '14990965', '18601527', '17685<br>'16716067', '21479270']
PMC3066	2	FALSE	3	FALSE		2	D ['17171570', '10552926'], S ['14629112', '21479269', '11683905']
PMC3066	3	FALSE	2	FALSE		3	D ['19389431', '19477903', '11714518'], S ['20453122', '21479268']
PMC3066	1	FALSE	1	FALSE		1	D ['9705459'], S ['21479267']
PMC3066	0	TRUE	4	FALSE		0	S ['19953606', '9855520', '16501573', '21479265']
PMC3066	1	FALSE	2	FALSE		1	D ['15014631'], S ['18067659', '21479264']
PMC3066	3	FALSE	1	FALSE		3	D ['15309610', '17479760', '14973484'], S ['21479262']
PMC3066	6	FALSE	1	FALSE		6	D ['18710875', '16882992', '20080507', '18287176', '17887954', '17189479'], S ['21468
PMC3066	3	FALSE	1	FALSE		3	D ['16420734', '17090325', '19946141'], S ['21468321']
PMC3066	0	TRUE	3	FALSE		0	S ['9203535', '9037487', '21483471']
PMC3066	1	FALSE	3	FALSE		1	D ['19203589'], S ['20627413', '16697110', '21468319']
PMC3066	2	FALSE	2	FALSE		2	D ['19779633', '15908579'], S ['21468318', '20018765']
PMC3072	3	FALSE	1	FALSE		3	D ['12714744', '18710654', '17044029'], S ['21447182']
PMC3077	6	FALSE	1	FALSE		6	D ['20371060', '19835577', '18823818', '19805305', '17234809', '18684813'], S ['21447
PMC3072	0	TRUE	4	FALSE		0	S ['21136184', '18345013', '21408169', '21483619']
PMC3073	1	FALSE	1	FALSE		1	D ['15063579'], S ['21443770']
PMC3078	0	TRUE	2	FALSE		0	S ['21443778', '11308089']
PMC3073	1	FALSE	1	FALSE		1	D ['15081214'], S ['21443765']
PMC3078	3	FALSE	2	FALSE		3	D ['19097638', '17223200', '16669716'], S ['21443801', '19247559']
PMC3074	0	TRUE	2	FALSE		0	S ['17211576', '21443787']
PMC3073	1	FALSE	1	FALSE		1	D ['18500980'], S ['21443802']
PMC3072	1	FALSE	2	FALSE		1	D ['19633998'], S ['20921541', '21443790']
PMC3078	2	FALSE	1	FALSE		2	D ['19582582', '18366690'], S ['21443761']
PMC3073	0	TRUE	1	FALSE		0	S ['21443803']
PMC3072	0	TRUE	7	FALSE		0	S ['18076744', '14606628', '14566938', '12403177', '21439093', '20596906', '17115331
PMC3076	0	TRUE	4	FALSE		0	S ['18429614', '21443768', '16097768', '15053574']
PMC3072	3	FALSE	1	FALSE		3	D ['20854656', '9611795', '15673735'], S ['21443791']
PMC3072	1	FALSE	2	FALSE		1	D ['18803985'], S ['19144173', '21487451']
PMC3076	0	TRUE	1	FALSE		0	S ['21499565']
PMC3077	2	FALSE	1	FALSE		2	D ['16987977', '20723216'], S ['21444341']
PMC3077	0	TRUE	1	FALSE		0	S ['21444340']
PMC3069	1	FALSE	1	FALSE		1	D ['21441983'], S ['21483762']
PMC3069	6	FALSE	1	FALSE		6	D ['12880847', '9626288', '15006680', '10698062', '10102425', '9445333'], S ['2148376
PMC3078	0	TRUE	1	FALSE		0	S ['21443780']
PMC3072	0	TRUE	1	FALSE		0	S ['21443766']

'], S
554',
322']
170']
']
1']

PMC3078	1	FALSE	1	FALSE		1	D ['10395870'], S ['21443769']
PMC3065	1	FALSE	1	FALSE		1	D ['15170709'], S ['21461336']
PMC3065	11	FALSE	2	FALSE		11	D ['10574708', '12015115', '17662976', '11331308', '18327251', '12670870', '12736206 '11304546', '12432066', '10022929', '19435803'], S ['11238456', '21444757']
PMC3069	0	TRUE	1	FALSE		0	S ['21468271']
PMC3069	5	FALSE	1	FALSE		5	D ['15471123', '18392724', '20382597', '18971878', '19881238'], S ['21468270']
PMC3069	3	FALSE	1	FALSE		3	D ['10353502', '10534592', '11790474'], S ['21468266']
PMC3069	1	FALSE	1	FALSE		1	D ['18477341'], S ['21468265']
PMC3069	1	FALSE	1	FALSE		1	D ['17643591'], S ['21468259']
PMC3069	2	FALSE	1	FALSE		2	D ['17940077', '18459994'], S ['21468258']
PMC3069	0	TRUE	1	FALSE		0	S ['21468257']
PMC3069	3	FALSE	1	FALSE		3	D ['19815859', '19822627', '20159893'], S ['21468256']
PMC3069	2	FALSE	1	FALSE		2	D ['18449604', '17632425'], S ['21468255']
PMC3069	1	FALSE	1	FALSE		1	D ['17451370'], S ['21468254']
PMC3069	1	FALSE	2	FALSE		1	D ['19171608'], S ['14966337', '21468252']
PMC3076	0	TRUE	1	FALSE		0	S ['21443763']
PMC3073	5	FALSE	1	FALSE		5	D ['10229652', '1106839', '12050021', '12033599', '2804473'], S ['21443799']
PMC3078	0	TRUE	1	FALSE		0	S ['21439091']
PMC3077	1	FALSE	4	FALSE		1	D ['7891866'], S ['19393862', '15870954', '21443795', '18478238']
PMC3078	3	FALSE	7	FALSE		3	D ['20089138', '16930406', '17021023'], S ['14749441', '21443797', '19590003', '93253 '15497039', '17407590', '16113037']
PMC3074	3	FALSE	1	FALSE		3	D ['17484375', '15979893', '18772584'], S ['21439094']
PMC3065	2	FALSE	1	FALSE		2	D ['12228019', '15242524'], S ['21464908']
PMC3065	5	FALSE	1	FALSE		5	D ['19939974', '15579160', '18343358', '19300505', '19633195'], S ['21464907']
PMC3065	0	TRUE	2	FALSE		0	S ['19435461', '21464905']
PMC3065	7	FALSE	4	FALSE		7	D ['9425234', '19459154', '15240799', '12732716', '17267005', '15735604', '18706439'] ['11484154', '19941038', '21464903', '15953638']
PMC3065	3	FALSE	4	FALSE		3	D ['18256280', '18755006', '18044991'], S ['19937774', '20177055', '21464901', '19052
PMC3065	1	FALSE	2	FALSE		1	D ['12614837'], S ['21464898', '19217764']
PMC3065	0	TRUE	2	FALSE		0	S ['21464896', '18362256']
PMC3065	0	TRUE	2	FALSE		0	S ['19182060', '21464895']
PMC3065	1	FALSE	1	FALSE		1	D ['19616766'], S ['21464894']
PMC3065	1	FALSE	4	FALSE		1	D ['19707197'], S ['15033932', '18627300', '21464892', '18778305']
PMC3065	6	FALSE	2	FALSE		6	D ['18210383', '1687577', '10455629', '12804080', '8961174', '15527912'], S ['1951376 '21464891']
PMC3065	0	TRUE	4	FALSE		0	S ['19010416', '17645533', '21464890', '18082973']
PMC3065	0	TRUE	4	FALSE		0	S ['9106808', '21464942', '11098101', '19570226']
PMC3065	1	FALSE	1	FALSE		1	D ['19378336'], S ['21464941']
PMC3065	0	TRUE	1	FALSE		0	S ['21464940']
PMC3065	6	FALSE	3	FALSE		6	D ['17075435', '17710126', '18711774', '10592848', '16819172', '15958519'], S ['21464 '19423264', '21219992']
PMC3065	0	TRUE	1	FALSE		0	S ['21464937']

',
65',
, S
223']
3',
939',

PMC3065	1	FALSE	2	FALSE		1	D ['12817185'], S ['11230970', '21464936']
PMC3065	0	TRUE	2	FALSE		0	S ['21464934', '19064345']
PMC3065	6	FALSE	5	FALSE		6	D ['10808092', '16965698', '17193877', '19661209', '12354864', '19185432'], S ['19413 '21208459', '21464930', '19603075', '19543404']
PMC3065	6	FALSE	6	FALSE		6	D ['18813227', '20702615', '19153461', '17475623', '18987632', '10024172'], S ['201338 '12438621', '18585749', '14645582', '18796704', '21464929']
PMC3065	3	FALSE	2	FALSE		3	D ['15705809', '17623807', '15723063'], S ['17609980', '21464928']
PMC3065	4	FALSE	2	FALSE		4	D ['11735240', '12435367', '10368936', '16191163'], S ['21464927', '19124089']
PMC3065	1	FALSE	1	FALSE		1	D ['19002529'], S ['21464926']
PMC3065	1	FALSE	2	FALSE		1	D ['15380044'], S ['21464925', '16339583']
PMC3065	0	TRUE	4	FALSE		0	S ['10862526', '11903872', '21464924', '9232546']
PMC3065	5	FALSE	2	FALSE		5	D ['15733667', '19771161', '17631442', '12408806', '16107473'], S ['19712730', '21464
PMC3065	3	FALSE	5	FALSE		3	D ['15748755', '17349705', '20399521'], S ['17148126', '20161769', '21464920', '14634 '17566777']
PMC3065	0	TRUE	2	FALSE		0	S ['21464919', '14963115']
PMC3065	0	TRUE	2	FALSE		0	S ['21464918', '19551239']
PMC3070	5	FALSE	1	FALSE		5	D ['15995029', '18254012', '15060233', '9627595', '14734882'], S ['21468324']
PMC3073	2	FALSE	1	FALSE		2	D ['20632560', '18975688'], S ['21439096']
PMC3072	0	TRUE	3	FALSE		0	S ['20042117', '21443794', '19091083']
PMC3074	2	FALSE	2	FALSE		2	D ['19134177', '17711567'], S ['21439095', '16691271']
PMC3078	0	TRUE	1	FALSE		0	S ['21443789']
PMC3072	3	FALSE	1	FALSE		3	D ['18506129', '15591005', '9330130'], S ['21490942']
PMC3070	3	FALSE	1	FALSE		3	D ['11044052', '15517291', '15723217'], S ['21472133']
PMC3070	1	FALSE	1	FALSE		1	D ['11041397'], S ['21472132']
PMC3070	2	FALSE	2	FALSE		2	D ['20112343', '9370944'], S ['16830362', '21472131']
PMC3070	3	FALSE	1	FALSE		3	D ['10075360', '11981681', '11799296'], S ['21472130']
PMC3070	1	FALSE	1	FALSE		1	D ['15809755'], S ['21472129']
PMC3070	0	TRUE	1	FALSE		0	S ['21472128']
PMC3070	1	FALSE	1	FALSE		1	D ['19212172'], S ['21472127']
PMC3070	0	TRUE	1	FALSE		0	S ['21472126']
PMC3070	0	TRUE	1	FALSE		0	S ['21472125']
PMC3070	1	FALSE	1	FALSE		1	D ['18573233'], S ['21472124']
PMC3070	0	TRUE	2	FALSE		0	S ['21472123', '10338220']
PMC3070	2	FALSE	1	FALSE		2	D ['18694399', '20002296'], S ['21472120']
PMC3070	2	FALSE	1	FALSE		2	D ['18324677', '17112622'], S ['21472115']
PMC3070	5	FALSE	1	FALSE		5	D ['17211138', '17973645', '19774643', '14517348', '10647628'], S ['21472114']
PMC3078	1	FALSE	1	FALSE		1	D ['10597222'], S ['21439087']
PMC3076	6	FALSE	1	FALSE		6	D ['17029808', '10584814', '16411159', '15838205', '17122543', '14965570'], S ['21494
PMC3066	0	TRUE	1	FALSE		0	S ['21490697']
PMC3076	1	FALSE	1	FALSE		1	D ['10698529'], S ['21499554']
PMC3078	2	FALSE	1	FALSE		2	D ['20309404', '17620749'], S ['21439089']
PMC3074	2	FALSE	1	FALSE		2	D ['20837075', '18455515'], S ['21487538']

493',
840',
923']
671',
401']

PMC3073	3	FALSE	1	FALSE		3	D ['9834262', '14512287', '15940617'], S ['21439078']
PMC3072	0	TRUE	1	FALSE		0	S ['21439077']
PMC3072	0	TRUE	2	FALSE		0	S ['21439080', '19581290']
PMC3072	2	FALSE	1	FALSE		2	D ['17904097', '20655035'], S ['21439084']
PMC3073	2	FALSE	1	FALSE		2	D ['1750006', '20043768'], S ['21439083']
PMC3078	0	TRUE	1	FALSE		0	S ['21439082']
PMC3077	2	FALSE	1	FALSE		2	D ['12135316', '17893310'], S ['21499494']
PMC3077	0	TRUE	1	FALSE		0	S ['21499496']
PMC3077	0	TRUE	1	FALSE		0	S ['21499495']
PMC3071	1	FALSE	1	FALSE		1	D ['18006120'], S ['21439029']
PMC3073	0	TRUE	1	FALSE		0	S ['21435275']
PMC3071	2	FALSE	1	FALSE		2	D ['11520714', '16208676'], S ['21439045']
PMC3072	0	TRUE	2	FALSE		0	S ['17010387', '21483617']
PMC3072	0	TRUE	2	FALSE		0	S ['10795991', '21483616']
PMC3076	0	TRUE	1	FALSE		0	S ['21439037']
PMC3073	1	FALSE	2	FALSE		1	D ['19398573'], S ['21439039', '19179548']
PMC3071	2	FALSE	1	FALSE		2	D ['15214025', '9707603'], S ['21439073']
PMC3074	0	TRUE	1	FALSE		0	S ['21435277']
PMC3070	0	TRUE	1	FALSE		0	S ['21435273']
PMC3076	5	FALSE	2	FALSE		5	D ['18285835', '17873650', '17467974', '17363414', '17621639'], S ['21439053', '17850
PMC3073	0	TRUE	1	FALSE		0	S ['21439033']
PMC3074	4	FALSE	3	FALSE		4	D ['12142416', '8253685', '12193624', '21299642'], S ['18757823', '15317765', '214390
PMC3076	0	TRUE	1	FALSE		0	S ['21439041']
PMC3078	1	FALSE	2	FALSE		1	D ['16826162'], S ['19417579', '21439069']
PMC3078	2	FALSE	1	FALSE		2	D ['19576671', '16670031'], S ['21439043']
PMC3078	0	TRUE	1	FALSE		0	S ['21439040']
PMC3072	0	TRUE	1	FALSE		0	S ['21439062']
PMC3071	1	FALSE	1	FALSE		1	D ['16238724'], S ['21439059']
PMC3071	0	TRUE	1	FALSE		0	S ['21439049']
PMC3071	0	TRUE	2	FALSE		0	S ['21439044', '18631284']
PMC3073	0	TRUE	1	FALSE		0	S ['21439058']
PMC3066	1	FALSE	1	FALSE		1	D ['20208465'], S ['21472032']
PMC3072	1	FALSE	2	FALSE		1	D ['20303665'], S ['21439051', '20236172']
PMC3072	0	TRUE	1	FALSE		0	S ['21439047']
PMC3063	0	TRUE	1	FALSE		0	S ['21468248']
PMC3072	0	TRUE	1	FALSE		0	S ['21439052']
PMC3065	0	TRUE	2	FALSE		0	S ['20682291', '21448341']
PMC3076	0	TRUE	1	FALSE		0	S ['21439055']
PMC3072	0	TRUE	1	FALSE		0	S ['21439030']
PMC3071	5	FALSE	1	FALSE		5	D ['9040939', '18343598', '12231537', '12810677', '16551475'], S ['21439064']
PMC3072	1	FALSE	1	FALSE		1	D ['2549063'], S ['21439072']
PMC3072	2	FALSE	1	FALSE		2	D ['19319544', '20684749'], S ['21439035']

622']
67']

PMC3071	3	FALSE	1	FALSE		3	D ['21179021', '19536200', '19838166'], S ['21435272']
PMC3071	1	FALSE	1	FALSE		1	D ['12672206'], S ['21439056']
PMC3070	1	FALSE	1	FALSE		1	D ['9862558'], S ['21483704']
PMC3064	1	FALSE	4	FALSE		1	D ['14530484'], S ['20473899', '20155263', '21464917', '20484421']
PMC3064	3	FALSE	5	FALSE		3	D ['19458183', '18716230', '19915672'], S ['18550737', '17005759', '18234864', '17192 '21464915']
PMC3064	0	TRUE	1	FALSE		0	S ['21464914']
PMC3064	1	FALSE	4	FALSE		1	D ['20518707'], S ['10960092', '11489869', '21464913', '9622362']
PMC3064	1	FALSE	1	FALSE		1	D ['18291435'], S ['21464971']
PMC3064	0	TRUE	1	FALSE		0	S ['21464969']
PMC3064	0	TRUE	1	FALSE		0	S ['21464968']
PMC3064	1	FALSE	1	FALSE		1	D ['1940799'], S ['21464967']
PMC3064	0	TRUE	1	FALSE		0	S ['21464966']
PMC3064	2	FALSE	3	FALSE		2	D ['18077311', '19474064'], S ['21464963', '20118165', '17374506']
PMC3064	1	FALSE	2	FALSE		1	D ['17240341'], S ['21464962', '20485685']
PMC3064	1	FALSE	1	FALSE		1	D ['10454621'], S ['21464961']

416',
