# Supplementary material for: Using Noun Phrases for Navigating Biomedical Literature on Pubmed: How Many Updates Are We Losing Track of?
Source: PLoS One. 2011 Sep 14;6(9):e24920. doi: 10.1371/journal.pone.0024920 (PMC3173492; doi:10.1371/journal.pone.0024920)
Supplement: Table S2 — Measurements for citation validated searches. This table lists each of the 883 papers by Pubmed Central identifier with citation-validated search terms (CV-D and CV-S). The data in this table was used in Figures 3, 4, and 6. (PDF) [file pone.0024920.s002.pdf]

PMC ID	CV-D	CV-D > 0	CV-S	CV-S > 0		Unique PubMed citations	Non-overlappin search results >= 1	670	Non-overlappin search results >= 5	771	Non-overlappin search results >= 10	816	Non-overlappin search results >= 20	
PMC3072200	3	FALSE	7	FALSE	123	5	10	TRUE	9	TRUE	7	TRUE	6	TRU
PMC3077826	6	FALSE	1	FALSE	16	7	6	TRUE	5	TRUE	5	TRUE	5	TRU
PMC3063339	8	FALSE	11	FALSE		9	16	TRUE	14	TRUE	10	TRUE	7	TRU
PMC3062632	14	FALSE	7	FALSE		12	20	TRUE	16	TRUE	15	TRUE	14	TRU
PMC3077071	1	FALSE	15	FALSE		5	13	TRUE	12	TRUE	11	TRUE	10	TRU

852	868	Non-overlappin search results >= 100	839	Searches
JE	TRUE	4	TRUE	efficacy estimates for uptake (59 results) S ['20072614'], available cardiovascular risk scores (191 results) S ['21474616'], QRISK (20 results) D ['18573856', '17615182'], risk predictors (386 results) S ['21474616'], cardiovascular risk scores (54 results) S ['21474616'], prior use of platelet (5213 results) S ['21474616'], risk scores (1361 results) S ['21474616'], QRISK2 (8 results) D ['20466793', '18573856'], QRISK2 (8 results) S ['21474616'], validation study of QRISK (5 results) D ['18573856', '17615182']
JE	TRUE	1	TRUE	automatic precision estimates (25 results) D ['18817555'], Abbreviation definition identification (1 results) D ['18817555'], biological literature (139 results) D ['19958517'], dictionary of abbreviations (34 results) D ['20360059', '17050571', '16982707', '12386112'], dictionary of abbreviations (34 results) S ['15905486'], abbreviations from MEDLINE (42 results) D ['20360059', '18817555', '17050571', '16982707'], online dictionary of abbreviations (3 results) D ['12386112']
JE	TRUE	2	TRUE	pathway scaffold (10 results) D ['10712921'], pathway scaffold (10 results) S ['21237154'], cleavage of JIP (3 results) D ['12640031'], cleavage of JIP (3 results) S ['21237154'], JIP scaffold proteins (59 results) D ['17052208'], JIP scaffold proteins (59 results) S ['21237154'], JIP1 (64 results) S ['21237154'], JNK pathway scaffold protein (110 results) S ['21237154'], JIP1 cleavage (6 results) S ['21237154'], JIP scaffold (59 results) D ['17052208'], JIP scaffold (59 results) S ['21237154'], pathway scaffold protein JIP1 (15 results) D ['15767678', '11562351', '10490659'], pathway scaffold protein JIP1 (15 results) S ['21237154'], cleavage of JIP1 (6 results) S ['21237154'], JNK pathway scaffold (110 results) S ['21237154'], differential requirements for caspase (13 results) S ['10794718'], Ghayur Wong Kamen Weichselbaum (1 results) D ['8557034'], Kharbanda Robertson Ghayur (1 results) D ['8557034'], Robertson Ghayur Wong Kamen (1 results) D ['8557034']
JE	TRUE	9	TRUE	reveal autophagic neurodegeneration (5 results) D ['14985441'], ataxia exhibit oxidative stress (21 results) D ['16919418'], ataxia exhibit oxidative (26 results) D ['16919418'], frataxin (621 results) S ['21315377'], ataxia reveal autophagic neurodegeneration (1 results) D ['14985441'], Friedreich's point (71 results) D ['20495089', '16911956'], Friedreich's (1598 results) S ['21315377'], Friedreich ataxia mouse (96 results) D ['19805308', '18815198', '18725397', '18621680', '18463734'], FRDA (398 results) S ['21315377'], Friedreich ataxia exhibit oxidative (8 results) D ['16919418'], Friedreich's autosomal recessive (151 results) D ['18621680'], Friedreich's autosomal recessive (151 results) S ['21315377'], ataxia mouse (5 results) D ['18463734', '14985441', '10767347'], Friedreich's autosomal recessive disease (104 results) D ['18621680'], Friedreich's autosomal recessive disease (104 results) S ['21315377'], Friedreich's point mutations (51 results) D ['16911956'], Friedreich ataxia exhibit (20 results) D ['16919418', '11175786'], Friedreich's autosomal (187 results) S ['21315377'], Friedreich's ataxia (1384 results) S ['21315377'], ataxia reveal autophagic (2 results) D ['14985441'], sensory ataxia reveal autophagic (2 results) D ['14985441']
JE	TRUE	5	TRUE	prediction of membrane (3917 results) S ['21493661'], protein topology predictions (5 results) S ['15215532'], membrane protein topology (105 results) S ['21493661', '19429891', '18477697'], transmembrane protein topology (31 results) D ['15111065'], transmembrane protein topology (31 results) S ['15215532'], TOPCONS algorithm (1 results) S ['19429891'], TOPCONS-single performs (1 results) S ['21493661'], consensus prediction of membrane (76 results) S ['21493661', '19429891'], entire international protein (278 results) S ['21493661'], consensus prediction (70 results) S ['19429891'], topology predictions (37 results) S ['21493661', '19429891'], entire international protein index (17 results) S ['21493661'], protein topology (297 results) S ['21493661'], membrane protein (30795 results) S ['21493661'], TOPCONS-single (1 results) S ['21493661'], Viklund Elofsson (10 results) S ['19429891', '18477697', '15215532']

PMC3078977	6	FALSE	9	FALSE		7	10	TRUE	7	TRUE	6	TRUE	5	TRU
PMC3077211	0	TRUE	6	FALSE		3	6	FALSE	6	TRUE	5	TRUE	5	TRU
PMC3078664	0	TRUE	10	FALSE		3	9	FALSE	9	TRUE	7	TRUE	7	TRU
PMC3073994	4	FALSE	6	FALSE		8	7	TRUE	5	TRUE	4	TRUE	4	TRU
PMC3073993	2	FALSE	17	FALSE		2	18	TRUE	16	TRUE	15	TRUE	13	TRU
PMC3073990	9	FALSE	6	FALSE		12	15	TRUE	14	TRUE	14	TRUE	13	TRU

JE	TRUE	5	TRUE	Cancer Res (47408 results) S ['21487065'], international blind trial (3 results) D ['12883091'], methyltransferase-P140K allows intensive (1 results) S ['14707273'], hematopoietic cells (9658 results) S ['21487065'], xenograft model (9715 results) S ['21487065'], continuous administration of o6-benzylguanine (8 results) D ['18006772', '16192602'], O6-benzylguanine (296 results) D ['19204199', '18006772'], functional arrest of immature (102 results) D ['20668220'], DNA methyltransferase-P140K allows (1 results) S ['14707273'], methyltransferase-P140K allows intensive treatment (1 results) S ['14707273'], myelosuppressive xenobiotics (2 results) D ['12883091'], single-dose temozolomide (19 results) D ['18006772', '16192602'], DNA methyltransferase-P140K allows intensive (1 results) S ['14707273'], methyltransferase-P140K allows (1 results) S ['14707273'], DNA methyltransferase-P140K (5 results) S ['14707273']
JE	TRUE	4	TRUE	male parolees (11 results) S ['21499448', '19861321', '19461219'], interpretive (3561 results) S ['21499448'], understanding for practice (24115 results) S ['21499448'], Todres method (31 results) S ['21499448'], embodied (2058 results) S ['21499448'], phenomenological (7778 results) S ['21499448']
JE	TRUE	5	TRUE	surveillance protocol (297 results) S ['21486880'], Paillier cryptosystem (1 results) S ['21486880'], Paillier (9 results) S ['21486880'], practical surveillance protocol (174 results) S ['21486880'], practical method for providers (549 results) S ['21486880'], cryptosystem (34 results) S ['21486880'], patient data for sentinel (2129 results) S ['21486880'], re-identification (62 results) S ['20361870', '19074299'], re-identification risk (8 results) S ['20361870', '19074299'], confidentiality needs of providers (135 results) S ['21486880']
JE	TRUE	2	TRUE	intertype recombinant between genotypes (2 results) S ['15784891', '15215682'], Indian chronic HBV isolates (3 results) D ['18985816', '16847957'], recombinant between genotypes (6934 results) S ['21494570'], intertype recombinant (7 results) S ['15784891', '15215682'], ape genotypes (2 results) D ['19640977', '16306618'], new intertype (4 results) S ['15784891'], clinical implication of core (55 results) D ['18318825'], new intertype recombinant (1 results) S ['15784891'], Indian chronic HBV (54 results) D ['18985816'], HBV recombinant (1661 results) S ['21494570']
JE	TRUE	12	TRUE	variables on conscious (1524 results) S ['21494569'], conscious experience (304 results) S ['21494569'], emotion on conscious (2350 results) S ['21494569'], anterior cingulate (6070 results) S ['21494569'], subliminal below processing (16 results) S ['21494569'], anterior cingulate cortex (3259 results) S ['21494569'], trials with faces (3255 results) S ['21494569'], versus unconscious visual discrimination (8 results) D ['7597090'], subliminal (1264 results) S ['21494569'], cingulate cortex (5442 results) S ['21494569'], interaction between emotion (5136 results) S ['21494569'], impact of emotion (7710 results) S ['21494569'], direct evidence of interaction (5047 results) S ['21494569'], cingulate (11558 results) S ['21494569'], measures of reportability (2 results) S ['21494569'], subliminal trials (3 results) S ['21494569'], reportability (26 results) S ['21494569'], conscious versus unconscious visual (19 results) D ['7597090'], measures conscious reportability (1 results) S ['21494569']
JE	TRUE	8	TRUE	Risk factors for lactic (670 results) S ['21494566'], hyperlactataemia (153 results) D ['19702629'], transcriptase inhibitor lactic (74 results) D ['17713158', '17578788'], generic fixed-dose antiretroviral incidence (13 results) D ['19732926'], reverse transcriptase inhibitor lactic (369 results) D ['19732926', '19702629'], factors for lactic (6554 results) S ['21494566'], severe hyperlactataemia (8 results) D ['19702629', '19043928', '18025882', '12131210'], lactic acidosis (5101 results) S ['21494566'], transcriptase inhibitor lactic acidosis (45 results) D ['17713158', '17578788', '17019496', '16515487'], hyperlactatemia (464 results) S ['21494566'], generic fixed-dose antiretroviral (40 results) D ['19732926'], antiretroviral incidence (8673 results) S ['21494566'], symptomatic hyperlactatemia (27 results) D ['18090299', '17578788', '14600521'], acidosis (34491 results) S ['21494566'], fixed-dose antiretroviral incidence (34 results) D ['19732926']

PMC3073989	6	FALSE	5	FALSE		7	11	TRUE	10	TRUE	8	TRUE	7	TRU
PMC3073988	3	FALSE	17	FALSE		7	15	TRUE	10	TRUE	9	TRUE	8	TRU
PMC3073987	3	FALSE	1	FALSE		3	4	FALSE	4	FALSE	4	TRUE	3	TRU
PMC3073986	4	FALSE	7	FALSE		3	11	TRUE	10	TRUE	8	TRUE	6	TRU
PMC3073985	6	FALSE	11	FALSE		4	14	TRUE	13	TRUE	8	TRUE	7	TRU
PMC3073979	4	FALSE	9	FALSE		6	13	TRUE	11	TRUE	10	TRUE	8	TRU

JE	TRUE	7	TRUE	depletion ameliorates circulatory (7 results) D ['11705906'], Plasmodium berghei (4399 results) S ['21494565'], malaria parasite CD36 (149 results) D ['20941396'], experimental cerebral malaria (105 results) D ['20720206'], experimental cerebral malaria (105 results) S ['21494565'], brain-sequestered (6 results) S ['12761155', '12444144'], cerebral malaria (2242 results) S ['21494565'], parasite CD36 (171 results) D ['20941396'], berghei (4605 results) S ['21494565'], Murine malaria parasite CD36 (18 results) D ['16051702'], depletion ameliorates circulatory shock (6 results) D ['11705906']
JE	TRUE	6	TRUE	inhibitory phosphosite of Separase (1 results) S ['21494564'], phosphosite of Separase (1 results) S ['21494564'], separase phosphorylation (3 results) S ['19124608', '16030258'], inhibitory phosphorylation (353 results) S ['21494564'], dimorphism effect on PGCs (1 results) S ['21494564'], gender-specific discrepancy of Securin (1 results) S ['21494564'], inhibitory phosphorylation of separase (12 results) D ['16868023', '11747808'], inhibitory phosphorylation of separase (12 results) S ['21494564', '19124608', '16030258'], germ cells (16385 results) S ['21494564'], Separase deregulation (3 results) S ['21494564', '19124608'], separase (260 results) D ['20817533'], separase (260 results) S ['21494564'], PGCs of mutant (52 results) S ['21494564'], RAD51C deficiency (4 results) D ['17312021'], genome stability of murine (2148 results) S ['21494564'], separase phosphorylation act (2 results) S ['16030258'], phosphorylation of separase (54 results) S ['21494564', '19124608'], Securin (328 results) S ['21494564'], PGCs from Separase (1 results) S ['21494564'], inhibitory phosphosite (2 results) S ['21494564']
JE	TRUE	3	TRUE	taxonomic (14410 results) S ['21494563'], broad taxonomic hypotheses (124 results) D ['19723283'], taxonomic patterns (19 results) D ['19723283', '18400019'], naturalization (207 results) D ['19723283']
JE	TRUE	3	TRUE	naturalistic stimulation (10 results) D ['12860926'], Information theoretical evaluation (2 results) D ['11369048'], Novel Estimator (12 results) S ['21494562'], estimators of information (546 results) S ['21494562'], information rate (96 results) S ['21494562'], sensory receptor neuron (83415 results) S ['21494562'], prohibitive amounts of data (9 results) S ['21494562'], information transfer of naturalistic (11 results) D ['12860926'], information transfer (1582 results) S ['21494562'], continuous signals (38 results) D ['12860926'], continuous signals (38 results) S ['21494562']
JE	TRUE	5	TRUE	Emu sequences (117 results) S ['21494561'], King Island (8 results) S ['21494561'], zebra insights from mtDNA (4 results) D ['18466230'], congeneric bird species (36 results) S ['21494561'], Glu92Lys (1 results) D ['16734695'], mononucleotide repeats (197 results) D ['20569204'], modern Emu (8 results) S ['21494561'], extinct emu (11 results) S ['21494561'], King Island Emu (1 results) S ['21494561'], Island Emu (7 results) S ['21494561'], plains zebra insights (1 results) D ['18466230'], long mononucleotide (10 results) D ['20569204'], haplotypes (35943 results) S ['21494561'], long mononucleotide repeats (6 results) D ['20569204'], congeneric bird (41 results) S ['21494561'], taxa show (15221 results) S ['21494561'], mitochondrial control (500 results) S ['21494561']
JE	TRUE	8	TRUE	selective functional impairment (4 results) D ['11205890'], arterial pressure (44557 results) S ['21494558'], ACE activity during development (706 results) S ['21494558'], reduces tension development (126 results) S ['18820769'], chronic lead (726 results) S ['21494558', '21364929', '20396857'], Kininergic (14 results) D ['10604532'], rat right ventricular myocardium (4 results) S ['18820769'], lead exposure (3472 results) S ['21494558'], lead-induced (788 results) S ['21494558'], chronic lead exposure (212 results) S ['21494558', '21364929', '20396857'], Kininergic system (6 results) D ['10604532'], Catcholamine (11 results) D ['11205890'], ACE activity (2119 results) S ['21494558', '21364929']

PMC3073978	9	FALSE	9	FALSE		7	16	TRUE	9	TRUE	5	TRUE	4	TR[illegible]
PMC3073976	14	FALSE	21	FALSE		13	33	TRUE	25	TRUE	23	TRUE	17	TR[illegible]
PMC3073976	14	FALSE	21	FALSE		13	33	TRUE	25	TRUE	23	TRUE	17	TR[illegible]

JE	TRUE	4	TRUE	ADAMTS-12 mRNA (3 results) D ['19151918'], ADAMTS-12 mRNA (3 results) S ['21494557'], ADAM-TS12 (1 results) D ['11279086'], intracellular processing of ADAM-TS12 (1 results) D ['11279086'], ADAMTS-12 expression (7 results) D ['19638407', '19151918'], ADAMTS-12 expression (7 results) S ['21494557'], trophoblast (9304 results) S ['21494557'], ADAMTS-12 (10 results) D ['20208563', '19638407', '19151918'], ADAMTS-12 (10 results) S ['21494557'], multiple thrombospondin-1 repeats (15 results) D ['12562771', '11279086'], thrombospondin-1 repeats (6 results) D ['12562771', '11279086'], ADAMTS subtypes (3 results) S ['21494557', '12855604'], extravillous (1031 results) S ['21494557'], processing of ADAM-TS12 (2 results) D ['11279086'], ADAMTS (812 results) S ['21494557'], ADAMTS-12 mRNA levels (2 results) D ['19151918'], ADAMTS-12 mRNA levels (2 results) S ['21494557'], human extravillous (1003 results) S ['21494557']
JE	TRUE	10	TRUE	Mild Pelizaeus-Merzbacher disease (4 results) D ['15837131'], Molecular diagnosis of Menkes (29 results) D ['18256395', '15693857'], Molecular diagnosis of Menkes (29 results) S ['19501626'], splice site mutations (640 results) S ['21494555'], bioinformatics tool (137 results) S ['21494555'], splice site (5912 results) S ['21494555'], Menkes disease (553 results) S ['21494555'], Menkes (1960 results) S ['21494555'], wild-type BTK transcripts (1 results) D ['12405164'], BTK splice-site (17 results) D ['12405164'], site mutations (1590 results) S ['21494555'], different classical Menkes disease (4 results) D ['7977350'], different classical Menkes disease (4 results) S ['10739752', '8981948'], abolishes Golgi localization (13 results) D ['9467005'], different classical Menkes (4 results) D ['7977350'], different classical Menkes (4 results) S ['10739752', '8981948'], ATP7A gene mutations (3 results) D ['11241493'], ATP7A gene (58 results) S ['21494555', '19888294', '19501626'], occipital horn syndrome (48 results) D ['11241493'], occipital horn syndrome (48 results) S ['21494555', '10739752'], ATP7A (432 results) S ['21494555'], ATP7A gene lead (11 results) S ['10739752'], gene abolishes Golgi localization (4 results) D ['9467005'], occipital horn (141 results) S ['21494555'], BTK splice-site mutations (16 results) D ['12405164'], Menkes genotype-phenotype (6 results) S ['19501626'], patients with Menkes (443 results) S ['21494555', '19888294'], classical Menkes (39 results) S ['21494555', '10739752'], ATP7A gene abolishes (2 results) D ['9467005'], ATP7A gene abolishes Golgi (1 results) D ['9467005'], classical Menkes disease (20 results) D ['10319589', '7977350'], classical Menkes disease (20 results) S ['10739752'], occipital (26808 results) S ['21494555'], horn syndrome (64 results) S ['21494555'], Menkes genotype-phenotype correlation (9 results) S ['19501626']
JE	TRUE	10	TRUE	Mild Pelizaeus-Merzbacher disease (4 results) D ['15837131'], Molecular diagnosis of Menkes (29 results) D ['18256395', '15693857'], Molecular diagnosis of Menkes (29 results) S ['19501626'], splice site mutations (640 results) S ['21494555'], bioinformatics tool (137 results) S ['21494555'], splice site (5912 results) S ['21494555'], Menkes disease (553 results) S ['21494555'], Menkes (1960 results) S ['21494555'], wild-type BTK transcripts (1 results) D ['12405164'], BTK splice-site (17 results) D ['12405164'], site mutations (1590 results) S ['21494555'], different classical Menkes disease (4 results) D ['7977350'], different classical Menkes disease (4 results) S ['10739752', '8981948'], abolishes Golgi localization (13 results) D ['9467005'], different classical Menkes (4 results) D ['7977350'], different classical Menkes (4 results) S ['10739752', '8981948'], ATP7A gene mutations (3 results) D ['11241493'], ATP7A gene (58 results) S ['21494555', '19888294', '19501626'], occipital horn syndrome (48 results) D ['11241493'], occipital horn syndrome (48 results) S ['21494555', '10739752'], ATP7A (432 results) S ['21494555'], ATP7A gene lead (11 results) S ['10739752'], gene abolishes Golgi localization (4 results) D ['9467005'], occipital horn (141 results) S ['21494555'], BTK splice-site mutations (16 results) D ['12405164'], Menkes genotype-phenotype (6 results) S ['19501626'], patients with Menkes (443 results) S ['21494555', '19888294'], classical Menkes (39 results) S ['21494555', '10739752'], ATP7A gene abolishes (2 results) D ['9467005'], ATP7A gene abolishes Golgi (1 results) D ['9467005'], classical Menkes disease (20 results) D ['10319589', '7977350'], classical Menkes disease (20 results) S ['10739752'], occipital (26808 results) S ['21494555'], horn syndrome (64 results) S ['21494555'], Menkes genotype-phenotype correlation (9 results) S ['19501626']

PMC3073975	10	FALSE	8	FALSE		5	15	TRUE	14	TRUE	13	TRUE	12	TRU
PMC3073972	10	FALSE	4	FALSE		6	14	TRUE	12	TRUE	8	TRUE	6	TRU
PMC3073971	5	FALSE	5	FALSE		6	9	TRUE	6	TRUE	5	TRUE	3	TRU
PMC3073970	8	FALSE	14	FALSE		6	21	TRUE	18	TRUE	15	TRUE	13	TRU

JE	TRUE	11	TRUE	IFN-beta (4473 results) S ['21494554'], model defines Tip-DCs (1 results) D ['21179567'], cellular source of interferon (1558 results) D ['21179567'], defines Tip-DCs (1 results) D ['21179567'], Novel reporter mouse (4 results) S ['19667093'], interferon-producing (266 results) D ['19325882'], reporter model defines (26 results) D ['21179567'], Listeria monocytogenes (12214 results) S ['21494554'], reporter model defines Tip-DCs (1 results) D ['21179567'], fluorescence reporter model defines (6 results) D ['21179567'], monocytogenes infection (771 results) D ['21179567'], monocytogenes infection (771 results) S ['21494554'], Review Listeria monocytogenes (945 results) D ['21178482'], reporter mouse reveals constitutive (18 results) S ['19667093'], Listeria (14497 results) S ['21494554'], fluorescence reporter model (574 results) D ['21179567'], monocytogenes (12430 results) S ['21494554'], mouse reveals constitutive (153 results) S ['19667093']
JE	TRUE	3	TRUE	Knockdown of aminopeptidase-N (9 results) D ['17213205'], triatomine bug Rhodnius (13 results) D ['16935217'], dsRNA (4559 results) S ['21494551'], gland nitrophorin (15 results) D ['16935217'], triatomine bug Rhodnius prolixus (10 results) D ['16935217'], species-specific insecticides (2 results) D ['19815067'], larvae (38478 results) S ['21494551'], salivary gland nitrophorin (27 results) D ['16935217'], dsRNA ingestion (3 results) D ['16935217'], Mamestra configurata characterization (9 results) D ['12701112'], larval (25839 results) S ['21494551'], configurata characterization (9 results) D ['12701112'], RNAi for pest (38 results) D ['19837076'], RNAi for pest (38 results) S ['21494551']
JE	TRUE	3	TRUE	IL-33 enhances (11 results) D ['19906013', '19553541', '18955562'], IL-33 enhances (11 results) S ['21494550'], monoclonal Abs for IL-33 (1 results) S ['21494550'], soluble ST2-Fc fusion (2 results) D ['15210768'], soluble ST2-Fc fusion (2 results) S ['21494550'], lipopolysaccharide receptor (140 results) D ['19553541'], lipopolysaccharide receptor complex (13 results) D ['19553541'], IL-33 (227 results) S ['21494550'], pathophysiological of IL-33 (2 results) D ['19506243'], cytokine production (18074 results) S ['21494550']
JE	TRUE	8	TRUE	Chronic hexosamine flux stimulates (1 results) D ['17227772'], differential effects on glucosamine (109 results) D ['15199059'], glucosamine on hexosamine (12074 results) S ['21494549'], O-GlcNAcylation (182 results) S ['21494549'], O-linked (3537 results) S ['21494549'], Glucosamine cardioprotection (12 results) S ['17208994', '16337959'], Chronic hexosamine flux (16 results) D ['17227772'], hexosamine (1959 results) S ['21494549'], hexosamine flux stimulates (8 results) D ['17227772'], hexosamine flux stimulates (8 results) S ['21494549'], N-acetylglucosamine protein modification (4 results) S ['17208994'], flux stimulates (549 results) S ['21494549'], hexosamine flux stimulates fatty (2 results) D ['17227772'], hexosamine flux stimulates fatty (2 results) S ['21494549'], hexosamine biosynthesis (135 results) S ['21494549'], Glucosamine (16024 results) S ['21494549'], flux stimulates fatty (52 results) D ['17227772'], flux stimulates fatty (52 results) S ['21494549'], N-acetylglucosamine protein (8 results) S ['17208994'], Dynamic actions of glucose (32 results) D ['18445751', '15199059'], flux stimulates fatty acid (93 results) D ['17227772'], flux stimulates fatty acid (93 results) S ['21494549']

PMC3073969	9	FALSE	16	FALSE		9	23	TRUE	17	TRUE	15	TRUE	12	TRU
PMC3073968	2	FALSE	4	FALSE		3	5	TRUE	3	TRUE	3	TRUE	2	TRU
PMC3073967	4	FALSE	11	FALSE		4	13	TRUE	9	TRUE	8	TRUE	8	TRU
PMC3073951	7	FALSE	16	FALSE		6	23	TRUE	20	TRUE	17	TRUE	16	TRU

JE	TRUE	6	TRUE	human gene SLCO1B1 (110 results) S ['20660695'], phase of Category-1 (29 results) D ['18160750'], SLCO1B1 polymorphisms on rifampin (5 results) S ['20660695'], concentrations of NALC-NaOH (5 results) D ['14596969'], concentrations of NALC-NaOH (5 results) S ['19919781'], Moxifloxacin versus ethambutol (7 results) D ['19345831'], Moxifloxacin versus ethambutol (7 results) S ['19406981', '16675781'], protocol for TBTC (1 results) S ['21494548'], polymorphisms on rifampin (52 results) S ['20660695'], human gene SLCO1B1 polymorphisms (63 results) S ['20660695'], gene SLCO1B1 polymorphisms (70 results) S ['20660695'], African patients (900 results) S ['21494548'], culture conversion (121 results) S ['21494548'], re-emergent Mycobacterium (4 results) D ['14598967'], re-emergent Mycobacterium tuberculosis (3 results) D ['14598967'], versus ethambutol (86 results) D ['19345831'], versus ethambutol (86 results) S ['19406981'], intensive phase of Category-1 (4 results) D ['18160750'], conversion time among patients (6925 results) S ['21494548'], Study protocol-correct (12 results) S ['21494548'], sputum (25372 results) S ['21494548'], NALC-NaOH for decontamination (16 results) D ['14596969'], NALC-NaOH for decontamination (16 results) S ['19919781'], isoniazid during intensive (206 results) S ['19406981'], era of re-emergent (1 results) D ['14598967']
JE	TRUE	1	TRUE	matter inhibits alveolar fluid (1 results) S ['16439801'], pollution particles causes mild (26 results) D ['19654918'], particulate matter inhibits alveolar (17 results) S ['16439801'], particulate matter (9691 results) S ['21494547'], matter inhibits alveolar (4 results) S ['16439801'], particles causes mild cardiopulmonary (5 results) D ['19654918']
JE	TRUE	8	TRUE	callosum (12620 results) S ['21494606'], parasagittal corpora callosa (1 results) D ['17481923'], tensor (9539 results) S ['21494606'], corpus callosum (12598 results) S ['21494606'], comparison with slicewise (1 results) S ['20574966'], whole-brain evaluation of three-dimensional (112 results) S ['20574966'], splenium (1022 results) S ['21494606'], leptin (19481 results) S ['21494606'], Tractography-guided (4 results) D ['17481923'], slicewise correction (2 results) S ['20574966'], affine whole-brain (8 results) S ['20574966'], affine whole-brain evaluation (5 results) S ['20574966'], diffusion tensor (4587 results) S ['21494606'], gender difference of fiber (407 results) D ['20435024'], parasagittal corpora (3 results) D ['17481923']
JE	TRUE	11	TRUE	neural stem cell adhesion (4 results) S ['19053638'], polypyrrole (884 results) S ['21494605'], PPy counter ion (5 results) S ['21494605'], polypyrrole biomaterials (136 results) D ['20056273'], neural stem (4855 results) S ['21494605'], NSC (5699 results) S ['21494605'], gel layer (582 results) S ['21494605'], biocompatibility (10377 results) S ['21494605'], implications of polypyrrole (6 results) D ['18765899'], electronic polymer surface switch (6 results) S ['19053638'], electromechanical properties (170 results) D ['20056273'], polymer reduction (6 results) S ['21494605'], NSC culture (16 results) S ['21494605'], Biocompatibility implications of polypyrrole (2 results) D ['18765899'], basement membrane matrix (321 results) S ['21494605'], PPy substrates (41 results) D ['18765899'], PPy substrates (41 results) S ['21494605'], PPy (717 results) S ['21494605'], neural stem cell (1415 results) S ['21494605'], biocompatibility of polypyrrole (29 results) D ['19643473', '18765899', '15621241'], biocompatibility of polypyrrole (29 results) S ['21494605'], polypyrrole implants (25 results) D ['18765899', '15621241'], membrane matrix (637 results) S ['21494605']

PMC3073946	14	FALSE	6	FALSE		16	19	TRUE	12	TRUE	9	TRUE	6	TRU
PMC3073945	18	FALSE	9	FALSE		11	23	TRUE	15	TRUE	10	TRUE	10	TRU
PMC3073944	1	FALSE	11	FALSE		3	5	FALSE	5	FALSE	5	TRUE	4	TRU
PMC3073943	17	FALSE	10	FALSE		5	19	TRUE	15	TRUE	12	TRUE	12	TRU

JE	TRUE	2	TRUE	Widespread requirement for Hedgehog (2 results) D ['14520411'], Smoothened-independent mechanisms (2 results) D ['19136624'], Gli1 contributes (27 results) D ['18410405'], GLI1 (668 results) S ['21494603'], REG4 (54 results) D ['20349522', '20183800', '19900450', '19834624', '19789838', '19716164', '19546835'], Smoothened-independent (4 results) D ['19136624'], marker REG4 (30 results) D ['20349522', '20183800', '19789838', '19716164', '19546835', '19143768', '17260007'], tumor marker REG4 (29 results) D ['20349522', '20183800', '19789838', '19716164', '19546835', '19143768', '17260007', '17237819'], RegIV gene promoter (1 results) S ['21494603'], Hedgehog ligand stimulation (18 results) D ['14520411'], RegIV gene (3 results) S ['21494603'], mediates PDAC cell (8 results) D ['19136624'], RegIV (14 results) D ['20349522', '15059891'], RegIV (14 results) S ['21494603'], gene promoter (10853 results) S ['21494603'], Novel tumor marker REG4 (10 results) D ['19789838', '17237819', '16918991'], mediates PDAC (8 results) D ['19136624'], mediates PDAC cell survival (2 results) D ['19136624'], RegIV expression (4 results) D ['20349522', '15059891'], RegIV expression (4 results) S ['21494603']
JE	TRUE	5	TRUE	polymerase gene RPOTm (10 results) D ['20231244'], GTPase (15837 results) S ['21494602'], Review Unequal genetic redundancies (1 results) D ['16949326'], RNA polymerase gene RPOTm (10 results) D ['20231244'], mitochondrial phage-type RNA (33 results) D ['20231244'], mitochondrial phage-type (33 results) D ['20231244'], Arabidopsis Miro GTPase (3 results) D ['20931334', '18344283'], Arabidopsis Miro GTPase (3 results) S ['21494602'], Arabidopsis RPL21M protein (1 results) D ['16698901'], lifespan of adjacent (51 results) D ['21135240'], pollen tube growth (556 results) S ['21494602'], mitochondrial phage-type RNA polymerase (32 results) D ['20231244'], FUSION DEFECTIVE1 encodes (4 results) D ['16698901'], MIRO1 influences (2 results) D ['20931334', '18344283'], miro2-2 (1 results) S ['21494602'], mitochondrial morphology (793 results) S ['21494602'], Miro GTPase (5 results) D ['19098100', '18344283', '15479738'], Miro GTPase (5 results) S ['21494602'], Arabidopsis Miro (3 results) D ['20931334', '18344283'], Arabidopsis Miro (3 results) S ['21494602'], phage-type RNA polymerase gene (45 results) D ['20231244'], MIRO1 (6 results) D ['20931334', '19103291', '18344283'], MIRO1 (6 results) S ['21494602'], embryogenesis (20209 results) S ['21494602'], gene RPOTm (10 results) D ['20231244'], windows flexible strategies (6 results) D ['9396791'], influences mitochondrial morphology (363 results) D ['20931334']
JE	TRUE	4	TRUE	albicans (26474 results) S ['21494601'], Candida albicans thiamine (1 results) S ['18652651'], periplasmic (8080 results) S ['21494601'], characterization of CA1462 (1 results) S ['18652651'], albicans thiamine pyrophosphokinase (1 results) S ['18652651'], albicans thiamine (13 results) S ['18652651'], Candida albicans thiamine pyrophosphokinase (1 results) S ['18652651'], CA3427 (1 results) S ['21494601'], Structural characterization of CA1462 (1 results) S ['18652651'], Candida albicans (24867 results) S ['21494601'], CA3427 protein (1 results) S ['21494601'], microdilution method for echinocandin (101 results) D ['20335424']
JE	TRUE	7	TRUE	Nicotiana alata reveals differential (1 results) D ['19925809'], proteinase (35046 results) S ['21494600'], proteinase inhibitor (4722 results) S ['21494600'], alata reveals differential stabilization (1 results) D ['19925809'], proteinase inhibitor from Nicotiana (265 results) D ['19925809'], PI-II domains (7 results) D ['7903168'], PI-II domains (7 results) S ['21494600'], inhibitor-1 from Russet (1 results) D ['2494344'], alata reveals (4 results) D ['19925809'], Burbank potato tubers (3 results) D ['2494344'], alata reveals differential (1 results) D ['19925809'], Nicotiana alata reveals (4 results) D ['19925809'], disulphide bonds (1045 results) S ['21494600'], cysteine residues (8767 results) S ['21494600'], reveals differential stabilization (33 results) D ['19925809'], Russet Burbank potato tubers (3 results) D ['2494344'], disulphide (5222 results) S ['21494600'], Streptomyces griseus proteinase (22 results) D ['2494344'], griseus proteinase (23 results) D ['2494344'], reaction centre (503 results) S ['21494600'], Pi7C (1 results) S ['21494600'], PI-II (48 results) D ['15821877'], PI-II (48 results) S ['21494600'], chymotrypsin inhibitor-1 from Russet (1 results) D ['2494344'], Pi6C (1 results) S ['21494600'], chymotrypsin inhibitor-1 (8 results) D ['2494344'], polypeptide chymotrypsin inhibitor-1 (1 results) D ['2494344']

PMC3073938	11	FALSE	4	FALSE		4	11	TRUE	10	TRUE	7	TRUE	4	TRU
PMC3073934	2	FALSE	6	FALSE		3	7	TRUE	6	TRUE	6	TRUE	4	TRU
PMC3073933	4	FALSE	4	FALSE		4	8	TRUE	6	TRUE	6	TRUE	4	TRU
PMC3073932	6	FALSE	8	FALSE		11	14	TRUE	8	TRUE	8	TRUE	8	TRU
PMC3073931	7	FALSE	13	FALSE		9	16	TRUE	12	TRUE	11	TRUE	10	TRU
PMC3073930	8	FALSE	8	FALSE		9	14	TRUE	9	TRUE	7	TRUE	7	TRU

JE	TRUE	4	TRUE	Probiotic Lactobacillus reuteri suppress (7 results) D ['18425802'], reuteri suppress (10 results) D ['18425802'], mucosal sites predicts (18 results) D ['19079189'], Lactobacillus reuteri suppress (10 results) D ['18425802'], reconstitution (21856 results) S ['21494598'], sites predicts AIDS (13 results) D ['19079189'], Lactobacillus reuteri suppress proinflammatory (1 results) D ['18425802'], levels of bacterial (56226 results) S ['21494598'], Microbial translocation (123 results) S ['21494598'], reuteri suppress proinflammatory (1 results) D ['18425802'], mucosal sites predicts AIDS (1 results) D ['19079189'], reuteri suppress proinflammatory cytokines (1 results) D ['18425802'], HIV fallout (11 results) D ['16482171'], Plasma levels of bacterial (3556 results) S ['21494598'], Review HIV fallout (2 results) D ['16482171']
JE	TRUE	3	TRUE	dual-attention theory (1 results) D ['19028591'], Neural correlates of relational (22 results) S ['15689557'], episodic insights from fMRI (20 results) D ['19028591'], main DMN regions (5 results) S ['21494597'], DMN regions (16 results) S ['21494597'], default mode (499 results) S ['21494597'], DMN (1275 results) S ['21494597'], memory retrieval (1571 results) S ['21494597']
JE	TRUE	4	TRUE	plasma membrane reductase activity (2 results) D ['7965925'], eukaryotic trends for neuroscience (5 results) D ['7512770'], Paramecium tetraurelia (590 results) S ['21494596'], glutamate chemosensory signal (12 results) D ['9372445'], Paramecium (2955 results) S ['21494596'], tetraurelia (628 results) S ['21494596'], repelling (404 results) S ['21494596'], glutamate chemosensory signal transduction (16 results) D ['9372445']
JE	TRUE	4	TRUE	ECF sigma factor (99 results) D ['20154128', '19737356', '19218445'], ECF sigma factor (99 results) S ['21494595'], PhyR stress response (7 results) D ['20735776', '20154128', '19218445'], oxygen network with species-specific (2 results) S ['15911751'], general starvation-stress genes (4 results) S ['21494595'], PhyR stress (7 results) D ['20735776', '20154128', '19218445'], response of swarmer (41 results) D ['19948804', '11972783'], response of swarmer (41 results) S ['21494595', '18723629', '12237413'], crescentus (959 results) S ['21494595'], Caulobacter (1159 results) S ['21494595'], sigma factor (5111 results) S ['21494595'], Caulobacter crescentus (950 results) S ['21494595'], PhyR stress response regulator (5 results) D ['20735776', '19218445'], COG new developments (4 results) D ['11125040']
JE	TRUE	5	TRUE	Contrast-dependence of surround (8 results) D ['20079853', '11226503'], Center-surround visual motion (64 results) S ['20505209'], perceptual centre-surround (5 results) D ['12867982'], perceptual centre-surround (5 results) S ['21494594'], perceptual centre-surround suppression (1 results) S ['21494594'], surround suppression (105 results) S ['21494594'], excitatory visual processes (239 results) S ['21494594'], perceptual surround suppression (52 results) S ['21494594'], Macaque experimental testing (170 results) D ['20079853'], interictal cortical hyperexcitability (3 results) D ['16162255', '11940694'], suppression incorporate excitatory (1 results) S ['21494594'], perceptual surround (209 results) S ['21494594'], incorporate excitatory feedback (2 results) S ['21494594'], Center-surround visual motion processing (21 results) D ['17079669', '15694323'], Center-surround visual motion processing (21 results) S ['20505209'], Contrast-dependence (69 results) D ['20079853'], suppression incorporate excitatory feedback (1 results) S ['21494594'], Center-surround visual (248 results) S ['20505209'], neuronal surround suppression incorporate (1 results) S ['21494594'], recurrent network model (31 results) D ['20079853']
JE	TRUE	6	TRUE	dioxin-responsive enhancer (38 results) D ['8384216', '1313023'], alphaB-crystallin (735 results) S ['21494593'], enhancer (32184 results) S ['21494593'], hydrocarbon receptor sans endogenous (1 results) D ['17535977'], hydrocarbon (27458 results) S ['21494593'], dioxin-independent gene (3 results) D ['19759094', '16214954'], aryl hydrocarbon (13189 results) S ['21494593'], hydrocarbon receptor (3087 results) S ['21494593'], aryl hydrocarbon receptor (2962 results) S ['21494593'], Orientation-dependent influence (2 results) S ['12403771'], novel induction mechanism (1 results) D ['15144902'], intergenic enhancer (9 results) S ['12403771'], rat CYP1A2 gene (3 results) D ['15144902'], post-Golgi compartment of frog (6 results) D ['8207008'], dioxin-independent (6 results) D ['19759094', '16214954'], receptor-Arnt heterodimer (3 results) D ['15144902']

PMC3073929	4	FALSE	11	FALSE		10	15	TRUE	11	TRUE	7	TRUE	6	TRU
PMC3073928	10	FALSE	3	FALSE		5	11	TRUE	9	TRUE	7	TRUE	6	TRU
PMC3057017	8	FALSE	13	FALSE		18	20	TRUE	18	TRUE	18	TRUE	18	TRU
PMC3060345	0	TRUE	0	TRUE		0	0	FALSE	0	FALSE	0	FALSE	0	FAL
PMC3072770	0	TRUE	19	FALSE		2	19	TRUE	17	TRUE	17	TRUE	15	TRU

JE	TRUE	3	TRUE	phosphodiesterase (28867 results) S ['21494592'], Phosphodiesterase upregulation (885 results) S ['21494592'], PDE10A expression (27 results) D ['17400452'], MCT PASMCs (6 results) S ['21494592', '20530035'], phosphodiesterase inhibitor tolafentrine (10 results) S ['16262900', '15031263'], PDE10A (83 results) S ['21494592'], dual-selective phosphodiesterase inhibitor tolafentrine (5 results) S ['15031263'], MCT-PH (8 results) S ['21494592'], PASMCs (335 results) S ['21494592'], MCT-PH rats (3 results) S ['21494592'], tandem GAF domain (5 results) D ['16330539'], dual-selective phosphodiesterase inhibitor (12 results) S ['15031263'], medial strips of pig (29 results) D ['8004394'], pulmonary role for PDE1 (10 results) D ['16980375', '15377497'], pulmonary role for PDE1 (10 results) S ['17438150']
JE	TRUE	2	TRUE	inhibits B16F10 melanoma metastasis (40 results) D ['19523924'], Ligustrazine inhibits B16F10 melanoma (1 results) D ['19523924'], CD34-reactive (14 results) D ['18976402'], inhibits B16F10 melanoma (93 results) D ['19523924'], cell-resistant tumors (2 results) D ['12566422'], CD34 (21401 results) S ['21494591'], Ligustrazine inhibits B16F10 (1 results) D ['19523924'], hematopoietic component for polyp (2 results) S ['18077429'], inhibits B16F10 (95 results) D ['19523924'], Ligustrazine inhibits (24 results) D ['19523924'], B16F10 melanoma metastasis (6 results) D ['19523924'], vascular permeability (7912 results) S ['21494591'], STAT6-dependent process (8 results) D ['12566422']
JE	TRUE	14	TRUE	NR5A1 (422 results) S ['21078366'], steroidogenic factor-1 genes (675 results) S ['20887963'], hyperandrogenic hirsute women (2 results) D ['11297612'], steroidogenic factor causes (1385 results) D ['20660055'], steroidogenic factor causes (1385 results) S ['20887963'], Steroidogenic factor-1 gene mutation (249 results) D ['20302644', '20203099', '20080844', '19616058', '19318730'], Steroidogenic factor-1 gene mutation (249 results) S ['20887963', '20595937'], Jameson Gonadal (54 results) S ['17200175', '16684822'], factor NR5A1 (391 results) D ['20660055'], factor NR5A1 (391 results) S ['21078366', '20887963', '20595937'], Achermann McElreavey (3 results) S ['20887963', '19246354'], Mutational analysis of steroidogenic (48 results) D ['20302644', '17656604', '17431898'], Mutational analysis of steroidogenic (48 results) S ['19439508', '17940071'], hyperandrogenic hirsute (61 results) D ['11297612'], human steroidogenic factor causes (747 results) D ['20660055'], human steroidogenic factor causes (747 results) S ['20887963', '20595937'], Zenaty Lin (1 results) S ['17940071'], steroidogenic factor NR5A1 (385 results) D ['20660055'], steroidogenic factor NR5A1 (385 results) S ['21078366', '20887963', '20595937'], primary adrenal (728 results) S ['21078366'], normal adrenal function (126 results) S ['18987494', '17940071']
.SE	FALSE	0	FALSE	searches
JE	TRUE	9	TRUE	demands on pathway (444 results) S ['21478484'], RGD Team (6 results) S ['21478484', '18996890'], various pathway resources (1251 results) S ['21478484'], pathway data (79 results) S ['21478484'], Rat Genome Database variation (19 results) S ['18996890'], Genome Database (917 results) S ['21478484'], comprehensive pathway data sets (66 results) S ['21478484'], Dwinell (80 results) S ['21478484', '18996890'], Laulederkind (19 results) S ['18996890'], Shimoyama (1776 results) S ['21478484'], more comprehensive pathway data (251 results) S ['21478484'], pathway diagrams (35 results) S ['18996890'], comprehensive pathway data (1127 results) S ['21478484'], Pathway Portal resources (2 results) S ['21478484'], Rat Genome (674 results) S ['21478484'], RGD (5132 results) S ['21478484'], Rat Genome Database (24 results) S ['21478484', '18996890'], pathway data manipulation (393 results) S ['21478484'], Twigger (54 results) S ['21478484', '18996890']

PMC3073004	17	FALSE	10	FALSE		12	26	TRUE	23	TRUE	17	TRUE	14	TRU
PMC3073003	9	FALSE	6	FALSE		6	10	TRUE	8	TRUE	6	TRUE	5	TRU
PMC3073001	1	FALSE	5	FALSE		2	6	FALSE	6	FALSE	6	FALSE	6	TRU
PMC3073000	6	FALSE	13	FALSE		7	18	TRUE	16	TRUE	14	TRUE	11	TRU
PMC3072999	8	FALSE	10	FALSE		10	17	TRUE	15	TRUE	14	TRUE	12	TRU

JE	TRUE	8	TRUE	Separate mechanical processes underlie (3 results) D ['12611913'], Medial olivocochlear efferent reflex (1 results) D ['12799992'], medial efferent feedback (57 results) D ['19118109'], slow MOC effects (7 results) D ['19232534', '17086072'], slow MOC effects (7 results) S ['21494578'], cholinergic slow effect (534 results) D ['19232534'], SOAE level (8 results) D ['6520309'], SOAE level (8 results) S ['21494578', '19798532'], otoacoustic emission measurement issues (4 results) D ['16568366', '12799992'], cochlear (25803 results) S ['21494578'], guinea action of gentamicin (84 results) D ['19232534'], olivocochlear efferent (149 results) S ['21494578'], otoacoustic emissions (3634 results) S ['21494578'], efferent stimulation (121 results) D ['19232534', '17344378'], olivocochlear (730 results) S ['21494578'], Assoc Res Otolaryngol (13 results) D ['16568366'], half-octave offset (3 results) D ['19118109'], efferent (20066 results) S ['21494578'], Res Otolaryngol (16 results) D ['16568366'], slow MOC (11 results) D ['19232534', '17086072'], slow MOC (11 results) S ['21494578'], slow effect of efferent (94 results) D ['19232534', '17344378'], awake guinea action (52 results) D ['9325351'], otoacoustic (3851 results) S ['21494578'], Assoc Res (44 results) D ['16568366'], medial olivocochlear efferent (58 results) S ['21494578'], novel cholinergic slow effect (10 results) D ['19232534', '17344378', '7751937']
JE	TRUE	3	TRUE	heart myofilaments (7 results) D ['2973419'], Woodbury (1012 results) S ['21494577'], immobilization onto immunoanalytical platforms (1 results) D ['18023402'], enzyme modulators (31 results) S ['20408521'], onto immunoanalytical (1 results) D ['18023402'], Gonzalez-Moa (18 results) D ['19305911'], antibody immobilization onto immunoanalytical (1 results) D ['18023402'], onto immunoanalytical platforms (1 results) D ['18023402'], peptide space (7 results) S ['20408521'], immobilization onto immunoanalytical (1 results) D ['18023402'], High-Affinity Capture Agents (3 results) D ['12670539'], enzymes on surfaces (9460 results) S ['21494577'], immunoanalytical platforms (2 results) D ['18023402'], immobilization (33272 results) S ['21494577'], protein orientation (96 results) S ['21494577']
JE	TRUE	5	TRUE	sympathetic nerve (10084 results) S ['21494635'], nerve activity during apnea (565 results) S ['21494635'], sympathetic nerve activity (4453 results) S ['21494635'], young healthy effect modification (81 results) D ['19672410'], nerve activity (7161 results) S ['21494635'], airway inflammation (7662 results) S ['21494635']
JE	TRUE	10	TRUE	MAMEF-based (1 results) D ['20622988'], synthetic oriC (8 results) S ['21494634'], enterica serovars (269 results) S ['21494634'], detection of Salmonella (3369 results) S ['21494634'], Metal-Enhanced (156 results) S ['21494634'], Paratyphi (2027 results) S ['21494634'], clinical enteric fever (1660 results) S ['21494634'], PCR of non-typhoidal (12 results) S ['20231882'], Salmonella enterica (6332 results) S ['21494634'], virulent serovars Choleraesuis (4 results) D ['18923008'], Salmonella enterica serovars Typhi (124 results) D ['20492644'], Salmonella enterica serovars Typhi (124 results) S ['21494634'], enterica (6425 results) S ['21494634'], serovars Choleraesuis (11 results) D ['18923008'], prognosis of non-typhoid (19 results) D ['16534567'], virulent serovars (5 results) D ['18923008'], serovars (3352 results) S ['21494634'], enterica serovars Typhi (99 results) S ['21494634'], silver-island films for applications (10 results) S ['16341780']
JE	TRUE	4	TRUE	CO2 inhalation study (2 results) D ['16585453'], intermediate phenotype of panic (12 results) S ['19923875'], Review False suffocation (25 results) D ['17765379'], panic disorder (8006 results) S ['21494633'], Gen Psychiatry (49 results) D ['17765379'], Review False suffocation alarms (6 results) D ['17765379', '8466392'], childhood parental (67 results) S ['19923875', '19124689'], childhood separation from parental (466 results) S ['21494633'], separation anxiety (2162 results) S ['21494633'], ascent of advances (15 results) D ['16138108'], Pesenti-Gritti (11 results) S ['21494633', '21184587', '19124689', '18040986', '17254605'], childhood separation (52 results) D ['17765379'], childhood separation (52 results) S ['21494633', '19923875', '19124689'], Arch Gen (115 results) D ['17765379'], childhood parental loss (30 results) S ['19923875', '19124689'], perturbation of infant-mother (1 results) S ['21494633'], childhood separation anxiety (32 results) D ['17765379'], childhood separation anxiety (32 results) S ['19923875', '19124689']

PMC3072995	10	FALSE	2	FALSE		7	11	TRUE	8	TRUE	6	TRUE	5	TRU
PMC3072994	5	FALSE	22	FALSE		17	25	TRUE	22	TRUE	20	TRUE	13	TRU
PMC3072993	3	FALSE	8	FALSE		5	11	FALSE	11	TRUE	10	TRUE	9	TRU
PMC3072992	5	FALSE	6	FALSE		5	8	TRUE	7	TRUE	7	TRUE	7	TRU
PMC3072990	0	TRUE	12	FALSE		3	12	FALSE	12	TRUE	11	TRUE	11	TRU

JE	TRUE	2	TRUE	source of non-genetic (19 results) D ['12935899'], Noise-driven stem (6 results) D ['18698344'], Levy flight (50 results) D ['17960243'], progenitor population dynamics (1 results) D ['18698344'], noise generates power-law (6 results) D ['16090291'], Interdependence of behavioural (29 results) D ['21076396'], power-law (5452 results) S ['21494629'], three-dimensional population-scale (3 results) D ['16820497'], white noise generates (22 results) D ['16090291'], Noise-driven stem cell (5 results) D ['18698344'], white noise generates power-law (2 results) D ['16090291'], Chemotaxis (29067 results) S ['21494629']
JE	TRUE	6	TRUE	neuroactive (2683 results) S ['21494628'], basal DOC levels (46 results) S ['21494628'], chronic ethanol exposure attenuates (36 results) S ['15936001'], O'Buckley (19 results) S ['21494628', '20028362', '20021565', '19913195', '18096321', '12867266'], BXD recombinant (314 results) D ['19958391'], deoxycorticosterone (3440 results) S ['21494628'], elevates deoxycorticosterone (8 results) S ['15936001'], BXD (395 results) S ['21494628'], GABAergic neuroactive steroids (19 results) D ['12510009'], GABAergic neuroactive steroids (19 results) S ['20028362', '20021565', '19913195', '17290803', '12867266', '11744078'], GABAergic neuroactive (160 results) S ['21494628', '20028362', '20021565', '19913195'], basal DOC (93 results) S ['21494628'], Systemic ethanol administration (17 results) D ['15719223'], Systemic ethanol administration (17 results) S ['15936001', '12867266', '11744078'], Porcu (489 results) S ['21494628'], DOC levels (75 results) S ['21494628', '16133132'], deoxycorticosterone levels (28 results) S ['18096321', '16133132', '15936001'], administration elevates deoxycorticosterone levels (1 results) S ['15936001'], elevates deoxycorticosterone levels (3 results) S ['15936001'], Neuroactive steroid modulates (35 results) S ['12867266'], administration elevates deoxycorticosterone (3 results) S ['15936001'], Alcohol acceptance (9 results) D ['7625571', '7695038'], ethanol administration elevates (25 results) S ['19913195', '15936001', '12867266', '10684899'], Systemic ethanol administration elevates (5 results) S ['15936001', '12867266', '10684899'], neurosteroid DOC (13 results) D ['11978855'], neurosteroid DOC (13 results) S ['21494628', '11744078'], ethanol administration elevates deoxycorticosterone (1 results) S ['15936001']
JE	TRUE	7	TRUE	asthma prevalence with air (2466 results) S ['21494627'], geographical latitude (41 results) S ['21494627'], general population asthma prevalence (1284 results) S ['21494627'], prevalence of asthma (20324 results) S ['21494627'], deficiency among inner-city (84 results) D ['20236657', '17919705'], general population asthma (1974 results) S ['21494627'], geographic variability of asthma (20 results) D ['14982513'], asthma prevalence (854 results) S ['21494627'], population asthma prevalence (6236 results) S ['21494627'], geographical variability of asthma (8 results) D ['19769843'], air temperature (2248 results) S ['21494627']
JE	TRUE	5	TRUE	Galectin-3 preserves renal (1 results) D ['20962111'], Galectin-3 preserves (1 results) D ['20962111'], MCP (12413 results) S ['21494626'], Galectin-3 preserves renal tubules (1 results) D ['20962111'], Galectin-3 (1492 results) S ['21494626'], pectin (3957 results) S ['21494626'], preserves renal tubules (25 results) D ['20962111'], Angiopoietin-1 therapy enhances (30 results) S ['18480750'], citrus pectin (181 results) D ['20462856', '19061992'], citrus pectin (181 results) S ['21494626'], Angiopoietin-1 therapy enhances fibrosis (3 results) S ['18480750']
JE	TRUE	6	TRUE	Prostatitis (5307 results) S ['21494624'], irritable bowel (6925 results) S ['21494624'], seminal leukocyte concentration (9 results) S ['11098023', '10438421'], male accessory gland infection (33 results) S ['11098023'], non-inflammatory prostatitis (27 results) S ['21494624'], diagnostic categories of prostatitis (42 results) S ['21494624'], Chronic Prostatitis (1619 results) S ['21494624'], gastroenterologists for symptoms (1661 results) S ['21494624'], irritable bowel syndrome (6714 results) S ['21494624'], Prostatitis Syndrome (59 results) S ['21494624'], III questionnaire for IBS (63 results) S ['21494624'], bowel syndrome (9569 results) S ['21494624']

PMC3072989	4	FALSE	17	FALSE		7	19	TRUE	17	TRUE	15	TRUE	14	TRU
PMC3072986	1	FALSE	3	FALSE		2	4	TRUE	2	TRUE	2	TRUE	2	TRU
PMC3072985	7	FALSE	7	FALSE		10	14	TRUE	11	TRUE	10	TRUE	7	TRU
PMC3072984	9	FALSE	7	FALSE		4	10	TRUE	8	TRUE	8	TRUE	7	TRU
PMC3072983	3	FALSE	7	FALSE		3	10	TRUE	8	TRUE	7	TRUE	7	TRU
PMC3072982	4	FALSE	2	FALSE		4	6	TRUE	3	TRUE	3	TRUE	3	TRU
PMC3072981	4	FALSE	10	FALSE		6	12	TRUE	10	TRUE	8	TRUE	8	TRU

JE	TRUE	9	TRUE	metabotropic glutamate (6540 results) S ['21494623'], eukaryotic membrane proteins (69 results) S ['21494623', '20099144'], functional recombinant bovine rhodopsin (44 results) D ['10455014'], eukaryotic membrane (159 results) S ['21494623'], eukaryotic MPs (26 results) S ['21494623'], metabotropic glutamate receptor (4341 results) S ['21494623'], fly eye system (259 results) S ['21494623', '20099144'], functional eukaryotic MPs (2 results) S ['21494623'], Drosophila metabotropic (58 results) S ['21494623', '20099144'], Drosophila metabotropic glutamate (6 results) S ['12923296'], species-selective recognition of citalopram (1 results) D ['9677366'], metabotropic (7888 results) S ['21494623'], human serotonin molecular (3591 results) S ['21494623'], immunoaffinity purification of metabotropic (2 results) S ['12880777'], membrane proteins (137308 results) S ['21494623'], Drosophila metabotropic glutamate receptor (6 results) S ['12923296'], cocaine-sensitive human serotonin molecular (12 results) D ['7681602'], glutamate receptor from Drosophila (305 results) S ['21494623'], dictates species-selective recognition (1 results) D ['9677366'], glutamate receptor (12295 results) S ['21494623'], purification of metabotropic (62 results) S ['12923296', '12880777']
JE	TRUE	2	TRUE	Trade-offs between gaze (2 results) D ['18085948'], memory-intensive strategy (3 results) S ['21494620'], model visits (3692 results) S ['21494620'], trade-off (5088 results) S ['21494620']
JE	TRUE	5	TRUE	bovine myoglobin with bound-ligand (2 results) D ['10446357'], L-chain ferritin (29 results) D ['16790936', '12459904', '11679711'], myoglobin with bound-ligand (52 results) D ['10446357'], BfrB (20 results) D ['12057942'], BfrB (20 results) S ['21494619'], ferroxidase (653 results) S ['21494619'], ferro-oxidase (3 results) D ['3192527'], Mycobacterium incorporation of selenomethionine (16 results) S ['19946376'], ferritin of Escherichia (308 results) D ['20138126'], ferritin iron activity (1278 results) S ['21494619'], Mtb BfrB (3 results) S ['21494619'], Mycobacterium (73730 results) S ['21494619'], H-chain ferritins (7 results) D ['2714436', '3192527'], ferritin (17527 results) S ['21494619']
JE	TRUE	7	TRUE	immuno-inhibitor B7-H1 (1 results) D ['18490751'], dampens morbidity (16 results) D ['19380790'], Theiler's virus (423 results) S ['21494618'], infection with Theiler's (537 results) S ['21494618'], antigen-presenting (22178 results) S ['21494618'], B7-H1 regulation contributes (3 results) D ['19380790'], confers strong immune implications (1 results) D ['15758163'], B7-H1 regulation (3 results) D ['19380790'], homolog confers strong immune (1 results) D ['15758163'], encephalomyelitis (17756 results) S ['21494618'], Target-dependent B7-H1 (1 results) D ['19380790'], Theiler's (894 results) S ['21494618'], Target-dependent B7-H1 regulation contributes (1 results) D ['19380790'], microglia (13144 results) S ['21494618'], Target-dependent B7-H1 regulation (1 results) D ['19380790'], B7-H1 (324 results) S ['21494618']
JE	TRUE	6	TRUE	Sputum (25372 results) S ['21494617'], treatment outcome (465875 results) S ['21494617'], patients' bodyweight (30 results) S ['21494617'], large tuberculosis treatment trial (3 results) D ['16709935'], large tuberculosis treatment (6 results) D ['16709935'], patients' bodyweight over time (5 results) S ['21494617'], short-course chemotherapy programs (122 results) D ['19402266'], bodyweight over time (288 results) S ['21494617'], tuberculosis treatment (1358 results) S ['21494617'], bodyweight (3898 results) S ['21494617']
JE	TRUE	3	TRUE	AGER polymorphisms (2 results) D ['18575614'], promotor region polymorphism (2 results) D ['8223882'], alpha gene G-308A polymorphism (101 results) D ['20459604'], Tumor necrosis polymorphism (3412 results) S ['21494616'], association between TNF (4419 results) S ['21494616'], gene region polymorphisms (5 results) D ['20459604']
JE	TRUE	4	TRUE	oxaliplatin on ion (65 results) D ['19422847'], precede development of neuropathy (28 results) S ['19745023'], excitability precede (28 results) S ['19745023'], chemotherapeutic oxaliplatin alters (1 results) D ['11011028'], sensory nerve function (133 results) S ['19164207'], Oxaliplatin induces hyperexcitability (1 results) D ['16231011'], oxaliplatin-induced (206 results) S ['21494615'], oxaliplatin alters (8 results) D ['11011028'], Acute abnormalities (31 results) S ['19164207'], oxaliplatin (4067 results) S ['21494615'], neurotoxicity (20214 results) S ['21494615'], axonal excitability precede development (2 results) S ['19745023'], axonal excitability precede (5 results) S ['19745023'], excitability precede development (10 results) S ['19745023']

PMC3072980	9	FALSE	8	FALSE		10	17	TRUE	12	TRUE	10	TRUE	10	TRU
PMC3072979	10	FALSE	9	FALSE		12	19	FALSE	19	TRUE	16	TRUE	14	TRU
PMC3072978	4	FALSE	29	FALSE		3	24	TRUE	18	TRUE	15	TRUE	14	TRU
PMC3072977	6	FALSE	4	FALSE		6	8	TRUE	7	TRUE	7	TRUE	7	TRU

JE	TRUE	5	TRUE	Review Beyond Wnt (35 results) D ['18322270'], otic capsule functional (29 results) D ['18991062'], NTR domains of netrins (2 results) D ['10452607'], melanoma cells adjust (5 results) D ['19708915'], Beyond Wnt new functions (5 results) D ['18322270'], Frizzled-related (502 results) S ['21494614'], malignant melanoma (18871 results) S ['21494614'], Frizzled (1833 results) S ['21494614'], molecular control of otic (38 results) D ['18991062'], SFRP3 (35 results) D ['20208569', '18991062', '17433286', '17079093'], SFRP3 (35 results) S ['21494614'], addition of SFRP3 (6 results) D ['17433286', '16266997'], addition of SFRP3 (6 results) S ['21494614'], otic capsule functional role (8 results) D ['18991062'], Wnt5a (604 results) S ['21494614'], Wnt5a-dependent (6 results) S ['21494614', '19901340'], melanoma cells (11643 results) S ['21494614']
JE	TRUE	7	TRUE	structure of GAD65 (39 results) D ['12296864'], structure of GAD65 (39 results) S ['19783309', '19465164', '18184926', '17384644', '16564157'], basis of autoreactivity (56 results) S ['18184926'], autoreactive conformational (36 results) D ['10222205'], autoreactive conformational (36 results) S ['16564157'], stiff-man syndrome (378 results) D ['16027042'], decarboxylase diagnostic clues (9 results) D ['18687732'], stiff-man (385 results) D ['16970217', '16027042'], autoantibody (11362 results) S ['21494613'], identify distinct immunization profiles (6 results) D ['17325256'], specificity identify distinct immunization (32 results) D ['17325256'], molecular basis of autoreactivity (29 results) S ['18184926'], GAD65 (1232 results) S ['21494613'], GAD67 (860 results) S ['21494613'], epitope specificity identify distinct (133 results) D ['17325256'], acid decarboxylase diagnostic clues (7 results) D ['18687732'], GAD autoantibody affinity (15 results) D ['17325256'], GAD autoantibody affinity (15 results) S ['21494613'], autoantigen (5988 results) S ['21494613']
JE	TRUE	12	TRUE	novel micro-electroporation (3 results) S ['20532636'], non-dimensional primary current distribution (2 results) S ['21494612', '20532636'], micro-electroporation devices (7 results) S ['21494612', '20532636'], channel-like conduction pathway (1 results) D ['19450553'], secondary current distribution (2521 results) S ['21494612'], insulator (3402 results) S ['21494612'], channel-like conduction (10 results) D ['19450553'], micro-electroporation channel configuration (2 results) S ['21494612', '20532636'], ion channel-like conduction pathway (1 results) D ['19450553'], micro-electroporation channel (2 results) S ['21494612', '20532636'], electric field (12560 results) S ['21494612'], current distribution model (4 results) S ['21494612', '20532636'], primary current distribution model (612 results) S ['21494612'], electric field magnitudes (166 results) S ['21494612', '20532636'], insulator thickness (5 results) S ['21494612'], novel micro-electroporation channel (1 results) S ['20532636'], ion channel-like conduction (9 results) D ['19450553'], novel micro-electroporation channel configuration (1 results) S ['20532636'], electrode kinetics (36 results) S ['21494612'], non-dimensional primary (6 results) S ['21494612', '20532636'], field magnitudes (24 results) S ['21494612', '20532636'], primary current distribution (5 results) S ['21494612', '20532636'], singularity-induced (3 results) S ['21494612'], secondary current distribution model (259 results) S ['21494612'], electrode kinetics on electric (857 results) S ['21494612'], distribution model (497 results) S ['21494612'], electroporation (7345 results) S ['21494612'], micro-electroporation configuration (2 results) S ['21494612', '20532636'], kinetics on electric (11635 results) S ['21494612'], current distribution (783 results) S ['21494612'], micro-electroporation configuration with platinum (1 results) S ['21494612'], electrolyte (55987 results) S ['21494612'], micro-electroporation (13 results) S ['21494612', '20532636']
JE	TRUE	5	TRUE	type III phosphodiesterase inhibitor (30 results) D ['10434004'], Ibudilast suppresses (3 results) D ['18176632', '10434004'], phosphodiesterase (28867 results) S ['21494611'], Ibudilast suppresses TNFalpha production (1 results) D ['10434004'], Ibudilast suppresses TNFalpha (1 results) D ['10434004'], phosphodiesterase inhibitor (3450 results) S ['21494611'], Ibudilast (106 results) D ['20399770', '20200338', '19929708'], Ibudilast (106 results) S ['21494611'], III phosphodiesterase inhibitor (32 results) D ['10434004'], TNFalpha production (543 results) S ['21494611']

PMC3072973	3	FALSE	26	FALSE		4	18	TRUE	15	TRUE	11	TRUE	11	TRU
PMC3072972	16	FALSE	16	FALSE		12	29	TRUE	26	TRUE	19	TRUE	11	TRU
PMC3072971	20	FALSE	6	FALSE		12	17	TRUE	10	TRUE	6	TRUE	4	TRU

JE	TRUE	10	TRUE	plate aggregation system highlights (1 results) S ['17185609'], pluripotent (8823 results) S ['21494607'], novel V-96 plate aggregation (1 results) S ['17185609'], embryonic stem cells (13148 results) S ['21494607'], interline variability (3 results) S ['21494607', '17185609'], embryoid body homogeneity (9 results) D ['18767184'], embryoid body homogeneity (9 results) S ['17185609'], pluripotent stem (4990 results) S ['21494607'], system highlights interline variability (1 results) S ['17185609'], pluripotent stem cells (4515 results) S ['21494607'], V-96 plate (2 results) S ['17185609'], highlights interline (1 results) S ['17185609'], system highlights interline (1 results) S ['17185609'], aggregation system highlights interline (1 results) S ['17185609'], cardiomyocytes (13251 results) S ['21494607'], highlights interline variability (1 results) S ['17185609'], human embryonic (10644 results) S ['21494607'], plate aggregation system (102 results) S ['17185609'], V-96 plate aggregation system (1 results) S ['17185609'], cardiac differentiation (339 results) S ['21494607'], cell embryoid body homogeneity (9 results) D ['18767184'], cell embryoid body homogeneity (9 results) S ['17185609'], novel V-96 plate (1 results) S ['17185609'], aggregation system highlights (22 results) S ['17185609'], V-96 plate aggregation (1 results) S ['17185609'], redirects differentiation from cardiogenic (2 results) D ['18096723'], novel V-96 (1 results) S ['17185609'], embryoid (1929 results) S ['21494607'], human embryonic stem (3254 results) S ['21494607']
JE	TRUE	8	TRUE	cortical granule-free (17 results) D ['12710965'], cortical granule-free (17 results) S ['21494665'], actin cap (28 results) D ['20004659'], actin cap (28 results) S ['21494665'], activation couples spindle (10 results) D ['16431375'], granule redistribution (3 results) D ['12710965'], cytokinesis during mouse (888 results) S ['21494665'], cortical granule-free domain (9 results) D ['12710965'], cortical granule-free domain (9 results) S ['21494665'], mouse oocytes (1972 results) S ['21494665'], meiotic (14224 results) S ['21494665'], cytokinesis (6185 results) S ['21494665'], meiotic maturation (1382 results) S ['21494665'], mouse oocytes requires CDC42 (2 results) D ['16782018', '16452714'], Cdc42 activation couples spindle (1 results) D ['16431375'], regulates meiotic spindle stability (6 results) D ['17276347'], granule-free domain (9 results) D ['12710965'], granule-free domain (9 results) S ['21494665'], oocytes requires CDC42 (3 results) D ['16782018', '16452714'], CK666 (1 results) S ['21494665'], oocyte meiotic maturation (190 results) S ['21494665', '20515799'], asymmetric division (471 results) S ['21494665'], actin-polymerization nucleator (29 results) D ['20237478', '18614018', '16990851'], late steps of cytokinesis (20 results) D ['16989804'], regulates oocyte polarization (17 results) D ['17276347'], regulates oocyte polarization (17 results) S ['21494665'], oocyte meiotic (4112 results) S ['21494665'], spindle migration (19 results) D ['17276347', '16989804', '16782018'], spindle migration (19 results) S ['21494665'], oocytes requires CDC42 function (3 results) D ['16782018', '16452714'], Cdc42 activation couples (19 results) D ['16431375'], oocyte polarization (9 results) S ['21494665']
JE	TRUE	2	TRUE	Stelliferin Riboside (3 results) D ['16562838', '11421753'], tetractinellid sponges (2 results) D ['15579383', '12066683'], lithistid (14 results) D ['17125225', '8087187'], lithistid (14 results) S ['21494664'], Astrophorida (6 results) D ['18583159', '15579383', '12066683'], Astrophorida (6 results) S ['21494664'], Bioactive Isomalabaricane Triterpenes (3 results) D ['16562838', '11858759'], classification of lithistid (6 results) D ['8087187'], classification of lithistid (6 results) S ['21494664'], New Guinea lithistid sponge (2 results) D ['17125225'], Triterpene Monosaccharide (1 results) D ['11421753'], biochemical characters versus morphology (1 results) D ['16325431'], Sponge Geodia globostellifera (1 results) D ['11421753'], Fijian Sponge Geodia (1 results) D ['11421753'], Astrophorida Demospongiae (6 results) D ['18583159', '15579383', '12066683'], Astrophorida Demospongiae (6 results) S ['21494664'], Guinea marine sponge Neamphius (1 results) D ['15332865'], Fijian Sponge Geodia globostellifera (1 results) D ['11421753'], support for order-level (54 results) D ['18583159'], phylogeny (96509 results) S ['21494664'], Bioactive isomalabaricane (3 results) D ['16562838', '11858759'], Norlanostane triterpenoidal saponins (1 results) D ['16124767'], new HIV-Inhibitory depsipeptide (3 results) D ['15332865'], Reassessment of homology (33 results) D ['12066683'], tetractinellid (2 results) D ['15579383', '12066683'], Demospongiae (127 results) S ['21494664']

PMC3072969	1	FALSE	4	FALSE		2	5	TRUE	4	TRUE	4	TRUE	4	TRU
PMC3072967	11	FALSE	7	FALSE		6	18	TRUE	14	TRUE	13	TRUE	9	TRU
PMC3072966	1	FALSE	18	FALSE		2	17	TRUE	15	TRUE	14	TRUE	13	TRU
PMC3072965	0	TRUE	6	FALSE		1	6	TRUE	3	TRUE	2	TRUE	2	TRU
PMC3072964	5	FALSE	4	FALSE		4	9	FALSE	9	TRUE	7	TRUE	7	TRU
PMC3072963	5	FALSE	11	FALSE		7	16	FALSE	16	TRUE	14	TRUE	12	TRU
PMC3072765	1	FALSE	7	FALSE		2	8	FALSE	8	TRUE	7	TRUE	7	TRU

JE	TRUE	3	TRUE	Chinese authors (31 results) S ['21494662'], 10-year survey of literature (376 results) S ['21494662'], Scientific publications (941 results) S ['21494662'], Foundation practice guidelines (4 results) D ['12859163'], 10-year survey (166 results) S ['21494662']
JE	TRUE	7	TRUE	free precession (879 results) S ['21494660'], suppresses growth of androgen-independent (37 results) D ['19399749'], SNR (5169 results) S ['21494660'], steady-state free precession (841 results) S ['21494660'], prostatic environment suppresses growth (7 results) D ['19399749'], Antisense-MDM2 sensitizes LNCaP (4 results) D ['17637390'], supplementation of celecoxib (17 results) D ['15126378'], mouse prostate (653 results) S ['21494660'], Antisense-MDM2 sensitizes (4 results) D ['17637390'], prostatic environment suppresses (12 results) D ['19399749'], precession (1836 results) S ['21494660'], Antisense-MDM2 sensitizes LNCaP prostate (4 results) D ['17637390'], mouse prostate imaging (573 results) S ['21494660'], histopathological characterization of prostate (23 results) D ['17187397'], prostatic environment (2 results) D ['19399749'], triterpenoid CDDO-Me inhibits (11 results) D ['19189297'], dietary supplementation of celecoxib (20 results) D ['15126378'], monitor tumour growth (1201 results) S ['21494660']
JE	TRUE	11	TRUE	Optical coherence tomography (9502 results) S ['21494659'], brain atrophy (2464 results) S ['21494659'], disease duration (7710 results) S ['21494659'], potential measure of axonal (152 results) D ['18825432'], retinal nerve fiber (1468 results) S ['21494659'], retinal nerve (1916 results) S ['21494659'], axonal damage longitudinally (13 results) S ['21494659'], retinal nerve fiber layer (1399 results) S ['21494659'], TMV with BPF (1 results) S ['21494659'], coherence tomography (7595 results) S ['21494659'], association for RNFLT (10 results) S ['21494659'], duration whereas BPF (4 results) S ['21494659'], whereas BPF (27 results) S ['21494659'], disease duration whereas BPF (3 results) S ['21494659'], within-patient inter-eye relations (1 results) S ['21494659'], nerve fiber layer (2121 results) S ['21494659'], RNFLT (77 results) S ['21494659'], BPF (381 results) S ['21494659'], fiber layer (2367 results) S ['21494659']
JE	TRUE	2	TRUE	random walker (101 results) S ['21494658'], map equation (3 results) S ['21494658'], hierarchical map (4 results) S ['21494658'], hierarchical map equation (2 results) S ['21494658'], multilevel (7895 results) S ['21494658'], hierarchical network partition (10 results) S ['21494658']
JE	TRUE	4	TRUE	PANTHER version protein (7 results) D ['17130144'], Genomic copy number determination (7472 results) S ['21494657'], melanoma cell (6311 results) S ['21494657'], CGH simulation (46 results) D ['18603568'], PANTHER version protein sequence (6 results) D ['17130144'], melanoma cell lines (3013 results) S ['21494657'], array CGH simulation (32 results) D ['18603568'], CGH (4217 results) S ['21494657'], Genome-wide loss (40 results) D ['17363583']
JE	TRUE	7	TRUE	adolescent non-suicidal self-injury (44 results) D ['19826208'], suicidal empirical tests (12 results) D ['19685959'], non-suicidal (278 results) S ['21494656'], NSSI (88 results) S ['21494656'], suicidal ideation (3164 results) S ['21494656'], suicidal ideation on suicide (2653 results) S ['21494656'], ideation (4506 results) S ['21494656'], nonsuicidal identification (15 results) D ['18991124'], suicide attempts (3998 results) S ['21494656'], non-suicidal self-injury (69 results) S ['21494656'], Non-suicidal self-injury among diagnostic (24 results) D ['19826208', '19171079'], ideation on suicide (2946 results) S ['21494656'], association of NSSI (8 results) S ['21494656'], Review Nonsuicidal (29 results) D ['20207935', '12819435'], association between NSSI (8 results) S ['21494656'], self-injury (959 results) S ['21494656']
JE	TRUE	6	TRUE	CGNs (206 results) S ['21382016'], Active caspase-8 translocates (8 results) D ['12065591'], AIF (1507 results) S ['21382016'], OGD (880 results) S ['21382016'], Reox (37 results) S ['21382016'], granule neurons (1907 results) S ['21382016'], cerebellar granule (4007 results) S ['21382016'], cerebellar granule neurons (1283 results) S ['21382016']

PMC3072764	18	FALSE	4	FALSE		15	17	TRUE	13	TRUE	9	TRUE	6	TRU
PMC3072763	0	TRUE	21	FALSE		5	20	TRUE	18	TRUE	16	TRUE	15	TRU
PMC3078894	2	FALSE	8	FALSE		3	10	FALSE	10	FALSE	10	FALSE	10	TRU

JE	TRUE	5	TRUE	Dimethylfumarate induces immunosuppression (1 results) D ['17235328'], Dimethylfumarate inhibits (18 results) D ['20482831', '19465513', '18405893', '17381463', '15649822', '11971029'], Dimethylfumarate (89 results) D ['20482831'], nuclear entry of NF-kappa (123 results) D ['19465513'], NOS2 (6498 results) S ['21382015'], Dimethylfumarate inhibits nuclear binding (5 results) D ['19465513', '17381463', '11971029'], levels of GSHs (7 results) S ['21382015'], drug dimethylfumarate (138 results) D ['20482831'], fumarate towards glutathione (1 results) D ['17049250'], methylhydrogen fumarate towards glutathione (1 results) D ['17049250'], antipsoriatic drug dimethylfumarate (19 results) D ['19465513', '11422029', '11168807'], inducer dimethylfumarate on glutathione (7 results) D ['10440245', '2123743'], inducer dimethylfumarate on glutathione (7 results) S ['21382015'], thiosuccinic acid esters (3 results) D ['17049250'], induces immunosuppression via glutathione (2 results) D ['17235328'], BG-12 (21 results) D ['19721818', '18970976'], Dimethylfumarate inhibits function (15 results) D ['20482831', '19465513', '18405893', '17381463', '15649822', '11971029'], dimethyl fumarate (184 results) S ['21382015'], methylhydrogen fumarate (1 results) D ['17049250'], oral fumarate (4 results) D ['18970976'], methylhydrogen fumarate towards (1 results) D ['17049250'], entry of NF-kappa (221 results) D ['19465513']
JE	TRUE	10	TRUE	immunoreactivity against anti-GD2 (2 results) S ['21395555'], predominant ganglioside (41 results) S ['17883393'], marker molecules of NSCs (17 results) S ['20855890', '19776077'], form of neurospheres (144 results) S ['21395555'], SSEA-1 (531 results) S ['21395555'], SSEA-1 embryonic (610 results) S ['21395555'], expression of gangliosides (1742 results) S ['21395555'], self-renewal with retention (57 results) S ['21395555', '20855890', '19776077', '19716368'], clonal aggregates (5 results) S ['21395555'], molecules of NSCs (71 results) S ['20855890'], anti-GD2 (163 results) S ['21395555'], GD2 (823 results) S ['21395555'], brains Ngamukote (1 results) S ['17883393'], antibody against GD2 (174 results) S ['21395555'], GD3 (2358 results) S ['21395555'], mouse NSCs (22 results) S ['20855890'], gangliosides (10178 results) S ['21395555'], b-series ganglioside (7 results) S ['21395555', '19776077'], anti-GD2 antibody (48 results) S ['21395555'], retention of multipotency (10 results) S ['21395555', '20855890', '19776077', '19716368'], antibody against GD3 (189 results) S ['21395555']
JE	TRUE	5	TRUE	cryoinjury (286 results) S ['21473762'], new myocardium (29 results) S ['21473762'], ventricular wall (6574 results) S ['21473762'], zebrafish heart (81 results) D ['20336145', '20336144'], zebrafish heart (81 results) S ['21473762'], Tenascin-C (1128 results) S ['21473762'], mammalian infarcts (199283 results) S ['21473762'], cryoinjury results (130 results) S ['21473762'], heart regeneration (50 results) D ['20336145', '20336144'], heart regeneration (50 results) S ['21473762']

PMC3072472	0	TRUE	29	FALSE		4	29	FALSE	29	FALSE	29	TRUE	26	[illegible]
PMC3072235	2	FALSE	17	FALSE		3	19	FALSE	19	TRUE	17	TRUE	17	[illegible]
PMC3072769	4	FALSE	25	FALSE		7	23	TRUE	20	TRUE	17	TRUE	16	[illegible]

JE	TRUE	23	TRUE	alcohol attributable fractions (43 results) S ['21474525', '20202213'], hazard rate ratios (89 results) S ['21474525'], attributable cancer (17 results) S ['21474525'], alcohol exposure information (1273 results) S ['21474525'], alcohol attributable cancer incidence (287 results) S ['21474525'], European Prospective Investigation (731 results) S ['21474525'], attributable cancer cases (1947 results) S ['21474525'], rate ratios (2608 results) S ['21474525'], upper aerodigestive (2148 results) S ['21474525'], attributable incidence of cancer (3345 results) S ['21474525'], cancer incidence (9354 results) S ['21474525'], rate ratios for alcohol (1557 results) S ['21474525'], lower alcohol attributable fractions (14 results) S ['17251244'], attributable fraction for upper (16 results) S ['21474525'], alcohol attributable cancer cases (157 results) S ['21474525', '19190158'], alcohol attributable cancer (424 results) S ['21474525'], attributable cancer incidence (3345 results) S ['21474525'], hazard rate (769 results) S ['21474525'], attributable fraction for liver (139 results) S ['21474525'], attributable fractions (384 results) S ['21474525'], attributable burden of cancer (343 results) S ['21474525'], higher alcohol attributable fraction (35 results) S ['21474525'], aerodigestive tract (2399 results) S ['21474525'], Prospective Investigation into Cancer (4559 results) S ['21474525'], aerodigestive (2766 results) S ['21474525'], overall alcohol attributable fractions (141 results) S ['21474525', '20202213'], fraction for liver (18224 results) S ['21474525'], upper aerodigestive tract (1959 results) S ['21474525'], fraction for upper (2008 results) S ['21474525']
JE	TRUE	15	TRUE	nulliparous pregnant Norwegian (9 results) D ['19369368'], factors for pre-eclampsia (4316 results) S ['21474517'], clinical risk (3354 results) S ['21474517'], multicentre prospective design (911 results) S ['21474517'], populations of nulliparous (1119 results) S ['21474517'], artery Doppler (1077 results) S ['21474517'], risk factors for pre-eclampsia (2311 results) S ['21474517'], uterine artery Doppler (393 results) S ['21474517'], uterine artery (3299 results) S ['21474517'], nulliparous pregnant Norwegian women (9 results) D ['19369368'], nulliparous women (2317 results) S ['21474517'], other nulliparous populations (288 results) S ['21474517'], risk profile for nulliparous (21 results) S ['21474517'], other nulliparous (1014 results) S ['21474517'], healthy nulliparous (415 results) S ['21474517'], nulliparous (5168 results) S ['21474517'], profile for nulliparous (89 results) S ['21474517'], study populations of nulliparous (804 results) S ['21474517'], healthy nulliparous women (108 results) S ['21474517', '19155903']
JE	TRUE	10	TRUE	Blast2GO (21 results) S ['21474551'], other metabolic reconstruction BioCyc (2 results) S ['21474551'], Pathway Tools system (520 results) S ['21474551'], PhylomeDB (5 results) S ['21474551', '20482636', '17962297'], network reconstruction (170 results) S ['21474551'], TricaCyc (1 results) S ['21474551'], aphid (2491 results) S ['21474551'], CycADS system (7 results) S ['21474551'], Pathway Tools (34 results) D ['19955237', '17965431'], Pathway Tools (34 results) S ['21474551'], pisum (3890 results) S ['21474551'], AcypiCyc database webpage (1 results) S ['21474551'], GenBank (171086 results) S ['21474551'], BioCyc databases (3 results) S ['21474551'], Pathway Tools software (17 results) D ['17965431', '12169551'], BioCyc collection of databases (9 results) D ['17965431', '16246909'], pea aphid (289 results) S ['21474551'], annotation file (7 results) S ['21474551'], BioCyc (37 results) D ['17965431'], BioCyc (37 results) S ['21474551'], Annotation Database System (523 results) S ['21474551'], AcypiCyc database (1 results) S ['21474551'], different annotation (1551 results) S ['21474551'], DromeCyc (1 results) S ['21474551'], metabolic network (965 results) S ['21474551'], AcypiCyc (1 results) S ['21474551'], CycADS (95 results) S ['21474551'], castaneum (667 results) S ['21474551'], CycADS pipeline for annotation (1 results) S ['21474551']

PMC3076504	4	FALSE	14	FALSE		3	15	TRUE	13	TRUE	13	TRUE	12	TRU
PMC3072364	19	FALSE	2	FALSE		4	16	TRUE	8	TRUE	6	TRUE	6	TRU
PMC3072363	5	FALSE	11	FALSE		7	15	TRUE	11	TRUE	7	TRUE	4	TRU
PMC3072361	5	FALSE	9	FALSE		6	12	TRUE	10	TRUE	9	TRUE	9	TRU
PMC3072358	5	FALSE	15	FALSE		10	19	TRUE	17	TRUE	16	TRUE	12	TRU

JE	TRUE	11	TRUE	glyoxylate bypass pathway (5 results) S ['21494431'], Mannich (1232 results) S ['21494431'], El-Subbagh Abu-Zaid (1 results) D ['10956199'], ICL (1341 results) S ['21494431'], isocitrate into succinate (1211 results) S ['21494431'], mannich base compounds (3 results) S ['21494431'], isocitrate lyase (940 results) S ['21494431'], Abu-Zaid Mahran (1 results) D ['10956199'], Mycobacterium tuberculosis (38586 results) S ['21494431'], isocitrate (6032 results) S ['21494431'], bypass pathway (64 results) S ['21494431'], glyoxylate bypass (107 results) S ['21494431'], catalysis isocitrate into succinate (16 results) D ['10801489'], El-Subbagh Abu-Zaid Mahran (1 results) D ['10956199'], mannich base (112 results) S ['21494431'], Mycobacterium (73730 results) S ['21494431'], glyoxylate (2251 results) S ['21494431'], lyase (17544 results) S ['21494431']
JE	TRUE	4	TRUE	Differential Interfacial Tension Hypothesis (1 results) D ['12002128'], tension governs cell (21 results) D ['19879142'], cell bond (9 results) D ['19879142'], self-rearrangement of embryonic (1 results) D ['12002128'], anteroposterior compartment (131 results) D ['19879142'], Interfacial Tension Hypothesis (1 results) D ['12002128'], tension governs (4 results) D ['19879142'], compartment boundary (115 results) D ['19879142'], compartment boundary (115 results) S ['21490725'], Drosophila anteroposterior compartment boundary (3 results) D ['19879142'], cell bond tension governs (1 results) D ['19879142'], bond tension (3 results) D ['19879142'], bond tension governs cell (1 results) D ['19879142'], tissue-organizers during growth (4 results) S ['21490725'], bond tension governs (2 results) D ['19879142'], cell bond tension (2 results) D ['19879142'], Adhesion-guided (3 results) D ['1207170'], anteroposterior compartment boundary (8 results) D ['19879142'], Drosophila anteroposterior compartment (3 results) D ['19879142'], governs cell (51 results) D ['19879142'], Drosophila anteroposterior (329 results) D ['19879142']
JE	TRUE	4	TRUE	Vibrissal kinematics (11 results) D ['18614027'], Vibrissal kinematics (11 results) S ['21490724', '19828793', '16928873'], profiles during free-air (16 results) S ['18436634'], velocity profiles during free-air (3 results) S ['18436634'], whiskers (919 results) S ['21490724'], whisker array (6 results) D ['17567777'], rats anticipate head movements (2 results) S ['16928873'], vibrissal (462 results) S ['21490724'], anticipate head movements (17 results) S ['16928873'], rats anticipate head (5 results) S ['16928873'], curvature (16912 results) S ['21490724'], whisker (1577 results) S ['21490724'], vibrissal array (6 results) D ['11466447'], vibrissal array (6 results) S ['21490724'], whisker-dependent tactile discriminations (1 results) D ['11466447'], whisker-dependent tactile (3 results) D ['11466447']
JE	TRUE	6	TRUE	surface during isochoric (9 results) D ['18946028'], MDCK cystogenesis (25 results) D ['20385777'], MDCK cystogenesis (25 results) S ['21490722', '19476639'], luminal cell death (1030 results) S ['21490722'], silico components (386 results) S ['21490722'], measures of cystogenesis (3 results) S ['21490722', '19476639'], isochoric lumen initiation (1 results) D ['18946028'], simulation cycles (3 results) S ['20236957'], MDCK (5172 results) S ['21490722'], axioms (285 results) S ['20236957'], cystogenesis (273 results) S ['21490722'], preapical patch into apical (1 results) D ['18946028'], multiple lumens (23 results) D ['9893125'], luminal cell (400 results) S ['21490722']
JE	TRUE	9	TRUE	vertebrates (30973 results) S ['21490719'], relationships between duplicability (1 results) D ['17512629'], singleton (13343 results) S ['21490719'], gene duplicability (17 results) D ['18689880', '17512629', '15454568', '14660792'], gene duplicability (17 results) S ['21490719'], tissue-selective (616 results) S ['21490719'], protein interaction network (585 results) S ['21490719'], metazoans (2215 results) S ['21490719'], protein interaction (16141 results) S ['21490719'], duplicability (33 results) D ['18689880', '17512629'], duplicability (33 results) S ['21490719', '19906700', '18675489'], singleton genes (16 results) D ['17559966'], control through microRNAs (1757 results) S ['21490719'], network properties (491 results) S ['21490719'], dosage control through microRNAs (32 results) S ['21490719'], ohnologs (17 results) D ['20439718', '17652425'], interaction network (1139 results) S ['21490719'], vertebrate-specific whole genome (7 results) S ['21490719'], vertebrate-specific whole genome duplication (4 results) S ['21490719'], singleton hubs (2 results) S ['21490719']

PMC3072375	10	FALSE	14	FALSE		9	21	TRUE	20	TRUE	17	TRUE	16	TRU
PMC3072374	8	FALSE	9	FALSE		10	16	TRUE	14	TRUE	10	TRUE	7	TRU
PMC3072368	7	FALSE	15	FALSE		11	21	TRUE	16	TRUE	13	TRUE	11	TRU
PMC3072367	1	FALSE	18	FALSE		7	17	TRUE	12	TRUE	11	TRUE	10	TRU

JE	TRUE	8	TRUE	SMN mutant neuroblasts (1 results) S ['21490958'], muscular atrophy reveals (78 results) D ['20705736'], muscular atrophy reveals (78 results) S ['19464282'], minimal SMN complex facilitates (1 results) D ['18621711'], survival motor neuron (540 results) S ['21490958'], spinal muscular atrophy reveals (44 results) D ['20705736', '17353360'], spinal muscular atrophy reveals (44 results) S ['19464282'], neuroblasts (2636 results) S ['21490958'], SMN gradient leads (1 results) S ['21490958'], highest levels of SMN (6 results) D ['16236758'], overexpression of SMN (32 results) D ['18923150'], SMN levels (40 results) D ['20705736'], SMN levels (40 results) S ['21490958'], SMN expression (50 results) D ['18791638'], SMN (1307 results) S ['21490958'], atrophy reveals (380 results) D ['20705736'], SMN gradient (5 results) S ['21490958'], spinal muscular atrophy (3473 results) S ['21490958'], melanogaster model of spinal (12 results) D ['18791638', '17353360', '12783845'], melanogaster model of spinal (12 results) S ['19464282'], survival motor (11767 results) S ['21490958'], SMN complex facilitates formation (10 results) D ['18621711'], muscular atrophy (11865 results) S ['21490958'], motor neuron (10031 results) S ['21490958']
JE	TRUE	4	TRUE	Review ThermoTRP channels (6 results) D ['12838328'], TRPV3 gene (33 results) S ['21490957'], ThermoTRP (22 results) D ['19164517', '16829128'], ThermoTRP (22 results) S ['16926268'], TRPV genes (4 results) S ['21490957'], TRPV (2493 results) S ['21490957'], cold C-terminal (402 results) S ['21490957'], camphor sensor (7 results) D ['15746429'], novel TRPV genes (18 results) S ['21490957'], channels TRPV3 (121 results) S ['21490957'], 2-APB sensitivity (1 results) D ['19164517'], Review ThermoTRP (6 results) D ['12838328'], region of TRPV3 (8 results) D ['19160498'], frog TRPV3 (14 results) D ['19164517', '19160498', '17420775', '15194687'], frog TRPV3 (14 results) S ['21490957', '16926268'], TRPV3 (125 results) S ['21490957'], cold C-terminal domain determines (4 results) D ['16672657']
JE	TRUE	3	TRUE	maintenance component interacts (91 results) D ['19854131'], yeast culture arrays (37 results) D ['17408510'], yeast culture arrays (37 results) S ['20509870'], telomere maintenance component interacts (9 results) D ['19854131', '12975323'], proliferation kinetics from time-lapse (46 results) D ['17408510'], enhancer analysis (21 results) S ['18845848'], enhancer analysis of cdc13-1 (2 results) S ['21490951', '18845848'], agar yeast culture arrays (3 results) D ['17408510'], agar yeast culture arrays (3 results) S ['20509870'], RPA-like mammalian complex binds (2 results) D ['19854130'], micro-organism growth characteristics (12 results) S ['20509870'], component interacts with STN1 (2 results) D ['19854131'], genomewide suppressor (31 results) S ['18845848'], quantification of micro-organism (7 results) S ['20509870'], telomerase (10110 results) S ['21490951'], yeast yku70Delta mutants (13 results) S ['12154123'], Cdc13 (250 results) S ['21490951'], analysis of cdc13-1 (11 results) S ['21490951', '18845848', '18828915'], telomere (12678 results) S ['21490951'], cdc13-1 reveals (4 results) S ['21490951', '18845848', '18828915'], yeast yku70Delta (23 results) S ['21490951', '18756267', '12154123'], cdc13-1 (40 results) S ['21490951', '18845848', '18828915', '18756267', '16564010']
JE	TRUE	6	TRUE	motility share similar cystic (1 results) S ['18178183'], pkd2 morphants (2 results) D ['16216239'], pkd2 morphants (2 results) S ['18178183'], Zebrafish mutations (34 results) S ['18178183'], cilia (12088 results) S ['21490950'], cilia motility share (7 results) S ['18178183'], interaction between Sec10 (5 results) S ['21490950', '12665531'], polycystic (20023 results) S ['21490950'], share similar cystic (83 results) S ['18178183'], pkd2 (487 results) S ['21490950'], Sec10 knockdown (2 results) S ['21490950', '19297529'], Sec10 (35 results) S ['21490950', '19297529', '12665531', '11102522'], exocyst (299 results) S ['21490950'], cilia motility (30 results) S ['19395640', '18178183'], polycystic kidney (8377 results) S ['21490950'], polycystin-2 (271 results) S ['21490950'], share similar cystic phenotypes (15 results) S ['18178183'], cystic phenotypes (2 results) S ['18178183'], differs from pkd2 (3 results) S ['18178183']

PMC3072366	5	FALSE	10	FALSE		7	15	TRUE	13	TRUE	10	TRUE	9	TRU
PMC3072365	3	FALSE	10	FALSE		5	10	FALSE	10	TRUE	8	TRUE	7	TRU
PMC3071630	4	FALSE	4	FALSE		4	8	FALSE	8	FALSE	8	TRUE	5	TRU
PMC3072421	12	FALSE	9	FALSE		8	19	TRUE	18	TRUE	14	TRUE	11	TRU
PMC3072418	2	FALSE	8	FALSE		4	10	TRUE	9	TRUE	9	TRUE	8	TRU

JE	TRUE	5	TRUE	IGF2BP2 (85 results) D ['20550665', '20509872'], T2D case controls (84 results) D ['20512086'], CDKAL1 (98 results) S ['21490949'], Indian T2D case controls (2 results) D ['19247373'], genetic architecture of T2D (5 results) S ['21490949'], European GWAS for T2D (7 results) S ['21490949'], T2D (1052 results) S ['21490949'], Indian T2D (15 results) D ['19247373', '17665514'], architecture of T2D (6 results) S ['21490949'], GWAS (1115 results) S ['21490949'], Asian Indians cases (165 results) S ['21490949', '19892838'], association studies (9722 results) S ['21490949'], genome-wide association studies (2621 results) S ['21490949'], GWAS for T2D (22 results) S ['21490949'], Indian T2D case (7 results) D ['19247373', '17665514']
JE	TRUE	6	TRUE	derivation (11757 results) S ['21490948'], efficient derivation of embryonic (112 results) S ['21490948'], generic pluripotent ground state (1 results) S ['20519324'], Res Establishment of rat (8 results) D ['10896787'], rats exhibit properties consistent (157 results) S ['20519324'], efficient derivation (52 results) S ['21490948'], derivation of embryonic (1174 results) S ['21490948'], generic pluripotent ground (1 results) S ['20519324'], rat embryonic stem cell (7 results) D ['19899136', '11821902'], embryonic stem cells (13148 results) S ['21490948'], generic pluripotent (11 results) S ['20519324'], germline chimerae (1 results) D ['19899136'], stem cells across mammalian (1161 results) S ['21490948']
JE	TRUE	3	TRUE	P450s including CYP2C (16 results) D ['18619574'], food intake measurements (14 results) S ['8320406'], CYP1A2 genes (47 results) D ['19802894'], CYP1A2 genes (47 results) S ['21490707'], CYP1A1 (6756 results) S ['21490707'], human CYP1A1 (227 results) D ['19802894'], CYP2C isoforms (17 results) D ['18619574'], CYP1A2 (3810 results) S ['21490707']
JE	TRUE	4	TRUE	MIB-producing (9 results) D ['21174459', '17489392', '15237602'], MIB-producing (9 results) S ['21490938'], earthy odorant (8 results) D ['18563898', '17873868'], odorant methylisoborneol (8 results) D ['18563898'], ecological control of geosmin (1 results) D ['17400777'], 2-methylisoborneol (146 results) D ['21174459'], 2-methylisoborneol (146 results) S ['21490938'], 2-MIB (31 results) D ['18492804'], 2-MIB (31 results) S ['21490938'], Pseudanabaena (50 results) D ['21174459'], Pseudanabaena (50 results) S ['21490938'], earthy odorant methylisoborneol (3 results) D ['18563898'], cyanobacteria (12367 results) S ['21490938'], raciborskii (131 results) S ['21490938'], bifunctional Streptomyces coelicolor enzyme (21 results) D ['17873868'], 2-MIB biosynthesis (14 results) D ['18492804'], 2-MIB biosynthesis (14 results) S ['21490938'], putative 2-MIB (1 results) S ['21490938'], biosynthesis of 2-methylisoborneol (60 results) D ['21174459', '18563898', '18492804'], biosynthesis of 2-methylisoborneol (60 results) S ['21490938'], control of geosmin (20 results) D ['17400777']
JE	TRUE	5	TRUE	islet miRNAs (23 results) S ['21490936'], islets (39310 results) S ['21490936'], pancreatic islets (8170 results) S ['21490936'], insulin secretion (18716 results) S ['21490936'], t-SNARE proteins improves (11 results) D ['10580425'], Goto-Kakizaki rat (86 results) S ['21490936'], islet gene (41 results) D ['19165461', '17919192'], insulin exocytotic machinery (4 results) S ['21490936'], Goto-Kakizaki (510 results) S ['21490936'], rat pancreatic islet gene (1750 results) S ['21490936']

PMC3072417	12	FALSE	15	FALSE		6	26	TRUE	12	TRUE	12	TRUE	11	TRU
PMC3072416	10	FALSE	7	FALSE		6	13	TRUE	10	TRUE	10	TRUE	7	TRU
PMC3072414	10	FALSE	17	FALSE		6	15	TRUE	4	TRUE	2	TRUE	2	TRU

JE	TRUE	6	TRUE	thermoregulatory tactics (3 results) D ['20038657'], thermoregulatory tactics (3 results) S ['21490935'], thermoregulatory behaviour (56 results) D ['20038657', '17142675'], Coevolution of color (23 results) D ['11926503'], polymorphic pygmy grasshoppers Tetrix (4 results) D ['11926503'], rubber boas physiology (2 results) D ['9231403'], behavioural thermoregulation (65 results) D ['20802122'], behavioural thermoregulation (65 results) S ['21490935'], Individual thermal (12311 results) S ['21490935'], different thermal behaviour tactics (1 results) S ['21490935'], charr (283 results) S ['21490935'], Magnan (634 results) S ['21490935'], lacustrine brook charr (2 results) S ['21490935'], ectotherms (435 results) S ['21490935'], lacustrine brook (4 results) S ['21490935'], grasshoppers Tetrix undulata (4 results) D ['11926503'], thermoregulatory tactics of ectotherms (2 results) D ['20038657'], thermoregulatory tactics of ectotherms (2 results) S ['21490935'], pygmy grasshoppers Tetrix undulata (2 results) D ['11926503'], thermal behaviour tactics (4 results) D ['20038657'], thermal behaviour tactics (4 results) S ['21490935'], tactics of ectotherms (2 results) D ['20038657'], tactics of ectotherms (2 results) S ['21490935'], Salvelinus (753 results) S ['21490935'], thermoregulatory (4422 results) S ['21490935'], complementary shifts of thermal (18 results) D ['17142675'], brook charr (60 results) S ['21490935']
JE	TRUE	5	TRUE	Cells Nijmegen (5412 results) S ['21490934'], Autophagy inhibits reactive oxygen (13 results) D ['19187231'], NOD2 stimulation induces (16 results) D ['19966812'], Crohn's disease (23645 results) S ['21490934'], inflammation dance (36 results) D ['19043437'], Autophagy inhibits reactive (13 results) D ['19187231'], Inflammasome-Independent Modulation (1 results) S ['21490934'], Crohn's (24433 results) S ['21490934'], inflammasome for processing (121 results) S ['21490934'], Inflammasome-Independent Modulation of Cytokine (1 results) S ['21490934'], p38-nuclear factor-kappa (1 results) D ['19187231'], ATG16L1 T300A polymorphism (2 results) D ['19337756'], T300A polymorphism (2 results) D ['19337756'], ATG16L1 (132 results) S ['21490934'], beta posttranslational processing (2 results) D ['9261337'], stimulation induces autophagy (47 results) D ['19966812'], NOD2 stimulation induces autophagy (1 results) D ['19966812']
JE	TRUE	1	TRUE	fossil Laonastes (4 results) S ['21490933', '19694873'], rat Laonastes (2 results) S ['21490933', '19694873'], Masticatory muscle architecture (73 results) S ['19694873'], hystricognathy (2 results) S ['21490933', '19694873'], hystricognathous jaws (2 results) S ['21490933', '19694873'], evolution of hystricognathy (1 results) S ['19694873'], Laonastes aenigmamus (5 results) S ['21490933', '19694873', '16527978'], rock rat Laonastes (2 results) S ['21490933', '19694873'], hystricognathous rodents (4 results) D ['21364934', '17794202'], caviomorph relationships among species (4 results) D ['12644405'], hystricognathous (8 results) D ['21364934', '17794202'], hystricognathous (8 results) S ['21490933', '19694873'], eocene hystricognathous (3 results) D ['21364934', '17794202'], American caviomorph relationships (3 results) D ['12644405'], Laonastes (5 results) S ['21490933', '19694873', '16527978'], family of hystricognathous (2 results) D ['21364934', '17794202'], rat Laonastes aenigmamus (2 results) S ['21490933', '19694873'], eocene hystricognathous rodent (3 results) D ['21364934', '17794202'], New Family of Hystricognathous (1 results) D ['21364934'], masticatory (11139 results) S ['21490933'], Laonastes aenigmamus new insights (1 results) S ['19694873'], South American caviomorph relationships (3 results) D ['12644405'], caviomorph relationships (5 results) D ['12644405'], Laotian rock rat Laonastes (2 results) S ['21490933', '19694873'], aenigmamus new insights (1 results) S ['19694873'], rock rat Laonastes aenigmamus (2 results) S ['21490933', '19694873'], aenigmamus (5 results) S ['21490933', '19694873', '16527978']

PMC3072412	13	FALSE	6	FALSE		16	16	TRUE	8	TRUE	7	TRUE	6	TRU
PMC3072409	12	FALSE	4	FALSE		11	14	TRUE	8	TRUE	7	TRUE	7	TRU
PMC3072407	10	FALSE	10	FALSE		6	20	TRUE	13	TRUE	11	TRUE	9	TRU
PMC3072406	5	FALSE	5	FALSE		4	10	TRUE	9	TRUE	9	TRUE	9	TRU
PMC3072405	7	FALSE	9	FALSE		4	14	TRUE	10	TRUE	9	TRUE	8	TRU

JE	TRUE	2	TRUE	import adaptor Importin-alpha3 (2 results) D ['18423435'], multiple Dishevelled-dependent (3 results) D ['17569865', '11274398'], cuticle (4905 results) S ['21490931'], myristyl-independent activity (1 results) D ['17942091'], Dishevelled-dependent pathways (3 results) D ['17569865', '11274398'], Naked2 escorts (2 results) S ['15064403'], antagonize Wnt signaling (19 results) D ['12354775', '11356022'], adaptor Importin-alpha3 (2 results) D ['18423435'], Dishevelled-dependent (9 results) D ['17569865', '11274398'], myristyl-independent (1 results) D ['17942091'], Nkd1 (31 results) D ['19956716', '17438140'], Nkd1 (31 results) S ['21490931', '20177058', '19888210', '17689523'], multiple Dishevelled-dependent pathways (2 results) D ['17569865', '11274398'], nuclear import adaptor Importin-alpha3 (2 results) D ['18423435'], cuticle engages (2 results) D ['18423435'], antagonize Wnt (100 results) S ['20177058'], Naked2 (7 results) S ['20177058', '18757723', '18504258', '17689523', '17553928', '15064403'], Cuticle bind (69 results) D ['19956716'], Drosophila antagonist (380 results) S ['20177058']
JE	TRUE	4	TRUE	odorant (3436 results) S ['21490930'], adipokinetic hormones produces (5 results) D ['16319199'], immunolocalization of AGalphaq (1 results) D ['17029251'], basal vomeronasal activity-dependent (2 results) D ['19129398'], pheromone-sensitive (67 results) D ['20154200', '20011135', '19301013'], modulate pheromone-sensitive (5 results) D ['19301013'], adipokinetic hormones produces differential (1 results) D ['16319199'], cockroach adipokinetic hormones produces (2 results) D ['16319199'], odorant receptors (1703 results) S ['21490930'], Drosophila odorant receptors (13 results) D ['18625400', '18408711', '16402857', '10943836'], basal vomeronasal activity-dependent expression (2 results) D ['19129398'], olfactory (31381 results) S ['21490930'], modulate pheromone-sensitive olfactory sensilla (2 results) D ['19301013'], cockroach adipokinetic hormones (49 results) D ['16319199'], cockroach adipokinetic (61 results) D ['16319199'], Drosophila odorant (375 results) S ['21490930']
JE	TRUE	5	TRUE	multisensory integration elicits sense (2 results) D ['19820918'], integration elicits (102 results) D ['19820918'], auditory concomitant (421 results) S ['21490928'], own voice during speech (198 results) S ['21490928'], Hands only multisensory integration (14 results) D ['19820918'], own voice (151 results) S ['21490928'], rubber feeling of body (25 results) D ['16280594'], multisensory integration elicits (4 results) D ['19820918'], elicits sense of ownership (2 results) D ['19820918'], Hands only multisensory (33 results) D ['19820918'], elicits sense (80 results) D ['19820918'], Adaptive control of vowel (6 results) S ['16938984'], auditory feedback (853 results) S ['21490928'], only multisensory integration elicits (2 results) D ['19820918'], Perceptual calibration (4 results) S ['11008824'], feedback perturbation (17 results) S ['11008824'], vowel formant evidence (64 results) S ['16938984'], integration elicits sense (6 results) D ['19820918'], formant evidence from real-time (2 results) S ['16938984'], real-time manipulation of formants (2 results) S ['16642842']
JE	TRUE	5	TRUE	cooperation with NFAT (35 results) D ['16873067'], domain activates IKK gamma (52 results) D ['18216269'], FOXP3 controls regulatory (580 results) S ['21490927'], zipper domain activates IKK (4 results) D ['18216269'], Foxp3 (5226 results) S ['21490927'], FOXP3 controls (653 results) S ['21490927'], controls regulatory (12376 results) S ['21490927'], Nuclear factor-kappaB modulates regulatory (32 results) D ['20064449'], c-Rel (1189 results) S ['21490927'], factor-kappaB modulates regulatory (32 results) D ['20064449']
JE	TRUE	7	TRUE	detection of Lobaria (1 results) D ['11555256'], number of bryophyte (133 results) S ['21490926'], habitat fragmentation on eclogical (972 results) S ['21490926'], lichen species (150 results) S ['21490926'], Lobaria pulmonaria diaspores (3 results) D ['11555256'], lichens (1745 results) S ['21490926'], old forests (19 results) S ['21490926'], boreal (1139 results) S ['21490926'], higher number of bryophyte (34 results) S ['21490926'], Species-specific detection of Lobaria (1 results) D ['11555256'], colonization rate of boreal (2 results) D ['19294922'], bryophyte (250 results) S ['21490926'], boreal bryophytes (3 results) D ['19294922'], pulmonaria diaspores (4 results) D ['11555256'], overstory removal on growth (6 results) D ['16827013'], old-growth (285 results) S ['21490926']

PMC3072404	7	FALSE	20	FALSE		6	20	TRUE	15	TRUE	14	TRUE	12	TRU
PMC3072403	7	FALSE	3	FALSE		8	9	TRUE	7	TRUE	7	TRUE	6	TRU
PMC3072402	10	FALSE	16	FALSE		5	22	TRUE	15	TRUE	15	TRUE	12	TRU
PMC3072401	7	FALSE	5	FALSE		4	11	TRUE	7	TRUE	7	TRUE	6	TRU

JE	TRUE	9	TRUE	DeltaNS1 H5N1 influenza (1 results) S ['19543385'], fusion peptide pocket regulate (18 results) D ['19193808'], replication-deficient intranasal DeltaNS1 H5N1 (1 results) S ['19543385'], hemagglutinin protein regulates H5N1 (2 results) D ['19923184'], H5N1 (3992 results) S ['21490925'], intranasal (17157 results) S ['21490925'], regulates H5N1 influenza (7 results) D ['19923184'], protein regulates H5N1 (5 results) D ['19923184'], DeltaNS1 H5N1 influenza vaccine (1 results) S ['19543385'], avian influenza (4701 results) S ['21490925'], H5N1 influenza vaccine (32 results) S ['19543385'], replication-deficient intranasal (38 results) S ['20039806', '19543385'], intranasal DeltaNS1 H5N1 (1 results) S ['19543385'], intranasal DeltaNS1 (6 results) S ['20039806', '19543385'], DeltaNS1 H5N1 (1 results) S ['19543385'], hemagglutinin (10559 results) S ['21490925'], DeltaNS1 (13 results) S ['20039806', '19543385'], fusion peptide pocket (1 results) D ['19193808'], intranasal DeltaNS1 H5N1 influenza (1 results) S ['19543385'], hemagglutinin protein regulates (99 results) D ['19923184'], Adaptation of egg-grown (5 results) D ['9217063'], replication-deficient intranasal DeltaNS1 (3 results) S ['20039806', '19543385'], avian influenza viruses (950 results) S ['21490925'], H5N1 influenza (669 results) S ['21490925'], influenza virus (19416 results) S ['21490925'], infectivity (17938 results) S ['21490925'], influenza vaccine (4479 results) S ['21490925']
JE	TRUE	5	TRUE	efficient DNAzymes against muscle (3 results) D ['10331809'], ribozymes (2483 results) S ['21490924'], versatile chemical component (106 results) D ['20669202'], deoxyribozymes (140 results) D ['20373266'], Unique Purine-Pyrimidine Dinucleotide (5 results) D ['18699741'], deoxyribozyme-strategies (1 results) D ['15578948'], Catalytic nucleic from lab (11 results) D ['10977866'], DNAzyme (325 results) S ['21490924'], Sun L-Q (96 results) D ['20036985'], DNAzymes (262 results) S ['21490924']
JE	TRUE	6	TRUE	QY101 (4 results) S ['21490923', '12766810'], Broad-spectrum biofilm inhibition (12 results) D ['16894146'], Broad-spectrum biofilm inhibition (12 results) S ['21490923'], extracellular polysaccharide mutant (2 results) D ['830643'], gallium disrupts Pseudomonas (1 results) D ['17364024'], disrupts Pseudomonas (50 results) D ['17364024'], disrupts Pseudomonas (50 results) S ['21490923'], polysaccharide (27053 results) S ['21490923'], disrupts Pseudomonas aeruginosa iron (2 results) D ['17364024'], disrupts Pseudomonas aeruginosa iron (2 results) S ['21490923'], pre-A101 (2 results) S ['21490923'], marine bacterium Vibrio (237 results) S ['21490923'], marine bacterium (1205 results) S ['21490923'], transition metal gallium disrupts (3 results) D ['17364024'], metal gallium disrupts Pseudomonas (1 results) D ['17364024'], disrupts Pseudomonas aeruginosa (32 results) D ['17364024'], disrupts Pseudomonas aeruginosa (32 results) S ['21490923'], antibiofilm (127 results) S ['21490923'], antibiofilm activity (43 results) S ['21490923'], metal gallium disrupts (3 results) D ['17364024'], antibiofilm activity of A101 (1 results) S ['21490923'], gallium disrupts Pseudomonas aeruginosa (1 results) D ['17364024'], Vibrio (18994 results) S ['21490923'], A101 (85 results) S ['21490923'], activity of A101 (11 results) S ['21490923'], biofilm formation (4499 results) S ['21490923']
JE	TRUE	5	TRUE	residual bone marrow injury (107 results) S ['19925862'], novel prototype DNA-PK (2 results) D ['16249792'], body irradiation causes residual (271 results) S ['19925862'], blocks NF-kappa B-dependent transcription (18 results) D ['12403772'], irradiation causes residual bone (447 results) S ['19925862'], double-strand (11399 results) S ['21490922'], prototype DNA-PK (2 results) D ['16249792'], prototype DNA-PK inhibitor NU7026 (1 results) D ['16249792'], blocks NF-kappa B-dependent (22 results) D ['12403772'], IKK (2207 results) S ['21490922'], prototype DNA-PK inhibitor (2 results) D ['16249792'], novel prototype DNA-PK inhibitor (2 results) D ['16249792']

PMC3072400	11	FALSE	11	FALSE		8	17	TRUE	13	TRUE	13	TRUE	13	TRU
PMC3072399	7	FALSE	4	FALSE		7	11	TRUE	10	TRUE	10	TRUE	8	TRU
PMC3072398	6	FALSE	4	FALSE		4	3	TRUE	1	TRUE	0	TRUE	0	TRU
PMC3072397	8	FALSE	8	FALSE		6	15	TRUE	14	TRUE	11	TRUE	8	TRU
PMC3072395	2	FALSE	15	FALSE		6	17	FALSE	17	TRUE	14	TRUE	14	TRU

JE	TRUE	11	TRUE	parenchyma vessels co-express aminopeptidase (1 results) D ['10518110'], intermediate filament protein (1185 results) S ['21490921'], filament (23773 results) S ['21490921'], Chemical identification of nestin-immunoreactive (1 results) D ['20153393'], Review Nestin property (1 results) D ['15526158'], nestin-immunoreactive (48 results) D ['20153393', '18805450', '16997483'], adult rat (16288 results) S ['21490921'], intermediate filament (9474 results) S ['21490921'], nestin-immunoreactive neurons (4 results) D ['20153393', '18805450', '16997483'], basal forebrain of adult (10221 results) S ['21490921'], normal adult human forebrain (16509 results) S ['21490921'], Review Nestin (91 results) D ['20429619'], filament protein (1499 results) S ['21490921'], group of nestin-immunoreactive (6 results) D ['16997483', '11769312'], nestin (3063 results) S ['21490921'], Nestin property of multi-lineage (1 results) D ['15526158'], distinct group of nestin-immunoreactive (1 results) D ['16997483'], alterations of nestin-immunoreactive (3 results) D ['18805450'], expression of nestin (1896 results) S ['21490921'], identification of nestin-immunoreactive (2 results) D ['20153393'], basal forebrain (3885 results) S ['21490921'], forebrain (21178 results) S ['21490921']
JE	TRUE	5	TRUE	Ferreira-Gonzalez (87 results) D ['19380859'], Thrombosis Prevention (441 results) S ['21490920'], contemporary cardiovascular systematic review (547 results) D ['21041324'], contemporary cardiovascular systematic (85 results) D ['21041324'], 10-point scale of events (69 results) S ['21490920'], continuous measures with Cronbach's (16 results) D ['2010781'], binary composite outcome (3 results) D ['20719825', '20529275'], composite outcome (517 results) S ['21490920'], Permanyer-Miralda (150 results) D ['19380859'], panel of anaesthesiology (120 results) S ['21490920'], binary composite (19 results) D ['20719825', '20558037', '20529275']
JE	FALSE	0	FALSE	mCASZ1b (1 results) S ['21490919'], CASZ1b (1 results) S ['21490919'], PC12 REST4 (5 results) D ['10490617'], CASZ1 (7 results) S ['21490919', '16631614'], PC12 REST4 silences (1 results) D ['10490617'], transcriptional variant of coexpresses (1 results) D ['17486064'], CASZ1a (1 results) S ['21490919'], Human p16gamma (1 results) D ['17486064'], p16gamma (1 results) D ['17486064'], variant of coexpresses (2 results) D ['17486064']
JE	TRUE	5	TRUE	Periostin advances atherosclerotic (1 results) S ['20551517'], basement membrane (33321 results) S ['21490918'], human fibrotic scar formation (223 results) D ['20531985'], domain suppresses nuclear entry (7 results) D ['15988021'], periostin during skin (12 results) D ['20531985', '17226767'], keratinocyte (11020 results) S ['21490918'], periostin mRNA (25 results) D ['20531985'], periostin mRNA (25 results) S ['21490918'], Periostin advances (2 results) S ['20551517'], human fibrotic scar (456 results) D ['20531985'], rheumatic cardiac valve (6 results) S ['20551517'], fibrotic scar formation (18 results) D ['20531985'], Periostin (265 results) S ['21490918'], Spatiotemporal expression of periostin (7 results) D ['20531985'], rheumatic cardiac valve degeneration (100 results) S ['20551517'], medium accelerates skin (20 results) D ['20206158']
JE	TRUE	10	TRUE	newborn male neurons (5683 results) S ['21490976'], dendritic spine (1185 results) S ['21490976'], anchoring via AKAP150 (6 results) D ['18463244'], glutamate receptor signaling (51 results) S ['19846715'], sex difference correlates (1732 results) S ['21490976'], metabotropic glutamate receptor signaling (25 results) S ['19846715'], many dendritic spine synapses (321 results) S ['21490976'], estradiol's upregulation (6 results) S ['21490976'], AMPA receptor (3784 results) S ['21490976'], AMPA receptor insertion onto (10 results) D ['15009682'], estradiol induction of prostaglandin-E2 (93 results) S ['18726914', '15156148'], masculinization of adult (2839 results) S ['21490976'], masculinization (1073 results) S ['21490976'], AMPA (11274 results) S ['21490976'], perinatal masculinization of adult (36 results) S ['18726914', '15156148'], masculinization of brain (377 results) S ['21490976'], adult sex behavior (53450 results) S ['21490976']

PMC3072394	3	FALSE	17	FALSE		8	17	TRUE	13	TRUE	13	TRUE	13	TRU
PMC3072393	4	FALSE	3	FALSE		3	5	TRUE	4	TRUE	4	TRUE	2	TRU
PMC3072390	3	FALSE	12	FALSE		9	11	TRUE	9	TRUE	9	TRUE	9	TRU
PMC3072389	10	FALSE	6	FALSE		6	16	TRUE	9	TRUE	9	TRUE	6	TRU
PMC3072388	9	FALSE	3	FALSE		9	11	TRUE	7	TRUE	7	TRUE	6	TRU

JE	TRUE	9	TRUE	Cbl (2349 results) S ['21490975'], thymic (19502 results) S ['21490975'], SLP-76 (388 results) S ['21490975'], ZAP-70 (1470 results) S ['21490975'], Vav1 transduces (6 results) D ['14764585', '11994416'], c-Cbl reverses (6 results) S ['19074136', '15238603'], Vav1 (584 results) S ['21490975'], TCR (23107 results) S ['21490975'], c-Cbl (1076 results) S ['21490975'], Cbl Vav1 thymocytes (4 results) S ['21490975'], c-Cbl reverses neonatal (1 results) S ['15238603'], Cbl inactivation (6 results) S ['21490975', '19074136', '15238603'], Vav1 thymocytes (24 results) D ['14764585', '11994416', '9354466'], Vav1 thymocytes (24 results) S ['21490975'], ubiquitin ligase (4738 results) S ['21490975'], thymocytes (14520 results) S ['21490975'], reverses neonatal lethality (1 results) S ['15238603'], Cbl Vav1 (31 results) D ['12881521', '11070165'], Cbl Vav1 (31 results) S ['21490975'], c-Cbl reverses neonatal lethality (1 results) S ['15238603']
JE	TRUE	0	FALSE	rater's specific abilities (1 results) S ['21490974'], Long-term semantic memory versus (27 results) D ['12696812'], semantic memory versus contextual (17 results) D ['12696812'], words modulates conscious access (2 results) D ['16648261'], Janet's (54 results) S ['21490974'], words modulates conscious (15 results) D ['16648261'], buffer zone between fantasy (1 results) S ['21490974']
JE	TRUE	2	TRUE	Gambia Liver Cancer Infection (25 results) D ['8268773'], Gambia Liver Cancer Infection (25 results) S ['18990765', '15734960', '15095302', '14752840'], Liver Cancer (7991 results) S ['21490972'], Gambia Hepatitis Intervention (23 results) S ['18990765', '15095302', '11336472'], activates PEG10 (2 results) D ['17369855'], ethnicity-related (100 results) S ['20162609'], Gambia Hepatitis Intervention assessment (1 results) S ['18990765'], Ser-249TP53 mutation (1 results) S ['15095302'], Gambia Liver Cancer (53 results) S ['21490972', '20162609', '18990765', '15734960', '15095302'], cervix cancer rates (3 results) S ['20162609'], Androgen activates PEG10 (1 results) D ['17369855'], West Evidence from Years (1813 results) S ['21490972'], protective effectiveness against liver (80 results) S ['18990765'], Ser-249TP53 (1 results) S ['15095302'], Gambia Liver (78 results) S ['21490972', '20162609', '18990765']
JE	TRUE	4	TRUE	trans-regulatory control (3 results) D ['19029190'], bean chalcone synthase gene (3 results) D ['8980536'], anthocyanin genes contributes (13 results) D ['19029190'], chalcone (2193 results) S ['21490971'], R2R3-MYB transcription factors controls (18 results) D ['17419845'], anthocyanin (2066 results) S ['21490971'], multiple anthocyanin genes (59 results) D ['19029190'], multiple anthocyanin genes contributes (3 results) D ['19029190'], factors controls flavonol accumulation (4 results) D ['17419845'], flower color (165 results) S ['21490971'], chalcone synthase (614 results) S ['21490971'], motifs essential for floral (13 results) D ['8980536'], floral anthocyanins (5 results) S ['17107490'], trans-regulatory control of gene (43 results) D ['19029190'], adaptive floral anthocyanins (4 results) S ['17107490'], flower color evolution (3 results) D ['19029190', '18298642']
JE	TRUE	4	TRUE	genetic cell ablation (4 results) D ['18571792'], mediate interleukin 1beta induction (147 results) D ['20018936'], deacetylase activity induces developmental (18 results) D ['17855562'], oligodendrocyte (5850 results) S ['21490970'], oligodendrocyte differentiation (423 results) D ['21051629'], oligodendrocyte differentiation (423 results) S ['21490970'], oligodendrocytes interferes (30 results) D ['17515609'], Age-dependent epigenetic control (1 results) S ['19160500'], HDAC2 regulate oligodendrocyte differentiation (2 results) D ['19503085'], HDAC2 regulate oligodendrocyte (2 results) D ['19503085'], activity induces developmental plasticity (38 results) D ['17855562'], mRNA networks (5 results) D ['18987208']

PMC3072387	13	FALSE	5	FALSE		6	9	TRUE	8	TRUE	7	TRUE	4	TRU
PMC3072386	4	FALSE	8	FALSE		5	12	TRUE	10	TRUE	7	TRUE	5	TRU
PMC3072385	10	FALSE	27	FALSE		18	34	TRUE	32	TRUE	32	TRUE	32	TRU

JE	TRUE	3	TRUE	larval insights from tropical (8 results) D ['18422556'], current connectivity for rocky (2 results) D ['19434808'], connectivity for rocky (15 results) D ['19434808'], paleacea (17 results) S ['21490969'], identical dispersal potential (19 results) D ['14871364'], Mitochondrial genomes of Galathealinum (1 results) D ['10666709'], Disparate patterns of post-glacial (1 results) D ['14871364'], predator-resistant shells (1 results) D ['16593946'], paleacea populations (1 results) S ['21490969'], induce more predator-resistant (1 results) D ['16593946'], larval dispersal ability (40 results) D ['19434808', '18422556'], genomes of Galathealinum (1 results) D ['10666709'], more predator-resistant shells (1 results) D ['16593946'], larval (25839 results) S ['21490969'], prey induce more predator-resistant (1 results) D ['16593946'], induce more predator-resistant shells (1 results) D ['16593946'], population structure (4963 results) S ['21490969'], seagrass (356 results) S ['21490969']
JE	TRUE	4	TRUE	atherosclerotic plaques (4773 results) S ['21490968'], fuel CO2 over Europe (8 results) D ['18037473'], Stockholm Atherosclerosis Gene (2 results) S ['19997623'], carotid plaque (1148 results) S ['21490968'], Jungfraujoch observations (6 results) D ['18037473'], Atherosclerosis Gene (6 results) S ['19997623'], muscle-specific AMP deaminase (15 results) D ['15793265'], age of carotid (11758 results) S ['21490968'], Stockholm Atherosclerosis Gene Expression (2 results) S ['19997623'], recent plaque formation time (152 results) D ['20167929'], plaque age (14 results) S ['21490968'], gene module (49 results) S ['19997623']
JE	TRUE	19	TRUE	year-round delivery (27 results) S ['21490967'], delivery strategy EPI (34 results) S ['21490967', '20559558'], IPTi (61 results) D ['20368815', '19765816'], IPTi (61 results) S ['21490967', '20559558'], IPT drug (268 results) D ['21304925', '20574538'], IPT drug (268 results) S ['21490967'], DALYs (447 results) S ['21490967'], transmission intensities (49 results) S ['21490967', '18628828'], IPT cost components (4 results) D ['18826594'], IPT cost components (4 results) S ['21490967'], IPTc (36 results) D ['21304925', '21304923', '21214940', '19789648', '19675675', '19098989'], IPTc (36 results) S ['21490967', '21304921', '20808923'], IPT drug and (268 results) D ['21304925', '20574538'], IPT drug and (268 results) S ['21490967'], intermittent preventive treatment (272 results) D ['21304925', '21304923', '21304919'], intermittent preventive treatment (272 results) S ['21490967', '21304921'], multiple strategies where (979 results) S ['21490967'], intermittent preventive (727 results) D ['21304925', '21304923', '21304919'], intermittent preventive (727 results) S ['21490967', '21304921'], impact on DALYs (125 results) S ['21490967'], IPT (1084 results) S ['21490967'], seasonal delivery (420 results) S ['21490967', '21304921'], target five-year age-band (1 results) S ['21490967'], sulfadoxine-pyrimethamine for malaria (1422 results) S ['21304921'], IPT programme (26 results) S ['21304921'], preventive treatment (2788 results) S ['21490967'], preventive treatment for malaria (1051 results) D ['21304925', '21304923', '21304919'], preventive treatment for malaria (1051 results) S ['21490967', '21304921'], number of DALYs (87 results) S ['21490967'], cost-effectiveness for settings (2043 results) S ['21490967'], five-year age-band for IPTc (1 results) S ['21490967'], seasonal setting (695 results) S ['21490967'], constant transmission settings (24 results) S ['21490967'], age-band for IPTc (1 results) S ['21490967'], other potential IPT (31 results) D ['20574538', '18826594'], substantial protection against malaria (45 results) D ['21304925', '21304923', '17638703'], substantial protection against malaria (45 results) S ['18784833']

PMC3072383	16	FALSE	8	FALSE		10	21	TRUE	15	TRUE	14	TRUE	9	TRU
PMC3072382	1	FALSE	26	FALSE		3	25	TRUE	22	TRUE	19	TRUE	16	TRU
PMC3072378	8	FALSE	4	FALSE		3	11	TRUE	9	TRUE	7	TRUE	6	TRU
PMC3072379	2	FALSE	14	FALSE		4	14	TRUE	9	TRUE	8	TRUE	8	TRU
PMC3072377	1	FALSE	8	FALSE		3	8	FALSE	8	TRUE	7	TRUE	7	TRU

JE	TRUE	6	TRUE	chain homodimers drive proliferation (1 results) D ['2545439'], platelet-derived (18942 results) S ['21490965'], drive proliferation of bipotential (4 results) D ['2545439'], A-chain mRNA heterogeneity (4 results) D ['1360804'], chain homodimers drive (5 results) D ['2545439'], neural stem (4855 results) S ['21490965'], PDGFR alpha-positive (13 results) D ['16846854'], PDGF-A (970 results) S ['21490965'], factor A-chain mRNA heterogeneity (2 results) D ['1360804'], alternative exon usage predicts (7 results) D ['2832727'], Betsholtz (185 results) S ['18483217'], form glioma-like growths (1 results) D ['16846854'], exon usage predicts (14 results) D ['2832727'], glioma-like (18 results) D ['16846854'], glioma-like (18 results) S ['21490965'], homodimers drive proliferation (3 results) D ['2545439'], A-chain transcripts (21 results) D ['2233732'], long splice version (14 results) D ['1782212'], transgenic expression of PDGFB (38 results) D ['20643125'], transgenic expression of PDGFB (38 results) S ['19115382'], PDGF (10765 results) S ['21490965'], neural stem cells (3715 results) S ['21490965'], glioma-like growths (1 results) D ['16846854'], form glioma-like (5 results) D ['16846854']
JE	TRUE	12	TRUE	cerebral pain modulation (4 results) D ['17325514'], accumbens signal (672 results) S ['21490964'], negative expectation (17 results) S ['21490964'], appetitive reward pleasantness (3 results) S ['21490964'], reward task activity (885 results) S ['21490964'], prediction error for relief (6 results) S ['21490964'], ventromedial prefrontal (537 results) S ['21490964'], ventromedial prefrontal cortex (450 results) S ['21490964'], pleasantness (867 results) S ['21490964'], reward pleasantness (39 results) S ['21490964', '18671736'], relief pleasantness (7 results) S ['21490964', '18671736'], task pleasantness (87 results) S ['21490964'], hedonic (1655 results) S ['21490964'], only relief pleasantness (2 results) S ['21490964', '18671736'], nucleus accumbens (12793 results) S ['21490964'], appetitive reward task activity (51 results) S ['21490964'], appetitive reward task (173 results) S ['21490964'], prefrontal cortex (20361 results) S ['21490964'], hedonics (115 results) S ['21490964'], accumbens (13324 results) S ['21490964'], appetitive (4638 results) S ['21490964'], reward task (63 results) S ['21490964'], reward task pleasantness (2 results) S ['21490964'], appetitive reward (12 results) S ['21490964'], reward hedonics (17 results) S ['21490964'], appetitive reward task pleasantness (1 results) S ['21490964'], pleasantness of relief (7 results) S ['21490964', '18671736']
JE	TRUE	4	TRUE	Wnt activation occur (98 results) D ['19377513'], autocrine Wnt activation (38 results) D ['19377513'], pulmonary GOLD executive (8 results) D ['17507545'], human airway (2476 results) S ['21490961'], pulmonary GOLD executive summary (4 results) D ['17507545'], human airway epithelium (216 results) S ['21490961'], Beta-catenin regulates differentiation (393 results) S ['21490961'], airway epithelium (2478 results) S ['21490961'], obstructive pulmonary GOLD executive (6 results) D ['17507545'], pathway aberrations including autocrine (2 results) D ['19377513'], aberrations including autocrine (15 results) D ['19377513'], autocrine Wnt activation occur (1 results) D ['19377513']
JE	TRUE	6	TRUE	exhibits synonymy (6 results) D ['11101562'], Citrobacter rodentium (239 results) S ['21490962'], genome of ICC168 (3 results) S ['21490962', '19897651'], ICC168 genome (3 results) S ['21490962', '19897651'], EX-33 (1 results) S ['21490962'], rodentium strain (46 results) D ['19332804'], rodentium strain (46 results) S ['21490962', '19897651'], rodentium (294 results) S ['21490962'], ICC168 (3 results) S ['21490962', '19897651'], Citrobacter sequence (513 results) S ['21490962'], ICC168 genome sequence (3 results) S ['21490962', '19897651'], strain ICC168 (3 results) S ['21490962', '19897651'], sequence elements on bacterial (21512 results) S ['21490962'], PFGE (5228 results) S ['21490962'], Citrobacter (3512 results) S ['21490962'], strain EX-33 (1 results) S ['21490962']
JE	TRUE	5	TRUE	Resveratrol enhances GLUT-4 translocation (1 results) D ['18266981'], herpesvirus (51510 results) S ['21490960'], Kaposi's (9254 results) S ['21490960'], monocyte metabolism (10 results) S ['21490960'], ligase modulates targets (33 results) S ['17409151'], KSHV (1525 results) S ['21490960'], Kaposi's sarcoma (8486 results) S ['21490960'], ubiquitin ligase (4738 results) S ['21490960'], ubiquitin ligase modulates targets (26 results) S ['17409151']

PMC3072372	5	FALSE	25	FALSE		8	20	TRUE	15	TRUE	14	TRUE	14	TRU
PMC3072369	8	FALSE	14	FALSE		13	22	TRUE	18	TRUE	17	TRUE	13	TRU
PMC3072645	4	FALSE	15	FALSE		4	19	FALSE	19	FALSE	19	FALSE	19	TRU
PMC3072644	6	FALSE	6	FALSE		4	10	FALSE	10	TRUE	3	TRUE	3	TRU

JE	TRUE	9	TRUE	emodepside susceptibility (1 results) S ['21490955'], nematodes (11046 results) S ['21490955'], slo-1 promoters (1 results) S ['21490955'], slo-1 (38 results) S ['21490955', '17962986', '17583712'], restores DAF-16 developmental function (2 results) D ['16442538'], inhibit smooth muscle high-conductance (6 results) D ['7514038'], parasitic nematodes (1175 results) S ['21490955'], anthelmintic (4198 results) S ['21490955'], parasite slo-1 (2 results) S ['21490955'], putative slo-1 (2 results) S ['21490955'], putative slo-1 promoters (1 results) S ['21490955'], Caenorhabditis elegans (16941 results) S ['21490955'], Caenorhabditis elegans behaviour (2 results) S ['17962986', '17325734'], restores DAF-16 developmental (2 results) D ['16442538'], Strongyloides stercoralis restores DAF-16 (1 results) D ['16442538'], elegans slo-1 (24 results) D ['14675531'], elegans slo-1 (24 results) S ['21490955', '17962986', '17583712'], contortus (2081 results) S ['21490955'], novel cyclo-octadepsipeptide (2 results) S ['17962986', '17583712'], elegans slo-1 promoter (2 results) S ['21490955'], Haemonchus contortus (1971 results) S ['21490955'], cyclo-octadepsipeptide (2 results) S ['17962986', '17583712'], potassium channel (16028 results) S ['21490955'], emodepside (33 results) S ['21490955', '17962986', '17583712', '17157854'], parasite slo-1 promoters (1 results) S ['21490955'], cyclo-octadepsipeptide anthelmintic (2 results) S ['17962986', '17583712'], Haemonchus (2521 results) S ['21490955'], Caenorhabditis (17390 results) S ['21490955'], novel cyclo-octadepsipeptide anthelmintic (2 results) S ['17962986', '17583712'], Holden-Dye (89 results) S ['21490955']
JE	TRUE	4	TRUE	Plasmodium knowlesi (645 results) S ['21490952'], knowlesi mtDNA (3 results) S ['21490952'], Kapit (24 results) S ['21490952', '19635025', '18377652', '15051281'], knowlesi infections (28 results) D ['19878553'], knowlesi infections (28 results) S ['19383118', '15051281'], knowlesi (792 results) S ['21490952'], Plasmodium knowlesi infections (20 results) D ['19878553'], Plasmodium knowlesi infections (20 results) S ['19383118', '15051281'], macaques (8061 results) S ['21490952'], cryptic Plasmodium knowlesi malaria (2 results) D ['19284284'], Anopheles latens (5 results) S ['18377652', '16725166'], Plasmodium knowlesi malaria (46 results) D ['19284284', '15663864'], Plasmodium knowlesi malaria (46 results) S ['18171245'], knowlesi malaria (73 results) D ['19478250', '19284284'], knowlesi malaria (73 results) S ['21490952', '19635025', '18377652'], malaria parasite (3622 results) S ['21490952'], large focus (67 results) D ['18710577'], large focus (67 results) S ['18377652'], past population dynamics (14 results) D ['15703244'], Matusop (9 results) S ['21490952', '18377652', '18171245', '15051281'], cryptic Plasmodium knowlesi (2 results) D ['19284284'], differential counts of Plasmodium (20 results) S ['19383118']
JE	TRUE	18	TRUE	Review Preoperative nutritional (312 results) S ['21483641'], colorectal cancer patients (3385 results) S ['21483641'], oil emulsion after gastrointestinal (52 results) D ['20473991'], parenteral nutrition (24237 results) S ['21483641'], postoperative omega-3 (151 results) S ['21483641'], postoperative omega-3 fatty (147 results) S ['21483641'], Enteral (19831 results) S ['21483641'], nutritional risk of colorectal (328 results) S ['21483641'], parenteral nutrition on clinical (6010 results) S ['21483641'], insufficient knowledge of nutritional (115 results) D ['19203814'], omega-3 fatty (3410 results) S ['21483641'], support procedures for colorectal (12427 results) S ['21483641'], emulsion after gastrointestinal (198 results) D ['20473991'], nutrition support (1607 results) S ['21483641'], Review Preoperative nutritional support (231 results) D ['19696603'], Review Preoperative nutritional support (231 results) S ['21483641'], appropriate nutrition support strategies (236 results) S ['21483641'], rectal cancer from January (1083 results) S ['21483641'], appropriate nutritional support procedures (1171 results) S ['21483641']
JE	TRUE	1	TRUE	agonist exaggerates (8 results) S ['18809416'], S-transferase supergene regulation (8 results) D ['8770536'], glutathione S-transferase supergene regulation (9 results) D ['8770536'], receptor agonist exaggerates (7 results) S ['18809416'], GSTM3 (178 results) S ['21483640'], thyrocytes enhances (24 results) D ['17400807'], He-Bin Tang (1 results) S ['21483640'], thyrocytes enhances cellular growth (7 results) D ['17400807'], mouse HCC cells (6 results) S ['21483640'], supergene regulation of GST (6 results) D ['8770536'], thyrocytes enhances cellular (23 results) D ['17400807'], neurokinin-1 receptor agonist exaggerates (1 results) S ['18809416']

PMC3072643	0	TRUE	20	FALSE		2	19	TRUE	16	TRUE	15	TRUE	14	TRU
PMC3072642	8	FALSE	16	FALSE		8	24	TRUE	18	TRUE	17	TRUE	16	TRU
PMC3072641	2	FALSE	18	FALSE		2	20	TRUE	18	TRUE	14	TRUE	13	TRU
PMC3072640	4	FALSE	13	FALSE		4	17	FALSE	17	FALSE	17	TRUE	11	TRU
PMC3072639	1	FALSE	6	FALSE		2	5	FALSE	5	TRUE	4	TRUE	4	TRU

JE	TRUE	12	TRUE	upper gastrointestinal mesenchymal (71 results) S ['21483639'], endoscopic ultrasonography (2324 results) S ['21483639'], endoscopic therapy for mesenchymal (216 results) S ['21483639'], electrosection (19 results) S ['21483639'], upper gastrointestinal (14806 results) S ['21483639'], underwent different endoscopic (2479 results) S ['21483639'], upper GIMTs (3 results) S ['21483639'], submucosal (11415 results) S ['21483639'], snare electrosection (1 results) S ['21483639'], different endoscopic therapies (4 results) S ['21483639'], endoscopic therapy (1718 results) S ['21483639'], endoloop (145 results) S ['21483639'], patients with GIMTs (5 results) S ['21483639', '18505417'], leiomyomas (3057 results) S ['21483639'], mesenchymal tumors (1447 results) S ['21483639'], GIMTs (10 results) S ['21483639', '18505417'], gastrointestinal mesenchymal (1153 results) S ['21483639'], stromal tumors (4637 results) S ['21483639'], underwent different endoscopic therapies (776 results) S ['21483639'], gastrointestinal mesenchymal tumors (55 results) S ['21483639', '18505417']
JE	TRUE	7	TRUE	Oct4 pseudogene (6 results) D ['17280643'], Polymorphism rs6983267 (4 results) S ['21483638'], rs6983267 polymorphisms (32 results) D ['20648012', '20627891', '20530476', '19520795'], rs6983267 polymorphisms (32 results) S ['21483638'], non-cardiac type (217 results) S ['21483638'], non-cardiac type of gastric (11 results) S ['21483638'], gastric cancer of non-cardiac (28 results) S ['21483638'], rs6983267 (60 results) D ['20648012'], rs6983267 (60 results) S ['21483638'], 8q24 (1118 results) S ['21483638'], intestinal-type gastric cancer (170 results) D ['20131315'], risk of gastric (21170 results) S ['21483638'], genotype frequencies of rs6983267 (3 results) S ['21483638'], rs7008482 (4 results) D ['19520795', '17978284'], rs7008482 (4 results) S ['21483638'], association between rs7008482 (4 results) D ['19520795', '17978284'], association between rs7008482 (4 results) S ['21483638'], non-cardiac (2504 results) S ['21483638'], intestinal type (2040 results) S ['21483638'], cancer of non-cardiac (120 results) S ['21483638'], associations between rs6983267 (52 results) D ['20648012', '20627891', '20530476'], associations between rs6983267 (52 results) S ['21483638'], genotype of rs6983267 (47 results) D ['20648012', '20530476'], genotype of rs6983267 (47 results) S ['21483638']
JE	TRUE	12	TRUE	main pattern of reflux (41 results) S ['21483637'], Seventy-eight patients with liver (180 results) S ['21483637'], bile reflux incidence (314 results) S ['21483637'], gastroesophageal endoscopy (9 results) S ['21483637'], simultaneous ambulatory 24-h esophageal (4 results) D ['18613384'], simultaneous ambulatory 24-h esophageal (4 results) S ['21483637'], upper gastroesophageal endoscopy (1210 results) S ['21483637'], gastroesophageal reflux (21754 results) S ['21483637'], cirrhotic patients (7021 results) S ['21483637'], protocol including Child-Pugh (8 results) S ['21483637'], varices (15120 results) S ['21483637'], esophageal motility (2257 results) S ['21483637'], esophageal motor (561 results) S ['21483637'], motor disorders (1474 results) S ['21483637'], upper gastroesophageal (2371 results) S ['21483637'], incidence of LESP (14 results) S ['21483637'], simultaneous ambulatory 24-h (7 results) D ['18613384'], simultaneous ambulatory 24-h (7 results) S ['21483637'], esophageal varices (5219 results) S ['21483637'], cirrhotic patients without esophageal (1162 results) S ['21483637']
JE	TRUE	7	TRUE	burn injury (3812 results) S ['21483636'], burn shock (641 results) S ['21483636', '20061857'], intravenous resuscitation for burn (127 results) S ['21483636'], Enteral resuscitation (14 results) D ['17091077'], Enteral resuscitation (14 results) S ['20061857'], resuscitation of burn (1910 results) S ['21483636'], intestinal absorption of fluid (3448 results) S ['21483636'], oral fluid resuscitation (11 results) S ['21483636'], Enteral resuscitation of burn (54 results) D ['17091077'], Enteral resuscitation of burn (54 results) S ['20061857'], GES for resuscitation (14 results) S ['21483636', '20061857'], pigs with TBSA (37 results) D ['17091077'], oral resuscitation (12 results) D ['12768127'], oral resuscitation (12 results) S ['21483636'], intestinal mucosa (57843 results) S ['21483636'], carbachol (17169 results) S ['21483636'], oral resuscitation of burn (58 results) S ['21483636']
JE	TRUE	2	TRUE	MR-AP (6 results) S ['21483635'], liver lesions (3233 results) S ['21483635'], arterioportography (54 results) S ['21483635'], effectiveness of MR-arterioportography (1 results) S ['21483635'], liver-specific contrast (110 results) S ['21483635'], arterioportal contrast (67 results) D ['18087183'], MR-arterioportography (1 results) S ['21483635']

PMC3072638	0	TRUE	3	FALSE		1	3	FALSE	3	FALSE	3	FALSE	3	FAL
PMC3072637	4	FALSE	20	FALSE		5	24	TRUE	23	TRUE	23	TRUE	21	TRU
PMC3072636	6	FALSE	6	FALSE		7	11	FALSE	11	TRUE	9	TRUE	8	TRU
PMC3072635	0	TRUE	17	FALSE		1	12	FALSE	12	FALSE	12	FALSE	12	TRU
PMC3072634	3	FALSE	17	FALSE		3	16	TRUE	14	TRUE	13	TRUE	12	TRU

.SE	TRUE	3	TRUE	gastrotomy (578 results) S ['21483634'], transgastric (833 results) S ['21483634'], peritoneal cavity (16824 results) S ['21483634']
JE	TRUE	14	TRUE	pivotal role of NKT (37 results) D ['20034047'], intrahepatic NKT (64 results) D ['19318971'], intrahepatic NKT (64 results) S ['21483633'], nonalcoholic fatty liver (1905 results) S ['21483633'], NAFLD (1952 results) S ['21483633'], role of NKT (962 results) S ['21483633'], Th0 cytokine secretion patterns (35 results) D ['10438977'], severe steatosis (187 results) S ['21483633'], higher percentage of intrahepatic (71 results) S ['21483633'], mild steatosis (95 results) S ['21483633'], biopsies for steatosis (1709 results) S ['21483633'], nonalcoholic (4562 results) S ['21483633'], higher percentage of NKT (23 results) S ['21483633'], liver biopsies for steatosis (1692 results) S ['21483633'], nonalcoholic fatty liver disease (1790 results) S ['21483633'], NKT (2335 results) S ['21483633'], cells with nonalcoholic (589 results) S ['21483633'], nonalcoholic fatty (2705 results) S ['21483633'], cells with steatosis (1670 results) S ['21483633'], NKT cells (1898 results) S ['21483633'], percentage of intrahepatic (266 results) S ['21483633'], NKT cells with steatosis (15 results) D ['20512988', '20034047'], NKT cells with steatosis (15 results) S ['21483633'], bariatric Adler (3 results) S ['21483633']
JE	TRUE	4	TRUE	gastric ischemia-reperfusion of rats (207 results) D ['19931544'], gastric ischemia-reperfusion of rats (207 results) S ['21483632', '20198432'], gastric mucosal (7589 results) S ['21483632'], Bax protein (5742 results) S ['21483632'], GI-R injury (10 results) D ['19931544'], GI-R injury (10 results) S ['20198432', '18807130', '17352016'], gastric ischemia-reperfusion (37 results) D ['19931544'], gastric ischemia-reperfusion (37 results) S ['21483632', '20198432', '18807130', '17352016', '17287895'], GI-R (37 results) D ['19931544'], GI-R (37 results) S ['21483632', '20198432', '18807130', '17352016', '17287895'], Topographic association of gastric (12 results) D ['12492185'], Bcl-2 with antralization (1 results) D ['12492185']
JE	TRUE	9	TRUE	prognostic marker (5358 results) S ['21483631'], survival prognosis of gastric (9755 results) S ['21483631'], genome expression (743 results) S ['21483631'], deregulation of ribosome (41 results) S ['21483631'], Illumina HumanWG-6 BeadChip (1 results) S ['21483631'], gastric cancer patients (2801 results) S ['21483631'], ten-gene prognostic (1 results) S ['21483631'], HumanWG-6 BeadChip (1 results) S ['21483631'], independent validation sample batch (1 results) S ['21483631'], Cox regression analysis Interestingly (84 results) S ['21483631'], consistent prognosis (12428 results) S ['21483631'], ribosome (17160 results) S ['21483631'], ten-gene (34 results) S ['21483631'], ribosome protein genes (6958 results) S ['21483631'], ten-gene prognostic marker (1 results) S ['21483631'], consistent prognosis results (7225 results) S ['21483631'], whole genome expression (350 results) S ['21483631']
JE	TRUE	11	TRUE	endoscopic submucosal dissection (582 results) S ['21483630'], resection rates (325 results) S ['21483630'], submucosal (11415 results) S ['21483630'], early gastrointestinal (13830 results) S ['21483630'], Gokhan Dindar (1 results) S ['21483630'], early gastrointestinal cancers (15 results) S ['21483630'], premalignant (6935 results) S ['21483630'], colorectal epithelial neoplasms (10 results) D ['17466600'], endoscopic submucosal (647 results) S ['21483630'], ESD for premalignant (4 results) D ['19032991'], ESD for premalignant (4 results) S ['21483630'], noninvasive early gastrointestinal cancers (142 results) S ['21483630'], Tolga Konduk (1 results) S ['21483630'], ESD procedures (26 results) S ['21483630'], noninvasive early gastrointestinal (101 results) S ['21483630'], submucosal dissection (637 results) S ['21483630'], Goktug Sirin (1 results) S ['21483630'], premalignant lesions (1822 results) S ['21483630'], flat adenomas (147 results) D ['19032991'], Sadettin Hulagu (1 results) S ['21483630']

PMC3072633	2	FALSE	18	FALSE		4	20	TRUE	19	TRUE	17	TRUE	16	TRU
PMC3072631	9	FALSE	10	FALSE		5	19	FALSE	19	TRUE	17	TRUE	14	TRU
PMC3072629	11	FALSE	12	FALSE		10	20	TRUE	17	TRUE	15	TRUE	13	TRU
PMC3072628	12	FALSE	11	FALSE		11	23	TRUE	19	TRUE	19	TRUE	18	TRU

JE	TRUE	14	TRUE	Bonkovsky (247 results) S ['21483629'], total silymarin flavonolignans (2 results) D ['17913795'], CON1 (105 results) S ['21483629'], decrease expression of Bach1 (9 results) S ['16530877'], Nrf2 (1938 results) S ['21483629'], Nrf2 protein (128 results) S ['21483629'], Bach1 (200 results) S ['21483629'], NS5A (837 results) S ['21483629'], human hepatoma (4838 results) S ['21483629'], level of Bach1 (17 results) D ['17942419'], full length HCV genotype (120 results) S ['21483629'], CON1 cells (76 results) S ['21483629'], length HCV genotype (377 results) S ['21483629'], hepatoma cells (7835 results) S ['21483629'], HMOX-1 (39 results) S ['21483629'], full-length HCV genotype (117 results) S ['21483629'], heme oxygenase-1 (4695 results) S ['21483629'], human hepatoma cells (1659 results) S ['21483629'], HCV genotype (2414 results) S ['21483629'], downregulates HCV (7 results) S ['21483629']
JE	TRUE	9	TRUE	hepatic arterio-portal shunts (19 results) D ['20422890'], therapies versus supportive care (418 results) D ['20656222'], hepatectomy (22595 results) S ['21483627'], hepatocellular carcinoma with portal (3587 results) S ['21483627'], vein embolization (486 results) S ['21483627'], vein involvement main portal (64 results) D ['20590905'], intermediate-advanced (18 results) D ['20590905'], intermediate-advanced (18 results) S ['21483627'], locoregional therapies (64 results) D ['20656222', '20594260'], locoregional therapies (64 results) S ['21483627'], portal vein embolization (444 results) S ['21483627'], two-stage hepatectomy (55 results) S ['21483627'], chemoembolization (3755 results) S ['21483627'], locoregional therapies versus (353 results) D ['20656222'], locoregional therapies versus supportive (7 results) D ['20656222'], possible hepatic arterio-portal (10 results) D ['20422890'], hepatic artery (14018 results) S ['21483627'], carcinoma with portal (5655 results) S ['21483627'], hepatic arterio-portal (96 results) D ['20422890']
JE	TRUE	7	TRUE	BMDC-tumor cell fusion (1 results) D ['19055949'], mucosal repair (253 results) S ['21483625'], ILFs (25 results) S ['21483625', '19557549'], BMDC-tumor cell (1 results) D ['19055949'], aggregates of ulcerative (123 results) S ['20132083'], high amount of stroma (148 results) D ['19478385'], pre-cDCs (4 results) D ['20026742'], lymphoid aggregates of ulcerative (62 results) D ['15578077'], lymphoid aggregates of ulcerative (62 results) S ['20132083'], I-II colon cancer (4 results) D ['19478385'], SMAD4 predict (17 results) D ['19478385'], stem cells ameliorates dextran (2 results) D ['18950645'], role of ILFs (12 results) S ['21483625', '19557549'], crypt (8519 results) S ['21483625'], BMDC-tumor (1 results) D ['19055949'], subepithelial myofibroblasts (87 results) S ['21483625', '19557549'], myofibroblasts (4849 results) S ['21483625'], lymphoid follicles (1815 results) S ['21483625'], Review Colonic subepithelial (48 results) D ['16378172'], Review Colonic subepithelial (48 results) S ['19557549'], Review Colonic subepithelial myofibroblasts (7 results) D ['16378172', '10444394'], Review Colonic subepithelial myofibroblasts (7 results) S ['19557549'], subepithelial (5611 results) S ['21483625']
JE	TRUE	13	TRUE	transvaginal (8016 results) S ['21483624'], transesophageal NOTES (5 results) S ['21483624'], transgastric closure (116 results) D ['20644962'], transgastric closure (116 results) S ['21483624'], complications during NOTES (3161 results) S ['21483624'], transgastric NOTES (32 results) D ['20541750'], transluminal endoscopic surgery peritoneoscopy (3 results) D ['20541750'], transvaginal access (42 results) D ['20333406', '19913991', '19717392'], transvaginal access (42 results) S ['21483624'], transesophageal (17649 results) S ['21483624'], natural orifice (839 results) S ['21483624'], spatial orientation during NOTES (55 results) D ['19438294'], transgastric cases (136 results) D ['20504792'], transrectal (6430 results) S ['21483624'], transgastric (833 results) S ['21483624'], prospective NOTES registry (46 results) D ['20504792'], cases of transgastric (136 results) D ['20504792'], gastrotomy site selection (2 results) D ['20541750'], endoscopic surgery peritoneoscopy (3 results) D ['20541750'], majority of transgastric (13 results) S ['19198959'], submucosal tunnel (124 results) D ['20354937'], NOTES procedures (107 results) S ['21483624'], orientation during NOTES (119 results) D ['20644962', '20039826', '19438294']

PMC3071611	0	TRUE	9	FALSE		1	9	FALSE	9	FALSE	9	TRUE	7	TRU
PMC3071610	1	FALSE	10	FALSE		2	11	TRUE	10	TRUE	10	TRUE	10	TRU
PMC3077216	2	FALSE	8	FALSE		2	10	TRUE	9	TRUE	9	TRUE	9	TRU
PMC3071871	5	FALSE	11	FALSE		6	13	TRUE	9	TRUE	9	TRUE	6	TRU
PMC3071844	10	FALSE	11	FALSE		9	21	FALSE	21	TRUE	18	TRUE	17	TRU

JE	TRUE	7	TRUE	management of bronchiolitis (798 results) S ['21471175'], acute management of bronchiolitis (287 results) S ['21471175'], admissions on day (2576 results) S ['21471175'], safety outcomes for bronchiolitis (19 results) S ['21471175'], bronchiolitis (8080 results) S ['21471175'], bronchodilators (3428 results) S ['21471175'], intervention bronchodilator (1634 results) S ['21471175'], single large trial (14 results) S ['21471175'], large trial (398 results) S ['21471175']
JE	TRUE	9	TRUE	statin users (292 results) S ['21471172'], short period of statin (138 results) S ['21471172'], month period after pneumonia (427 results) S ['21471172'], conventional multivariable methods (2 results) D ['16632131'], propensity score (2057 results) S ['21471172'], period of statin (1951 results) S ['21471172'], period after pneumonia (2965 results) S ['21471172'], statin users with non-users (57 results) S ['21471172'], non-users (2344 results) S ['21471172'], effect of statins (7819 results) S ['21471172'], statin treatment (1406 results) S ['21471172']
JE	TRUE	8	TRUE	experiences of pregnancy (4617 results) S ['21499449'], Most studies of pregnancy (136229 results) S ['21499449'], Young single women's experiences (22 results) D ['15177861'], existential (1962 results) S ['21499449'], women's experiences of pregnancy (1044 results) S ['21499449'], life opening (1724 results) S ['21499449'], early pregnancy (10682 results) S ['21499449'], single women's experiences (2 results) D ['15177861'], women's experiences (1011 results) S ['21499449'], phenomenological study of women's (276 results) S ['21499449']
JE	TRUE	4	TRUE	Chile psychiatric prevalence study (3 results) D ['16877648'], prioritization approach (11 results) S ['19602527'], prioritization (2036 results) S ['21494644'], Antidepressant medication change (2 results) D ['10520974'], multi-dimensional (2115 results) S ['21494644'], complex diseases-schizophrenia (1 results) S ['19602527'], candidate gene prioritization approach (47 results) S ['21494644', '19602527'], Chile psychiatric prevalence (3 results) D ['16877648'], candidate gene (8927 results) S ['21494644'], gene prioritization approach (2 results) S ['19602527'], candidate gene prioritization (18 results) S ['19602527'], DEPgenes (1 results) S ['21494644'], postpartum depression point (137 results) D ['20029403'], diseases-schizophrenia (1 results) S ['19602527'], gene prioritization (52 results) S ['21494644'], composite index of neuroticism (17 results) D ['15351774']
JE	TRUE	9	TRUE	Oxford classification (23 results) D ['19571791', '19571790'], biglycan (1026 results) S ['21494642'], growth factor-beta (43188 results) S ['21494642'], NLRP3 inflammasome via toll-like (7 results) D ['19605353'], GBM heparan sulfate proteoglycan (105 results) D ['19144998'], inflammasome via toll-like (22 results) D ['19605353'], IgA pathology definitions (7 results) D ['19571790'], mesangial (11225 results) S ['21494642'], patterns of decorin (117 results) S ['21494642'], Podocyte proteoglycan (48 results) D ['19144998', '18660676'], Podocyte proteoglycan (48 results) S ['16622173', '15585670'], Podocyte proteoglycan synthesis (31 results) D ['18660676'], Podocyte proteoglycan synthesis (31 results) S ['16622173', '15585670'], original Cockcroft (6 results) D ['19404852'], expression patterns of decorin (77 results) S ['21494642'], IgA rationale (46 results) D ['19571791'], proteoglycans (23643 results) S ['21494642'], IgA (40062 results) S ['21494642'], Oxford classification of IgA (20 results) D ['19571791', '19571790'], factor-beta (43985 results) S ['21494642'], decorin (1830 results) S ['21494642']

PMC3071843	7	FALSE	23	FALSE		6	27	TRUE	26	TRUE	22	TRUE	20	TRU
PMC3071842	2	FALSE	10	FALSE		5	12	TRUE	11	TRUE	11	TRUE	11	TRU
PMC3071841	0	TRUE	3	FALSE		1	3	FALSE	3	FALSE	3	FALSE	3	FAL
PMC3071840	7	FALSE	2	FALSE		4	9	TRUE	6	TRUE	6	TRUE	4	TRU
PMC3071839	7	FALSE	21	FALSE		15	25	TRUE	23	TRUE	18	TRUE	18	TRU

JE	TRUE	13	TRUE	dyslexics (609 results) S ['21494641'], fixation point during conjugate (21 results) D ['9196724'], variability for dyslexics (7 results) S ['21494641'], loose yoking of saccades (1 results) S ['19003978'], human binocular fixation (1098 results) S ['20573592'], binocular fixation point (7 results) D ['19757909', '9196724'], disconjugacy drift during fixations (6 results) S ['21494641', '20573592', '19003978'], non-conjugate gaze-shifts (1 results) D ['9196724'], disparity (12771 results) S ['21494641'], yoking (55 results) S ['21494641', '19003978'], disconjugacy drift (11 results) S ['21494641', '20573592', '19003978'], drift during fixations (22 results) S ['21494641', '20573592', '19003978'], yoking of saccades (15 results) S ['21494641', '19003978'], disconjugacy (54 results) S ['21494641', '20573592', '19003978'], human binocular fixation point (130 results) D ['19757909'], saccade disconjugacy (10 results) S ['19003978'], Free exploration (84 results) S ['19003978'], dyslexic (1306 results) S ['21494641'], eye movements (26308 results) S ['21494641'], binocular fixation during reading (78 results) D ['19757909', '18729571'], binocular fixation during reading (78 results) S ['21494641', '20573592'], binocular fixation (130 results) D ['19757909'], binocular fixation (130 results) S ['21494641'], fixation disparity (254 results) D ['19757909'], fixation disparity (254 results) S ['21494641', '20573592'], loose yoking (1 results) S ['19003978'], fixation during reading (1404 results) S ['21494641'], saccades (8556 results) S ['21494641'], Kapoula (91 results) S ['21494641', '19003978'], binocular (10982 results) S ['21494641']
JE	TRUE	6	TRUE	Differential expression of interleukin-17 (55 results) S ['20020510'], granzyme (2796 results) S ['21494640'], allograft (34821 results) S ['21494640'], rejection correlates with inflammation (42 results) S ['19424039'], Low numbers of FOXP3 (131 results) S ['21494640'], vasculopathy (4779 results) S ['21494640'], allograft vasculopathy (897 results) S ['21494640'], Intragraft FOXP3 protein (30 results) D ['19357258'], Intragraft FOXP3 protein (30 results) S ['19424039'], FOXP3 (5226 results) S ['21494640'], Renal Allograft Updates (4 results) D ['18294345'], renal allograft rejection correlates (96 results) S ['19424039']
.SE	TRUE	3	TRUE	Hollis (1891 results) S ['21494639'], plasma vitamin (1847 results) S ['21494639'], development of prostate (10927 results) S ['21494639']
JE	TRUE	2	TRUE	Constitutive transcriptional activation (22 results) D ['9065401'], Regulation of cyclooxygenase-2 (5348 results) S ['21494638'], tumor suppressor counteracts beta-catenin (5 results) D ['16510874'], suppressor counteracts beta-catenin (5 results) D ['16510874'], Chem Regulation of cyclooxygenase-2 (16 results) D ['11274170'], APC tumor suppressor counteracts (3 results) D ['16510874'], counteracts beta-catenin (25 results) D ['16510874'], cyclooxygenase-2 (19386 results) S ['21494638'], counteracts beta-catenin activation (15 results) D ['16510874']
JE	TRUE	11	TRUE	nigrostriatal pathway dopaminergic (3 results) S ['21494637', '19630976', '18590612'], repeat kinase (313 results) S ['21494637', '21248115'], transgenic mice (64982 results) S ['21494637'], pathway dopaminergic (5714 results) S ['21494637'], LRRK2 transgenic (31 results) D ['20659558', '20130188', '19890007'], LRRK2 transgenic (31 results) S ['21494637', '20064389', '19741132', '19503083'], LRRK2 transgenic mice (30 results) D ['20659558', '20457918', '20130188', '19890007'], LRRK2 transgenic mice (30 results) S ['21494637', '20064389', '20016100', '19503083'], motor performance with LRRK2 (2 results) D ['20130188'], G2019S LRRK2 transgenic (11 results) D ['20659558', '20130188', '19890007', '18258746'], G2019S LRRK2 transgenic (11 results) S ['21494637', '19741132', '18367605'], leucine-rich (3757 results) S ['21494637'], disease mutation G2019S (193 results) S ['21248115'], nigrostriatal pathway dopaminergic neurons (3 results) S ['21494637', '19630976', '18590612'], Parkinson's disease mutation G2019S (188 results) S ['21248115'], G2019S LRRK2 transgenic mice (10 results) D ['20659558', '20130188', '19890007'], G2019S LRRK2 transgenic mice (10 results) S ['21494637', '18367605'], LRRK2 (660 results) S ['21494637'], performance with LRRK2 (9 results) D ['20130188'], dopaminergic neurons (7300 results) S ['21494637'], G2019S LRRK2 (36 results) D ['20659558', '19625511'], G2019S LRRK2 (36 results) S ['21494637', '21248115'], G2019S (212 results) S ['21494637', '21248115'], pathway dopaminergic neurons (3 results) S ['21494637', '19630976', '18590612'], G2019S LRRK2 induces (4 results) S ['21494637'], leucine-rich repeat kinase (310 results) S ['21494637', '21248115'], leucine-rich repeat (2096 results) S ['21494637'], expression of G2019S (27 results) S ['21494637', '21248115']

PMC3071838	2	FALSE	13	FALSE		5	13	TRUE	9	TRUE	9	TRUE	9	TRU
PMC3071837	4	FALSE	7	FALSE		5	11	TRUE	9	TRUE	8	TRUE	8	TRU
PMC3071833	0	TRUE	25	FALSE		1	22	TRUE	20	TRUE	20	TRUE	18	TRU
PMC3071832	7	FALSE	5	FALSE		6	11	TRUE	7	TRUE	7	TRUE	7	TRU
PMC3071831	1	FALSE	20	FALSE		5	16	TRUE	15	TRUE	14	TRUE	10	TRU

JE	TRUE	6	TRUE	U19MH081835 (3 results) S ['20419144'], Minocycline down-regulates (2 results) D ['17395590'], reveals neuroprotection (58 results) S ['20479889'], resonance spectroscopy (141865 results) S ['21494695'], SIV (4549 results) S ['21494695'], NeuroAIDS (194 results) S ['21494695'], resonance spectroscopy reveals neuroprotection (2 results) S ['20479889'], HIV perivascular (288 results) S ['20419144'], R01NS06897 (1 results) S ['20419144'], Minocycline down-regulates MHC (1 results) D ['17395590'], oral minocycline (82 results) S ['20479889'], resonance spectroscopy reveals (29 results) S ['20479889', '16110325'], spectroscopy reveals neuroprotection (3 results) S ['20479889'], minocycline (5012 results) S ['21494695'], magnetic resonance spectroscopy (117535 results) S ['21494695']
JE	TRUE	6	TRUE	WHO guideline (13963 results) S ['21494694'], cohort of tuberculosis (2133 results) S ['21494694'], Zomba Hospital (2 results) D ['9861414'], abdominal ultrasound for diagnosis (27761 results) S ['21494694'], Diagnosis of smear-negative (757 results) S ['21494694'], smear-negative (961 results) S ['21494694'], immediate treatment initiation (3 results) S ['21494694'], Review Diagnosis of smear-negative (81 results) D ['17624822', '17574096'], Kang'ombe (11 results) D ['11055765', '9861414'], Nyangulu (31 results) D ['11055765', '9861414'], smear-negative pulmonary (149 results) S ['21494694']
JE	TRUE	12	TRUE	gophers (119 results) S ['21494690'], Deinandra fasciculata (1 results) S ['21494690'], herbivores (2994 results) S ['21494690'], californica (3985 results) S ['21494690'], conspecifics (2083 results) S ['21494690'], defenses from tolerance (302 results) S ['21494690'], mainland populations more tolerant (2 results) S ['21494690'], gopher herbivory (3 results) S ['21494690'], herbivory (1296 results) S ['21494690'], root herbivory (24 results) S ['21494690'], populations from gophers (22 results) S ['21494690'], Captive gophers (1 results) S ['21494690'], alkaloid (10907 results) S ['21494690'], pocket gophers (70 results) S ['21494690'], fasciculata (2545 results) S ['21494690'], root damage (139 results) S ['21494690'], Tolerance- Are mainland populations (12 results) S ['21494690'], belowground plant (26 results) S ['21494690'], mainland populations (103 results) S ['21494690'], Deinandra (1 results) S ['21494690'], Tolerance- Are mainland (26 results) S ['21494690'], more tolerant of root (414 results) S ['21494690'], belowground herbivory (15 results) S ['21494690'], belowground (472 results) S ['21494690'], island conspecifics (61 results) S ['21494690']
JE	TRUE	5	TRUE	review board practices (3 results) D ['15173501'], readability (1111 results) S ['21494689'], Readability standards for informed-consent (295 results) D ['19884804'], consent forms for oncology (65 results) D ['19884804'], assent forms for multicenter (3 results) D ['16651328'], Institutional review board practices (3 results) D ['15173501'], Readability of pediatric (225 results) S ['21494689'], study information (178 results) S ['21494689'], reading ease of study (211 results) S ['21494689'], actual readability (1 results) D ['12594317'], Readability standards (5 results) D ['15577434', '12594317'], pediatric RIL (26 results) S ['21494689']
JE	TRUE	9	TRUE	phospholipase D1-mTORC2 (1 results) S ['21228924'], Morphoproteomic (20 results) S ['21494688', '21228924', '19429803', '16000081'], insulin-like growth (40238 results) S ['21494688'], Ewing's sarcoma merits (12 results) S ['21494688'], Ewing's (6254 results) S ['21494688'], sarcoma merits (97 results) S ['21494688'], D1-mTORC2 pathway (1 results) S ['21228924'], mTOR inhibitor (841 results) S ['21494688'], Morphoproteomic analysis reveals (4 results) S ['21228924'], human monoclonal antibody insulin-like (842 results) D ['20975071'], morphoproteomic assessment (1 results) S ['21494688'], patients with Ewing's (2177 results) S ['21494688'], phospholipase D1-mTORC2 pathway (1 results) S ['21228924'], insulin-like (42371 results) S ['21494688'], IGF1R inhibitor (11 results) S ['21494688'], IGF1R (464 results) S ['21494688'], Ewing's sarcoma (5969 results) S ['21494688'], Morphoproteomic analysis (10 results) S ['21494688', '21228924'], sarcoma merits further investigation (15 results) S ['21494688'], D1-mTORC2 (1 results) S ['21228924'], rapamycin (8873 results) S ['21494688']

PMC3071830	2	FALSE	9	FALSE		9	7	TRUE	6	TRUE	6	TRUE	4	TRU
PMC3071829	6	FALSE	3	FALSE		4	6	TRUE	4	TRUE	4	TRUE	3	TRU
PMC3071828	4	FALSE	17	FALSE		7	17	TRUE	9	TRUE	9	TRUE	7	TRU
PMC3071827	14	FALSE	3	FALSE		9	15	TRUE	9	TRUE	9	TRUE	7	TRU
PMC3071826	10	FALSE	6	FALSE		7	8	TRUE	4	TRUE	3	TRUE	2	TRU

JE	TRUE	4	TRUE	beta-cell (16033 results) S ['21494687'], beta cell (16033 results) S ['21494687'], islets (39310 results) S ['21494687'], pancreatic beta (8934 results) S ['21494687'], UFM1 (18 results) D ['20018847', '18448419', '18321862', '17182609', '16617122', '15071506'], UFM1 (18 results) S ['21494687'], factor alpha dephosphorylation potentiates (4 results) S ['17158450'], glucose-inducible insulin special reference (1 results) D ['2163307'], UFBP1 (1 results) S ['21494687'], UFM1-UFBP1 (1 results) S ['21494687'], UFM1-UFBP1 conjugation (1 results) S ['21494687']
JE	TRUE	2	TRUE	impairs circadian synchronisation (1 results) D ['12181309'], circadian (62985 results) S ['21494686'], hyperleptinaemia (127 results) S ['21494686'], circadian synchronisation (2 results) D ['12181309'], stress causes higher emotionality (39 results) D ['17658641'], Prenatal hypoxia impairs circadian (1 results) D ['12181309'], hypoxia impairs circadian synchronisation (1 results) D ['12181309'], hypoxia impairs circadian (4 results) D ['12181309'], gestation on 6-sulphatoxymelatonin (13 results) S ['11316330']
JE	TRUE	2	TRUE	mid-height selective (1 results) S ['21494685'], snouts (105 results) S ['21494685'], diplodocoid (4 results) S ['21494685', '18030355'], Nigersaurus (3 results) S ['21494685', '18030355'], diplodocoid sauropods (3 results) S ['21494685', '18030355'], complete sauropod dinosaur skull (6 results) S ['20179896', '18030355'], Diplodocus (13 results) D ['10221910'], Diplodocus (13 results) S ['21494685'], large proportion of pits (33 results) S ['21494685'], dental microwear (84 results) S ['21494685'], sauropod dentition (4 results) S ['20179896'], sauropod (70 results) S ['21494685', '20179896'], microwear (137 results) S ['21494685'], diplodocoids (4 results) D ['10558986'], diplodocoids (4 results) S ['21494685', '18030355'], evolution of sauropod (27 results) D ['18845734'], evolution of sauropod (27 results) S ['20179896'], incisors of ceropithecid (1 results) D ['822732'], snout shape (5 results) S ['21494685'], snouts of Dicraeosaurus (1 results) S ['21494685'], Dicraeosaurus (1 results) S ['21494685']
JE	TRUE	4	TRUE	temporal window for audiovisual (17 results) D ['19793985', '16137867'], audiovisual speech (140 results) D ['20117103'], precedes cross-modal (5 results) D ['19817154'], requires transient audiovisual events (1 results) D ['20498844'], Signals Requires Transient Audiovisual (1 results) D ['20498844'], asynchronous (5728 results) S ['21494684'], asynchronous audiovisual speech extends (2 results) D ['16137867'], visual speech stream (60 results) D ['20117103'], visual speech stream (60 results) S ['21494684'], signals requires transient (230 results) D ['20498844'], visual speech (236 results) S ['21494684', '20419130'], audiovisual speech extends (4 results) D ['16137867'], window for audiovisual (26 results) D ['19793985', '16137867'], precedes cross-modal integration (4 results) D ['19817154'], Transient Audiovisual Events (4 results) D ['20498844'], auditory signals requires transient (4 results) D ['20498844'], temporal ventriloquism (20 results) D ['19817154', '16846297', '12763201']
JE	TRUE	1	TRUE	genome epidemiologic study (2 results) D ['17299477'], Asian populations uncovers genetic (1 results) S ['19396169'], Pax6 (1712 results) S ['21494683'], Concise Pax6 transcription (1 results) D ['18467663'], populations uncovers genetic factors (11 results) S ['19396169'], enhancers reflect global gene (2 results) D ['19295514'], Asian populations uncovers (1 results) S ['19396169'], Concise Pax6 (1 results) D ['18467663'], requires mGluR-PLCbeta4 signaling (1 results) D ['12640460'], populations uncovers genetic (40 results) S ['19396169'], Insomnia increases insulin (5 results) D ['20398173'], Concise Pax6 transcription factor (1 results) D ['18467663'], Insomnia increases insulin resistance (5 results) D ['20398173'], suprachiasmatic nucleus requires mGluR-PLCbeta4 (1 results) D ['12640460'], large-scale genome-wide association study (8 results) S ['19396169'], requires mGluR-PLCbeta4 (1 results) D ['12640460']

PMC3071825	2	FALSE	24	FALSE		8	22	TRUE	14	TRUE	12	TRUE	10	TRU
PMC3071824	15	FALSE	9	FALSE		13	24	TRUE	21	TRUE	18	TRUE	16	TRU
PMC3071823	14	FALSE	10	FALSE		10	22	TRUE	18	TRUE	18	TRUE	17	TRU

JE	TRUE	4	TRUE	periodical cicadas Magicicada (25 results) S ['21494682', '16674582'], Allochronic speciation (10 results) D ['11005299'], Allochronic speciation (10 results) S ['11298977'], periodical cicadas (33 results) S ['21494682', '19451640', '19146596', '16674582'], Magicicada neotredecem (1 results) S ['11005298'], cicada contact zone (6 results) S ['16674582', '11298977'], cicadas Magicicada (25 results) S ['21494682', '16674582'], numerical model of periodical (25 results) S ['21494682', '19451640', '19146596'], 13-year Magicicada (6 results) S ['21494682', '16674582', '11298977', '11005298'], periodical cicada evolution (13 results) D ['12683538'], periodical cicada evolution (13 results) S ['19146596', '11298977', '11005298'], prime-numbered (3 results) S ['19451640', '19146596'], broods (652 results) S ['21494682'], cicadas (109 results) S ['21494682', '19451640', '19146596'], neotredecem (1 results) S ['11005298'], cicadas Magicicada genetic (3 results) S ['16674582', '11298977'], Allee effect (139 results) S ['21494682'], Magicicada (31 results) S ['21494682', '16674582'], periodical cicada contact (5 results) S ['16674582', '11298977'], periodical cicada contact zone (2 results) S ['16674582', '11298977'], Magicicada genetic (4 results) S ['16674582', '11298977'], asymmetrical reproductive character displacement (3 results) S ['16674582'], periodical cicadas Magicicada genetic (3 results) S ['16674582', '11298977'], Cooley (2890 results) S ['21494682'], support for allochronic (23 results) S ['11298977'], 13-year Magicicada neotredecem (1 results) S ['11005298']
JE	TRUE	9	TRUE	high intra-population diversity (18 results) D ['16847467'], Colloquium human adaptations (3 results) D ['20445095', '20445092'], NAT2 (1241 results) S ['21494681'], NAT2 implications (32 results) D ['19379125', '18680468', '16550165'], NAT2 implications (32 results) S ['18304320'], human arylamine (1664 results) S ['21494681'], nucleotide diversity of NAT2 (10 results) D ['19164093', '18043717', '16847467', '16786516'], nucleotide diversity of NAT2 (10 results) S ['18304320'], advantageous amino acid (5 results) D ['18773084'], NAT2 gene supports (8 results) D ['18043717'], reveals homogeneity across Native (2 results) D ['16847467'], Review Interethnic (103 results) D ['18680468'], arylamine (2869 results) S ['21494681'], homogeneity across Native (20 results) D ['16847467'], Multiple advantageous amino (70 results) D ['18773084'], Multiple advantageous amino acid (89 results) D ['18773084'], N-acetyltransferase (4446 results) S ['21494681'], NAT2 gene (177 results) D ['20026257'], NAT2 gene (177 results) S ['21494681'], advantageous amino acid variants (35 results) D ['18773084'], human populations (5217 results) S ['21494681'], arylamine N-acetyltransferase (2355 results) S ['21494681'], region reveals homogeneity (69 results) D ['16847467'], human arylamine N-acetyltransferase (55 results) D ['20026257', '18680468', '16786516']
JE	TRUE	14	TRUE	virus-like particles reveals (33 results) D ['17204159'], NiV proteins (296 results) D ['21050901'], NiV proteins (296 results) S ['21494680'], Nipah (419 results) S ['21494680'], outer-layer proteins VP4 (1 results) D ['9151849'], renders antigens (2 results) D ['12163261'], Nipah virus proteins (193 results) D ['21050901'], Nipah virus proteins (193 results) S ['21494680'], paramyxovirus measles virus (194 results) D ['20552729'], paramyxovirus measles virus (194 results) S ['21494680'], Nipah virus (361 results) D ['21050901'], Nipah virus (361 results) S ['21494680'], challenge with SCoV (1 results) D ['18191004'], analysis of Nipah (181 results) D ['21050901', '20181713'], analysis of Nipah (181 results) S ['21494680'], virus-like (4768 results) S ['21494680'], particles reveals central role (14 results) D ['17204159'], terminal trailer region (21 results) D ['9847334'], terminal trailer region (21 results) S ['17698665'], virus-like particles (3618 results) S ['21494680'], VLPs (1447 results) S ['21494680'], Chimeric coronavirus-like particles (2 results) D ['18191004'], Chimeric coronavirus-like (2 results) D ['18191004'], Quantitative analysis of Nipah (3 results) D ['17204159']

PMC3071821	6	FALSE	13	FALSE		7	19	TRUE	17	TRUE	16	TRUE	11	TRU
PMC3071820	10	FALSE	10	FALSE		14	19	TRUE	17	TRUE	16	TRUE	14	TRU
PMC3071819	1	FALSE	15	FALSE		2	12	TRUE	10	TRUE	9	TRUE	9	TRU
PMC3071818	7	FALSE	15	FALSE		4	20	TRUE	15	TRUE	13	TRUE	10	TRU

JE	TRUE	7	TRUE	Review Male-male (40 results) D ['19120810'], total sexual selection (2 results) D ['19120810'], behavioural estimates (11 results) D ['12969481'], temporal pattern of female (8170 results) S ['21494678'], indirect direct female (10623 results) S ['21494678'], fallow (859 results) S ['21494678'], fallow deer (431 results) S ['21494678', '20380690'], Cumulative long-term investment (17 results) D ['10328804'], older female matings (35 results) S ['21494678'], mate choice (1263 results) S ['21494678'], dominant fallow deer males (3 results) S ['20380690'], Review Male-male competition (14 results) D ['19120810', '9155244'], deer over years (197 results) S ['21494678', '20380690'], indirect direct female mate (77 results) S ['21494678'], older male matings (26 results) S ['21494678'], assortative (1059 results) S ['21494678'], fallow deer over years (10 results) S ['21494678', '20380690'], success of fallow (16 results) D ['14676951', '12969481', '10328804'], success of fallow (16 results) S ['21494678']
JE	TRUE	8	TRUE	serum energetics (64 results) D ['18488985'], bovine serum albumin (30572 results) S ['21494677'], anticancer alkaloid sanguinarine (18 results) D ['21318089', '20442937', '15299076', '12700925'], anticancer alkaloid sanguinarine (18 results) S ['21494677'], alkanolamine (45 results) D ['12437384'], alkanolamine (45 results) S ['21494677'], benzophenanthridine alkaloids (71 results) D ['20378534', '17253712'], serum albumin (81409 results) S ['21494677'], alkaloid sanguinarine (45 results) D ['20442937'], alkaloid sanguinarine (45 results) S ['21494677'], iminium (699 results) S ['21494677'], alkaloid (10907 results) S ['21494677'], bovine serum (26395 results) S ['21494677'], phenazinium dye with serum (1 results) D ['20113428'], alkanolamine form (7 results) D ['12437384', '7802700', '2700895'], alkanolamine form (7 results) S ['21494677'], serum albumin conformational changes (2 results) D ['15293315'], benzophenanthridine (150 results) D ['21318089', '20378534'], sanguinarine (522 results) D ['21318089', '21281702'], sanguinarine (522 results) S ['21494677']
JE	TRUE	8	TRUE	ocular surface (3736 results) S ['21494676'], TGs-1 (1 results) S ['21494676'], muscarinic receptor (7308 results) S ['21494676'], Stevens-Johnson syndrome conjunctiva (2 results) D ['10027391'], sclera (10447 results) S ['21494676'], down-regulates TGs-1 (1 results) S ['21494676'], muscarinic antagonist down-regulates TGs-1 (1 results) S ['21494676'], muscarinic (28485 results) S ['21494676'], way of muscarinic (386 results) S ['21494676'], scleral (8960 results) S ['21494676'], manipulation of TGs (5 results) S ['21494676'], plausible method of intervention (183 results) S ['21494676'], antagonist down-regulates TGs-1 (1 results) S ['21494676'], muscarinic antagonist down-regulates (6 results) S ['21494676'], TGs (1031 results) S ['21494676'], scleral fibroblasts (74 results) S ['21494676']
JE	TRUE	5	TRUE	alkali digestion value (44 results) S ['21494675'], japonica (7050 results) S ['21494675'], GBSS1 (5 results) S ['21494675'], SBE3-5 (2 results) D ['16964521'], quality of japonica (161 results) S ['21494675'], palatability (1785 results) S ['21494675'], rice starch biosynthesis lead (11 results) D ['20018713'], SBE3 (5 results) D ['16964521'], SBE3 (5 results) S ['21494675'], SBE1 (22 results) D ['16964521'], SBE1 (22 results) S ['21494675'], expressions of SBE1 (1 results) S ['21494675'], SBE1-5 (11 results) D ['16964521'], nonwaxy rice sativa (7 results) D ['16964521'], japonica rice (247 results) S ['21494675'], Eight japonica rice varieties (8 results) S ['21494675'], diverse array of rice (44 results) D ['20018713'], palatability rice varieties (3 results) S ['21494675', '19334756'], Eight japonica rice (51 results) S ['21494675'], SBE3 expressions (1 results) S ['21494675'], palatability varieties (13 results) S ['21494675', '19334756'], starch synthase (419 results) S ['21494675']

PMC3071816	10	FALSE	7	FALSE		8	16	TRUE	15	TRUE	12	TRUE	8	TRU
PMC3071815	3	FALSE	21	FALSE		5	24	TRUE	23	TRUE	22	TRUE	20	TRU
PMC3071814	1	FALSE	12	FALSE		7	8	TRUE	7	TRUE	7	TRUE	7	TRU
PMC3071813	5	FALSE	8	FALSE		3	13	FALSE	13	TRUE	12	TRUE	12	TRU
PMC3071812	1	FALSE	9	FALSE		5	9	TRUE	7	TRUE	7	TRUE	5	TRU
PMC3071810	4	FALSE	2	FALSE		4	4	TRUE	3	TRUE	3	TRUE	2	TRU

JE	TRUE	3	TRUE	flavonoid naringenin (45 results) D ['18587069'], flavonoid naringenin (45 results) S ['18393287'], oral bioavailability of rutin (16 results) D ['10934740'], Apolipoprotein B-dependent hepatitis (1 results) S ['18393287'], flavanones naringenin (25 results) D ['11160539'], Caco-2 (8930 results) S ['21494673'], B-dependent hepatitis (9 results) S ['18393287'], citrus flavanone aglycones (19 results) D ['17047689'], naringenin (1131 results) S ['21494673'], flavanone aglycones (8 results) D ['17047689'], flavonoid (7218 results) S ['21494673'], aglycones hesperetin (35 results) D ['17047689'], citrus flavonoid naringenin (7 results) D ['15919788', '14514640'], Apolipoprotein B-dependent (2 results) S ['18393287'], citrus flavanone aglycones hesperetin (15 results) D ['17047689'], citrus flavanone (23 results) D ['17047689'], flavanone aglycones hesperetin (20 results) D ['17047689']
JE	TRUE	16	TRUE	pathway with complementary (6034 results) S ['21494672'], visual cortex (23242 results) S ['21494672'], neuritis (12804 results) S ['21494672'], nerve fiber (5243 results) S ['21494672'], potential measure of axonal (152 results) D ['18825432'], optic neuritis (5459 results) S ['21494672'], resonance semi-parametric (12 results) S ['15906296'], association of RNFLT (10 results) D ['18054962'], retinal nerve fiber layer (1399 results) S ['21494672'], Optical coherence (9666 results) S ['21494672'], coherence tomography (7595 results) S ['21494672'], association of BPF (22 results) D ['17938370'], matter NAA (21 results) S ['21494672'], visual pathway (1901 results) S ['21494672'], nerve fiber layer (2121 results) S ['21494672'], visual cortex NAA (25 results) S ['21494672'], optical coherence tomography (9502 results) S ['21494672'], white matter NAA (13 results) S ['21494672'], RNFLT (77 results) S ['21494672'], cortex NAA (3 results) S ['21494672'], normal-appearing (3904 results) S ['21494672'], BPF (381 results) S ['21494672'], fiber layer (2367 results) S ['21494672'], cortex NAA concentrations (145 results) S ['21494672']
JE	TRUE	7	TRUE	K14-Cdx2 basal (1 results) S ['21494671'], keratinocytes (24959 results) S ['21494671'], Cdx2 (852 results) S ['21494671'], K14-Cdx2 mice (1 results) S ['21494671'], Cdx2 expression (200 results) S ['21494671'], K14-Cdx2 basal keratinocytes (1 results) S ['21494671'], Cdx2 expression activates cell-cell (1 results) S ['14977637'], K14-Cdx2 (1 results) S ['21494671'], DK068366 (8 results) S ['19734199', '18819935', '18231635', '17463179'], esophagus (61798 results) S ['21494671'], basal cells (4692 results) S ['21494671'], Short exposure of oesophageal (148 results) D ['18593808'], Barrett's (5335 results) S ['21494671']
JE	TRUE	9	TRUE	human retrovirus XMRV murine (83 results) D ['21084477'], immune escape virulence factor (81 results) D ['20142478'], XMRV murine leukemia (123 results) S ['21494670'], xenotropic (675 results) S ['21494670'], xenotropic murine (197 results) S ['21494670'], protein of oncoretroviruses (34 results) D ['20142478'], envelope protein of oncoretroviruses (7 results) D ['20142478'], murine leukemia (12137 results) S ['21494670'], XMRV murine (128 results) S ['21494670'], XMRV (108 results) S ['21494670'], virus-related (2322 results) S ['21494670'], escape virulence factor (176 results) D ['20142478'], xenotropic murine leukemia (166 results) S ['21494670']
JE	TRUE	3	TRUE	unfamiliar yawns (2 results) S ['21494669'], outgroup (1554 results) S ['21494669'], chimpanzees (4474 results) S ['21494669'], videos of unfamiliar (15 results) S ['21494669'], familiar yawns (1 results) S ['21494669'], contagious yawning (25 results) D ['19452178', '18682357', '17698452', '17148320'], contagious yawning (25 results) S ['21494669'], ingroup-outgroup bias (4 results) S ['21494669'], ingroup-outgroup (19 results) S ['21494669'], yawning (879 results) S ['21494669']
JE	TRUE	2	TRUE	sponsor's incentive (4 results) D ['18363220'], antidepressants on cancer (3398 results) S ['21494667'], antidepressant (24882 results) S ['21494667'], Hormetic effects of hormones (15 results) D ['16422394'], industry sponsor's incentive (1 results) D ['18363220'], antipsychotic tricyclic compounds (1 results) D ['6127156']

PMC3071809	7	FALSE	8	FALSE		8	15	TRUE	12	TRUE	9	TRUE	9	TRU
PMC3071808	8	FALSE	6	FALSE		16	11	TRUE	7	TRUE	5	TRUE	4	TRU
PMC3071806	2	FALSE	13	FALSE		3	15	FALSE	15	TRUE	14	TRUE	10	TRU
PMC3071805	8	FALSE	8	FALSE		4	15	TRUE	12	TRUE	9	TRUE	9	TRU
PMC3071804	5	FALSE	13	FALSE		5	16	TRUE	12	TRUE	10	TRUE	8	TRU

JE	TRUE	7	TRUE	adult Malians (9 results) S ['17940609'], ligand C3dg (4 results) D ['11352728'], C2A domain of synaptotagmin (128 results) S ['19874007'], C2A domain (116 results) S ['19874007'], C2A (376 results) S ['21494666'], Efficient site-specific (76 results) S ['19874007'], complement receptor (2389 results) S ['21494666'], new radiopharmaceutical (262 results) S ['21494666', '19874007'], complement receptor type (725 results) D ['20332377'], antigen diabody (2 results) D ['17268029'], radiopharmaceutical (3935 results) S ['21494666'], Stable one-step technetium-99m (7 results) D ['10471933'], affinity Fcgamma receptors IIa (24 results) D ['11544262'], distinct thermodynamic properties (2 results) D ['11544262'], anti-carcinoembryonic antigen diabody (10 results) D ['17268029']
JE	TRUE	2	TRUE	High diversity of Panton-Valentine (6 results) D ['19571020', '18508934'], High diversity of Panton-Valentine (6 results) S ['17949441'], diversity of Panton-Valentine (31 results) D ['19571020', '19484277', '19402958', '18508934'], Azores predominance of SCCmec (1 results) D ['20229224'], Panton-Valentine (922 results) S ['21494333'], diverse genotypes of methicillin-resistant (48 results) D ['20084094', '19887459'], Atlantic Azores predominance (1 results) D ['20229224'], mec type rapid identification (6 results) D ['17043114'], type ccr gene complexes (14 results) D ['19064897', '18676883', '16207957'], Azores predominance (2 results) D ['20229224'], leukocidin gene reveal (2 results) S ['17581935'], Q6GD50 (1 results) S ['19911206'], Panton-Valentine leukocidin gene reveal (2 results) S ['17581935'], type ccr gene (218 results) S ['20525989']
JE	TRUE	8	TRUE	scene perception (96 results) S ['21494331'], overt visual attention (18 results) S ['21494331'], emotional dimensions modulate (15 results) D ['15929647'], emotional dimensions modulate (15 results) S ['21494331'], modulatory effect of valence (14 results) S ['21494331'], visual attention (2517 results) S ['21494331'], dimensions modulate (169 results) S ['21494331'], emotional stimuli (1068 results) S ['21494331'], task-relevant distractors (8 results) D ['14640845'], arousal (35631 results) S ['21494331'], scan path (50 results) S ['21494331'], emotional dimensions (124 results) S ['21494331'], modulatory (13523 results) S ['21494331'], larger shifts of attention (117 results) S ['21494331'], modulatory effect (2144 results) S ['21494331']
JE	TRUE	7	TRUE	DPC method (814 results) S ['21494330'], Arabidopsis thaliana cor15b (7 results) D ['8260628'], posttranscriptional regulation of starch (6 results) D ['15347792'], thaliana cor15b (5 results) D ['8260628'], partial correlation (1736 results) S ['21494330'], multivariate time series inference (21 results) S ['21494330'], time series (11670 results) S ['21494330'], vector autoregressive process (6 results) D ['17493252'], homologue of cor15a (2 results) D ['8260628'], DPC (2640 results) S ['21494330'], causal networks (28 results) D ['17493252'], effective model selection (3 results) D ['17493252'], apparent homologue of cor15a (1 results) D ['8260628'], multivariate inference techniques (242 results) S ['21494330'], starch metabolism (238 results) S ['21494330'], inference (12378 results) S ['21494330']
JE	TRUE	5	TRUE	blood lead concentration post-ban (1 results) S ['21494329'], California condors (14 results) D ['17051813', '12685072'], lead ammunition (21 results) D ['17051813', '12739854'], lead ammunition (21 results) S ['21494329'], turkey vultures (22 results) D ['12685072'], turkey vultures (22 results) S ['21494329'], vultures (140 results) S ['21494329'], eagles from Finland (2 results) D ['16846196'], lead concentration (1329 results) S ['21494329'], blood lead concentration (517 results) S ['21494329'], lead exposure (3472 results) S ['21494329'], sea eagles from Finland (2 results) D ['16846196'], blood lead (4323 results) S ['21494329'], concentration post-ban (7 results) S ['21494329'], lead concentration post-ban (1 results) S ['21494329'], hunter compliance with lead (9 results) S ['21494329'], ammunition ban (4 results) S ['21494329'], lead ammunition ban (2 results) S ['21494329']

PMC3071803	3	FALSE	8	FALSE		5	11	FALSE	11	FALSE	11	TRUE	9	TRU
PMC3071801	1	FALSE	6	FALSE		2	7	FALSE	7	FALSE	7	FALSE	7	TRU
PMC3062458	2	FALSE	9	FALSE		3	11	FALSE	11	FALSE	11	FALSE	11	TRU
PMC3062457	4	FALSE	9	FALSE		6	12	FALSE	12	TRUE	9	TRUE	8	TRU
PMC3062442	14	FALSE	4	FALSE		14	16	TRUE	12	TRUE	10	TRUE	8	TRU
PMC3078892	3	FALSE	20	FALSE		6	18	FALSE	18	TRUE	16	TRUE	11	TRU

JE	TRUE	5	TRUE	Relative Brain Size (99 results) S ['21494328'], Relative Brain (218 results) S ['21494328'], Brain size (1113 results) S ['21494328'], Big brains (14 results) D ['15784743'], novel environments (361 results) S ['21460547'], Establishment success (38 results) D ['18554145'], Establishment success (38 results) S ['21494328', '21460547'], amphibians increases (3557 results) S ['21460547'], practical guide for ecology (15 results) D ['19185386'], success of mammal (111754 results) S ['21494328'], presence of congeneric (79 results) S ['21460547']
JE	TRUE	5	TRUE	vultures (140 results) S ['21494326'], carrion (1075 results) S ['21494326'], lead exposure (3472 results) S ['21494326'], wild pig (75 results) D ['20043786'], wild pig (75 results) S ['21494326'], lead concentration (1329 results) S ['21494326'], blood lead (4323 results) S ['21494326']
JE	TRUE	8	TRUE	non-pathogenic (2858 results) S ['21460019'], leptospirosis (6632 results) S ['21460019'], isothermal amplification (568 results) S ['21460019'], inner primer (26 results) D ['10871386'], lipL41 (44 results) D ['19070450'], lower limit of detection (6868 results) S ['21460019'], diagnostic sensitivity (3431 results) S ['21460019'], intermediate group (558 results) S ['21460019'], Queensland Health Forensic (21 results) S ['21460019'], Leptospira (5326 results) S ['21460019'], Leptospira spp (220 results) S ['21460019']
JE	TRUE	5	TRUE	Amblyomma (1685 results) S ['21460018'], fever group (832 results) S ['21460018'], Rickettsia (6980 results) S ['21460018'], lowland (1925 results) S ['21460018'], variegatum ticks (55 results) S ['15381820'], Rickettsia africae (76 results) S ['21460018'], rickettsial agents africae (6 results) D ['12954562'], case of ATBF (11 results) D ['10447320'], africae only (23 results) D ['15546086'], africae infection (9 results) D ['11357153'], africae infection (9 results) S ['21460018'], africae (102 results) S ['21460018'], Emmanuel Mpoudi-Ngole (1 results) S ['21460018']
JE	TRUE	3	TRUE	malaria chemoprophylaxis (325 results) S ['21460003'], Mefloquine versus doxycycline (4 results) D ['14604928', '11463107', '9025805'], Israeli Air Force aircrew (9 results) D ['9025805'], versus quinine-doxycycline (1 results) D ['16407341'], cost considerations of malaria (28 results) D ['16968914'], intermittent exposure of Israeli (2 results) D ['9025805'], generic doxycycline monohydrate (9 results) D ['16968914'], comparative tolerability of doxycycline (15 results) D ['9182474'], doxycycline monohydrate (27 results) D ['12390596', '1764965'], tolerability of doxycycline (42 results) D ['17990426'], tolerability of doxycycline (42 results) S ['21460003'], chemoprophylaxis (4330 results) S ['21460003'], versus doxycycline for malaria (8 results) D ['16407341', '14604928', '11463107', '9025805', '1621888'], field trial of azithromycin (27 results) D ['9455524'], doxycycline (9783 results) S ['21460003'], artemether-lumefantrine versus quinine-doxycycline (1 results) D ['16407341'], drug-drug interactions with doxycycline (5 results) D ['2610502'], healthy nonimmune volunteers (13 results) D ['8291822']
JE	TRUE	9	TRUE	aminopeptidase (10736 results) S ['21466698'], Drosophila evolution (16 results) S ['21466698'], genomic investigations reveal heterogeneity (14 results) D ['19420050'], S-LAPs (1 results) S ['21466698'], additional insect genome (523 results) S ['20833280'], canonical M17 (1 results) S ['21466698'], S-LAP family (1 results) S ['21466698'], functional diversification (339 results) S ['21466698'], active site indicates (4157 results) S ['21466698'], gene family during Drosophila (6946 results) S ['21466698'], Drosophila sperm proteome (10 results) S ['21466698', '20833280', '18653731', '17099714'], sperm proteome (20 results) D ['18340633'], sperm proteome (20 results) S ['21466698', '20833280', '18653731', '17099714'], M17 (451 results) S ['21466698'], gene creation (16 results) S ['21466698', '18653731'], leucyl (16000 results) S ['21466698'], S-LAP gene (1 results) S ['21466698'], comparative genomic investigations reveal (31 results) D ['19420050'], S-LAP gene family (1 results) S ['21466698'], gene family composition (2733 results) S ['21466698'], S-LAP (7 results) S ['21466698'], Drosophila sperm (45 results) S ['21466698'], family during Drosophila (8966 results) S ['21466698']

PMC3078836	2	FALSE	15	FALSE		3	17	FALSE	17	TRUE	15	TRUE	15	TRU
PMC3078869	4	FALSE	12	FALSE		4	16	TRUE	14	TRUE	13	TRUE	13	TRU
PMC3071377	9	FALSE	7	FALSE		7	10	TRUE	8	TRUE	7	TRUE	4	TRU
PMC3071036	2	FALSE	13	FALSE		2	14	FALSE	14	FALSE	14	TRUE	13	TRU
PMC3071418	0	TRUE	6	FALSE		4	6	FALSE	6	FALSE	6	TRUE	5	TRU
PMC3071417	3	FALSE	3	FALSE		2	6	TRUE	5	TRUE	3	TRUE	1	TRU
PMC3071416	5	FALSE	1	FALSE		5	6	TRUE	5	TRUE	5	TRUE	5	TRU

JE	TRUE	11	TRUE	Information motivational support (5111 results) S ['21466699'], e-health support (326 results) S ['21466699'], process measures (681 results) S ['21466699'], LTCs (71 results) S ['21466699'], potential benefits of anonymous (115 results) S ['21466699'], Seven process measures (1370 results) S ['21466699'], people with LTCs (6 results) S ['21466699'], Internet self-efficacy (10 results) D ['19041453'], e-health support intervention (25 results) D ['18554819'], e-health support intervention (25 results) S ['21466699'], number of Internet (4581 results) S ['21466699'], LTC (2128 results) S ['21466699'], relevant information 'Information motivational (97 results) S ['21466699'], information 'Information motivational support (605 results) S ['21466699'], participants' confidence (716 results) S ['21466699'], e-health (874 results) S ['21466699'], information 'Information motivational (988 results) S ['21466699']
JE	TRUE	9	TRUE	H-reflex latency (89 results) D ['20417153'], H-reflex with clinical (643 results) S ['21466665'], patients with radiculopathy (2704 results) S ['21466665'], H-reflex amplitude (277 results) S ['21466665'], H-reflex amplitude asymmetry (10 results) S ['21466665'], reflex amplitude (536 results) S ['21466665'], method of Jankus (4 results) D ['8291959'], root involvement (168 results) S ['21466665'], amplitude asymmetry (74 results) S ['21466665'], radiculopathy (4931 results) S ['21466665'], H-reflex amplitude use (787 results) S ['21466665'], H-reflex (2675 results) S ['21466665'], nerve root involvement (84 results) S ['21466665'], nerve root (4587 results) S ['21466665'], S1-foramen H-reflex (3 results) D ['20417153'], stable H-reflex amplitude (27 results) D ['11234570']
JE	TRUE	4	TRUE	discretionary points award (3 results) D ['12663405'], high impact decisions (904 results) S ['21467101'], adjudication (634 results) S ['21467101'], relative odds (637 results) S ['21467101'], examinations-equal opportunities (1 results) D ['10657339'], General Medical (7159 results) S ['21467101'], Bias against foreign-born (2 results) D ['21070342'], referral for adjudication (14 results) S ['21467101'], Oral examinations-equal (1 results) D ['10657339'], impact decisions (16 results) S ['21467101'], non-white consultants (2 results) D ['15090443', '12663405'], Shaista Hickman (1 results) S ['21467101'], Oral examinations-equal opportunities (1 results) D ['10657339'], points award comparison (6 results) D ['12663405'], discretionary points award comparison (1 results) D ['12663405'], Nunez-Smith (14 results) D ['20502974', '17200221']
JE	TRUE	12	TRUE	mean proportion (561 results) S ['21467104'], sample of NSABP (15 results) D ['9474067'], twenty-year sample of NSABP (1 results) D ['9474067'], mean proportion of criteria (2517 results) S ['21467104'], subsequent publications (82 results) S ['21467104'], information on eligibility (1483 results) S ['21467104'], reporting of eligibility (529 results) S ['21467104'], prespecified (2204 results) S ['21467104'], eligibility criteria (3177 results) S ['21467104'], proportion of eligibility (606 results) S ['21467104'], narrower study (2508 results) S ['21467104'], narrower study population (310 results) S ['21467104'], Many users of trial (362 results) S ['21467104'], trial protocols (212 results) S ['21467104'], broader study population (1628 results) S ['21467104']
JE	TRUE	2	TRUE	Biostatistics Yusuf (13 results) S ['18479744', '16875901'], PeriOperative ISchemic Evaluation (620 results) S ['21422131'], extended-release metoprolol succinate (22 results) S ['18479744'], POISE Trial (23 results) S ['21422131', '18479744'], Devereaux (389 results) S ['21422131'], metoprolol versus placebo (81 results) S ['17070177', '16875901']
JE	TRUE	1	TRUE	Joint Health (184 results) S ['21422129'], Alberta Bone (18 results) D ['19366494'], Alberta Bone (18 results) S ['21422129'], Joint Health Institute (10 results) D ['19366494'], Joint Health Institute (10 results) S ['21422129'], Alberta Hip (2 results) D ['19366494']
JE	TRUE	4	TRUE	LMWH versus usual patient (7 results) D ['19635277', '17208082', '17145251'], venous thromboembolic American College (56 results) D ['18574272'], Long-term heparin versus (169 results) D ['19635277'], heparin therapy (2631 results) S ['21422138'], proximal-vein thrombosis (106 results) D ['17208082', '17145251'], versus usual patient satisfaction (116 results) D ['19635277']

PMC3071415	1	FALSE	10	FALSE		2	11	TRUE	10	TRUE	10	TRUE	8	TRU
PMC3071414	1	FALSE	9	FALSE		2	10	TRUE	8	TRUE	7	TRUE	7	TRU
PMC3071386	0	TRUE	19	FALSE		2	19	TRUE	16	TRUE	16	TRUE	12	TRU
PMC3071385	1	FALSE	10	FALSE		3	11	FALSE	11	FALSE	11	FALSE	11	TRU
PMC3073201	1	FALSE	10	FALSE		3	11	FALSE	11	TRUE	9	TRUE	8	TRU
PMC3073200	0	TRUE	6	FALSE		1	6	FALSE	6	FALSE	6	FALSE	6	FAL
PMC3073199	0	TRUE	11	FALSE		1	11	FALSE	11	FALSE	11	FALSE	11	TRU

JE	TRUE	3	TRUE	analysis studies (829 results) S ['21402681'], Nikolaos Polyzos (33 results) S ['21402681'], Antonis Valachis (18 results) S ['21402681'], Valachis (26 results) S ['21402681'], baseline assumptions (96 results) S ['21402681'], thresholds of cytologic (12 results) D ['10819705'], cost-effectiveness diagnostic accuracy (1120 results) S ['21402681'], Pap test (1236 results) S ['21402681'], lower baseline assumptions (69 results) S ['21402681'], manufacturer involvement (38 results) S ['21402681'], cost-effectiveness analysis studies (5 results) S ['21402681']
JE	TRUE	2	TRUE	Pei-Kun Sung (4 results) S ['21398246'], Cheng-Chieh Lin (155 results) S ['21398246'], lower BMI cutoff value (39 results) S ['21398246'], Shin-Li Tsai (2 results) S ['21398246'], Jeanine Albu (52 results) S ['21398246'], Current definitions of obesity (93 results) S ['21398246'], Pi-Sunyer (435 results) S ['21398246'], Taiwanese implications of obesity (6 results) D ['12587007'], Kuo-Chin Huang (76 results) S ['21398246'], Wen-Yuan Lin (34 results) S ['21398246']
JE	TRUE	7	TRUE	low-priority triage (13 results) S ['21398248'], lower-priority emergency department (4 results) S ['21398248'], worse performance on indices (97 results) S ['21398248'], Atzema (17 results) S ['21398248', '19157653'], low-priority (646 results) S ['21398248'], depression on triage (88 results) S ['21398248'], benchmark times for patients (135 results) S ['21398248'], meeting benchmark times (16 results) S ['21398248'], triage score (52 results) S ['21398248', '19157653'], lower-priority emergency department triage (3 results) S ['21398248'], emergency department triage (126 results) S ['21398248'], department triage (126 results) S ['21398248'], lower-priority emergency (5 results) S ['21398248'], department triage score (617 results) S ['21398248'], Acuity Scale (78 results) S ['21398248'], benchmark time of minutes (96 results) S ['21398248'], emergency department triage score (429 results) S ['21398248'], analysis with triage (2473 results) S ['21398248'], Clare Atzema (16 results) S ['21398248', '19157653']
JE	TRUE	9	TRUE	central laboratory repository (56 results) S ['19840369'], Brenda Hemmelgarn (129 results) S ['21422125'], high vascular risk ONTARGET (32 results) D ['18707986'], angiotensin-receptor blocker (987 results) S ['21422125'], combination angiotensin (7666 results) S ['21422125'], ACE inhibitor (6489 results) S ['21422125'], renal dysfunction with combination (3372 results) S ['21422125'], users of monotherapy (144 results) S ['21422125'], hyperkalemia (6114 results) S ['21422125'], renal outcomes (379 results) S ['21422125'], Finlay McAlister (170 results) S ['21422125']
JE	TRUE	7	TRUE	occupational cancer (546 results) S ['21489219'], many additional workplace (89 results) S ['21489219'], ECNIS (7 results) D ['19079723'], many additional workplace exposures (17 results) S ['21489219'], carcinogens (139322 results) S ['21489219'], workplace exposures (285 results) S ['21489219'], occupational exposures (2721 results) S ['21489219'], human carcinogenesis (967 results) S ['21489219'], white men (2754 results) S ['21489219'], human carcinogens (544 results) S ['21489219'], quantitative exposure assessments (8 results) S ['1553986']
.SE	TRUE	6	TRUE	acute lymphoblastic (18883 results) S ['21489218'], acute lymphoblastic leukaemia (3461 results) S ['21489218'], lymphoblastic leukaemia (3804 results) S ['21489218'], leukaemia (27464 results) S ['21489218'], best therapy differs (247 results) S ['21489218'], childhood cancer (3788 results) S ['21489218']
JE	TRUE	10	TRUE	social inequalities (942 results) S ['21489217'], great variability across countries (23 results) S ['21489217'], knowledge on effectiveness (10461 results) S ['21489217'], survival across countries (1172 results) S ['21489217'], cancer incidence (9354 results) S ['21489217'], poor prognosis stomach (1980 results) S ['21489217'], present knowledge on effectiveness (944 results) S ['21489217'], remarks on policies (136 results) S ['21489217'], more evaluation studies (166284 results) S ['21489217'], effectiveness of policies (9565 results) S ['21489217'], cancer survival (2063 results) S ['21489217']

PMC3073196	1	FALSE	8	FALSE		2	9	FALSE	9	TRUE	8	TRUE	8	TRU
PMC3073195	2	FALSE	19	FALSE		3	21	TRUE	19	TRUE	17	TRUE	15	TRU
PMC3073194	4	FALSE	3	FALSE		2	6	FALSE	6	FALSE	6	FALSE	6	TRU
PMC3073193	7	FALSE	1	FALSE		2	8	TRUE	6	TRUE	6	TRUE	6	TRU
PMC3073192	1	FALSE	9	FALSE		3	8	FALSE	8	TRUE	5	TRUE	4	TRU
PMC3073191	2	FALSE	10	FALSE		2	7	FALSE	7	TRUE	6	TRUE	6	TRU
PMC3073190	0	TRUE	8	FALSE		3	8	FALSE	8	TRUE	7	TRUE	4	TRU

JE	TRUE	8	TRUE	epidemiological research (3207 results) S ['21489214'], example of radon (109 results) S ['21489214'], radon (5677 results) S ['21489214'], no-threshold relationship (8 results) D ['19332842'], evidence on radon (242 results) S ['21489214'], epidemiological research on radiation (7836 results) S ['21489214'], epidemiological data (8120 results) S ['21489214'], radon progeny (309 results) S ['21489214'], dose-response relationship (345388 results) S ['21489214']
JE	TRUE	14	TRUE	THMs (387 results) S ['21489213'], carcinogenicity (11346 results) S ['21489213'], certificate studies (1508 results) S ['21489213'], carcinogenicity of dioxins (140 results) S ['21489213'], hundreds of DBPs (5 results) S ['21489213'], misclassification (3830 results) S ['21489213'], dioxins (6798 results) S ['21489213'], extreme exposure misclassification (12 results) S ['21489213'], exposure assessment (2978 results) S ['21489213'], DBPs (489 results) S ['21489213'], exposure misclassification (274 results) S ['21489213'], death certificate studies (5 results) S ['21489213'], cancer research on dioxins (624 results) S ['21489213'], Achilles heel for studies (264 results) S ['21489213'], wide contrast of exposure (656 results) S ['21489213'], epidemiological research on DBPs (45 results) S ['21489213'], carcinogenicity of DBPs (10 results) D ['17980649'], carcinogenicity of DBPs (10 results) S ['21489213'], study designs (3416 results) S ['21489213'], toxicological evidence on routes (16 results) S ['21489213'], phenoxy herbicides (130 results) D ['17119216']
JE	TRUE	4	TRUE	Gambia Hepatitis Intervention (23 results) D ['18990765'], low-resource (815 results) S ['21489212'], Gambia Hepatitis Intervention assessment (1 results) D ['18990765'], present contribution summarizes (115 results) S ['21489212'], cancer control (5984 results) S ['21489212'], protective effectiveness against liver (80 results) D ['18990765'], protective effectiveness (105 results) D ['18990765']
JE	TRUE	5	TRUE	promising interventions into health (1361 results) S ['21489211'], aromatic hydrocarbon DNA (110 results) D ['20658679'], aromatic hydrocarbon DNA adducts (85 results) D ['20658679'], hydrocarbon DNA adducts (114 results) D ['20658679'], adductomic (3 results) D ['20658679'], polycyclic aromatic hydrocarbon DNA (109 results) D ['20658679'], adductomic method (3 results) D ['20658679'], hydrocarbon DNA (146 results) D ['20658679']
JE	TRUE	4	TRUE	Tomatis (592 results) S ['21489210'], primary prevention of cancer (20520 results) S ['21489210'], distort perceptions of environmental (18 results) S ['11149907'], primary prevention (19653 results) S ['21489210'], environmental cancer (177 results) S ['21489210'], Lorenzo Tomatis (9 results) D ['17119190'], Lorenzo Tomatis (9 results) S ['21489210'], environmental cancer risks (7 results) S ['11149907'], misconceptions' distort perceptions (1 results) S ['11149907'], misconceptions' distort (1 results) S ['11149907']
JE	TRUE	6	TRUE	SOS repair phenomenology (1 results) D ['1103845'], once-commonest lethal cancer (1 results) S ['21489209'], molecular biology of bacteria (54959 results) S ['21489209'], once-commonest (1 results) S ['21489209'], contribution from biological (24081 results) S ['21489209'], stomach cancer (5599 results) S ['21489209'], lethal cancer (115 results) S ['21489209'], biological research (6850 results) S ['21489209'], Review SOS repair phenomenology (1 results) D ['1103845'], current drop (8 results) S ['21489209'], Environ Health (546 results) S ['21489209'], once-commonest lethal (1 results) S ['21489209']
JE	TRUE	2	TRUE	profiles reveals novel correlations (7 results) S ['18306933'], somatic mutations (4127 results) S ['21489208'], reveals novel correlations (48 results) S ['18306933'], somatic mutation-Darwinian selection (12 results) S ['21489208'], paroxysmal nocturnal hemoglobinuria genotype (24 results) S ['10220445'], mutation-Darwinian selection (230 results) S ['21489208'], nocturnal hemoglobinuria genotype (20 results) S ['10220445'], somatic mutation-Darwinian (16 results) S ['21489208']

PMC3073189	0	TRUE	23	FALSE		9	19	TRUE	18	TRUE	18	TRUE	13	TRU
PMC3074441	11	FALSE	1	FALSE		3	9	TRUE	8	TRUE	7	TRUE	4	TRU
PMC3074437	3	FALSE	19	FALSE		13	20	TRUE	15	TRUE	12	TRUE	9	TRU
PMC3074429	17	FALSE	2	FALSE		20	19	TRUE	17	TRUE	16	TRUE	12	TRU

JE	TRUE	9	TRUE	etheno adducts (97 results) S ['21489207', '19822158', '19037091'], cancer-prone liver (40 results) S ['21489207', '19822158', '16909291', '14742317'], adducts (21489 results) S ['21489207'], Danish mother-newborn (1 results) S ['19037091'], etheno (616 results) S ['21489207'], mother-newborn child pairs (20 results) S ['21489207', '19037091'], methods for etheno-adducts (18 results) S ['21489207', '9264272', '7697821'], study on etheno (163 results) S ['19822158', '19489076', '19037091'], cancer-prone inflammatory (19 results) S ['19822158', '17854706', '16909291'], peroxidation-derived (116 results) S ['21489207', '19822158'], Danish mother-newborn child pairs (1 results) S ['19037091'], patients with cancer-prone (292 results) S ['21489207', '19822158'], cancer-prone (812 results) S ['21489207'], Nair (8685 results) S ['21489207'], cancer-prone liver diseases (3 results) S ['21489207', '19822158', '14742317'], pilot study on etheno (3 results) S ['19822158', '19037091'], Typical signature of DNA (119 results) S ['19037091'], adduct types (13 results) S ['17854706'], Danish mother-newborn child (1 results) S ['19037091'], Bartsch (3213 results) S ['21489207'], etheno-DNA adducts (60 results) S ['21489207', '19489076', '19037091', '16909291'], etheno-DNA (78 results) S ['21489207', '19489076', '19037091', '16909291'], detection methods for etheno-adducts (14 results) S ['21489207', '9264272', '7697821']
JE	TRUE	1	TRUE	transporter-1 differential tonic (1 results) D ['12815026'], versus GABAB (33 results) D ['12815026'], GABA transporter-1 differential tonic (1 results) D ['12815026'], versus GABAB receptors (25 results) D ['12815026'], transporter-1 differential tonic activation (1 results) D ['12815026'], GABAA versus GABAB (19 results) D ['12815026'], GABAA versus GABAB receptors (17 results) D ['12815026'], Fast homeostatic plasticity (20 results) D ['18714334'], GAT-1 regulates (9 results) S ['18248614'], GABA transporter-1 differential (37 results) D ['12815026'], activity-dependent vesicular filling (5 results) D ['18714334'], activity-dependent vesicular (104 results) D ['18714334']
JE	TRUE	5	TRUE	Genoarchitectonic (3 results) S ['21344401', '19790262'], pretectal domains (4 results) S ['21344401', '18331887', '17912743'], pretectal gene expression pattern (15 results) S ['21344401', '18331887'], pretectal (1120 results) S ['21344401'], Embryonic genoarchitecture (1 results) S ['21344401'], pretectal gene (54 results) S ['21344401', '19790262', '18331887', '17912743'], pretectal gene expression (45 results) S ['21344401', '19790262', '18331887', '17912743'], chicken embryonic (19462 results) S ['21465619'], nuclear groups (177 results) S ['19790262'], Comp Neurol (133 results) S ['19790262'], anteroposterior tripartition (2 results) S ['21344401', '18331887'], early molecular regionalization (100 results) S ['18331887', '17912743'], chicken embryonic pretectum (11 results) D ['15063186', '9130671'], chicken embryonic pretectum (11 results) S ['19790262', '17912743', '11923005', '10842210'], major lineages of galliform (6 results) D ['20730714', '20444289', '10082609'], tripartition (14 results) S ['21344401', '18331887'], molecular regionalization (11 results) D ['17045408'], molecular regionalization (11 results) S ['18331887', '17912743'], pretectum (616 results) S ['21344401', '19790262'], Genoarchitectonic profile (2 results) S ['19790262'], embryonic pretectum (59 results) S ['21344401', '19790262', '17912743'], chicken pretectum (2 results) S ['19790262']
JE	TRUE	5	TRUE	nucleus tegmenti pedunculopontinus pars (15 results) D ['6738860', '6886052'], pedunculopontine tegmental nucleus (527 results) D ['21198985'], PPN neurons (45 results) D ['17331213'], PPN neurons (45 results) S ['20603194', '18440991'], pedunculopontine tegmental (799 results) D ['21198985'], tegmenti pedunculopontinus pars compacta (17 results) D ['6738860', '6886052'], PPN receives (10 results) D ['21198985', '17331213', '7284825'], non-cholinergic (2104 results) D ['21198985'], nucleus tegmenti pedunculopontinus (54 results) D ['1720145', '2471715'], tegmenti pedunculopontinus (56 results) D ['1720145', '2471715'], reciprocal electrophysiological influence (25 results) D ['2823982'], synaptic organization of pallidal (21 results) D ['9183698', '2439552'], cholinergic input from PPN (13 results) D ['9596529', '3540040'], innervate PPN neurons (4 results) D ['9596529'], PPN (581 results) D ['21198985'], tegmental nucleus (1006 results) D ['21198985'], rostral PPN (27 results) D ['8842892', '8077458', '1761086', '1707734'], rostral PPN (27 results) S ['19459217', '18440991'], antero-grade transport (2 results) D ['1281170']

PMC3069634	10	FALSE	12	FALSE		8	15	TRUE	9	TRUE	9	TRUE	7	TRU
PMC3071375	10	FALSE	6	FALSE		10	15	TRUE	8	TRUE	6	TRUE	5	TRU
PMC3071373	3	FALSE	6	FALSE		7	8	TRUE	6	TRUE	3	TRUE	3	TRU
PMC3071368	2	FALSE	4	FALSE		3	4	TRUE	3	TRUE	2	TRUE	2	TRU
PMC3071372	10	FALSE	13	FALSE		10	20	TRUE	19	TRUE	16	TRUE	14	TRU

JE	TRUE	5	TRUE	two-species microbial community (39 results) S ['17277211'], Review Host-bacterial (41 results) D ['19527880'], temporal population dynamics (13 results) S ['17277211'], symbiont (1906 results) S ['21467263'], medicinal leech (412 results) S ['21467263'], digestive-tract microbiota (4 results) S ['18689513', '16820471'], FOR DIFFERENTIALING MUCOSUBSTANCES (1 results) D ['14327695'], Review Host-bacterial mutualism (5 results) D ['19527880', '15790844'], Rikenella-like bacteria (4 results) S ['18689513', '17616592', '17277211'], FOR DIFFERENTIALING (2 results) D ['14327695'], leech (3559 results) S ['21467263'], DIFFERENTIALING MUCOSUBSTANCES (1 results) D ['14327695'], digestive-tract microbiota of Hirudo (1 results) S ['18689513'], DIFFERENTIALING MUCOSUBSTANCES HISTOCHEMICALLY (1 results) D ['14327695'], METHODS FOR DIFFERENTIALING MUCOSUBSTANCES (1 results) D ['14327695'], Hirudo (853 results) S ['21467263'], METHODS FOR DIFFERENTIALING (2 results) D ['14327695'], DIAMINE METHODS FOR DIFFERENTIALING (1 results) D ['14327695'], uncultured (2124 results) S ['21467263'], medicinal leech reveals (17 results) S ['16820471'], FOR DIFFERENTIALING MUCOSUBSTANCES HISTOCHEMICALLY (1 results) D ['14327695'], Rikenella-like (5 results) S ['21467263', '18689513', '17616592', '17277211']
JE	TRUE	2	TRUE	clonable gold (4 results) D ['19114107', '17692533'], miniSOG (1 results) S ['21483721'], clonable (192 results) D ['19114107', '17692533'], fluorescent protein paintbox (2 results) S ['19565590'], Large-scale chromatin fibers (6 results) D ['11398975'], block-face (78 results) S ['21483721'], clonable tag (3 results) D ['19114107', '17692533'], clonable gold label (2 results) D ['17692533'], freeze substitution preserves (22 results) S ['17962040'], serial block-face (14 results) D ['18617436', '15514700'], efficient protein detection (3 results) D ['17967812'], clonable tag for electron (5 results) D ['19114107', '17692533'], metallothionein tag (44 results) D ['19114107', '17967812', '17692533'], chemical fixation procedures (8 results) S ['17962040'], utero intraventricular injection (3 results) D ['18997887'], phototropin (232 results) S ['21483721']
JE	TRUE	3	TRUE	EY017081 (8 results) S ['19179278'], macaque (8855 results) S ['21483719'], cat striate cortex cells (3 results) S ['6875912'], macaque maps (3 results) S ['19179278', '10195149'], location information across object-selective (1 results) D ['18326624'], macaque temporal (3205 results) S ['21483719'], parahippocampal cortex mediates spatial (7 results) D ['16990438', '12718867'], parahippocampal (2557 results) S ['21483719'], information across object-selective (9 results) D ['18326624']
JE	TRUE	2	TRUE	ocean-scale integrity (1 results) S ['18728776'], disrupt ecosystem functioning (7 results) D ['21352458'], Extinction order (3 results) D ['21352458'], Effects of biodiversity (3008 results) S ['21483714'], ecosystem functioning (418 results) S ['21483714'], ocean-scale integrity of coral (1 results) S ['18728776']
JE	TRUE	8	TRUE	invasive pneumococcal (2029 results) S ['21483718'], pneumococcal serotypes over decades (10 results) D ['20047478'], serotype-specific attack (6 results) D ['16897668'], serotypes over decades (38 results) D ['20047478'], serotype-specific attack rates (4 results) D ['16897668'], Capsular serotype-specific (153 results) D ['20715907'], conjugate vaccine (2903 results) S ['21483718'], pneumococcal conjugate (1514 results) S ['21483718'], higher valency vaccines (8 results) S ['21483718'], descriptive epidemiology of Streptococcus (60 results) D ['18162940'], Dynamic models of pneumococcal (17 results) S ['20377886'], pneumococcal serotype replacement (111 results) D ['21031138', '21029807'], pneumococcal serotype replacement (111 results) S ['21483718'], serotype replacement (75 results) D ['21031138'], serotype replacement (75 results) S ['21483718', '20377886'], pre-conjugate vaccine England (2 results) S ['20035785', '16181510'], valency vaccines (29 results) S ['21483718'], Capsular serotype-specific attack rates (1 results) D ['16897668'], pneumococcal conjugate vaccine (1285 results) S ['21483718'], higher valency (19 results) S ['21483718'], Capsular serotype-specific attack (1 results) D ['16897668'], serotype-specific (1445 results) S ['21483718'], high CCRs (47 results) S ['21483718']

PMC3071370	1	FALSE	6	FALSE		4	5	FALSE	5	FALSE	5	TRUE	2	TRU
PMC3071369	1	FALSE	3	FALSE		2	4	FALSE	4	FALSE	4	FALSE	4	TRU
PMC3071366	5	FALSE	12	FALSE		10	17	TRUE	14	TRUE	9	TRUE	9	TRU
PMC3071374	4	FALSE	16	FALSE		6	19	TRUE	13	TRUE	11	TRUE	6	TRU
PMC3071371	4	FALSE	16	FALSE		4	20	FALSE	20	TRUE	18	TRUE	16	TRU
PMC3071367	4	FALSE	16	FALSE		4	16	TRUE	13	TRUE	12	TRUE	9	TRU

JE	TRUE	1	TRUE	whistleblowers (97 results) S ['21483716', '18765704'], off-label (2302 results) S ['21483716'], constitute off-label marketing (1 results) S ['21483716'], off-label promotion (20 results) D ['18959472'], off-label promotion (20 results) S ['19357413'], whistleblower-initiated (1 results) S ['18765704'], scope of off-label (13 results) S ['21483716']
JE	TRUE	2	TRUE	Millennium Development Goal Lancet (85 results) D ['20382417'], MDGs (190 results) S ['21483715'], reproductive rights (842 results) S ['21483715'], African framework for women's (63 results) S ['21483715']
JE	TRUE	5	TRUE	programmes fit with MRC (4 results) D ['20123834'], Wasunna (113 results) S ['21483712', '19695001', '18719161', '18495913'], inpatient paediatric care (5 results) S ['15194254'], Wagai (86 results) S ['21483712', '19695001', '19627594', '19627588'], Ayieko (9 results) S ['21483712', '19627588', '18719161'], Ntoburi (8 results) S ['21483712', '19695001', '19627594', '19627588', '18719161'], district hospitals (3277 results) S ['21483712'], health facility cluster survey (828 results) D ['21248161'], Kenyan district (34 results) S ['21483712', '19627594', '19627588', '18495913'], low-income easier (28 results) S ['18495913'], health improvement programmes fit (7 results) D ['20123834'], Wamae (54 results) S ['21483712', '21220265', '19695001', '19627594', '19627588', '18719161', '18495913'], health facility cluster (1936 results) D ['21248161'], improvement programmes fit (9 results) D ['20123834'], paediatric care (237 results) S ['21483712'], Kenyan district understanding (11 results) S ['19627594', '19627588'], Irimu (15 results) S ['21483712', '21220265', '19695001', '19627594', '19627588', '18719161', '18495913']
JE	TRUE	4	TRUE	cyclin-dependent kinase (23782 results) S ['21483720'], crk3 gene of Leishmania (7 results) D ['9851613'], crk3 gene of Leishmania (7 results) S ['11295173', '9553063'], cyclin-dependent (26634 results) S ['21483720'], Leishmania mexicana encodes (19 results) S ['11295173', '9553063'], Leishmania (16392 results) S ['21483720'], cdc2-related (155 results) S ['21483720'], mexicana encodes (20 results) S ['11295173', '9553063'], T-loop residue (17 results) S ['20338198'], CRK3 (24 results) D ['15010459'], CRK3 (24 results) S ['21483720', '20338198', '11295173'], major CRK3 (3 results) D ['9851613'], major CRK3 (3 results) S ['21483720', '20338198'], associates with p12 (13 results) S ['9553063'], CYC6 (16 results) S ['21483720'], T-loop residue Thr178 (1 results) S ['20338198'], residue Thr178 (4 results) S ['20338198'], Recombinant Leishmania mexicana (4 results) S ['20338198'], crk3 gene (4 results) D ['9851613'], crk3 gene (4 results) S ['11295173', '9553063']
JE	TRUE	14	TRUE	selectivity panel development (108 results) S ['21483717'], drug target (3386 results) S ['21483717'], human African trypanosomiasis (639 results) S ['21483717'], Trypanosoma (22276 results) S ['21483717'], kinase selectivity panel development (47 results) S ['21483717'], African trypanosomiasis (4532 results) S ['21483717'], trypanosomiasis therapy (74 results) S ['18644955'], trypanosomiasis (9306 results) S ['21483717'], Kinase-3 (16775 results) S ['21483717'], synthase kinase-3 inhibitor improves (16 results) D ['16169938'], Synthase Kinase-3 (4698 results) S ['21483717'], Glycogen Synthase Kinase-3 (4697 results) S ['21483717'], kinase selectivity panel (170 results) S ['21483717'], Glycogen synthase kinase (5613 results) S ['21483717'], synthase kinase (5643 results) S ['21483717'], biochemical kinase selectivity panel (17 results) D ['19073965'], brucei (7580 results) S ['21483717'], kinase-3 inhibitor improves glucose (9 results) D ['16169938'], High-throughput biochemical kinase selectivity (6 results) D ['19073965'], Trypanosoma brucei (7281 results) S ['21483717']
JE	TRUE	6	TRUE	whilst static-site service (1 results) S ['21483713'], trachomatous findings (172 results) S ['21483713'], eye care worker training (16 results) D ['15019339'], eye care worker training (16 results) S ['21483713'], trachomatous trichiasis (77 results) S ['21483713'], Surgery for trichiasis (340 results) S ['21483713'], static-site (8 results) S ['21483713'], Good health system management (3213 results) S ['21483713'], Bejiga (16 results) D ['15019339'], static-site facilities (1 results) S ['21483713'], whilst static-site service productivity (1 results) S ['21483713'], trachomatous (383 results) S ['21483713'], Vertical surgery campaigns (2 results) S ['21483713'], regional prevention of blindness (55 results) D ['19098034'], trichiasis (521 results) S ['21483713'], static-site service productivity (1 results) S ['21483713'], trichiasis surgeons (2 results) D ['16973662'], surgery for trachomatous (127 results) S ['21483713'], Surgeon attrition rates (2 results) S ['21483713'], Amhara (97 results) S ['21483713']

PMC3071364	1	FALSE	27	FALSE		11	20	TRUE	14	TRUE	13	TRUE	10	TRU
PMC3071363	1	FALSE	14	FALSE		5	11	TRUE	7	TRUE	6	TRUE	6	TRU
PMC3071362	0	TRUE	16	FALSE		6	16	TRUE	15	TRUE	14	TRUE	13	TRU
PMC3071361	17	FALSE	7	FALSE		16	22	TRUE	18	TRUE	16	TRUE	13	TRU

JE	TRUE	6	TRUE	helminth pathogen (10 results) S ['21483711', '20070308', '19443417', '18848453', '18296439', '14754899'], helminth pathogen Fasciola expansion (1 results) S ['18296439'], prefers proline (35 results) S ['19383516'], FhCL2 (3 results) S ['21483711', '19443417', '19383516'], pathogen Fasciola expansion (1 results) S ['18296439'], Fasciola (4126 results) S ['21483711'], FhCL3 (3 results) S ['21483711', '19443417', '19383516'], helminth pathogen Fasciola proteins (19 results) S ['19443417', '18848453', '14754899'], invasive juvenile Fasciola hepatica (5 results) S ['20374642', '19383516', '18573308'], Fasciola hepatica prefers (2 results) S ['19383516'], Fasciola hepatica prefers proline (1 results) S ['19383516'], hepatica (3736 results) S ['21483711'], parasitic liver (98 results) S ['18160404'], Fasciola hepatica (3286 results) S ['21483711'], virulence-associated (1312 results) S ['21483711'], helminth (20979 results) S ['21483711'], pathogen Fasciola (29 results) D ['20006979'], pathogen Fasciola (29 results) S ['21483711', '19443417', '18848453', '18296439', '14754899'], Opisthorchis regulation (19 results) S ['20070308'], invasive juvenile Fasciola (5 results) S ['20374642', '19383516', '18573308'], parasitic liver fluke (3 results) S ['18160404'], hepatica prefers (2 results) S ['19383516'], juvenile Fasciola hepatica prefers (1 results) S ['19383516'], Fasciola expansion (8 results) S ['18296439'], hepatica prefers proline (1 results) S ['19383516'], helminth pathogen Fasciola (4 results) S ['21483711', '19443417', '18296439', '14754899'], pathogen Fasciola proteins (19 results) S ['19443417', '18848453', '14754899'], cathepsin (14477 results) S ['21483711']
JE	TRUE	5	TRUE	transporter SGTP4 (7 results) S ['20532163', '8622989'], intramammalian-stage Schistosoma mansoni (1 results) S ['8622989'], schistosomes impairs parasite (1 results) S ['20532163'], tegument (2099 results) S ['21483710'], schistosomes (1682 results) S ['21483710'], schistosomes impairs (2 results) S ['20532163'], apical surface of intramammalian-stage (1 results) S ['8622989'], Skelly (657 results) S ['21483710'], intramammalian-stage (2 results) S ['8622989'], tegumental (904 results) S ['21483710'], surface of intramammalian-stage (1 results) S ['8622989'], outer membrane of schistosomula (30 results) D ['12878186'], intramammalian-stage Schistosoma (2 results) S ['8622989'], adult Schistosoma mansoni (279 results) S ['17304823'], glucose transporter SGTP4 (6 results) S ['20532163', '8622989']
JE	TRUE	8	TRUE	hospital care during floods (22 results) S ['18981509'], Diarrheal epidemics (7 results) S ['18981509', '16760521'], care during floods (65 results) S ['18981509'], 2-year period from diarrheal (20 results) S ['17548483', '10618058'], major diarrheal pathogens (3 results) S ['18981509'], enterotoxigenic Escherichia coli (2054 results) S ['21483709'], major diarrheal (530 results) S ['21483709'], enterotoxigenic Escherichia (2067 results) S ['21483709'], coli with STh (58 results) S ['16943355'], STp genotypes (24 results) S ['16943355'], cholerae (8944 results) S ['21483709'], Dhaka (2855 results) S ['21483709'], enterotoxigenic (3530 results) S ['21483709'], diarrheal patients (128 results) S ['21483709'], diarrheal (5016 results) S ['21483709'], Escherichia coli with STh (51 results) S ['16943355']
JE	TRUE	6	TRUE	Entamoeba histolytica lectin (6 results) D ['9693367'], Entamoeba suggestive evidence (4 results) D ['9317033'], multiple CXXC (20 results) D ['11500468'], motile pathogenic role (31 results) D ['15617520', '10884615'], multiple CXXC sequence (14 results) D ['11500468'], immuno-dominant variable surface (3 results) D ['1696956'], Entamoeba histolytica (6082 results) S ['21483708'], histolytica lectin (12 results) D ['9693367'], Entamoeba (7009 results) S ['21483708'], uropod formation (39 results) D ['15504914', '10092792'], uropod formation (39 results) S ['21483708'], lectin of Entamoeba (357 results) D ['20581296', '20083116', '19936071'], lectin of Entamoeba (357 results) S ['21483708'], multiple CXXC sequence motifs (6 results) D ['11500468'], overexpression of EhGEF1 (1 results) D ['15713446'], immuno-dominant variable surface antigen (1 results) D ['1696956'], EhGEF1 (2 results) D ['15713446'], uropods (100 results) D ['19373240'], uropods (100 results) S ['21483708'], Entamoeba histolytica involves (34 results) D ['15287584'], histolytica involves (34 results) D ['15287584'], histolytica (6482 results) S ['21483708'], Almaraz-Barrera (6 results) D ['17323375', '15713446'], uropod (285 results) S ['21483708']

PMC3071750	4	FALSE	15	FALSE		7	17	TRUE	8	TRUE	8	TRUE	6	TRU
PMC3071737	4	FALSE	10	FALSE		6	14	TRUE	10	TRUE	7	TRUE	7	TRU
PMC3071734	9	FALSE	12	FALSE		5	21	TRUE	19	TRUE	14	TRUE	14	TRU
PMC3071732	6	FALSE	14	FALSE		5	19	TRUE	17	TRUE	17	TRUE	16	TRU
PMC3071731	4	FALSE	6	FALSE		4	9	TRUE	7	TRUE	7	TRUE	5	TRU

JE	TRUE	3	TRUE	cross-race risk for development (2 results) S ['18048766'], causal relationship between Lp-PLA (1 results) S ['21490708'], factor acetylhydrolase deficiency (5 results) D ['10051281', '8675689'], PLA2G7 (33 results) D ['20479152', '19034521'], PLA2G7 (33 results) S ['21490708'], PLA2G7 279F (2 results) S ['21490708'], PLA2G7 gene polymorphisms (13 results) D ['19034521'], Korean population implicate (26 results) S ['18048766'], cross-race risk (2 results) S ['18048766'], lipoprotein-associated (1791 results) S ['21490708'], Lp-PLA (146 results) S ['21490708'], confers high cross-race (2 results) S ['18048766'], South Korean population implicate (1 results) S ['18048766'], phospholipase (39868 results) S ['21490708'], high cross-race (5 results) S ['18048766'], Val279Phe variant (2 results) S ['16787988'], Associations of PLA2G7 (18 results) D ['20479152', '19034521'], high cross-race risk (2 results) S ['18048766'], confers high cross-race risk (2 results) S ['18048766']
JE	TRUE	3	TRUE	polymorphisms of beta3-adrenergic (55 results) S ['17439327'], northern Han (91 results) S ['21483652'], Chinese male hypertensive patients (2 results) S ['17439327'], A46G (7 results) S ['21483652', '20739939'], beta2-adrenergic (1508 results) S ['21483652'], beta2-AR Gln27Glu (4 results) S ['17439327'], beta2-adrenergic receptor (916 results) S ['21483652'], Chinese male hypertensive (2 results) S ['17439327'], Minority Health Summit report (7 results) D ['15769755'], essential hypertension (19178 results) S ['21483652'], cardiovascular Minority Health Summit (5 results) D ['15769755'], beta2-Adrenergic receptor gene (97 results) S ['21483652', '20739939'], Review beta2-Adrenergic receptor gene (32 results) D ['15638826'], beta2-Adrenergic receptor gene variations (11 results) D ['15638826', '10872552']
JE	TRUE	10	TRUE	nutlin-3a treatment (10 results) D ['17545546'], glioblastoma cells (1845 results) S ['21483692'], p53 glioma (1791 results) S ['21483692'], p53-murine double minute interaction (5 results) D ['17545546', '16081689'], wild-type p53 glioma cell (359 results) S ['21483692'], p53-dependent (3672 results) S ['21483692'], p53 glioma cell (1459 results) S ['21483692'], Inhibition of p53-murine (4 results) D ['17545546'], MDM2 (4567 results) S ['21483692'], glioma cell lines (1764 results) S ['21483692'], p53-murine double (8 results) D ['17545546', '16081689'], nutlin-3A stabilizes p53 (8 results) D ['17545546'], MDM2 antagonists (33 results) D ['17440969'], MDM2 antagonists (33 results) S ['21483692'], mTOR pathway (1118 results) S ['21483692'], glioma cells (6978 results) S ['21483692'], nutlin-3a (62 results) D ['20606252'], nutlin-3a (62 results) S ['21483692'], nutlin-3A stabilizes (8 results) D ['17545546'], p53-murine double minute (8 results) D ['17545546', '16081689'], p53 glioma cell lines (642 results) S ['21483692']
JE	TRUE	11	TRUE	ababa tuberculosis centre (18 results) D ['11440221'], negative sputum (265 results) S ['21483690'], clinical-radiographic score (36 results) S ['21483690'], diagnosis of smear-negative (757 results) S ['21483690'], acid fast bacilli represent (31 results) S ['21483690'], addis ababa tuberculosis (3 results) D ['11440221'], smear-negative pulmonary tuberculosis (121 results) S ['21483690'], pulmonary tuberculosis (66870 results) S ['21483690'], bacilli smears (23 results) D ['10206504'], Maynart-Badiane (1 results) D ['10206504'], smear-negative (961 results) S ['21483690'], Review Diagnosis of smear-negative (81 results) D ['17805454', '17574096'], negative sputum acid-fast bacilli (450 results) S ['21483690'], bacilli (18499 results) S ['21483690'], sputum acid-fast bacilli smears (300 results) S ['21483690'], fast bacilli represent (41 results) S ['21483690'], probability of smear-negative (103 results) S ['21483690'], sputum smears (423 results) S ['21483690'], smear-negative pulmonary (149 results) S ['21483690'], negative sputum acid-fast (2 results) D ['10206504']
JE	TRUE	5	TRUE	simian-human (504 results) S ['21483689'], simian-human immunodeficiency virus (451 results) S ['21483689'], gp120 (7990 results) S ['21483689'], envelope glycoprotein (4489 results) S ['21483689'], simian-human immunodeficiency (493 results) S ['21483689'], HIV-1gp120 (17 results) D ['16199516'], HIV-1gp120 (17 results) S ['21483689'], type gp120 induces abnormal (2 results) D ['15331709'], gp120 induces abnormal maturation (1 results) D ['15331709'], gp120 induces abnormal (4 results) D ['19208751', '15331709']

PMC3071730	5	FALSE	8	FALSE		6	12	TRUE	11	TRUE	9	TRUE	6	TRU
PMC3071729	3	FALSE	4	FALSE		5	6	FALSE	6	TRUE	5	TRUE	5	TRU
PMC3071728	2	FALSE	5	FALSE		6	7	TRUE	4	TRUE	4	TRUE	2	TRU
PMC3071726	10	FALSE	6	FALSE		8	11	TRUE	7	TRUE	6	TRUE	6	TRU
PMC3071724	3	FALSE	27	FALSE		4	22	TRUE	14	TRUE	13	TRUE	12	TRU

JE	TRUE	6	TRUE	Acids Res Identification (139 results) D ['19854910'], global identification of sRNAs (7 results) D ['19854910'], Small RNAs (2073 results) S ['21483688'], AE005674 AE005674 (3 results) S ['12384590'], Shigella genome (7 results) S ['21483688', '16939645'], sRNAs (330 results) S ['21483688'], sORF-specific (11 results) D ['16510898'], small open reading frames (108 results) D ['16510898'], small open reading frames (108 results) S ['21483688'], sORFs (19 results) D ['16510898'], sORFs (19 results) S ['21483688'], Sf301 chromosome (2 results) S ['21483688', '16822325'], Shigella (13441 results) S ['21483688']
JE	TRUE	4	TRUE	HIV latency activation (398 results) D ['20585398'], HIV latency activation (398 results) S ['21483687'], stimulate latent HIV (31 results) S ['21483687', '14569007'], latent HIV (252 results) S ['21483687'], Immuno-activation with anti-CD3 (1 results) D ['10597782'], Stavudine-loaded (7 results) D ['16640829'], bryostatin (693 results) S ['21483687']
JE	TRUE	2	TRUE	AKAP12 isoforms with distinct (4 results) S ['15590635', '15496411'], AKAP12 (127 results) D ['20979053'], AKAP12 (127 results) S ['21483686'], novel retinoid-response (14 results) S ['11181072'], AKAP12 isoforms (3 results) D ['15258566'], AKAP12 isoforms (3 results) S ['15590635', '15496411'], novel retinoid-response gene (11 results) S ['11181072']
JE	TRUE	3	TRUE	acid triggers curative Th1 (1 results) D ['16002718'], experimental visceral leishmaniasis (167 results) S ['20403680'], Beta-glycyrrhetinic acid triggers (8 results) D ['16002718'], benzodioxoloquinolizine alkaloid (2 results) D ['12807487', '12034375'], Quassin alters (1 results) S ['19036753'], Berberine chloride (59 results) S ['21483684', '19669998'], Berberine (2435 results) S ['21483684'], triggers curative Th1 (2 results) D ['16002718'], acid triggers curative (5 results) D ['16002718'], visceral defective activation (28 results) D ['11179319'], benzodioxoloquinolizine (2 results) D ['12807487', '12034375'], Berberine chloride causes (27 results) S ['19669998'], curative Th1 response (4 results) D ['16002718'], Beta-glycyrrhetinic acid triggers curative (1 results) D ['16002718'], macrophages through generation (5849 results) S ['21483684'], triggers curative Th1 response (2 results) D ['16002718']
JE	TRUE	6	TRUE	conduction inhomogeneity (152 results) S ['21483682'], simulation domain (19 results) S ['21483682'], inhomogeneities on scroll-wave (2 results) S ['21483682', '19270753'], Scroll-wave dynamics (4 results) D ['10827961'], Scroll-wave dynamics (4 results) S ['21483682', '19270753'], scroll-wave filament (9 results) S ['21483682'], conduction inhomogeneity of radius (2 results) S ['21483682'], fiber rotation (50 results) S ['21483682'], absence of inhomogeneities (48 results) S ['19270753'], TNNP (4 results) D ['18495166'], TNNP (4 results) S ['21483682', '19270753'], scroll wave (54 results) S ['21483682', '19270753'], cylindrical ionic (208 results) S ['21483682'], ionic inhomogeneity of radius (1 results) S ['21483682'], TNNP model (4 results) D ['18495166'], TNNP model (4 results) S ['21483682', '19270753'], inhomogeneity (3968 results) S ['21483682'], inhomogeneity of radius (39 results) S ['21483682'], scroll-wave filament exhibits (1 results) S ['21483682'], cylindrical (14220 results) S ['21483682'], cylindrical conduction (130 results) S ['21483682'], transmural (10690 results) S ['21483682'], displays strong meandering (1 results) S ['21483682'], Scroll-wave break-up (1 results) S ['21483682'], cylindrical conduction inhomogeneity (4 results) S ['21483682'], scroll-wave (54 results) S ['21483682', '19270753'], scroll-wave filament exhibits weak (1 results) S ['21483682'], filament exhibits weak meandering (1 results) S ['21483682'], ionic inhomogeneity (39 results) S ['21483682'], cylindrical ionic inhomogeneity (1 results) S ['21483682']

PMC3071723	9	FALSE	7	FALSE		7	15	TRUE	9	TRUE	8	TRUE	7	TRU
PMC3071722	6	FALSE	10	FALSE		6	16	TRUE	15	TRUE	14	TRUE	10	TRU
PMC3071721	3	FALSE	10	FALSE		8	12	TRUE	11	TRUE	10	TRUE	9	TRU
PMC3071720	2	FALSE	20	FALSE		2	16	TRUE	13	TRUE	13	TRUE	8	TRU
PMC3071719	6	FALSE	0	TRUE		2	6	FALSE	6	FALSE	6	FALSE	6	TRU
PMC3071718	5	FALSE	5	FALSE		8	9	TRUE	8	TRUE	8	TRUE	6	TRU

JE	TRUE	4	TRUE	Xiphophorus (558 results) S ['21483681'], Blast2GO (21 results) D ['18445632'], marine shrimp Marsupenaeus japonicus (23 results) S ['17462931'], Xiphophorus models (284 results) S ['21483681'], High-throughput functional annotation (3 results) D ['18445632'], vitellogenin locus (3 results) D ['10905350'], maculatus (858 results) S ['21483681'], cancer identifies peroxisome (11 results) D ['15299082'], gene X-src (2 results) D ['8290610'], marine shrimp Marsupenaeus (24 results) S ['17462931'], endometrial cancer identifies peroxisome (1 results) D ['15299082'], Blast2GO suite (3 results) D ['18445632'], maculatus transcriptome (9 results) S ['21483681', '19049829'], kinase gene X-src (2 results) D ['8290610'], contigs (1702 results) S ['21483681'], tyrosine kinase gene X-src (2 results) D ['8290610']
JE	TRUE	9	TRUE	amyloid fibril formation (681 results) S ['21483680'], denatured (13797 results) S ['21483680'], Lysozyme Amyloid (238 results) S ['21483680'], charge repulsion (254 results) S ['21483680'], amyloidogenic (2591 results) S ['21483680'], Lysozyme Amyloid Oligomers (13 results) D ['17134716'], Lysozyme Amyloid Oligomers (13 results) S ['19413984'], amyloid fibril (1567 results) S ['21483680'], fibril formation (2080 results) S ['21483680'], amyloidogenic behavior (5 results) D ['10350460'], Fibrils Induce Cellular Death (34 results) D ['17134716'], lysozyme (18613 results) S ['21483680'], neutron comparison of lysozyme (12 results) D ['9826592'], disease-related variants (10 results) D ['10350460'], Disease-Related Mutational Variants (19 results) D ['10940237'], fibril (6512 results) S ['21483680']
JE	TRUE	6	TRUE	song sparrow hypothalamus (15 results) D ['19997634'], dominance hierarchies (159 results) S ['21483679'], subdominant with dominant (263 results) S ['21483679'], hybridisation library from dominance (10 results) S ['16052453'], ependymin expression (1 results) D ['10508924'], hierarchies of rainbow (626 results) S ['21483679'], rainbow trout (7566 results) S ['21483679'], subdominant (538 results) S ['21483679'], dominance hierarchies of rainbow (35 results) S ['21483679', '16052453'], sub-dominant individuals (3 results) S ['21483679'], transcript profiles with gene (1670 results) S ['21483679'], ependymin (50 results) D ['19322784', '17302986', '10508924', '9858266'], ependymin (50 results) S ['21483679', '10235674']
JE	TRUE	8	TRUE	other Malay (353 results) S ['21483678'], Malay sub-ethnic groups (1 results) S ['21483678'], Melayu Minang (1 results) S ['21483678'], silk mtDNA sequences (11 results) D ['9837835'], Kelantan (1033 results) S ['21483678'], Peninsular Malaysia (539 results) S ['21483678'], sub-ethnic (12 results) S ['21483678'], other Malay sub-ethnic (1 results) S ['21483678'], other Malay sub-ethnic groups (1 results) S ['21483678'], Malay populations (14 results) S ['21483678'], other Malay populations (202 results) S ['21483678'], Melayu Kelantan (2 results) S ['21483678'], genotype data from Indonesia (139 results) S ['21483678'], Malay sub-ethnic (1 results) S ['21483678'], silk mtDNA (16 results) D ['9837835'], Universiti (4503 results) S ['21483678'], Melayu Jawa (1 results) S ['21483678'], other populations' genotype data (2 results) S ['21483678'], genetic structure (4680 results) S ['21483678'], sub-ethnic groups (4 results) S ['21483678'], genetic relationship with Indonesian (17 results) S ['21483678'], genotype data (1116 results) S ['21483678']
JE	TRUE	4	TRUE	MMWR Recomm Rep (367 results) D ['20689501'], MMWR (837 results) D ['20689501'], monovalent recommendations (46 results) D ['20689501'], March Euro Surveill (86 results) D ['20504388'], Immunization Practices MMWR (284 results) D ['20689501'], Practices MMWR Recomm Rep (125 results) D ['20689501']
JE	TRUE	4	TRUE	perforin (3829 results) S ['21483676'], CD4-independent proliferation of CD8 (5 results) S ['15872023'], Long-term immunovirologic control (1 results) D ['20549847'], polyfunctional antigen-specific CD8 (22 results) D ['20221423'], Distinct profiles of cytotoxic (128 results) S ['19176626'], perforin expression (271 results) D ['20523897'], perforin expression (271 results) S ['21483676'], polyfunctional antigen-specific (34 results) D ['20221423'], association of polyfunctional (11 results) D ['18200635'], association of polyfunctional (11 results) S ['17911249']

PMC3071717	12	FALSE	3	FALSE		7	13	TRUE	10	TRUE	8	TRUE	7	TRU
PMC3071716	0	TRUE	3	FALSE		1	3	FALSE	3	FALSE	3	FALSE	3	FAL
PMC3071710	6	FALSE	9	FALSE		7	14	TRUE	11	TRUE	11	TRUE	5	TRU
PMC3071703	2	FALSE	11	FALSE		4	12	TRUE	10	TRUE	9	TRUE	7	TRU
PMC3071699	0	TRUE	4	FALSE		1	4	FALSE	4	FALSE	4	FALSE	4	FAL
PMC3071698	2	FALSE	17	FALSE		4	18	TRUE	17	TRUE	14	TRUE	10	TRU
PMC3071697	3	FALSE	12	FALSE		4	15	TRUE	12	TRUE	12	TRUE	12	TRU

JE	TRUE	4	TRUE	anti-ampicillin single-chain (1 results) D ['11397088'], amyloid pathogenetic mechanisms (102 results) D ['18184371'], stable single-chain framework (26 results) D ['12215438'], x-ray structure of anti-ampicillin (1 results) D ['11397088'], interferon beta bypasses (6 results) D ['15145605'], growth factor rescues recognition (10 results) D ['15728733'], Intranasal (17157 results) S ['21483675'], beta-amyloid (8611 results) S ['21483675'], Review Cerebral amyloid pathogenetic (71 results) D ['18184371'], factor rescues recognition (16 results) D ['15728733'], single-chain framework (3 results) D ['12215438'], intrabody libraries (4 results) D ['12215438'], orexin-A reduces (32 results) D ['18160631'], structure of anti-ampicillin (3 results) D ['11397088'], single-chain (7542 results) S ['21483675']
.SE	TRUE	3	TRUE	synaptic transmission (50534 results) S ['21483674'], colliculus (8303 results) S ['21483674'], superior colliculus (5182 results) S ['21483674']
JE	TRUE	5	TRUE	dendrotoxin homologues on subtypes (3 results) D ['8612784'], Functional Effects of Channels (7734 results) S ['21483673'], islets (39310 results) S ['21483673'], pancreatic beta role (6075 results) S ['21483673'], hybridization reveal early disturbances (3 results) S ['14686896'], voltage-dependent (17179 results) S ['21483673'], Novel effects of dendrotoxin (18 results) D ['8612784'], Kv2 voltage-dependent channel families (12 results) D ['11463864'], causes megencephaly (14 results) D ['8995755'], causes megencephaly (14 results) S ['14686897', '14686896'], megencephaly (17 results) D ['8995755'], megencephaly (17 results) S ['21483673', '14686897', '14686896'], megencephalic mouse (1 results) S ['14686896'], islet electrical activity (5 results) D ['17767909'], glucose-induced (5396 results) S ['21483673']
JE	TRUE	6	TRUE	pneumococcal pneumonia (4079 results) S ['21483672'], non-lethal intranasal (14 results) D ['19467320'], chemotoxic stress (6 results) S ['21483672'], host defense (11551 results) S ['21483672'], chemotoxic (68 results) S ['21483672'], kinome profiles (4 results) S ['17178854'], Substrate Phosphorylation of c-Raf (132 results) S ['19649278'], non-lethal intranasal challenges (1 results) D ['19467320'], CDK activity (508 results) S ['21483672'], Comparison of kinome (16 results) S ['17178854'], CDK (5613 results) S ['21483672'], kinome profiles of Barrett's (2 results) S ['17178854'], homogenates (28477 results) S ['21483672']
.SE	TRUE	4	TRUE	GOIs (120 results) S ['21483670'], Bladder Cancer pathway (774 results) S ['21483670'], urinary bladder cancer (935 results) S ['21483670'], KEGG molecular pathway (153 results) S ['21483670']
JE	TRUE	7	TRUE	PV-infected (39 results) S ['21483669'], findings potentiate (5088 results) S ['21483669'], decoy receptors (594 results) S ['21483669'], JNK phosphorylation (621 results) S ['21483669'], virus type originates (81 results) D ['19390619'], MDM (1222 results) S ['21483669'], agonistic anti-DR5 antibody (17 results) S ['21483669'], MDM cells (6 results) S ['21483669'], intracellular c-FLIP (239 results) S ['21483669'], immunodeficiency virus type originates (12 results) D ['19390619'], potentiate clinical usage (11 results) S ['21483669'], TRAIL AD5-10 (8 results) S ['21483669', '19468286', '16234248'], TRAIL decoy receptors (23 results) S ['21483669'], c-FLIP (541 results) S ['21483669'], agonistic anti-DR5 (17 results) S ['21483669'], findings potentiate clinical usage (5 results) S ['21483669'], findings potentiate clinical (896 results) S ['21483669'], tumoricidal activity induces caspase-dependent (1 results) S ['16234248'], AD5-10 (12 results) S ['21483669', '19468286', '16234248']
JE	TRUE	12	TRUE	size associations with rectal (153 results) D ['20383576'], methylator (352 results) S ['21483668'], CpG island (3067 results) S ['21483668'], non-CIMP tumors (4 results) D ['17096326', '16407376'], non-CIMP tumors (4 results) S ['21483668'], island methylator (296 results) S ['21483668'], rectal tumor mutations (2 results) D ['20383576'], CpG island methylator phenotype (293 results) S ['21483668'], methylator phenotype (321 results) S ['21483668'], associations with CpG (1102 results) S ['21483668'], CpG island methylator (295 results) S ['21483668'], body size (13557 results) S ['21483668'], methylator phenotype of colorectal (216 results) S ['21483668'], island methylator phenotype (293 results) S ['21483668'], body size associations (24183 results) S ['21483668']

PMC3071695	13	FALSE	6	FALSE		7	17	TRUE	13	TRUE	10	TRUE	6	TRU
PMC3071679	1	FALSE	8	FALSE		2	9	TRUE	8	TRUE	7	TRUE	5	TRU
PMC3071676	17	FALSE	18	FALSE		17	35	TRUE	29	TRUE	25	TRUE	22	TRU
PMC3073956	0	TRUE	12	FALSE		1	12	FALSE	12	FALSE	12	FALSE	12	TRU
PMC3078886	0	TRUE	14	FALSE		1	11	TRUE	10	TRUE	10	TRUE	7	TRU

JE	TRUE	5	TRUE	temporal lobe sources (9 results) D ['12866829'], clinical Guidelines for eliciting (64 results) D ['19796989'], administration ofACTH (2 results) D ['10420094'], subchronic intranasal administration ofACTH (1 results) D ['10420094'], intranasal administration ofACTH (1 results) D ['10420094'], stress enhances glutamatergic transmission (8 results) D ['19666502'], tentative clue for this (12 results) S ['21483666'], Acute stress enhances glutamatergic (4 results) D ['19666502'], attention after acute (3010 results) S ['21483666'], subchronic intranasal (15 results) D ['10420094'], stress impairs selective attention (6 results) D ['12899196'], lobe sources (11 results) D ['12866829'], selective attention (3146 results) S ['21483666'], stressor (6997 results) S ['21483666'], ofACTH (2 results) D ['10420094'], stress enhances glutamatergic (17 results) D ['19666502', '2119850'], imbalance on selective (604 results) S ['21483666'], subchronic intranasal administration (4 results) D ['10420094'], Acute stress (3553 results) S ['21483666']
JE	TRUE	3	TRUE	frequent geographical locations (44 results) S ['21483665'], geographical span (4 results) S ['21483665'], community detection algorithm (6 results) S ['21483665'], nodes within communities (146 results) D ['20562860'], individuals within network (3892 results) S ['21483665'], topological positions (13 results) S ['21483665'], network communities (13 results) S ['21483665'], such geographic constraints (342 results) S ['21483665'], geographic positions of individuals (29 results) S ['21483665']
JE	TRUE	6	TRUE	Hth haltere (3 results) D ['12070087'], Hth haltere (3 results) S ['21483663'], Hth (471 results) S ['21483663'], Ultrabithorax (405 results) S ['21483663', '19730678'], Hth leg (20 results) S ['21483663'], Drosophila leg (69 results) D ['16631729'], Drosophila leg (69 results) S ['20585625', '18194655'], correlation between Ubx (9 results) D ['19651300'], Hox (3678 results) S ['21483663'], haltere imaginal discs (8 results) D ['17174297'], late expression of Bar (51 results) D ['16631729'], Ubx haltere (42 results) D ['17855427', '17174297', '17050628', '17045257', '16414040', '15753212'], Ubx haltere (42 results) S ['21483663', '17166918', '16741075'], haltere Hth (3 results) D ['12070087'], haltere Hth (3 results) S ['21483663'], Ultrabithorax regulates (29 results) D ['17050628', '12070087', '9585507'], Ultrabithorax regulates (29 results) S ['21483663'], Ubx leg (28 results) S ['21483663'], haltere imaginal (39 results) D ['17174297'], haltere imaginal (39 results) S ['21483663'], Ubx regulates (37 results) D ['17050628'], Ubx regulates (37 results) S ['21483663'], mammalian dioxin receptor (4 results) D ['16631729', '9573046'], Ultrabithorax regulates genes (25 results) D ['12070087', '9585507'], Ultrabithorax regulates genes (25 results) S ['21483663'], haltere Ubx (42 results) D ['17855427', '17174297', '17050628', '17045257', '16414040', '15753212'], haltere Ubx (42 results) S ['21483663', '17166918', '16741075'], Drosophila haltere (4 results) D ['9585507'], haltere (107 results) D ['17855427', '17174297', '17050628'], haltere (107 results) S ['21483663', '17166918'], Ubx Hth (10 results) D ['12070087'], Ubx Hth (10 results) S ['21483663'], Ubx (536 results) S ['21483663'], Drosophila leg development (16 results) D ['16631729'], Drosophila leg development (16 results) S ['20585625', '18194655']
JE	TRUE	10	TRUE	in-service training of professionals (1816 results) S ['21463506'], training programme (2819 results) S ['21463506'], Delphi technique (2156 results) S ['21463506'], primary care of allergy (3144 results) S ['21463506'], allergy patients (240 results) S ['21463506'], field of allergy (2719 results) S ['21463506'], primary care providers (2434 results) S ['21463506'], practice amongst primary (423 results) S ['21463506'], good practice amongst primary (28 results) S ['21463506'], practice amongst primary care (357 results) S ['21463506'], core competencies (701 results) S ['21463506'], amongst primary care providers (52 results) S ['21463506']
JE	TRUE	3	TRUE	association between SES (1058 results) S ['21463527'], socio-economic characteristics (423 results) S ['21463527'], high-duration walkers (1 results) S ['21463527'], duration walkers (47 results) S ['21463527'], high duration (16 results) S ['21463527'], urban-regional variations (1 results) S ['21463527'], high-duration (16 results) S ['21463527'], normal weight range (59 results) S ['21463527'], lower-duration (59 results) S ['21463527'], urban-regional (32 results) S ['21463527'], lower-duration walkers (1 results) S ['21463527'], non-walkers (20 results) S ['21463527'], strong association between SES (104 results) S ['21463527'], high duration walkers (3 results) S ['21463527']

PMC3076276	0	TRUE	20	FALSE		4	15	TRUE	10	TRUE	9	TRUE	6	TRU
PMC3078868	1	FALSE	22	FALSE		4	23	FALSE	23	TRUE	22	TRUE	21	TRU
PMC3078867	0	TRUE	12	FALSE		1	8	FALSE	8	TRUE	7	TRUE	7	TRU
PMC3070432	2	FALSE	1	FALSE		3	3	TRUE	2	TRUE	2	TRUE	1	TRU
PMC3076224	0	TRUE	22	FALSE		3	22	TRUE	18	TRUE	16	TRUE	14	TRU
PMC3073059	5	FALSE	0	TRUE		4	3	TRUE	1	TRUE	1	TRUE	1	TRU

JE	TRUE	6	TRUE	Kate Westgate (2 results) S ['21463520'], behaviour change (1182 results) S ['21463520'], domiciliary intervention (4 results) S ['15491494'], Imogen Hobbis (2 results) S ['21463520'], intensive treatment (3035 results) S ['21463520'], intensive multifactorial intervention (11 results) S ['11063279'], Facilitators taught patients (5 results) S ['21463520'], medication adherence (4083 results) S ['21463520'], multiple behaviours (15 results) S ['21463520'], intensive multifactorial (515 results) S ['21463520'], Sue Boase (2 results) S ['21463520'], ADDITION-Plus trial (1 results) S ['21463520'], behavioural interventions (482 results) S ['21463520'], ADDITION-Plus (1 results) S ['21463520'], Kinmonth (264 results) S ['21463520'], facilitator-led (14 results) S ['21463520'], Roslyn Barling (1 results) S ['19435491'], domiciliary intervention programme (9 results) S ['15491494'], one-hour introductory meeting (1 results) S ['21463520'], Fiona Whittle (1 results) S ['21463520']
JE	TRUE	18	TRUE	control with exhaustion (2342 results) S ['21463503'], whereas job control (286 results) S ['21463503'], emotional exhaustion (750 results) S ['21463503'], Psychosocial work environment (293 results) S ['21463503'], exhaustion among women (3672 results) S ['21463503'], job control (486 results) S ['21463503'], work factors (251 results) S ['21463503'], psychosocial factors (6844 results) S ['21463503'], factors with exhaustion (2733 results) S ['21463503'], Karasek's job control (72 results) S ['21463503', '18440254'], physical work (1944 results) S ['21463503'], job control with exhaustion (160 results) S ['21463503'], Karasek's (122 results) S ['21463503'], physical work factors (17 results) S ['21463503'], psychosocial work (596 results) S ['21463503'], psychosocial factors with exhaustion (1337 results) S ['21463503'], relational justice (11 results) D ['17905053', '15483310'], occupational class (320 results) S ['21463503'], organizational justice (93 results) S ['21463503'], men job control (3683 results) S ['21463503'], work factors with emotional (3355 results) S ['21463503'], associations with exhaustion (463 results) S ['21463503'], psychosocial work factors (64 results) S ['21463503']
JE	TRUE	7	TRUE	Service Brabant Zuidoost (1 results) S ['21463499'], Health Service Brabant Zuidoost (1 results) S ['21463499'], Health Service Brabant-Zuidoost (1 results) S ['21463499'], association between binge (549 results) S ['21463499'], harmful way (9 results) S ['21463499'], binge drinking (1618 results) S ['21463499'], report mental health problems (1934 results) S ['21463499'], poor mental health (572 results) S ['21463499'], drinkers (8627 results) S ['21463499'], adolescents report anxiety (2599 results) S ['21463499'], Service Brabant-Zuidoost (1 results) S ['21463499'], binge drinkers (262 results) S ['21463499']
JE	TRUE	1	TRUE	retrospective single centre analysis (14 results) D ['17245696'], doctor-patient communication about imminent (3 results) D ['11099281'], further treatment (5681 results) S ['21464103']
JE	TRUE	8	TRUE	experimental responses (65 results) S ['21463509'], American palm (119 results) S ['21463509'], plant odor (21 results) S ['21463509'], Rhynchophorus behavioral (3 results) S ['16256688', '16222808'], Interactions between acetoin (15 results) S ['16222808'], Rhynchophorus (29 results) S ['16256688', '16222808'], palmarum (45 results) S ['16256688', '16222808'], aggregation pheromone (158 results) S ['21463509'], host plant odors (10 results) S ['21463509', '16256688'], synergy between aggregation (120 results) S ['21463509'], maximum mixture response (565 results) S ['21463509'], olfactometer (550 results) S ['21463509'], weevil (810 results) S ['21463509'], olfactory synergy (18 results) S ['21463509', '16222808'], olfactory synergistic (74 results) S ['21463509'], palm weevil (23 results) S ['21463509', '16256688'], response thresholds (543 results) S ['21463509'], pheromone (6032 results) S ['21463509'], American palm weevil (2 results) S ['21463509'], 4-arm olfactometer (2 results) S ['21463509'], Rhynchophorus palmarum (10 results) S ['16256688', '16222808'], palmarum for behavioral (3 results) S ['16256688', '16222808']
JE	TRUE	0	FALSE	altitude sustain cerebral (2 results) D ['12062213'], functions of NSCs (81 results) D ['20852629'], cerebral oxygen delivery Severinghaus (3 results) D ['12062213', '11258585'], delivery Severinghaus revisited (1 results) D ['12062213'], hydroxylates Notch ICD (1 results) D ['18299578']

PMC3074506	0	TRUE	5	FALSE		7	5	TRUE	3	TRUE	2	TRUE	1	TRU
PMC3071874	2	FALSE	13	FALSE		3	15	TRUE	12	TRUE	11	TRUE	8	TRU
PMC3071870	1	FALSE	16	FALSE		3	17	TRUE	16	TRUE	15	TRUE	14	TRU
PMC3070740	4	FALSE	8	FALSE		9	10	TRUE	6	TRUE	5	TRUE	4	TRU
PMC3070738	4	FALSE	20	FALSE		2	23	FALSE	23	TRUE	21	TRUE	16	TRU

JE	TRUE	0	FALSE	neuron depolarization via H2O2 (4 results) S ['16251452'], dopamine neurons via ATP-sensitive (11 results) S ['19605638', '18632893', '17115944', '16251452', '15858048'], neurons via ATP-sensitive (82 results) S ['19605638', '18632893'], Glutamate-dependent inhibition (3 results) S ['13679582', '12684460'], depolarization via H2O2 (18 results) S ['16251452']
JE	TRUE	5	TRUE	fellows' satisfaction (14 results) S ['19638779'], medical BEME (37 results) D ['17074699'], fellowship programme (28 results) S ['21475643'], faculty development (1339 results) S ['21475643'], programme evaluation data (5 results) S ['21475643'], faculty development programme (14 results) S ['21475643'], completion requirements (5 results) S ['21475643'], better outcome data (7 results) S ['21475643'], programme evaluation (219 results) S ['21475643'], educational skills of faculty (1920 results) S ['21475643'], graduation requirements (26 results) S ['21475643'], medical BEME Guide (11 results) D ['17074699'], medical education fellowships (2180 results) S ['21475643'], evaluation methods (1674 results) S ['21475643'], education fellowships (4 results) S ['21475643']
JE	TRUE	11	TRUE	discipline-specific (235 results) S ['21475642'], discipline-specific examinations (4 results) S ['21475642'], PISCES-specific (13135 results) S ['21475642'], traditional clerkship students (189 results) S ['21475642'], Parnassus (2269 results) S ['21475642'], continuity with patients (9722 results) S ['21475642'], direct observation of clinical (2210 results) S ['21475642'], traditional clerkships (15 results) D ['16799280'], continuity with faculty (853 results) S ['21475642'], core clerkships (27 results) S ['21475642'], LIC students (7 results) S ['19907386'], continuity with preceptors (44 results) S ['21475642', '19907386'], PISCES students (63 results) S ['21475642'], acute care sessions (522 results) S ['21475642'], traditional peers (296 results) S ['21475642'], clerkship (3921 results) S ['21475642'], preceptors (1189 results) S ['21475642']
JE	TRUE	0	FALSE	performance of nonphysician (44 results) D ['20562630'], offering cotrimoxazole (5 results) S ['17972365'], antiretroviral treatment program (32 results) D ['19687491'], antiretroviral treatment program (32 results) S ['19617847'], offering cotrimoxazole prophylaxis (3 results) S ['17972365'], clinics offering cotrimoxazole prophylaxis (1 results) S ['17972365'], national antiretroviral treatment (20 results) S ['19617847'], clinics offering cotrimoxazole (1 results) S ['17972365'], regimen-switch rates (2 results) S ['21483703'], antiretroviral comparison between low-income (9 results) D ['16530575'], antiretroviral therapy programs (26 results) D ['20586956', '19745753', '17941716'], national antiretroviral treatment program (3 results) S ['19617847']
JE	TRUE	13	TRUE	HIV-positive women (1287 results) S ['21483701'], HAART initiation (276 results) S ['21483701'], cancer mortality (7903 results) S ['21483701'], faster progression of cervical (14 results) S ['21483701'], cervical precancerous (2968 results) S ['21483701'], intraepithelial lesions amongst HIV-positive (1 results) D ['9662202'], intraepithelial lesions amongst (24 results) D ['9662202'], cervical cancer mortality (220 results) S ['21483701'], cervical cancer death amongst (6 results) S ['21483701'], HAART (8162 results) S ['21483701'], cumulative cervical (931 results) S ['21483701'], precancerous (24907 results) S ['21483701'], cumulative cervical cancer (509 results) S ['21483701'], squamous intraepithelial lesions amongst (9 results) D ['9662202'], cumulative cervical cancer mortality (170 results) S ['21483701'], lifetime cumulative cervical cancer (15 results) S ['21483701'], lifetime cumulative cervical (18 results) S ['21483701'], amongst women on HAART (29 results) S ['21483701'], scenarios with HAART (45 results) S ['21483701'], active antiretroviral therapy (8046 results) S ['21483701'], lesions amongst HIV-positive (13 results) D ['9662202'], lesions amongst HIV-positive (13 results) S ['21483701'], precancerous lesions (2439 results) S ['21483701'], active antiretroviral (8642 results) S ['21483701']

PMC3070736	0	TRUE	14	FALSE		3	12	TRUE	5	TRUE	5	TRUE	4	TRU
PMC3070735	0	TRUE	4	FALSE		1	4	FALSE	4	FALSE	4	FALSE	4	FAL
PMC3070733	1	FALSE	15	FALSE		5	15	TRUE	14	TRUE	13	TRUE	10	TRU
PMC3070731	10	FALSE	21	FALSE		9	30	TRUE	27	TRUE	23	TRUE	18	TRU

JE	TRUE	3	TRUE	positional versus object-location recall (2 results) S ['19883520'], object-location recall (4 results) S ['19883520'], medial temporal (3613 results) S ['21483699'], versus object-location (13 results) S ['19883520'], Alzheimer positional versus (2 results) S ['19883520'], Alzheimer positional versus object-location (1 results) S ['19883520'], Alzheimer positional (58 results) S ['19883520'], positional versus object-location (3 results) S ['19883520'], versus object-location recall (5 results) S ['19883520'], mechanism adjusts for ambiguous (1 results) S ['20381625'], adjusts for ambiguous (4 results) S ['20381625'], atrophy on MRI (10470 results) S ['21483699'], Categorical spatial memory (2 results) S ['19883520'], implicit memory (972 results) S ['21483699']
.SE	TRUE	4	TRUE	multidrug resistance protein (1788 results) S ['21483698'], uremic (14863 results) S ['21483698'], resistance protein (3355 results) S ['21483698'], multidrug resistance (14915 results) S ['21483698']
JE	TRUE	5	TRUE	myelinogenic pathways (4 results) S ['19211887', '17376971'], parallels memory impairment (85 results) S ['19211887'], Hippocampal expression analyses reveal (44 results) D ['16815214'], Hippocampal expression analyses reveal (44 results) S ['17376971'], selective association of immediate-early (16 results) S ['17376971'], active period (527 results) S ['21483696'], myelinogenic pathways with cognitive (2 results) S ['19211887', '17376971'], bioenergetic shift precedes (17 results) S ['19211887'], period deep sleep (10935 results) S ['21483696'], inactive period (95 results) S ['21483696'], neuroenergetic (7 results) S ['20427664', '17376971'], deep sleep (510 results) S ['21483696'], myelinogenic (61 results) S ['19211887', '17376971'], active period deep sleep (789 results) S ['21483696'], analyses reveal selective association (19 results) S ['17376971'], pathways with cognitive (5248 results) S ['21483696']
JE	TRUE	9	TRUE	cell line panel (250 results) S ['21483694', '20830292'], possibility of simvastatin (75 results) D ['18310894'], knockout causes lung adenocarcinoma (29 results) D ['17873910'], HCT-116 cells (374 results) S ['21483694'], NCI60 cell (65 results) S ['21483694', '21314952', '20830292'], HCT-116 (1068 results) S ['21483694'], line panel (258 results) S ['21483694', '20830292'], novel 38-kD islet (2 results) D ['8567980'], NCI60 (67 results) S ['21483694', '21314952', '20830292'], NCI60 cell line (8 results) S ['21483694', '20830292', '18330920'], NCI60 cancer cell lines (6 results) S ['21314952'], all-trans retinoic acid-resistant promyelocytic (33 results) D ['18310894'], simvastatin (6106 results) S ['21483694'], cell line panel indicates (181 results) S ['20830292'], novel 38-kD islet mitochondrial (1 results) D ['8567980'], Bioinformatic analyses identifies novel (7 results) S ['21314952'], NCI60 cancer cell (13 results) S ['21483694', '21314952', '20830292'], line panel indicates (168 results) S ['20830292'], 38-kD islet mitochondrial autoantigen (2 results) D ['8567980'], EAF2 expression (20 results) D ['17873910', '17044034', '15775981'], EAF2 expression (20 results) S ['21483694'], NCI60 cell line panel (7 results) S ['21483694', '20830292'], novel 38-kD (24 results) D ['8567980'], association study of NCI60 (5 results) S ['21483694', '21314952', '20830292', '18330920'], islet mitochondrial autoantigen (12 results) D ['8567980'], study of NCI60 (31 results) S ['21483694', '21314952', '20830292', '18330920'], lovastatin (4730 results) S ['21483694'], whole-genome SNP association study (198 results) S ['21483694'], EAF2 (26 results) D ['17873910', '17044034'], EAF2 (26 results) S ['21483694'], Bioinformatic analyses identifies (16 results) S ['21314952']

PMC3070730	1	FALSE	22	FALSE		6	21	TRUE	17	TRUE	16	TRUE	14	TRU
PMC3070728	4	FALSE	20	FALSE		5	23	TRUE	20	TRUE	20	TRUE	16	TRU
PMC3070727	1	FALSE	10	FALSE		4	11	TRUE	10	TRUE	10	TRUE	9	TRU
PMC3070726	7	FALSE	2	FALSE		6	9	TRUE	8	TRUE	7	TRUE	7	TRU
PMC3070725	4	FALSE	6	FALSE		3	7	TRUE	6	TRUE	6	TRUE	6	TRU
PMC3070724	0	TRUE	1	FALSE		1	1	FALSE	1	FALSE	1	FALSE	1	FAL

JE	TRUE	13	TRUE	Plasmodium vinckei petteri infections (2 results) S ['11191914'], vinckei petteri infections (2 results) S ['11191914'], basis of refractoriness (199 results) S ['21483693'], mosquito midgut (292 results) D ['20844583'], Plasmodium mechanism (1595 results) S ['21483693'], AcNOS (2 results) S ['21483693'], culicifacies species (20 results) S ['21483693', '11191914', '10717735', '3068854'], Anopheles (10848 results) S ['21483693'], molecular basis of refractoriness (34 results) S ['21483693'], culicifacies (388 results) S ['21483693'], AcNOS gene (1 results) S ['21483693'], AcNOS activity (1 results) S ['21483693'], refractory species (17 results) S ['21483693'], vivax (6300 results) S ['21483693'], refractoriness (5082 results) S ['21483693'], culicifacies mosquito (377 results) S ['21483693'], L-NAME (10036 results) S ['21483693'], midgut (5960 results) S ['21483693'], petteri infections (2 results) S ['11191914'], mechanism of refractoriness (595 results) S ['21483693'], oocyst (2609 results) S ['21483693'], Anopheles culicifacies (255 results) S ['21483693'], culicifacies mosquito midgut (7 results) S ['21483693', '17502004']
JE	TRUE	10	TRUE	calpain inhibitor MDL28170 (16 results) S ['21483751', '20457672', '19250597'], Trypanosoma (22276 results) S ['21483751'], inhibitor MDL28170 (17 results) S ['21483751', '20457672', '19250597'], detection of calpain (84 results) S ['19250597'], MDL28170 (48 results) S ['21483751', '20457672', '19250597'], relevant forms of Trypanosoma (48 results) S ['20457672'], cruzi (10926 results) S ['21483751'], midgut surface of Rhodnius (12 results) D ['17306256'], midgut surface of Rhodnius (12 results) S ['21483751'], Ennes-Vidal (3 results) S ['21483751', '20457672', '19250597'], Rhodnius prolixus (835 results) S ['21483751'], calpain (6060 results) S ['21483751'], calpain homologues (5 results) S ['19250597'], surface of Rhodnius (62 results) D ['17306256'], surface of Rhodnius (62 results) S ['21483751'], prolixus (863 results) S ['21483751'], calpain inhibitor (922 results) S ['21483751'], luminal midgut surface (5 results) D ['17306256'], luminal midgut surface (5 results) S ['21483751'], luminal midgut (99 results) S ['21483751'], Trypanosoma cruzi (10362 results) S ['21483751'], metacyclogenesis (151 results) S ['21483751'], epimastigote forms (238 results) D ['19374451'], epimastigote forms (238 results) S ['19250597']
JE	TRUE	5	TRUE	human psoriasis (68 results) S ['21483750'], Tie2 leads (28 results) S ['19342373'], cell-specific overexpression (33 results) S ['19342373'], Transcriptional profiles of leukocyte (426 results) S ['20686622'], mouse phenotypes (71 results) S ['21483750'], cell-specific overexpression of Tie2 (3 results) S ['19342373'], development of psoriasis (1938 results) S ['21483750'], psoriasis mouse models (330 results) S ['21483750'], psoriatic (9605 results) S ['21483750'], IFNgamma TNF regulates (18 results) D ['17928893'], psoriatic skin (1277 results) S ['21483750']
JE	TRUE	2	TRUE	hormonal regulation of adiposity (65 results) D ['17363694'], adipose tissue SR-BI (8 results) D ['17363694'], prepubertal twins (5 results) D ['19723754'], paternal intergenerational transmission (51 results) D ['20375189'], paternal intergenerational (121 results) D ['20375189'], Heritability (7856 results) S ['21483749'], pubertal relationship with anthropometric (53 results) D ['20450333'], Heritability of leptin (55 results) D ['19214176'], Heritability of leptin (55 results) S ['21483749']
JE	TRUE	6	TRUE	stem cell transplant analysis (17118 results) S ['21483748'], allogeneic stem cell (3584 results) S ['21483748'], aspergillosis after allogeneic (207 results) S ['21483748'], allogeneic stem cell transplantation (3083 results) S ['21483748'], SEIFEM-2008 (1 results) D ['19850903'], TLR6 polymorphisms (2 results) D ['16461792'], aspergillosis (13382 results) S ['21483748'], SEIFEM-2008 registry (1 results) D ['19850903'], SEIFEM-2008 registry study (1 results) D ['19850903'], allogeneic stem (3689 results) S ['21483748']
.SE	TRUE	1	TRUE	PCI (4308 results) S ['21483747']

PMC3070723	7	FALSE	11	FALSE		5	18	FALSE	18	FALSE	18	TRUE	16	TRU
PMC3070722	24	FALSE	5	FALSE		9	24	TRUE	19	TRUE	18	TRUE	14	TRU
PMC3070720	4	FALSE	23	FALSE		11	24	TRUE	18	TRUE	12	TRUE	11	TRU
PMC3070719	4	FALSE	4	FALSE		4	8	TRUE	7	TRUE	6	TRUE	4	TRU

JE	TRUE	13	TRUE	QFT-GIT with risk (19 results) D ['21239993'], QFT-GIT with risk (19 results) S ['21483746'], interferon-gamma release (461 results) S ['21483746'], QFT-GIT (46 results) D ['21239993'], QFT-GIT (46 results) S ['21483746'], latent tuberculosis (1262 results) S ['21483746'], Review T-cell interferon-gamma release (138 results) D ['19783328', '19387262'], skin test (9152 results) S ['21483746'], tuberculosis infection (3637 results) S ['21483746'], smear positive index case (161 results) D ['20388756'], smear positive index case (161 results) S ['21483746'], tuberculous infection (1595 results) S ['21483746'], release assays (1239 results) S ['21483746'], Review Interferon-gamma release (354 results) D ['21239993'], Review Interferon-gamma release assays (68 results) D ['21239993'], interferon-gamma release assays (199 results) D ['21239993'], interferon-gamma release assays (199 results) S ['21483746'], TST (2068 results) S ['21483746']
JE	TRUE	3	TRUE	Forino (82 results) D ['19852440', '18063381'], Ciminiello (44 results) D ['19852440', '18063381', '16944897'], palytoxin analogue (4 results) D ['19852440', '18063381'], palytoxin analogue from Hawaiian (1 results) D ['19852440'], palytoxin-like (18 results) D ['20797402', '19852440', '18063381'], palytoxin-like (18 results) S ['21483745'], Mediterranean Ostreopsis (15 results) D ['20797402', '18063381', '16944897'], Ostreopsis (50 results) D ['20797402'], Ostreopsis (50 results) S ['21255599'], New palytoxin-like (6 results) D ['20797402', '19852440', '18063381'], Palythoa subspecies (1 results) D ['19852440'], palytoxin-like molecules (2 results) D ['20797402'], Hawaiian Palythoa subspecies (1 results) D ['19852440'], ovata (368 results) D ['20797402'], Palythoa (68 results) D ['20797402', '19852440'], Palythoa (68 results) S ['21483745', '19505494'], Hawaiian Palythoa (1 results) D ['19852440'], New palytoxin-like molecules (1 results) D ['20797402'], palytoxin (325 results) D ['20797402'], palytoxin (325 results) S ['21483745', '21255599'], new palytoxin analogue (3 results) D ['19852440', '18063381'], analogue from Hawaiian (6 results) D ['19852440'], Dell'Aversano (47 results) D ['19852440', '18063381', '16944897'], new palytoxin (45 results) D ['20797402', '19852440', '18063381'], Ostreopsis ovata (15 results) D ['18063381', '16944897'], Genoa outbreak (23 results) D ['16944897'], zoanthid (46 results) D ['18433818'], zoanthid (46 results) S ['21483745', '21255599', '19505494', '19267653'], putative palytoxin (6 results) D ['18063381', '16944897']
JE	TRUE	6	TRUE	apicoplast periphery identifies abundant (1 results) S ['18586952'], novel constituents for host-parasite (5 results) S ['19134112', '18765740', '16002398'], constituents for host-parasite (33 results) S ['19134112', '18765740', '16002398'], Toxoplasma gondii (8892 results) S ['21483743'], periphery identifies abundant (4 results) S ['18586952'], identifies abundant candidate (18 results) S ['18586952'], reveals many novel constituents (3 results) S ['16002398'], apicoplast periphery (3 results) S ['18586952'], Proteomic analysis of rhoptry (8 results) D ['17022100'], Proteomic analysis of rhoptry (8 results) S ['17997128', '16002398'], identifies abundant candidate transport (2 results) S ['18586952'], microneme protein mediates (3 results) D ['12062488'], rhoptry organelles reveals (10 results) S ['16309467', '16002398'], abundant candidate transport vesicles (10 results) S ['18586952'], Neospora (1442 results) S ['21483743'], rhoptry proteomic (8 results) D ['17022100'], rhoptry proteomic (8 results) S ['17997128', '16002398'], abundant candidate transport (57 results) S ['18586952'], periphery identifies abundant candidate (1 results) S ['18586952'], Toxoplasma (12836 results) S ['21483743'], apicoplast periphery identifies (1 results) S ['18586952'], caninum (2194 results) S ['21483743'], Neospora caninum (1266 results) S ['21483743'], periphery identifies (60 results) S ['18586952'], Toxoplasma integration (43 results) D ['19218426'], Toxoplasma integration (43 results) S ['18644147'], gondii (9114 results) S ['21483743']
JE	TRUE	3	TRUE	ultimatum game Evidence (14 results) D ['17251437'], complex social strategic (118 results) S ['21483742'], social strategic behaviors (622 results) S ['21483742'], Ultimatum Game (77 results) S ['21483742'], superior stronger recruitment (11 results) D ['16122946'], stronger recruitment of posterior (6 results) D ['16122946'], electromagnetic tomography study (5 results) D ['12668236'], Mathematically (4703 results) S ['21483742']

PMC3070701	7	FALSE	24	FALSE		5	30	TRUE	24	TRUE	22	TRUE	20	TRU
PMC3070700	9	FALSE	4	FALSE		7	12	TRUE	11	TRUE	9	TRUE	7	TRU
PMC3070699	4	FALSE	9	FALSE		4	13	FALSE	13	FALSE	13	TRUE	12	TRU
PMC3070698	6	FALSE	11	FALSE		7	14	TRUE	9	TRUE	8	TRUE	8	TRU
PMC3070697	1	FALSE	10	FALSE		3	11	TRUE	10	TRUE	9	TRUE	8	TRU

JE	TRUE	15	TRUE	species richness (2605 results) S ['21483739'], richness of vertebrate (2053 results) S ['21483739'], comparative approach on quantification (678 results) S ['21483739'], tree crowns of oak (3 results) S ['21483739'], herbivorous arthropods (30 results) S ['21483739'], herbivory through predation (107 results) S ['21483739'], bird predation on arthropods (182 results) D ['20380198'], bird predation on arthropods (182 results) S ['21483739'], herbivory (1296 results) S ['21483739'], leaf damage highlights (5 results) S ['21483739'], effects of insectivory (4 results) D ['16315345'], insectivorous vertebrates on leaf (12 results) D ['18665271'], insectivorous vertebrates on leaf (12 results) S ['21483739'], vertebrate predators for control (333 results) S ['21483739'], arthropod herbivory (9 results) S ['21483739'], arboreal herbivorous arthropods (1 results) S ['21483739'], predation on arthropods (2402 results) S ['21483739'], effects of vertebrate (3685822 results) S ['21483739'], indirect effects of insectivory (2 results) D ['16315345'], forest composition (48 results) S ['21483739'], species richness of vertebrate (1152 results) S ['21483739'], tropical agroecosystems (8 results) D ['19831075'], temperate (7499 results) S ['21483739'], predation (4201 results) S ['21483739'], arthropod (6539 results) S ['21483739'], leaf damage (151 results) S ['21483739'], clear effects of insectivorous (3 results) S ['21483739'], Functional richness (27 results) D ['19831075'], vertebrate predators (42 results) S ['21483739'], insectivory (32 results) D ['16315345'], exclusion experiments with nets (2 results) S ['21483739']
JE	TRUE	4	TRUE	Listeria (14497 results) S ['21483738'], non-culturable state of Salmonella (11 results) D ['10379808'], vivo detection of Listeria (22 results) D ['10919515'], saliva-conditioned (6 results) D ['10966431'], coadhesion with Streptococcus (11 results) D ['10966431'], Listeria monocytogenes (12214 results) S ['21483738'], ecometric technique (5 results) D ['14503736', '7216926'], fluorescent protein expression vectors (6 results) D ['10919515'], state of Salmonella (3834 results) S ['21483738'], ecometric (23 results) D ['14503736'], species-specific marker (31 results) D ['10966431', '9720030'], monocytogenes (12430 results) S ['21483738'], so-called ecometric (1 results) D ['7216926']
JE	TRUE	10	TRUE	metabolome (1553 results) S ['21483737'], lateral sclerosis (13338 results) S ['21483737'], lateral sclerosis without superoxide (2320 results) S ['21483737'], SOD1 (2688 results) S ['21483737'], FALS (593 results) S ['21483737'], amyotrophic lateral sclerosis (13197 results) S ['21483737'], SALS (304 results) S ['21483737'], sclerosis without superoxide (2405 results) S ['21483737'], cerebral creatinine involvement (72 results) D ['18752646'], amyotrophic (13391 results) S ['21483737'], negative for SOD1 (75 results) D ['19496940'], cases negative for SOD1 (15 results) D ['19496940'], amyotrophic lateral discovery (186 results) D ['20698585']
JE	TRUE	6	TRUE	sequence-dependent activation of Toll-like (10 results) D ['18537714', '17355967'], receptor associates with c-Src (110 results) D ['16858407'], correlation spectroscopy resolves EGFR (1 results) S ['20586411'], Fluorescence lifetime (2273 results) S ['21483736'], Fluorescence lifetime cross correlation (36 results) S ['21483736', '20586411'], CpG DNA (745 results) S ['21483736'], live-cell fluorescence fluctuation spectroscopy (4 results) D ['18431466', '18077328'], spectroscopy resolves EGFR (2 results) S ['20586411'], sequence-independent cooperative effect (4 results) D ['17355967'], correlation spectroscopy (3278 results) S ['21483736'], TLR9 (1971 results) S ['21483736'], stoichiometry (13348 results) S ['21483736'], cooperative effect on sequence-dependent (4 results) D ['17355967'], non CpG DNA (27 results) S ['21483736'], TLR9-GFP (1 results) S ['21483736'], cross correlation spectroscopy resolves (1 results) S ['20586411'], live-cell fluorescence fluctuation (6 results) D ['18431466', '18077328']
JE	TRUE	7	TRUE	application of phylogenetic (1220 results) S ['21483735'], genome protein phylogenetic (11341 results) S ['21483735'], phylogenetic profiles (149 results) S ['21483735'], silico prioritisation of candidate (3 results) S ['19292914'], genome protein phylogenetic profiles (336 results) S ['21483735'], agalactiae (6941 results) S ['21483735'], silico prioritisation (6 results) S ['19292914'], prioritisation of candidate (12 results) S ['19292914'], Neisseria core genome (49 results) D ['17159225'], virulence genes (2514 results) S ['21483735'], prokaryotic gene (130 results) S ['19292914']

PMC3070695	13	FALSE	4	FALSE		6	12	TRUE	8	TRUE	8	TRUE	7	TRU
PMC3070694	9	FALSE	1	FALSE		9	10	TRUE	4	TRUE	3	TRUE	2	TRU
PMC3070693	5	FALSE	2	FALSE		7	7	TRUE	2	TRUE	1	TRUE	0	TRU
PMC3076264	5	FALSE	12	FALSE		4	13	TRUE	8	TRUE	8	TRUE	6	TRU
PMC3076229	2	FALSE	7	FALSE		3	9	TRUE	8	TRUE	8	TRUE	7	TRU
PMC3076263	5	FALSE	8	FALSE		6	13	TRUE	12	TRUE	11	TRUE	9	TRU
PMC3076266	2	FALSE	9	FALSE		6	11	TRUE	10	TRUE	9	TRUE	7	TRU

JE	TRUE	3	TRUE	domestication (1488 results) S ['21483733'], canine MHC-II (5 results) D ['16101828'], histocompatibility complex Loci (48 results) D ['16702436'], major histocompatibility complex Loci (47 results) D ['16702436'], representative American Kennel (1 results) D ['16101828'], coat color (923 results) S ['21483733'], Considerable haplotypic diversity (4 results) D ['15578174'], domestic European pig breeds (35 results) D ['18565161'], domestic European pig breeds (35 results) S ['21483733'], canine MHC-II DLA-DQB1 (1 results) D ['16101828'], RT1-CE class (2 results) D ['15578174'], MHC-II DLA-DQB1 (1 results) D ['16101828'], Considerable haplotypic (14 results) D ['15578174'], representative American Kennel Club (1 results) D ['16101828'], nucleotide diversity (1088 results) S ['21483733'], RT1-CE (2 results) D ['15578174'], mutation rates from high-coverage (1 results) D ['18725384']
JE	TRUE	1	TRUE	Harveian Lectures (31 results) D ['20759317', '20759310', '20759303'], Maryland Tuberculosis Sanatoria (2 results) D ['18015444'], pulmonary tuberculosis (66870 results) S ['21483732'], Danish tuberculosis patients (3 results) D ['5301648'], Danish mass tuberculosis campaign (2 results) D ['13716055'], Italian Multidrug-Resistant Tuberculosis Outbreak (4 results) D ['9662207'], mass tuberculosis campaign (2 results) D ['13716055'], Harveian Lectures on Prognosis (9 results) D ['20759317', '20759310', '20759303'], Harveian Society of London (21 results) D ['20759317', '20759310', '20759303'], five-year epidemiological study (3 results) D ['4549498']
JE	FALSE	0	FALSE	medicinal Mexican morning (6 results) D ['18500841', '16562846'], bacterial NorA efflux (4 results) D ['18524600'], bacterial NorA efflux (4 results) S ['16203150'], NorA efflux pump inhibitors (6 results) D ['18848780', '18524600'], NorA efflux pump inhibitors (6 results) S ['16203150'], bacterial efflux pump (15 results) D ['18358571', '17275293'], medicinal Mexican morning glory (5 results) D ['18500841', '16562846']
JE	TRUE	6	TRUE	European ST-T Database (19 results) D ['19692287'], European ST-T Database (19 results) S ['21457580'], bi-phasic (365 results) S ['21457580'], well-known ECG delineator (4 results) D ['15072211'], well-known ECG delineator (4 results) S ['21457580'], linear algebra online processing (1 results) S ['21457580'], QRS detection (113 results) S ['21457580'], integer linear algebra processing (1 results) S ['21457580'], ECG characteristic points (2 results) D ['7851927'], 32-bit integer linear (1 results) S ['21457580'], wave delineation criterion (4 results) D ['19692287'], Detection of ECG (5643 results) S ['21457580'], 32-bit integer linear algebra (1 results) S ['21457580'], ECG delineation (152 results) D ['19692287'], ECG delineation (152 results) S ['21457580'], wavelet-based (780 results) S ['21457580'], integer linear algebra (2 results) S ['21457580']
JE	TRUE	6	TRUE	Korean nonsmokers (95 results) S ['21457578'], tobacco smoke exposure (933 results) S ['21457578'], environmental tobacco (2579 results) S ['21457578'], smoke exposure (2775 results) S ['21457578'], environmental tobacco smoke (2519 results) S ['21457578'], environmental tobacco smoke exposure (364 results) S ['21457578'], tobacco smoke (12292 results) S ['21457578'], ETS exposure during leisure (11 results) D ['9850125'], Scania Public Health (2 results) D ['15204180']
JE	TRUE	6	TRUE	chondrosarcoma specimens (11 results) S ['21457573'], gene survivin (50 results) S ['21457573', '17982126'], adult renal tubular implications (117 results) S ['17982126'], long-term infusion therapy (10 results) D ['3480081'], chondrosarcoma (6731 results) S ['21457573'], tumor gene survivin (1509 results) S ['21457573'], chondrosarcoma cells (225 results) S ['21457573'], Curative treatment of chondrosarcoma (51 results) D ['19602594', '18378543'], human chondrosarcoma (214 results) S ['21457573'], plasma adriamycin concentrations (5 results) D ['3480081'], Ewing's National Cancer Data (54 results) D ['17414166'], human high grade chondrosarcoma (325 results) S ['21457573'], plasma adriamycin (16 results) D ['3480081']
JE	TRUE	5	TRUE	decision support systems (4903 results) S ['21457572'], support systems (8785 results) S ['21457572'], pediatric caregivers (16 results) S ['21457572'], caregiver role (144 results) S ['21457572'], pharmacotherapy guidance (4493 results) S ['21457572'], pediatric pharmacotherapy (25 results) S ['21457572', '18226244', '17656617'], caregivers via email (10 results) S ['21457572'], pharmacotherapeutic evaluation (4 results) S ['16239364'], pharmacotherapy resources (6440 results) S ['21457572'], Stockholm metropolitan health (34 results) D ['16621683'], pediatric knowledgebase (19 results) D ['17200261']

PMC3029495	3	FALSE	4	FALSE		4	6	TRUE	3	TRUE	3	TRUE	1	TRU
PMC3064481	3	FALSE	17	FALSE		5	20	TRUE	19	TRUE	17	TRUE	15	TRU
PMC3062696	1	FALSE	2	FALSE		2	3	FALSE	3	FALSE	3	FALSE	3	TRU
PMC3071976	11	FALSE	8	FALSE		7	19	TRUE	17	TRUE	17	TRUE	8	TRU
PMC3075411	0	TRUE	2	FALSE		1	2	FALSE	2	FALSE	2	FALSE	2	FAL
PMC3075798	4	FALSE	9	FALSE		3	13	FALSE	13	TRUE	10	TRUE	10	TRU
PMC3074228	2	FALSE	8	FALSE		5	10	TRUE	8	TRUE	6	TRUE	6	TRU

JE	TRUE	0	FALSE	June 2004-April MMWR (1 results) D ['15973239'], homonegativity scores (2 results) S ['20043200'], higher homonegativity (13 results) D ['19072529'], higher homonegativity (13 results) S ['20043200', '19526346'], homonegativity (41 results) S ['20043200', '19526346'], 2004-April MMWR (3 results) D ['15973239'], higher homonegativity scores (6 results) S ['20043200', '19526346']
JE	TRUE	7	TRUE	dichotomous outcome of sex (73 results) S ['21271397'], public sex venues (171 results) S ['21271397'], gay community (301 results) S ['21271397'], sex environments (19 results) D ['18956982', '15832876', '12511214'], public sex (32 results) D ['18956982', '15832876'], public sex (32 results) S ['21271397'], Latino MSM (28 results) S ['21271397', '19030982'], recent immigration (105 results) S ['21271397'], gay community involvement (4 results) S ['21271397'], public place (115 results) S ['21271397'], anal intercourse among immigrant (9 results) S ['19030982'], commercial sex environments (6 results) D ['12511214'], sex venues (28 results) S ['21271397'], intercourse among immigrant (37 results) S ['19030982'], greater gay community involvement (12 results) S ['21271397'], practices of HIV-positive (1202 results) S ['21271397'], bathhouses (99 results) S ['21271397'], lower self-efficacy for safer (35 results) S ['21271397'], settings among Latino (426 results) S ['21271397'], self-efficacy for safer (219 results) S ['21271397']
JE	TRUE	1	TRUE	MMSE scores over time (259 results) S ['20808140'], multilevel regression models (92 results) S ['20808140'], subclinical Alzheimer pathology (32 results) D ['15383746']
JE	TRUE	7	TRUE	Autocorrelation curves (17 results) D ['17447726'], fluorescence correlation (1330 results) S ['21396180'], form solution for FCS (15 results) D ['21038906'], FCS with one-photon (11 results) D ['17447726'], FCCS excitation (21 results) D ['20073480'], fluorescence lifetime correlation spectroscopy (12 results) D ['17171439'], fluorescence lifetime correlation spectroscopy (12 results) S ['21396180'], total internal reflection fluorescence (866 results) S ['21396180'], waveguides with FCS (5 results) D ['18607415'], time-correlated (804 results) S ['21396180'], Spatial two-photon fluorescence cross-correlation (5 results) D ['12236358'], fluorescence correlation spectroscopy (1268 results) S ['21396180'], fluorescence cross-correlation spectroscopy (127 results) D ['20073480'], lifetime correlation spectroscopy (12 results) D ['17171439'], lifetime correlation spectroscopy (12 results) S ['21396180'], fluorescence lifetime correlation (12 results) D ['17171439'], fluorescence lifetime correlation (12 results) S ['21396180'], lifetime correlation (1453 results) S ['21396180'], fluorescence cross-correlation (146 results) D ['20073480']
.SE	TRUE	2	TRUE	Disease Study (1916 results) S ['21482873'], Carotenoids (15017 results) S ['21482873']
JE	TRUE	9	TRUE	serum cotinine (401 results) S ['21464381'], SHS exposure on mental (8 results) D ['20529994'], cotinine (3696 results) S ['21464381'], Optimal serum cotinine levels (9 results) D ['19019851'], Bandiera (409 results) S ['21464381'], psychological mechanisms of associations (1336 results) S ['21464381'], conduct disorder (3255 results) S ['21464381'], Merikangas (288 results) S ['21464381'], Optimal serum cotinine (9 results) D ['19019851'], cotinine level (235 results) D ['20529994'], cotinine level (235 results) S ['21464381'], serum cotinine level (45 results) S ['21464381'], DSM-IV symptoms (117 results) S ['21464381']
JE	TRUE	6	TRUE	associations of MDD (635 results) S ['21464384'], media exposures (9 results) D ['19592322'], media exposures (9 results) S ['19306219', '19188540'], Dillman Carpentier (2 results) S ['21464384'], Switzer (958 results) S ['21464384'], Participants with MDD (457 results) S ['21464384'], Primack (256 results) S ['21464384'], Depressive Disorder Division (2019 results) S ['21464384'], Lifetime Version initial reliability (4 results) D ['9204677'], media use (232 results) S ['21464384']

PMC3064729	0	TRUE	20	FALSE		3	20	TRUE	18	TRUE	16	TRUE	16	TRU
PMC3074478	7	FALSE	10	FALSE		8	17	TRUE	10	TRUE	8	TRUE	7	TRU
PMC3062784	6	FALSE	14	FALSE		9	19	TRUE	11	TRUE	4	TRUE	3	TRU
PMC3060341	5	FALSE	14	FALSE		8	11	TRUE	6	TRUE	5	TRUE	3	TRU

JE	TRUE	10	TRUE	regulatory fit (30 results) S ['21264696', '19412324', '18229485'], stimulus identification task (568 results) S ['21264696'], fit effect on sensitivity (963 results) S ['21264696'], regulatory mismatch (5 results) S ['21264696'], regulatory fit leads (29 results) S ['19412324'], local reward structure (37 results) S ['21264696'], regulatory focus (119 results) S ['21264696'], regulatory fit effect (141 results) S ['21264696'], task reward structure (3 results) S ['21264696', '19412324'], local task (48 results) S ['21264696'], task reward (8 results) S ['21264696', '19412324'], local task reward (71 results) S ['21264696'], internal perceptual (936 results) S ['21264696'], promotion-losses (149 results) S ['21264696'], reward structure (40 results) S ['21264696', '19412324'], local task reward structure (8 results) S ['21264696'], stimulus identification (124 results) S ['21264696'], perceptual sensitivity (232 results) S ['21264696'], promotion-gains (381 results) S ['21264696'], global regulatory focus (157 results) S ['21264696']
JE	TRUE	1	TRUE	pair of G-quadruplexes (31 results) S ['19601575'], DNA G-quadruplexes (31 results) S ['18355457'], c-Myc implications for drug (86 results) S ['19385599'], intramolecular DNA (88 results) D ['18092816'], intramolecular DNA (88 results) S ['18355457'], eukaryotic promoter regions (12 results) S ['18355457'], reveal G-quadruplex DNA motifs (6 results) D ['18767830'], single topoisomers (3 results) D ['6096558'], G-quadruplex DNA motifs (89 results) S ['21428953'], analyses reveal G-quadruplex DNA (4 results) D ['18767830'], novel GGA repeat region (3 results) S ['18252774'], analyses reveal G-quadruplex (4 results) D ['18767830'], G-quadruplexes (1222 results) S ['21428953'], intramolecular DNA G-quadruplexes (5 results) D ['18092816'], intramolecular DNA G-quadruplexes (5 results) S ['18355457'], novel GGA repeat (8 results) S ['18252774'], expression analyses reveal G-quadruplex (2 results) D ['18767830']
JE	TRUE	0	FALSE	metal ion stress (8 results) S ['21223946'], Crz1p (34 results) S ['21223946', '17337637'], membrane protein Alr1 controls (3 results) S ['11279208'], plasma membrane protein Alr1 (7 results) S ['16903865', '11279208'], tetraspan topology (4 results) D ['12538885'], Alr1 controls (5 results) S ['11279208'], prevents intracellular cell wall (60 results) D ['18799621'], membrane protein Alr1 (18 results) S ['16903865', '11279208'], Sur7 protein (10 results) D ['18799621', '16496001'], PUN1 (7 results) S ['21223946'], protein Alr1 controls (4 results) S ['11279208'], Pun1p (2 results) S ['21223946'], Sur7 protein regulates (2 results) D ['18799621'], finger proteins Msn2p (8 results) S ['9472026', '8641288'], ion stress (28 results) S ['21223946'], zinc finger proteins Msn2p (8 results) S ['9472026', '8641288'], Sur7 protein regulates plasma (2 results) D ['18799621'], intracellular cell wall (10 results) D ['18799621'], induction of PUN1 (1 results) S ['21223946'], regulation of PUN1 (3 results) S ['21223946']
JE	TRUE	2	TRUE	Green-Thompson Jimenez-Sanchez (1 results) S ['20959619'], Bryson-Richardson Hall Berger (4 results) D ['19736328', '17438294'], Davies Futter (4 results) S ['20959619'], Rubinsztein Regulation (17 results) S ['21256213', '20959619', '20566712', '18701682'], Sachs Geisler Stemple (1 results) D ['19736328'], Futter Garcia-Arencibia Green-Thompson Jimenez-Sanchez (1 results) S ['20959619'], Korolchuk Renna (4 results) S ['20959619', '20566712', '19461070'], Renna (210 results) S ['20959619'], Rubinsztein (262 results) S ['21256213', '20959619'], Sarkar Davies Futter (1 results) S ['20959619'], Narayanan Renna Siddiqi Underwood (1 results) S ['20959619'], Garcia-Arencibia (19 results) S ['20959619', '20566712'], Sachs Geisler Stemple Currie (1 results) D ['19736328'], Futter Garcia-Arencibia Green-Thompson (1 results) S ['20959619'], Korolchuk Lichtenberg (3 results) S ['20959619', '19461070'], Murphy Rubinsztein (8 results) S ['20566712'], Jacoby Busch-Nentwich Bryson-Richardson Hall (1 results) D ['19736328'], Korolchuk (26 results) S ['20959619', '20566712', '19461070', '19411822'], Sachs Geisler (4 results) D ['19736328']

PMC3078889	3	FALSE	18	FALSE		8	19	TRUE	16	TRUE	12	TRUE	9	TRU
PMC3078853	8	FALSE	1	FALSE		9	8	TRUE	5	TRUE	3	TRUE	2	TRU
PMC3069684	0	TRUE	10	FALSE		1	10	FALSE	10	FALSE	10	TRUE	9	TRU
PMC3066848	0	TRUE	9	FALSE		1	9	TRUE	7	TRUE	7	TRUE	6	TRU
PMC3066847	0	TRUE	15	FALSE		1	15	TRUE	11	TRUE	9	TRUE	9	TRU
PMC3066845	2	FALSE	3	FALSE		2	1	TRUE	0	TRUE	0	TRUE	0	TRU
PMC3066842	9	FALSE	5	FALSE		3	6	TRUE	5	TRUE	3	TRUE	3	TRU

JE	TRUE	3	TRUE	River gorilla population (9 results) D ['18077351'], River gorilla population (9 results) S ['21457536', '18521886', '17257109'], contemporary gorillas (17 results) S ['21457536', '15643961'], Cross River population (317 results) S ['21457536'], Cross River population size (49 results) S ['21457536', '18521886'], contemporary Cross River (6 results) S ['21457536'], contemporary Cross River gorillas (1 results) S ['21457536'], ape populations (28 results) D ['16417405'], ape populations (28 results) S ['21457536'], chimpanzees (4474 results) S ['21457536'], gorilla population (14 results) S ['21457536', '18521886', '17257109'], secondary admixture of western (14 results) S ['21457536'], Cross River (151 results) S ['21457536'], River gorillas (3 results) S ['21457536'], Cross River museum specimens (1 results) S ['21457536'], contemporary samples (46 results) S ['21457536'], western gorillas (37 results) D ['19804402', '17712021'], western gorillas (37 results) S ['21457536'], Cross River gorillas (3 results) S ['21457536'], Cross River gorilla population (5 results) S ['21457536', '18521886', '17257109'], secondary admixture (8 results) S ['21457536']
JE	TRUE	1	TRUE	BCL2 gene promoter (3 results) D ['19196738', '17908970', '16960146'], prognostic value of Bag-1 (8 results) D ['18430249'], value of Bag-1 (25 results) D ['18430249'], Bcl-2 expression (4182 results) S ['21457555'], regulatory BCL2 promoter polymorphism (4 results) D ['19196738', '17908970'], regulatory BCL2 promoter (4 results) D ['19196738', '17908970'], BCL2 promoter polymorphism (4 results) D ['19196738', '17908970'], detectable bcl-2 expression (6 results) D ['7547253'], BCL2 promoter (21 results) D ['20082279', '19237173', '19196738', '17959858', '17908970', '16960146']
JE	TRUE	8	TRUE	adolescent mental health services (226 results) S ['21459975'], care arm odds (245 results) S ['21459975'], routine care arm odds (18 results) S ['21459975'], developmental group (31 results) S ['21459975'], episodes between developmental (826 results) S ['21459975'], self harm (1804 results) S ['21459975'], proportional odds (397 results) S ['21459975'], routine care (1940 results) S ['21459975'], adolescent mental health (895 results) S ['21459975'], care arm odds ratio (212 results) S ['21459975']
JE	TRUE	4	TRUE	medical care resembles (86 results) S ['21450778'], multidisciplinary centres (12 results) S ['21450778'], healthcare quality (890 results) S ['21450778'], relevant clinical disciplines (3 results) S ['21450778'], serious man-made threat (4 results) S ['21450778'], Multidisciplinary centres for safety (26 results) S ['21450778'], practical experience (1283 results) S ['21450778'], clinical disciplines (281 results) S ['21450778'], practical experience of improvement (374 results) S ['21450778']
JE	TRUE	8	TRUE	effective intergroup dynamics and (2 results) S ['21450775'], socialisation (323 results) S ['21450775'], superordinate (425 results) S ['21450775'], effective intergroup relationships (5 results) S ['21450775'], intergroup (4491 results) S ['21450775'], healthcare settings (1069 results) S ['21450775'], more effective intergroup (163 results) S ['21450775'], effective intergroup (383 results) S ['21450775'], intergroup dynamics (10 results) S ['21450775'], more effective intergroup dynamics (2 results) S ['21450775'], more effective intergroup relationships (2 results) S ['21450775'], dual identities within healthcare (9 results) S ['21450775'], intergroup relationships (23 results) S ['21450775'], communities of practice (8463 results) S ['21450775'], social identity (497 results) S ['21450775']
JE	FALSE	0	FALSE	Medical Research Council guidance (1 results) D ['18824488'], snail moral syndromes (1 results) S ['21450772'], respectful interaction between evangelist (1 results) S ['21450772'], interaction between evangelist (1 results) S ['21450772'], new Medical Research Council (4 results) D ['18824488']
JE	TRUE	2	TRUE	improvement initiatives (595 results) S ['21450770'], meets actor-network theory (1 results) D ['20185218'], unit of Mendota (9 results) D ['15278987'], strong structuration theory meets (1 results) D ['20185218'], structuration theory meets (1 results) D ['20185218'], theory meets actor-network (1 results) D ['20185218'], theory meets actor-network theory (1 results) D ['20185218'], legitimate value sets (5 results) S ['21450770'], origins of coercion (9 results) D ['15278987'], meets actor-network (1 results) D ['20185218'], quality improvement initiatives (438 results) S ['21450770'], structuration theory meets actor-network (1 results) D ['20185218'], micropolitical dimensions (1 results) S ['21450770'], value sets (37 results) S ['21450770']

PMC3066840	0	TRUE	2	FALSE		1	2	FALSE	2	FALSE	2	FALSE	2	FAL
PMC3066838	2	FALSE	3	FALSE		3	5	FALSE	5	TRUE	4	TRUE	4	TRU
PMC3066837	2	FALSE	2	FALSE		3	4	TRUE	2	TRUE	1	TRUE	0	TRU
PMC3066836	0	TRUE	3	FALSE		1	3	FALSE	3	FALSE	3	FALSE	3	TRU
PMC3066835	2	FALSE	2	FALSE		3	3	TRUE	2	TRUE	1	TRUE	1	TRU
PMC3066793	4	FALSE	4	FALSE		5	7	TRUE	5	TRUE	5	TRUE	3	TRU
PMC3066789	0	TRUE	3	FALSE		1	3	FALSE	3	FALSE	3	FALSE	3	FAL
PMC3066698	4	FALSE	3	FALSE		2	7	FALSE	7	TRUE	5	TRUE	5	TRU
PMC3066696	3	FALSE	3	FALSE		2	6	TRUE	4	TRUE	3	TRUE	3	TRU
PMC3066695	3	FALSE	1	FALSE		3	4	TRUE	3	TRUE	3	TRUE	3	TRU
PMC3064992	8	FALSE	9	FALSE		8	15	TRUE	13	TRUE	12	TRUE	12	TRU
PMC3078283	14	FALSE	3	FALSE		12	16	TRUE	12	TRUE	11	TRUE	10	TRU

.SE	TRUE	2	TRUE	role of innovation (5173 results) S ['21450771'], positive innovation (1370 results) S ['21450771']
JE	TRUE	2	TRUE	strength of implications (3409 results) S ['21450773'], Ann Intern (57 results) S ['21450773'], physicians' inpatient treatment decisions (45 results) D ['15109337'], journals report research (649 results) S ['21450773'], general theory of indifference (7 results) D ['17716420']
JE	FALSE	0	FALSE	participants obscures (11 results) S ['20009058'], study participants obscures (8 results) S ['20009058'], Seventy-five trials (4 results) D ['20877712'], general health care journals (4 results) D ['11914308']
JE	TRUE	2	TRUE	Institute for Healthcare (272 results) S ['21450765'], Plato's (100 results) S ['21450765'], Healthcare Improvement (416 results) S ['21450765']
JE	TRUE	0	FALSE	pediatric infectious diseases consultation (2 results) D ['15865280'], publishable knowledge (10 results) S ['21450777'], gown isolation precautions (1 results) D ['3600729'], improvement work (96 results) S ['21450777']
JE	TRUE	2	TRUE	across-case approaches (2 results) D ['12891720'], organisational research (13 results) S ['21450767'], case studies of quality (12369 results) S ['21450767'], case study research (90 results) S ['21450767'], realist evaluation of whole-scale (1 results) D ['19523123'], study research (425 results) S ['21450767'], magic targets (3 results) D ['12146782'], organizational case study (12 results) D ['19948064', '19523123']
.SE	TRUE	3	TRUE	longitudinal factorial (161 results) S ['21450768'], Health-services researchers (239 results) S ['21450768'], clinical researchers (655 results) S ['21450768']
JE	TRUE	3	TRUE	clinical quality improvement (101 results) S ['21450763'], protein free synthetic (23 results) D ['20091513'], clinical quality (732 results) S ['21450763'], EBM (2001 results) S ['21450763'], protein free synthetic surfactant (8 results) D ['20091513'], free synthetic surfactant (8 results) D ['20091513'], Prophylactic protein free synthetic (21 results) D ['20091513']
JE	TRUE	1	TRUE	generalisability of improvement (24 results) S ['21450781'], SQUIRE Publication (30 results) D ['19025090'], theory of improvement (2615 results) S ['21450781'], SQUIRE project (5 results) D ['19025090'], SQUIRE Publication evolution (5 results) D ['19025090'], improvement replication programme (6 results) S ['21450781']
JE	TRUE	1	TRUE	Organizational readiness (63 results) D ['19594942'], elder care building context (4 results) D ['19671167'], Rycroft-Malone (47 results) D ['19671167'], context influences (129 results) S ['21450764']
JE	TRUE	1	TRUE	Braddon Wadsworth (2 results) D ['3256576'], short-term precision of hip (43 results) S ['15869917'], hip structural analysis (50 results) D ['18684092'], hip structural analysis (50 results) S ['21130909'], structural geometry (73 results) S ['21130909'], Stone Hillier Cauley (27 results) D ['18684092'], Beavan Cole (1 results) S ['9537615'], breast-milk output (23 results) S ['9537615'], Wahner Structural trends (1 results) D ['11127194'], bone strength during lactation (41 results) D ['16091837'], bone strength during lactation (41 results) S ['21130909'], endosteal diameter (22 results) D ['15005842'], absorptiometry scans from multi-center (3 results) S ['15869917'], femur geometry variables (61 results) D ['18684092'], femur geometry variables (61 results) S ['21130909'], hip during lactation (126 results) S ['21130909'], Wahner Structural (6 results) D ['11127194']
JE	TRUE	1	TRUE	blind spot enlargement (42 results) D ['18439564'], outer segment line (311 results) D ['20673590'], outer retinal microstructures (8 results) D ['20472291'], AZOOR complex diseases (4 results) D ['18439564', '12614758'], eyes with AZOOR (30 results) D ['18778338', '18547281', '18439564', '17846358'], eyes with AZOOR (30 results) S ['20661173'], AZOOR complex disorders (4 results) D ['18439564', '12614758'], outer retinopathy acute macular (38 results) D ['18778338'], severe AZOOR cases (1 results) D ['19684865'], cone outer segment (99 results) D ['20164460'], cone outer segment tips (16 results) D ['20164460', '18385077'], diseases including AZOOR (6 results) D ['18778338', '12208243'], patient with AZOOR (27 results) D ['19684865', '18778338', '18547281', '18439564', '17846358', '12208243'], patient with AZOOR (27 results) S ['20661173'], AZOOR (36 results) D ['19684865', '18778338', '18547281', '18439564', '17846358'], AZOOR (36 results) S ['20661173'], intact ELM (5 results) D ['20472291']

PMC3072155	6	FALSE	10	FALSE		4	16	TRUE	7	TRUE	7	TRUE	5	TRU
PMC3062546	6	FALSE	16	FALSE		7	22	FALSE	22	TRUE	21	TRUE	19	TRU
PMC3077807	0	TRUE	1	FALSE		1	1	FALSE	1	FALSE	1	FALSE	1	FAL
PMC3076118	10	FALSE	20	FALSE		7	28	TRUE	26	TRUE	26	TRUE	22	TRU

JE	TRUE	4	TRUE	Denmark region (31 results) D ['20865114'], LABKA system (3 results) D ['2772556'], LABKA system (3 results) S ['21487452'], laboratory information system (314 results) S ['21487452'], LABKA data (3 results) D ['2772556'], LABKA data (3 results) S ['21487452'], LABKA research (2 results) S ['21487452'], research database (1327 results) S ['21487452'], North Denmark region (19 results) D ['20865114'], laboratory information (2271 results) S ['21487452'], LABKA (3 results) D ['2772556'], LABKA (3 results) S ['21487452'], LABKA research database (2 results) S ['21487452'], LABKA database (2 results) S ['21487452'], Central Denmark (16 results) S ['21487452'], NPU (241 results) D ['19745311']
JE	TRUE	10	TRUE	antituberculosis drug resistance (71 results) S ['21427397', '19375159'], new era for antituberculosis (41 results) D ['19861002'], new era for antituberculosis (41 results) S ['21427397'], drug resistance surveillance (106 results) S ['21427397', '19375159'], antituberculosis drug resistance surveillance (10 results) S ['21427397'], treatment with mono-resistance (18 results) D ['20101802'], Health Union Against Tuberculosis (277 results) D ['20819252'], Global Project on Anti-Tuberculosis (25 results) D ['16032561'], Global Project on Anti-Tuberculosis (25 results) S ['19375159', '18369201'], era for antituberculosis (146 results) D ['19861002'], era for antituberculosis (146 results) S ['21427397'], resistance surveillance (448 results) S ['21427397'], antituberculosis (3507 results) S ['21427397'], drug-resistant tuberculosis (1227 results) S ['21427397'], Anti-Tuberculosis Drug Resistance Surveillance (21 results) D ['16032561'], Anti-Tuberculosis Drug Resistance Surveillance (21 results) S ['19375159', '18369201'], Routine surveillance (815 results) S ['21427397'], Project on Anti-Tuberculosis (50 results) S ['19375159', '18369201'], surveillance activities (459 results) S ['21427397'], antituberculosis drug (343 results) S ['21427397'], Anti-Tuberculosis Drug Resistance (90 results) S ['19375159'], drug resistance Global Project (70 results) S ['21427397', '19375159', '18369201']
.SE	TRUE	1	TRUE	Clin Neuropsychol (675 results) S ['21491347']
JE	TRUE	17	TRUE	Postkeratoplasty (230 results) S ['21499568'], DALK (107 results) S ['21499568'], suture removal (552 results) S ['21499568'], techniques on astigmatism (4665 results) S ['21499568'], patients after DALK (53 results) S ['21499568'], lamellar keratoplasty (859 results) S ['21499568'], suture adjustment versus (16 results) D ['9820936', '2008275'], anterior lamellar (779 results) S ['21499568'], continuous suture adjustment versus (2 results) D ['2008275'], keratoconus patients (232 results) S ['21499568'], anterior lamellar keratoplasty (194 results) S ['21499568'], suture adjustment (64 results) D ['17133048'], Single continuous suture adjustment (2 results) D ['9894932', '2008275'], underwent DALK (43 results) S ['21499568'], suture adjustment versus selective (3 results) D ['2008275'], suture (52163 results) S ['21499568'], astigmatism (7539 results) S ['21499568'], keratoconus patients after DALK (33 results) S ['21499568'], deep anterior lamellar keratoplasty (161 results) S ['21499568'], keratoconus (3448 results) S ['21499568'], lamellar (12498 results) S ['21499568'], regularity of postkeratoplasty (11 results) D ['14597524'], astigmatism after suture (577 results) S ['21499568'], continuous suture adjustment (2 results) D ['9894932', '2008275'], keratoplasty (7704 results) S ['21499568'], postkeratoplasty astigmatism (102 results) D ['17133048', '14597524'], postkeratoplasty astigmatism (102 results) S ['21499568'], Single continuous suture (13 results) D ['9894932', '2008275'], Postkeratoplasty astigmatism control (32 results) D ['8506906'], groups after DALK (13 results) S ['21499568']

PMC3072221	3	FALSE	14	FALSE		6	16	TRUE	15	TRUE	12	TRUE	9	TRU
PMC3068859	9	FALSE	1	FALSE		10	10	TRUE	6	TRUE	6	TRUE	4	TRU
PMC3073238	2	FALSE	7	FALSE		2	8	TRUE	7	TRUE	6	TRUE	4	TRU
PMC3078837	5	FALSE	6	FALSE		3	9	TRUE	8	TRUE	8	TRUE	7	TRU
PMC3075999	16	FALSE	10	FALSE		11	24	TRUE	21	TRUE	17	TRUE	16	TRU

JE	TRUE	5	TRUE	derogation (126 results) S ['21432689'], pDMPFC (1 results) S ['21432689'], Inhibitory intentional (44 results) S ['19426813'], attractive alternatives on trials (66 results) S ['21432689'], role of romantic (190 results) S ['21432689'], romantic relationship (193 results) S ['21432689'], regulation predicts affective (13 results) D ['16702153'], romantic relationship status (8 results) S ['18453390'], attractive alternatives (233 results) S ['21432689'], RVLPFC (7 results) S ['21432689', '17576282'], emotion regulation predicts affective (5 results) D ['16702153'], emotion divergent consequences (12 results) D ['9457784'], derogation effect (13 results) S ['21432689'], deliberate emotion regulation (29 results) S ['21432689'], attractive opposite-sex (8 results) S ['18453390'], disrupts amygdala activity (32 results) S ['17576282'], neural activation for trials (574 results) S ['21432689']
JE	TRUE	2	TRUE	histamine pathway (13 results) S ['21104347'], HNMT levels (28 results) D ['19178400', '19025430', '17985251'], Thr105Ile (20 results) D ['19773194', '19025430', '18543121', '17985251'], Thr105Ile polymorphism (7 results) D ['19025430', '18543121', '17985251'], H4R (67 results) D ['19547708', '19153539'], acute phase of Mycobacterium (143 results) D ['19822651'], nonsynonymous Thr105Ile polymorphism (4 results) D ['18543121', '17985251'], Th1 effector cell responses (2 results) D ['14982842'], nonsynonymous Thr105Ile (4 results) D ['18543121', '17985251'], histamine N-methyltransferase (421 results) D ['19773194', '19178400']
JE	TRUE	4	TRUE	verbal processing system (697 results) S ['21462011'], right posterior negativity (4 results) S ['21462011'], anterior negativities (18 results) S ['21462011'], quantitative manipulation (8 results) S ['21462011'], language-dominant linguistical (1 results) D ['8498198'], Cortical DC-potentials (13 results) D ['8498198'], negativities (220 results) S ['21462011'], arithmetic problems (125 results) S ['21462011'], quantitative manipulation system (222 results) S ['21462011']
JE	TRUE	4	TRUE	workplace health (438 results) S ['21453552'], workplace health assessment (2295 results) S ['21453552'], PDACT (1 results) D ['19157967'], construction workers (945 results) S ['21453552'], workplace health assessment pilot (74 results) S ['21453552'], compensation costs among construction (42 results) D ['19749601'], evaluation of PDACT (1 results) D ['19157967'], handheld devices (112 results) S ['21453552'], trial data collection (5 results) D ['19157967'], construction workplace health assessment (60 results) S ['21453552'], Seebregts (20 results) D ['19157967']
JE	TRUE	8	TRUE	interproximal caries lesions (5 results) D ['19891346'], caries lesions (934 results) S ['21494394'], nanocomplexes on approximal (1 results) D ['18446025'], enamel subsurface (38 results) D ['20090326', '18435723'], sugar-free gum (47 results) D ['20090326', '18446025'], CPP-ACP (84 results) S ['21494394'], CPP-ACP nanocomplexes on approximal (1 results) D ['18446025'], Review Caries infiltration (9 results) D ['19894293', '19891346'], Review Caries infiltration (9 results) S ['21494394'], anticariogenic effect of sugar-free (2 results) D ['18446025'], caries-sealing (409 results) S ['21494394'], resin infiltration (96 results) D ['20090328', '19894293'], resin infiltration (96 results) S ['21494394'], subsurface lesions (95 results) D ['20090326', '18782377'], progression of enamel (424 results) S ['21344828'], control half-slabs (4 results) D ['20090326', '18435723'], enamel interproximal caries (61 results) D ['19891346'], caries progression (239 results) D ['20090328'], caries progression (239 results) S ['21494394', '21344828'], aim of resin (3190 results) S ['21494394'], caries (41229 results) S ['21494394'], non-cavitated (133 results) S ['21494394', '21344828'], partial carious dentin removal (6 results) D ['20123388'], enamel subsurface lesions (19 results) D ['20090326', '18435723', '12598550'], infiltration of interproximal (6 results) D ['19891346'], partial carious dentin (23 results) D ['20123388']

PMC3075996	1	FALSE	12	FALSE		2	13	FALSE	13	FALSE	13	FALSE	13	TRU
PMC3075993	2	FALSE	9	FALSE		2	11	TRUE	10	TRUE	10	TRUE	8	TRU
PMC3075991	5	FALSE	1	FALSE		5	6	FALSE	6	TRUE	4	TRUE	3	TRU
PMC3076672	1	FALSE	9	FALSE		2	10	FALSE	10	FALSE	10	TRUE	8	TRU
PMC3058709	3	FALSE	14	FALSE		4	17	TRUE	16	TRUE	16	TRUE	14	TRU
PMC3058702	8	FALSE	12	FALSE		6	19	TRUE	14	TRUE	12	TRUE	9	TRU

JE	TRUE	10	TRUE	hybrid composite resin (95 results) D ['18768000'], hybrid composite resin (95 results) S ['21494382'], carbamide (1051 results) S ['21494382'], composite resin (5412 results) S ['21494382'], human enamel (1220 results) S ['21494382'], procedure with carbamide (25918 results) S ['21494382'], carbamide substance (826 results) S ['21494382'], Peroxide Carbamide Specimens (233 results) S ['21494382'], upper jaw for days (257 results) S ['21494382'], resin surface alterations (95 results) S ['21494382'], Carbamide Specimens (1187 results) S ['21494382'], carbamide peroxide (830 results) S ['21494382'], Effects of carbamide (54691 results) S ['21494382']
JE	TRUE	8	TRUE	King Saud (4607 results) S ['21494387'], King Saud University (4421 results) S ['21494387'], Saud University (4431 results) S ['21494387'], physician attire (16 results) D ['3783905'], Maha AlSarheed (3 results) S ['21494387'], Pediatric Dentistry (10148 results) S ['21494387'], officer attitudes (12 results) D ['3783905'], Fear of dentists (440 results) S ['21494387'], Riyadh (10880 results) S ['21494387'], College of Dentistry (5439 results) S ['21494387'], Saud (4658 results) S ['21494387']
JE	TRUE	1	TRUE	fluoride intake of 6-month (10 results) D ['4050730', '7355802'], brands of chocolate (26 results) D ['14762499'], Market Dental fluorosis (17 results) D ['4050730'], fluoride (31804 results) S ['21494379'], experimental study of bio-availability (7 results) D ['3459412'], several brands of chocolate (26 results) D ['14762499']
JE	TRUE	6	TRUE	hemangioma size on pain (106 results) S ['21383624'], hepatic cavernous (706 results) S ['21383624'], hepatic hemangiomas (262 results) S ['21383624'], hemangioma size (18 results) D ['17914976'], hemangioma size (18 results) S ['21383624'], cavernous hemangioma (5979 results) S ['21383624'], hepatology clinic (61 results) S ['21383624'], single lesion (808 results) S ['21383624'], general hepatology clinic (372 results) S ['21383624'], single giant hemangioma (31 results) S ['21383624']
JE	TRUE	8	TRUE	adrenal development (118 results) D ['20660055'], adrenal development (118 results) S ['21163858', '18984668'], human fetal adrenal development (3 results) S ['18984668'], Ang2 (233 results) S ['21163858'], ANGPT2 promoter (35 results) S ['21163858'], human fetal adrenal (232 results) D ['18364383'], human fetal adrenal (232 results) S ['18984668'], adrenal gland Developmental (1008 results) S ['21163858'], reveals angiopoietin (24 results) S ['21163858'], analysis reveals angiopoietin (17 results) S ['21163858'], NCI-H295R human adrenal cells (89 results) S ['21163858', '18984668'], ANGPT2 (66 results) S ['21163858'], adrenocortical cells (1752 results) S ['21163858'], target of steroidogenic (368 results) S ['21163858'], novel target of steroidogenic (56 results) S ['21163858'], fetal adrenal development (17 results) S ['18984668'], definitive zone (69 results) D ['18364383']
JE	TRUE	1	TRUE	hepatocytes ASM (11 results) D ['19074137'], hepatocytes ASM (11 results) S ['21163859'], Acid sphingomyelinase regulates (50 results) D ['19074137'], Acid sphingomyelinase regulates (50 results) S ['21163859'], ASM on glucose (9 results) D ['19074137'], ASM on glucose (9 results) S ['21163859'], sphingomyelinase regulates glucose (3 results) D ['19074137'], sphingomyelinase regulates glucose (3 results) S ['21163859'], sphingomyelinase regulates (81 results) D ['19074137'], sphingomyelinase regulates (81 results) S ['21163859'], Ad5ASM (1 results) S ['21163859'], ASM deficiency causes (67 results) D ['19074137', '18567738'], Exogenous ASM (31 results) S ['21163859'], protein kinase suppression (2 results) S ['21163859'], glycogen deposition (425 results) S ['21163859'], DN-AMPK (14 results) S ['18220291'], Acid sphingomyelinase regulates glucose (3 results) D ['19074137'], Acid sphingomyelinase regulates glucose (3 results) S ['21163859'], S1P formation (21 results) D ['12069819'], S1P formation (21 results) S ['15946935']

PMC3049876	5	FALSE	5	FALSE		6	8	TRUE	6	TRUE	4	TRUE	3	TRU
PMC3071972	11	FALSE	2	FALSE		6	13	TRUE	8	TRUE	6	TRUE	5	TRU
PMC3072528	3	FALSE	4	FALSE		4	7	TRUE	5	TRUE	5	TRUE	3	TRU
PMC3072165	8	FALSE	12	FALSE		8	17	TRUE	15	TRUE	12	TRUE	7	TRU
PMC3069233	0	TRUE	9	FALSE		4	8	TRUE	5	TRUE	4	TRUE	3	TRU

JE	TRUE	0	FALSE	Hirotani Nakayama (7 results) D ['15158121', '12672819', '11851362', '11815436'], oxidase-4 mediates (10 results) S ['20921387'], Higuchi Otsu (25 results) D ['15158121', '12672819', '11869860', '11851362', '11815436'], mediates protection against chronic (29 results) S ['20921387'], Matsumura Ueno Tada Hori (3 results) D ['11851362', '11815436'], NADPH oxidase-4 mediates protection (1 results) S ['20921387'], oxidase-4 mediates protection (1 results) S ['20921387'], NADPH oxidase-4 mediates (10 results) S ['20921387'], Yamaguchi Hikoso (21 results) D ['15158121', '12672819'], Tada Hori (75 results) D ['11869860', '11851362', '11815436']
JE	TRUE	2	TRUE	only multisensory integration elicits (2 results) D ['19820918'], integration elicits (102 results) D ['19820918'], Hands only multisensory integration (14 results) D ['19820918'], non-corporeal objects (4 results) D ['19820918'], multisensory integration elicits (4 results) D ['19820918'], elicits sense of ownership (2 results) D ['19820918'], multisensory integration elicits sense (2 results) D ['19820918'], elicits sense (80 results) D ['19820918'], rubber hand sensitivity (46 results) D ['17317221'], Hands only multisensory (33 results) D ['19820918'], integration elicits sense (6 results) D ['19820918'], Ehrsson (158 results) S ['19400438', '19074189', '19050755'], higher-order somatosensory areas (6 results) S ['16336049']
JE	TRUE	1	TRUE	bTBI (12 results) S ['21441982', '19397421'], astrocytic production of interleukin-6 (17 results) D ['11784794'], Agoston (338 results) S ['21441982', '20674607'], chronic astrocytic production (26 results) D ['11784794'], Interleukin-6 Vallieres (5 results) D ['11784794'], Front Neurol (72 results) S ['21441982'], Kamnaksh (2 results) S ['21441982']
JE	TRUE	1	TRUE	Salierno Etchenique (5 results) D ['19506708'], Fino Araya Peterka (1 results) D ['19506708'], modeler's view (3 results) S ['20685378'], long-range patchy (29 results) D ['16855096'], long-range patchy (29 results) S ['20685378', '19866352'], local connectivity between pyramidal (94 results) D ['20550566'], reliable intermediate-range (7 results) S ['19225575'], reliable intermediate-range projections (1 results) S ['19225575'], patchy connections (18 results) S ['20685378', '19866352'], intrinsic horizontal connectivity (33 results) D ['16855096'], intrinsic horizontal connectivity (33 results) S ['20685378'], patchy horizontal connectivity (7 results) D ['16855096'], patchy horizontal connectivity (7 results) S ['20685378', '19866352'], intermediate-range projections (1 results) S ['19225575'], current view on cortical (430 results) S ['20685378'], horizontal connectivity (12 results) D ['16855096'], horizontal connectivity (12 results) S ['20685378'], neocortical networks (87 results) S ['20685378'], long-range patchy connections (15 results) D ['16855096', '1542406', '6954508'], long-range patchy connections (15 results) S ['20685378', '19866352']
JE	TRUE	2	TRUE	mediastinal gray (136 results) S ['21454882', '20971819'], PMLBCL (18 results) S ['15322489'], mediastinal gray zone (9 results) S ['21454882', '20971819', '16224207', '15322489'], hypermethylated (1533 results) S ['21454882'], MGZL (4 results) S ['16224207', '15322489'], MGZL shows (4 results) S ['16224207', '15322489'], epigenetic profile (54 results) S ['21454882'], cases of MGZL (2 results) S ['16224207'], profile of MGZL (1 results) S ['15322489']

PMC3069222	14	FALSE	9	FALSE		9	23	TRUE	17	TRUE	16	TRUE	11	TRU
PMC3069221	1	FALSE	1	FALSE		3	2	FALSE	2	FALSE	2	FALSE	2	FAL
PMC3069220	2	FALSE	5	FALSE		5	5	FALSE	5	TRUE	4	TRUE	4	TRU
PMC3058615	1	FALSE	17	FALSE		3	17	TRUE	16	TRUE	16	TRUE	14	TRU
PMC3056084	3	FALSE	6	FALSE		6	9	TRUE	7	TRUE	5	TRUE	4	TRU
PMC3077574	1	FALSE	9	FALSE		2	10	TRUE	8	TRUE	8	TRUE	7	TRU

JE	TRUE	3	TRUE	Monosomal karyotype (14 results) D ['21072042', '20562328', '20553926'], Monosomal karyotype (14 results) S ['21454879', '18695255'], patients with MK-AML (20 results) D ['20562328', '20553926'], patients with MK-AML (20 results) S ['18695255'], remission acute myeloid leukemia (8 results) D ['17213292'], prognostic subgroup of AML (102 results) D ['20553926'], prognostic subgroup of AML (102 results) S ['21410371'], myeloid determination of prognostic (2127 results) S ['21410371'], Monosomal (41 results) D ['21072042', '20562328', '20553926'], Monosomal (41 results) S ['21454879', '18695255'], impact of monosomal (5 results) D ['20562328'], impact of monosomal (5 results) S ['18695255'], versus no-donor analysis (67 results) D ['17213292'], MK-AML (30 results) D ['20562328', '20553926'], MK-AML (30 results) S ['18695255'], donor versus no-donor analysis (67 results) D ['17213292'], no-donor analysis of myeloablative (6 results) D ['19509382', '17213292'], consolidation treatment for poor-risk (93 results) D ['19509382'], consolidation treatment for poor-risk (93 results) S ['21220605'], remission acute myeloid (11 results) D ['17213292'], no-donor analysis (5 results) D ['17213292'], Prognostic impact of monosomal (5 results) D ['20562328'], Prognostic impact of monosomal (5 results) S ['18695255']
.SE	TRUE	2	TRUE	effects on HbF (420 results) D ['20378564'], hydroxycarbamide (117 results) S ['21454878', '21131035']
JE	TRUE	3	TRUE	HFE (2425 results) S ['21408141'], inadequate production of hepcidin (9 results) D ['17724144'], hepcidin Round Robin (1 results) S ['19996119'], hereditary hemochromatosis (1303 results) S ['21454877'], hepcidin Round (1 results) S ['19996119'], TFR2 patients (91 results) D ['20471131'], hemochromatosis (8013 results) S ['21454877']
JE	TRUE	7	TRUE	sulcal depth (31 results) S ['21391248'], CAE group (25 results) S ['21391248'], cortical morphometry measures (284 results) S ['21391248'], mesial superior frontal region (74 results) D ['18266751'], sulcal regions (23 results) S ['21391248', '16269250'], age-cortical morphometry (566 results) S ['21391248'], sulcal (1007 results) S ['21391248'], cortical morphometry (14 results) S ['21391248'], morphometry measures (884 results) S ['21391248'], gray matter tissue thickness (62 results) S ['21391248'], depth of sulcal (65 results) S ['21391248'], children with CAE (131 results) S ['21391248'], matter tissue thickness (254 results) S ['21391248'], PIQ (325 results) S ['21391248'], variable brain-cognition relationships (1 results) S ['21391248'], PIQ scores (25 results) S ['21391248'], variable brain-cognition (2 results) S ['21391248'], sulcal depth with age (16 results) S ['21391248']
JE	TRUE	3	TRUE	NIMH R01 MH072681 (12 results) S ['21391254', '17329470', '15955488'], auditory oddball (605 results) S ['21391254'], Review Spontaneous low-frequency (68 results) D ['17556752'], Granger causality reveals distinct (4 results) S ['19245841'], Granger causality reveals (18 results) S ['19245841'], Review Spontaneous low-frequency fluctuations (6 results) D ['17556752'], DMN (1275 results) S ['21391254'], default modes (5 results) D ['12958209'], lateral differences (135 results) S ['21391254']
JE	TRUE	5	TRUE	hospital-onset incidence rates (13 results) S ['21460483'], metrics for multidrug-resistant (2 results) D ['18808340'], metrics for multidrug-resistant (2 results) S ['21460483'], Recommendations for metrics (123 results) S ['21460483'], hospital-onset incidence of MRSA (21 results) S ['21460483'], nosocomial infection measure (1385 results) S ['21460483'], MRSA infection (1100 results) S ['21460483'], multidrug-resistant organisms (205 results) S ['21460483'], denominator (3138 results) S ['21460483'], hospital-onset incidence (33 results) S ['21460483']

PMC3076501	3	FALSE	13	FALSE		3	13	TRUE	11	TRUE	10	TRUE	8	TRU
PMC3075480	0	TRUE	7	FALSE		2	4	FALSE	4	TRUE	3	TRUE	3	TRU
PMC3071598	3	FALSE	22	FALSE		7	23	TRUE	21	TRUE	20	TRUE	20	TRU
PMC3059541	3	FALSE	16	FALSE		7	13	TRUE	10	TRUE	9	TRUE	9	TRU
PMC3059540	4	FALSE	4	FALSE		6	8	TRUE	7	TRUE	6	TRUE	4	TRU

JE	TRUE	4	TRUE	bmMSCs (81 results) S ['21494428'], bone marrow mesenchymal stromal (85 results) S ['21494428'], supernatant of bmMSCs (1 results) S ['21494428'], mesenchymal stromal cells (916 results) S ['21494428'], pbMSCs (7 results) D ['16514623'], supernatant of bone (1262 results) S ['21494428'], marrow mesenchymal stromal cells (74 results) S ['21494428'], ipbMSCs (1 results) S ['21494428'], pbMNCs (246 results) S ['21494428'], marrow mesenchymal stromal (89 results) S ['21494428'], isolation of bmMSCs (4 results) S ['21494428'], differentiation of pbMSCs (4 results) D ['16514623'], mesenchymal stromal (1039 results) S ['21494428'], combination with ipbMSCs (1 results) S ['21494428'], stromal morphology (18 results) D ['11056678'], stromal morphology (18 results) S ['21494428']
JE	TRUE	1	TRUE	angiogenesis with microbubbles (103 results) S ['21343825'], CA134659 (8 results) S ['21343825'], tumor angiogenesis with microbubbles (57 results) S ['21343825'], 1R43CA137913 (1 results) S ['21343825'], Microultrasound molecular (1 results) S ['18092513'], cRGD-MB (1 results) S ['21343825'], robust microbubble (27 results) S ['21343825']
JE	TRUE	9	TRUE	farmworker knowledge (34 results) S ['21462026', '17291182'], eye injury prevention program (62 results) D ['20208215', '17135138', '15551366'], eye protection among farmworkers (3 results) S ['21462026', '18479993', '11440328'], farmworkers (385 results) S ['21462026'], farmworker risk (72 results) S ['21462026'], eye protection (487 results) S ['21462026'], protection among farmworkers (24 results) S ['21462026', '18479993'], farmworkers believe (1 results) S ['21462026'], many farmworkers (3 results) S ['21462026'], farmworker use of eye (2 results) S ['21462026'], ocular protection (67 results) S ['18479993'], protection use (60 results) S ['21462026'], farmworker use (217 results) S ['21462026'], Carolina Farmworkers (95 results) S ['21462026'], North Carolina Farmworkers (94 results) S ['21462026'], eye safety (69 results) D ['20208215', '17135138'], eye safety (69 results) S ['21462026', '18479993'], Florida migrant community (40 results) D ['20208215'], eye injuries (12210 results) S ['21462026'], knowledge about eye (5788 results) S ['21462026'], eye health (578 results) S ['21462026'], beliefs about eye (13111 results) S ['21462026'], eye protection use (9 results) S ['21462026', '11440328'], farmworker (217 results) S ['21462026'], risk beliefs about eye (565 results) S ['21462026']
JE	TRUE	5	TRUE	DNA aptamers (275 results) S ['21455479'], soman derivative cross-react (1 results) S ['19051203'], soman derivative (25 results) S ['19051203'], rhGH aptamers (2 results) S ['21455479'], aptamers (2765 results) S ['21455479'], aptamers for cytochemical (1 results) S ['18311508'], human growth hormone (14181 results) S ['21455479'], methylphosphonic acid core (6 results) S ['19051203'], spectrometrical analysis of recombinant (1 results) D ['15707495'], DNA aptamers for cytochemical (1 results) S ['18311508'], novel kDa hGH-N (3 results) D ['8920950'], growth hormone reveals amino (93 results) D ['16372270', '15707495'], rhGH (1276 results) S ['21455479'], conjugate opsonins (75 results) S ['18671260'], DNA aptamer-Fc conjugate opsonins (1 results) S ['18671260'], aptamer-Fc conjugate opsonins (1 results) S ['18671260'], human growth (15418 results) S ['21455479'], derivative cross-react (64 results) S ['19051203'], cytochemical detection of acetylcholine (4 results) S ['18311508']
JE	TRUE	3	TRUE	Trizol extracts (7 results) D ['19382428', '18803417'], concurrent extraction of RNA (17 results) D ['16970899'], concurrent extraction of RNA (17 results) S ['21455480'], Trizol (291 results) S ['21455480'], Trizol fractions (2 results) D ['16556310'], cell line Trizol (42 results) D ['17489233'], DNA fraction (382 results) S ['21455480'], nuclear proteins (61420 results) S ['21455480']

PMC3059538	0	TRUE	15	FALSE		2	15	TRUE	12	TRUE	11	TRUE	9	TRU
PMC3059537	3	FALSE	3	FALSE		2	6	TRUE	3	TRUE	3	TRUE	3	TRU
PMC3059536	13	FALSE	5	FALSE		9	16	TRUE	14	TRUE	13	TRUE	12	TRU
PMC3059535	6	FALSE	10	FALSE		5	13	TRUE	12	TRUE	10	TRUE	4	TRU

JE	TRUE	5	TRUE	background proteins (99 results) S ['21455478'], Facilities Proteomics (2 results) S ['17513294'], Resource Facilities Proteomics (2 results) S ['17513294'], PRG (367 results) S ['21455478'], complex mixtures of background (20263 results) S ['21455478'], quantitative proteomics (811 results) S ['21455478'], participants ABRF (10 results) S ['21455478'], Facilities Proteomics Research Group (13 results) S ['21455478', '17513294'], Facilities Proteomics Research (56 results) S ['21455478'], PRG study (183 results) S ['21455478'], Biomolecular Resource Facilities Proteomics (2 results) S ['17513294'], Resource Facilities Proteomics Research (12 results) S ['21455478', '17513294'], Biomolecular Resource Facilities (29 results) S ['21455478', '17513294'], Resource Facilities (43 results) S ['21455478', '17513294'], Proteomics Research (616 results) S ['21455478']
JE	TRUE	3	TRUE	metabolome (1555 results) S ['21455475'], gene expression dynamics inspector (5 results) D ['18173289'], expression dynamics inspector (5 results) D ['18173289'], negative TOF-MS (310 results) S ['21455475'], USA Metabolomics (701 results) S ['21455475'], dynamics inspector (5 results) D ['18173289']
JE	TRUE	5	TRUE	perinatal cards (1 results) D ['16645211'], blood spots on perinatal (33 results) D ['19551829', '16645211'], forensic filter step (5 results) S ['21455476'], retrospective diagnosis (381 results) D ['20570211'], serosurvey of cytomegalovirus (6 results) D ['16957061'], human congenital cytomegalovirus infection (3 results) D ['16645211'], spot filter cards (29 results) D ['20570211', '12589833'], spots on perinatal (48 results) D ['19551829'], Clin Virol (26 results) D ['19781984'], DNA extraction (3358 results) S ['21455476'], blood spots for retrospective (132 results) D ['19551829'], Cytomegalovirus DNA detection (15 results) D ['19781984', '10996112'], National serosurvey of cytomegalovirus (1 results) D ['16957061'], allele Ann Med (79 results) S ['20092400'], DNA extraction method (266 results) S ['21455476'], sensitivity of HCMV-DNA (98 results) D ['20570211', '16645211'], extraction methods (2330 results) S ['21455476'], blood spot filter cards (27 results) D ['20570211', '12589833']
JE	TRUE	4	TRUE	percent of phosphopeptides (8 results) D ['17322306'], confident identification of phosphopeptides (13 results) D ['18318008'], confident identification of phosphopeptides (13 results) S ['21455477'], SIMAC elution (1 results) D ['18039691'], database search engine analysis (172 results) S ['21455477'], DIMAC method (8 results) S ['21455477'], DIMAC enrichment (1 results) S ['21455477'], phosphopeptide identifications (17 results) D ['17874083'], phosphopeptide identifications (17 results) S ['21455477'], phosphopeptide (2779 results) S ['21455477'], DIMAC enrichment strategy (1 results) S ['21455477'], phosphopeptides (3738 results) S ['21455477'], identification of phosphopeptides (623 results) S ['21455477'], DIMAC (16 results) S ['21455477'], SIMAC method (16 results) D ['18039691'], phosphoproteomics strategy (5 results) D ['18039691']

PMC3049901	8	FALSE	24	FALSE		15	30	TRUE	22	TRUE	19	TRUE	16	TRU
PMC2896988	2	FALSE	13	FALSE		8	15	TRUE	9	TRUE	6	TRUE	4	TRU
PMC3071432	0	TRUE	8	FALSE		3	8	TRUE	6	TRUE	5	TRUE	4	TRU
PMC3076284	14	FALSE	4	FALSE		15	18	TRUE	15	TRUE	13	TRUE	11	TRU
PMC3053430	3	FALSE	8	FALSE		9	10	TRUE	7	TRUE	7	TRUE	6	TRU

JE	TRUE	6	TRUE	larger PSDs (30 results) S ['21280048'], MPTP-treated (888 results) S ['21280048'], PSD perforations (11 results) D ['6957887'], PSD perforations (11 results) S ['21280048'], vGluT1-containing (10 results) S ['21280048', '18380666', '16977615', '16538684'], glutamatergic axo-spinous (6 results) S ['21280048', '18380666'], axo-spinous synapses (29 results) S ['21280048', '18380666'], thalamostriatal (133 results) D ['18971464'], thalamostriatal (133 results) S ['21280048', '20082980'], thalamostriatal glutamatergic (31 results) D ['18971464', '18562619', '16226840'], thalamostriatal glutamatergic (31 results) S ['21280048', '20082980', '18805468', '18380666', '16977615'], axo-spinous thalamostriatal (3 results) S ['21280048', '18380666'], corticostriatal (1182 results) S ['21280048'], axo-spinous (61 results) S ['21280048', '18380666'], thalamic boutons (334 results) S ['18380666'], vGluT2-positive (29 results) D ['18971464'], vGluT2-positive (29 results) S ['21280048', '16977615'], corticostriatal glutamatergic system (4 results) S ['21280048'], thalamostriatal system (7 results) S ['18805468', '18380666', '15331233'], striatal spine (90 results) D ['20197093', '20118184', '20105237'], striatal spine (90 results) S ['21280048', '20082980'], parkinsonian monkeys (167 results) S ['21280048', '20138865', '20082980'], pattern of multisynaptic (31 results) S ['21280048'], glutamatergic axo-spinous synapses (5 results) S ['21280048', '18380666'], striatofugal neurons (12 results) S ['20082980'], thalamostriatal synapses (3 results) D ['18971464', '18562619'], multisynaptic connectivity (10 results) S ['21280048'], vGluT2-positive axo-spinous synapses (1 results) S ['21280048'], properties of thalamostriatal (4 results) D ['18562619'], thalamostriatal glutamatergic systems (6 results) D ['18562619'], thalamostriatal glutamatergic systems (6 results) S ['21280048', '18805468', '18380666'], vGluT2-positive axo-spinous (1 results) S ['21280048']
JE	TRUE	1	TRUE	actual similarity level (56 results) S ['19147902'], CBIR-based (2 results) S ['20204448'], reference ROIs (3 results) S ['19147902'], tomography JAFROC (10 results) D ['19000867'], visual similarity of breast (19 results) D ['17278499'], visual similarity of breast (19 results) S ['19147902', '17659237', '16485416'], image retrieval schemes (2 results) S ['19147902'], CBIR scheme (3 results) S ['18072498'], CBIR (84 results) S ['20204448'], retrieval schemes (8 results) S ['19147902'], CAD scheme (103 results) S ['19201355'], similar breast (37 results) S ['19147902'], diagnosis environment (3 results) S ['16485416'], reference database of ROIs (9 results) S ['20204448', '19147902'], true-positive ROIs (3 results) S ['20204448']
JE	TRUE	2	TRUE	more naturalistic auditory scenes (2 results) S ['20000928'], Gygi (336 results) S ['21355664'], Centroid Velocity (2 results) S ['18344871'], Incongruency advantage (6 results) S ['21355664'], points better identification accuracy (26 results) S ['21355664'], target sounds (49 results) S ['20000928'], environmental sounds (377 results) S ['21355664'], percentage points better identification (11 results) S ['21355664']
JE	TRUE	4	TRUE	FLT3 downstream pathways (47 results) D ['18192505'], FLT3 downstream pathways (47 results) S ['21453545'], FLT3-TKD mutations (11 results) D ['15769897', '15718420', '12036858'], cocultures with FLT3 (93 results) D ['18955566'], MLN518 CEP-701 (7 results) D ['19654408'], Lestaurtinib trials (21 results) D ['15126317'], FLT3 (2677 results) S ['21453545'], FLT3-ITD expression (5 results) D ['14981546'], such first-generation FLT3 (3 results) D ['20809224', '19654408'], FLT3 activation (28 results) D ['17655729'], FLT3 activation (28 results) S ['21453545'], FLT3 inhibitors (105 results) D ['20809224'], FLT3-ITD mutations (88 results) D ['20807885'], such first-generation FLT3 inhibitors (3 results) D ['20809224', '19654408'], patients show FLT3 phosphorylation (101 results) D ['19654408'], FLT3 mutations (218 results) D ['21067377'], FLT3-ITD cells (16 results) D ['18192505'], FLT3-ITD cells (16 results) S ['17050201', '16432846']
JE	TRUE	3	TRUE	high-salt diet (1401 results) S ['21346625'], urinary angiotensinogen levels (4 results) S ['18353869'], intrarenal angiotensinogen contributes (13 results) S ['15888567'], renal AGT mRNA (39 results) D ['20462965'], renal AGT mRNA (39 results) S ['17634399'], Angiotensinogen depletion (4 results) D ['7759857'], ACE inhibition effects (5 results) S ['1317125'], salt-induced (1790 results) S ['21346625'], intrarenal angiotensinogen (24 results) S ['17634399', '17200690', '15888567', '11849399'], angiotensinogen (4048 results) S ['21346625'], tonic stimulation of angiotensinogen (1 results) D ['7759857']

PMC2905487	6	FALSE	2	FALSE		4	5	TRUE	3	TRUE	2	TRUE	2	TRU
PMC2889239	0	TRUE	3	FALSE		1	3	FALSE	3	FALSE	3	FALSE	3	TRU
PMC3069719	5	FALSE	17	FALSE		10	22	TRUE	21	TRUE	20	TRUE	19	TRU
PMC3072141	4	FALSE	1	FALSE		5	5	FALSE	5	TRUE	3	TRUE	3	TRU
PMC3066587	13	FALSE	7	FALSE		4	13	TRUE	5	TRUE	3	TRUE	1	TRU
PMC3066586	1	FALSE	10	FALSE		2	6	FALSE	6	FALSE	6	FALSE	6	TRU
PMC3066584	13	FALSE	1	FALSE		9	11	TRUE	10	TRUE	7	TRUE	4	TRU

JE	TRUE	0	FALSE	migrants' discourses of health (1 results) D ['14574068'], spiritual factors more frequently (30 results) S ['20131000'], South Asian womens' views (1 results) D ['14528550'], Asian womens' views (1 results) D ['14528550'], South Asian womens' (2 results) D ['14528550'], older Sikh (7 results) D ['16671279'], spiritual factors (75 results) S ['20131000'], older Sikh women (4 results) D ['16671279']
JE	TRUE	0	FALSE	nativity for mortality (59 results) S ['19621260'], English language use (22 results) S ['19621260'], 12-year mortality (30 results) S ['19621260']
JE	TRUE	8	TRUE	fast spin-echo (2137 results) S ['21448939'], three-dimensional isotropic resolution sequences (32 results) S ['19011194'], spin-echo sequences (1135 results) S ['21448939'], intermediate-weighted (80 results) D ['19789228'], continuous slices through joints (11 results) S ['21448939'], fast spin-echo sequences (215 results) S ['21448939'], three-dimensional isotropic (38 results) D ['19188318', '19155413'], three-dimensional isotropic (38 results) S ['19703886'], sequences with isotropic (260 results) S ['21448939'], isotropic resolution (249 results) S ['21448939'], reformat images (7 results) S ['21448939'], resolution fast spin-echo (584 results) S ['21448939'], three-dimensional sequences with isotropic (72 results) D ['19789228', '19668001'], isotropic resolution fast spin-echo (30 results) D ['18408924'], isotropic resolution fast spin-echo (30 results) S ['21448939', '19703886', '19011194', '17449772'], resolution fast spin-echo sequences (263 results) S ['21448939'], isotropic resolution sequences (79 results) S ['21448939'], three-dimensional isotropic resolution (5 results) S ['19703886'], thin continuous slices (24 results) S ['21448939'], two-dimensional fast spin-echo sequences (78 results) D ['19789228', '17717327'], two-dimensional fast spin-echo sequences (78 results) S ['19011194'], identical tissue contrast (3640 results) S ['21448939']
JE	TRUE	0	FALSE	marital behavior (24 results) D ['14992621', '9823523'], Fragile families (94 results) S ['21479113'], marital behavior from infancy (61 results) D ['14992621'], Interrelatedness of marital (10 results) D ['7644602'], EAS temperament structure (8 results) D ['10190344']
JE	TRUE	0	FALSE	Huth Altman Gardner (2 results) D ['2190518'], Gay Humphrey Rogers (1 results) S ['15360816'], Bass Rubin Haynes (1 results) D ['8460861'], informative abstracts (18 results) D ['8460861', '2190518', '3348568'], More informative abstracts (10 results) D ['8460861', '2190518', '3348568'], Initiative's Medical Text (2 results) S ['15360816'], informative abstracts revisited (2 results) D ['2190518'], Wilson Tunis Bass (5 results) D ['8460861'], Aronson Mork Gay Humphrey (1 results) S ['15360816'], Aronson Mork Gay (1 results) S ['15360816'], abstracts revisited (13 results) D ['2190518'], Mulrow Huth Altman Gardner (2 results) D ['2190518'], Hayward Wilson Tunis (5 results) D ['8460861'], informative abstracts of articles (24 results) D ['8460861'], Mork Gay Humphrey Rogers (1 results) S ['15360816'], Mulrow Thacker (8 results) D ['3348568'], Initiative's Medical Text Indexer (1 results) S ['15360816'], Thacker Pugh (2 results) D ['3348568'], Mork Gay Humphrey (1 results) S ['15360816'], More informative abstracts revisited (2 results) D ['2190518']
JE	TRUE	5	TRUE	e-reader's (1 results) S ['21464848'], hospital physicians' work practices (37 results) D ['19717793'], medicine clerkship (438 results) S ['21464848'], e-reader's limitations (1 results) S ['21464848'], e-reader's navigation (1 results) S ['21464848'], additional uses for Kindles (1 results) S ['21464848'], fourth-year (2091 results) S ['21464848'], family medicine clerkship (155 results) S ['21464848'], e-reader's major advantages (1 results) S ['21464848'], clerkship (3922 results) S ['21464848'], preceptors (1192 results) S ['21464848']
JE	TRUE	0	FALSE	Tunks' spinal cord (1 results) D ['12820785'], patient language (24 results) D ['17957419', '16646745'], patient language (24 results) S ['21464851'], step towards consumer health (34 results) D ['15361006'], towards consumer health informatics (14 results) D ['15361006'], pain classification schemes (3 results) D ['12820785'], CINAHL list of subject (8 results) D ['3995203'], Tunks' spinal (1 results) D ['12820785'], injury pain classification schemes (8 results) D ['12820785'], culture-specific models of disease (11 results) D ['17164940'], Luce McLennan (6 results) D ['20207461'], patients' attitudes toward euthanasia (39 results) D ['16317867'], Greer Gupta (15 results) D ['3995203'], Tunks' spinal cord injury (1 results) D ['12820785']

PMC3066582	4	FALSE	14	FALSE		4	13	TRUE	8	TRUE	7	TRUE	6	TRU
PMC3066576	11	FALSE	2	FALSE		10	9	TRUE	5	TRUE	5	TRUE	2	TRU
PMC3066575	19	FALSE	3	FALSE		6	17	TRUE	13	TRUE	12	TRUE	10	TRU
PMC3043123	1	FALSE	6	FALSE		4	4	FALSE	4	TRUE	3	TRUE	2	TRU
PMC2919613	3	FALSE	11	FALSE		6	11	TRUE	5	TRUE	2	TRUE	0	TRU
PMC3071867	1	FALSE	8	FALSE		4	9	TRUE	6	TRUE	6	TRUE	2	TRU

JE	TRUE	3	TRUE	Affective authority of information (9 results) S ['21464850'], Turner Durrance (1 results) D ['18974809'], pieces endometriosis (25 results) S ['21464850'], Eleven blogs (1 results) S ['21464850'], authority claims (251 results) S ['21464850'], claimsmaking (3 results) S ['19940086'], Endometriosis patients (386 results) S ['21464850'], information mediary behavior (1 results) D ['18974809'], clients' talk (69 results) S ['19940086'], affective authority (45 results) S ['21464850'], clients' talk about interventions (4 results) S ['19940086'], consumer health (1467 results) S ['21464850'], Abrahamson Fisher (3 results) D ['18974809'], Affective authority claims (1 results) S ['21464850'], cognitive authority (4 results) S ['21464850'], interpretative repertoires (17 results) D ['10798336'], cognitive authority claims (4 results) S ['21464850'], authors' experiences with endometriosis (1 results) S ['21464850']
JE	TRUE	1	TRUE	non-selective non-steroidal anti-inflammatory drugs (74 results) S ['20346263'], Reuben Rosenthal Steinberg (3 results) D ['15590255'], outpatient anterior cruciate (16 results) D ['17578979', '17578978', '10357331'], Poehlman case (3 results) D ['16522625'], Steinberg Faruqi (1 results) D ['15590255'], retraction notices of papers (4 results) D ['2676047'], Reuben Ekman (4 results) D ['17578979', '17578978', '15741619'], Reuben Ekman Charron (1 results) D ['17578978'], Reuben Pristas (1 results) D ['16428550'], bibliographic databases (3968 results) S ['21464856'], outpatient anterior cruciate ligament (16 results) D ['17578979', '17578978', '10357331'], Rosenthal Steinberg (17 results) D ['15590255'], Reuben Rosenthal Steinberg Faruqi (1 results) D ['15590255']
JE	TRUE	6	TRUE	librarianship (264 results) D ['19626146', '19626143'], MLA Research Section (1 results) S ['21464847'], sciences librarianship (47 results) D ['19626146', '19626143'], Library Association's research (15 results) D ['18379666'], Section Research Agenda Committee (4 results) D ['19626143'], Research Section (1245 results) S ['21464847'], MLA Research (1 results) S ['21464847'], Medical Library Association's research (14 results) D ['18379666'], Nordberg Palmer (40 results) D ['19626146'], Grefsheim Rankin (5 results) D ['18379666'], Library Association's research policy (3 results) D ['18379666'], Palmer Piorun (2 results) D ['19626146'], Medical Library (1802 results) D ['20648260'], Gore Nordberg (1 results) D ['19626146'], Medical Library Association (378 results) D ['20648260', '19626146', '19626143', '19404505'], Section Research Agenda (99 results) D ['19626143'], health sciences librarianship (47 results) D ['19626146', '19626143'], MLA leaders (7 results) D ['19626143'], Piorun Trends (1 results) D ['19626146'], Gore Nordberg Palmer (1 results) D ['19626146'], more than MLA (131 results) D ['20648260', '19626143'], Library Association (406 results) D ['20648260', '19626146', '19626143', '19404505']
JE	TRUE	2	TRUE	FLAIR lesion (8 results) S ['19888926'], same- versus alternate-form memory (1 results) S ['16248908'], FLAIR hyperintense lesions (120 results) S ['19369605'], versus alternate-form memory (1 results) S ['16248908'], versus alternate-form memory tests (1 results) S ['16248908'], FLAIR hyperintense (14 results) D ['10696031'], higher cerebral lesion load (131 results) S ['19888926']
JE	FALSE	0	FALSE	T1rho values for head (4 results) D ['9665548'], T1rho values for head (4 results) S ['15065163'], Hippocampal transverse relaxation (12 results) D ['9314985'], Temporal Lobe Mohammad Haris (1 results) S ['20331502'], Alzheimer's hippocampus T1rho estimation (1 results) S ['19388096'], Alzheimer's hippocampus T1rho (3 results) S ['19388096'], vivo measurement of T1rho (7 results) S ['18777535', '15065163'], measurement of T1rho (20 results) S ['18777535', '15065163'], T1rho estimation (3 results) S ['19388096'], T1rho dispersion (10 results) S ['15065163'], Hippocampal transverse relaxation times (7 results) D ['9314985'], hippocampus T1rho estimation (1 results) S ['19388096'], hippocampus T1rho (4 results) S ['19388096'], T1rho-prepared (2 results) S ['18777535']
JE	TRUE	1	TRUE	EARLY ALLIANCE prevention trial (3 results) S ['11324096', '11146259'], high risk subsample (4 results) S ['21170589'], early-onset conduct intervention outcomes (19 results) D ['15028546'], early-onset conduct intervention outcomes (19 results) S ['11324096'], one-year follow-up assessment (17 results) S ['21170589'], efficacy of PACE (896 results) S ['21170589'], parental outcomes (13 results) S ['21170589'], EARLY ALLIANCE prevention (3 results) S ['11324096', '11146259'], parent outcomes (25 results) S ['21170589']

PMC3072596	8	FALSE	10	FALSE		3	17	TRUE	15	TRUE	15	TRUE	12	TRU
PMC3072595	14	FALSE	13	FALSE		7	25	TRUE	23	TRUE	23	TRUE	19	TRU
PMC3072593	2	FALSE	11	FALSE		4	13	TRUE	10	TRUE	8	TRUE	5	TRU
PMC3072592	12	FALSE	13	FALSE		13	18	TRUE	15	TRUE	13	TRUE	9	TRU

JE	TRUE	6	TRUE	PHYB gene (15 results) S ['21462389'], potato influences photosynthetic performance (1 results) D ['10318685'], phytochrome genes (37 results) D ['17251177'], potato influences photosynthetic (3 results) D ['10318685'], PHYB lines (41 results) S ['21462389'], phytochrome (2473 results) S ['21462389'], homologous phytochrome genes (43 results) D ['17251177'], thaliana PHYB gene (80 results) S ['21462389'], homologous phytochrome (69 results) D ['17251177'], transgenic potato influences photosynthetic (2 results) D ['10318685'], influences photosynthetic performance (11 results) D ['10318685'], PHYB (396 results) S ['21462389'], PHYB transgenic lines (21 results) S ['21462389'], expression of PHYB (216 results) S ['21462389'], transgenic potato influences (12 results) D ['10318685'], photosynthetic rate (1027 results) S ['21462389'], cotton plants (308 results) S ['21462389'], Arabidopsis thaliana PHYB gene (229 results) S ['21462389']
JE	TRUE	8	TRUE	implication for phytoextraction (2 results) D ['20047795'], species under hydroponic (109 results) S ['21462388'], rice varieties grown (89 results) D ['20047795'], multiflorum (555 results) S ['21462388'], different rice varieties grown (34 results) D ['20047795'], different rice (128 results) D ['20047795'], perenne (854 results) S ['21462388'], phytochelatin concentration (3 results) S ['20439132'], Lolium (1568 results) S ['21462388'], cadmium interactions on root (44 results) D ['19427116'], annual ryegrass multiflorum Lam (19 results) D ['19817001'], microbial community of paddy (38 results) D ['19817001'], different phosphorus fertilizer treatments (73 results) D ['19817001'], Lolium mutiforum (1 results) S ['20439132'], mutiforum (1 results) S ['20439132'], hydroponic conditions (70 results) S ['21462388'], morphological traits (1037 results) S ['21462388'], annual ryegrass multiflorum (42 results) D ['19817001'], different rice varieties (20 results) D ['20047795'], root morphological (2637 results) S ['21462388'], root morphological traits (11 results) D ['19783279'], root morphological traits (11 results) S ['21462388'], ryegrass multiflorum (216 results) S ['21462388'], deficient stress among rice (34 results) D ['18500783'], contrast wheat recombinant (75 results) D ['19783279'], ryegrass multiflorum Lam (78 results) D ['19817001'], ryegrass multiflorum Lam (78 results) S ['21462388']
JE	TRUE	3	TRUE	subject underwent extubation (7 results) S ['21462385'], percutaneous lumboperitoneal shunt (6 results) D ['831309', '1194948'], percutaneous lumboperitoneal shunt (6 results) S ['21462385'], shunt tube (174 results) S ['21462385'], underwent extubation (13 results) S ['21462385'], Fifteen obese subjects (4 results) S ['21462385'], symptoms completely cases (12772 results) S ['21462385'], lumboperitoneal (284 results) S ['21462385'], probability of shunt-tube (2 results) S ['21462385'], percutaneous lumboperitoneal (15 results) D ['8455762', '831309', '1194948'], percutaneous lumboperitoneal (15 results) S ['21462385'], abdominal end (25 results) S ['21462385'], Fifteen obese (48 results) S ['21462385']
JE	TRUE	5	TRUE	TGFBI gene mutations (13 results) D ['16683255', '15623763'], novel Thr538Pro mutation (1 results) S ['16809844'], novel Thr538Pro (1 results) S ['16809844'], Chinese families with granular (38 results) S ['18752451'], BIGH3 Arg124 mutations (1 results) D ['12392546'], Thr538Pro (1 results) S ['16809844'], Chinese patients with lattice (18 results) S ['21462384', '16809844'], Thr538Pro mutation (1 results) S ['16809844'], TGFBI gene (98 results) S ['21462384'], Trp mutations of TGFBI (1 results) S ['18752451'], BIGH3 Arg124 (1 results) D ['12392546'], distinct kerato-epithelin mutations (28 results) D ['15623763', '11923233'], distinct kerato-epithelin (37 results) D ['15623763'], homozygous R124H (16 results) D ['9924333'], lattice corneal dystrophy (186 results) S ['21462384'], granular corneal dystrophy (139 results) S ['21462384'], kerato-epithelin mutations (3 results) D ['9780098', '9054935'], TGFBI-linked (5 results) D ['16636649'], kerato-epithelin (17 results) D ['14502125', '11923233', '9780098', '9727509', '9054935'], kerato-epithelin gene (6 results) D ['9727509'], corneal dystrophy (1296 results) S ['21462384'], granular corneal (150 results) S ['21462384'], Arg124 mutations (7 results) D ['12392546'], homozygous R124H mutation (2 results) D ['9924333'], TGFBI (193 results) S ['21462384']

PMC3072590	9	FALSE	12	FALSE		4	21	TRUE	19	TRUE	17	TRUE	16	TRU
PMC3072589	14	FALSE	10	FALSE		8	21	TRUE	18	TRUE	16	TRUE	12	TRU
PMC3072470	5	FALSE	4	FALSE		5	8	FALSE	8	TRUE	7	TRUE	5	TRU
PMC3036775	2	FALSE	3	FALSE		2	5	TRUE	3	TRUE	3	TRUE	0	TRU
PMC3074097	3	FALSE	12	FALSE		7	15	TRUE	14	TRUE	14	TRUE	12	TRU
PMC3071087	1	FALSE	16	FALSE		6	12	TRUE	11	TRUE	9	TRUE	8	TRU

JE	TRUE	14	TRUE	radial artery (5752 results) S ['21462382'], great saphenous (869 results) S ['21462382'], great saphenous vein (761 results) S ['21462382'], right internal thoracic six-year (3 results) D ['17643621'], internal thoracic artery (1991 results) S ['21462382'], internal thoracic six-year (8 results) D ['17643621', '15023868'], vein grafts (4082 results) S ['21462382'], internal thoracic six-year clinical (4 results) D ['17643621'], right internal thoracic (275 results) D ['20106358'], right internal thoracic (275 results) S ['21462382'], great saphenous vein grafts (8 results) S ['21462382'], saphenous-vein coronary surgery (5946 results) S ['21462382'], thoracic six-year clinical (34 results) D ['17643621'], thoracic artery (2145 results) S ['21462382'], thoracic six-year clinical results (14 results) D ['17643621'], saphenous vein beyond results (59 results) D ['20106358'], internal thoracic (2374 results) S ['21462382'], saphenous vein (16210 results) S ['21462382'], right internal thoracic artery (241 results) D ['20106358'], right internal thoracic artery (241 results) S ['21462382'], thoracic six-year (112 results) D ['17643621']
JE	TRUE	8	TRUE	h-hemoperfusion (339 results) D ['19439972'], h-hemoperfusion (339 results) S ['21462381'], cytokine adsorbent column (17 results) D ['17160419'], Review Extracorporeal endotoxin (34 results) D ['12921125'], fiber column on cytokine (28 results) D ['20473253'], adsorber system during hyperdynamic (1 results) D ['19439972'], direct hemoperfusion (214 results) D ['20472994'], extracorporeal endotoxin adsorber system (5 results) D ['19439972'], endotoxin adsorber (11 results) D ['19439972', '18483452'], endotoxin adsorber (11 results) S ['21462381'], novel adsorber (26 results) D ['18483452'], novel adsorber (26 results) S ['21462381'], endotoxin removal strategy (20 results) S ['21462381'], hemoperfusion (3484 results) S ['21462381'], adsorber (284 results) S ['21462381'], endotoxin adsorber system (3 results) D ['19439972'], PVDF-Ser adsorber (1 results) S ['21462381'], Extracorporeal endotoxin removal (7 results) D ['12921125'], endotoxin removal (119 results) S ['21462381'], system during hyperdynamic (328 results) D ['19439972'], extracorporeal endotoxin adsorber (3 results) D ['19439972'], PVDF-Ser (1 results) S ['21462381'], extracorporeal endotoxin (204 results) S ['21462381'], Review Extracorporeal endotoxin removal (17 results) D ['12921125', '9372979']
JE	TRUE	1	TRUE	stem cells confer chemoresistance (10 results) D ['17891174'], sphere clones (26 results) S ['21462380'], human brain tumour (60 results) D ['15549107'], sphere clones of gallbladder (1 results) S ['21462380'], Prospective identification of tumorigenic (11 results) D ['12629218'], clones of gallbladder (21 results) S ['21462380'], tumorigenic breast cancer (24 results) D ['15994920', '12629218'], gallbladder cancer (1555 results) S ['21462380'], tumorigenic breast cancer cells (12 results) D ['15994920', '12629218']
JE	FALSE	0	FALSE	IDIFs (12 results) D ['18344438'], IDIFs (12 results) S ['20521133'], OSEM-MAP (2 results) S ['20521133'], late blood sample (3 results) D ['18344438'], IDIF (18 results) S ['20521133']
JE	TRUE	8	TRUE	Duffy blood (700 results) S ['21468018'], Plasmodium vivax transmission (19 results) S ['20689816'], Duffy blood group (685 results) S ['21468018'], global frequency maps (181 results) S ['21468018'], blood group (40546 results) S ['21468018'], vivax transmission (38 results) S ['20689816'], continuous global frequency maps (5 results) S ['19323591'], Duffy alleles (201 results) S ['21468018'], vivax (6302 results) S ['21468018'], Duffy blood group locus (17 results) D ['11753822', '10762551', '5246559'], urban Tunisian population (39 results) D ['18608113'], common Duffy alleles (39 results) D ['18608113'], common Duffy alleles (39 results) S ['21468018'], Duffy variant (125 results) S ['21468018'], Plasmodium vivax (4163 results) S ['21468018']
JE	TRUE	6	TRUE	apoptotic pathway (3819 results) S ['21472143'], risk genotypes (481 results) S ['21472143'], R01CA109193 (3 results) S ['20453000'], NOS3 rs2070744 (15 results) S ['21472143'], esophageal adenocarcinoma (1625 results) S ['21472143'], Asomaning (39 results) S ['21472143', '20453000', '20385987', '19302219', '18780893'], etiologic patterns of esophageal (10 results) D ['17185192'], etiologic patterns of esophageal (10 results) S ['20385987'], CASP8 rs1035142 (1 results) S ['21472143'], rs720321 (1 results) S ['21472143'], distinct etiologic patterns (64 results) S ['20385987'], BCL2 rs720321 (1 results) S ['21472143'], apoptotic genes CASP8 (446 results) S ['21472143'], rs2070744 BCL2 rs720321 (1 results) S ['21472143'], younger-aged (191 results) S ['21472143'], NOS3 (4898 results) S ['21472143'], rs1035142 (1 results) S ['21472143']

PMC3071082	1	FALSE	19	FALSE		8	13	TRUE	11	TRUE	11	TRUE	9	TRU
PMC3071081	4	FALSE	13	FALSE		7	17	TRUE	16	TRUE	9	TRUE	9	TRU
PMC3071079	2	FALSE	8	FALSE		9	10	TRUE	9	TRUE	8	TRUE	7	TRU
PMC3071078	3	FALSE	13	FALSE		5	16	TRUE	15	TRUE	13	TRUE	13	TRU
PMC3070234	1	FALSE	2	FALSE		2	3	FALSE	3	TRUE	1	TRUE	1	TRU
PMC3070231	0	TRUE	14	FALSE		1	14	FALSE	14	FALSE	14	TRUE	13	TRU

JE	TRUE	5	TRUE	death biomarkers (2 results) S ['19628770'], etoposide (18330 results) S ['21472138'], M65 ELISA cell death (23 results) S ['21472138', '19628770', '19010845', '18813353', '18347133'], mimetic ABT-737 (34 results) S ['19010845'], M65 ELISA cell (24 results) S ['21472138', '19628770', '19010845', '18813353', '18347133'], obatoclax antagonizes MCL-1 (1 results) S ['18040043'], obatoclax (56 results) D ['20200450', '20165849'], obatoclax (56 results) S ['21472138'], obatoclax antagonizes (1 results) S ['18040043'], molecule obatoclax antagonizes (1 results) S ['18040043'], Bcl-2 family (4436 results) S ['21472138'], serological cell death biomarkers (1 results) S ['19628770'], SCLC cell lines (561 results) S ['21472138'], molecule obatoclax antagonizes MCL-1 (1 results) S ['18040043'], small molecule family (2388 results) S ['21472138'], cell death biomarkers (2 results) S ['19628770'], serological cell death (1 results) S ['19628770'], SCLC (4325 results) S ['21472138'], Small molecule obatoclax antagonizes (1 results) S ['18040043'], Clinical evaluation of M30 (24 results) S ['19628770', '19010845', '18813353', '18347133']
JE	TRUE	4	TRUE	nab-paclitaxel treatment (60 results) S ['21472137'], anti-vascular endothelial growth factor (529 results) S ['21472137'], anti-vascular (730 results) S ['21472137'], 231-LuC (8 results) S ['17597103'], nab-paclitaxel (60 results) S ['21472137'], orthotopic 435-LuC (2 results) D ['15377849'], 435-LuC (22 results) D ['15377849', '11351044'], Cremophor-based (8 results) S ['16489089'], 231-LuC tumors (7 results) S ['17597103'], anti-vascular endothelial (544 results) S ['21472137'], concurrent anti-vascular endothelial (7 results) S ['18516298'], anal preliminary evidence (38 results) D ['11745194'], concurrent anti-vascular endothelial growth (7 results) S ['18516298'], concurrent anti-vascular (8 results) S ['18516298'], Luciferase-tagged (41 results) S ['21472137', '18516298'], anti-vascular endothelial growth (533 results) S ['21472137'], 435-LuC tumors (10 results) D ['15377849', '11351044']
JE	TRUE	3	TRUE	kinase inhibitor dasatinib (31 results) S ['19383922'], rHDL (236 results) S ['21472135'], CA110793 (60 results) S ['21160526', '20708159'], CA128797 (37 results) S ['21160526', '20708159'], Antivascular therapy for orthotopic (25 results) S ['17077358', '16000591'], intratumoral androgens during progression (14 results) D ['19866465'], SR-B1 (117 results) D ['19866465'], SR-B1 (117 results) S ['21472135'], HeyA8-MDR (9 results) S ['16361572'], rHDL nanoparticles (4 results) S ['21472135', '19637935']
JE	TRUE	5	TRUE	intertubular vessels (71 results) S ['21472134'], hypoxic regions (325 results) S ['21472134'], bavituximab (10 results) S ['18570887'], duramycin binds (6 results) D ['18632826'], membrane leaflet (305 results) S ['21472134'], PE-positive vessels (2 results) S ['21472134'], PE-positive (32 results) S ['21472134'], peptide duramycin (47 results) D ['18983507', '18632826', '17483159'], peptide duramycin (47 results) S ['21472134', '18570887'], 800CW-linDUR (27 results) S ['21472134'], duramycin (55 results) D ['18983507', '18632826', '17483159'], duramycin (55 results) S ['21472134', '18570887'], 800CW (27 results) S ['21472134'], tumor vasculature (1573 results) S ['21472134'], vascular endothelium (75997 results) S ['21472134'], biotinylated (10876 results) S ['21472134']
JE	TRUE	1	TRUE	relative recall (7 results) D ['19626144'], relative recall (7 results) S ['21464862'], risk factors for bladder (5761 results) S ['21464862']
JE	TRUE	11	TRUE	electronic health records (2242 results) S ['21464859'], Health Information Technology (662 results) S ['21464859'], health records (3603 results) S ['21464859'], EHRs (531 results) S ['21464859'], Coordinator for Health (1436 results) S ['21464859'], National Coordinator (64 results) S ['21464859'], user satisfaction (631 results) S ['21464859'], Kentucky Department of Corrections (12 results) S ['21464859'], online survey with satisfaction (586 results) S ['21464859'], perception of usability (101 results) S ['21464859'], EHR (1334 results) S ['21464859'], KyDOC EHR (1335 results) S ['21464859'], organizational setting (76 results) S ['21464859'], National Coordinator for Health (322 results) S ['21464859']

PMC3069980	10	FALSE	5	FALSE		7	15	TRUE	6	TRUE	5	TRUE	5	TRU
PMC3069979	6	FALSE	5	FALSE		6	10	TRUE	9	TRUE	9	TRUE	8	TRU
PMC3069976	2	FALSE	17	FALSE		3	16	TRUE	14	TRUE	11	TRUE	11	TRU
PMC3069975	5	FALSE	12	FALSE		7	17	FALSE	17	TRUE	14	TRUE	13	TRU
PMC3069974	3	FALSE	17	FALSE		7	20	TRUE	18	TRUE	13	TRUE	10	TRU

JE	TRUE	4	TRUE	human cytomegalovirus (6341 results) S ['21483780'], Human cytomegalovirus smallest capsid (7 results) D ['12552013', '9971801', '8764088'], human cytomegalovirus UL80a (3 results) D ['17079329', '8985337'], amino-conserved (3 results) D ['17079329', '8985337'], HCMV UL48 (10 results) D ['12552013', '8764088'], cytomegalovirus smallest capsid protein (7 results) D ['12552013', '9971801', '8764088'], cytomegalovirus smallest capsid (7 results) D ['12552013', '9971801', '8764088'], cytomegalovirus UL80a proteins (3 results) D ['17079329', '8985337'], virion (16786 results) S ['21483780'], HCMV (3975 results) S ['21483780'], human cytomegalovirus gene (57 results) S ['15883374'], cytomegalovirus UL80a (3 results) D ['17079329', '8985337'], capsid (22378 results) S ['21483780'], human cytomegalovirus UL80a proteins (3 results) D ['17079329', '8985337'], UL80a proteins (3 results) D ['17079329', '8985337']
JE	TRUE	6	TRUE	turtle ventricular myocytes (1 results) D ['16887918'], trisphosphate-operated (4 results) D ['18818435'], cell-derived (11351 results) S ['21483779'], developmental regulation of transients (49 results) D ['16787575'], sarcoplasmic (19523 results) S ['21483779'], turtle ventricular (175 results) D ['16887918'], reticulum for calcium (21673 results) S ['21483779'], sarcoplasmic reticulum for calcium (14837 results) S ['21483779'], sarcoplasmic reticulum (18427 results) S ['21483779'], Local control of excitation-contraction (48 results) D ['19404384'], Rapid photochemical inactivation (18 results) D ['6308474']
JE	TRUE	9	TRUE	trimeric (5731 results) S ['21483777'], trimeric spike proteins (71 results) D ['19372381'], trimeric spike proteins (71 results) S ['21483777'], RSV (6549 results) S ['21483777'], trivalent VHH (1 results) S ['21483777'], H5N1 (3994 results) S ['21483777'], biparatopic VHH (2 results) S ['21483777'], Rabies (11413 results) S ['21483777'], monovalent VHH (6 results) D ['16736523'], bivalent (4851 results) S ['21483777'], biparatopic (6 results) S ['21483777'], VHH constructs (9 results) S ['21483777'], hemagglutinin (10561 results) S ['21483777'], biparatopic constructs (1 results) S ['21483777'], VHH (150 results) S ['21483777'], VHH against Influenza (2 results) S ['21483777'], Influenza H5N1 (411 results) S ['21483777'], biparatopic VHH construct (1 results) S ['21483777'], trivalent (4567 results) S ['21483777']
JE	TRUE	10	TRUE	concanavalin (27867 results) S ['21483776'], vanilloid (2544 results) S ['21483776'], suppressor cells (5224 results) S ['21483776'], cannabinoid (8373 results) S ['21483776'], experimental autoimmune hepatitis (36 results) S ['21483776', '18388242'], CD44-deficient mice exhibit (9 results) S ['11342603'], autoimmune hepatitis (3392 results) S ['21483776'], new immunoregulatory pathway (61 results) D ['18674538'], myeloid-derived (432 results) S ['21483776'], Oromucosal for neuropathic (10 results) D ['18035205'], hepatitis after concanavalin (177 results) S ['21483776'], cannabidiol (890 results) S ['21483776'], TRPV1 (2240 results) S ['21483776'], Vanilloid TRPV1 receptor mediates (8 results) D ['15313881'], activation of TRPV1 (877 results) S ['21483776'], TRPV1 receptor mediates (14 results) D ['15313881'], IFN-gamma treatment impairs (94 results) D ['19637228']
JE	TRUE	7	TRUE	assortment modifier (10 results) D ['19154394'], assortment modifier (10 results) S ['21483775', '19780814'], fixation of modifiers (21 results) S ['19780814', '18177673'], evolution of assortment (176 results) S ['21483775'], small degree of assortment (11 results) S ['21483775', '19780814'], intermediate level of assortment (4 results) S ['19780814'], degree of dominance (1620 results) S ['21483775'], levels of assortment (131 results) S ['19780814'], intermediate dominance (10 results) S ['21483775'], higher levels of assortment (17 results) S ['19780814'], ecological locus (329 results) S ['21483775'], high levels of assortment (37 results) S ['17236413'], assortative mating (830 results) S ['21483775'], assortative (1060 results) S ['21483775'], degree of assortment (69 results) S ['21483775', '19780814'], strong assortment (6 results) D ['19154394'], strong assortment (6 results) S ['17236413', '16599933'], strength of assortment (21 results) D ['18660541'], strength of assortment (21 results) S ['17236413', '16599933'], modifier locus (159 results) S ['21483775', '19780814']

PMC3069973	10	FALSE	16	FALSE		8	21	TRUE	12	TRUE	9	TRUE	8	TRU
PMC3069972	2	FALSE	18	FALSE		5	19	TRUE	11	TRUE	11	TRUE	11	TRU
PMC3069971	1	FALSE	11	FALSE		5	12	TRUE	11	TRUE	8	TRUE	5	TRU
PMC3069970	10	FALSE	18	FALSE		10	28	TRUE	27	TRUE	26	TRUE	25	TRU

JE	TRUE	4	TRUE	neonicotinoids of insect-specific (3 results) S ['16868180'], asperparaline (6 results) D ['12589698'], asperparaline (6 results) S ['21483774', '10705455'], nicotinic acetylcholine receptors (3751 results) S ['21483774'], thoracic ganglion neurons (6 results) D ['15014137', '14637375'], acetylcholine receptors (7485 results) S ['21483774'], japonicus JV-23 (2 results) S ['21483774', '10705455'], cockroach thoracic ganglion neurons (3 results) D ['15014137', '14637375'], paralytic alkaloids (1 results) S ['10705455'], cockroach thoracic ganglion (4 results) D ['15014137', '14637375'], JV-23 (2 results) S ['21483774', '10705455'], asperparalines (3 results) D ['12589698'], asperparalines (3 results) S ['21483774', '10705455'], nAChRs (2486 results) S ['21483774'], insect-specific basic residues (2 results) S ['16868180'], Fipronil modulation (3 results) D ['14637375'], selectivity of neonicotinoids (23 results) S ['16868180'], mori larval brain (46 results) S ['21483774'], nicotinic acetylcholine (8360 results) S ['21483774'], cockroach nicotinic acetylcholine (51 results) D ['15518655'], New paralytic alkaloids (1 results) S ['10705455'], cockroach nicotinic acetylcholine receptors (61 results) D ['15518655'], Aspergillus japonicus JV-23 (2 results) S ['21483774', '10705455'], mori nAChRs (6 results) D ['17868469'], mori nAChRs (6 results) S ['21483774'], cockroach neurons (19 results) D ['15518655', '15014137']
JE	TRUE	7	TRUE	serum miR-126 (3 results) S ['21483773'], early detection of MPM (52 results) S ['21483773'], miR-126 (105 results) S ['21483773'], non-cancerous tissue sample pairs (3 results) S ['21483773'], MPM (1303 results) S ['21483773'], association with SMRPs (2 results) S ['21483773'], malignant pleural mesothelioma (1644 results) S ['21483773'], detection of MPM (51 results) S ['21483773'], marker of MPM (122 results) S ['21483773'], malignant pleural (3431 results) S ['21483773'], shipyard pipe coverers (2 results) D ['5113726'], combination with SMRPs (4 results) S ['21483773', '18199721'], surveillance procedure for cohorts (5468 results) S ['21483773'], fresh-frozen biopsies of MPM (1 results) S ['21483773'], specific marker of MPM (30 results) S ['21483773'], adjacent non-cancerous tissue sample (3 results) S ['21483773'], SMRPs (6 results) D ['19945835', '16456138'], SMRPs (6 results) S ['21483773', '18199721'], biopsies of MPM (77 results) S ['21483773'], MPM tissue (219 results) S ['21483773']
JE	TRUE	3	TRUE	pesticide exposure (1208 results) S ['21483772'], ecosystem risk organophosphate (7 results) S ['19244487'], ecosystem risk organophosphate pesticides (7 results) S ['19244487'], surface water mobility index (13 results) D ['11833798'], pesticide exposure assessment (31 results) S ['20036451'], pesticide environmental fate data (247 results) S ['21483772'], ecosystem risk (14 results) S ['19244487'], use-exposure (54 results) S ['21483772'], use-exposure relationship (6 results) S ['21483772'], pesticide environmental fate (3 results) S ['18384237'], crop scenarios (155 results) S ['21483772'], environmental fate data (12 results) S ['18384237']
JE	TRUE	16	TRUE	white-tailed (1316 results) S ['21483771'], prion protein (5740 results) S ['21483771'], prion protein PrP TSE (435 results) S ['21483771'], elk elaphus (240 results) D ['20436863'], pathological prion protein (99 results) S ['21483771'], cervids (299 results) S ['21483771'], humans with transmissible (4184 results) S ['21483771'], Scrapie infectivity correlates (10 results) D ['9094691'], normal hamster brain homogenate (20 results) D ['17643109'], transmission of chronic (11220 results) S ['21483771'], prion (10031 results) S ['21483771'], presence of PrP (1080 results) S ['21483771'], CWD-infected (32 results) D ['20610667', '19788803', '17223321'], CWD-infected (32 results) S ['21483771'], skeletal muscles (10593 results) S ['21483771'], clinical signs of CWD (83 results) D ['19788803', '18487485'], clinical signs of CWD (83 results) S ['21483771'], CWD (474 results) S ['21483771'], WTD (91 results) S ['21483771'], protein PrP TSE (440 results) S ['21483771'], biological prion infectivity (392 results) D ['20466881'], biological prion infectivity (392 results) S ['21483771'], stage of CWD (28 results) D ['20436863'], guanidine denaturation studies (3 results) D ['9094691'], cardiac muscle of elk (27 results) D ['20436863', '17030881'], muscle of elk (154 results) D ['20436863'], muscle of elk (154 results) S ['21483771'], PrP TSE (28 results) S ['21483771']

PMC3069969	19	FALSE	4	FALSE		8	16	TRUE	15	TRUE	14	TRUE	9	TRU
PMC3069967	8	FALSE	4	FALSE		7	12	TRUE	11	TRUE	7	TRUE	6	TRU
PMC3069966	1	FALSE	14	FALSE		6	14	TRUE	10	TRUE	9	TRUE	7	TRU
PMC3069965	6	FALSE	16	FALSE		7	20	TRUE	19	TRUE	18	TRUE	16	TRU
PMC3069964	6	FALSE	4	FALSE		12	9	TRUE	8	TRUE	7	TRUE	5	TRU

JE	TRUE	3	TRUE	mesodermal (5156 results) S ['21483770'], Review Sprouty (31 results) D ['19570949', '18219583', '16337795'], endothelial antigen ESAM (19 results) D ['19096010'], hematopoietic progenitors throughout life (58 results) D ['19096010'], Intermolecular interactions of Sprouty (1 results) D ['19570949'], endothelial antigen ESAM marks (1 results) D ['19096010'], antigen ESAM marks primitive (1 results) D ['19096010'], marks primitive hematopoietic progenitors (9 results) D ['19096010'], mesodermal precursors (60 results) D ['18339678', '16926294'], antigen ESAM (19 results) D ['19096010'], erythropoiesis from mesodermal (22 results) D ['16926294'], Tie2 (847 results) S ['21483770'], ESAM marks primitive (1 results) D ['19096010'], ESAM marks primitive hematopoietic (1 results) D ['19096010'], stepwise specification of embryonic (19 results) D ['18339678'], Spry1 (93 results) S ['21483770'], primitive hematopoietic (949 results) S ['21483770'], ESAM marks (1 results) D ['19096010'], interactions of Sprouty (23 results) D ['19570949'], Primitive erythropoiesis from mesodermal (12 results) D ['16926294'], marks primitive hematopoietic (19 results) D ['19096010'], Suppression of Sproutys (2 results) D ['19424491'], antigen ESAM marks (1 results) D ['19096010']
JE	TRUE	5	TRUE	molecular correlates for polarization (70 results) D ['19193240'], receptor ligand stimulates Th2 (17 results) D ['15067049'], autocrine PGE2 (322 results) D ['19564345'], Th2 responses (1485 results) S ['21483768'], lectin pattern recognition receptors (6 results) D ['16849491'], Pam CSK4 (8 results) S ['21483768'], individual species of mannosides (4 results) D ['16849491'], TLR2 (4098 results) S ['21483768'], C-type lectin pattern recognition (7 results) D ['16849491'], C-type lectin pattern (7 results) D ['16849491'], vaccae (364 results) D ['20118767', '19438539'], vaccae (364 results) S ['21483768']
JE	TRUE	2	TRUE	human intraparietal sulcus (12 results) S ['16432904'], parietal cytoarchitectonic (127 results) S ['18651173'], Receptor architecture (28 results) S ['19034899'], Cytoarchitectonic identification (39 results) S ['16432904'], parietal cytoarchitectonic parcellation (10 results) S ['16949304', '16432904'], inferior parietal cytoarchitectonic (29 results) S ['18651173', '16949304'], entorhinal intersubject (3 results) S ['16208455'], ventral bank (83 results) D ['14729132'], ventral bank (83 results) S ['16432904'], four-region neurobiological (2 results) S ['19034899'], inferior parietal cytoarchitectonic parcellation (4 results) S ['16949304'], anterior ventral bank (103 results) S ['16432904'], four-region neurobiological model (1 results) S ['19034899'], entorhinal intersubject variability (2 results) S ['16208455'], human inferior parietal cytoarchitectonic (14 results) S ['18651173', '16949304']
JE	TRUE	14	TRUE	ethylmalonic (157 results) D ['19757035'], ethylmalonic (157 results) S ['20443061'], Antioxidant potential risk (18376 results) S ['21483766'], short-chain acyl-CoA dehydrogenase deficiency (45 results) D ['18714522'], short-chain acyl-CoA dehydrogenase deficiency (45 results) S ['21483766'], ACADS gene variation spectrum (1 results) S ['18523805'], acyl-CoA dehydrogenase deficiency (491 results) S ['21483766'], acyl-CoA (8074 results) S ['21483766'], ACADS gene variation (4 results) S ['18523805'], risk for neurotoxicity (3384 results) S ['21483766'], short-chain acyl-CoA (240 results) D ['19757035'], short-chain acyl-CoA (240 results) S ['21483766'], potential risk for neurotoxicity (3384 results) S ['21483766'], SCADD fibroblasts (1 results) S ['21483766'], short-chain acyl-CoA dehydrogenase (160 results) D ['19757035'], short-chain acyl-CoA dehydrogenase (160 results) S ['21483766'], Bezafibrate increases acyl-CoA dehydrogenase (6 results) D ['16115821'], acyl-CoA dehydrogenase (1867 results) S ['21483766'], short-chain (8434 results) S ['21483766'], SCADD (15 results) D ['18714522', '16926354'], SCADD (15 results) S ['21483766'], dehydrogenase deficiency (5732 results) S ['21483766']
JE	TRUE	2	TRUE	Xenopus evidence for cooperation (7 results) S ['16214870'], load dependence of kinesin's (3 results) D ['9238012'], bidirectional motility of melanophore (14 results) D ['12551954', '9852150', '9108044', '1348251'], dynein molecule characterization (11 results) D ['8580344'], microtubules (31026 results) S ['21483765'], diffusion of CFTR (47 results) D ['17483157'], endocytic effects of dynamitin (2 results) D ['10588646', '9334349'], pigment granules along microtubules (28 results) D ['9108044'], pigment granules along microtubules (28 results) S ['19751659', '16214870'], Organelle transport along microtubules (540 results) S ['21483765']

PMC3069963	6	FALSE	14	FALSE		9	20	FALSE	20	TRUE	19	TRUE	14	TRU
PMC3069654	0	TRUE	13	FALSE		5	13	TRUE	12	TRUE	11	TRUE	10	TRU
PMC3069653	5	FALSE	6	FALSE		6	11	TRUE	10	TRUE	10	TRUE	7	TRU
PMC3069651	0	TRUE	14	FALSE		1	14	FALSE	14	FALSE	14	TRUE	13	TRU
PMC3069650	0	TRUE	1	FALSE		1	1	FALSE	1	FALSE	1	FALSE	1	FAL
PMC3069649	0	TRUE	1	FALSE		1	1	FALSE	1	FALSE	1	FALSE	1	FAL
PMC3068990	14	FALSE	9	FALSE		4	23	FALSE	23	FALSE	23	TRUE	22	TRU

JE	TRUE	8	TRUE	human T-cell (10315 results) S ['21483764'], leukemia virus (18931 results) S ['21483764'], Synergy between anti-CD4 (13 results) D ['7908442'], HTLV-1 (3033 results) S ['21483764'], adult T-cell alternate molecular (14 results) D ['1757474'], leukemia virus type (2124 results) S ['21483764'], arthropathy (5849 results) S ['21483764'], HTLV-1 promotes oncogenesis (6 results) S ['17479090'], cell leukemia virus (2693 results) S ['21483764'], arthropathic Tax transgenic (21 results) D ['10072546', '9933086', '9117461', '7636219'], arthropathic Tax transgenic (21 results) S ['21483764'], inflammatory arthropathy (171 results) S ['21483764'], Tax transgenic (33 results) D ['16550188'], Tax transgenic (33 results) S ['21483764'], T-cell leukemia (8218 results) S ['21483764'], arthropathic Tax (61 results) S ['21483764'], arthropathic Tax transgenic mice (20 results) D ['10072546', '9933086', '9117461', '7636219'], arthropathic Tax transgenic mice (20 results) S ['21483764'], Tax transgenic mice (26 results) D ['16550188', '9117461'], Tax transgenic mice (26 results) S ['21483764']
JE	TRUE	7	TRUE	DCE-MRI (740 results) S ['21461174'], carcinoma xenograft tumors (9 results) S ['20885892', '19724685'], renal cell carcinoma xenograft (17 results) S ['20885892', '19724685'], isoflavones mixture (187 results) S ['20309614'], kidney tumors (998 results) S ['21461174'], cell carcinoma xenograft (57 results) S ['20885892', '19724685'], vascular changes (4561 results) S ['21461174'], isoflavones (7037 results) S ['21461174'], soy isoflavones (705 results) S ['21461174'], cell carcinoma xenograft tumors (3 results) S ['20885892', '19724685'], regularization of tumor (110 results) S ['21461174', '20885892'], soy isoflavones mixture (66 results) S ['20309614', '19101986'], sunitinib-induced (55 results) S ['21461174', '20885892']
JE	TRUE	2	TRUE	SOCS gene (29 results) D ['18754879'], anti-tumor monoclonal antibody recognizes (43 results) S ['12470474'], silencing of SOCS-1 (42 results) D ['18754879'], silencing of SOCS-1 (42 results) S ['21461173'], invasive murine melanoma cells (2 results) S ['12470474'], B16F10-Nex2 (14 results) S ['21461173'], SOCS gene expression (16 results) D ['18754879'], SOCS-1 (454 results) S ['21461173'], SOCS protein expression influences (11 results) D ['20398276'], SOCS-1 protein (24 results) D ['15456882', '15373779'], SOCS (1670 results) S ['21461173']
JE	TRUE	13	TRUE	MNCs of healthy (126 results) S ['21461171'], phosphorylation of Src (10098 results) S ['21461171'], Src family proteins (18 results) S ['21461171'], MNCs from healthy (126 results) S ['21461171'], MNCs (1213 results) S ['21461171'], family proteins (4496 results) S ['21461171'], pancreatic tumor (2042 results) S ['21461171'], candidate markers for pancreatic (203 results) S ['21461171'], Src family (7475 results) S ['21461171'], healthy mice (741 results) S ['21461171'], protein spots of interest (297 results) S ['21461171'], MNCs from mice (225 results) S ['21461171'], protein spots (3178 results) S ['21461171'], markers of pancreatic (7859 results) S ['21461171']
.SE	TRUE	1	TRUE	tumor-targeted (473 results) S ['21461170']
.SE	TRUE	1	TRUE	TKIs (765 results) S ['21461169']
JE	TRUE	14	TRUE	efficient piggyBac mutagenesis system (14 results) D ['21418605', '16260745'], transposase (2150 results) D ['21418605'], blood-stage (1313 results) D ['21418605'], piggyBac (229 results) D ['21418605'], piggyBac (229 results) S ['21453557'], Plasmodium genes (30 results) D ['21418605'], genetic tools for Plasmodium (115 results) D ['19422698'], genetic screens (907 results) D ['21418605'], genetic screens (907 results) S ['21453557'], berghei (4606 results) D ['21418605'], berghei (4606 results) S ['21453557'], piggyBac mutagenesis (73 results) D ['21418605'], piggyBac mutagenesis (73 results) S ['21453557'], genome-wide mutagenesis (44 results) S ['21453557'], mutagenesis system for genome-wide (166 results) D ['21418605'], mutagenesis system for genome-wide (166 results) S ['21453557'], Fonager (38 results) D ['21418605'], piggyBac mutagenesis system (33 results) D ['21418605'], piggyBac mutagenesis system (33 results) S ['21453557'], mutagenesis system (168 results) S ['21453557'], Plasmodium parasites (288 results) S ['21453557'], piggyBac insertion (100 results) D ['21418605'], human malaria (1908 results) D ['21418605']

PMC3073913	6	FALSE	22	FALSE		14	26	TRUE	24	TRUE	20	TRUE	17	TRU
PMC3078843	3	FALSE	21	FALSE		4	23	TRUE	22	TRUE	21	TRUE	16	TRU
PMC3078852	1	FALSE	7	FALSE		3	8	FALSE	8	FALSE	8	TRUE	7	TRU
PMC3078834	2	FALSE	14	FALSE		6	16	TRUE	11	TRUE	11	TRUE	10	TRU

JE	TRUE	11	TRUE	morphological patterns (539 results) S ['21453486'], new isolates (630 results) S ['21453486'], major groups (2856 results) S ['21453486'], polar granules (121 results) S ['19397201'], dictyostelia morphological evolution (11 results) S ['21453486', '20524591', '19397201'], austroandinum (1 results) S ['20943559'], major group of Dictyostelia (37 results) D ['19215294'], major group of Dictyostelia (37 results) S ['21453486', '20303322'], dictyostelid tree (6 results) S ['21453486', '20303322', '17663129'], Global Biodiversity Survey (94 results) S ['21453486'], Dictyostelia (12 results) D ['17563079'], Dictyostelia (12 results) S ['21453486', '20303322', '17068267'], traditional genera (6 results) S ['21453486'], dictyostelid (28 results) D ['18592909'], dictyostelid (28 results) S ['21453486', '20943559', '20524591', '20303322', '19397201', '17663129', '17068267', '15851658'], social amoebas (11 results) D ['17563079'], social amoebas (11 results) S ['21453486', '20303322', '17068267'], phylogeny of Dictyostelia (192 results) S ['21453486', '20943559', '20524591', '20303322'], discoideum (4765 results) S ['21453486'], species of Dictyostelia (473 results) D ['21148389'], species of Dictyostelia (473 results) S ['21453486', '20943559', '20524591'], dictyostelids (23 results) D ['18592909'], dictyostelids (23 results) S ['20943559', '20303322', '19397201', '17663129', '17056743', '16396357'], dictyostelia SSU rDNA sequences (5 results) S ['20943559', '20524591', '20303322', '19397201'], valdivianum (1 results) S ['20943559'], amoebae (2747 results) S ['21453486'], deep morphological patterns (168 results) S ['21453486'], deep taxon (148 results) S ['21453486']
JE	TRUE	8	TRUE	different from individual-level (966 results) S ['21453534'], agreement between aggregate-level (9 results) S ['21453534'], correlations between individual-level (172 results) S ['21453534'], university degree (215 results) S ['21453534'], individual-level measures (36 results) S ['21453534'], aggregate-level (336 results) S ['21453534'], biserial correlation (70 results) S ['21453534'], individual-level measures of income (120 results) S ['21453534'], worst estimations of income (1 results) S ['21453534'], aggregate-level measures (3 results) S ['21453534'], income category (62 results) D ['19620271'], income category (62 results) S ['21453534'], lowest income category (16 results) D ['19620271'], lowest income category (16 results) S ['21453534'], individual-level income (15 results) D ['16224305', '11454499'], individual-level income (15 results) S ['21453534'], aggregate-level measures of income (14 results) S ['21453534'], Canadian census (137 results) S ['21453534'], aggregate-level education (62 results) S ['21453534'], coefficients between individual-level (123 results) S ['21453534'], correlation coefficients between individual-level (66 results) S ['21453534'], Point biserial correlation (54 results) S ['21453534'], agreement between individual-level (143 results) S ['21453534'], Aggregate-level income (37 results) S ['21453534']
JE	TRUE	7	TRUE	relationship of 5HTT (14 results) D ['17987668'], older parents (147 results) S ['21453505'], CpGs (1025 results) S ['21453505'], significant CpGs (123 results) S ['21308978'], parental age (1247 results) S ['21453505'], newborn DNA methylation (395 results) S ['21453505', '21308978'], methylation levels (777 results) S ['21453505'], paternal age (1202 results) S ['21453505']
JE	TRUE	7	TRUE	Selective estrogen receptor discrimination (19 results) D ['14973112'], postgenome cohort study (3 results) S ['18271962'], systemic gene set (446 results) S ['21453500'], NOWAC postgenome (5 results) S ['21453500', '20300640', '18271962'], Cancer postgenome cohort (2 results) S ['18271962'], Receptor Discrimination of Agonistic (29 results) D ['14973112'], novel oestrogen gene (2369 results) S ['21453500'], Gene-wise analysis (2 results) S ['21453500'], NOWAC (27 results) S ['21453500', '20300640', '18271962', '17644530', '15305384'], hormone concentrations (4647 results) S ['21453500'], gene sets (1028 results) S ['21453500'], Seven gene sets (749 results) S ['21453500'], SHBG (3448 results) S ['21453500'], postgenome cohort (2 results) S ['18271962'], oestrogen signature (256 results) S ['21453500'], postgenome (100 results) S ['21453500', '20300640']

PMC3076278	4	FALSE	15	FALSE		5	17	TRUE	16	TRUE	16	TRUE	15	TRU
PMC3078854	7	FALSE	9	FALSE		9	16	TRUE	13	TRUE	8	TRUE	5	TRU
PMC3076296	3	FALSE	6	FALSE		3	9	FALSE	9	FALSE	9	TRUE	6	TRU
PMC3078846	0	TRUE	14	FALSE		2	13	TRUE	12	TRUE	12	TRUE	11	TRU
PMC3078091	1	FALSE	11	FALSE		2	12	FALSE	12	TRUE	11	TRUE	11	TRU
PMC3078883	2	FALSE	13	FALSE		8	13	FALSE	13	TRUE	12	TRUE	9	TRU

JE	TRUE	13	TRUE	professional-cluster trials (1 results) S ['21453543'], patient recruitment strategies (4 results) S ['21453543'], selection bias (6801 results) S ['21453543'], evaluate patient recruitment strategies (1225 results) D ['21085696'], evaluate patient recruitment strategies (1225 results) S ['21453543'], professional-cluster (1 results) S ['21453543'], primary care settings (2020 results) S ['21453543'], practice guideline for acute (5534 results) S ['21453543'], practice staff (254 results) S ['21453543'], quantitative items (13 results) S ['21453543'], recruitment barrier surveys (61 results) D ['19254374'], recruitment strategy over another (268 results) D ['21085696'], patient recruitment (487 results) S ['21453543'], poor patient recruitment (3575 results) S ['21453543'], control arm of dissemination (31 results) S ['18294375'], guideline for acute (7414 results) S ['21453543'], recruitment strategies (623 results) D ['21144048'], recruitment strategies (623 results) S ['21453543'], evaluate patient recruitment (17362 results) S ['21453543']
JE	TRUE	4	TRUE	greA deletion (6 results) D ['17766423', '16553885', '7538676'], greA deletion (6 results) S ['21453489'], DksA (91 results) D ['20478253'], factor GreA (15 results) D ['17766423', '15294156'], factor GreA (15 results) S ['21453489'], replication forks (1709 results) S ['21453489'], GreA (113 results) D ['20478253', '18333883'], GreA (113 results) S ['21453489'], greA mutant (10 results) D ['17766423', '11118220', '7538676'], greA mutant (10 results) S ['21453489'], replication-independent (211 results) S ['21453489'], D41A (5 results) S ['21453489'], elongation factor GreA (8 results) D ['17766423'], elongation factor GreA (8 results) S ['21453489'], deletion of dksA (14 results) D ['2180916'], D41N (2 results) S ['21453489']
JE	TRUE	6	TRUE	Umbilical Artery (4741 results) S ['21453488'], Artery Blood Velocity Waveforms (18 results) D ['9685561'], cotinine (3696 results) S ['21453488'], urinary cotinine (436 results) S ['21453488'], Umbilical Artery Blood Velocity (19 results) D ['9685561'], resistance indices (500 results) S ['21453488'], fetal cerebral artery blood (1259 results) S ['21453488'], fetal cerebral artery (14 results) D ['15363837'], pregnant smokers (266 results) S ['21453488']
JE	TRUE	7	TRUE	rs25531 polymorphisms (4 results) S ['21453464'], transporter promoter polymorphism (78 results) S ['21453464'], 5-HTTLPR segment (1 results) S ['20450949'], transporter promoter (232 results) S ['21453464'], serotonin transporter promoter (205 results) S ['21453464'], serotonin transporter gene (944 results) S ['21453464'], whole sample of volunteers (309 results) S ['21453464'], serotonin transporter promoter polymorphism (77 results) S ['21453464'], rs25531 (64 results) S ['21453464'], 5-HTTLPR (922 results) S ['21453464'], modulation of 5-HTTLPR (29 results) S ['20450949'], anxiety-related (2119 results) S ['21453464'], influence of rs25531 (12 results) S ['21453464', '20450949'], association studies on personality (5585 results) S ['21453464']
JE	TRUE	6	TRUE	cohabitation status (61 results) S ['21453478'], attempts decreases with age (94 results) S ['21453478'], Spanish general population (46 results) D ['17980118'], equation logistic (1526 results) S ['21453478'], suicide attempt age (3124 results) S ['21453478'], suicide attempt decreases (39 results) S ['21453478'], equation logistic regression (23 results) S ['21453478'], equation logistic regression models (6 results) S ['21453478'], previous suicide attempts (225 results) S ['21453478'], previous suicide (325 results) S ['21453478'], psychiatric diagnosis (3058 results) S ['21453478'], Affective Disorders (9083 results) S ['21453478']
JE	TRUE	7	TRUE	minutes group (159 results) S ['21453540'], step goal (13 results) D ['18029834', '14768840', '14715035'], step goal (13 results) S ['21453540'], day message (19 results) S ['21453540'], day message on physical (67 results) S ['21453540'], Trial ACTRN12609000176268 (1 results) S ['19703317'], pedometer (742 results) S ['21453540'], physical activity levels (1618 results) S ['21453540'], messages on physical (1028 results) S ['21453540'], sedentary overweight (52 results) S ['21453540', '19703317'], steps group versus (324 results) S ['21453540'], ACTRN12609000176268 (1 results) S ['19703317'], Yamax Digi-Walker (11 results) D ['16825270', '14767259', '14523320'], average of steps (2985 results) S ['21453540'], steps goal (2577 results) S ['21453540']

PMC3078882	10	FALSE	18	FALSE		11	26	TRUE	16	TRUE	13	TRUE	11	TRU
PMC3078098	7	FALSE	5	FALSE		4	11	TRUE	6	TRUE	6	TRUE	6	TRU
PMC3076275	0	TRUE	11	FALSE		2	10	TRUE	7	TRUE	7	TRUE	7	TRU
PMC3078871	5	FALSE	16	FALSE		4	16	TRUE	15	TRUE	11	TRUE	10	TRU

JE	TRUE	7	TRUE	conjunctivochalasis patients (5 results) D ['20019361', '16227820', '14989959'], ophthalmoscope for examination (447 results) S ['21453468'], Tseng's system (1 results) S ['21453468'], epidemiologic study (4814 results) S ['21453468'], prevalence rates of conjunctivochalasis (1 results) S ['21453468'], conjunctivochalasis (59 results) D ['20137283', '20019361', '19403112', '18775527'], conjunctivochalasis (59 results) S ['21453468'], Meller (1180 results) S ['21453468'], grade of conjunctivochalasis (13 results) D ['20019361', '19403112', '18775527', '17916317', '17413955'], grade of conjunctivochalasis (13 results) S ['21453468'], Caoyangxincun community (2 results) D ['20137283'], Caoyangxincun community (2 results) S ['21453468'], eye disease (3511 results) S ['21453468'], symptomatic conjunctivochalasis (4 results) D ['18724161', '18580254'], study of conjunctivochalasis (26 results) D ['20137283', '19403112', '18775527', '17413955', '16227820'], study of conjunctivochalasis (26 results) S ['21453468'], significant conjunctivochalasis (13 results) D ['20019361', '17916317', '17413955'], community of Shanghai (561 results) S ['21453468'], severity of conjunctivochalasis (8 results) D ['19403112', '18775527'], severity of conjunctivochalasis (8 results) S ['21453468'], large scale epidemiologic (147 results) S ['21453468'], Caoyangxincun community of Shanghai (2 results) D ['20137283'], Caoyangxincun community of Shanghai (2 results) S ['21453468'], CaoYangXinCun (2 results) D ['20137283'], CaoYangXinCun (2 results) S ['21453468'], scale epidemiologic data (18716 results) S ['21453468'], rates of conjunctivochalasis (3 results) S ['21453468'], Tseng's (2 results) S ['21453468']
JE	TRUE	5	TRUE	preschool-age Mexican-American (3 results) D ['12146552'], Jamaican coexistence (4 results) D ['12020379'], Jamaican coexistence of under- (1 results) D ['12020379'], preschool-age Mexican-American children (3 results) D ['12146552'], Nutritional status of Jamaican (39 results) D ['12020379'], food insecurity (660 results) S ['21453491'], nutritional outcomes of preschool-age (4 results) D ['12146552'], childhood overweight (517 results) S ['21453491'], Household food (462 results) S ['21453491'], Child-specific food insecurity (2 results) D ['18203906'], food-insecure (199 results) S ['21453491'], Household food insecurity (125 results) S ['21453491']
JE	TRUE	5	TRUE	several research gaps remain (758 results) S ['21453509'], qualitative evaluation (1687 results) S ['21453509'], physical activity consultation (5 results) S ['18655723'], discussions with WWW (67 results) S ['21453509'], WWW study (4 results) S ['21453509'], assessment on motivation (9678 results) S ['21453509'], West' on physical (1 results) S ['18655723'], pedometer (742 results) S ['21453509'], wider community (353 results) S ['21453509'], pedometer-based (60 results) S ['21453509'], activity consultation (5 results) S ['18655723']
JE	TRUE	7	TRUE	interval for E2F1 (9 results) D ['17535433'], expression of KIAA0191 (1 results) S ['21453498'], relationship between E2F1 (141 results) S ['21453498'], microRNA biogenesis through uridylation (8 results) D ['19703396'], comprise many genes (560 results) S ['21453498'], levels of KIAA0191 (1 results) S ['21453498'], relationship of KIAA0191 (1 results) S ['21453498'], pathology laboratories (783 results) S ['21453498'], molecular signatures (775 results) S ['21453498'], KIAA0191 expression (1 results) S ['21453498'], available prognostic signatures (62 results) S ['21453498'], 2-gene signature (2 results) S ['21453498'], confidence interval for E2F1 (8 results) D ['17535433'], breast cancer patient survival (14 results) S ['21453498'], E2F1 (2010 results) S ['21453498'], KIAA0191 (1 results) S ['21453498'], proliferation gene (37 results) D ['18635567'], E2F1 transcript levels (27 results) D ['17535433'], hospital pathology laboratories (6 results) S ['21453498'], cancer patient survival (124 results) S ['21453498'], E2F1 expression (115 results) S ['21453498']

PMC3076272	5	FALSE	20	FALSE		5	22	TRUE	19	TRUE	17	TRUE	16	TRU
PMC3069219	1	FALSE	8	FALSE		6	8	TRUE	6	TRUE	6	TRUE	5	TRU
PMC3068910	6	FALSE	21	FALSE		7	27	TRUE	26	TRUE	20	TRUE	18	TRU
PMC3077328	1	FALSE	13	FALSE		4	6	FALSE	6	FALSE	6	TRUE	5	TRU
PMC3071494	1	FALSE	0	TRUE		1	1	TRUE	0	TRUE	0	TRUE	0	TRU

JE	TRUE	13	TRUE	relative LCGU (2 results) S ['21453518'], cerebral glucose (2369 results) S ['21453518'], relative local cerebral glucose (121 results) S ['21453518'], relative local cerebral (2106 results) S ['21453518'], central CRF system (10 results) S ['19428773'], glucose utilization (6323 results) S ['21453518'], total serum immunoreactive neurophysins (5 results) D ['6675037'], LCGU (305 results) S ['21453518', '19428773'], vivo evidence for ligand-specific (10 results) S ['19428773'], fiber tract lesions implicate (2 results) D ['1333341'], relative LCGU after restraint (1 results) S ['21453518'], central CRF (136 results) S ['19428773'], LCGU after restraint (1 results) S ['21453518'], tract lesions implicate (17 results) D ['1333341'], evidence for ligand-specific (65 results) S ['19428773'], corticotropin-releasing (12602 results) S ['21453518'], restraint stress (2985 results) S ['21453518'], local cerebral glucose (753 results) S ['21453518', '19428773'], restraint stress on glucose (302 results) S ['21453518'], cerebral glucose utilization (951 results) S ['21453518', '19428773'], local cerebral glucose utilization (612 results) S ['21453518', '19428773'], CRF system (150 results) S ['21453518'], hindlimb restraint (1 results) D ['3417799'], serum immunoreactive neurophysins (25 results) D ['6675037'], ligand-specific receptor (31 results) S ['19428773']
JE	TRUE	3	TRUE	South-East Region Prostate Cancer (6 results) S ['21454449', '18609293', '15548438', '12745718'], Region Prostate Cancer (11559 results) S ['21454449'], Region Prostate Cancer Register (3 results) S ['21454449', '15548438', '12745718'], risk ratio for prostate (2740 results) S ['21454449'], Cancer Register (522 results) S ['21454449'], Prostate Cancer Register (25 results) S ['21454449', '18609293', '18098207'], South-East Region Prostate (6 results) S ['21454449', '18609293', '15548438', '12745718'], ERSPC trial (13 results) D ['19904272'], South-East Region (80 results) S ['21454449', '18609293']
JE	TRUE	13	TRUE	mutations 581G (8 results) S ['20573194'], co-trimoxazole prophylaxis (53 results) S ['21454456'], quintuple mutant (53 results) S ['20439953'], co-trimoxazole against malaria (18 results) D ['16235184'], co-trimoxazole against malaria (18 results) S ['21454456', '20573194'], antifolate resistance (135 results) D ['20209126'], antifolate resistance (135 results) S ['21454456', '20573194', '20439953'], trial co-trimoxazole prophylaxis (346 results) S ['21454456'], co-trimoxazole (2001 results) S ['21454456'], Effect of co-trimoxazole (534 results) S ['20573194'], HIV exposure until years (51 results) S ['21454456'], HIV negative after cessation (38 results) S ['21454456'], Mozambican single-blind (2 results) D ['16338450'], protective efficacy of co-trimoxazole (9 results) D ['16235184'], protective efficacy of co-trimoxazole (9 results) S ['21454456', '20573194'], efficacy of co-trimoxazole (605 results) S ['21454456', '20573194'], antifolate (1564 results) S ['21454456'], co-trimoxazole prophylaxis against malaria (9 results) D ['16235184'], co-trimoxazole prophylaxis against malaria (9 results) S ['21454456', '20573194'], co-trimoxazole prophylaxis protects (6 results) S ['20573194'], prophylaxis protects children (343 results) S ['20573194'], new episodes of malaria (116 results) D ['20209126'], new episodes of malaria (116 results) S ['19877969'], association between co-trimoxazole (186 results) S ['20439953'], protective efficacy (2624 results) S ['21454456'], Tororo (94 results) S ['21454456', '20439953'], prophylaxis protects HIV (151 results) S ['20573194']
JE	TRUE	4	TRUE	fibrotic disease (274 results) S ['21453480'], sulfate-dependent ERK activation contributes (1 results) S ['18240216'], Constitutive thrombospondin-1 (22 results) D ['15855645'], scleroderma (18687 results) S ['21453480'], Heparan sulfate-dependent ERK (1 results) S ['18240216'], multistation tensioning-culture force monitor (1 results) S ['21453480'], multistation tensioning-culture (1 results) S ['21453480'], multistation tensioning-culture force (1 results) S ['21453480'], scleroderma fibroblasts (186 results) S ['21453480'], sulfate-dependent ERK (1 results) S ['18240216'], Matrix contraction (129 results) S ['21453480'], Heparan sulfate-dependent ERK activation (1 results) S ['18240216'], chronic fibrotic disease (11 results) S ['16314481'], sulfate-dependent ERK activation (1 results) S ['18240216']
JE	FALSE	0	FALSE	new applications of MEMRI (2 results) D ['15617052']

PMC3071499	0	TRUE	6	FALSE		2	3	TRUE	2	TRUE	2	TRUE	2	TRU
PMC3071977	7	FALSE	8	FALSE		10	14	TRUE	8	TRUE	6	TRUE	4	TRU
PMC3071492	5	FALSE	0	TRUE		3	5	FALSE	5	TRUE	2	TRUE	1	TRU
PMC3071501	1	FALSE	8	FALSE		2	9	TRUE	6	TRUE	4	TRUE	1	TRU
PMC3071500	2	FALSE	4	FALSE		7	6	TRUE	4	TRUE	1	TRUE	1	TRU
PMC3078831	1	FALSE	13	FALSE		3	14	TRUE	13	TRUE	13	TRUE	13	TRU
PMC3076507	15	FALSE	15	FALSE		19	24	TRUE	23	TRUE	21	TRUE	14	TRU

JE	TRUE	0	FALSE	personality predicts dopamine-dependent (1 results) S ['17507572'], capacity differences (29 results) S ['16180627'], Impulsive personality predicts dopamine-dependent (1 results) S ['17507572'], personality predicts dopamine-dependent changes (1 results) S ['17507572'], frontostriatal activity during component (3 results) S ['17507572'], Impulsive personality predicts (29 results) S ['17507572']
JE	TRUE	1	TRUE	cortical gustatory area (13 results) D ['6716115', '6468568'], fluid self-administration (7 results) S ['15574797'], cortex reflects disengagement (4 results) S ['15574797'], BLA stimulation (26 results) D ['19028592'], rat gustatory cortex reflects (3 results) S ['15574797'], gustatory cortex (121 results) D ['20164341', '19634934'], amygdalo-cortical (17 results) D ['16307602'], gustatory cortex reflects disengagement (2 results) S ['15574797'], aversion retention (5 results) D ['10661514'], reflects disengagement (22 results) S ['15574797'], gustatory cortex reflects (7 results) S ['15574797'], amygdala-cortical ensembles (1 results) S ['18337417'], fluid self-administration task (2 results) S ['15574797'], taste aversion retention (5 results) D ['10661514'], limbic connections (28 results) D ['1719041']
JE	TRUE	0	FALSE	Wisconsin Brief Pain Questionnaire (6 results) D ['6646795'], Gill Pain Questionnaire (9 results) D ['3757388'], Wisconsin Brief Pain (13 results) D ['6646795'], Brief Pain Questionnaire (8 results) D ['6646795'], McGill pain from description (65 results) D ['15983473']
JE	TRUE	0	FALSE	chronic PTZ regime (2 results) S ['19154620'], Drosophila systems (18 results) S ['19154620'], Drosophila systems model (2 results) S ['19154620'], systems model of pentylenetetrazole (30 results) S ['19154620'], locomotor plasticity responsive (9 results) S ['19154620'], PTZ regime (2 results) S ['19154620'], chronic PTZ (16 results) D ['18034659'], chronic PTZ (16 results) S ['19154620'], locomotor plasticity (10 results) S ['19154620']
JE	TRUE	0	FALSE	Vasudevan Wolf (6 results) D ['12969510'], postsynaptic proteome (3 results) S ['16635246'], NMDA receptor cytoplasmic interaction (82 results) S ['18197970'], postsynaptic complexes (9 results) D ['17623647'], postsynaptic complexes (9 results) S ['19455133', '15572359'], hPSD (5 results) S ['21170055']
JE	TRUE	9	TRUE	girls' BMI (88 results) S ['21453516', '20965379'], family environment factors (23 results) S ['21453516'], weight-related (996 results) S ['21453516'], adolescent girls' family environments (53 results) S ['21453516'], mutually-adjusted (124 results) S ['21453516'], adolescents' activity (444 results) S ['21453516'], environment factors (447 results) S ['21453516'], girls' behavior (888 results) S ['21453516'], family environment (1627 results) S ['21453516'], girls' physical activity (167 results) D ['20398306'], girls' physical activity (167 results) S ['21453516', '20965379'], adolescent girls (3718 results) S ['21453516'], girls' intake (83 results) S ['21453516', '20965379'], DK063107 (4 results) S ['20965379']
JE	TRUE	11	TRUE	furnissii oligopeptide permease (1 results) S ['17873048'], furnissii oligopeptide (1 results) S ['17873048'], Vibrio furnissii oligopeptide (1 results) S ['17873048'], hemolytic (47390 results) S ['21494434'], hemolytic activity (3579 results) S ['21494434'], Vibrio furnissii oligopeptide permease (1 results) S ['17873048'], bacterial reversible amyloid (10 results) D ['16026154'], Vibrio hollisae (37 results) D ['17704283', '13130058', '8997558', '8871530', '7591167', '7790052', '7929777'], hollisae hemolysin (20 results) D ['8871530', '1884983', '2592352', '2584761', '2513246', '3178217', '3094925'], hollisae hemolysin (20 results) S ['21494434'], Arrhenius effect (13 results) D ['16026154'], Arrhenius effect (13 results) S ['21494434'], Vibrio hollisae hemolysin (19 results) D ['8871530', '1884983', '2592352', '2584761', '2513246', '3178217', '3094925'], hollisae infection (3 results) D ['17704283', '8997558', '8011809'], direct hemolysin from vibrio (302 results) D ['20335168'], thermostable direct hemolysin (272 results) D ['20335168'], thermostable direct hemolysin (272 results) S ['21494434'], bacterial reversible amyloid toxin (2 results) D ['16026154'], direct hemolysin (280 results) D ['20335168'], direct hemolysin (280 results) S ['21494434'], hollisae (62 results) D ['17704283', '13130058'], hollisae (62 results) S ['21494434'], hollisae strain (13 results) D ['1884983', '3178217', '7076812'], Arrhenius (6103 results) S ['21494434'], Gh-rTDH (1 results) S ['21494434'], direct hemolysin of Vibrio (302 results) D ['20335168'], thermostable (5551 results) S ['21494434'], hemolysin (10410 results) S ['21494434'], Vp-TDH (21 results) D ['8871530', '2592352', '2513246', '3094925'], reversible amyloid toxin (9 results) D ['16026154']

PMC3070658	2	FALSE	11	FALSE		2	13	TRUE	12	TRUE	12	TRUE	12	TRU
PMC3076569	0	TRUE	0	TRUE		0	0	FALSE	0	FALSE	0	FALSE	0	FAL
PMC3076566	0	TRUE	0	TRUE		0	0	FALSE	0	FALSE	0	FALSE	0	FAL
PMC3077325	4	FALSE	5	FALSE		5	8	TRUE	6	TRUE	4	TRUE	4	TRU
PMC3077335	1	FALSE	13	FALSE		3	14	TRUE	7	TRUE	7	TRUE	4	TRU
PMC3077319	3	FALSE	10	FALSE		6	13	TRUE	12	TRUE	9	TRUE	8	TRU
PMC3074526	0	TRUE	21	FALSE		2	19	TRUE	14	TRUE	12	TRUE	12	TRU
PMC3076247	3	FALSE	9	FALSE		7	12	TRUE	11	TRUE	11	TRUE	9	TRU

JE	TRUE	9	TRUE	people with somatic (890 results) S ['21468299'], individuals with somatic (2498 results) S ['21468299'], substantive theory (184 results) S ['21468299'], subjective well-being (1120 results) S ['21468299'], lower expectations (81 results) S ['21468299'], Australian rural women's ways (2 results) D ['17935458'], calibrating (1615 results) S ['21468299'], somatic health (246 results) S ['21468299'], important others (73 results) S ['21468299'], adjustment process (192 results) S ['21468299'], somatic health problems (32 results) S ['21468299'], rural women's ways (107 results) D ['17935458'], main concern (687 results) S ['21468299']
.SE	FALSE	0	FALSE	searches
.SE	FALSE	0	FALSE	searches
JE	TRUE	3	TRUE	intracellular calcein fluorescence (9 results) S ['21453502'], LS081 (1 results) S ['21453502'], CD95 ligand mRNA expression (4 results) D ['9353266'], iron facilitation (43 results) S ['21453502'], Iron Import Proteins (5 results) D ['18223212', '16641131'], Caco2 cells (238 results) S ['21453502'], molecule inhibitors of divalent (174 results) D ['19179627'], iron uptake (2753 results) S ['21453502'], cellular iron import (6 results) D ['18223212']
JE	TRUE	3	TRUE	environmental health speciality (19 results) S ['17765014'], health speciality units (2 results) S ['17765014'], pediatric environmental history (2 results) S ['21453474'], herbal supplements (271 results) S ['21453474'], Horsetail remedies (3 results) D ['10589442'], Horsetail remedies (3 results) S ['21453474'], environmental health speciality units (2 results) S ['17765014'], horsetail (79 results) S ['21453474'], thiaminase (181 results) S ['21453474'], Paediatric environmental health speciality (4 results) S ['17765014'], pediatric environmental (17 results) S ['21453474'], horsetail exposure (3 results) S ['21453474'], Paediatric environmental health (11 results) S ['17765014'], thiamine (13215 results) S ['21453474']
JE	TRUE	5	TRUE	natural killer cells (33404 results) S ['21453520'], anti-inflammatory properties via IGF-1 (9 results) S ['19457161'], atorvastatin-associated (6 results) S ['17981803'], HMG-CoA (6386 results) S ['21453520'], NCAM-derived (11 results) S ['19457161', '18468731'], atorvastatin on blood-brain (16 results) D ['15854590'], barrier permeability during L-NAME (62 results) D ['15854590'], interferon-gamma modulation (4 results) S ['19457161'], HMG-CoA reductase inhibitor (1363 results) S ['21453520'], HMG-CoA reductase (5995 results) S ['21453520'], reductase inhibitor (3957 results) S ['21453520'], properties via IGF-1 (73 results) S ['19457161'], innate producers of interferon-gamma (34 results) D ['15619519']
JE	TRUE	11	TRUE	ambulance specific stressors (2 results) S ['21450112', '19046466'], emotional exhaustion (751 results) S ['21450112'], nationwide study of Norwegian (146 results) S ['21450112'], whereas health complaints (403 results) S ['21450112'], health complaints (830 results) S ['21450112'], operational Norwegian ambulance (6 results) S ['21450112', '19046466'], personality variable neuroticism (156 results) S ['21450112'], Norwegian ambulance personnel (4 results) S ['21450112', '19046466'], serious operational tasks (9 results) S ['19046466'], operational Norwegian ambulance personnel (4 results) S ['21450112', '19046466'], musculoskeletal pain (2173 results) S ['21450112'], stressor serious operational (1 results) S ['19046466'], Norwegian ambulance (191 results) S ['21450112'], specific stressors (156 results) S ['21450112'], higher levels of musculoskeletal (548 results) S ['21450112'], ambulance work (10 results) S ['19046466'], health complaints exhaustion (80 results) S ['21450112'], variable neuroticism (4 results) S ['21450112'], whereas ambulance specific physical (2 results) S ['21450112'], one-year follow-up (3780 results) S ['21450112'], ambulance personnel (236 results) S ['21450112']
JE	TRUE	4	TRUE	negative breast cancer (2089 results) S ['21453513'], antigen loss (205 results) S ['21453513'], Kmieciak (60 results) S ['21453513', '19849846', '18381452', '17304628'], generates breast cancer stem (17 results) S ['19276366'], Manjili (43 results) S ['21453513', '19849846', '19276366', '18381452'], immune pressure (285 results) S ['21453513'], Immune-induced (126 results) S ['19276366'], IFNGR-2 subunits (4 results) D ['10888113'], interleukin-12 burst (44 results) D ['17293384'], vivo generates breast cancer (43 results) S ['19276366'], vivo generates breast (48 results) S ['19276366'], interleukin-12 burst secretion (11 results) D ['17293384']

PMC3078904	10	FALSE	7	FALSE		16	17	TRUE	16	TRUE	15	TRUE	15	TRU
PMC3076273	5	FALSE	16	FALSE		3	19	TRUE	14	TRUE	12	TRUE	10	TRU
PMC3076248	1	FALSE	18	FALSE		2	19	TRUE	18	TRUE	18	TRUE	16	TRU
PMC3068929	3	FALSE	14	FALSE		4	13	TRUE	11	TRUE	9	TRUE	7	TRU

JE	TRUE	8	TRUE	measure of intraerythrocytic (30 results) D ['19017530'], malaria drug resistance challenge (105 results) D ['19666223'], IIIA on monocytes (146 results) D ['20231419'], flow cytometer (2711 results) S ['21453521'], Hz-containing (8 results) S ['21453521', '19917088', '18549498'], Hanscheid (74 results) S ['21453521', '19917088', '18549498'], semi-immune patients from flow (3 results) S ['11590625'], hematological predictors of disease (60 results) D ['20201087', '20196168'], parasite inhibitory (28 results) D ['19851453'], receptor IIIA on monocytes (59 results) D ['20231419'], depolarized (8910 results) S ['21453521'], cytometer (3138 results) S ['21453521'], Hematological predictors (138 results) D ['20201087', '20196168'], Abbott Cell-Dyn hematology analyzer (28 results) D ['14641842'], Grobusch (117 results) S ['21453521', '19917088'], malarial pigment (106 results) D ['20732999', '20637890', '20196168', '20041181', '19086817'], Reliability of antimalarial (66 results) D ['20220159']
JE	TRUE	7	TRUE	IPTp (88 results) S ['21453493'], high transmission areas (58 results) S ['21453493'], stable endemic (686 results) S ['21453493'], women during ANVs (2 results) S ['21453493'], intermittent preventive treatment (272 results) S ['21453493'], Massougbodji (75 results) S ['21453493'], intermittent preventive (728 results) S ['21453493'], last dose of IPTp (2 results) S ['21453493'], ANVs (4 results) S ['21453493'], monthly sulfadoxine-pyrimethamine intermittent (20 results) D ['16826475', '9840604'], high transmission (678 results) S ['21453493'], prospective routine ANVs (1 results) S ['21453493'], preventive treatment (2789 results) S ['21453493'], monthly sulfadoxine-pyrimethamine intermittent preventive (16 results) D ['16826475'], versus monthly sulfadoxine-pyrimethamine (8 results) D ['16826475'], 2-dose versus monthly sulfadoxine-pyrimethamine (3 results) D ['16826475'], malaria infection (1805 results) S ['21453493'], versus monthly sulfadoxine-pyrimethamine intermittent (7 results) D ['16826475'], ANVs during pregnancy (1 results) S ['21453493'], transmission areas (158 results) S ['21453493'], stable endemic areas (3 results) S ['21453493']
JE	TRUE	15	TRUE	vivax malaria (2766 results) S ['21453465'], Parasitological Chloroquine (232 results) S ['21453465'], chloroquine (15939 results) S ['21453465'], malaria endemic areas (517 results) S ['21453465'], endemic areas of Ethiopia (106 results) S ['21453465'], CRPv strains (12 results) S ['21453465'], malaria endemic (1436 results) S ['21453465'], emergence of CRPv (2 results) S ['21453465'], chloroquine resistant (1691 results) S ['21453465'], Halaba (26 results) S ['21453465'], recurrence of parasitemia (200 results) S ['21453465'], parasitemia (5945 results) S ['21453465'], report on CRPv (18 results) S ['21453465'], efficacy of chloroquine (1143 results) S ['21453465'], Therapeutic efficacy of chloroquine (931 results) S ['21453465'], parasitemia of relapse (246 results) D ['19835832'], parasitemia of relapse (246 results) S ['21453465'], Plasmodium vivax (4163 results) S ['21453465'], areas of Ethiopia (606 results) S ['21453465']
JE	TRUE	4	TRUE	succinate (25490 results) S ['21483483'], bovine heart ubiquinol-cytochrome (9 results) D ['9211907'], electron transport (41794 results) S ['21483483'], stopped-flow rapid-scan spectrophotometry (5 results) D ['9211907'], rapid-scan spectrophotometry (13 results) D ['9211907'], respiration underlies (97 results) S ['20041200'], mitochondrial respiration underlies paradoxical (1 results) S ['20041200'], Bistability of mitochondrial (9 results) S ['21483483', '20041200'], respiration underlies paradoxical reactive (1 results) S ['20041200'], paradoxical reactive oxygen (111 results) S ['20041200'], paradoxical reactive oxygen species (160 results) S ['20041200'], underlies paradoxical reactive oxygen (1 results) S ['20041200'], underlies paradoxical reactive (2 results) S ['20041200'], metabolomic estimation of metabolic (12 results) S ['17000750'], respiration underlies paradoxical (1 results) S ['20041200'], mitochondrial respiration underlies (24 results) S ['20041200'], underlies paradoxical (33 results) S ['20041200']

PMC3068927	5	FALSE	10	FALSE		8	11	TRUE	10	TRUE	9	TRUE	8	TRU
PMC3068926	6	FALSE	13	FALSE		6	18	FALSE	18	TRUE	17	TRUE	13	TRU
PMC3068925	4	FALSE	2	FALSE		4	4	FALSE	4	TRUE	3	TRUE	2	TRU
PMC3068924	5	FALSE	4	FALSE		2	9	TRUE	7	TRUE	7	TRUE	5	TRU
PMC3068921	1	FALSE	11	FALSE		2	12	TRUE	11	TRUE	11	TRUE	8	TRU

JE	TRUE	5	TRUE	HSPA1A-related (1 results) S ['21483481'], chemical-protein interactome (5 results) S ['21483481', '20221449', '19629158', '19417066'], HSP70-2 variants (6 results) D ['7579351'], agranulocytosis (8027 results) S ['21483481'], antithesis chemical-protein interactome (1 results) S ['21483481'], chemical-protein (89 results) S ['21483481', '20221449', '19629158'], interactome (754 results) S ['21483481'], immunologic explanatory (27 results) D ['16955993'], clozapine-induced (583 results) S ['21483481'], antithesis chemical-protein (1 results) S ['21483481'], clozapine (8657 results) S ['21483481'], reveals common functional modules (20 results) D ['20140234'], human disease similarities reveals (97 results) D ['20140234'], site-moiety map (1 results) D ['20519201'], PLoS Comput Biol (1644 results) S ['21483481']
JE	TRUE	4	TRUE	KT2440 metabolic (32 results) D ['20540110', '18793442'], KT2440 metabolic (32 results) S ['18974823'], putida KT2440 metabolic (32 results) D ['20540110', '18793442'], putida KT2440 metabolic (32 results) S ['18974823'], Pseudomonas putida (4863 results) S ['21483480'], Genome-scale (1497 results) S ['21483480'], KT2440 metabolic network facilitates (1 results) S ['18974823'], Genome-scale reconstruction (32 results) D ['18793442'], Genome-scale reconstruction (32 results) S ['20709898', '19888215', '18974823'], network facilitates applications (34 results) S ['18974823'], KT2440 metabolic network (12 results) D ['20540110', '18793442'], KT2440 metabolic network (12 results) S ['18974823'], putida (5172 results) S ['21483480'], putida KT2440 metabolic network (12 results) D ['20540110', '18793442'], putida KT2440 metabolic network (12 results) S ['18974823'], Pseudomonas putida KT2440 metabolic (32 results) D ['20540110', '18793442'], Pseudomonas putida KT2440 metabolic (32 results) S ['18974823'], metabolic network (965 results) S ['21483480'], metabolic network facilitates applications (6 results) S ['18974823']
JE	TRUE	1	TRUE	Review Shape-dependent control (6 results) D ['11082279'], ActA polarity (1 results) D ['19593363'], Review Shape-dependent (18 results) D ['11082279'], energy foldings of eukaryotic (1 results) S ['3669684'], speed of Listeria (51 results) D ['19593363'], PLoS Comput Biol (1644 results) S ['21483479']
JE	TRUE	4	TRUE	somatic mutation data set (146 results) S ['21483478'], Dynamic modularity (3 results) D ['19182785'], networks predicts breast (16 results) D ['19182785'], interaction networks predicts breast (2 results) D ['19182785'], protein interaction networks predicts (55 results) D ['19182785'], networks predicts breast cancer (15 results) D ['19182785'], protein interaction networks (757 results) S ['21483478'], cancer outcome (393 results) S ['21483478'], interaction networks (1345 results) S ['21483478']
JE	TRUE	4	TRUE	hand position (385 results) S ['21483475'], Bayesian observer (18 results) D ['16547513'], Bayesian observer (18 results) S ['21483475'], prior over visuomotor (16 results) S ['21483475'], full covariance structure (3 results) S ['21483475'], visuomotor transformations (77 results) S ['21483475'], covariance structure (563 results) S ['21483475'], visuomotor (2432 results) S ['21483475'], observer model (73 results) S ['21483475'], sensorimotor task (69 results) S ['21483475'], prior covariance (398 results) S ['21483475'], Bayesian observer model (75 results) S ['21483475']

PMC3069122	9	FALSE	15	FALSE		12	19	TRUE	16	TRUE	9	TRUE	4	TRU
PMC3069119	9	FALSE	18	FALSE		6	23	TRUE	18	TRUE	15	TRUE	14	TRU
PMC3069118	0	TRUE	2	FALSE		1	2	FALSE	2	FALSE	2	FALSE	2	FAL
PMC3069113	10	FALSE	5	FALSE		13	15	TRUE	10	TRUE	8	TRUE	8	TRU
PMC3069109	6	FALSE	14	FALSE		9	19	TRUE	15	TRUE	9	TRUE	8	TRU

JE	TRUE	2	TRUE	AGO1 (254 results) S ['21483759'], hua1 hua2 (7 results) S ['12913148', '12530963', '11923195', '11874905', '10198637'], determinacy defects (12 results) S ['12530963'], ZWILLE potentiates (1 results) D ['18653559'], HD-Zip (140 results) S ['21483759'], hua2 (10 results) S ['12913148', '12530963', '11923195', '11874905', '10198637'], ZWILLE potentiates WUSCHEL (1 results) D ['18653559'], hua1 hua2 hen6 (7 results) S ['12913148', '12530963', '11923195', '11874905', '10198637'], floral determinacy defects (9 results) S ['12530963'], ZWILLE potentiates WUSCHEL function (1 results) D ['18653559'], ARGONAUTE1 (55 results) D ['19763164'], ARGONAUTE1 (55 results) S ['21483759'], potentiates WUSCHEL (1 results) D ['18653559'], HD-Zip genes (16 results) S ['21483759'], potentiates WUSCHEL function (1 results) D ['18653559'], hua1 hen7 (11 results) S ['12913148', '12530963', '11923195', '11874905', '10198637'], hua2 hen6 (10 results) S ['12913148', '12530963', '11923195', '11874905', '10198637'], hua1 (11 results) S ['12913148', '12530963', '11923195', '11874905', '10198637'], floral determinacy (16 results) D ['11440722'], floral determinacy (16 results) S ['12530963', '11874905'], functions of miR172 (6 results) S ['21483759', '17573799'], AGO10 (8 results) D ['19763164', '18483398'], AGO10 (8 results) S ['21483759'], recruits microRNAs (16 results) D ['16081530']
JE	TRUE	8	TRUE	Acetobacter genetic organization (7 results) D ['9721278'], diguanylate (176 results) S ['21483756'], novel diguanylate cyclases participate (1 results) S ['19888998'], cholerae (8944 results) S ['21483756'], CD0757 (1 results) S ['21483756'], novel diguanylate (32 results) D ['18502872'], novel diguanylate (32 results) S ['19888998'], diguanylate cyclases participate (4 results) S ['19888998'], cyclases (1948 results) S ['21483756'], GGDEF (146 results) D ['20576684'], GGDEF (146 results) S ['21483756'], encode novel diguanylate cyclases (2 results) D ['18502872'], encode novel diguanylate cyclases (2 results) S ['19888998'], diguanylate cyclases (44 results) D ['20576684'], diguanylate cyclases (44 results) S ['21483756', '19888998'], functional DGCs (15 results) S ['21483756'], encode novel diguanylate (2 results) D ['18502872'], encode novel diguanylate (2 results) S ['19888998'], activity of CD0757 (1 results) S ['21483756'], Vibrio cholerae (8447 results) S ['21483756'], Di-GMP (299 results) S ['21483756'], exopolysaccharide through regulation (345 results) D ['20576684'], versions of CD0522 (1 results) S ['21483756'], novel diguanylate cyclases (10 results) D ['18502872'], novel diguanylate cyclases (10 results) S ['19888998'], cyclases participate (21 results) S ['19888998'], biotype Vibrio cholerae characterization (47 results) D ['18227161']
.SE	TRUE	2	TRUE	Menendez (2000 results) S ['21483755'], noncanonical (1465 results) S ['21483755']
JE	TRUE	3	TRUE	LYS4 trimethylation correlates (2 results) D ['17652096'], euchromatic factors Set1 (5 results) D ['17925448', '17898715', '12353038', '11752412'], lysine on histone (6078 results) S ['21483810'], trimethylation correlates with degree (4 results) D ['17652096'], factors Set1 (96 results) D ['19379692', '19160454'], Global assessment of combinatorial (9 results) D ['17652096'], deacetylases Hst1p (8 results) D ['17676954', '11316790'], Set1 (181 results) S ['21483810'], Hst1 (90 results) D ['19379692'], Hst1 (90 results) S ['21483810'], trimethylation correlates (41 results) D ['18849979'], histone modifications (2144 results) S ['21483810'], H3K4ac (2 results) D ['20299449'], H3K4ac (2 results) S ['21483810'], Genome-wide analysis of mono- (23 results) D ['19508735']
JE	TRUE	4	TRUE	brca2 (5025 results) S ['21483806'], zebrafish spermatogenesis (2 results) S ['21483806'], Fanconi gene (1246 results) S ['21483806'], zebrafish sex differentiation (77 results) S ['20661450'], brca2 mutants (9 results) D ['18266476'], brca2 mutants (9 results) S ['21483806'], Fanconi (4941 results) S ['21483806'], ovary-like tissue (2 results) D ['11914381'], zebrafish Anti-Mullerian (7 results) D ['15768398'], zebrafish Anti-Mullerian (7 results) S ['15939378'], Drosophila brca2 (11 results) D ['18266476'], zebrafish brca2 (4 results) S ['21483806', '19101574'], zebrafish Anti-Mullerian hormone (7 results) D ['15768398'], zebrafish Anti-Mullerian hormone (7 results) S ['15939378'], transition from ovary-like (1 results) D ['11914381'], tp53 mutant zebrafish (5 results) S ['21483806', '20661450', '15630097'], oocyte nuclear architecture (50 results) S ['21483806'], tp53 mutant (38 results) S ['15630097'], tp53 (4955 results) S ['21483806'], Fanconi gene network (24 results) S ['19101574', '16515849']

PMC3069107	3	FALSE	8	FALSE		6	10	TRUE	7	TRUE	5	TRUE	2	TRU
PMC3069106	0	TRUE	22	FALSE		7	11	TRUE	9	TRUE	5	TRUE	5	TRU
PMC3069099	10	FALSE	14	FALSE		10	22	TRUE	14	TRUE	8	TRUE	7	TRU
PMC3070753	6	FALSE	14	FALSE		10	15	TRUE	11	TRUE	9	TRUE	8	TRU

JE	TRUE	2	TRUE	esophageal noninvasive precursor lesions (2 results) S ['16224217'], esophageal noninvasive precursor (2 results) S ['16224217'], HPP1 occurs (7 results) D ['15824739'], LUMA Methylation (9 results) D ['16624287'], gastroesophageal evidence of homozygous (2 results) S ['16224217'], hypomethylation (2220 results) S ['21483804'], High-resolution genome-wide cytosine methylation (14 results) S ['19386619'], neoplastic progression (1157 results) S ['21483804'], LUMA Methylation high throughput (1 results) D ['16624287'], High-resolution genome-wide cytosine (14 results) S ['19386619'], adverse prognostic influence (17 results) S ['19252414']
JE	TRUE	5	TRUE	AIMP2 promotes TNFalpha-dependent (1 results) S ['19584093'], promotes TNFalpha-dependent apoptosis (1 results) S ['19584093'], multi-tRNA synthetase (12 results) S ['20306515', '18695251', '17062567', '10358004'], AIMP2 promotes (2 results) S ['21483803', '19584093'], Multidirectional tumor-suppressive (1 results) S ['19622630'], Multidirectional tumor-suppressive activity (1 results) S ['19622630'], AIMP2 heterozygous mice (1 results) S ['19622630'], multi-tRNA synthetase implications (4 results) S ['20306515', '17062567'], AIMP2 (10 results) S ['21483803', '20306515', '19622630', '19584093', '18695251'], tRNA synthetase (4537 results) S ['21483803'], multi-tRNA synthetase complex (12 results) S ['20306515', '18695251', '17062567', '10358004'], multi-tRNA (12 results) S ['20306515', '18695251', '17062567', '10358004'], synthetase (37322 results) S ['21483803'], synthetase complex (3010 results) S ['21483803'], tRNA (24823 results) S ['21483803'], susceptibility of AIMP2 (3 results) S ['21483803', '19622630', '18695251'], AIMP2-DX2 (1 results) S ['21483803'], promotes TNFalpha-dependent (3 results) S ['19584093'], activity of tRNA (8808 results) S ['21483803'], expression ratio of AIMP2-DX2 (1 results) S ['21483803'], AIMP2 heterozygous (1 results) S ['19622630'], AIMP2 promotes TNFalpha-dependent apoptosis (1 results) S ['19584093']
JE	TRUE	3	TRUE	Myb-like (126 results) S ['21483796', '19805390'], RVE8 (3 results) S ['21483796'], CLOCK ASSOCIATED gene (3 results) D ['17519251'], Constitutive expression of CIR1 (2 results) D ['17587236'], Myb-like transcription factor (22 results) S ['19805390'], ASSOCIATED gene disrupts circadian (9 results) D ['9657154', '9657153'], REVEILLE1 (1 results) S ['19805390'], expression of CIR1 (10 results) D ['17587236'], expression of CIR1 (10 results) S ['19805390'], Myb-like transcription (87 results) S ['21483796', '19805390'], circadian clock (3823 results) S ['21483796'], levels of PRR5 (6 results) D ['19131357', '17693530'], circadian rhythms (7389 results) S ['21483796'], disrupts circadian rhythms (14 results) D ['9657154', '9657153'], CLOCK ASSOCIATED gene disrupts (5 results) D ['9657154', '9657153'], RVE8-OX (1 results) S ['21483796'], auxin pathways (8 results) S ['19805390'], CIR1 affects (2 results) D ['17587236'], CIR1 affects (2 results) S ['19805390'], PRR5 promoter (6 results) D ['20233950'], PRR5 promoter (6 results) S ['21483796'], PRR5 (33 results) D ['20407420', '20233950', '19131357'], PRR5 (33 results) S ['21483796'], RVE8 binds (2 results) S ['21483796']
JE	TRUE	4	TRUE	cyanase activity (12 results) D ['18796334', '18708510', '15691951', '11157213', '9446686', '9294430'], cyanase activity (12 results) S ['21494323'], plant cyanases (1 results) S ['21494323'], source of cyanate (123 results) D ['18708510'], coli cyanase (30 results) D ['18796334', '18708510', '10801492', '9294430', '2495763'], AtCYN (1 results) S ['21494323'], catalytic residues (1408 results) S ['21494323'], level of cyanase (8 results) D ['18796334', '18708510', '11157213', '10801492', '9294430'], level of cyanase (8 results) S ['21494323'], other organs of Arabidopsis (546 results) S ['21494323'], plant cyanase (4 results) D ['10801492'], plant cyanase (4 results) S ['21494323'], AtCYN transcription (1 results) S ['21494323'], roles of cyanases (1 results) S ['21494323'], cyanate (856 results) S ['21494323'], OsCYN (1 results) S ['21494323'], cyanase (43 results) D ['18796334', '18708510', '17122352', '15691951', '11157213', '10801492', '9446686', '9294430'], cyanase (43 results) S ['21494323'], cyanases (2 results) S ['21494323'], KCNO (23 results) S ['21494323']

PMC3069111	10	FALSE	12	FALSE		8	18	TRUE	17	TRUE	14	TRUE	8	TRU
PMC3069108	9	FALSE	7	FALSE		11	14	TRUE	13	TRUE	12	TRUE	8	TRU
PMC3069105	12	FALSE	3	FALSE		2	13	TRUE	10	TRUE	9	TRUE	7	TRU
PMC3069102	4	FALSE	6	FALSE		6	8	FALSE	8	TRUE	6	TRUE	5	TRU
PMC3069101	10	FALSE	10	FALSE		9	17	TRUE	16	TRUE	14	TRUE	13	TRU

JE	TRUE	6	TRUE	glenohumeral (3002 results) S ['21483808'], vivo estimation (136 results) D ['19875120'], vivo estimation (136 results) S ['21483808'], GH-JRC (1 results) S ['21483808'], IHA (1335 results) S ['21483808'], location identification (14 results) D ['19428014'], joint centre location (8 results) D ['19428014', '19268947'], glenohumeral joint (1471 results) S ['21483808'], predictive methods of glenohumeral (24 results) D ['19428014'], joint centre location identification (10 results) D ['19428014'], methods of glenohumeral (1464 results) S ['21483808'], MRI development (10 results) D ['19428014'], repeatability assessment (14 results) D ['19875120'], ISB standardization (11 results) S ['15844264'], geometric GH-JRC (1 results) S ['21483808'], glenohumeral joint rotation center (2 results) S ['21483808', '9596544'], glenohumeral joint centre (4 results) D ['19875120', '19428014', '17631297'], Review ISB recommendation (1 results) S ['15844264'], glenohumeral joint centre location (18 results) D ['19875120', '19428014', '17631297', '11006387'], rotation center (90 results) S ['21483808'], scapular bony landmarks (16 results) D ['11006387'], scapular bony landmarks (16 results) S ['9596544']
JE	TRUE	2	TRUE	Ly49A (127 results) S ['21483805'], RMA display mosaic expression (1 results) S ['10799866'], Ly49 family (69 results) D ['19605564', '15345220'], Ly49 family (69 results) S ['21248256', '17056508'], Ly49 genes (43 results) D ['19605564', '14707081'], Ly49 genes (43 results) S ['21483805', '16750269'], RMA display mosaic (1 results) S ['10799866'], Ly49 (336 results) S ['21483805', '21248256'], distinct Ly-49 MHC (13 results) D ['11342639'], Ly-49 MHC (70 results) D ['11342639'], distinct Ly-49 MHC class (12 results) D ['11342639'], RMA display (14 results) S ['10799866'], mouse Ly49 genes (3 results) D ['11398966'], Ly49A expression (7 results) D ['10023056'], Ly-49 MHC class (66 results) D ['11342639'], distinct Ly-49 (18 results) D ['11342639']
JE	TRUE	2	TRUE	Rac activation signals (323 results) S ['21483802'], rigidity governs smooth muscle (2 results) D ['15669099'], governs smooth muscle (44 results) D ['15669099'], smooth muscle cell motility (23 results) D ['15669099'], Extracellular matrix rigidity governs (1 results) D ['15669099'], governs smooth muscle cell (23 results) D ['15669099'], matrix rigidity governs smooth (1 results) D ['15669099'], muscle cell motility (35 results) D ['15669099'], rigidity governs (28 results) D ['15669099'], rigidity governs smooth (2 results) D ['15669099'], Extracellular matrix rigidity (8 results) D ['15669099'], ECM stiffness (16 results) D ['15669099'], ECM stiffness (16 results) S ['21483802'], migration speed (215 results) S ['21483802'], matrix rigidity governs (3 results) D ['15669099']
JE	TRUE	4	TRUE	self interview (160 results) S ['21187357'], non-CASI (1 results) S ['21483799'], response bias among STD (10 results) D ['16199744', '10342804'], non-CASI periods (1 results) S ['21483799'], sexual health clinics (129 results) D ['20551234'], sexual health clinics (129 results) S ['20975481'], interview modes (19 results) D ['16199744'], CASI affects (9 results) S ['21483799'], bias among STD (97 results) D ['16199744'], sexual history (880 results) S ['21483799']
JE	TRUE	10	TRUE	Heme oxygenase-1 (4696 results) S ['21483798'], sickle mice (44 results) D ['19617415', '16485041', '15962839'], NQO1-null mice lead (1 results) D ['15781611'], SCD (4116 results) S ['21483798'], transgenic sickle mice (21 results) D ['16485041', '15962839', '15665055', '15001449', '12543857'], SCD patients (483 results) S ['21483798'], oxidoreductase (11126 results) S ['21483798'], cell disease (8809 results) S ['21483798'], vaso-occlusion (236 results) S ['21483798'], Beauty inhibits (10 results) D ['20306336'], oxygenase-1 (4911 results) S ['21483798'], Beauty inhibits vascular (1 results) D ['20306336'], Beauty inhibits vascular stasis (1 results) D ['20306336'], NQO1-null mice (5 results) D ['15781611'], cytoprotective enzymes (64 results) S ['21483798'], transgenic sickle (235 results) D ['20306336'], transgenic sickle (235 results) S ['21483798'], inhibits vascular stasis (8 results) D ['20306336', '16485041'], sickle cell disease (7745 results) S ['21483798'], transgenic mice with SCD (97 results) D ['20306336']

PMC3069098	15	FALSE	5	FALSE		6	16	TRUE	13	TRUE	12	TRUE	10	TRU
PMC3069096	0	TRUE	2	FALSE		1	2	FALSE	2	FALSE	2	FALSE	2	FAL
PMC3069095	6	FALSE	11	FALSE		7	15	TRUE	11	TRUE	10	TRUE	9	TRU
PMC3069094	4	FALSE	8	FALSE		4	10	TRUE	9	TRUE	8	TRUE	8	TRU
PMC3069093	1	FALSE	9	FALSE		2	8	TRUE	7	TRUE	6	TRUE	6	TRU
PMC3069092	7	FALSE	10	FALSE		10	15	TRUE	8	TRUE	8	TRUE	8	TRU

JE	TRUE	7	TRUE	induces simultaneous cortical axon (11 results) D ['19420254'], augment neurite (34 results) D ['18817543'], neurite (11178 results) S ['21483795'], H1152 augment neurite (1 results) D ['18817543'], guidance cue for dopaminergic (13 results) D ['19812329', '11353855'], induces simultaneous cortical (122 results) D ['19420254'], anterior-posterior organization (5 results) D ['21106844'], intracortical targeting (28 results) D ['19812329'], H1152 augment neurite extension (1 results) D ['18817543'], cell polarity (10172 results) S ['21483795'], augment neurite extension (5 results) D ['18817543'], simultaneous cortical axon outgrowth (10 results) D ['19420254'], Wnt5a (605 results) S ['21483795'], organization of monoaminergic (93 results) D ['21106844'], H1152 augment (1 results) D ['18817543'], simultaneous cortical axon (197 results) D ['19420254'], midbrain dopaminergic (690 results) S ['21483795'], Wnt5a induces simultaneous cortical (2 results) D ['19420254'], anterior-posterior organization of monoaminergic (1 results) D ['21106844'], axon guidance (1935 results) S ['21483795']
.SE	TRUE	2	TRUE	neurite (11178 results) S ['21483793'], neurite outgrowth (6681 results) S ['21483793']
JE	TRUE	4	TRUE	NFkappaB promotes inflammation (34 results) D ['20150534'], alternate-day (1375 results) S ['21483792'], flavonoid tomato intake (16 results) S ['16920850'], flavonoid tomato intake reduces (1 results) S ['16920850'], Transgenic flavonoid tomato intake (3 results) S ['16920850'], dietary cholesterol (8056 results) S ['21483792'], van Erk (30 results) S ['20098690', '17892536'], Calorie restriction decreases microalbuminuria (2 results) D ['1415585'], restriction modulates renal expression (10 results) D ['15944339'], tomato intake reduces C-reactive (1 results) S ['16920850'], liver inflammation (691 results) S ['21483792'], restriction decreases microalbuminuria (4 results) D ['1415585'], calorie restriction (1249 results) S ['21483792'], restriction modulates renal (22 results) D ['15944339'], Calorie Restriction Modulates Renal (2 results) D ['15944339'], Transgenic flavonoid tomato (30 results) S ['16920850'], cholesterol-free Western diet (23 results) S ['21483792']
JE	TRUE	3	TRUE	metformin induces casein (1 results) S ['17525164'], nutrient signals (71 results) D ['19833968'], cryptochrome phosphorylation (61 results) D ['19833968'], regulates food (3029 results) S ['21483791'], AMP-kinase regulates (2200 results) S ['21483791'], protein ancient energy gauge (3 results) D ['16054041'], AMPK regulates (383 results) S ['21483791'], AMP-kinase regulates food intake (34 results) S ['21483791'], drug metformin induces casein (1 results) S ['17525164'], induces casein kinase Iepsilon (6 results) S ['17525164'], AMP-kinase regulates food (65 results) D ['19833968'], AMP-kinase regulates food (65 results) S ['21483791']
JE	TRUE	4	TRUE	PfPV1 protein (1 results) S ['21483790'], pv1 locus (2 results) S ['21483790'], vacuole (6865 results) S ['21483790'], parasitophorous (1031 results) S ['21483790'], importance of parasitophorous (26 results) S ['21483790'], PfPV1 (1 results) S ['21483790'], pv1 gene (41 results) S ['21483790'], parasitophorous vacuole (792 results) S ['21483790'], pv1 (160 results) S ['21483790'], separate blocks of tandem (8 results) D ['6085696']
JE	TRUE	2	TRUE	A92-308110 (3 results) D ['12656618', '10480571', '10480570'], uptake receptor expression (2 results) D ['15128841'], immunosuppressant (5287 results) S ['21483789'], novel immunosuppressant sanglifehrin (4 results) D ['15772070', '11390463'], immunosuppressant blocks bioactive IL-12 (1 results) S ['12847216'], Sanglifehrins (3 results) D ['10480571', '10480570'], blocks bioactive IL-12 (2 results) S ['12847216'], blocks bioactive IL-12 production (2 results) S ['12847216'], key dendritic cell (2 results) S ['17391124'], antigen uptake receptor expression (2 results) D ['15128841'], Sanglifehrin (52 results) S ['21483789', '17391124'], cyclophilin-binding (57 results) D ['15128841', '15067056'], cyclophilin-binding (57 results) S ['21483789', '17391124'], drug sanglifehrin (31 results) D ['15128841', '15067056'], drug sanglifehrin (31 results) S ['17391124'], SFA (2004 results) S ['21483789'], blocks key dendritic cell (32 results) S ['17391124']

PMC3069091	10	FALSE	11	FALSE		3	20	TRUE	14	TRUE	10	TRUE	5	TRU
PMC3069090	1	FALSE	16	FALSE		3	14	TRUE	9	TRUE	8	TRUE	6	TRU
PMC3069089	12	FALSE	20	FALSE		8	27	TRUE	14	TRUE	10	TRUE	9	TRU

JE	TRUE	2	TRUE	stem cell-like gene (8 results) D ['18443585'], embryonic stem cell-like gene (2 results) D ['18443585'], glioma co-express (16 results) S ['21483788'], high grade glioma co-express (2 results) S ['21483788'], aggressive human tumors (21 results) D ['18443585'], Eset partners with Oct4 (1 results) D ['19884257'], trophoblast lineage potential (48 results) D ['19884257'], stem cell-like gene expression (8 results) D ['18443585'], Sox2 (1473 results) S ['21483788'], glioma cells co-express neural (2 results) S ['21483788'], Nanog (1260 results) S ['21483788'], pluripotent stem cell traits (16 results) S ['21483788'], gliomas mesodermal- (35 results) S ['21483788'], grade gliomas mesodermal- (8 results) S ['21483788'], Persistent presence of pluripotent (10 results) S ['21483788'], partners with Oct4 (12 results) D ['19884257'], grade glioma co-express (4 results) S ['21483788'], extraembryonic trophoblast lineage potential (11 results) D ['19884257'], cell-like gene expression signature (4 results) D ['18443585'], endodermal-specific transcription (5 results) S ['21483788'], cell-like gene expression (15 results) D ['18443585']
JE	TRUE	4	TRUE	T20-insensitive HIV-1 from naive (1 results) S ['15721359'], T20-insensitive HIV-1 (1 results) S ['15721359'], immunodeficiency virus type (17353 results) S ['21483787'], different AZT (499 results) S ['21483787'], human immunodeficiency virus type (17330 results) S ['21483787'], dual-color competition assay (5 results) S ['15721359'], AZT resistance pathway (14 results) D ['16485036'], AZT resistance pathway (14 results) S ['21483787'], novel dual-color competition (2 results) S ['15721359'], T20-insensitive (1 results) S ['15721359'], dual-color competition (7 results) S ['15721359'], epistasis (4060 results) S ['21483787'], novel dual-color competition assay (2 results) S ['15721359'], exhibits high viral fitness (5 results) S ['15721359'], naive patients exhibits (33 results) S ['15721359'], different AZT concentrations (86 results) S ['21483787'], high viral fitness (3 results) S ['15721359']
JE	TRUE	5	TRUE	Histone demethylase JMJD3 contributes (6 results) D ['19779457', '19451218', '19451217'], lysine 27-specific demethylase Jmjd3 (2 results) D ['18716661'], lysine 27-specific demethylase Jmjd3 (2 results) S ['21483786'], JMJD3 contributes (6 results) D ['19779457', '19451218', '19451217'], demethylase (5327 results) S ['21483786'], 27-specific demethylase (3 results) D ['18716661'], 27-specific demethylase (3 results) S ['21483786'], demethylase JMJD3 contributes (6 results) D ['19779457', '19451218', '19451217'], lysine 27-specific demethylase (3 results) D ['18716661'], lysine 27-specific demethylase (3 results) S ['21483786'], JMJD3 induces p53 stabilization (1 results) S ['21483786'], mouse neural stem (85 results) S ['21483786', '20868483'], NSCs through ARF-dependent (1 results) S ['21483786'], mouse neural stem cell (13 results) S ['21483786', '19415678'], JMJD3 induces p53 (1 results) S ['21483786'], Histone demethylase JMJD3 (4 results) D ['19451218'], H3K27me3 (326 results) S ['21483786'], mouse NSC differentiation (292 results) S ['21483786'], subventricular zone implication (10 results) D ['16436596'], JMJD3 (38 results) D ['19779457'], JMJD3 (38 results) S ['21483786'], demethylase JMJD3 (10 results) D ['19451218', '19451217', '18716661'], demethylase JMJD3 (10 results) S ['21483786'], neural stem cell (1415 results) S ['21483786'], common pathway during neurogenesis (54 results) S ['21483786'], 27-specific demethylase Jmjd3 (2 results) D ['18716661'], 27-specific demethylase Jmjd3 (2 results) S ['21483786'], mouse NSCs through ARF-dependent (1 results) S ['21483786'], mouse NSC (10 results) S ['21483786', '19415678'], JMJD3 induces (1 results) S ['21483786'], H3K27me3 demethylase JMJD3 contributes (4 results) D ['19779457', '19451217'], ARF (7183 results) S ['21483786']

PMC3069088	11	FALSE	8	FALSE		11	15	TRUE	12	TRUE	12	TRUE	10	TRU
PMC3069087	6	FALSE	6	FALSE		6	11	TRUE	9	TRUE	7	TRUE	5	TRU
PMC3069086	2	FALSE	10	FALSE		7	11	TRUE	8	TRUE	6	TRUE	5	TRU
PMC3069085	9	FALSE	7	FALSE		6	15	TRUE	9	TRUE	6	TRUE	5	TRU
PMC3069084	0	TRUE	14	FALSE		4	12	TRUE	11	TRUE	9	TRUE	7	TRU

JE	TRUE	4	TRUE	decarboxylases triggers allosteric enzyme (1 results) D ['19246454'], structure assignment (165 results) S ['21483785', '19606500'], structural alphabet (47 results) D ['20525797'], structural alphabet (47 results) S ['19625218'], triggers allosteric enzyme activation (17 results) D ['19246454'], triggers allosteric enzyme (59 results) D ['19246454'], PPII assignment (4 results) S ['21483785'], yeast pyruvate decarboxylases triggers (1 results) D ['19246454'], consensus assignment (4 results) D ['8332595'], consensus assignment (4 results) S ['21483785'], polyproline (954 results) S ['21483785'], decarboxylases triggers (71 results) D ['19246454'], PPII (207 results) S ['21483785'], protein blocks (212 results) S ['19625218'], pyruvate decarboxylases triggers allosteric (1 results) D ['19246454'], Secondary structure assignment (43 results) D ['16351757'], Secondary structure assignment (43 results) S ['21483785', '19606500', '17597878', '16164759'], decarboxylases triggers allosteric (1 results) D ['19246454'], Conservation of polyproline (12 results) D ['15657931', '7756993']
JE	TRUE	5	TRUE	pulmonary GOLD executive (8 results) D ['17507545'], neutrophil chemokine receptor (5 results) D ['15819701'], neutrophilic (6792 results) S ['21483784'], pulmonary GOLD executive summary (4 results) D ['17507545'], neutrophilic bronchitis (12 results) S ['19555716'], Obstructive Pulmonary GOLD Executive (6 results) D ['17507545'], airflow (9408 results) S ['21483784'], sputum relates (20 results) D ['17112387'], diseases with neutrophilic (3106 results) S ['21483784'], airflow obstruction (2785 results) S ['21483784'], neutrophil GM-CSF enhances TLR2 (1 results) D ['12176910'], airway diseases with neutrophilic (349 results) S ['21483784']
JE	TRUE	4	TRUE	Chintala (125 results) S ['21483783'], retinal ganglion qualitative (75 results) D ['19264888'], Optic nerve ligation leads (3 results) S ['14746883'], metalloproteinase-9 promotes retinal degeneration (1 results) S ['15223820'], metalloproteinase-9 promotes retinal (3 results) S ['15223820'], optic nerve (29245 results) S ['21483783'], retinal ganglion (10724 results) S ['21483783'], optic nerve ligation (17 results) S ['14746883', '12813409', '12354772'], matrix metalloproteinase-9 promotes retinal (3 results) S ['15223820'], Neurostatin (10 results) D ['10820217'], Neurostatin (10 results) S ['21483783'], retinal ganglion cells (10031 results) S ['21483783']
JE	TRUE	2	TRUE	human immunodeficiency virus assays (3 results) S ['18353938'], HIV RDTs (8 results) D ['19197370'], HIV RDTs (8 results) S ['21483842'], RDT operators (3 results) S ['21483842'], rapid human immunodeficiency (58 results) D ['19172705'], rapid human immunodeficiency virus (57 results) D ['19172705'], RDT results (22 results) S ['21483842'], RDT (456 results) S ['21483842'], cost-effectiveness of Capillus (1 results) D ['18923015'], immunodeficiency virus assays (3 results) S ['18353938'], RDTs (168 results) S ['21483842'], immunodeficiency virus antibody assays (3 results) D ['18923015'], Inaccurate diagnosis of HIV-1 (6 results) D ['19893738'], prevention demonstration project (14 results) D ['19172705'], Afwamba (2 results) D ['18923015'], HIV prevention demonstration project (3 results) D ['19172705']
JE	TRUE	4	TRUE	Phylogeographic reconstruction (6 results) S ['19922616'], dissimilar geographic (57 results) S ['17933898'], distinct groups within Burkholderia (29 results) S ['17933898'], Within-host evolution of Burkholderia (1 results) S ['20090837'], transfer event defines (8 results) S ['17933898'], Burkholderia pseudomallei (1273 results) S ['21483841'], pseudomallei (1631 results) S ['21483841'], Within-host evolution (19 results) S ['20090837'], Burkholderia (4406 results) S ['21483841'], pseudomallei congruent (1 results) S ['21483841'], acute melioidosis (30 results) S ['20090837'], melioidosis (1627 results) S ['21483841'], dissimilar geographic distributions (19 results) S ['17933898'], gene transfer event defines (4 results) S ['17933898']

PMC3069083	4	FALSE	19	FALSE		7	20	TRUE	8	TRUE	8	TRUE	8	TRU
PMC3069081	6	FALSE	3	FALSE		5	9	TRUE	6	TRUE	5	TRUE	3	TRU
PMC3069080	3	FALSE	18	FALSE		8	21	TRUE	17	TRUE	13	TRUE	11	TRU
PMC3069079	5	FALSE	17	FALSE		6	21	TRUE	17	TRUE	12	TRUE	9	TRU
PMC3069078	9	FALSE	3	FALSE		8	12	TRUE	9	TRUE	7	TRUE	7	TRU

JE	TRUE	6	TRUE	defense peptides (176 results) S ['21483840'], systemic inoculation of histidine-rich (1 results) S ['19351852'], parent L-hexapeptide (2 results) D ['8253750'], L-hexapeptide (5 results) D ['8253750'], sledgehammer for drug (3 results) S ['19865528'], L-amino Acid membrane lysis (4 results) S ['15313920'], tissue sarcoma (3816 results) S ['21483840'], host defense-like lytic (3 results) S ['21483840', '19351852'], Host defense peptides (145 results) S ['21483840'], human solid tumor growth (3 results) S ['19351852'], Anticancer alpha-helical (27 results) D ['17168782'], soft tissue sarcoma (3799 results) S ['21483840'], lytic peptides (84 results) S ['19351852'], Host defense (11554 results) S ['21483840'], host defense-like (5 results) S ['21483840', '19351852'], pH-dependent host defense-like (1 results) S ['19351852'], L-amino Acid membrane (383 results) S ['21483840'], anticancer dawn (4 results) D ['17426345'], host defense-like lytic peptides (3 results) S ['21483840', '19351852'], defense-like lytic peptides (3 results) S ['21483840', '19351852'], pH-dependent host defense-like lytic (1 results) S ['19351852'], defense-like lytic (3 results) S ['21483840', '19351852'], inoculation of histidine-rich (5 results) S ['19351852']
JE	TRUE	2	TRUE	multiple phosphoproteins (6 results) D ['15020100'], microwave fixation system (60 results) S ['21483838'], frontal cortex of mice (2288 results) S ['21483838'], antidepressant sertraline improves (19 results) D ['18096160'], brain preserves (584 results) S ['21483838'], neurobehavioural stress research (4 results) D ['15814150'], potential utility for neurobehavioural (4 results) D ['15814150'], utility for neurobehavioural (12 results) D ['15814150'], Synaptic imbalance (3 results) D ['18550748']
JE	TRUE	7	TRUE	Dnmt3L allele (24 results) S ['21483837'], spermatids (6606 results) S ['21483837'], germ cells from Dnmt3L (48 results) D ['18663141', '17875220'], germ cells from Dnmt3L (48 results) S ['21483837'], human Klinefelter's (1339 results) S ['21483837'], DNMT3L production (4 results) S ['15753313'], aneuploidy (13718 results) S ['21483837'], Dnmt3L (120 results) S ['21483837'], Dnmt3L heterozygous males (3 results) S ['21483837'], ion channel regulator (6 results) S ['18502891'], Dnmt3L expression (8 results) D ['17060371'], Dnmt3L expression (8 results) S ['15753313'], Dnmt3L heterozygous (3 results) S ['21483837'], Dnmt3L allele results (10 results) S ['21483837'], gametogenetin (11 results) S ['18502891'], cells from Dnmt3L (84 results) S ['21483837'], Characterization of gametogenetin (3 results) S ['18502891'], haploid (8298 results) S ['21483837'], DNMT3L deficiency (15 results) D ['18544626', '15318244'], autosomal genes (388 results) S ['21483837'], heterozygous males (127 results) S ['21483837']
JE	TRUE	5	TRUE	high-elevation (398 results) S ['21483836'], roles of meteorologic (31 results) S ['18784755'], high-elevation pelagic (1 results) S ['18784755'], meteorologic variability (16 results) S ['18784755'], Can phosphorus limitation inhibit (6 results) D ['12037613'], limitation of planktonic (57 results) S ['21483836'], bacterioplankton (548 results) S ['21483836'], dark dilution culture (23 results) S ['21483836'], Can Phosphorus Limitation (548 results) S ['21483836'], dark dilution (2 results) S ['21483836'], phosphorus limitation of planktonic (9 results) D ['19719561'], phosphorus limitation of planktonic (9 results) S ['21483836'], Phosphorus Limitation Inhibit (6 results) D ['12037613'], nutrient enrichment (271 results) S ['21483836'], planktonic heterotrophic bacteria (5 results) D ['19719561'], Phenology of high-elevation (5 results) S ['18784755'], catchment inputs (7 results) S ['18784755'], bacterial community (2341 results) S ['21483836'], transparent microcosms (2 results) S ['21483836'], bacterioplankton growth (17 results) D ['16691326', '15965720'], bacterioplankton growth (17 results) S ['21483836'], thermal stratification (64 results) S ['18784755']
JE	TRUE	4	TRUE	Band oligomeric (221 results) D ['19389033'], pre-storage gamma (4 results) D ['16456250'], Prestorage leukocyte filtration (6 results) D ['10208393'], static pressure during shear-rate (7 results) D ['11872008', '11482485'], Prestorage (251 results) D ['20441510'], Prestorage (251 results) S ['21483835'], storage time (2344 results) S ['21483835'], storage variables of RBCs (32 results) D ['18798805'], Influence of prestorage (23 results) D ['18798805'], leucocyte filtration (29 results) S ['21483835'], pre-storage gamma irradiation (2 results) D ['16456250'], bioactive substance accumulation (5 results) D ['10208393']

PMC3069077	5	FALSE	12	FALSE		7	15	TRUE	10	TRUE	9	TRUE	8	TRU
PMC3069076	11	FALSE	11	FALSE		7	13	FALSE	13	TRUE	12	TRUE	12	TRU
PMC3069071	10	FALSE	5	FALSE		6	12	TRUE	10	TRUE	8	TRUE	6	TRU
PMC3069070	7	FALSE	6	FALSE		9	13	TRUE	12	TRUE	11	TRUE	10	TRU

JE	TRUE	5	TRUE	leptospiral strains (45 results) D ['12890019'], serovar (7375 results) S ['21483834'], leptospiral burden (2 results) D ['16428731', '15668855'], Alveolar septal deposition (2 results) S ['14982864'], complement parallels pulmonary hemorrhage (3 results) S ['14982864'], leptospirosis (6634 results) S ['21483834'], TLR recognition of leptospiral (2 results) D ['16237097'], recognition of leptospiral (32 results) D ['16237097'], leptospires (925 results) S ['21483834'], septal deposition (5 results) S ['14982864'], leptospiral (1353 results) S ['21483834'], exposure of leptospires (54 results) D ['19345023'], Leptospira (5327 results) S ['21483834'], parallels pulmonary hemorrhage (4 results) S ['14982864'], complement parallels pulmonary (7 results) S ['14982864'], septal deposition of immunoglobulin (20 results) S ['14982864'], non-virulent leptospires (1 results) S ['21483834']
JE	TRUE	10	TRUE	COMMD1 forms oligomeric (1 results) D ['18940794'], CFTR ubiquitination (6 results) S ['21483833'], Commd1 null mice (1 results) D ['17371845'], Chem COMMD1 (1 results) D ['20237237'], functional homologs of MURR1 (1 results) D ['15799966'], COMMD1 (66 results) D ['20433422', '20237237', '18940794'], COMMD1 (66 results) S ['21483833'], conductance regulator (6612 results) S ['21483833'], homologs of MURR1 (1 results) D ['15799966'], Commd1 null (1 results) D ['17371845'], transmembrane conductance regulator (6585 results) S ['21483833'], CFTR (6577 results) S ['21483833'], sodium channel through Nedd4-2 (112 results) D ['20237237'], COMMD1 forms (2 results) D ['18940794', '14645214'], COMMD1 forms oligomeric complexes (1 results) D ['18940794'], fibrosis transmembrane conductance (6477 results) S ['21483833'], fibrosis transmembrane (6956 results) S ['21483833'], COMMD1 downregulates (1 results) D ['20237237'], transmembrane conductance (6603 results) S ['21483833'], cystic fibrosis transmembrane conductance (6477 results) S ['21483833'], fibrosis transmembrane conductance regulator (6469 results) S ['21483833'], cystic fibrosis transmembrane (6619 results) S ['21483833']
JE	TRUE	5	TRUE	Bax-deficient carcinoma (7 results) D ['20308427', '11875499'], Endogenous Bak inhibitors (19 results) D ['20308427'], cholangiocarcinoma cells (189 results) S ['21483830'], XIAP bypasses (1 results) D ['18829553'], Endogenous Bak (6 results) D ['20308427'], differential impact on TRAIL (12 results) D ['20308427'], XIAP (1785 results) S ['21483830'], XIAP bypasses resistance (1 results) D ['18829553'], Bak inhibitors Mcl-1 (108 results) D ['20308427'], Endogenous Bak inhibitors Mcl-1 (2 results) D ['20308427'], Cytotoxicity-dependent APO-1 proteins (1 results) D ['8521815'], cholangiocarcinoma (5896 results) S ['21483830'], Mcl-1 mediates tumor necrosis (4 results) S ['15150106'], cooperates with TRAIL (28 results) D ['18829553'], TRAIL resistance (234 results) S ['21483830']
JE	TRUE	7	TRUE	trafficking-defective (22 results) D ['18096051', '16610352', '16361248', '15760896', '15072950'], antibiotics restore functional (77 results) D ['19324319'], hERG mutant channels (173 results) D ['19940115'], hERG mutant channels (173 results) S ['21483829'], Aminoglycoside antibiotics restore functional (27 results) D ['19324319'], HERG (1588 results) S ['21483829'], antibiotics restore functional expression (29 results) D ['19324319'], LQT2 (278 results) S ['21483829'], mutant channels (566 results) S ['21483829'], Most LQT2 mutations (176 results) S ['21483829'], Most LQT2 (278 results) S ['21483829'], pharmacological rescue of trafficking-defective (8 results) D ['16610352', '16361248', '15072950', '12837749'], rescue of trafficking-defective (10 results) D ['16610352', '16361248', '15072950', '12837749']

PMC3069068	10	FALSE	11	FALSE		12	16	TRUE	13	TRUE	12	TRUE	8	TRU
PMC3069067	4	FALSE	17	FALSE		11	19	TRUE	17	TRUE	17	TRUE	14	TRU
PMC3069066	0	TRUE	14	FALSE		2	14	FALSE	14	TRUE	13	TRUE	11	TRU
PMC3069063	17	FALSE	3	FALSE		19	18	TRUE	11	TRUE	11	TRUE	10	TRU

JE	TRUE	7	TRUE	interethnic differences (201 results) S ['21483827', '19929378'], interferon-gamma response against Plasmodium (146 results) S ['19929378'], BDCA3-positive (1 results) D ['17000725'], frequency of BDCA3-positive (1 results) D ['17000725'], sympatric Fulani (25 results) D ['18174328'], sympatric Fulani (25 results) S ['21483827', '19929378', '18831954', '18793404', '17662633', '16298405', '15853923', '15772314', '15716067'], interferon-gamma response (117 results) S ['19929378'], African Common TLR-4 variants (3 results) D ['16371473'], falciparum infection (1151 results) S ['21483827'], sympatric ethnic groups (18 results) D ['18174328'], sympatric ethnic groups (18 results) S ['21483827', '18831954', '18793404', '15772314'], Fulani (221 results) S ['21483827'], African Common TLR-4 (3 results) D ['16371473'], circulation of Kenyan (13 results) D ['17000725'], BDCA3-positive dendritic cells (1 results) D ['17000725'], Plasmodium falciparum correlates (152 results) S ['19929378'], sympatric (2698 results) S ['21483827'], correlates with interethnic (7 results) S ['19929378'], falciparum correlates with interethnic (1 results) S ['19929378'], peripheral circulation of Kenyan (4 results) D ['17000725'], BDCA3-positive dendritic (1 results) D ['17000725']
JE	TRUE	7	TRUE	vivo homeostatic synaptic (60 results) S ['21483826', '20505084'], GluR1-S845 phosphorylation (4 results) S ['21483826', '19892736'], RNA granules during homeostatic (4 results) D ['18840692'], visual cortex (23244 results) S ['21483826'], S845 site (19 results) D ['12843287'], S845 site (19 results) S ['21483826', '19906877', '19892736'], Cross-modal regulation of synaptic (1 results) S ['16819524'], subunit GluR1 phosphorylation (156 results) S ['21483826'], homeostatic synaptic plasticity (43 results) S ['21483826', '20505084'], GluR1 phosphorylation sites (3 results) S ['17923095', '12628184', '10879537'], subunit GluR1 phosphorylation sites (40 results) D ['16272153'], subunit GluR1 phosphorylation sites (40 results) S ['21483826', '19906877', '19892736'], GluR1 serine phosphorylation (83 results) S ['21483826', '19906877', '19892736'], experience-induced (129 results) S ['20505084'], Stabilization of AMPA (90 results) S ['19892736'], GluR1 (1547 results) S ['21483826'], receptor subunit GluR1 phosphorylation (152 results) S ['19906877'], homeostatic synaptic (620 results) S ['21483826'], granules during homeostatic (33 results) D ['18840692'], superficial layers of mouse (407 results) S ['20505084'], Cross-modal regulation (14 results) S ['16819524']
JE	TRUE	8	TRUE	marker of physiologic (6001 results) S ['21483825'], cortisol variability across women (28 results) S ['21483825'], morning urinary (2484 results) S ['21483825'], cortisol secretion (2528 results) S ['21483825'], physiologic stress levels (6 results) S ['21483825'], Mayan population (17 results) S ['15368600'], morning urinary cortisol levels (211 results) S ['21483825'], basal cortisol (745 results) S ['21483825'], rural Mayan population (36 results) S ['15368600'], cortisol levels (8136 results) S ['21483825'], physiologic stress (381 results) S ['21483825'], rural Mayan (43 results) S ['15368600'], menstrual cycle (19702 results) S ['21483825'], morning urinary cortisol (12 results) S ['21483825']
JE	TRUE	3	TRUE	Zhengdong Zhang (98 results) S ['21483822'], rs11614913 polymorphism (3 results) D ['20640596', '20229273'], rs11614913 polymorphism (3 results) S ['21483822'], microRNA-196a2 (5 results) D ['21080878', '20466450', '19293314'], rs11614913 (24 results) D ['21080878', '21073609', '20842445', '20722507', '20640596', '20549817', '20520619', '20501619', '20229273', '20188135', '19847796', '19834808', '19567675'], rs11614913 (24 results) S ['21483822'], miR-499 (35 results) D ['21073609', '20549817'], miR-221 overexpression contributes (2 results) D ['20018759'], mature miR-125a alters (1 results) D ['17400653'], susceptibility loci for esophageal (31 results) D ['19138993'], Italian familial breast cancer (131 results) D ['19847796'], Mature microRNA sequence polymorphism (31 results) D ['20501619', '19567675'], pre-microRNA genes (5 results) D ['20549817'], Avissar-Whiting (4 results) D ['20501619'], Italian familial breast (194 results) D ['19847796'], miR-125a alters (2 results) D ['17400653'], miR196a2 (4 results) D ['20842445', '20501619', '20188135', '19847796'], loci for esophageal (174 results) D ['19138993'], familial breast cancer cases (66 results) D ['19847796'], low-penetrance alleles (40 results) D ['19847796']

PMC3069060	3	FALSE	19	FALSE		9	16	TRUE	15	TRUE	13	TRUE	13	TRU
PMC3069057	6	FALSE	5	FALSE		9	11	FALSE	11	FALSE	11	TRUE	9	TRU
PMC3069056	6	FALSE	8	FALSE		8	14	TRUE	10	TRUE	8	TRUE	8	TRU
PMC3069055	1	FALSE	18	FALSE		5	14	TRUE	10	TRUE	9	TRUE	8	TRU
PMC3069054	10	FALSE	4	FALSE		6	13	FALSE	13	TRUE	12	TRUE	8	TRU

JE	TRUE	7	TRUE	experimental Lyme HgCl2 treatment (1 results) S ['17672870'], erythema migrans (867 results) S ['21483819'], Austria with Lyme (138 results) D ['19571749'], HgCl2 treatment decreases (29 results) S ['17672870'], type 1-like responses (81 results) S ['17672870'], experimental Lyme HgCl2 (1 results) S ['17672870'], Borrelia-specific interferon-gamma cells (10 results) S ['16176932', '15122525', '10193424', '9394788'], Lyme HgCl2 (1 results) S ['17672870'], Ekerfelt (62 results) S ['21483819', '17672870', '16176932'], Lyme HgCl2 treatment (1 results) S ['17672870'], Borrelia-specific interferon-gamma (11 results) S ['16176932', '15122525', '10193424', '9394788'], Lyme borreliosis (2001 results) S ['21483819'], patients with erythema (9279 results) S ['21483819'], Borrelia-specific (74 results) S ['19793326'], non-viral prospective cohort study (26 results) D ['16950834'], non-viral prospective cohort (2 results) D ['16950834'], Borrelia (8639 results) S ['21483819'], delays eradication (31 results) S ['17672870'], burgdorferi (7093 results) S ['21483819'], Lyme HgCl2 treatment decreases (1 results) S ['17672870'], delays eradication of Borrelia (1 results) S ['17672870'], borreliosis (2599 results) S ['21483819']
JE	TRUE	4	TRUE	H274Y oseltamivir-resistant influenza (37 results) D ['20100088', '19962344'], oseltamivir-resistant influenza viruses (12 results) D ['19331731', '19193257'], oseltamivir-resistant influenza (70 results) D ['20665943'], oseltamivir-resistant influenza (70 results) S ['21483816'], H274Y oseltamivir-resistant influenza viruses (36 results) D ['20100088', '19962344'], reassortment (1076 results) S ['21483816'], H274Y oseltamivir-resistant (37 results) D ['20100088', '19962344'], oseltamivir-resistant (198 results) S ['21483816'], Emergence of H274Y (20 results) D ['19962344', '19501261', '19255111', '12103431'], reassortment human influenza viruses (369 results) S ['21483816'], oseltamivir (1802 results) S ['21483816']
JE	TRUE	5	TRUE	SHIV strains with tier (2 results) S ['20657739'], robust one-tube real-time reverse (3 results) S ['10957722'], strains with tier (49 results) S ['20657739'], mucosal SHIV (125 results) S ['21483815'], SHIV (789 results) S ['21483815'], simian-human (504 results) S ['20657739'], simian-human immunodeficiency (493 results) S ['20657739'], macaques after coimmunization (2 results) D ['18000037'], SHIV challenge (59 results) D ['19525965', '19436712'], SHIV challenge (59 results) S ['21483815'], recombinant glycoprotein HIV-1 vaccine (386 results) D ['20577269'], coimmunization with SHIV (2 results) D ['18000037'], mucosal SHIV challenge (8 results) D ['19525965', '19436712'], bivalent recombinant glycoprotein HIV-1 (6 results) D ['17109337']
JE	TRUE	5	TRUE	PDE9Q453E (1 results) S ['21483814'], Glu406 (1 results) S ['21483814'], phosphodiesterase (28869 results) S ['21483814'], phosphodiesterase-9A selective (1 results) S ['20121115'], phosphodiesterase shows orientation variation (3 results) S ['15210993'], cGMP (16914 results) S ['21483814'], Crystal structures of phosphodiesterases (94 results) S ['20121115'], Gln453 (2 results) S ['21483814'], phosphodiesterase-9A (6 results) S ['20121115'], Crystal structure of phosphodiesterase (253 results) S ['21483814'], structure of phosphodiesterase (4178 results) S ['21483814'], phosphodiesterase shows (54097 results) S ['21483814'], PDE9 inhibitors (3 results) S ['21483814', '20121115'], phosphodiesterase-9A selective inhibitors (1 results) S ['20121115'], PDE9Q453E mutant (1 results) S ['21483814'], Glu453 (5 results) S ['21483814'], orientation variation of inhibitor (13 results) S ['15210993'], PDE9 (34 results) D ['18757755', '18674549'], PDE9 (34 results) S ['21483814', '20121115']
JE	TRUE	4	TRUE	prognostic marker for ESCC (100 results) S ['21483813'], induces autoantibody overproduction (9 results) D ['12875719'], esophageal squamous cell (2593 results) S ['21483813'], clinical relevance of Th17 (15 results) D ['19470694'], esophageal squamous (11770 results) S ['21483813'], IL-17 induces autoantibody (13 results) D ['12875719'], NPC progression (16 results) D ['20064222'], intratumoral cells correlate (101 results) D ['19329213'], lymphocytes correlate with NPC (11 results) D ['20064222'], spontaneous ocular inflammation (80 results) D ['19151391'], esophageal squamous cell carcinoma (2222 results) S ['21483813'], tumor environments (27 results) D ['19470694'], IL-17 induces autoantibody overproduction (1 results) D ['12875719'], correlate with NPC (81 results) D ['20064222']

PMC3069053	4	FALSE	17	FALSE		6	17	TRUE	14	TRUE	14	TRUE	11	TRU
PMC3069051	0	TRUE	1	FALSE		1	1	FALSE	1	FALSE	1	FALSE	1	FAL
PMC3069050	2	FALSE	5	FALSE		4	7	FALSE	7	FALSE	7	TRUE	6	TRU
PMC3069049	2	FALSE	14	FALSE		4	16	FALSE	16	FALSE	16	FALSE	16	TRU
PMC3069048	1	FALSE	18	FALSE		4	9	TRUE	3	TRUE	3	TRUE	3	TRU
PMC3069047	8	FALSE	3	FALSE		5	11	TRUE	9	TRUE	7	TRUE	5	TRU

JE	TRUE	6	TRUE	successful molecular approach (2115 results) S ['21483872'], biodiversity assessment (47 results) D ['20525585', '20003263'], assemblages of chafers (1 results) D ['17420144'], nuclear rDNA expansion (77 results) S ['20836845'], composition of staphylinid (2 results) S ['21483872'], rDNA expansion (310 results) S ['20836845'], barcoding (484 results) S ['21483872'], multi-species assemblages of chafers (1 results) D ['17420144'], D3-marker (1 results) S ['21483872'], morphospecies (255 results) S ['21483872'], rDNA expansion segments (51 results) S ['20836845'], Central European ground beetles (2 results) S ['20836845'], Central European ground (36 results) S ['20836845'], species composition of staphylinid (2 results) S ['21483872'], richness number of morphospecies (11 results) S ['18717696'], ants of Mauritius (1 results) D ['20003263'], biodiversity patterns (66 results) S ['21483872'], Front Zool (160 results) S ['20836845'], changes biodiversity patterns (340 results) S ['21483872'], wide gradient of disturbance (14 results) S ['18717696'], nuclear rDNA expansion segments (15 results) S ['20836845']
.SE	TRUE	1	TRUE	gp41 (3528 results) S ['21483871']
JE	TRUE	4	TRUE	1beta overexpression inhibits (107 results) S ['20404331'], muscle protein synthesis (1038 results) S ['21483870'], skeletal muscle of hypercatabolic (41 results) D ['12107253'], muscle protein (3598 results) S ['21483870'], muscle translation initiation (952 results) S ['21483870'], 1beta overexpression inhibits muscle (15 results) S ['20404331'], muscle transcriptome (33 results) D ['18997871']
JE	TRUE	12	TRUE	different multiple alignment problems (44 results) S ['21483869'], sequence alignment (72588 results) S ['21483869'], multiple alignments (607 results) S ['21483869'], many different alignment problems (61 results) D ['18218900'], many different alignment problems (61 results) S ['21483869'], multiple sequence alignment (1222 results) S ['21483869'], sequences on MSA (247 results) D ['20455266'], sequences on MSA (247 results) S ['21483869'], new alignment benchmark (179 results) S ['21483869', '20639539'], protein sequences (6293 results) S ['21483869'], benchmark test (82 results) S ['21483869'], multiple sequence (2244 results) S ['21483869'], alignments of protein (4582 results) S ['21483869'], multiple alignments of protein (1643 results) S ['21483869'], alignment accuracy (228 results) S ['21483869'], Review Multiple alignment (448 results) S ['20639539']
JE	TRUE	2	TRUE	novel Lecudina phyllochaetopteri (1 results) S ['20008106'], Heliospora caprellae (1 results) S ['21483868'], Difficilina (2 results) S ['20008106'], Difficilina tubulani (1 results) S ['20008106'], gregarines (88 results) S ['21483868', '20008106', '18226585'], novel Lecudina (3 results) S ['20008106'], Cephaloidophora (3 results) S ['21483868'], gregarines from North-eastern (2 results) S ['20008106'], North-eastern Pacific polychaetes (1 results) S ['20008106'], Thiriotia (1 results) S ['21483868'], marine gregarines from North-eastern (1 results) S ['20008106'], Thiriotia pugettiae (1 results) S ['21483868'], communis from Balanus (4 results) S ['21483868'], pugettiae (1 results) S ['21483868'], microbial eukaryotes (106 results) D ['20007768'], Lecudina phyllochaetopteri (1 results) S ['20008106'], phyllochaetopteri (1 results) S ['20008106'], Heliospora (2 results) S ['21483868'], crustaceans (2557 results) S ['21483868']
JE	TRUE	3	TRUE	virus type strain Jamaican (6 results) D ['16847122'], cDNA clones (11066 results) S ['21483867'], Infectious RNA transcripts (44 results) D ['9188607'], infectious cDNA (422 results) S ['21483867'], type strain Jamaican (6 results) D ['16847122'], type-2 RNA genome Guinea (11 results) D ['7557426'], full-length dengue virus (3 results) D ['9188607'], Ribosome-messenger mRNA (12 results) D ['2011495'], full-length dengue virus type (67 results) D ['16847122'], Ribosome-messenger mRNA target (2 results) D ['2011495'], dengue virus (4742 results) S ['21483867']

PMC3069046	16	FALSE	1	FALSE		11	16	TRUE	8	TRUE	7	TRUE	4	TRU
PMC3069045	11	FALSE	8	FALSE		8	19	TRUE	15	TRUE	12	TRUE	11	TRU
PMC3069043	6	FALSE	13	FALSE		6	15	TRUE	11	TRUE	11	TRUE	11	TRU
PMC3069042	6	FALSE	2	FALSE		4	7	TRUE	4	TRUE	4	TRUE	3	TRU
PMC3069041	12	FALSE	4	FALSE		9	11	TRUE	9	TRUE	8	TRUE	6	TRU

JE	TRUE	1	TRUE	p32 (1921 results) S ['21483866'], increases DP5 (6 results) D ['16524368'], house ceramide (16 results) D ['17161984'], protein Hrk (4 results) D ['15031724'], bioactive lipid lessons (2 results) D ['18216770'], peptide increases DP5 expression (3 results) D ['16524368'], beta peptide increases DP5 (3 results) D ['16524368'], increases DP5 expression (3 results) D ['16524368'], peptide increases DP5 (4 results) D ['16524368'], keratoconus corneal fibroblasts (3 results) D ['18515575'], pore independent of Bax (60 results) D ['19217411'], DP5 expression via activation (2 results) D ['16524368'], lipid lessons from sphingolipids (15 results) D ['18216770'], Review Principles of bioactive (76 results) D ['18216770'], Nox-4 mediates endoplasmic reticulum (1 results) D ['15572675'], DP5 expression (28 results) D ['19629134', '17428807', '16524368', '15031724'], Homocysteine activates oxidase (12 results) D ['15496169']
JE	TRUE	8	TRUE	Plasmodium cynomolgi (301 results) S ['19015340'], hepatic forms (60 results) S ['21483865', '19876040'], vivax (6302 results) S ['21483865'], Plasmodium vivax malaria (482 results) D ['21151554'], liver stages of Plasmodium (418 results) D ['21151554'], cynomolgi (398 results) S ['21483865', '19015340'], exoerythrocytic parasites (21 results) D ['3006528', '2984950'], late post-sporozoite (5 results) D ['3250556'], late post-sporozoite tissue (5 results) D ['3250556'], post-sporozoite tissue (5 results) D ['3250556'], hypnozoites (68 results) D ['21151554'], hypnozoites (68 results) S ['21483865'], cynomolgi hepatic (9 results) D ['2929849'], cynomolgi hepatic (9 results) S ['21483865'], stages of Plasmodium (2980 results) S ['21483865'], late post-sporozoite tissue stages (5 results) D ['3250556'], post-sporozoite tissue stages (6 results) D ['3250556'], primaquine (1864 results) D ['21151554'], primaquine (1864 results) S ['21483865']
JE	TRUE	6	TRUE	orientation of counter-stereotypical (1 results) S ['21483863'], processes understanding disclosure (93 results) D ['20192562'], postdisclosure outcomes among people (1 results) D ['20192562'], Sexual orientation perception (3733 results) S ['21483863'], disclosure processes understanding (93 results) D ['20192562'], postdisclosure outcomes (4 results) D ['20192562'], orthogonal social category (4 results) S ['21483863'], counter-stereotypical groups (1 results) S ['21483863'], orientation perception (74 results) S ['20682754'], facial cues (104 results) S ['20682754'], sexual orientation of counter-stereotypical (1 results) S ['21483863'], judgments of sexual (472 results) S ['21483863'], Sexual orientation perception involves (42 results) S ['20682754'], role of masculinity-femininity (43 results) D ['18810629'], Swagger (3 results) S ['17723051', '16262776'], Accuracy of judgments (1754 results) S ['21483863'], gendered (866 results) S ['21483863'], orientation perception involves (286 results) S ['20682754'], gaydar accuracy (2 results) D ['18810629']
JE	TRUE	2	TRUE	complex class I-bound determinant (2 results) D ['8258346'], I-bound determinant (4 results) D ['8258346'], alpha-galactosylceramide (774 results) S ['21483862'], vivo requires CD40 ligation (14 results) D ['15197224'], Lang-DTREGFP (1 results) D ['18086865'], NKT cells (1898 results) S ['21483862'], class I-bound determinant (2 results) D ['8258346'], requires CD40 ligation (55 results) D ['15197224']
JE	TRUE	0	FALSE	Orivel (27 results) S ['18850082'], Irlbachia alata (2 results) D ['10395496'], extrafloral nectar (50 results) D ['19769117'], ant-plant Acacia constricta (1 results) D ['16450177'], indirect extrafloral nectar (24 results) D ['18493790', '18086230'], alatus individuals (1 results) S ['21483861'], ant-plant Acacia (11 results) D ['16450177'], dried-out (81 results) S ['21483861'], phenotype redux (6 results) D ['19255576'], ant-plant Acacia constricta traits (1 results) D ['16450177'], Acacia constricta traits (1 results) D ['16450177'], ant-plant (77 results) D ['21203550'], Irlbachia (2 results) D ['10395496'], alatus (63 results) S ['21483861'], optimal defence hypothesis (14 results) D ['18493790'], constricta traits (1 results) D ['16450177']

PMC3069040	7	FALSE	11	FALSE		9	14	FALSE	14	TRUE	11	TRUE	4	TRU
PMC3069039	0	TRUE	20	FALSE		3	19	TRUE	15	TRUE	14	TRUE	12	TRU
PMC3069036	11	FALSE	4	FALSE		6	15	TRUE	9	TRUE	8	TRUE	7	TRU
PMC3069035	5	FALSE	3	FALSE		5	8	TRUE	7	TRUE	6	TRUE	5	TRU
PMC3069033	14	FALSE	5	FALSE		7	13	FALSE	13	FALSE	13	TRUE	12	TRU

JE	TRUE	2	TRUE	CXXXCXXC (15 results) D ['19501188', '18307109'], CXXXCXXC (15 results) S ['21483860'], bona fide ceramide (12 results) D ['18547394'], bona fide ceramide (12 results) S ['21483860'], OsCERK (1 results) S ['21483860'], fide ceramide (12 results) D ['18547394'], fide ceramide (12 results) S ['21483860'], ceramide kinase (104 results) S ['21483860'], bona fide ceramide kinase (1 results) S ['21483860'], CERK (57 results) D ['19501188'], CERK (57 results) S ['21483860'], novel lipid kinase (8 results) D ['11956206'], acd5 (3 results) S ['21483860', '14563678', '10978297'], CERK activity (12 results) D ['18662741', '16170208'], fide ceramide kinase (1 results) S ['21483860'], CXXXCXXC motif (7 results) D ['19501188'], CXXXCXXC motif (7 results) S ['21483860'], ceramides (5476 results) S ['21483860']
JE	TRUE	9	TRUE	Kalimantan (175 results) S ['21483859'], orangutan (741 results) S ['21483859'], underlie conservation (181 results) S ['21483859'], census techniques (5 results) S ['18372567'], underlie threats (23 results) S ['21483859'], underlie conservation threats (3 results) S ['21483859'], sensitivity of resultant (1675 results) S ['21483859'], Ancrenaz (26 results) S ['21483859', '15630475'], nest decay implications (1 results) S ['18372567'], relative encounter rates (201 results) S ['21483859'], orangutan presence (145 results) S ['21483859'], Meijaard (21 results) S ['21483859', '18372567'], orangutan census (4 results) S ['18372567'], decay implications for population (50 results) S ['18372567'], relative encounter (2536 results) S ['21483859'], interview surveys (437 results) S ['21483859'], Conservation research aims (440 results) S ['21483859'], orangutan census techniques (3 results) S ['18372567'], nest decay (14 results) S ['18372567'], Kalimantan range (8 results) S ['18372567']
JE	TRUE	3	TRUE	Rossion (110 results) D ['20051235'], variance account (4 results) D ['18055223'], Does physical interstimulus variance (36 results) D ['18055223'], Faces Sustain Neural Activity (3 results) S ['21483856'], Review Does physical interstimulus (2 results) D ['18055223'], physical interstimulus variance (36 results) D ['18055223'], physical interstimulus variance account (3 results) D ['18055223'], N170 (424 results) S ['21483856'], rapid ERP responses (2 results) D ['19330049'], interstimulus variance account (11 results) D ['18055223'], early face representations (85 results) D ['20051235'], animacy (135 results) S ['21097720'], Faces Sustain Neural (10 results) S ['21483856'], Ten lessons (27 results) D ['18055223'], reference electrode site (4 results) D ['16214404']
JE	TRUE	3	TRUE	adipogenesis (3381 results) S ['21483855'], preadipocyte (1215 results) S ['21483855'], Macrophages block insulin action (3 results) D ['16926380'], Macrophages block insulin (29 results) D ['16926380'], abdominal role of Wnt (16 results) D ['19351711'], delta-like variants (7 results) D ['10234807'], macrophages aggravates inflammatory role (27 results) D ['16123319'], preadipocytes (2412 results) S ['21483855']
JE	TRUE	6	TRUE	cells during infliximab (649 results) S ['21483853'], AutoMACS-separated (1 results) S ['19958773'], Review Hepatosplenic T-cell (62 results) D ['20371449'], infliximab treatment of Crohn's (1603 results) S ['21483853'], young man with Crohn's (81 results) D ['20371449'], T-cell lymphoma including gammadelta (22 results) D ['18955564'], cell receptor delta repertoire (1 results) D ['12016128'], lymphoma including gammadelta (22 results) D ['18955564'], Review Hepatosplenic (180 results) D ['20371449'], Hepatosplenic T-gammadelta (1 results) D ['12688327'], infliximab (6540 results) S ['21483853'], T-gammadelta lymphoma (1 results) D ['12688327'], T-cell lymphoma (11387 results) S ['21483853'], Hepatosplenic T-cell (113 results) D ['20371449', '19749141'], receptor delta repertoire (1 results) D ['12016128'], T-gammadelta (18 results) D ['12688327'], Hepatosplenic T-cell lymphoma (99 results) D ['20371449', '19749141'], Hepatosplenic T-gammadelta lymphoma (1 results) D ['12688327'], Review Hepatosplenic T-cell lymphoma (58 results) D ['20371449']

PMC3069032	9	FALSE	10	FALSE		15	14	TRUE	11	TRUE	10	TRUE	8	TRU
PMC3069031	16	FALSE	5	FALSE		6	13	TRUE	10	TRUE	10	TRUE	10	TRU
PMC3069028	1	FALSE	18	FALSE		7	19	TRUE	14	TRUE	12	TRUE	11	TRU
PMC3069027	10	FALSE	6	FALSE		5	14	TRUE	13	TRUE	9	TRUE	8	TRU

JE	TRUE	4	TRUE	new hybrid antimicrobial antibiotic (293 results) D ['20189105'], polyketide antibiotic (106 results) D ['20189105'], polyketide antibiotic (106 results) S ['20190824'], precedes pseudomonic (1 results) S ['17383964'], holomycin (15 results) D ['21041678', '11158751'], holomycin (15 results) S ['21483852'], antibiotic holomycin (2 results) D ['21041678'], pseudomonic acid (75 results) D ['12672810'], pseudomonic acid (75 results) S ['21483852', '17383964', '16039529', '15726184', '12770824'], biosynthesis precedes pseudomonic (1 results) S ['17383964'], NCIMB10586 (2 results) S ['16039529', '15583005'], precedes pseudomonic acid (3 results) S ['17383964'], dithiolopyrrolone antibiotic holomycin (1 results) D ['21041678'], biosynthesis precedes pseudomonic acid (1 results) S ['17383964'], thiomarinol (7 results) D ['9207918', '7592043', '8071134', '8294241'], mupirocin (1176 results) S ['21483852'], pseudomonic (79 results) D ['12672810'], pseudomonic (79 results) S ['21483852', '17383964', '16039529', '15726184', '12770824'], dithiolopyrrolone antibiotic (7 results) D ['21041678']
JE	TRUE	4	TRUE	falciparum competent strains (3 results) D ['19596869', '18493601'], drug efficacy-resistance profile (1 results) D ['19815871'], antimalarial drug efficacy-resistance (1 results) D ['19815871'], Plasmodium falciparum competent strains (3 results) D ['19596869', '18493601'], human erythrocytes (9377 results) S ['21483851'], C2A clone of Plasmodium (1 results) D ['19815871'], antimalarial drug efficacy-resistance profile (1 results) D ['19815871'], vivo selection of competent (86 results) D ['18493601'], non-myelodepleted (2 results) D ['19596869', '18493601'], murine model of falciparum-malaria (366 results) S ['21483851'], multidrug-resistant C2A (1 results) D ['19815871'], competent strains (55 results) D ['19596869', '18493601'], vivo antimalarial drug efficacy-resistance (1 results) D ['19815871'], model of falciparum-malaria (1314 results) S ['21483851'], falciparum competent (35 results) D ['19596869', '18493601'], Thai multidrug-resistant C2A (1 results) D ['19815871'], murine model of malaria (823 results) S ['21483851'], Plasmodium falciparum competent (30 results) D ['19596869', '18493601'], NOD-scid IL2Rgammanull mice (5 results) D ['19596869'], defense mechanisms against Plasmodium (35 results) D ['19837977'], defense mechanisms against Plasmodium (35 results) S ['17709281']
JE	TRUE	6	TRUE	pathogen Helicobacter pylori reveals (18 results) S ['18946507'], human pathogen Helicobacter (67 results) S ['21483848', '18946507'], Helicobacter pylori (29726 results) S ['21483848'], Helicobacter pylori HtrA (11 results) S ['21483848', '20814423', '18946507'], human pathogen Helicobacter pylori (66 results) S ['21483848', '18946507'], fuzzy pharmacophores (4 results) S ['17896338'], pylori (31150 results) S ['21483848'], pylori HtrA (11 results) S ['21483848', '20814423', '18946507'], pseudo-ligand approach (3 results) S ['16787149'], cleaves E-cadherin (24 results) S ['20814423'], protein cavities (69 results) D ['18570371'], protein cavities (69 results) S ['19142949'], pathogen Helicobacter pylori (340 results) S ['21483848'], pylori reveals proteolytic (3 results) S ['18946507'], pylori reveals proteolytic activity (2 results) S ['18946507'], HtrA (378 results) S ['21483848'], pathogen Helicobacter (359 results) S ['21483848'], Helicobacter pylori reveals proteolytic (3 results) S ['18946507'], receptor-based (771 results) S ['21483848']
JE	TRUE	2	TRUE	temozolomide versus radiotherapy (43 results) D ['19269895'], risky miRNAs (3 results) S ['21483847'], EORTC-NCIC trial (7 results) D ['19269895'], miR-31 ablates expression (1 results) D ['20145132'], GBM (4688 results) S ['21483847'], microRNA survival (861 results) S ['21483847'], HIF regulatory factor FIH (10 results) D ['20145132'], regulatory factor FIH (10 results) D ['20145132'], microRNA survival signature (59 results) S ['21483847'], GBM patient (20 results) S ['21483847'], miR-31 ablates (1 results) D ['20145132'], miR-200 expression regulates (9 results) D ['19671845'], adjuvant temozolomide versus radiotherapy (22 results) D ['19269895'], protective miRNAs (49 results) S ['21483847'], expression of miR-106a (38 results) D ['20219352'], adjuvant temozolomide versus (28 results) D ['19269895']

PMC3069026	3	FALSE	5	FALSE		11	8	TRUE	7	TRUE	7	TRUE	6	TRU
PMC3069025	1	FALSE	9	FALSE		3	10	FALSE	10	FALSE	10	FALSE	10	TRU
PMC3069004	2	FALSE	13	FALSE		4	13	TRUE	11	TRUE	9	TRUE	8	TRU
PMC3069003	5	FALSE	7	FALSE		7	12	TRUE	10	TRUE	8	TRUE	6	TRU
PMC3069001	0	TRUE	3	FALSE		1	3	FALSE	3	FALSE	3	FALSE	3	FAL
PMC3069000	4	FALSE	3	FALSE		4	7	FALSE	7	TRUE	5	TRUE	5	TRU
PMC3068999	5	FALSE	15	FALSE		8	18	TRUE	14	TRUE	14	TRUE	12	TRU

JE	TRUE	5	TRUE	specific previous conditions (4695 results) S ['21483846'], previous lung diseases (12 results) D ['19812684', '19169896', '11139328', '11075875', '8785672', '7771438', '1442729', '2257230'], previous lung diseases (12 results) S ['21483846'], nonmalignant respiratory evidence (34 results) D ['20044861', '16733074'], previous lung (118 results) D ['19812684', '19169896'], previous lung (118 results) S ['21483846'], lung cancer risk (2106 results) S ['21483846'], smokers for pneumonia (454 results) S ['21483846']
JE	TRUE	7	TRUE	HFE (2425 results) S ['21483845'], C282Y (1226 results) S ['21483845'], Iron Overload (6077 results) S ['21483845'], C282Y mutation (540 results) S ['21483845'], German first-time blood donors (22 results) D ['14648375'], hemochromatosis (8013 results) S ['21483845'], HEIRS Study (34 results) S ['21483845'], serum ferritin (5766 results) S ['21483845'], serum iron (4200 results) S ['21483845'], Roche Hitachi analyzer (34 results) S ['17511979']
JE	TRUE	7	TRUE	multiple object (167 results) S ['21483844'], MOT with point-light (1 results) S ['21483844'], point-light (347 results) S ['21483844'], naturalistic aspects (3 results) S ['21483844'], Shape perception reduces activity (6 results) D ['12417754'], tracking of point-light (4 results) S ['21483844'], non-biological (572 results) S ['21483844'], naturalistic aspects of biological (14 results) S ['21483844'], high-level visual processes (9 results) D ['16604312'], influence of high-level (2380 results) S ['21483844'], walkers on MOT (1 results) S ['21483844'], biological motion (355 results) S ['21483844'], point-light walkers (31 results) S ['21483844', '18466408'], object of attention (4358 results) S ['21483844'], biological stimuli (108 results) S ['21483844']
JE	TRUE	3	TRUE	domestic pigeons (131 results) D ['18779923'], barnyard (97 results) S ['21483843'], H5N2 (301 results) S ['21483843'], H7N3 (108 results) S ['21483843'], H5N2 virus (40 results) S ['21483843'], H5N2 experiment (8 results) D ['7483266'], quasi-natural laboratory environment (2 results) S ['21483843'], barnyard experiments (8 results) S ['21483843'], Transmission of LPAIV (12 results) D ['16863079'], Minute excretion (5 results) D ['17947534'], quasi-natural laboratory (11 results) S ['21483843'], LPAIV (37 results) D ['20521685', '20408403']
.SE	TRUE	3	TRUE	Universal Hospital Admission (258 results) S ['21483492'], isolation per infection (7727 results) S ['21483492'], additional cost of isolation (458 results) S ['21483492']
JE	TRUE	1	TRUE	interruption persist (50 results) D ['16951642'], multidrug-resistant final results (35 results) D ['16951642'], treatment interruption persist (34 results) D ['16951642'], treatment interruption (814 results) S ['21483491'], CPCRA study (64 results) D ['16951642'], non-AIDS serious adverse events (7 results) S ['19430303'], OPTIMA Trial (7 results) S ['21483491', '12865041']
JE	TRUE	8	TRUE	Structure of B-MLV (3 results) S ['18222469'], Identification of postentry (11 results) D ['18799582'], leukemia virus (18931 results) S ['21483490'], murine leukemia (12139 results) S ['21483490'], TRIM5alpha (214 results) D ['20956011'], TRIM5alpha (214 results) S ['21483490'], TRIM5alpha domain (74 results) D ['19656869'], capsid amino-terminal domain (3 results) S ['19007792', '18222469'], B-MLV capsid amino-terminal (1 results) S ['18222469'], murine leukemia virus (12184 results) S ['21483490'], amino-terminal domain reveals key (18 results) S ['18222469'], capsid amino-terminal domain reveals (5 results) S ['18222469'], retrovirus restriction (29 results) D ['19656869', '15994791'], retrovirus restriction (29 results) S ['16912305'], B-MLV capsid amino-terminal domain (1 results) S ['18222469'], B-MLV capsid (5 results) S ['18222469'], capsid (22378 results) S ['21483490'], retrovirus (13450 results) S ['21483490'], B-box surface (21 results) D ['19656869'], capsid amino-terminal (337 results) S ['19007792', '18222469']

PMC3068998	10	FALSE	17	FALSE		15	27	TRUE	22	TRUE	17	TRUE	14	TRU
PMC3068997	9	FALSE	21	FALSE		11	21	TRUE	13	TRUE	12	TRUE	11	TRU
PMC3068996	3	FALSE	18	FALSE		7	9	FALSE	9	FALSE	9	TRUE	6	TRU

JE	TRUE	12	TRUE	expression of SV2C (9 results) D ['10625067'], expression of SV2C (9 results) S ['16543415'], uptake with botulinum (162 results) D ['20704566'], SV2C (20 results) D ['16545378', '15466855', '10625067', '10624962'], SV2C (20 results) S ['18815274', '16543415'], synaptic vesicle protein (519 results) S ['21483489'], bind gangliosides (620 results) D ['20507178'], bind gangliosides (620 results) S ['21483489', '20219474'], SV2C antibody (3 results) D ['10625067'], vesicle protein (857 results) S ['21483489'], hippocampal neurons (9078 results) S ['21483489'], domain of SV2C (5 results) D ['16545378', '15466855'], domain of SV2C (5 results) S ['18815274', '16543415'], domains of SV2 (18 results) D ['19476346', '1355409', '1519064', '1730776'], isoforms of SV2 (44 results) D ['19650874'], isoforms of SV2 (44 results) S ['21483489', '18815274', '16543415'], neurotoxins (26084 results) S ['21483489'], botulinum neurotoxin (1402 results) S ['21483489'], gangliosides (10180 results) S ['21483489'], SV2 (376 results) S ['21483489'], synaptic vesicle (3614 results) S ['21483489'], chimeric receptors (732 results) S ['21483489'], SV2 luminal (8 results) S ['18815274'], cytoplasmic domain of SV2C (3 results) D ['15466855'], cytoplasmic domain of SV2C (3 results) S ['18815274', '16543415'], SV2 isoforms (9 results) D ['10625067', '10624962'], SV2 isoforms (9 results) S ['21483489', '16543415']
JE	TRUE	4	TRUE	Lectin receptor (420 results) S ['21483488'], CW-PM adhesions (1 results) S ['21483488'], L-type lectin receptor (19 results) S ['19773388'], Arabidopsis L-type (6 results) S ['19773388'], Rpi-blb1 potato (6 results) S ['19888819', '18682852'], RanGAP2 mediates nucleocytoplasmic (1 results) D ['21169509'], Arabidopsis L-type lectin receptor (1 results) S ['19773388'], RanGAP2 mediates (3 results) D ['21169509'], infestans (1540 results) S ['21483488'], Phytophthora (1981 results) S ['21483488'], plasma membrane-cell wall (27 results) D ['11226194'], plasma membrane-cell wall (27 results) S ['16361528', '9807828'], Arabidopsis-Phytophthora resistance requires (1 results) D ['11722772'], Phytophthora protein (840 results) S ['21483488'], functional PAD2 gene (2 results) D ['11722772'], Lectin receptor kinases (4 results) S ['19773388', '16361528'], wall adhesions (33 results) S ['16361528'], plasma membrane-cell wall adhesions (2 results) S ['16361528'], virulent on Rpi-blb1 (1 results) S ['19888819'], plasma membrane-cell (40 results) D ['11226194'], plasma membrane-cell (40 results) S ['16361528'], lectin receptor phylogeny (85 results) S ['19773388'], Arabidopsis-Phytophthora resistance (2 results) D ['20569384', '11722772'], functional PAD2 (3 results) D ['11722772'], Lectin receptor kinases participate (29 results) S ['16361528'], 35S-ipiO1 (1 results) S ['21483488'], Arabidopsis L-type lectin (1 results) S ['19773388'], membrane-cell wall adhesions (3 results) S ['16361528'], pathogen Phytophthora infestans requires (5 results) D ['20687803'], L-type lectin receptor phylogeny (1 results) S ['19773388']
JE	TRUE	4	TRUE	avirulent bradyzoites (15 results) D ['9746587'], avirulent bradyzoites (15 results) S ['21483487'], nucleolus (14199 results) S ['21483487'], anti-TgNF3 (1 results) S ['21483487'], domain of TgNF3 (1 results) S ['21483487'], TgNF3 protein (1 results) S ['21483487'], expression of TgNF3 (1 results) S ['21483487'], gondii gene promoters (22 results) D ['21124925', '18433450', '17559302', '16287846'], gondii gene promoters (22 results) S ['21483487', '15784612'], stage-specifically (105 results) S ['21483487'], TgNF3 interacts (1 results) S ['21483487'], tachyzoites (1582 results) S ['21483487'], ectopic expression of TgNF3 (1 results) S ['21483487'], TgNF3 shares (1 results) S ['21483487'], N-terminal domain of TgNF3 (1 results) S ['21483487'], nucleosome activities (421 results) S ['21483487'], TgNF3 shares similarities (1 results) S ['21483487'], anti-TgNF3 antibodies (1 results) S ['21483487'], TgNF3-YFP (1 results) S ['21483487'], TgNF3 (1 results) S ['21483487'], regulate gene expression pertinent (30 results) D ['16287846']

PMC3068995	14	FALSE	8	FALSE		7	19	TRUE	15	TRUE	15	TRUE	12	TRU
PMC3068994	14	FALSE	20	FALSE		19	31	TRUE	26	TRUE	26	TRUE	23	TRU
PMC3068993	4	FALSE	18	FALSE		9	14	TRUE	12	TRUE	10	TRUE	8	TRU

JE	TRUE	6	TRUE	IAV entry (16 results) S ['21483486'], macropinocytic (81 results) D ['20439710', '19279100'], stomatitis virus enters (29 results) D ['19390604', '7931146'], dynamin-dependent (258 results) S ['21483486'], entry of IAV (16 results) S ['21483486'], polymerization-dependent macropinocytic pathway (1 results) D ['19279100'], distinct forms of macropinocytosis (3 results) D ['20439710'], enters cells through vesicles (272 results) D ['19390604'], enters cells through vesicles (272 results) S ['21483486'], polymerization-dependent macropinocytic (2 results) D ['19279100'], stomatitis virus enters cells (27 results) D ['19390604', '7931146'], forms of macropinocytosis (23 results) D ['20439710'], actin polymerization-dependent macropinocytic (2 results) D ['19279100'], herpesvirus utilizes (144 results) D ['19279100'], macropinocytosis (597 results) S ['21483486'], actin polymerization-dependent macropinocytic pathway (1 results) D ['19279100'], IAV entry route (1 results) S ['21483486'], IAV (269 results) S ['21483486'], macropinocytosis for host-cell (29 results) D ['20439710', '19404330'], macropinocytosis for host-cell (29 results) S ['21483486'], coxsackievirus entry through epithelial (18 results) D ['16413486'], macropinocytic pathway (5 results) D ['19279100']
JE	TRUE	17	TRUE	number of LAPCs (1 results) S ['21483485'], sheep retrovirus capsid protein (2 results) D ['19081577', '12014498'], predominant neoplastic cell type (2 results) D ['12014498'], sheep retrovirus (152 results) D ['20219922'], sheep retrovirus (152 results) S ['21483485'], LAPCs (4 results) S ['21483485'], pulmonary adenomatosis (423 results) D ['19114283', '19081577'], pulmonary adenomatosis (423 results) S ['18818869'], JSRV (139 results) D ['20219922'], JSRV (139 results) S ['21483485', '19244321'], predominant neoplastic cell (5 results) D ['12014498'], Jaagsiekte (234 results) D ['20219922'], Jaagsiekte (234 results) S ['21483485'], jaagsiekte sheep retrovirus (149 results) D ['20219922'], jaagsiekte sheep retrovirus (149 results) S ['21483485'], adult sheep (1585 results) S ['21483485'], ovine (16255 results) S ['21483485'], sheep retrovirus capsid (52 results) D ['19081577', '12014498'], Jaagsiekte retrovirus (23 results) S ['10196296', '9000089', '8627682', '7595380'], lifespan of sheep (90 results) S ['21483485'], jaagsiekte sheep (307 results) D ['20219922'], jaagsiekte sheep (307 results) S ['21483485'], jaagsiekte sheep retrovirus capsid (3 results) D ['19081577', '12014498'], envelope protein of jaagsiekte (59 results) D ['20219922', '17177996'], envelope protein of jaagsiekte (59 results) S ['19244321'], possess abundant LAPCs (1 results) S ['21483485'], JSRV infection (13 results) D ['17177996', '12596895'], JSRV infection (13 results) S ['21483485', '15950254', '10196296'], commercial lifespan of sheep (3 results) S ['15950254'], replication for Jaagsiekte (31 results) D ['17177996', '16352558', '15829964'], replication for Jaagsiekte (31 results) S ['19244321', '18818869', '17997604', '16873259', '12890624'], ovine pulmonary (1451 results) S ['21483485'], retrovirus (13450 results) S ['21483485'], pulmonary adenocarcinoma (960 results) S ['21483485']
JE	TRUE	8	TRUE	genomic island PPHGI-1 (4 results) S ['21483484', '21199566', '20672876', '16360685'], syringae (2637 results) S ['21483484'], F532 grown (1 results) S ['21483484'], cultivar-specificity toward pea (1 results) D ['8126435'], Review Pseudomonas syringae (103 results) S ['19849780'], sequential Pseudomonas aeruginosa airway (12 results) D ['11021913'], island PPHGI-1 (4 results) S ['21483484', '21199566', '20672876', '16360685'], phaseolicola 1302A (4 results) S ['21483484', '20672876', '11069647'], resistance response leads (2001 results) S ['21483484'], phaseolicola colony (7 results) S ['20672876'], PPHGI-1 (4 results) S ['21483484', '21199566', '20672876', '16360685'], phaseolicola colony development (1 results) S ['20672876'], F532 cells (9 results) S ['21483484'], genomic island (294 results) S ['21483484', '21199566'], phaseolicola F532 (1 results) S ['21483484'], F532 (20 results) S ['21483484'], Loss of PPHGI-1 (3 results) S ['21483484', '21199566', '16360685'], Pseudomonas syringae (2539 results) S ['21483484'], other incompatibility group (3 results) D ['8126435'], Helicobacter genome evolution (222 results) D ['20478826'], phaseolicola (279 results) S ['21483484', '21199566', '20672876'], bean reveals (117 results) S ['20672876']

PMC3077324	2	FALSE	16	FALSE		2	18	TRUE	16	TRUE	14	TRUE	13	TRU
PMC3072745	1	FALSE	7	FALSE		6	5	TRUE	4	TRUE	4	TRUE	3	TRU
PMC3072744	1	FALSE	5	FALSE		3	6	TRUE	5	TRUE	5	TRUE	5	TRU
PMC3072743	9	FALSE	1	FALSE		8	10	FALSE	10	TRUE	9	TRUE	9	TRU
PMC3072741	4	FALSE	9	FALSE		5	12	FALSE	12	FALSE	12	FALSE	12	TRU
PMC3077330	8	FALSE	21	FALSE		9	27	TRUE	20	TRUE	19	TRUE	14	TRU

JE	TRUE	10	TRUE	emergency consent (6 results) S ['21453454'], voluntary participation (250 results) S ['21453454'], point of enrolment (85 results) S ['21453454'], emergency interventions (120 results) S ['21453454'], fluid resuscitation (2428 results) S ['21453454'], pediatric resuscitation community consultation (6 results) D ['15342853'], many district hospitals (562 results) S ['21453454'], specific emergency consent process (22 results) D ['15342853'], specific emergency consent process (22 results) S ['21453454'], assent (464 results) S ['21453454'], ethics committees (7967 results) S ['21453454'], trial assent waiver (4 results) S ['21453454'], trial of fluid (20088 results) S ['21453454'], emergency research (172 results) S ['21453454'], specific emergency consent (122 results) S ['21453454'], consent process (982 results) S ['21453454'], verbal assent (12 results) S ['21453454'], parental verbal assent (2 results) S ['21453454']
JE	TRUE	2	TRUE	pressure outcomes Belgian DRIVER (1 results) S ['21155943'], Effectiveness of valsartan (57 results) S ['21490947'], BP-CARE study (1 results) D ['21047877'], outcomes Belgian DRIVER (2 results) S ['21155943'], valsartan (1876 results) S ['21490947'], heuristic profile (1 results) S ['20689688'], valsartan for treatment (1521 results) S ['21490947'], outcomes PREVIEW (15 results) S ['19716190', '19351876']
JE	TRUE	5	TRUE	Can Doppler ultrasonographic indices (108 results) S ['19765420'], flow velocity (52022 results) S ['21490946'], transcranial Doppler (5419 results) S ['21490946'], blood flow velocity (48235 results) S ['21490946'], post-surgical evaluation with transcranial (3 results) D ['10371076'], hypercholesterolemia (29245 results) S ['21490946']
JE	TRUE	5	TRUE	Heart Association Task Force (258 results) D ['21173346'], Association Task Force (340 results) D ['21173346'], upstream therapy (36 results) D ['20334907'], Appendage Transcatheter Study (24 results) D ['19628179', '15992628'], design of apixaban (9 results) D ['20211294', '20211292'], antiarrhythmic gene therapy (758 results) S ['21490945'], Heart Association Task (258 results) D ['21173346'], Cardiology Committee for Practice (427 results) D ['21173346'], European Heart Rhythm (53 results) D ['20802247'], PLAATO Left Atrial (34 results) D ['19628179']
JE	TRUE	10	TRUE	olmesartan (691 results) S ['21490943'], olmesartan medoxomil (347 results) D ['21142262'], olmesartan medoxomil (347 results) S ['21490943'], young hypertensive patients (78 results) S ['21490943'], medoxomil (356 results) D ['21142262'], medoxomil (356 results) S ['21490943'], lifestyle changes (2652 results) S ['21490943'], oral olmesartan (60 results) D ['21142262', '20385971'], young hypertensive (3513 results) S ['21490943'], good tolerability (1192 results) S ['21490943'], oral olmesartan medoxomil (3 results) D ['21142262', '18547134', '11451212'], adolescents with hypertension (30045 results) S ['21490943'], good tolerability profile (131 results) S ['21490943']
JE	TRUE	10	TRUE	viral gene expression (1772 results) S ['21453528'], HDAC3 with HCMV (3 results) D ['18024889', '11867539'], HDAC3 with HCMV (3 results) S ['21453528'], viral gene (4389 results) S ['21453528'], AI050709 (11 results) S ['16226322'], interaction of HDAC3 (73 results) S ['21453528'], HCMV DNA (487 results) S ['21453528'], MIE locus (6 results) S ['21453528'], genes upstream of MIE (40 results) D ['15113893'], heterogeneous interaction of HDAC3 (1 results) S ['21453528'], HDAC inhibitors enhance viral (20 results) D ['16123451'], HDAC inhibitors enhance viral (20 results) S ['16765083'], HDAC2 (474 results) S ['21453528'], HDACs (1471 results) S ['21453528'], chromatinization (12 results) D ['18786996'], chromatinization (12 results) S ['21453528', '16765083'], HCMV genomic DNA (5 results) S ['21453528'], chromatinization of HCMV (1 results) S ['21453528'], HCMV genomic (1164 results) S ['21453528'], promoter of UL127 (5 results) D ['15113893'], DNA with HDACs (470 results) S ['21453528'], MIE promoter (36 results) D ['18024889'], MIE promoter (36 results) S ['21453528'], HCMV (3975 results) S ['21453528'], IE2 (431 results) S ['20631086'], transcription of UL127 (5 results) D ['15113893'], HCMV DNA with HDACs (4 results) D ['18024889'], HCMV DNA with HDACs (4 results) S ['21453528', '20631086'], HDAC3 (347 results) S ['21453528']

PMC3072930	9	FALSE	19	FALSE		9	25	TRUE	22	TRUE	15	TRUE	13	TRU
PMC3073960	1	FALSE	5	FALSE		5	6	FALSE	6	FALSE	6	FALSE	6	TRU
PMC3072115	25	FALSE	8	FALSE		3	21	TRUE	18	TRUE	17	TRUE	14	TRU
PMC3072114	0	TRUE	7	FALSE		2	5	TRUE	4	TRUE	3	TRUE	3	TRU
PMC3078862	3	FALSE	9	FALSE		7	12	TRUE	11	TRUE	10	TRUE	6	TRU

JE	TRUE	10	TRUE	ribosome entry (1486 results) S ['21450110'], ribosome entry site (1190 results) S ['21450110'], DHV-1 genome (3 results) D ['17067712'], DHV-1 genome (3 results) S ['21450110'], HCV-like (59 results) D ['18077729'], HCV-like (59 results) S ['21450110'], internal ribosome entry site (1185 results) S ['21450110'], 5'-UTR of DHV-1 (6 results) D ['17030865'], 5'-UTR of DHV-1 (6 results) S ['21450110'], DHV-1 IRES (1 results) S ['21450110'], porcine enterovirus-8 simian (3 results) D ['17392358', '16528042'], normal internal initiation (516 results) S ['21450110'], DHV-1 (17 results) D ['17067712', '17030865'], DHV-1 (17 results) S ['21450110'], requirement for eIF4F (56 results) D ['16287867'], 5'-UTR (2482 results) S ['21450110'], DHV-1 5'-UTR (6 results) D ['17030865'], DHV-1 5'-UTR (6 results) S ['21450110'], IRES recruits ribosomal (11 results) D ['16556939', '9660832'], picornavirus (1352 results) S ['21450110'], internal initiation (410 results) S ['21450110'], DHV-1 IRES structure (1 results) S ['21450110'], IRES element within DHV-1 (1 results) S ['21450110'], internal ribosome (1505 results) S ['21450110'], genome of DHV-1 (10 results) D ['17067712', '17030865'], genome of DHV-1 (10 results) S ['21450110'], internal ribosome entry (1468 results) S ['21450110'], eIF4F (333 results) S ['21450110']
JE	TRUE	5	TRUE	platelet membrane (8593 results) S ['21450092'], Tph2 (312 results) S ['21450092'], Bio molecular considerations (22 results) S ['20518726'], intestinal serotonin inhibits (103 results) D ['19041748'], membrane viscosity (221 results) S ['21450092', '20825633', '20525273'], ontological (979 results) S ['21450092']
JE	TRUE	9	TRUE	perfusion multiple-exposure laser speckle (1 results) D ['20459289'], spatial averaging (163 results) S ['21483623'], laser Doppler-like spectra (1 results) D ['20459289'], Doppler-like (7 results) D ['20459289'], multiple-exposure laser speckle analysis (3 results) D ['20459289'], speckle analysis generates laser (3 results) D ['20459289'], Tissue perfusion multiple-exposure laser (1 results) D ['20459289'], laser speckle analysis (220 results) D ['20459289'], laser speckle analysis (220 results) S ['21483623'], laser speckle (495 results) S ['21483623'], speckle (3366 results) S ['21483623'], speckle analysis (18 results) D ['20459289'], multiple-exposure laser (20 results) D ['20459289'], multiple-exposure laser speckle (1 results) D ['20459289'], Tissue perfusion multiple-exposure (1 results) D ['20459289'], speckle size (48 results) D ['19079481'], pixel size (568 results) S ['21483623'], speckle analysis generates (12 results) D ['20459289'], generates laser Doppler-like (1 results) D ['20459289'], Doppler-like spectra (1 results) D ['20459289'], laser speckle analysis generates (3 results) D ['20459289'], perfusion multiple-exposure (1 results) D ['20459289'], minimum speckle (57 results) D ['19079481'], analysis generates laser (262 results) D ['20459289'], speckle contrast (148 results) S ['21483623'], linear correction (53 results) S ['21483623'], multiple-exposure (352 results) D ['20459289'], generates laser Doppler-like spectra (1 results) D ['20459289'], analysis generates laser Doppler-like (1 results) D ['20459289'], laser Doppler-like (1 results) D ['20459289'], perfusion multiple-exposure laser (1 results) D ['20459289'], laser speckle contrast (60 results) D ['20459289'], laser speckle contrast (60 results) S ['21483623']
JE	TRUE	3	TRUE	phantom-lesions (9 results) S ['21483622'], agent Omocianine (1 results) S ['19798535'], scatterplot of fluorescence (5 results) S ['21483622'], fluorescence images (744 results) S ['21483622'], scatterplot (190 results) S ['21483622'], scatterplots (226 results) S ['21483622'], Omocianine (1 results) S ['19798535']
JE	TRUE	4	TRUE	influenza dynamics (17 results) D ['18801714', '17185596'], influenza dynamics (17 results) S ['19379841'], evolution of influenza (1984 results) S ['21450071'], Water-borne transmission (30 results) S ['19379841'], indirect influenza transmission (52 results) D ['19081209'], influenza evolution (13 results) D ['12660783'], influenza evolution (13 results) S ['21450071'], dominance of strain (650 results) S ['21352779'], Camargue area (9 results) S ['19379841'], environmental transmission (107 results) S ['21352779'], avian influenza dynamics (4 results) S ['19379841'], evolutionary dynamics (1419 results) S ['21450071']

PMC3078864	3	FALSE	13	FALSE		5	13	TRUE	12	TRUE	12	TRUE	10	TRU
PMC3078888	8	FALSE	20	FALSE		6	22	TRUE	16	TRUE	16	TRUE	16	TRU
PMC3072960	5	FALSE	21	FALSE		7	20	TRUE	17	TRUE	15	TRUE	8	TRU
PMC3078849	0	TRUE	8	FALSE		2	6	TRUE	4	TRUE	4	TRUE	4	TRU
PMC3072347	2	FALSE	6	FALSE		3	8	TRUE	7	TRUE	7	TRUE	7	TRU

JE	TRUE	8	TRUE	transposase (2150 results) S ['21447194'], piggyBac (229 results) S ['21447194'], active transposon (20 results) S ['17005721'], Tol2 hotspots (1 results) S ['21447194'], DNA transposons (196 results) S ['21447194'], cons of piggyBac (1 results) S ['21447194'], short piggyBac (17 results) D ['19397814'], site-specific therapeutic gene targeting (136 results) S ['21447194'], site-specific therapeutic gene (682 results) S ['21447194'], SB11 hyperactive (5 results) S ['17005721'], Tol2 (105 results) D ['20372108'], Tol2 (105 results) S ['21447194'], therapeutic gene (1186 results) S ['21447194'], piggyBac target (35 results) D ['20372108', '19471016'], piggyBac target (35 results) S ['21447194'], Tol2 display (1 results) S ['21447194']
JE	TRUE	8	TRUE	reef fishes (198 results) S ['21450094'], shape disparity over ontogeny (1 results) S ['21450094'], ontogeny of damselfishes (1 results) S ['21450094'], shape disparity (4 results) S ['21450094'], damselfishes exemplifies (1 results) S ['21450094'], bipartite life-cycle of damselfishes (1 results) S ['21450094'], perch Cymatogaster aggregata Gibbons (1 results) D ['17714296'], divergence of allometric (31 results) S ['21450094'], coral reef fishes (98 results) S ['21450094'], coral reef (939 results) S ['21450094'], variation of disparity (596 results) S ['21450094'], allometric (2424 results) S ['21450094'], disparity over ontogeny (3 results) S ['21450094'], ontogenetic allometry (30 results) D ['17714296'], ontogenetic allometry (30 results) S ['21450094'], diverse reef fish phylogenetics (1 results) D ['19135160'], radiation of coral (338 results) S ['21450094'], length of ontogenetic (278 results) S ['21450094'], Habitat-dependent geographical (2 results) D ['17714296'], Habitat-dependent geographical variation (2 results) D ['17714296'], damselfishes (24 results) D ['19183467', '19135160'], damselfishes (24 results) S ['21450094', '17935195'], ontogenetic (4482 results) S ['21450094'], life-cycle of damselfishes (2 results) S ['21450094'], ontogenetic trajectories (54 results) D ['20637087'], ontogenetic trajectories (54 results) S ['21450094'], allometry (989 results) S ['21450094'], reef fish phylogenetics (2 results) D ['19135160']
JE	TRUE	6	TRUE	data warehouse Rice (3 results) S ['21450055'], bioinformatic analysis (1359 results) S ['21450055'], Rice Genome Annotation (13 results) D ['17145706', '15888674', '12519988'], bioinformatic analysis platforms (40 results) S ['21450055'], data warehouse Rice Mart (1 results) S ['21450055'], annotation entries (208 results) S ['21450055'], BioMart-powered (1 results) S ['21450055'], annotation tracks for japonica (1 results) S ['21450055'], tracks for japonica (4 results) S ['21450055'], japonica (7054 results) S ['21450055'], annotation tracks (15 results) D ['12045153'], annotation tracks (15 results) S ['21450055'], indica annotation (19 results) D ['20627892', '12519988'], indica annotation (19 results) S ['21450055'], Rice Mart (1 results) S ['21450055'], ideal rice architecture gene (6 results) S ['20495565'], pre-computed (119 results) S ['21450055'], SNPs between japonica (52 results) D ['20627892'], annotation entries interactively (3 results) S ['21450055'], warehouse Rice Mart (1 results) S ['21450055'], genome browser (322 results) S ['21450055'], annotation for indica (19 results) D ['20627892', '12519988'], annotation for indica (19 results) S ['21450055'], rice genome (854 results) S ['21450055'], Rice-Map (8 results) S ['21450055'], intra-species homologies (1 results) S ['21450055']
JE	TRUE	1	TRUE	community occupational therapy (22 results) S ['21450063', '18171718'], CI-strategy (3 results) S ['21450063'], educational interventions managers (67 results) S ['21450063'], COTiD (1 results) S ['21450063'], adherence of OTs (4 results) S ['21450063'], usual educational strategy (56 results) S ['21450063'], OTs (644 results) S ['21450063'], COTiD program (1 results) S ['21450063']
JE	TRUE	4	TRUE	participants' prior beliefs (50 results) D ['21450083'], participants' prior beliefs (50 results) S ['21450084'], Moher (367 results) S ['21450084'], favorable systematic review (8857 results) S ['21450084'], PRISMA statement (6 results) S ['21450084', '19622552'], PRISMA (569 results) S ['21450084'], favorable systematic review results (2818 results) S ['21450084'], cross-sectional study of SRs (53 results) D ['21450083']

PMC3073869	0	TRUE	15	FALSE		1	15	TRUE	14	TRUE	13	TRUE	13	TRU
PMC3078092	7	FALSE	16	FALSE		4	19	TRUE	14	TRUE	13	TRUE	13	TRU
PMC3078848	0	TRUE	4	FALSE		1	4	FALSE	4	FALSE	4	FALSE	4	TRU
PMC3072324	11	FALSE	11	FALSE		5	22	TRUE	21	TRUE	18	TRUE	14	TRU
PMC3076274	4	FALSE	15	FALSE		4	18	TRUE	17	TRUE	16	TRUE	13	TRU
PMC3074546	0	TRUE	6	FALSE		2	6	FALSE	6	FALSE	6	FALSE	6	FAL

JE	TRUE	8	TRUE	clinical microbiology results (3 results) S ['21447199'], microbiology reports differ (497 results) S ['21447199'], microbiology results (79 results) S ['21447199'], reduction of hardcopy (9 results) S ['21447199'], post-interview study (25 results) S ['21447199'], correct communication of results (2227 results) S ['21447199'], hardcopy (147 results) S ['21447199'], therapy decisions (208 results) S ['21447199'], clinical microbiologist (74 results) S ['21447199'], culture results (3319 results) S ['21447199'], microbiology reports (63 results) S ['21447199'], meaning of microbiology (372 results) S ['21447199'], turnaround time of microbiological (256 results) S ['21447199'], microbiologist (523 results) S ['21447199'], electronic reporting (91 results) S ['21447199']
JE	TRUE	7	TRUE	Shewanella (1291 results) S ['21450087'], oneidensis (437 results) S ['21450087'], etrA knockout (3 results) D ['11466298'], EtrA7-1 (1 results) S ['21450087'], regulatory role of EtrA (4 results) D ['11466298'], regulatory role of EtrA (4 results) S ['21450087'], EtrA7-1 mutant (1 results) S ['21450087'], fumarate (5168 results) S ['21450087'], etrA deletion (1 results) S ['21450087'], etrA knockout mutant (3 results) D ['11466298'], anaerobic metabolism (1924 results) S ['21450087'], Fnr (1223 results) S ['21450087'], nitrate reduction (1140 results) S ['21450087'], Energy metabolism category (3 results) S ['21450087'], DMSO reduction pathways (66 results) D ['19395492'], DMSO reduction pathways (66 results) S ['21450087'], EtrA affects (6 results) D ['19060154'], EtrA (68 results) D ['19060154'], EtrA (68 results) S ['21450087'], electron acceptors (2336 results) S ['21450087'], role of EtrA (23 results) D ['11466298'], role of EtrA (23 results) S ['21450087'], etrA deletion mutant (1 results) S ['21450087']
JE	TRUE	3	TRUE	CSF acute phase factors (298 results) S ['21450100'], Lipoperoxide radical (75 results) S ['21450100'], ischemic role of nitric (2384 results) S ['21450100'], stroke severity (1076 results) S ['21450100']
JE	TRUE	10	TRUE	small-vessel occlusion stroke (122 results) S ['21447190'], ISODATA ischemic (17 results) D ['12468779'], Multiparametric MRI ISODATA (8 results) D ['12468779'], edaravone (355 results) S ['21447190'], edaravone on acute (142 results) D ['20491547'], edaravone on acute (142 results) S ['21447190'], effect of edaravone (225 results) S ['21447190'], ISODATA ischemic lesion (15 results) D ['12468779'], small-vessel occlusion (101 results) S ['21447190'], radical scavenger (4321 results) S ['21447190'], lacunar infarction (649 results) S ['21447190'], free radical scavenger (2485 results) S ['21447190'], mri isodata ischemic (17 results) D ['12468779'], Multiparametric MRI ISODATA ischemic (8 results) D ['12468779'], small-vessel (3576 results) S ['21447190'], Edaravone versus (8 results) D ['19321945', '16595989'], ISODATA ischemic lesion correlation (5 results) D ['12468779'], occlusion stroke (23 results) S ['21447190'], edaravone treatment (48 results) S ['21447190'], mri isodata ischemic lesion (15 results) D ['12468779'], MRI ISODATA (21 results) D ['12468779'], single-parameter mri techniques (24 results) D ['12468779']
JE	TRUE	11	TRUE	prenatal decisions (8 results) D ['16927359'], termination of foetuses (40 results) S ['21447191'], prevention of thalassaemia (1026 results) S ['21447191'], premarital (1938 results) S ['21447191'], positive attitudes toward prevention (752 results) S ['21447191'], unsupportive of selective (1 results) S ['21447191'], educational interventions (1883 results) S ['21447191'], thalassaemia carrier (28 results) D ['16475227'], congruent educational interventions (11 results) S ['21447191'], selective termination (197 results) S ['21447191'], thalassaemia major (859 results) S ['21447191'], attitudes toward thalassaemia (13 results) S ['21447191'], awareness of thalassaemia (102 results) D ['19390864'], awareness of thalassaemia (102 results) S ['21447191'], selective termination of foetuses (3 results) S ['21447191'], pregnancies with thalassaemia (3058 results) S ['21447191'], knowledge of thalassaemia (344 results) S ['21447191'], prenatal decisions for sickle (16 results) D ['16927359'], thalassaemia (3672 results) S ['21447191']
.SE	TRUE	6	TRUE	antineoplastic drugs (1397 results) S ['21450074', '21112930'], occupationally (5152 results) S ['21450074'], metabolic detoxification glutathione (1410 results) S ['21450074'], female nurses (541 results) S ['21450074'], drug contaminations on surfaces (493 results) S ['21112930'], cancer risks of nurses (2123 results) S ['21450074']

PMC3078870	9	FALSE	13	FALSE		7	19	TRUE	11	TRUE	10	TRUE	10	TRU
PMC3078090	1	FALSE	12	FALSE		2	13	FALSE	13	TRUE	10	TRUE	10	TRU
PMC3076271	2	FALSE	13	FALSE		3	12	FALSE	12	TRUE	10	TRUE	10	TRU
PMC3072334	14	FALSE	14	FALSE		8	28	TRUE	26	TRUE	18	TRUE	14	TRU

JE	TRUE	2	TRUE	subglottic drainage port blockage (1 results) D ['17898392'], subglottic (2677 results) S ['21450078'], elective exchange (8 results) S ['21450078'], intermittent subglottic secretion (3 results) D ['20522796'], intermittent subglottic secretion (3 results) S ['21450078'], intermittent SSD (5 results) D ['20522796'], PneuX (1 results) S ['21450078'], tracheal seal monitor (4 results) S ['21450078'], secretion drainage (38 results) D ['20522796', '18359430', '15639202'], secretion drainage (38 results) S ['21450078'], LVLP cuff (2 results) D ['16505646'], patients underwent elective exchange (21 results) S ['21450078'], subglottic secretion (31 results) D ['20522796', '18359430', '15639202'], subglottic secretion (31 results) S ['21450078'], subglottic secretion drainage (24 results) D ['20522796', '18359430', '15639202'], subglottic secretion drainage (24 results) S ['21450078'], PneuX System (1 results) S ['21450078'], incidence of VAP (771 results) S ['21450078'], intermittent subglottic secretion drainage (3 results) D ['20522796'], intermittent subglottic secretion drainage (3 results) S ['21450078'], underwent elective exchange (31 results) S ['21450078'], subglottic drainage port (5 results) D ['17898392', '15483416']
JE	TRUE	6	TRUE	spasmodic physical activity (46 results) S ['21447192'], Australian farmers (7 results) S ['21447192'], abdominal adiposity (600 results) S ['21447192'], farm men (10 results) D ['19292570'], farm men (10 results) S ['21447192'], higher mental health burden (527 results) S ['21447192'], Rural Australians (35 results) S ['21447192'], long-term stress with feelings (933 results) S ['21447192'], higher mental health (43 results) S ['21447192'], high mental health burden (619 results) S ['21447192'], systemic cortisol (28 results) S ['21447192'], increases systemic cortisol that (152 results) S ['21447192'], complex correlation between obesity (169 results) S ['21447192']
JE	TRUE	8	TRUE	field workers (670 results) S ['21450064'], expectant (3191 results) S ['21450064'], community health workers (823 results) S ['21450064'], IEC interventions (8 results) D ['10977263'], Antenatal Care Handbook (6 results) S ['21450064'], handbook system (1 results) D ['10135606'], ANC handbook (1 results) S ['21450064'], understanding of ANC (32 results) S ['21450064'], expectant women (55 results) S ['21450064'], health facility (19720 results) S ['21450064'], facility arm (217 results) S ['21450064'], health facility arm (1022 results) S ['21450064'], ANC handbook system (1 results) S ['21450064'], pretesting (513 results) S ['21450064'], ANC (1933 results) S ['21450064']
JE	TRUE	8	TRUE	FATP4 function (57 results) S ['21450060', '19631310'], FATP domains (5 results) D ['18258213'], congenital verruciform hyperkeratosis (5 results) D ['20815031'], fatty acid transport protein (200 results) D ['20815031'], fatty acid transport protein (200 results) S ['21450060'], acid transport (9252 results) S ['21450060'], FATP motif (6 results) D ['18258213'], FATP4 protein (8 results) D ['18258213'], human FATP4 protein (39 results) D ['17495600'], human FATP4 protein (39 results) S ['19631310'], functional analysis of FATP4 (6 results) S ['21450060'], prematurity syndrome (8 results) D ['19119129'], prematurity syndrome (8 results) S ['21450060', '19631310', '14985385'], ichthyosis (4172 results) S ['21450060'], congenital verruciform (14 results) D ['20815031'], fatty acid transport (825 results) S ['21450060'], analysis of FATP4 (17 results) D ['17062637'], analysis of FATP4 (17 results) S ['21450060'], ichthyosis prematurity (56 results) D ['20815031', '19119129'], ichthyosis prematurity (56 results) S ['21450060', '19631310'], congenital ichthyosis subtype (7 results) D ['19119129'], ichthyosis subtype (18 results) D ['19119129'], FATP4 (62 results) S ['21450060', '19631310'], ichthyosis prematurity syndrome (8 results) D ['19119129'], ichthyosis prematurity syndrome (8 results) S ['21450060', '19631310', '14985385'], acid transport protein (249 results) D ['20815031'], acid transport protein (249 results) S ['21450060'], transport protein (4686 results) S ['21450060']

PMC3076300	7	FALSE	9	FALSE		8	16	TRUE	15	TRUE	10	TRUE	9	TRU
PMC3068305	4	FALSE	6	FALSE		7	9	TRUE	5	TRUE	2	TRUE	2	TRU
PMC3074550	3	FALSE	9	FALSE		2	12	TRUE	11	TRUE	10	TRUE	9	TRU
PMC3072924	12	FALSE	22	FALSE		8	33	TRUE	26	TRUE	22	TRUE	17	TRU

JE	TRUE	6	TRUE	RPR (865 results) S ['21447195'], Paeoniae Rubra (50 results) S ['21447195'], Mycobacterium-induced (31 results) S ['16301626'], NF-kappaB1 homodimers (9 results) D ['16835236'], Rubra on acute (105 results) D ['18230290', '16723077'], Paeoniae (213 results) S ['21447195'], Dual knockdown of p65 (6 results) D ['17530443'], Radix Paeoniae Rubra (48 results) S ['21447195'], Radix Paeoniae (109 results) S ['21447195'], Paeoniae Rubra on acute (7 results) D ['18230290', '16723077'], mycobacterial immune evasion (19 results) S ['21447195', '19401395'], Astragalus membranaceus against liver (8 results) D ['18816297'], Radix (2911 results) S ['21447195'], human primary synoviocytes (3 results) D ['17530443'], membranaceus against liver (8 results) D ['18816297'], Rubra (2098 results) S ['21447195']
JE	TRUE	2	TRUE	Okinawan diet (4 results) S ['20234038', '17986602'], traditional healthy Okinawan (4 results) S ['20234038', '17986602', '11710359'], Okinawan (251 results) S ['20234038', '21490698'], Drosophila confers hypersensitivity (6 results) D ['2539600'], Okinawan centenarians (9 results) S ['21490698'], younger controls (249 results) S ['21490698'], lipid peroxide plasma levels (2 results) D ['11380744'], lipoprotein tocopherol (7 results) D ['9322573'], traditional healthy Okinawan diet (3 results) S ['20234038', '17986602', '11710359'], peroxide plasma levels (4 results) D ['11380744']
JE	TRUE	6	TRUE	felodipine (1389 results) S ['21450068'], cardiac fibrosis (922 results) S ['21450068'], felodipine combination (238 results) S ['21450068'], collagen III (812 results) S ['21450068'], significant decrease for T-Chol (7 results) S ['21450068'], hyperglycaemia induces pancreatic morphogenesis (2 results) D ['17047919'], nude mice without hyperglycaemia (99 results) D ['17047919'], T-Chol (102 results) S ['21450068'], role for felodipine (94 results) S ['21450068'], decrease for T-Chol (12 results) S ['21450068'], combination of rosiglitazone (540 results) S ['21450068'], induces pancreatic morphogenesis (44 results) D ['17047919']
JE	TRUE	8	TRUE	PTTM shows (22 results) D ['20554399', '19788622', '18835104', '17219075', '15660700'], PTTM shows (22 results) S ['21450103'], PTTM cases (6 results) D ['20554399', '18835104'], PTTM cases (6 results) S ['21450103'], development of PTTM (2 results) D ['19788622'], Pulmonary tumor thrombotic (26 results) D ['20554399', '19788622', '18835104', '17539970', '17290069', '17219075', '15660700'], Pulmonary tumor thrombotic (26 results) S ['21450103'], analysis of autopsy (18968 results) S ['21450103'], gastric carcinoma (8887 results) S ['21450103'], cases of PTTM (6 results) D ['20554399', '18835104'], cases of PTTM (6 results) S ['21450103'], microangiopathy (4491 results) S ['21450103'], tumor thrombotic (28 results) D ['20554399', '19788622', '18835104', '17539970', '17290069', '17219075'], tumor thrombotic (28 results) S ['21450103'], tumor thrombotic microangiopathy (28 results) D ['20554399', '19788622', '18835104', '17539970', '17290069', '17219075'], tumor thrombotic microangiopathy (28 results) S ['21450103'], thrombotic microangiopathy (1292 results) S ['21450103'], TF-positive rate (4 results) S ['21450103'], autopsy cases (3406 results) S ['21450103'], higher TF-positive (20 results) S ['21450103'], PTTM cases among gastric (6 results) D ['20554399', '18835104'], PTTM cases among gastric (6 results) S ['21450103'], micrometer group (2 results) S ['21450103'], Pulmonary tumor thrombotic microangiopathy (26 results) D ['20554399', '19788622', '18835104', '17539970', '17290069', '17219075', '15660700'], Pulmonary tumor thrombotic microangiopathy (26 results) S ['21450103'], incidence of PTTM (1 results) D ['20554399'], Pulmonary tumor (652 results) S ['21450103'], immunohistochemical positive rate (3466 results) S ['21450103'], typical gastric carcinoma (373 results) S ['21450103'], under micrometer group (66 results) S ['21450103'], higher TF-positive rate (2 results) S ['21450103'], PTTM (22 results) D ['20554399', '19788622', '18835104', '17219075', '15660700'], PTTM (22 results) S ['21450103'], patients with PTTM (9 results) D ['20554399', '19788622', '17219075']

PMC3077039	12	FALSE	11	FALSE		8	18	TRUE	15	TRUE	13	TRUE	12	TRU
PMC3072933	7	FALSE	15	FALSE		12	22	TRUE	21	TRUE	21	TRUE	17	TRU
PMC3068306	12	FALSE	13	FALSE		7	23	TRUE	15	TRUE	13	TRUE	8	TRU
PMC3071493	2	FALSE	0	TRUE		3	2	TRUE	1	TRUE	1	TRUE	1	TRU

JE	TRUE	6	TRUE	whole-genome SSR panel (4 results) S ['21454301'], Japanese cultivar (74 results) S ['19531560'], Belo Alcochete (1 results) D ['17517133'], simple sequence repeat (1259 results) S ['21454301'], simple sequence (2360 results) S ['21454301'], Choi Hyten (8 results) D ['18278477', '17339218'], panel for high-throughput (523 results) S ['21454301'], whole-genome SSR (40 results) S ['21454301'], Livak Rafalski (2 results) D ['1979162'], sequence repeat (1470 results) S ['21454301'], SSR panel (51 results) D ['19588255'], SSR panel (51 results) S ['21454301'], Kubelik Livak Rafalski (1 results) D ['1979162'], Kubelik Livak (1 results) D ['1979162'], Hyten Choi (8 results) D ['18278477', '17339218'], Williams Kubelik Livak Rafalski (1 results) D ['1979162'], SSR loci (289 results) S ['21454301'], Williams Kubelik Livak (1 results) D ['1979162'], SSR markers (949 results) S ['21454301'], Tsubokura Hoshi (2 results) D ['18192280'], high-density linkage map (22 results) D ['18192280'], high-density linkage map (22 results) S ['19531560'], soybean transcript gene distribution (14 results) D ['17339218']
JE	TRUE	11	TRUE	environmental lead exposure (167 results) S ['21450073'], childhood lead (438 results) D ['20947467', '20226811'], Low-level environmental lead (17 results) S ['21450073', '16002379'], Low-level environmental lead exposure (16 results) S ['21450073', '16002379'], Yugoslavia Prospective Lead contributions (2 results) D ['11120386'], blood-lead (4323 results) S ['21450073'], blood-lead concentration (517 results) S ['21450073'], predicts intellectual functioning (12 results) S ['21450073'], blood lead (4323 results) S ['21450073'], childhood blood-lead concentration (104 results) D ['20947467', '18507499', '18507497', '18288325'], childhood blood-lead concentration (104 results) S ['21450073'], Lead contributions of prenatal (14 results) D ['11120386'], Full-Scale (4528 results) S ['21450073'], childhood blood-lead (47 results) D ['20947467', '20226811', '19619581', '18507499', '18507497', '16950987'], childhood blood-lead (47 results) S ['21450073'], lead exposure (3472 results) S ['21450073'], school-age lead exposure (43 results) D ['19167707'], school-age lead exposure (43 results) S ['21450073'], environmental lead (513 results) S ['21450073'], lead concentrations (1728 results) S ['21450073'], Low-level environmental (97 results) S ['21450073'], childhood lead exposure (74 results) D ['20947467', '20226811', '19672413', '19619581', '18507499', '18507497']
JE	TRUE	6	TRUE	expression of AMY1 (57 results) S ['18279338'], characterization of Schwanniomyces (19 results) D ['3502323', '6181739'], alpha-amylase (5859 results) S ['21490699'], alluvius amylolytic (2 results) D ['6181739'], yeast Cryptococcus flavus (3 results) S ['14987760'], alluvius (15 results) D ['3502323', '6181739'], Cryptococcus flavus (14 results) S ['21490699', '18279338', '14987760'], Biochemical characterization of alpha-amylase (60 results) S ['21490699'], flavus (3709 results) S ['21490699'], alpha-amylase gene from Cryptococcus (4 results) S ['21490699', '18279338'], Schwanniomyces alluvius (9 results) D ['3502323', '6181739'], Alpha-Amylase from Cryptococcus (12 results) D ['8836148'], Alpha-Amylase from Cryptococcus (12 results) S ['21490699', '18279338', '14987760'], characterization of alpha-amylase (551 results) S ['21490699'], yeast Schwanniomyces alluvius (3 results) D ['3502323'], Amy1 (141 results) S ['21490699'], intermediate thermal stability (3 results) D ['7662314'], gene from Cryptococcus (906 results) S ['21490699'], alluvius amylolytic enzymes (1 results) D ['6181739'], new alpha-amylase of intermediate (15 results) D ['7662314'], Schwanniomyces alluvius amylolytic enzymes (1 results) D ['6181739'], extracellular alpha-amylase activity (5 results) D ['3502323'], Cryptococcus (8356 results) S ['21490699'], Schwanniomyces alluvius amylolytic (2 results) D ['6181739'], heterologous expression of AMY1 (3 results) S ['18279338']
JE	TRUE	0	FALSE	neuronal response latency (5 results) D ['6640330'], neuronal latency estimation (23 results) D ['17000007', '9765132']

PMC3070953	5	FALSE	13	FALSE		8	18	TRUE	16	TRUE	11	TRUE	10	TRU
PMC3070261	9	FALSE	17	FALSE		8	26	TRUE	25	TRUE	23	TRUE	20	TRU
PMC3073926	0	TRUE	7	FALSE		1	7	FALSE	7	FALSE	7	FALSE	7	TRU
PMC3078830	0	TRUE	15	FALSE		1	14	FALSE	14	TRUE	13	TRUE	12	TRU
PMC3078829	1	FALSE	16	FALSE		2	17	FALSE	17	FALSE	17	TRUE	15	TRU

JE	TRUE	6	TRUE	postnatal-generated (2358 results) S ['21490706'], neonatal insults (7 results) S ['21490706'], granule cells (7820 results) S ['21490706'], mature GCs (7 results) D ['19955357'], lower threshold for perturbation (37 results) S ['21490706'], dentate (16805 results) S ['21490706'], Steiner Klempin (3 results) D ['17105671'], neural production (7 results) S ['21490706'], neurons after neonatal (3814 results) S ['21490706'], short-term consequences for neuronal (83 results) S ['21490706'], threshold for perturbation (341 results) S ['21490706'], alter granule cell production (52 results) S ['21490706'], dendritic complexity of newborn (76 results) S ['21490706'], newborn GCs (7 results) D ['19955357'], mice alter granule cell (125 results) S ['21490706'], Overstreet-Wadiche (12 results) D ['19955357', '16611826', '16495460', '16033936'], Overstreet-Wadiche (12 results) S ['21490706', '18522595'], basal synaptic inhibition (2 results) D ['17148958']
JE	TRUE	14	TRUE	pallidus (8855 results) S ['21483725'], inhibitory pallidal GABAergic (39 results) D ['19087170', '16154280'], inhibitory pallidal GABAergic (39 results) S ['21483725'], parkinsonian input patterns (11 results) D ['16367790', '12077211'], repetitive stimulation (2216 results) S ['21483725'], transmission during stimulation (23404 results) S ['21483725'], pallidal (1845 results) S ['21483725'], pallidal GABAergic synapses (29 results) D ['19087170', '16154280'], pallidal GABAergic synapses (29 results) S ['21483725'], undergo short-term depression (34 results) S ['21483725'], globus (9023 results) S ['21483725'], globus pallidus (8229 results) S ['21483725'], synaptic transmission during stimulation (19352 results) S ['21483725'], characteristics of globus (284 results) S ['20700458'], Electrophysiological characteristics of globus (20 results) D ['19087170'], Electrophysiological characteristics of globus (20 results) S ['20700458'], Functional characterization of GABAergic (57 results) D ['19087170'], inhibitory pallidal (150 results) D ['19087170', '18355810', '18234984'], inhibitory pallidal (150 results) S ['21483725'], GABAergic pallidopallidal (2 results) D ['19087170'], inhibitory pallidal GABAergic synapses (11 results) D ['19087170', '16154280'], inhibitory pallidal GABAergic synapses (11 results) S ['21483725'], pallidal GABAergic (157 results) D ['19087170'], pallidal GABAergic (157 results) S ['21483725'], GABAergic synapses (625 results) S ['21483725'], Short-term depression (225 results) S ['21483725']
JE	TRUE	5	TRUE	HIV test result (108 results) S ['21450053'], program developers (67 results) S ['21450053'], positive HIV test result (49 results) S ['21450053'], positive HIV test (164 results) S ['21450053'], HIV test (1629 results) S ['21450053'], positive HIV (895 results) S ['21450053'], non-targeted (718 results) S ['21450053']
JE	TRUE	10	TRUE	Logan Healthy (242 results) S ['21450069'], pre-trial behavioural (6 results) S ['21450069'], behavior intervention trials (3912 results) S ['21450069'], intervention effects for fruit (537 results) S ['21450069'], behavioural screening (22 results) S ['21450069'], behaviour intervention trials (3912 results) S ['21450069'], pre-trial behavioural screening (1 results) S ['21450069'], trial retention rate (1956 results) S ['21450069'], behaviour intervention (14 results) S ['21450069'], multiple behaviour intervention (3676 results) S ['21450069'], intervention dose (41 results) S ['21450069'], multiple behavior intervention trials (363 results) S ['21450069'], multiple behaviour intervention trials (363 results) S ['21450069'], intervention trials (1919 results) S ['21450069'], intervention effects (961 results) S ['21450069']
JE	TRUE	8	TRUE	associations between television (1347 results) S ['21450065'], energy-dense snacks (17 results) S ['21450065'], intakes of energy-dense (99 results) S ['21450065'], lower fruit (48 results) D ['19183442'], higher intakes of energy-dense (47 results) S ['21450065'], energy-dense beverage consumption (41 results) S ['21450065'], intakes of fruit (1581 results) S ['21450065'], longitudinal associations between television (123 results) S ['21450065'], value of television (951 results) S ['21450065'], energy-dense drinks (48 results) S ['21450065'], consumption of energy-dense (240 results) S ['21450065'], energy-dense (584 results) S ['21450065'], adolescents place on television (81 results) S ['21450065'], higher energy-dense beverage (25 results) S ['21450065'], higher energy-dense beverage consumption (13 results) S ['21450065'], adolescents place (15105 results) S ['21450065'], longitudinal associations (376 results) S ['21450065']

PMC3076502	1	FALSE	16	FALSE		2	17	FALSE	17	FALSE	17	TRUE	16	TRU
PMC3070226	3	FALSE	22	FALSE		4	23	TRUE	21	TRUE	21	TRUE	14	TRU
PMC3070225	7	FALSE	27	FALSE		16	32	TRUE	28	TRUE	23	TRUE	19	TRU
PMC3070221	3	FALSE	7	FALSE		3	10	FALSE	10	FALSE	10	FALSE	10	FAL

JE	TRUE	10	TRUE	types from Korea (5241 results) S ['21494429'], green types from China (208 results) S ['21494429'], sea cucumber (419 results) S ['21494429'], hatchery (907 results) S ['21494429'], japonicus (2160 results) S ['21494429'], color variants (72 results) S ['21494429'], mean allele (63 results) S ['21494429'], green type (39 results) S ['21494429'], Color variation among sea (30 results) D ['17043749'], Color variation among sea (30 results) S ['21494429'], green type populations (1032 results) S ['21494429'], green types from Korea (69 results) S ['21494429'], type populations from Korea (820 results) S ['21494429'], genetic structure (4683 results) S ['21494429'], allele frequencies (8945 results) S ['21494429'], microsatellite makers (17 results) S ['21494429'], number of alleles (13558 results) S ['21494429']
JE	TRUE	12	TRUE	experience of falls (1096 results) S ['21468286'], lot' of urgency (1 results) S ['21468286'], KHQ (118 results) S ['21468286'], factors for falls (9119 results) S ['21468286'], OAB (927 results) S ['21468286'], risk factors for falls (5202 results) S ['21468286'], Efficacy Scale-International (28 results) S ['21468286'], Falls Efficacy Scale-International (28 results) S ['21468286'], group with OAB (230 results) S ['21468286'], treatment of OAB (710 results) S ['21468286'], group without OAB (230 results) S ['21468286'], risk of falls (811 results) S ['21468286'], OAB on falls (16 results) D ['12516954', '11183900'], OAB on falls (16 results) S ['21468286'], Guri (177 results) S ['21468286'], urge incontinence (1841 results) S ['21468286'], KFES-I (1 results) S ['21468286'], Scale-International (14 results) D ['16267188'], Scale-International (14 results) S ['21468286'], Yangpyeong (4 results) S ['21468286'], Yangpyeong Health (2 results) S ['21468286'], Falls Efficacy (143 results) S ['21468286'], Guri Health (14 results) S ['21468286'], OAB with falls (16 results) D ['12516954', '11183900'], OAB with falls (16 results) S ['21468286']
JE	TRUE	11	TRUE	mature fusiform (99 results) S ['21468280'], major urothelial differentiation (42 results) D ['15958488', '15611339'], major urothelial differentiation (42 results) S ['21468280'], urothelial cells (1172 results) S ['21468280'], urothelial plaque size (3 results) D ['9878432'], urothelial plaque size (3 results) S ['21468280'], II-deficient (560 results) S ['21468280'], 16-nm particles (12 results) D ['8983014', '8530366', '7760330'], 16-nm particles (12 results) S ['21468280'], heterodimers (6931 results) S ['21468280'], II-deficient urothelium (1 results) S ['21468280'], ablation completely abolishes (9 results) S ['21468280'], abolishes plaque formation (35 results) S ['21468280'], fusiform vesicles (31 results) D ['15085953', '12475947'], fusiform vesicles (31 results) S ['21468280'], complete epithelial occlusion (46 results) S ['21468280'], major urothelial (393 results) S ['21468280'], mature fusiform vesicles (4 results) S ['21468280'], urothelial plaques (21 results) D ['19161373', '16609152', '15958488', '12972502', '11256943', '11085999', '9878432', '8175808'], permeability barrier (1623 results) S ['21468280'], organelles migrate (753 results) S ['21468280'], completely abolishes plaque formation (6 results) S ['21468280'], epithelial occlusion (5 results) S ['21468280'], 16-nm (1236 results) S ['21468280'], urothelium (4865 results) S ['21468280'], ablation completely abolishes plaque (1 results) S ['21468280'], apical plaques (6 results) D ['8175808'], mammalian UPs (9377 results) S ['21468280'], major urothelial differentiation marker (12 results) D ['2229070'], major urothelial differentiation marker (12 results) S ['21468280'], apical surface (2984 results) S ['21468280'], completely abolishes plaque (6 results) S ['21468280'], urothelium shows (4865 results) S ['21468280'], urothelial differentiation (96 results) S ['21468280']
.SE	TRUE	10	TRUE	urine storage (152 results) D ['20808873'], urine storage (152 results) S ['21468279'], immediate attentions (3569 results) S ['21468279'], excess diuresis (275 results) D ['20519376'], circadian rhythms (7389 results) S ['21468279'], circadian control (365 results) S ['21468279'], nocturnal rodents (128 results) D ['20808873'], nocturnal rodents (128 results) S ['21468279'], urine production (765 results) S ['21468279'], possible circadian (3508 results) S ['21468279']

PMC3070220	1	FALSE	17	FALSE		4	17	TRUE	13	TRUE	13	TRUE	12	TRU
PMC3070219	0	TRUE	8	FALSE		1	8	FALSE	8	FALSE	8	FALSE	8	FAL
PMC3073896	2	FALSE	20	FALSE		4	20	TRUE	13	TRUE	11	TRUE	11	TRU
PMC3072308	4	FALSE	18	FALSE		7	19	TRUE	14	TRUE	12	TRUE	7	TRU
PMC3076253	1	FALSE	20	FALSE		3	19	FALSE	19	FALSE	19	TRUE	17	TRU

JE	TRUE	9	TRUE	intravesical pressure (761 results) S ['21468282'], simultaneous registrations (15 results) S ['21468282'], SCI rats (151 results) S ['21468282'], detrusor overactivity (1199 results) S ['21468282'], Simultaneous Registrations of Intravesical (3 results) S ['21468282'], registration of IVP (5 results) S ['19034716', '17565725'], Registrations of Intravesical (4 results) S ['21468282'], clinical significance of DSD (4 results) D ['11018603'], filling phase (453 results) S ['21468282'], vascular clip (64 results) S ['21468282'], simultaneous registrations of IVP (1 results) S ['21468282'], long-term urological (937 results) S ['21468282'], intraabdominal (6223 results) S ['21468282'], intravesical PGE2 (30 results) S ['17565725'], intravesical (6696 results) S ['21468282'], intraabdominal pressures (61 results) S ['21468282'], long-term urological management (269 results) S ['21468282'], IAP (4082 results) S ['21468282']
.SE	TRUE	8	TRUE	caveolin-1 (3080 results) S ['21468281'], caveolin (3988 results) S ['21468281'], ovariectomy (20986 results) S ['21468281'], expression of caveolin-1 (1573 results) S ['21468281'], urinary bladders of female (1863 results) S ['21468281'], urinary bladders of rats (867 results) S ['21468281'], bladders of rats (897 results) S ['21468281'], hormonal alterations (339 results) S ['21468281']
JE	TRUE	7	TRUE	poststernotomy mediastinitis (84 results) D ['19138422'], sternotomy wound (127 results) S ['19698860'], sternal edges during NPWT (2 results) S ['21450095'], edges during NPWT (4 results) S ['21450095'], barrier during NPWT (2 results) S ['21450095'], rigid barrier (30 results) S ['21450095', '19698860'], heart rupture with lethal (148 results) S ['21450095'], distension upon traction (48 results) S ['21450095'], sternal edges (27 results) S ['21450095', '19698860', '14667639'], sternotomy (5956 results) S ['21450095'], NPWT (131 results) S ['21450095'], rupture with lethal (725 results) S ['21450095'], rigid barrier during NPWT (1 results) S ['21450095'], rupture during NPWT (3 results) S ['21450095'], rigid barrier disc (2 results) S ['21450095'], sternum (9447 results) S ['21450095'], heart rupture (2583 results) S ['21450095'], sternal-sparing (7 results) S ['14667639'], protection during NPWT (1 results) S ['21450095'], NPWT after cardiac (9 results) D ['19138422'], NPWT with regard (3 results) S ['21450095'], lungs during NPWT (4 results) S ['21450095']
JE	TRUE	3	TRUE	zootherapy (17 results) D ['19128461', '16504024', '16270931', '12576209'], zootherapy (17 results) S ['21450096'], tarantula' (2 results) S ['21450096'], Chol (2225 results) S ['21450096'], non-Chol (1 results) S ['21450096'], traditional Chol medicine (10 results) S ['21450096'], medicine man (68 results) S ['21450096'], traditional Mayan (49 results) S ['21450096'], urticating (45 results) D ['20809805'], tarantula-based (1 results) S ['21450096'], traditional Mayan medicine (27 results) S ['21450096'], Chol communities (4 results) S ['21450096'], tarantula (265 results) D ['20809805'], tarantula (265 results) S ['21450096'], tarantula Brachypelma vagans (2 results) S ['21450096'], vagans (17 results) S ['21450096'], Brachypelma (17 results) S ['21450096'], Chol people (17 results) S ['21450096'], tarantula Brachypelma (7 results) S ['21450096'], village of Pedra (4 results) D ['17101055'], non-Chol people (1 results) S ['21450096'], Brachypelma vagans (2 results) S ['21450096']
JE	TRUE	15	TRUE	clinical diagnosis of piriformis (96 results) S ['21450058'], Brucella (9313 results) S ['21450058'], piriformis (470 results) S ['21450058'], case of piriformis (139 results) S ['21450058'], spondylodiscitis (991 results) S ['21450058'], bacterial muscle infection (2308 results) S ['21450058'], melitensis (1874 results) S ['21450058'], brucellosis (10868 results) S ['21450058'], piriformis muscle (157 results) S ['21450058'], piriformis myositis difficult (1 results) S ['21450058'], rare bacterial muscle (243 results) S ['21450058'], abscess formation (3152 results) S ['21450058'], piriformis muscle infection (13 results) D ['15143360', '7743579'], piriformis muscle infection (13 results) S ['21450058'], muscle infection (66 results) S ['21450058'], myositis difficult (274 results) S ['21450058'], rare bacterial muscle infection (175 results) S ['21450058'], myositis (10476 results) S ['21450058'], hip pain (1429 results) S ['21450058'], diagnosis of piriformis (292 results) S ['21450058'], piriformis myositis (1 results) S ['21450058']

PMC3074547	1	FALSE	24	FALSE		2	25	TRUE	21	TRUE	21	TRUE	19	TRU
PMC3077316	8	FALSE	3	FALSE		17	10	TRUE	4	TRUE	3	TRUE	1	TRU
PMC3076246	0	TRUE	18	FALSE		2	13	TRUE	11	TRUE	11	TRUE	11	TRU
PMC3071482	3	FALSE	14	FALSE		8	17	FALSE	17	TRUE	15	TRUE	15	TRU
PMC3078893	12	FALSE	1	FALSE		6	11	TRUE	8	TRUE	6	TRUE	6	TRU

JE	TRUE	12	TRUE	skin compliance (13 results) S ['21447188'], forces during handshake (3 results) S ['21447188'], finger phalanx (17 results) S ['21447188'], solid internal geometry (83 results) S ['21447188'], synthetic finger (510 results) S ['21447188'], synthetic finger phalanges (3 results) S ['21447188'], synthetic skin (81 results) S ['21447188'], middle phalanges (209 results) S ['21447188'], contact forces (487 results) S ['21447188'], different configurations of synthetic (125 results) S ['21447188'], available prosthetic hand (71 results) S ['21447188'], synthetic finger phalanx (5 results) S ['21447188'], available prosthetic (42 results) S ['21447188'], finger phalanx model (59 results) S ['21447188'], phalanx (3361 results) S ['21447188'], finger phalanges (484 results) S ['21447188'], skin materials for prosthetic (159 results) S ['21447188'], skin compliance behaviour (1238 results) S ['21447188'], force-displacement (622 results) S ['21447188'], phalanges (2754 results) S ['21447188'], handshake (103 results) S ['21447188'], prosthetic hand (92 results) D ['20416036'], prosthetic hand (92 results) S ['21447188'], finger phalanx designs (5 results) S ['21447188'], human hand (752 results) S ['21447188']
JE	TRUE	0	FALSE	Schizosaccharomyces pombe checkpoint gene (5 results) D ['11557112', '10232579'], hRad17 (29 results) D ['17657792', '17561505', '15538388', '15078995', '15075340', '14500819', '12578958', '11907025', '11687627', '11606373', '11572977', '11557112', '11555598', '11418864'], hRad17 (29 results) S ['21450056'], pombe checkpoint gene rad17 (3 results) D ['11557112', '10232579'], checkpoint gene rad17 (4 results) D ['11557112', '10232579'], gene rad17 (4 results) D ['11557112', '10232579'], Schizosaccharomyces pombe checkpoint (12 results) D ['11557112', '10232579'], Overexpression of HRad17 (7 results) D ['11557112', '11555598', '11418864', '10208430'], Overexpression of HRad17 (7 results) S ['21450056'], Cancer Res HRad17 (3 results) D ['11606373', '10232579'], upregulation of hRad17 (1 results) S ['21450056']
JE	TRUE	6	TRUE	cells induce CXCL11-dependent migration (1 results) S ['16783574'], organotypic culture (722 results) S ['21450101'], organotypic (3987 results) S ['21450101'], CXCL11-dependent (2 results) S ['16783574'], migration toward tumor (699 results) S ['21450101'], CRC patient towards (93 results) S ['21450101'], induce CXCL11-dependent migration (1 results) S ['16783574'], migration of CTLs (48 results) S ['21450101'], CTLs toward tumor (89 results) S ['21450101'], migration of CTL (166 results) S ['21450101'], CXCR3-expressing (53 results) S ['16783574'], carcinoma cells induce CXCL11-dependent (1 results) S ['16783574'], T-cell migration (656 results) S ['21450101'], induce CXCL11-dependent (1 results) S ['16783574'], T-cell migration toward tumor (70 results) S ['21450101'], cells induce CXCL11-dependent (1 results) S ['16783574'], CCR2 (1921 results) S ['21450101'], CXCL11-dependent migration (2 results) S ['16783574']
JE	TRUE	9	TRUE	filling pressure (2238 results) S ['21490820'], milrinone (1543 results) S ['21490820'], intraoperative haemodynamic changes (7 results) S ['19203992'], subsequent short term outcomes (442 results) S ['21490820'], off-pump (3750 results) S ['21490820'], ventricular filling (3459 results) S ['21490820'], off-pump coronary (2168 results) S ['21490820'], echocardiographic index of ventricular (4061 results) S ['21490820'], hemodynamic variables during OPCAB (25 results) D ['15976197'], hemodynamic variables during OPCAB (25 results) S ['21490820', '15450558'], OPCAB (911 results) S ['21490820'], milrinone versus epinephrine (9 results) D ['16130060', '10698384'], lower through T2-T4 (41 results) S ['21490820'], milrinone without bolus (73 results) D ['20022262', '17602036'], milrinone without bolus (73 results) S ['21490820'], ventricular filling pressure (949 results) S ['21490820'], variables during OPCAB (66 results) S ['21490820']
JE	TRUE	2	TRUE	genotype affects allele-specific levels (33 results) D ['17172560'], GISSI Prevenzione (64 results) D ['17567623'], Pharmacogenetics of apolipoprotein (96 results) D ['18855536'], Harlem-Basset Study (1 results) D ['17172560'], ApoE polymorphism (389 results) D ['21059196'], APOE genotype (1349 results) S ['21450082'], GISSI Prevenzione Investigators Italiano (7 results) D ['11302109'], Prevenzione Investigators Italiano (7 results) D ['11302109'], low-dose pravastatin GISSI (2 results) D ['11302109'], Review Pharmacogenetics of apolipoprotein (49 results) D ['18855536'], Harlem-Basset (1 results) D ['17172560'], low-dose pravastatin GISSI Prevenzione (2 results) D ['11302109'], ApoE genotype affects allele-specific (5 results) D ['17172560']

PMC3078874	7	FALSE	6	FALSE		6	10	TRUE	9	TRUE	8	TRUE	7	TRU
PMC3073884	3	FALSE	22	FALSE		3	25	TRUE	22	TRUE	22	TRUE	21	TRU
PMC3068199	0	TRUE	1	FALSE		1	1	FALSE	1	FALSE	1	FALSE	1	FAL
PMC3068189	3	FALSE	8	FALSE		3	9	TRUE	7	TRUE	7	TRUE	6	TRU
PMC3068188	8	FALSE	7	FALSE		6	12	TRUE	11	TRUE	6	TRUE	5	TRU
PMC3068187	8	FALSE	6	FALSE		5	13	FALSE	13	TRUE	12	TRUE	10	TRU

JE	TRUE	4	TRUE	Artemin activates axonal (1 results) D ['17868192'], growth via SFK (22 results) D ['17868192'], Artemin (141 results) S ['21450093'], ARTN-induced (12 results) D ['17117942', '16914685'], PI-3K (734 results) S ['21450093'], sensory neuronal sensitization (284 results) S ['21450093'], GDNF (2814 results) S ['21450093'], factor family ligands enhance (80 results) S ['19285119'], axonal growth via SFK (1 results) D ['17868192'], Artemin activates (4 results) D ['17868192', '12953054'], mature dorsal root (7 results) D ['17868192'], Artemin activates axonal growth (1 results) D ['17868192'], GFLs (41 results) S ['21450093', '19285119']
JE	TRUE	16	TRUE	mice with p-CPA (32 results) S ['21447193'], CFA-induced (296 results) S ['21447193'], Freund's (10104 results) S ['21447193'], serotonin synthesis inhibitor p-CPA (42 results) D ['10619565'], serotonin synthesis inhibitor p-CPA (42 results) S ['21447193'], days after CFA (403 results) S ['21447193'], CFA injection (172 results) S ['21447193'], synthesis inhibitor p-CPA (3 results) D ['10619565'], hind paw inflammation (65 results) S ['18187350'], inhibitor p-CPA (4 results) D ['10619565'], hind paw (2302 results) S ['21447193'], exogenous 5-HIAA (85 results) S ['21447193'], sciatic nerve days (3793 results) S ['21447193'], p-CPA (181 results) S ['21447193'], 5-HIAA levels (737 results) S ['21447193'], Injection of 5-HIAA (1375 results) S ['21447193'], 5-HIAA (4131 results) S ['21447193'], potentiation of thermal (163 results) S ['21447193'], 5-HTT (832 results) S ['21447193'], inflammatory pain (1814 results) S ['21447193'], thermal hyperalgesia (1428 results) S ['21447193'], p-CPA pretreatment (13 results) S ['21447193'], wild-type mice with p-CPA (2 results) S ['21447193'], role of 5-HIAA (1016 results) S ['21447193'], mice after CFA (417 results) S ['21447193']
.SE	TRUE	1	TRUE	feedback mechanisms (1413 results) S ['21483474']
JE	TRUE	2	TRUE	wing camber (5 results) S ['21479137'], Pteropus wing morphology (160 results) D ['19258691'], tail membrane (30 results) S ['21479137'], leg angle (29 results) S ['21479137'], tail position (15 results) S ['21479137'], leg-induced (1 results) S ['21479137'], bat model (773 results) S ['21479137'], camber (82 results) S ['21479137'], aerofoil measurements (1 results) D ['18165249'], leg position during flight (63 results) S ['21479137'], airframe flight performance (2 results) D ['19258691']
JE	TRUE	2	TRUE	Swedish personal identity possibilities (2 results) D ['19504049'], personal identity possibilities (38 results) D ['19504049'], ski race (52 results) S ['21479136', '12603494'], classical long-distance ski (1 results) S ['12603494'], longitudinal Melbourne Women's Midlife (52 results) D ['16844440'], Swedish National Total Hip (6 results) D ['11061449'], severe knee (224 results) S ['21479136'], hip osteoarthritis (4524 results) S ['21479136'], classical long-distance ski race (1 results) S ['12603494'], Arthroplasty Register valid (8 results) D ['11061449'], Total Hip Arthroplasty Register (6 results) D ['11061449'], linkage scan for athlete (1 results) D ['18179392'], long-distance ski race (12 results) S ['21479136', '12603494'], long-distance ski (10 results) S ['21479136', '12603494'], Hip Arthroplasty Register valid (6 results) D ['11061449']
JE	TRUE	4	TRUE	in-solution hybridization capture (47 results) S ['21479135'], diverse human populations (58 results) D ['20811451'], in-solution target capture (54 results) S ['21479135'], resequencing (1086 results) S ['21479135'], genome variation from population-scale (14 results) D ['20981092'], human genome variation (88 results) D ['20981092'], PCR enrichment for large-scale (26 results) S ['19881494'], rare genetic variation (16 results) D ['20811451'], selection with ultra-long (1 results) D ['19182786'], sequence data from HapMap (244 results) S ['21479135'], population-scale (126 results) D ['20981092'], rare variants (913 results) S ['21479135'], population-scale sequencing (8 results) D ['20981092'], variation from population-scale (26 results) D ['20981092']

PMC3068185	3	FALSE	15	FALSE		9	18	TRUE	13	TRUE	12	TRUE	10	TRU
PMC3068184	10	FALSE	9	FALSE		7	17	TRUE	14	TRUE	13	TRUE	11	TRU
PMC3068182	1	FALSE	11	FALSE		5	11	TRUE	8	TRUE	8	TRUE	6	TRU
PMC3068180	7	FALSE	15	FALSE		5	20	TRUE	15	TRUE	13	TRUE	11	TRU

JE	TRUE	6	TRUE	developments towards clinical application (57 results) S ['21479193'], precision with autoradiography (81 results) D ['16243647'], vivo detection of thrombosis (192 results) S ['21479193'], Conformation-specific blockade (2 results) S ['16778135'], wall-adherent non-occlusive (2 results) S ['21479193'], Reversibility versus persistence (5 results) S ['14617694'], antiplatelet strategy (18 results) S ['16778135'], atherosclerotic resolution with dietary (64 results) D ['19038682'], detection of thrombosis (3876 results) S ['21479193'], von zur Muhlen (220 results) D ['19883911'], von zur Muhlen (220 results) S ['21479193', '18706288', '18574047', '18515970'], platelets allows (706 results) S ['21479193'], non-occlusive thrombosis (10 results) S ['21479193'], detection of wall-adherent (3 results) S ['21479193'], conformation-specific antibodies against glycoprotein (11 results) S ['18515970'], wall-adherent non-occlusive thrombosis (2 results) S ['21479193'], wall-adherent (32 results) S ['21479193'], SPECT-CT (764 results) S ['21479193']
JE	TRUE	8	TRUE	embryonic lung growth (12 results) D ['18367557'], zebrafish reference genes (49 results) D ['17492136'], stimulates embryonic lung growth (101 results) D ['18367557'], swimbladder (338 results) S ['21479192'], lung development (2203 results) S ['21479192'], tissue layers during lung (589 results) S ['21479192', '20682360'], co-receptor implication (7 results) D ['20543981'], acts upstream of N-myc (3 results) D ['15907834'], stimulates embryonic lung (163 results) D ['18367557'], layers during lung (1027 results) S ['21479192'], upstream of N-myc (29 results) D ['15907834'], Korzh (345 results) S ['21479192', '20682360'], chemical inhibitor IWR-1 (1 results) S ['21479192'], Wnt7b stimulates embryonic (2 results) D ['18367557'], Wnt7b stimulates embryonic lung (1 results) D ['18367557'], swimbladder morphogenesis (83 results) S ['19422819'], Wnt7b stimulates (3 results) D ['18367557'], tissue layers (794 results) S ['21479192'], tissue layers of swimbladder (14 results) S ['21479192', '19422819']
JE	TRUE	1	TRUE	Duchenne (7379 results) S ['21479190'], dystrophin-deficient canine (40 results) S ['21479190', '18827405', '18458171', '17140458'], dystrophin-deficient canine Purkinje fibers (1 results) S ['18458171'], Purkinje fibers despite preservation (2 results) S ['18458171'], MicroRNA-206 (14 results) D ['20007902'], MicroRNA-206 (14 results) S ['18827405'], fibers despite preservation (41 results) S ['18458171'], overexpression of Dp71 (2 results) S ['18458171'], expression levels of miR-1 (57 results) S ['21479190'], Selective vacuolar degeneration (61 results) S ['18458171'], muscle-specific miRNA levels (21 results) S ['21479190'], dystrophin-deficient canine Purkinje (2 results) S ['18458171']
JE	TRUE	6	TRUE	crayfish under climate (2 results) S ['21479188'], Review Alien species (86 results) D ['19712994'], B2a emission (2 results) S ['21479188'], swamp crayfish (67 results) S ['21479188'], potential distribution (715 results) S ['21479188'], aquatic invasive (155 results) S ['21479188'], pseudo-absence data (4 results) D ['19323182'], pseudo-absence (5 results) D ['19323182'], crayfish (3586 results) S ['21479188'], notorious global invaders (4 results) S ['21479188'], climatic habitat of non-native (6 results) D ['17594425'], climatic habitat of non-native (6 results) S ['21479188'], invasive species (888 results) S ['21479188'], clarkii (575 results) S ['21479188'], consensus-forecast approach (1 results) S ['21479188'], niche distribution shifts (51 results) S ['21479188'], non-native distribution data (108 results) S ['21479188'], Thuiller (37 results) D ['19712994'], B2a emission scenarios (1 results) S ['21479188'], Sample selection bias (49 results) D ['19323182'], aquatic invasive species (15 results) D ['18577081'], aquatic invasive species (15 results) S ['21479188']

PMC3068179	1	FALSE	23	FALSE		4	22	TRUE	20	TRUE	20	TRUE	20	TRU[illegible]
PMC3068177	3	FALSE	15	FALSE		4	17	TRUE	16	TRUE	14	TRUE	12	TRU[illegible]
PMC3068175	7	FALSE	11	FALSE		6	16	TRUE	10	TRUE	10	TRUE	9	TRU[illegible]
PMC3068174	5	FALSE	5	FALSE		6	8	TRUE	7	TRUE	5	TRUE	1	TRU[illegible]
PMC3068173	0	TRUE	10	FALSE		5	7	FALSE	7	FALSE	7	FALSE	7	TRU[illegible]

JE	TRUE	19	TRUE	multiple endocrine (5926 results) S ['21479187'], uncertain gene variants (269 results) S ['21479187'], RET protooncogene repository (1 results) S ['19177457'], hereditary medullary (125 results) S ['21479187'], proto-oncogene (109762 results) S ['21479187'], neoplasia type (3522 results) S ['21479187'], multiple endocrine neoplasia type (3500 results) S ['21479187'], type RET protooncogene (315 results) S ['19177457'], protooncogene repository (3 results) S ['19177457'], endocrine neoplasia (5647 results) S ['21479187'], computational prediction of missense (33 results) D ['20455263', '20059485'], neoplasia type RET protooncogene (300 results) S ['19177457'], type RET protooncogene repository (1 results) S ['19177457'], multiple endocrine neoplasia (5469 results) S ['21479187'], endocrine neoplasia type (3501 results) S ['21479187'], neoplasia type RET (1198 results) S ['21479187'], RET proto-oncogene (882 results) S ['21479187'], RET sequence variation (3 results) S ['19177457'], medullary thyroid (3561 results) S ['21479187'], curated (1189 results) S ['21479187'], gene variants (2568 results) S ['21479187'], endocrine neoplasia type RET (997 results) S ['21479187'], uncertain gene (4647 results) S ['21479187'], hereditary medullary thyroid (113 results) S ['21479187']
JE	TRUE	7	TRUE	native perennial (190 results) S ['21479185'], Great Basin (144 results) S ['21479185'], remnant native (148 results) S ['21479185'], adaptive value of remnant (5 results) S ['18686583'], trait shifts consistent (18 results) S ['21479185'], native plants (247 results) S ['21479185'], uninvaded (62 results) S ['21479185', '18686583'], shifts consistent with evolution (179 results) S ['21479185'], remnant native plants (38 results) S ['21479185', '18686583'], adult plants (226 results) S ['21479185'], tectorum competition (2 results) S ['21479185', '18686583'], Bromus tectorum (24 results) D ['16827008'], Bromus tectorum (24 results) S ['21479185', '18686583'], tectorum (65 results) S ['21479185', '18686583'], biological invasions alter (19 results) D ['18481527'], native perennial grass (7 results) S ['18686583'], invasions alter natural selection (6 results) D ['18481527'], evolutionary response (208 results) S ['21479185']
JE	TRUE	6	TRUE	Cross-modal individual recognition (2 results) D ['19075246'], attentional state (97 results) S ['21479184'], person's attentional state (4 results) S ['21479184'], Cross-modal (1383 results) S ['21479184'], use of human-given (3 results) D ['19588176'], human attentional states (3 results) D ['16944232'], attentional state of humans (955 results) S ['21479184'], Animals' ability for cross-modal (1 results) S ['21479184'], multisensory perception of humans (718 results) S ['21479184'], domestic horses (67 results) D ['19588176'], experimenter (2485 results) S ['21479184'], attention with multisensory (226 results) S ['21479184'], ability for cross-modal (132 results) S ['21479184'], capuchin monkeys' sensitivity (3 results) D ['16944232'], vocal order (3 results) S ['21479184'], apes' behavior from humans (14 results) D ['15034765'], Comp Psychol (28 results) D ['15088149'], unknown obedience levels (1 results) S ['21479184']
JE	TRUE	0	FALSE	Latitude affects degree (10 results) D ['18604561'], colonial cliff swallows (3 results) D ['19802326'], unequal phenological (1 results) S ['18771506'], reproductive consequences of long-distance (20 results) D ['15612297'], reproductive consequences of long-distance (20 results) S ['16672969'], colonial cliff (11 results) D ['19802326'], unequal phenological changes (1 results) S ['18771506'], affects degree of advancement (18 results) D ['18604561'], density-dependent reproduction (7 results) S ['19341143'], Experimental evidence for density-dependent (59 results) S ['19341143']
JE	TRUE	2	TRUE	mutation s334ter during photoreceptor (22 results) S ['20209167', '12939330'], CNTF (1409 results) S ['21479182'], s334ter during photoreceptor (43 results) S ['20209167'], rhodopsin mutation s334ter (3 results) S ['20209167', '12939330', '10366612'], rod implication (40 results) S ['18188971'], photostasis plasticity (1 results) S ['18188971'], rhodopsin mutation (83 results) S ['20209167'], s334ter (54 results) S ['20209167'], oncostatin (1210 results) S ['21479182'], mutation s334ter (3 results) S ['20209167', '12939330', '10366612']

PMC3068172	8	FALSE	14	FALSE		9	20	TRUE	17	TRUE	15	TRUE	14	TRU
PMC3068171	17	FALSE	17	FALSE		11	29	TRUE	21	TRUE	18	TRUE	15	TRU
PMC3068170	4	FALSE	4	FALSE		3	5	FALSE	5	TRUE	4	TRUE	3	TRU

JE	TRUE	9	TRUE	German draught horses (2 results) S ['17035951'], domestication (1488 results) S ['21479181'], domestic horses (67 results) D ['21187961', '19265018'], domestic horses (67 results) S ['21479181'], microsatellite loci (3696 results) S ['21479181'], Franches-Montagnes breed (6 results) S ['16441293'], German draught (17 results) S ['17035951', '15265065'], horse breeds (139 results) S ['21479181'], diversity among horse (365 results) D ['21187961'], diversity among horse (365 results) S ['21479181'], Genetic diversity among horse (1585 results) D ['21187961'], Genetic diversity among horse (1585 results) S ['21479181'], Spanish Celtic horse (3 results) S ['10690360'], refugia (805 results) S ['21479181'], Spanish Celtic horse breeds (1 results) S ['10690360'], rarefaction approach (47 results) D ['18779233'], Iberian (1453 results) S ['21479181'], mitochondrial DNA lineages (50 results) D ['21187961'], DNA lineages (69 results) D ['21187961'], European cattle breeds highlights (2 results) D ['19659482'], Celtic horse breeds (1 results) S ['10690360'], cattle breeds highlights (7 results) D ['19659482']
JE	TRUE	11	TRUE	bed bug (109 results) S ['21479180', '20828381'], alarm pheromone components (6 results) D ['19496429'], alarm pheromone components improves (1 results) D ['19496429'], Addition of alarm (259 results) S ['21479180'], colony of Cimex (7 results) D ['17427684', '12144303'], airborne aggregation (24 results) D ['18470566'], dusts against Cimex (1 results) D ['19496429'], desiccant dusts against Cimex (1 results) D ['19496429'], common bed bug (16 results) D ['18470566'], common bed bug (16 results) S ['21479180', '20828381', '20032111'], effectiveness of desiccant (4 results) D ['19496429'], bedbugs of interspecific (2 results) D ['2519691'], pheromone components improves (3 results) D ['19496429'], lectularius (168 results) S ['21479180', '20828381'], antennal receptors (18 results) D ['190731'], Cimex (232 results) S ['21479180', '20828381'], compounds of Hemiptera (490 results) S ['21479180'], defensive compounds of Hemiptera (22 results) D ['16124263'], hemipterus (59 results) D ['12510899'], hemipterus (59 results) S ['21479180'], laboratory colony of Cimex (3 results) D ['12144303'], Cimex lectularius (162 results) S ['21479180', '20828381'], Nymphs (3086 results) S ['21479180'], antennal olfactory system (3 results) S ['20032111'], airborne aggregation pheromone (2 results) D ['18470566'], common bed (4307 results) S ['21479180'], defence against conspecific (7 results) S ['20828381'], anti-aphrodisiac defence (1 results) S ['20828381'], alarm pheromone (122 results) S ['20828381'], pheromone (6032 results) S ['21479180'], lectularius bedbugs of interspecific (2 results) D ['2519691'], anti-aphrodisiac defence against conspecific (1 results) S ['20828381'], domestic infestations of Cimex (5 results) D ['2519691'], antennal (1990 results) S ['21479180']
JE	TRUE	3	TRUE	cervical intraepithelial neoplasia (7954 results) S ['21479179'], pregnant Thai implications (7 results) D ['16452834'], Thai implications for future (15 results) D ['16452834'], cervical intraepithelial (8148 results) S ['21479179'], HPV-positive Rwandan (1 results) D ['20976000'], HPV-positive Rwandan women (1 results) D ['20976000'], preventive strategies tests (768 results) S ['21479179'], HIV-HPV co-infection problem (1 results) S ['21479179']

PMC3068169	13	FALSE	17	FALSE		7	27	TRUE	22	TRUE	10	TRUE	10	TRU
PMC3068168	0	TRUE	5	FALSE		1	5	FALSE	5	FALSE	5	FALSE	5	FAL
PMC3068166	6	FALSE	12	FALSE		6	11	TRUE	8	TRUE	7	TRUE	7	TRU
PMC3068165	13	FALSE	16	FALSE		5	27	FALSE	27	TRUE	24	TRUE	18	TRU
PMC3068164	7	FALSE	2	FALSE		3	6	FALSE	6	TRUE	5	TRUE	5	TRU

JE	TRUE	6	TRUE	twin-arginine (455 results) S ['21479178'], subtilis YwbN (6 results) D ['18978042'], subtilis YwbN (6 results) S ['21479178', '18931290', '15554971'], YwbN (6 results) D ['18978042'], YwbN (6 results) S ['21479178', '18931290', '15554971'], Tat-dependent protein secretion (9 results) D ['18978042'], Tat-dependent protein secretion (9 results) S ['21479178', '18931290'], Bacillus subtilis TatAdCd (7 results) D ['18978042'], Bacillus subtilis TatAdCd (7 results) S ['15554971'], different cargo secretion (46 results) S ['18931290'], subtilis Tat (42 results) D ['19682248', '18978042'], subtilis Tat (42 results) S ['21479178', '18931290'], TatAdCd translocase (1 results) D ['18978042'], Bacillus subtilis (25404 results) S ['21479178'], subtilis TatAdCd translocase (1 results) D ['18978042'], Tat-dependent (218 results) S ['21479178'], translocase (2763 results) S ['21479178'], Bacillus subtilis TatAdCd translocase (1 results) D ['18978042'], Tat-dependent protein (212 results) S ['21479178'], Bacillus subtilis YwbN (6 results) D ['18978042'], Bacillus subtilis YwbN (6 results) S ['21479178', '18931290', '15554971'], subtilis Tat pathway (3 results) S ['18931290'], protein secretion (4472 results) S ['21479178'], twin-arginine translocation pathway (46 results) D ['19633084', '17935691'], tatAyCy (7 results) D ['18978042'], tatAyCy (7 results) S ['21479178', '18931290', '15554971'], TatAdCd (7 results) D ['18978042'], TatAdCd (7 results) S ['15554971'], subtilis TatAdCd (7 results) D ['18978042'], subtilis TatAdCd (7 results) S ['15554971']
.SE	TRUE	5	TRUE	caspase inhibitors (1458 results) S ['21479177'], cell cycle regulation (4528 results) S ['21479177'], inhibitor of apoptosis (3945 results) S ['21479177'], caspase-9 (6523 results) S ['21479177'], apoptosis protein (2117 results) S ['21479177']
JE	TRUE	3	TRUE	hyperglycemic (7020 results) S ['21479175'], Bombyx mori Akt (5 results) D ['18760376'], Bombyx mori Akt (5 results) S ['21479175'], larvae-pathogenic agent infection model (1 results) S ['17628142'], silkworm larvae-pathogenic agent infection (1 results) S ['17628142'], silkworms (483 results) S ['21479175'], transport rates through non-specific (47 results) S ['15963696'], bombyxin (75 results) D ['18760376', '18522456'], silkworm larvae-pathogenic (1 results) S ['17628142'], trehalose transporter gene sequence (55 results) D ['20035867'], larvae-pathogenic agent (1 results) S ['17628142'], bombyxin stimulation (7 results) D ['18760376'], trehalose import into peripheral (1 results) D ['20035867'], hyperglycemic silkworms (4 results) S ['21479175'], silkworm larvae-pathogenic agent (1 results) S ['17628142'], import into peripheral (164 results) D ['20035867'], mori Akt (98 results) S ['21479175'], larvae-pathogenic agent infection (1 results) S ['17628142']
JE	TRUE	10	TRUE	protease matriptase regulates (18 results) D ['20142489'], ARPE-19 cells (407 results) S ['21479174'], retinal pigment (10414 results) S ['21479174'], ARPE-19 monolayer barrier (12 results) S ['21479174'], protease matriptase regulates epithelial (8 results) D ['20142489'], cytokines stimulate CCL20 (25 results) D ['19295614'], IL-17A (609 results) S ['21479174'], diffusion rate of FITC-dextran (33 results) S ['21479174'], matriptase regulates (18 results) D ['20142489'], serine protease matriptase regulates (17 results) D ['20142489'], stimulate CCL20 expression (19 results) D ['19295614'], cytokines stimulate CCL20 expression (19 results) D ['19295614'], IL-22 (530 results) S ['21479174'], IL-22R (37 results) D ['19731362'], IL-22R (37 results) S ['21479174'], ARPE-19 monolayer (26 results) S ['21479174'], ARPE-19 (518 results) S ['21479174'], CCL20 (646 results) S ['21479174'], IL-17F (234 results) S ['21479174'], IL-17RC (32 results) D ['19234208'], IL-17RC (32 results) S ['21479174'], IL-17RA (73 results) S ['21479174'], Th17 cytokines stimulate CCL20 (1 results) D ['19295614'], ARPE-19 monolayer barrier function (9 results) S ['21479174'], occludin (1749 results) S ['21479174'], matriptase regulates epithelial barrier (1 results) D ['20142489'], rate of FITC-dextran (224 results) D ['20142489'], rate of FITC-dextran (224 results) S ['21479174'], matriptase regulates epithelial (8 results) D ['20142489']
JE	TRUE	2	TRUE	subsocial spider (10 results) D ['19860868'], tentoriicola (1 results) D ['19860868'], Relatedness facilitates (24 results) D ['19860868'], Relatedness facilitates cooperation (1 results) D ['19860868'], Stegodyphus tentoriicola (1 results) D ['19860868'], interaction networks facilitates (69 results) D ['19443505'], primate interaction networks facilitates (30 results) D ['19443505'], social relationships on cooperation (250 results) S ['21479173'], Social structure (1171 results) S ['21479173']

PMC3068163	1	FALSE	3	FALSE		3	4	TRUE	2	TRUE	1	TRUE	1	TRU
PMC3068160	3	FALSE	13	FALSE		4	15	TRUE	14	TRUE	12	TRUE	7	TRU
PMC3068159	21	FALSE	10	FALSE		10	23	TRUE	13	TRUE	10	TRUE	8	TRU
PMC3068158	16	FALSE	0	TRUE		6	15	TRUE	12	TRUE	8	TRUE	3	TRU

JE	TRUE	0	FALSE	abrogates invasive growth (37 results) S ['18323781'], 6th international melanoma congress (3 results) S ['20025711'], 6th international melanoma (3 results) S ['20025711'], K642E KIT mutation (6 results) D ['18510589']
JE	TRUE	4	TRUE	cumulative culture (13 results) S ['21479170'], Variable Cultural Acquisition Costs (1 results) S ['21479170'], cultural acquisition costs (39 results) S ['21479170'], cultural acquisition (3038 results) S ['21479170'], different cultural transmission rules (12 results) S ['21479170'], cultural evolution (749 results) S ['21479170'], cultural transmission (233 results) S ['21479170'], cumulative cultural evolution (9 results) S ['21479170'], Mesoudi (11 results) S ['21479170', '15058714'], cultural complexity (13 results) D ['19498164'], cultural complexity (13 results) S ['21479170'], culture increase (20 results) D ['18571686'], acquisition costs (554 results) S ['21479170'], variable cultural acquisition (49 results) S ['21479170'], human culture increase exponentially (97 results) D ['18571686'], cultural complexity accumulates (3 results) S ['21479170']
JE	TRUE	3	TRUE	existential threat (23 results) S ['21479169'], threat increases death-thought (2 results) D ['17484605'], evaluations of charismatic (7 results) D ['15563330'], mitigates worldview (1 results) D ['16938037'], Windows into Terror (1 results) D ['16784340'], worldview threat (5 results) D ['17484605'], worldview threat (5 results) S ['15982118'], support for IDT (143 results) S ['21479169'], existential (1962 results) S ['21479169'], acceptance of IDT (3 results) S ['21479169'], worldview threat increases (3 results) D ['17484605', '10560330'], mortality salience on worldview (33 results) D ['19234297', '18729692', '16938037'], mortality salience on worldview (33 results) S ['15982118'], worldview threat increases death-thought (2 results) D ['17484605'], mitigates worldview defense (1 results) D ['16938037'], increases death-thought (4 results) D ['17484605'], salience on worldview (36 results) D ['19234297', '18729692', '16938037'], salience on worldview (36 results) S ['15982118'], religiousness mitigates worldview (1 results) D ['16938037'], increases death-thought accessibility (4 results) D ['17484605'], IDT (359 results) S ['21479169'], death-thought accessibility (12 results) D ['19234297', '17484605', '16938037'], threat increases death-thought accessibility (2 results) D ['17484605'], religiousness mitigates worldview defense (1 results) D ['16938037'], intrinsic religiousness mitigates worldview (1 results) D ['16938037'], worldview defense (18 results) D ['18729692', '16938037', '12930486'], worldview defense (18 results) S ['15982118'], mortality salience on evaluations (10 results) D ['15563330'], religiousness mitigates (1 results) D ['16938037'], intrinsic religiousness mitigates (1 results) D ['16938037'], tripartite security system (3 results) S ['15982118']
JE	TRUE	0	FALSE	fetal neural dopamine-rich grafts (13 results) D ['15157925', '2753100'], neural dopamine-rich grafts (18 results) D ['15157925', '2753100'], necrosis factor-alpha impairs neuronal (13 results) D ['19840854'], syngeneic fetal dopamine-rich (5 results) D ['8162272', '2753100'], syngeneic fetal dopamine-rich neuronal (2 results) D ['8162272'], allogeneic mesencephalic tissue (25 results) D ['7715776'], precursor Role of Hes1 (47 results) D ['19840854'], factor-alpha impairs neuronal (13 results) D ['19840854'], fetal neural dopamine-rich (13 results) D ['15157925', '2753100'], neural dopamine-rich (30 results) D ['15157925'], fetal dopamine-rich neuronal (7 results) D ['8162272'], Sequential intracerebral transplantation (7 results) D ['8162272'], fetal dopamine-rich neuronal tissue (6 results) D ['8162272'], allogeneic fetal neural dopamine-rich (5 results) D ['2753100'], factor-alpha impairs neuronal differentiation (1 results) D ['19840854'], Neural cells express costimulatory (8 results) D ['15111308']

PMC3068155	5	FALSE	12	FALSE		11	16	TRUE	11	TRUE	11	TRUE	9	TRU
PMC3068154	2	FALSE	2	FALSE		2	4	TRUE	3	TRUE	2	TRUE	0	TRU
PMC3068153	3	FALSE	2	FALSE		4	5	FALSE	5	TRUE	2	TRUE	2	TRU
PMC3068152	0	TRUE	11	FALSE		2	11	TRUE	10	TRUE	8	TRUE	7	TRU
PMC3068151	16	FALSE	6	FALSE		7	19	TRUE	13	TRUE	11	TRUE	8	TRU
PMC3068150	1	FALSE	13	FALSE		8	11	FALSE	11	TRUE	8	TRUE	6	TRU

JE	TRUE	4	TRUE	total RBP4 (63 results) S ['21479165'], RBP4 MSIA (1 results) S ['21479165'], retinol-binding (4606 results) S ['21479165'], RBP4 measurements (20 results) D ['20156079', '16775236'], RBP4 variants (2 results) S ['21479165'], high-throughput mass spectrometric immunoassay (3 results) S ['11811966', '11476228'], RBP4 (346 results) S ['21479165'], mass spectrometric immunoassay system (3 results) S ['11811966', '11476228'], situ digestion liquid chromatography-tandem (3 results) D ['20007860'], spectrometric immunoassay system (3 results) S ['11811966', '11476228'], situ digestion liquid (117 results) D ['20007860'], spectrometric immunoassay (27 results) D ['20022981'], spectrometric immunoassay (27 results) S ['21479165', '16823976'], MSIA (19 results) S ['21479165', '12562854', '12237133', '11476228', '11161291'], retinol (11763 results) S ['21479165'], mass spectrometric immunoassay (27 results) D ['20022981'], mass spectrometric immunoassay (27 results) S ['21479165', '16823976']
JE	FALSE	0	FALSE	TNF-alpha regulate autophagy (4 results) D ['16942488'], modify early oxidative events (13 results) S ['16020663'], regulate autophagy through c-jun (6 results) D ['16942488'], synthase modify early oxidative (11 results) S ['16020663']
JE	TRUE	1	TRUE	large north American population (7 results) D ['19783586'], Western strategic design (38 results) D ['18980573'], sociodemographic factors of ASD (6 results) S ['21479223'], Caucasian singleton (6 results) D ['15910694'], paternal age (1202 results) S ['21479223']
JE	TRUE	6	TRUE	diagnostic methods for CFS (617 results) S ['21479222'], Gene expression subtypes (6 results) S ['18462164'], new population of CFS (23 results) S ['21479222'], expression subtypes (10 results) S ['18462164'], reporter genes (27568 results) S ['21479222'], broad CFS diagnostic (17 results) S ['21479222'], chronic fatigue (4264 results) S ['21479222'], fatigue syndrome (4554 results) S ['21479222'], chronic fatigue syndrome (4406 results) S ['21479222'], mRNA relative quantities (268 results) S ['21479222'], classifier of CFS (5 results) S ['21479222']
JE	TRUE	5	TRUE	LCA (2723 results) S ['21479221'], class ephrin-A5 binds (1 results) D ['15107857'], Eph receptors (399 results) S ['21479221'], Eph (2010 results) S ['21479221'], transglutaminase clusters (25 results) D ['17707797'], Tissue transglutaminase clusters (13 results) D ['17707797'], A-class Eph crystal (3 results) D ['19525919'], clusters soluble A-type ephrins (1 results) D ['17707797'], EphA2 kinase suppresses integrin (2 results) D ['10655584'], class ephrin-A5 (12 results) D ['15107857'], EphA2 (352 results) S ['21479221'], Tissue transglutaminase clusters soluble (2 results) D ['17707797'], Eph crystal structures (13 results) D ['19525919'], transglutaminase clusters soluble A-type (1 results) D ['17707797'], clusters soluble A-type (5 results) D ['17707797'], ephrin (1539 results) S ['21479221'], enantiomeric bile (31 results) D ['19054763', '17963371'], Activation of EphA2 (96 results) S ['21479221'], A-class Eph crystal structures (2 results) D ['19525919'], transglutaminase clusters soluble (3 results) D ['17707797'], EphA2 kinase (8 results) D ['10655584'], EphA2 kinase suppresses (10 results) D ['10655584']
JE	TRUE	3	TRUE	Akt-dependent proapoptotic (24 results) S ['19047102'], tensin sclerosis complex 2-deficient (1 results) S ['19047102'], 2-deficient mouse astrocytoma (1 results) S ['19047102'], complex 2-deficient mouse astrocytoma (1 results) S ['19047102'], sclerosis complex 2-deficient mouse (6 results) S ['19047102'], tensin sclerosis (28 results) S ['19047102'], complex 2-deficient mouse (105 results) S ['19047102'], sclerosis complex 2-deficient (6 results) S ['19047102'], astrocytoma (14972 results) S ['21479220'], restriction on late-stage (111 results) S ['19047102'], Akt-dependent proapoptotic effects (18 results) S ['19047102'], mouse astrocytoma (26 results) D ['17701911'], mouse astrocytoma (26 results) S ['21479220', '19047102', '18474106', '17313687', '15328205', '14520474', '12085212'], dietary restriction on late-stage (6 results) S ['19047102']

PMC3068149	3	FALSE	16	FALSE		3	19	TRUE	17	TRUE	16	TRUE	14	TRU
PMC3068148	2	FALSE	4	FALSE		4	6	TRUE	5	TRUE	5	TRUE	5	TRU
PMC3068147	1	FALSE	15	FALSE		5	14	TRUE	11	TRUE	10	TRUE	8	TRU
PMC3068145	2	FALSE	15	FALSE		5	17	FALSE	17	TRUE	16	TRUE	15	TRU
PMC3068144	0	TRUE	5	FALSE		2	5	TRUE	3	TRUE	3	TRUE	3	TRU
PMC3068142	4	FALSE	9	FALSE		4	13	TRUE	10	TRUE	10	TRUE	9	TRU

JE	TRUE	11	TRUE	severe bilateral hippocampal (6 results) D ['12791331'], volume change (1982 results) S ['21479219'], born-again (59 results) S ['21479219'], final region volume (626 results) S ['21479219'], hippocampal volume (1184 results) S ['21479219'], hippocampal atrophy (814 results) S ['21479219'], volume change between baseline (4647 results) S ['21479219'], baseline cerebral volume (2174 results) S ['21479219'], religious factors (131 results) S ['21479219'], baseline region volume (1418 results) S ['21479219'], religious experiences (70 results) S ['21479219'], late life religious experiences (4 results) S ['21479219'], cerebral volume (314 results) S ['21479219'], cardiac mediation of coping (23 results) D ['20207662'], late life (2964 results) S ['21479219'], Greater hippocampal atrophy (11 results) S ['21479219'], severe bilateral hippocampal atrophy (2 results) D ['12791331'], hippocampal volume change (16 results) S ['21479219'], greater hippocampal (5350 results) S ['21479219']
JE	TRUE	4	TRUE	Brain Tumor Heterogeneity (5 results) D ['20047002'], Intratumor (1169 results) S ['21479218'], Intratumor Heterogeneity (154 results) S ['21479218', '20101094'], tumor mechanism behind heterogeneity (31 results) D ['16144692'], dynamics of heterogeneity (49797 results) S ['21479218'], Tumor heterogeneity (594 results) S ['21479218']
JE	TRUE	3	TRUE	Primacy of hepatic (13 results) S ['14514627'], additional accumulation of visceral (58 results) S ['19366874'], Nocturnal free fatty acids (88 results) S ['17264230'], isocaloric moderate-fat diet (5 results) S ['14514627'], isocaloric moderate-fat (5 results) S ['14514627'], Nocturnal free fatty (58 results) D ['12606508'], Nocturnal free fatty (58 results) S ['17264230'], hyperinsulinemic compensation (5 results) S ['21479217'], Hypercaloric high fat feeding (13 results) S ['21479217', '19366874', '17264230'], complete hyperinsulinemic (78 results) S ['21479217'], prevents additional accumulation (120 results) S ['19366874'], non-adipose tissue size (9 results) S ['21479217'], complete hyperinsulinemic compensation (1 results) S ['21479217'], hepatic insulin (1481 results) S ['21479217'], Rimonabant prevents additional accumulation (1 results) S ['19366874'], FFA (6349 results) S ['21479217']
JE	TRUE	12	TRUE	free-ranging (2866 results) S ['21479215'], Urine C-peptide (40 results) D ['17011368'], UCP levels (14 results) S ['21479215'], plasma C-peptide (644 results) S ['21479215'], UCPs (366 results) S ['21479215'], fatness (2586 results) S ['21479215'], plasma C-peptide levels (143 results) S ['21479215'], non-invasive tool (582 results) S ['21479215'], reliable biomarkers of nutritional (89 results) S ['21479215'], UCP measurement (30 results) S ['21479215'], C-peptide levels (1476 results) S ['21479215'], urinary C-peptide (215 results) D ['19084530', '18638479', '18255067'], urinary C-peptide (215 results) S ['21479215'], C-peptide (10048 results) S ['21479215'], measurement of UCPs (6 results) S ['21479215'], skinfold fatness (502 results) S ['21479215'], UCP (1326 results) S ['21479215']
JE	TRUE	3	TRUE	insect species considered (1621 results) S ['21479214'], macroecological perspective (2 results) S ['20015316'], body size frequency distributions (5 results) S ['21479214', '20015316'], Review Body size variation (661 results) S ['21479214'], Review Body size (29754 results) S ['21479214']
JE	TRUE	6	TRUE	Protein structural conformation (5 results) D ['16715374'], virial coefficient relates (2 results) D ['16715374'], virial (796 results) S ['21479212'], favorable crystallization temperature (51 results) S ['21479212'], crystallization temperature (186 results) S ['21479212'], favorable crystallization (279 results) S ['21479212'], crystallization screens (32 results) S ['21479212'], osmotic virial coefficient (12 results) D ['18395000'], protein crystallization (522 results) S ['21479212'], virial coefficient (328 results) S ['21479212'], virial coefficient for protein (176 results) S ['21479212'], protein crystal practical approach (24 results) D ['12718919'], protein crystallization screens (5 results) S ['21479212']

PMC3068141	3	FALSE	19	FALSE		4	22	TRUE	19	TRUE	16	TRUE	16	[illegible]
PMC3068139	6	FALSE	4	FALSE		6	10	FALSE	10	FALSE	10	FALSE	10	[illegible]
PMC3068138	7	FALSE	7	FALSE		3	12	TRUE	7	TRUE	6	TRUE	4	[illegible]
PMC3068137	13	FALSE	8	FALSE		10	19	TRUE	15	TRUE	12	TRUE	11	[illegible]
PMC3068136	0	TRUE	11	FALSE		1	10	TRUE	8	TRUE	6	TRUE	5	[illegible]

JE	TRUE	8	TRUE	Drosophila melanogaster taught (6 results) D ['14631102'], ladybird (191 results) S ['21479211'], food conditions (115 results) S ['21479211'], larval food stress reduces (3 results) S ['21479211'], growth on lifetime (2291 results) S ['21479211'], high larval nutrition (76 results) S ['21479211'], compensatory growth on lifetime (5 results) S ['17584225'], adult food environments (7086 results) S ['21479211'], larval nutrition (60 results) S ['21479211'], larval food (113 results) S ['21479211'], food stress (35 results) D ['16869432'], food stress (35 results) S ['21479211'], high food adults environments (1882 results) S ['21479211'], ladybird beetle (46 results) S ['17584225'], melanogaster taught (6 results) D ['14631102'], food adults environments (7664 results) S ['21479211'], Dmitriew (10 results) S ['21479211', '17584225'], Larval food stress (93 results) S ['21479211'], reduces lifetime fitness regardless (2 results) S ['21479211'], lifetime fitness (86 results) S ['21479211'], early resource limitation (52 results) S ['17584225'], adult phenotype (249 results) S ['21479211']
JE	TRUE	6	TRUE	complaints after axillary (66 results) D ['19903919'], termination among employees (128 results) D ['18032531'], tumour stage on sickness (112 results) D ['18670868'], tumour stage on sickness (112 results) S ['21479209'], possible influence of tumour (4997 results) S ['21479209'], job termination among employees (26 results) D ['18032531'], education year prior (2706 results) S ['21479209'], breast cancer on sickness (178 results) S ['21479209'], Danish cohort study (61 results) D ['18640029'], arm complaints after axillary (22 results) D ['12915872']
JE	TRUE	3	TRUE	CDR-H3 (110 results) D ['20147404'], CDR-H3 (110 results) S ['21479208'], CDR-H3 length (5 results) D ['14636599'], CDR-H3 length (5 results) S ['21479208'], human CDR-H3 intervals (1 results) D ['14636599'], equal length exhibit distinct (7 results) D ['14636599'], SAD MAbs (5 results) S ['21479208'], length exhibit distinct repertoires (3 results) D ['14636599'], CDR-H3 intervals (2 results) D ['14636599'], CDR-H3s (11 results) S ['21479208'], Abs from acute (437 results) S ['21479208'], long CDR-H3s (1 results) S ['21479208'], human CDR-H3 (54 results) S ['21479208'], exhibit distinct repertoires (17 results) D ['14636599']
JE	TRUE	8	TRUE	Progerin elicits disease (3 results) D ['18769635'], zebrafish requires sterol (1 results) D ['19127516'], requires sterol regulatory element (119 results) D ['19127516'], progerin (56 results) S ['21479207'], ZMPSTE24 defects (49 results) D ['20458013', '18587406'], progeria syndrome (246 results) S ['21479207'], Hutchinson-Gilford (333 results) S ['21479207'], Hutchinson-Gilford progeria syndrome (219 results) S ['21479207'], zebrafish lamin (9 results) D ['11893082'], zebrafish lamin (9 results) S ['21479207'], Hutchinson-Gilford progeria syndrome mutation (3 results) D ['16862216', '16014412'], zebrafish requires sterol regulatory (1 results) D ['19127516'], requires sterol regulatory (144 results) D ['19127516'], lamin (2487 results) S ['21479207'], protein farnesyltransferase improves (8 results) D ['16862216', '16129834', '16014412'], progeria syndrome mutation (3 results) D ['16862216', '16014412'], Progerin elicits (3 results) D ['18769635'], farnesyltransferase improves (18 results) D ['18587406', '16862216', '16129834', '16014412'], nuclear blebbing (22 results) D ['16129833', '16014412'], progeria (1224 results) S ['21479207'], Hutchinson-Gilford progeria (275 results) S ['21479207']
JE	TRUE	4	TRUE	population encounters unfamiliar conditions (1 results) S ['21479206'], activity patterns (3740 results) S ['21479206'], patterns during emergencies (484 results) S ['21479206'], population encounters unfamiliar (8 results) S ['21479206'], human activity under extreme (301 results) S ['21479206'], quantitative view of behavioral (411 results) S ['21479206'], stationary human activity patterns (70 results) S ['21479206'], significant for non-emergencies (3 results) S ['21479206'], emergencies spreads globally (2 results) S ['21479206'], non-emergencies (13 results) S ['21479206'], social network of eyewitnesses (10 results) S ['21479206']

PMC3068135	11	FALSE	2	FALSE		7	9	TRUE	6	TRUE	3	TRUE	2	TRU
PMC3068134	1	FALSE	5	FALSE		4	6	FALSE	6	FALSE	6	FALSE	6	TRU
PMC3068132	3	FALSE	13	FALSE		5	16	TRUE	15	TRUE	14	TRUE	13	TRU
PMC3070446	5	FALSE	6	FALSE		2	11	FALSE	11	FALSE	11	TRUE	7	TRU
PMC3072955	5	FALSE	7	FALSE		5	12	TRUE	11	TRUE	6	TRUE	6	TRU
PMC3077322	8	FALSE	16	FALSE		3	22	TRUE	16	TRUE	13	TRUE	9	TRU
PMC3076250	0	TRUE	4	FALSE		1	4	FALSE	4	FALSE	4	FALSE	4	TRU

JE	TRUE	2	TRUE	intestinal anion secretion (11 results) D ['17584847'], chloride secretion (1000 results) S ['21479205'], Phytochemical reinvestigation of Xysmalobium (1 results) D ['17252392'], Uzara (6 results) D ['15176651', '17252392'], Uzara (6 results) S ['21479205'], Xysmalobium undulatum roots (1 results) D ['17252392'], Digitalis structure-activity relationship analyses (4 results) D ['6313008'], Abolition of intestinal (133 results) D ['17584847'], undulatum roots (9 results) D ['17252392'], uzara glycosides (1 results) D ['15176651'], multiple membrane ATPases (2 results) D ['8298181'], microbial paradigms for microbial-mucosal (7 results) D ['11292584'], reinvestigation of Xysmalobium (1 results) D ['17252392']
JE	TRUE	3	TRUE	Students' moral reasoning (34 results) D ['12668541', '9609863'], Students' moral reasoning (34 results) S ['16483330'], Personal Interest score (66 results) S ['21479204'], moral reasoning (450 results) S ['21479204'], hidden medical curriculum (269 results) S ['21479204'], Personal Interest scores (109 results) S ['21479204']
JE	TRUE	6	TRUE	subsequent NIV treatment (22 results) S ['21479202'], good compliance with subsequent (167 results) S ['21479202'], Noninvasive ventilation (934 results) S ['21479202'], efficacy of capnography (42 results) S ['21479202'], need for NIV (131 results) S ['21479202'], amyotrophic lateral sclerosis suffer (37 results) S ['21479202'], supine capnography (16 results) S ['21479202'], Sleep characteristics of amyotrophic (6 results) D ['17453638'], capnography (1270 results) S ['21479202'], patients with amyotrophic (5411 results) S ['21479202'], compliance with subsequent (2231 results) S ['21479202'], NIV treatment (21 results) S ['21479202'], subsequent NIV (34 results) S ['21479202'], volume ventilators for amyotrophic (4 results) D ['17167024'], lateral sclerosis suffer (67 results) S ['21479202'], amyotrophic lateral clinical guidelines (56 results) D ['17884681', '17653917']
JE	TRUE	5	TRUE	osteoarthropathy (2716 results) S ['21464881'], chronic suppurative lung processes (71 results) D ['8413003'], malignant HOA symptoms (13 results) D ['8413003'], suppurative lung processes cystic (12 results) D ['8413003'], cystic congenital heart disease (264 results) S ['21464881'], Hypertrophic osteoarthropathy (735 results) S ['21464881'], periosteal (5851 results) S ['21464881'], cases of HOA (81 results) S ['21464881'], cystic congenital heart (651 results) S ['21464881'], malignant HOA (14 results) D ['8413003'], lung processes cystic congenital (12 results) D ['8413003']
JE	TRUE	3	TRUE	receptor for RAGE (1498 results) S ['21450080'], pulmonary GOLD executive (8 results) D ['17507545'], correlation between sRAGE (21 results) D ['20595148'], S100A12 (199 results) S ['21450080'], pulmonary GOLD executive summary (4 results) D ['17507545'], sRAGE (226 results) S ['21450080'], obstructive pulmonary GOLD executive (6 results) D ['17507545'], RAGE ligands (61 results) S ['21450080'], plasma concentration of sRAGE (10 results) D ['15731496'], plasma concentration of sRAGE (10 results) S ['16926247'], S100A12 between cases (9 results) S ['21450080'], decoy receptor for RAGE (61 results) S ['21450080']
JE	TRUE	6	TRUE	lateral midpatellar portal (2 results) D ['12208907'], lateral midpatellar portal (2 results) S ['21447197'], medial femoral condyle (711 results) S ['21447197'], different intra-articular injection sites (15 results) D ['17149645'], midpatellar approach (2 results) D ['12208907'], intraarticular accuracy (93 results) S ['21447197'], lateral midpatellar approach (1 results) D ['12208907'], medial midpatellar (9 results) D ['17149645'], medial midpatellar (9 results) S ['21447197'], intraarticular injection accuracy (125 results) S ['21447197'], intraarticular injection (565 results) S ['21447197'], intraarticular (3794 results) S ['21447197'], lateral midpatellar (11 results) D ['17149645', '12208907'], lateral midpatellar (11 results) S ['21447197'], portal for intraarticular (27 results) S ['21447197'], midpatellar (20 results) D ['17149645', '12208907'], midpatellar (20 results) S ['21447197'], standard lateral midpatellar (2 results) S ['21447197'], anteriolateral (32 results) S ['21447197'], midpatellar portal (5 results) D ['12208907'], midpatellar portal (5 results) S ['21447197'], anteriolateral portal (1 results) S ['21447197'], medial femoral (1172 results) S ['21447197'], femoral condyle (1940 results) S ['21447197']
JE	TRUE	3	TRUE	FACT-B (81 results) S ['21450089'], larger future trial (968 results) S ['21450089'], future trial (131 results) S ['21450089'], enable people with cancer (102 results) S ['21450089']

PMC3074533	8	FALSE	14	FALSE		8	22	TRUE	20	TRUE	20	TRUE	15	TRU
PMC3061985	4	FALSE	23	FALSE		7	22	TRUE	19	TRUE	16	TRUE	11	TRU
PMC3072138	7	FALSE	8	FALSE		3	11	TRUE	9	TRUE	8	TRUE	7	TRU
PMC3072137	0	TRUE	6	FALSE		1	6	FALSE	6	FALSE	6	FALSE	6	FAL
PMC3072322	0	TRUE	18	FALSE		2	18	TRUE	13	TRUE	12	TRUE	12	TRU

JE	TRUE	10	TRUE	CAV sequences (158 results) S ['21447189'], anaemia sequence analysis (3063 results) S ['21447189'], Chinese viral sequences (2216 results) S ['21447189'], anemia virus (1102 results) S ['21447189'], VP1 (3653 results) S ['21447189'], CAV strains (12 results) D ['18459299', '8844608'], chicken anaemia sequence analysis (67 results) D ['18459299'], chicken anaemia sequence analysis (67 results) S ['21447189'], VP3 (1377 results) S ['21447189'], transfection capabilities (18 results) D ['1605740'], Chinese isolates (146 results) S ['21447189'], Malaysian Chicken anaemia (2 results) D ['14648297'], Chinese CAV (62 results) S ['21447189'], Malaysian Chicken anaemia virus (2 results) D ['14648297'], chicken anaemia sequence (146 results) S ['21447189'], anaemia sequence (5316 results) S ['21447189'], CAV genome (15 results) D ['16384622', '18670920'], CAV isolates (15 results) D ['16384622'], CAV isolates (15 results) S ['21447189'], VP2 (2116 results) S ['21447189'], chicken infectious anemia (45 results) D ['20952706'], chicken infectious anemia (45 results) S ['21447189']
JE	TRUE	1	TRUE	mRNA increases transposition rate (1 results) S ['16043504'], increases transposition rate (44 results) S ['16043504'], approach allows inducible (109 results) S ['20805932'], transposition rate (60 results) S ['16043504'], synthetic biology approach allows (82 results) S ['20805932'], Tto1 mRNA increases transposition (1 results) S ['16043504'], retrotransposon Tto1 mRNA (3 results) S ['21262516', '16043504'], retrotransposon Tto1 (14 results) D ['10929123', '8624443'], retrotransposon Tto1 (14 results) S ['21262516', '20805932', '18191436', '16043504'], plant retrotransposon (11 results) D ['8389699'], plant retrotransposon (11 results) S ['21262516', '18191436'], Syst Synth Biol (58 results) S ['20805932'], approach allows inducible retrotransposition (1 results) S ['20805932'], plant retrotransposon Tto1 (20 results) D ['10929123', '8624443', '8389699'], plant retrotransposon Tto1 (20 results) S ['21262516', '20805932', '18191436', '16043504'], Tto1 mRNA increases (1 results) S ['16043504'], retrotransposon Tto1 mRNA increases (1 results) S ['16043504'], Tto1 (27 results) D ['10929123'], Tto1 (27 results) S ['21262516', '20805932', '18191436', '16043504'], mRNA increases transposition (10 results) S ['16043504'], inducible retrotransposition (10 results) S ['20805932'], leader of retrotransposon (39 results) S ['16043504'], Tto1 mRNA (3 results) S ['21262516', '16043504'], synthetic biology approach (22 results) S ['20805932'], allows inducible retrotransposition (3 results) S ['20805932'], Unorthodox mRNA (23 results) S ['16043504'], biology approach allows inducible (30 results) S ['20805932']
JE	TRUE	4	TRUE	SpO2 camera technology (4 results) D ['16133912'], step toward SpO2 (1 results) D ['16133912'], synchronous detection (69 results) S ['21483621'], multiple wavelength photoplethysmographic (1 results) D ['16133912'], video frames (189 results) S ['21483621'], Contactless multiple wavelength photoplethysmographic (1 results) D ['16133912'], lock-in amplification (15 results) S ['21483621'], pulse waves (269 results) S ['21483621'], SpO2 camera (5 results) D ['16133912'], synchronous detection of cardiovascular (44 results) S ['21483621'], reference function (80 results) S ['21483621'], wavelength photoplethysmographic (10 results) D ['17477684', '16133912'], Contactless multiple wavelength (1 results) D ['16133912'], cardiovascular pulse waves (1344 results) S ['21483621'], photoplethysmographic (420 results) S ['21483621']
.SE	TRUE	6	TRUE	Hilbert (1477 results) S ['21483620'], off-axis (1631 results) S ['21483620'], interferometry (5273 results) S ['21483620'], retrieval algorithm (125 results) S ['21483620'], fine spatial (2366 results) S ['21483620'], FFT (1532 results) S ['21483620']
JE	TRUE	8	TRUE	III degradation fragment (4140 results) S ['21447148'], degradation fragment CO3-610 (3 results) S ['21447148', '20380828'], neo-epitope of type (24 results) S ['21447148', '20380828'], skin fibrosis progression (277 results) S ['21447148'], collagen III (812 results) S ['21447148'], potential positive biomarker (16689 results) S ['21447148'], collagen III degradation (8511 results) S ['21447148'], CO3-610 levels (3 results) S ['21447148', '20380828'], III degradation fragment CO3-610 (3 results) S ['21447148', '20380828'], fragment CO3-610 (3 results) S ['21447148', '20380828'], skin fibrosis (237 results) S ['21447148'], Barascuk (8 results) S ['21447148', '20380828'], creatinine corrections (42 results) S ['21447148'], bleomycin-induced (1703 results) S ['21447148'], degradation fragment (59 results) S ['21447148'], CO3-610 (3 results) S ['21447148', '20380828'], III degradation (32 results) S ['21447148'], collagen III degradation fragment (198 results) S ['21447148', '20380828']

PMC3076236	1	FALSE	6	FALSE		2	7	FALSE	7	TRUE	5	TRUE	4	TRU
PMC3078887	9	FALSE	15	FALSE		18	20	TRUE	16	TRUE	16	TRUE	10	TRU
PMC3078845	0	TRUE	14	FALSE		1	14	TRUE	13	TRUE	13	TRUE	13	TRU
PMC3072959	1	FALSE	19	FALSE		3	20	TRUE	14	TRUE	12	TRUE	12	TRU
PMC3073916	4	FALSE	4	FALSE		8	8	TRUE	6	TRUE	6	TRUE	4	TRU
PMC3073886	0	TRUE	0	TRUE		0	0	FALSE	0	FALSE	0	FALSE	0	FAL

JE	TRUE	2	TRUE	different prehospital (454 results) S ['21447161'], Acute Myocardial Executive Summary (12 results) D ['10468535'], data guardians (610 results) S ['21447161'], guardian abstraction (6 results) S ['21447161'], different prehospital treatment strategies (24 results) S ['21447161'], data guardian abstraction (6 results) S ['21447161'], PCI centre (26 results) S ['21447161']
JE	TRUE	4	TRUE	A180 upregulation (1 results) S ['21447186'], key functional sites (11 results) S ['18638376'], wave-sensitive (80 results) D ['20353595', '18598396', '18590925', '17015333'], wave-sensitive (80 results) S ['18638376'], opsin (2450 results) S ['21447186'], large visual-opsin gene repertoire (1 results) S ['21447186'], long wave-sensitive (33 results) D ['20353595', '18598396', '17015333'], long wave-sensitive (33 results) S ['18638376'], guppy cone (13 results) D ['21170644', '1843763', '2309457', '3424671', '6686498', '6850777', '7202641', '884718', '837406'], guppy cone (13 results) S ['21447186'], guppies (324 results) S ['21447186'], wave-sensitive opsins (23 results) D ['20353595', '18590925', '17015333'], wave-sensitive opsins (23 results) S ['18638376'], wave-sensitive opsin genes (14 results) D ['20353595', '17015333'], wave-sensitive opsin genes (14 results) S ['18638376'], large visual-opsin gene (1 results) S ['21447186'], visual-opsin gene repertoire (3 results) D ['16460888'], visual-opsin gene repertoire (3 results) S ['21447186', '19551143'], long wave-sensitive opsins (16 results) D ['20353595', '17015333'], long wave-sensitive opsins (16 results) S ['18638376'], long wave-sensitive opsin genes (1 results) D ['20353595'], large visual-opsin (2 results) S ['21447186'], opsin genes (192 results) D ['21170644', '20655939'], opsin genes (192 results) S ['21447186']
JE	TRUE	11	TRUE	performance feedback (498 results) S ['21443806'], better reflect contributions (64 results) S ['21443806'], interdisciplinary primary care teams (215 results) S ['21443806'], team performance (371 results) S ['21443806'], structures better reflect contributions (4 results) S ['21443806'], performance data (1335 results) S ['21443806'], care teams through team (4805 results) S ['21443806'], team performance feedback (262 results) S ['21443806'], feedback session (61 results) S ['21443806'], interdisciplinary teams (386 results) S ['21443806'], primary care teams (344 results) S ['21443806'], structures better reflect (139 results) S ['21443806'], performance indicators (1139 results) S ['21443806'], performance measurement (709 results) S ['21443806']
JE	TRUE	10	TRUE	social insect (235 results) S ['21447185'], eusociality (118 results) S ['21447185', '20337260'], eusocial evolution (10 results) S ['21447185'], task repertoire (206 results) S ['21447185'], caste (1134 results) S ['21447185'], eusocial (362 results) S ['21447185'], caste specific behaviors (990 results) S ['21447185'], lineage specific traits (10 results) S ['21447185'], eusocial traits (29 results) S ['21447185'], official gene set (5 results) S ['21447185'], taxonomically (1711 results) S ['21447185'], Hymenoptera (4130 results) S ['21447185'], official gene (215 results) S ['21447185'], bee official gene (2 results) S ['21447185'], novel eusocial (25 results) S ['21447185'], honey bee official gene (2 results) S ['21447185'], honey bee (1397 results) S ['21447185'], Apisimin (4 results) D ['12297291'], solitary ancestors (4 results) S ['21447185'], novel eusocial traits (3 results) S ['21447185']
JE	TRUE	2	TRUE	gyrA (1656 results) S ['21443804'], FQ-resistant MTB (2 results) D ['20452372'], gyrB (1069 results) S ['21443804'], FQ-resistant MTB isolates (2 results) D ['20452372'], ofloxacin-resistant (86 results) D ['20805388', '20185419', '19470506', '18559646', '18164184', '17035499'], ofloxacin-resistant (86 results) S ['21443804'], ofloxacin-resistant strains (20 results) D ['20805388', '19470506'], ofloxacin-resistant strains (20 results) S ['21443804']
.SE	FALSE	0	FALSE	searches

PMC3073879	7	FALSE	20	FALSE		8	27	TRUE	26	TRUE	24	TRUE	21	TRU
PMC3072947	0	TRUE	24	FALSE		9	18	TRUE	16	TRUE	14	TRUE	13	TRU
PMC3076230	10	FALSE	17	FALSE		10	20	TRUE	14	TRUE	12	TRUE	7	TRU

JE	TRUE	11	TRUE	cfa-mutant (4 results) D ['18562182'], bile tolerance (89 results) D ['20621375'], bile tolerance (89 results) S ['21447177'], natural protein diversity (2030 results) S ['21447177'], characteristic proteomic (325 results) S ['21447177'], characteristic proteomic profiles (45 results) S ['21447177', '19309013'], plantarum species with relation (34 results) D ['18539794'], plantarum species with relation (34 results) S ['21447177'], deleterious effects of bile (113 results) D ['20621375', '17827318'], Bsh activity (24 results) D ['18539794'], bile salts (3873 results) S ['21447177'], key proteins (1183 results) S ['21447177'], bile resistance (63 results) D ['19592587'], plantarum (2277 results) S ['21447177'], bile tolerance processes (10 results) D ['12839748'], bile tolerance processes (10 results) S ['21447177'], selection of probiotics (240 results) S ['21447177'], best probiotic potential (79 results) S ['21447177'], bacterial biomarkers (20 results) S ['21447177', '19309013'], early selection of probiotics (17 results) S ['19309013'], plantarum strains (125 results) S ['21447177'], proteomic profiles (290 results) S ['21447177'], differential bacterial properties (749 results) S ['21447177'], preliminary selection of strains (142 results) S ['21447177'], bacterial biomarkers for probiotic (59 results) S ['21447177', '19309013'], plantarum species (17 results) S ['21447177'], Bile tolerance properties (76 results) S ['21447177']
JE	TRUE	7	TRUE	young HRLF task (1 results) S ['21447183'], carpal tunnel performance (128 results) S ['19743505', '19686738'], sensorimotor declines (34 results) S ['20673790'], repetitive strain (283 results) S ['19743505'], young rats (7302 results) S ['21447183'], digitorum tendons with upper (58 results) S ['19743505'], rat model of MSD (9 results) S ['12507595'], cord inflammation (80 results) S ['20673790'], serum cytokine (1039 results) S ['21447183'], serum cytokine response (11 results) S ['21447183'], HRLF (7 results) S ['21447183', '20673790'], cytokine response (2682 results) S ['21447183'], HRLF task rats (2 results) S ['21447183', '20673790'], grip strength declines (73 results) S ['21447183', '20673790', '19429049'], young HRLF task rats (1 results) S ['21447183'], repetitive strain injuries (104 results) S ['19620321', '19032977'], HRLF rats (2 results) S ['21447183', '20673790'], cytokine response with age (7046 results) S ['21447183'], spinal cord neurochemicals (4 results) S ['19429049', '19032977', '18511022'], young HRLF (1 results) S ['21447183'], cord neurochemicals (4 results) S ['19429049', '19032977', '18511022'], repetitive task (91 results) S ['21447183', '20673790', '19429049'], spinal cord inflammation (63 results) S ['20673790'], HRLF task (2 results) S ['21447183', '20673790']
JE	TRUE	7	TRUE	AGL11 (7 results) D ['7549482'], VvAGL11 intragenic markers (1 results) S ['21447172'], VvAGL11 (1 results) S ['21447172'], VvAGL11 intragenic (1 results) S ['21447172'], seedless phenotype (18 results) D ['17426772', '15995866'], seedless phenotype (18 results) S ['21447172'], seedlessness (14 results) D ['19479258', '18419811', '17426772', '17103240', '12582493'], seedlessness (14 results) S ['21447172'], homeotic Arabidopsis gene SEEDSTICK (4 results) D ['17877710', '15722463'], Vitis (3554 results) S ['21447172'], inflorescence of seedless (2 results) D ['19479258', '17103240'], berry weight (17 results) D ['17426772', '15995866', '12582493'], berry weight (17 results) S ['21447172'], QTLs (2655 results) S ['21447172'], region of VvAGL11 (1 results) S ['21447172'], vinifera (1139 results) S ['21447172'], VMC7F2 (1 results) D ['17426772'], grapevine vinifera (435 results) S ['21447172'], regulatory region of VvAGL11 (1 results) S ['21447172'], seedless genotypes (3 results) D ['15995866', '12582493'], seedless genotypes (3 results) S ['21447172'], seedless (178 results) S ['21447172'], BMC Plant Biol (815 results) S ['21447172'], phenology-related (3 results) D ['18419811'], QTLs for seedlessness (4 results) D ['18419811', '17426772', '12582493'], QTLs for seedlessness (4 results) S ['21447172'], Vitis vinifera (1094 results) S ['21447172']

PMC3074532	2	FALSE	23	FALSE		5	20	TRUE	10	TRUE	9	TRUE	9	TRU
PMC3073904	1	FALSE	11	FALSE		2	12	FALSE	12	TRUE	11	TRUE	10	TRU
PMC3073961	11	FALSE	6	FALSE		5	17	TRUE	12	TRUE	10	TRUE	8	TRU
PMC3073910	4	FALSE	17	FALSE		3	18	FALSE	18	TRUE	17	TRUE	14	TRU
PMC3072333	0	TRUE	2	FALSE		1	2	FALSE	2	FALSE	2	FALSE	2	FAL

JE	TRUE	6	TRUE	worn-out burnout (6 results) S ['21447169', '20525178', '19948055'], frenetic burnout (4 results) S ['21447169', '20525178', '19948055'], different burnout types (54 results) S ['21447169', '19948055'], frenetic subtype (2 results) S ['21447169', '20525178'], worn-out profile (3 results) S ['21447169', '20525178'], type of burnout (200 results) S ['21447169', '20525178'], burnout subtypes (3 results) S ['21447169', '20525178', '19948055'], burnout syndrome (247 results) S ['21447169'], worn-out (157 results) S ['21447169', '20525178', '19948055'], frenetic (45 results) S ['21447169', '20525178', '19948055'], frenetic profile (1 results) S ['20525178'], automatic interaction detection analysis (10 results) D ['14678913'], different burnout (504 results) S ['21447169'], underchallenged (5 results) D ['10852153'], underchallenged (5 results) S ['21447169', '20525178', '19948055'], burnout types (3 results) S ['21447169', '19948055'], frenetic subjects (5 results) S ['21447169'], clinical profile (3304 results) S ['21447169'], burnout profile (68 results) S ['21447169', '20525178'], burnout subtype (3 results) S ['21447169', '20525178'], occupational characteristics (163 results) S ['21447169'], worn-out burnout subtype (2 results) S ['21447169', '20525178'], differential characterisation (3 results) S ['21447169'], different burnout subtypes (3 results) S ['21447169', '20525178', '19948055'], multi-occupational sample (2 results) S ['21447169']
JE	TRUE	8	TRUE	worldwide population (167 results) S ['21443805'], higher expenditures reported (307 results) S ['21443805'], acute morbidity (260 results) S ['21443805'], article of Werblow (9 results) D ['17311357'], hospitalization expenditures (11 results) S ['21443805'], determinants of hospitalization (1323 results) S ['21443805'], ENSANUT (26 results) S ['21443805'], higher probability (2375 results) S ['21443805'], group requires immediate attention (29 results) S ['21443805'], ambulatory care (45996 results) S ['21443805'], important demographic phenomena (283 results) S ['21443805'], health costs (2103 results) S ['21443805']
JE	TRUE	3	TRUE	PCR-AG (5 results) S ['21447159', '15139469'], PTB suspects (11 results) S ['21447159'], versus Ziehl-Neelsen smear microscopy (9 results) D ['16104634'], in-house IS6110 polymerase chain (27 results) D ['14974753'], house PCR (150 results) S ['21447159'], reaction versus Ziehl-Neelsen (5 results) D ['16104634'], PCR dot-blot (39 results) S ['21447159', '15139469'], reaction versus Ziehl-Neelsen smear (4 results) D ['16104634'], NAA commercial tests (8 results) D ['16202138'], Porto for laboratory (2672 results) S ['21447159'], chain reaction versus Ziehl-Neelsen (4 results) D ['16104634'], Ziehl-Neelsen smear microscopy (4 results) D ['16104634'], versus Ziehl-Neelsen smear (12 results) D ['16104634'], versus Ziehl-Neelsen (30 results) D ['16104634'], dot-blot (6145 results) S ['21447159'], in-house IS6110 polymerase (27 results) D ['14974753'], in-house IS6110 (30 results) D ['16202138', '14974753']
JE	TRUE	12	TRUE	salmon Salmo (1631 results) S ['21447175'], Salmo (4568 results) S ['21447175'], 44K oligo salmonid microarray (1 results) S ['21447175'], oligo salmonid microarray (14 results) D ['19585168'], oligo salmonid microarray (14 results) S ['21447175'], salmonid microarray (8 results) S ['21447175', '16164747'], salar (2326 results) S ['21447175'], gene expression during smoltification (19 results) D ['19585168'], Atlantic salmon salar (2153 results) S ['21447175'], salmonid (1483 results) S ['21447175'], salmon Salmo salar (1621 results) S ['21447175'], oligo salmonid (591 results) S ['21447175'], GSE25938 (1 results) S ['21447175'], expression during smoltification (31 results) D ['19585168'], smoltification of Atlantic (56 results) D ['19585168'], GSE25938 GSE25938 (1 results) S ['21447175'], Atlantic salmon Salmo salar (1482 results) S ['21447175'], Salmo salar (2145 results) S ['21447175'], salmon salar (2106 results) S ['21447175'], Atlantic salmon Salmo (1484 results) S ['21447175'], Atlantic salmon (2208 results) S ['21447175']
.SE	TRUE	2	TRUE	immunoexpression (1060 results) S ['21443808'], findings on co-expression (3355 results) S ['21443808']

PMC3070428	7	FALSE	8	FALSE		7	15	TRUE	14	TRUE	13	TRUE	11	TRU
PMC3076991	0	TRUE	3	FALSE		2	3	FALSE	3	TRUE	2	TRUE	2	TRU
PMC3070212	3	FALSE	21	FALSE		9	21	TRUE	19	TRUE	17	TRUE	12	TRU
PMC3070214	2	FALSE	7	FALSE		5	9	FALSE	9	FALSE	9	FALSE	9	TRU
PMC3070216	5	FALSE	16	FALSE		8	21	TRUE	16	TRUE	16	TRUE	15	TRU

JE	TRUE	5	TRUE	UniProtKB entries (5 results) D ['18957448', '18390879'], automatic annotation procedures (278 results) S ['21447597'], UniProtKB data (60 results) S ['21447597'], UniProt Consortium (15 results) D ['19426475'], UniProt Consortium (15 results) S ['21447597'], hub of protein (753 results) S ['21447597'], UniProt (273 results) S ['21447597'], Genome Database premier (7 results) D ['21051359'], central hub of protein (131 results) S ['21447597'], UniProt web (76 results) D ['19662629'], UniProtKB (88 results) S ['21447597'], manual curation (165 results) D ['21051359'], UniProt group (35 results) D ['19426475', '18957448'], automatic annotation (90 results) D ['19850725'], automatic annotation (90 results) S ['21447597']
JE	TRUE	1	TRUE	Historic Concentrations of Black (9 results) S ['21446726'], pollution exposures across Europe (26 results) S ['19875153'], model evaluation (354 results) S ['21446726']
JE	TRUE	7	TRUE	reinforcer devaluation task (6 results) S ['14657165'], critical role OFC (34 results) D ['20346766', '18538938'], reinforcer devaluation (40 results) S ['21483781'], orbitofrontal (3167 results) S ['21483781'], Schoenbaum (423 results) S ['21483781', '20206497'], outcome-expectant (2 results) S ['16338994'], fewer cue-selective neurons (1 results) S ['12948451'], cue-selective (13 results) S ['17100852', '16338994', '12948451'], Pavlovian reinforcer devaluation (3 results) S ['21483781'], devaluation (557 results) S ['21483781'], orbitofrontal cortex (2110 results) S ['21483781'], reversal-impaired (1 results) S ['16338994'], Pavlovian reinforcer (87 results) S ['21483781'], Pavlovian (2476 results) S ['21483781'], extinction probe test (38 results) S ['21483781'], cue-outcome associations (17 results) D ['15727536'], cue-outcome associations (17 results) S ['17100852'], cue-selective neurons (9 results) S ['16338994', '12948451'], devaluation task (13 results) D ['15727536'], devaluation task (13 results) S ['14657165'], fewer cue-selective (1 results) S ['12948451'], cue-outcome (40 results) S ['17100852'], reinforcer (2519 results) S ['21483781'], OFC (871 results) S ['21483781']
JE	TRUE	5	TRUE	tyrosine hydroxylase (12973 results) S ['21483723'], vertebrates New dopaminergic (11530 results) S ['21483723'], diencephalic (3063 results) S ['21483723'], dopaminergic territories (87 results) D ['19234064'], dopaminergic territories (87 results) S ['21192085', '20123022'], New dopaminergic territories (32 results) S ['20123022'], teleosts (3406 results) S ['21483723'], vertebrates New dopaminergic territories (32 results) S ['20123022'], Catecholamine systems (194 results) D ['18924139']
JE	TRUE	14	TRUE	spiny projection (171 results) D ['20613723'], spiny projection (171 results) S ['21483724', '20589098', '19474176'], corticostriatal response (3 results) S ['19350384'], facilitates striatal EPSPs (3 results) S ['9243608'], corticostriatal (1182 results) S ['21483724'], Front Syst Neurosci (102 results) S ['21483724'], indirect pathways (387 results) D ['20682746'], indirect pathways (387 results) S ['21483724'], Different corticostriatal integration (18 results) S ['21483724', '20589098'], hydroxylase-immunoreactive boutons (3 results) D ['6152036'], medium spiny neurons (604 results) S ['21483724'], Dopamine facilitates striatal EPSPs (2 results) S ['9243608'], spiny neurons (1037 results) S ['21483724'], Tyrosine hydroxylase-immunoreactive boutons (3 results) D ['6152036'], corticostriatal integration (49 results) S ['21483724', '20589098'], Different corticostriatal (218 results) S ['21483724', '20589098'], projection neurons (2892 results) S ['21483724'], spiny projection neurons (155 results) D ['20613723'], spiny projection neurons (155 results) S ['21483724', '20589098', '19474176'], spiny (3411 results) S ['21483724'], medium spiny (796 results) S ['21483724']

PMC3078839	3	FALSE	25	FALSE		7	26	TRUE	20	TRUE	20	TRUE	18	TRU
PMC3078838	5	FALSE	16	FALSE		12	21	TRUE	18	TRUE	17	TRUE	13	TRU
PMC3072321	5	FALSE	2	FALSE		5	7	TRUE	4	TRUE	3	TRUE	2	TRU
PMC3076503	0	TRUE	19	FALSE		5	14	TRUE	13	TRUE	13	TRUE	13	TRU

JE	TRUE	11	TRUE	fatigue scale (399 results) S ['21447160'], EORTC Quality (203 results) S ['21447160', '20972628'], item bank (132 results) S ['21447160'], EORTC item style (1 results) S ['21447160'], QLQ-C30 dimensions (72 results) S ['20231085'], pre-testing (250 results) S ['21447160'], EORTC item (188 results) S ['21447160', '20972628'], Life Group (167 results) S ['21447160', '20972628'], item list (90 results) S ['21447160'], EORTC fatigue item bank (3 results) S ['21447160'], fatigue item bank (3 results) D ['17001438'], Fatigue CAT (1 results) S ['21447160'], EORTC approach (4 results) S ['21447160'], QLQ-C30 dimensions general approach (3 results) S ['20231085'], comprehensive item selection (54 results) S ['21447160'], item style (747 results) S ['21447160'], QLQ-C30 (1337 results) S ['21447160'], item selection (250 results) S ['21447160'], EORTC Fatigue (372 results) S ['21447160'], EORTC QLQ-C30 dimensions (65 results) S ['20231085'], original QLQ-C30 (19 results) S ['21447160', '20231085'], QLQ-C30 fatigue (317 results) S ['21447160'], static short forms (95 results) D ['19738214'], fatigue item (18 results) D ['17001438', '12781305', '8463991'], EORTC CAT (5 results) S ['21447160', '20972628', '20231085'], QLQ-C30 fatigue scale (2 results) S ['21447160'], comprehensive item selection procedure (37 results) S ['21447160'], EORTC project (61 results) S ['21447160', '20231085']
JE	TRUE	8	TRUE	PANSS-EC (29 results) D ['18647402', '18255271', '17316169'], PANSS-EC (29 results) S ['21447155'], agitated (2758 results) S ['21447155'], equipercentile (12 results) D ['18255271', '17949948', '15982856'], equipercentile (12 results) S ['21447155'], scale's diagnostic validity (135 results) S ['21447155'], scale's diagnostic (232 results) S ['21447155'], PANSS-EC items (2 results) D ['17201610'], PANSS-EC items (2 results) S ['21447155'], acute psychotic episodes (81 results) S ['21447155'], quetiapine versus divalproex (22 results) D ['17201610'], PANSS (1813 results) S ['21447155'], PANSS-EC scales (17 results) D ['18255271', '17316169', '12920409', '12867218', '11982448', '11927174'], PANSS-EC scales (17 results) S ['21447155', '16697151'], acute psychosis (703 results) S ['21447155'], PANSS-EC subscale (2 results) S ['21447155'], acute psychotic (3585 results) S ['21447155'], intramuscular olanzapine (34 results) S ['16697151'], CGI-S (518 results) S ['21447155'], unifactorial structure (15 results) S ['21447155'], severity of agitation (904 results) S ['21447155']
JE	TRUE	1	TRUE	HPV-negative (861 results) S ['21447181'], sensitive broad-spectrum detection (3 results) D ['18193443'], OPSCCs (2 results) S ['21447181'], follow-up salivary rinses (6 results) D ['18329326'], aetiology of OSCCs (13 results) D ['18235120'], normal clinico-pathological evaluation (31 results) D ['18193443'], tonsil SCCs (3 results) D ['14724900']
JE	TRUE	10	TRUE	mitochondrion-neuron (1 results) S ['21494430'], rat mitochondrion-neuron (1 results) S ['21494430'], Brain Tissues Mitochondrial function (3741 results) S ['21494430'], identification of differential (13638 results) S ['21494430'], systems-biological study (30 results) S ['21494430'], rapid identification of differential (776 results) S ['21494430'], Genes between Frontal (1418 results) S ['21494430'], bioenergenesis (1 results) S ['21494430'], interpretation for microarray (1646 results) S ['21494430'], neurotransmitters (17120 results) S ['21494430'], bioinformatics tools (805 results) S ['21494430'], frontal cortex (13016 results) S ['21494430'], microarray results (824 results) S ['21494430'], rMNChip (1 results) S ['21494430'], rapid identification (4409 results) S ['21494430'], differential pathways (55 results) S ['21494430'], mitochondria-focused (5 results) S ['18690294', '18563568', '17390543', '16534508'], intuitive interpretation for microarray (21 results) S ['21494430'], rMNChip contains (1 results) S ['21494430']

PMC3077036	11	FALSE	10	FALSE		15	12	TRUE	9	TRUE	7	TRUE	7	TRU
PMC3073874	8	FALSE	10	FALSE		6	12	TRUE	9	TRUE	9	TRUE	9	TRU
PMC3078858	4	FALSE	13	FALSE		4	17	FALSE	17	FALSE	17	TRUE	16	TRU
PMC3076252	2	FALSE	7	FALSE		4	9	TRUE	8	TRUE	7	TRUE	7	TRU
PMC3075216	8	FALSE	7	FALSE		11	12	TRUE	10	TRUE	6	TRUE	3	TRU

JE	TRUE	0	FALSE	pyridine-thiazole cores (1 results) S ['19572592'], multistep Bohlmann-Rahtz (1 results) D ['15704975'], One-pot multistep Bohlmann-Rahtz heteroannulation (1 results) D ['15704975'], cores of thiopeptide (1 results) S ['19572592'], synthesis of pyridine-thiazole (1 results) S ['19572592'], Bohlmann-Rahtz heteroannulation synthesis (5 results) D ['15704975'], thiopeptide antibiotics (29 results) D ['19624105', '18540632', '15704975', '15700961', '12715871'], thiopeptide antibiotics (29 results) S ['19572592'], micrococcin (50 results) S ['20179875', '10836044'], thiocillin (8 results) D ['20522549', '20455532', '19911780', '19246004', '7328054'], thiocillin (8 results) S ['21446660'], pyridine-thiazole cores of thiopeptide (1 results) S ['19572592'], heteroannulation synthesis (37 results) D ['20707374'], thiopeptide (94 results) D ['20707374'], thiopeptide (94 results) S ['21446660'], Ciufolini (88 results) S ['21446660', '20179875', '19572592'], multistep Bohlmann-Rahtz heteroannulation (1 results) D ['15704975'], Xiong Bower (6 results) D ['15704975'], heteroannulation synthesis of dimethyl (3 results) D ['15704975'], pyridine-thiazole (1 results) S ['19572592'], One-pot multistep Bohlmann-Rahtz (1 results) D ['15704975']
JE	TRUE	4	TRUE	human lung cancer A549 (90 results) S ['21447176'], lung cancer A549 (217 results) S ['21447176'], tsugae extract inhibits expression (1 results) D ['19332363'], tsugae extract inhibits (1 results) D ['19332363'], inactivation of WEE1Hu (5 results) D ['15964826'], Osthole induces (1 results) S ['21447176'], Osthole induces arrest (1 results) S ['21447176'], A549 cells with Osthole (1 results) S ['21447176'], Triptolide sensitizes AML cells (2 results) D ['18187663'], cancer A549 cells (136 results) S ['21447176'], Osthole (84 results) D ['20622464'], Osthole (84 results) S ['21447176'], lung cancer A549 cells (132 results) S ['21447176'], Ganoderma tsugae extract inhibits (1 results) D ['19332363'], Triptolide sensitizes AML (2 results) D ['18187663'], cancer A549 (223 results) S ['21447176'], cells with Osthole (52 results) D ['20622464', '19678539'], cells with Osthole (52 results) S ['21447176']
JE	TRUE	15	TRUE	alternative salvage therapies (956 results) S ['21447168'], common cause of non-cirrhotic (32 results) D ['12243791'], Budd-Chiari syndrome with percutaneous (184 results) S ['21447168'], re-stenosis (378 results) S ['21447168'], hepatic venous (2502 results) S ['21447168'], Budd-Chiari (3030 results) S ['21447168'], Budd-Chiari syndrome (3008 results) S ['21447168'], recurrent Budd-Chiari (120 results) S ['21447168'], venous outflow (2155 results) S ['21447168'], percutaneous transluminal (11123 results) S ['21447168'], hepatic venous outflow (261 results) S ['21447168'], transluminal angioplasty (4069 results) S ['21447168'], transluminal angioplasty transjugular (121 results) D ['19652186'], therapeutic modalities fail (328 results) S ['21447168'], percutaneous transluminal angioplasty (3473 results) S ['21447168'], transluminal angioplasty transjugular intrahepatic (105 results) D ['19652186'], Aberrant Portal Blood Flow (20 results) D ['7751574']
JE	TRUE	5	TRUE	Medical Case Reports (24 results) D ['18822140', '17411446'], reinforce clinical knowledge (172 results) S ['21447163'], retrieval of relevant (2009 results) S ['21447163'], Ishikawa diagram (8 results) S ['21447163'], potential causes (2101 results) S ['21447163'], medical cases (253 results) S ['21447163'], steroid cell tumor (48 results) D ['18088412'], relevant medical cases (2 results) S ['21447163'], lifelong learning (648 results) S ['21447163']
JE	TRUE	2	TRUE	Bcl2 through Akt-dependent (7 results) S ['18199533'], t-Darpp promotes (1 results) S ['18199533'], anti-apoptotic actions of GSK3 (2 results) D ['16935409'], paradoxical pro- (17 results) D ['16935409'], El-Rifai (129 results) S ['21447180', '20836878'], suppress medulloblastoma growth (19 results) D ['20028853'], beta-catenin phosphorylation-independent manner (3 results) D ['18223684'], t-DARPP (12 results) D ['15363322'], t-DARPP (12 results) S ['21447180', '20836878', '18579663', '18199533', '16061638', '14508844', '12124342'], GSK-3beta (1878 results) S ['21447180'], t-Darpp promotes cancer cell (1 results) S ['18199533'], t-Darpp promotes cancer (1 results) S ['18199533'], beta-catenin phosphorylation-independent (7 results) D ['18223684'], Nuclear GSK-3beta (7 results) D ['18223684'], phosphorylation-independent manner (78 results) D ['18223684']

PMC3072353	3	FALSE	15	FALSE		2	18	TRUE	14	TRUE	13	TRUE	12	TRU
PMC3073958	1	FALSE	19	FALSE		10	17	TRUE	11	TRUE	8	TRUE	7	TRU
PMC3066135	6	FALSE	5	FALSE		5	5	TRUE	4	TRUE	4	TRUE	3	TRU
PMC3066128	8	FALSE	4	FALSE		14	11	TRUE	4	TRUE	3	TRUE	3	TRU
PMC3066134	3	FALSE	7	FALSE		5	10	FALSE	10	TRUE	8	TRUE	6	TRU
PMC3066129	3	FALSE	7	FALSE		3	10	TRUE	8	TRUE	8	TRUE	6	TRU

JE	TRUE	9	TRUE	RealTime assays for quantitative (28 results) D ['17409216'], RB1 status of cell (103 results) S ['21447152'], RB1 status (8 results) S ['21447152'], Functional loss of RB1 (35 results) S ['21447152'], Abbott RealTime assays (2 results) D ['17409216'], new Abbott RealTime assays (15 results) D ['17409216'], RB1-negative cancers (4 results) S ['21447152'], expression ratio (590 results) S ['21447152'], RB1-positive (3 results) S ['21447152'], prevalence of RB1 (74 results) S ['21447152'], status of RB1 (142 results) S ['21447152'], CDKN2A (1797 results) S ['21447152'], anti-tumor agents (384 results) S ['21447152'], xenograft tumors (911 results) S ['21447152'], RB1-negative (4 results) S ['21447152'], CCND1 (1209 results) S ['21447152'], RB1 (1789 results) S ['21447152'], clinical tumor (704 results) S ['21447152']
JE	TRUE	3	TRUE	Brunaud (172 results) S ['21447150'], FLAGdb application (1 results) S ['21447150'], FLAGdb database (2 results) S ['14681431'], FLcDNA cloning (1 results) S ['20964856'], grapevine terpene synthase gene (2 results) S ['20964856'], CATMA transcriptome data (3 results) S ['17980019', '17940091'], FLAGdb (5 results) D ['18644000'], FLAGdb (5 results) S ['21447150', '14681431'], orthology group (95 results) S ['21447150'], grapevine terpene synthase (2 results) S ['20964856'], plant genomes (754 results) S ['21447150'], Aubourg (201 results) S ['21447150', '20964856'], CATMA transcriptome (8 results) S ['17980019', '17940091'], grapevine terpene (42 results) S ['20964856'], Martin-Magniette (21 results) S ['18644002', '17980019', '17940091'], Analysis of CATMA (7 results) S ['17980019', '17940091'], Lecharny (57 results) S ['21447150', '17980019', '17940091', '17721507', '15608279', '15269332', '14681431'], Balzergue (22 results) S ['17980019', '17940091'], CATMA transcriptome data identifies (1 results) S ['17980019'], transcriptome data identifies hundreds (9 results) S ['17980019']
JE	TRUE	2	TRUE	alpha-chain mediate (171 results) S ['20519503'], receptor alpha-chain mediate ubiquitination (1 results) S ['20519503'], identifies Dsk2p (1 results) D ['15167887'], antigen receptor alpha-chain mediate (61 results) S ['20519503'], associates with p97 (21 results) D ['19818707', '15215856'], alpha-chain mediate ubiquitination (1 results) S ['20519503'], otubain YOD1 (1 results) D ['19818707'], receptor alpha-chain mediate (112 results) S ['20519503'], genomic screen identifies Dsk2p (1 results) D ['15167887'], ERAD of MHC-I (3 results) D ['17502423'], screen identifies Dsk2p (1 results) D ['15167887']
JE	TRUE	2	TRUE	d-tubocurarine analogs (4 results) D ['12243766', '9680250', '9312090'], Curariform antagonists (58 results) D ['12799358', '12682067', '8132615', '8415719'], nicotinic acetylcholine receptor homolog (3 results) S ['16505382', '15951818'], acetylcholine receptor homolog AChBP (3 results) S ['16505382', '15951818'], recombinant 5-hydroxytryptamine3A (5 results) D ['10347245'], curare selectivity (9 results) D ['8415719'], recombinant 5-hydroxytryptamine3A receptor subunits (1 results) D ['10347245'], Interaction of d-tubocurarine (276 results) D ['20441748', '16931985', '16723497'], 5-hydroxytryptamine3A receptor subunits (3 results) D ['10347245'], recombinant 5-hydroxytryptamine3A receptor (5 results) D ['10347245'], receptor homolog AChBP (3 results) S ['16505382', '15951818'], AChBP (131 results) S ['21468359']
JE	TRUE	4	TRUE	high proportion of intersubtype (7 results) D ['15186532'], resistance mutations (2466 results) S ['21468304'], zidovudine (10744 results) S ['21468304'], proportion of intersubtype (12 results) D ['15186532'], single-Dose NVP versus (14 results) D ['18684096', '11600822'], Drug Resistance Emergence (9 results) S ['21468304'], nevirapine (2928 results) S ['21468304'], Antiretroviral concentrations (22 results) S ['19114673'], Triple-Antiretroviral Prophylaxis (25 results) S ['21468304'], Mothers Drug Resistance (490 results) S ['21468304']
JE	TRUE	6	TRUE	PMTCT single-arm trial (2 results) S ['21468300'], maternal triple-antiretroviral regimen (2 results) S ['21468300'], zidovudine (10744 results) S ['21468300'], mothers with triple (125 results) D ['20554982'], triple-antiretroviral (124 results) D ['19730269'], triple-antiretroviral (124 results) S ['21468300'], maternal triple-antiretroviral (11 results) D ['19730269'], maternal triple-antiretroviral (11 results) S ['21468300'], nevirapine (2928 results) S ['21468300'], Kisumu (373 results) S ['21468300']

PMC3066162	5	FALSE	10	FALSE		8	13	TRUE	12	TRUE	11	TRUE	10	TRU
PMC3066159	11	FALSE	24	FALSE		13	31	TRUE	28	TRUE	23	TRUE	21	TRU
PMC3066158	5	FALSE	10	FALSE		7	14	TRUE	13	TRUE	12	TRUE	11	TRU
PMC3066153	3	FALSE	10	FALSE		8	11	TRUE	9	TRUE	9	TRUE	9	TRU
PMC3066143	7	FALSE	0	TRUE		4	7	FALSE	7	FALSE	7	TRUE	5	TRU

JE	TRUE	7	TRUE	Schistosoma (16960 results) S ['21468317'], trichuris infections on children's (21 results) S ['18523547'], Dec Infect (5 results) D ['11963321'], Schistosoma japonicum (2624 results) S ['21468317'], misclassification (3830 results) S ['21468317'], comparison of immunodiagnostic (87 results) D ['17556625'], japonicum infection after correction (1 results) S ['21468317'], joint misclassification of exposure (19 results) D ['15736279', '8410104'], japonicum (4589 results) S ['21468317'], children's anemia burden (42 results) S ['18523547'], japonicum infection (308 results) S ['21468317'], Western Samar province (1 results) S ['16799728'], hookworm infections among schoolchildren (122 results) D ['18982057'], Field comparison of immunodiagnostic (7 results) D ['17556625'], hookworm (3947 results) S ['21468317']
JE	TRUE	13	TRUE	multiple treatments (624 results) S ['21468315'], High-level ivermectin (30 results) D ['12237144'], High-level ivermectin (30 results) S ['15228717'], adult Onchocerca (113 results) D ['18831801', '12133654'], adult Onchocerca volvulus persist (3 results) D ['8134772'], adult Onchocerca volvulus persist (3 results) S ['15119969'], ivermectin (4890 results) S ['21468315'], annual treatment with ivermectin (159 results) S ['21468315'], village of Gami (1 results) D ['12061976'], doses of ivermectin (349 results) S ['21468315'], onchocerciasis-endemic foci (4 results) S ['15228717', '15119969'], multiple treatments with ivermectin (81 results) D ['18831801'], multiple treatments with ivermectin (81 results) S ['21468315'], Thirty-month follow-up of sub-optimal (1 results) S ['15228717'], treatments with ivermectin (1477 results) S ['21468315'], embryogenesis of Onchocerca (50 results) D ['19784672'], microfilaridermias (2 results) S ['21468315', '15119969'], intensity of Onchocerca (124 results) S ['17574093'], two-phase epidemiological study (7 results) S ['17574093'], persistent microfilaridermias (2 results) S ['21468315', '15119969'], treatments with IVM (832 results) S ['21468315'], Onchocerca (2497 results) S ['21468315'], skin microfilarial repopulation (8 results) D ['7992347'], skin microfilarial repopulation (8 results) S ['21468315', '19805362', '17574093'], adult Onchocerca volvulus (88 results) D ['18831801', '12133654'], Onchocerca volvulus (1679 results) S ['21468315'], field isolate of Haemonchus (18 results) D ['12237144'], volvulus (6627 results) S ['21468315'], onchocerciasis (3978 results) S ['21468315'], onchocerciasis-endemic (72 results) S ['15228717', '15119969'], ivermectin on adult (1352 results) S ['21468315'], multiple treatments with IVM (31 results) S ['21468315'], High-level ivermectin resistance (15 results) D ['12237144'], adult female Onchocerca volvulus (8 results) D ['1293732'], adult female Onchocerca volvulus (8 results) S ['21468315']
JE	TRUE	6	TRUE	minicircle DNA use (473 results) S ['21468314'], chicken model system (7 results) S ['21468314'], Trypanosoma flow cytometric analysis (27 results) D ['19393242'], reveal extensive DNA content (53 results) D ['19393242'], kDNA (458 results) S ['21468314'], cruzi infection (1600 results) S ['21468314'], chickens with kDNA (16 results) S ['21468314'], minicircle sequences (34 results) D ['17524149', '17123956'], minicircle sequences (34 results) S ['20169193'], minicircles (478 results) S ['21468314'], Trypanosoma flow cytometric (29 results) D ['19393242'], Trypanosoma cruzi (10364 results) S ['21468314'], kDNA mutations (1 results) S ['17072450'], minicircle (497 results) S ['21468314'], mithramycin-induced (5 results) D ['6182288']
JE	TRUE	6	TRUE	Aedes aegypti (5036 results) S ['21468313'], lightness of technocratic (1 results) S ['18346029'], dengue vector results (476 results) S ['21468313'], water container (42 results) D ['19263854'], control of dengue (2466 results) S ['21468313'], aegypti (5290 results) S ['21468313'], Effectiveness of dengue (120 results) S ['21468313'], dengue control (127 results) D ['20439962'], unbearable lightness of technocratic (1 results) S ['18346029'], dengue vectors with curtains (4 results) S ['16735334'], insecticidal water container (2 results) D ['19263854'], Vanlerberghe (88 results) S ['21468313', '19917036', '19840350', '19509031', '18346029'], vectors with curtains (38 results) S ['16735334']
JE	TRUE	1	TRUE	Trichomonas vaginalis repeat (30 results) D ['20502393'], vaginalis repeat (52 results) D ['20502393'], Epidemic dengue (47 results) D ['18337327'], Trichomonas vaginalis repeat infection (14 results) D ['20502393'], vaginalis infection among reproductive-age (21 results) D ['17968828'], Texas-Mexico (134 results) D ['20489680'], vaginalis repeat infection (17 results) D ['20502393']

PMC3066142	13	FALSE	12	FALSE		12	25	TRUE	22	TRUE	17	TRUE	17	TRU
PMC3066141	0	TRUE	23	FALSE		4	21	TRUE	17	TRUE	14	TRUE	12	TRU
PMC3066140	4	FALSE	10	FALSE		5	13	TRUE	12	TRUE	12	TRUE	11	TRU
PMC3066139	4	FALSE	13	FALSE		5	17	TRUE	13	TRUE	11	TRUE	6	TRU

JE	TRUE	7	TRUE	Schistosoma (16960 results) S ['21468311'], mansoni (12802 results) S ['21468311'], adult schistosome (46 results) D ['16101292'], adult schistosome (46 results) S ['16269422'], identification of tegumental (33 results) D ['18638509'], tegument protein Sm29 (6 results) D ['18827884'], tegument protein Sm29 (6 results) S ['21468311'], female Schistosoma bovis (106 results) D ['18638509'], tegument surface (35 results) D ['18827884', '18638509', '17134654'], tegument surface (35 results) S ['21468311', '16447162'], biotinylation (4734 results) S ['21468311'], tegument (2100 results) S ['21468311'], Schistosoma mansoni via DNA (31 results) D ['15828575', '12819068'], Schistosoma mansoni tegument (8 results) D ['18827884'], Schistosoma mansoni tegument (8 results) S ['21468311'], bovis worms (6 results) D ['18638509'], tegumental glycoprotein (4 results) D ['9427998', '1833182'], Schistosoma bovis worms (4 results) D ['18638509'], female Schistosoma bovis worms (78 results) D ['18638509'], mansoni via DNA (32 results) D ['15828575', '12819068'], Schistosoma mansoni (10835 results) S ['21468311'], Schistosoma mansoni tegument protein (247 results) S ['21468311'], schistosome surface (34 results) D ['17134654'], schistosome surface (34 results) S ['16269422'], tegument surface membranes (3 results) S ['16447162', '6669162']
JE	TRUE	9	TRUE	term tree (3099 results) S ['21468310'], multi-disease data management system (3 results) S ['21468310'], Marlize Coleman (4 results) S ['21468310'], geographical entity tree (2 results) S ['21468310'], Lars Eisen (39 results) S ['21468310', '20868280', '19399163', '18797648'], capacity for dengue (182 results) S ['21468310'], data management system (381 results) S ['21468310'], McEachen (30 results) S ['21468310'], evaluate control program performance (1852 results) S ['21468310'], Miguel Orlans (1 results) S ['21468310'], geographical entity (7 results) S ['21468310'], new diseases downstream (1764 results) S ['21468310'], current system with capacity (2019 results) S ['21468310'], Orlans (109 results) S ['21468310'], multi-disease (32 results) S ['21468310'], multi-disease data management (3 results) S ['21468310'], Lozano-Fuentes (11 results) S ['21468310', '19399163', '18797648'], Saul Lozano-Fuentes (11 results) S ['21468310', '19399163', '18797648'], multi-disease data (18 results) S ['21468310'], current capacity for dengue (14 results) S ['21468310'], Nathan McEachen (1 results) S ['21468310'], display labels (320 results) S ['21468310'], entity tree (232 results) S ['21468310']
JE	TRUE	8	TRUE	hookworms (708 results) S ['21468309'], beta-tubulin codon polymorphism (31 results) D ['19308251'], Ancylostoma life history parameters (1 results) D ['627275'], anthelmintic (4198 results) S ['21468309'], Trichuris (2405 results) S ['21468309'], albendazole (3741 results) S ['21468309'], dose of ALB (215 results) S ['21468309'], soil-transmitted (490 results) S ['21468309'], vitro anthelmintic sensitivity assays (38 results) S ['18242867'], Ancylostoma life history (16 results) D ['627275'], drug efficacy (2659 results) S ['21468309'], PLoS Negl Trop Dis (885 results) S ['21468309'], detection of Trichuris (76 results) S ['19172171'], sympatric hookworms (4 results) D ['627275']
JE	TRUE	3	TRUE	inpatient dengue cases (8 results) S ['21468308'], dengue inpatient (17 results) S ['21468308'], dengue inpatient cases (8 results) S ['21468308'], chimeric tetravalent dengue vaccine (30 results) D ['18762226'], dengue disease surveillance contribute (35 results) D ['18768080'], average age-group specific MFs (4 results) S ['21468308'], dengue disease surveillance (1807 results) S ['21468308'], dengue cases (242 results) S ['21468308'], flavivirus-primed (3 results) D ['18762226'], symptomatic dengue infection (5 results) S ['17467515'], symptomatic dengue (18 results) D ['3341519'], symptomatic dengue (18 results) S ['17467515'], dengue underrecognition (2 results) S ['21468308'], age-group specific MFs (84 results) S ['21468308'], inpatient dengue (17 results) S ['21468308'], outpatient dengue cases (14 results) S ['21468308'], dengue vaccine (165 results) S ['21468308']

PMC3066138	10	FALSE	1	FALSE		7	11	TRUE	10	TRUE	10	TRUE	8	TRU
PMC3066137	9	FALSE	19	FALSE		13	28	TRUE	23	TRUE	21	TRUE	17	TRU
PMC3066237	2	FALSE	7	FALSE		4	7	TRUE	6	TRUE	5	TRUE	5	TRU
PMC3066236	6	FALSE	11	FALSE		10	13	TRUE	12	TRUE	11	TRUE	4	TRU

JE	TRUE	1	TRUE	fly salivary proteins induce (11 results) D ['19461875'], Comparative real-time kinetic (105 results) D ['18023393'], consequences for Leishmania (74 results) D ['19461875'], recombinant Leishmania major strain (32 results) D ['15629360'], cutaneous implications for vaccine (45 results) D ['18389179'], adverse consequences for Leishmania (2 results) D ['19461875'], immunostimulatory oligodeoxynucleotides reduces (11 results) D ['12933855'], natural reservoir of visceral (41 results) D ['19461875'], Comparative real-time kinetic analysis (74 results) D ['18023393'], live vaccine against cutaneous (94 results) D ['19221514', '18389179'], live vaccine against cutaneous (94 results) S ['19543375']
JE	TRUE	7	TRUE	Oropsylla species (33 results) D ['19492944', '18787922', '17249353'], Yunnan Province (1129 results) S ['21468306'], commensal rodent (831 results) S ['21468306'], dynamics of Ctenocephalides (11 results) D ['16442233', '15638140'], floor flea (24 results) D ['9877075'], floor flea (24 results) S ['21468306'], villages endemic (1283 results) S ['21468306'], endemic commensal rodent (14 results) S ['21468306', '19068139'], villages endemic for commensal (4 results) S ['21468306', '19068139'], flea intensity (60 results) D ['19492944'], flea intensity (60 results) S ['21468306'], endemic commensal rodent plague (6 results) S ['21468306', '19068139'], households of villages (1305 results) S ['21468306'], commensal rodent plague (20 results) S ['21468306', '19068139'], flea abundance (23 results) D ['20158331', '19815894', '19492944', '18787922', '17032365'], flea abundance (23 results) S ['21468306'], small mammals (1613 results) S ['21468306'], rodent plague (29 results) S ['21468306', '19068139'], endemic for commensal (36 results) S ['21468306', '19068139'], commensal rats (19 results) D ['11426717'], Lianghe County (3 results) S ['21468306', '19068139'], flea species (179 results) D ['20158331'], flea species (179 results) S ['21468306'], host flea (9 results) S ['21468306'], endemic commensal (36 results) S ['21468306', '19068139'], Seasonal studies on commensal (5 results) D ['11426717'], prairie dog hosts (3 results) D ['20660742', '17249353'], Ctenocephalides felis populations (83 results) S ['21468306']
JE	TRUE	2	TRUE	chorioamnionitis prevents intestinal (1 results) S ['19503810'], Endotoxin (28295 results) S ['21479249'], Kallapur (80 results) S ['21479249'], chorioamnionitis prevents (8 results) S ['19503810'], preterm fetal lamb lungs (40 results) S ['16857758'], chorioamnionitis (2883 results) S ['21479249'], Alabama Preterm Birth polymorphonuclear (3 results) D ['16949415'], chorioamnionitis prevents intestinal development (1 results) S ['19503810'], Preterm Birth polymorphonuclear (48 results) D ['16949415']
JE	TRUE	2	TRUE	heparin-binding (5558 results) S ['21479248'], tuberculosis recombinant HBHA (17 results) D ['18815611', '16893986', '12149464'], tuberculosis recombinant HBHA (17 results) S ['21479248', '16622240', '16148186', '15451098'], HBHA (67 results) D ['20508213', '18815611'], HBHA (67 results) S ['21479248'], hemagglutinin-specific IFN-gamma (9 results) D ['20508213'], hemagglutinin (10561 results) S ['21479248'], tuberculosis evaluation of RD1-specific (2 results) S ['19930588', '17257436'], RD1-specific long-term (1 results) S ['19930588'], RD1-specific (12 results) S ['19930588', '17257436'], Mycobacterium tuberculosis recombinant HBHA (16 results) D ['18815611', '16893986', '12149464'], Mycobacterium tuberculosis recombinant HBHA (16 results) S ['16622240', '16148186', '15451098'], evaluation of RD1-specific (2 results) S ['19930588', '17257436'], tuberculosis recombinant HBHA protein (17 results) D ['18815611', '16893986', '12149464'], tuberculosis recombinant HBHA protein (17 results) S ['21479248', '16622240', '16148186', '15451098'], recombinant HBHA protein (3 results) D ['18815611'], RD1-specific long-term response (1 results) S ['19930588']

PMC3066235	8	FALSE	12	FALSE		16	18	TRUE	13	TRUE	12	TRUE	12	TRU
PMC3066234	3	FALSE	8	FALSE		2	9	TRUE	7	TRUE	6	TRUE	5	TRU
PMC3066233	8	FALSE	16	FALSE		9	22	TRUE	21	TRUE	21	TRUE	13	TRU
PMC3066232	6	FALSE	2	FALSE		2	6	TRUE	4	TRUE	1	TRUE	1	TRU
PMC3066231	1	FALSE	4	FALSE		5	5	FALSE	5	FALSE	5	TRUE	3	TRU
PMC3066230	7	FALSE	6	FALSE		5	12	TRUE	8	TRUE	6	TRUE	6	TRU

JE	TRUE	5	TRUE	aeruginosa PcrV antigen (26 results) D ['19903258', '19103766', '18583342', '15271936', '12495673'], aeruginosa PcrV antigen (26 results) S ['12089663'], aspects of T3S (4 results) S ['21479247'], type III secretion (2436 results) S ['21479247'], PcrV (49 results) D ['20487288', '19903258', '19103766', '18583342', '18039770', '17697254'], PcrV (49 results) S ['21479247'], Yersinia role of LcrV (35 results) D ['17966419', '16254184', '14962390', '14580388'], Pseudomonas aeruginosa PcrV antigen (26 results) D ['19903258', '19103766', '18583342', '15271936', '12495673'], Pseudomonas aeruginosa PcrV antigen (26 results) S ['12089663'], pcrV-null (1 results) S ['21479247'], LcrV (252 results) S ['21479247'], globular domain (1073 results) S ['21479247'], Ysc-Yop translocation (8 results) D ['14580388'], multifunctional aspects of T3S (1 results) S ['21479247'], Ysc-Yop translocation pore (2 results) D ['14580388'], III secretion (2463 results) S ['21479247'], wild-type PcrV (4 results) D ['15962216', '15271936'], PcrV derivatives (2 results) S ['21479247'], Yersinia (11539 results) S ['21479247'], host-cell sensing (4 results) D ['16888041']
JE	TRUE	4	TRUE	ODD-luciferase transgenic (1 results) S ['21479246'], prolyl hydroxylase assessment (20 results) D ['16373502'], palpable tumors (348 results) S ['21479246'], mammary tumors (4535 results) S ['21479246'], stimulates erythropoietin production (6 results) D ['16373502'], ODD-luciferase (3 results) S ['21479246'], spontaneous tumors (810 results) S ['21479246'], ODD-luciferase transgenic mouse (1 results) S ['21479246'], HIF prolyl hydroxylase assessment (4 results) D ['16373502'], substantial bioluminescent (32 results) S ['21479246'], bioluminescent (2198 results) S ['21479246']
JE	TRUE	8	TRUE	response host cell transcription (3054 results) S ['21479245'], host cell transcription (79 results) D ['20624917', '17183270', '17069459'], host cell transcription (79 results) S ['21479245', '18678671'], discharge correlates (14 results) S ['18678671'], rhoptry (432 results) D ['21199955'], cell transcription (566 results) S ['21479245'], Toxoplasma gondii rhoptry (16 results) S ['18678671'], gondii rhoptry (134 results) D ['21199955', '20624917', '19956582'], rhoptry discharge correlates (1 results) S ['18678671'], host cell transcription factor (6 results) S ['21479245', '18678671'], rhoptry discharge (16 results) D ['11406590'], rhoptry discharge (16 results) S ['18678671'], Toxoplasma gondii rhoptry discharge (14 results) D ['11406590'], Toxoplasma gondii rhoptry discharge (14 results) S ['18678671'], growth response host (8899 results) S ['21479245'], gondii rhoptry discharge correlates (1 results) S ['18678671'], regulatory factors control parathyroid (45 results) D ['20562104'], exchanger regulatory factors control (28 results) D ['20562104'], discharge correlates with activation (35 results) S ['18678671'], early growth response host (1078 results) S ['21479245'], growth response host cell (6747 results) S ['21479245'], cell transcription factor (309 results) S ['21479245'], gondii rhoptry discharge (14 results) D ['11406590'], gondii rhoptry discharge (14 results) S ['18678671']
JE	TRUE	1	TRUE	globe threatens (3 results) D ['19587236'], threatens coastal (10 results) D ['19587236'], threatens coastal ecosystems (5 results) D ['19587236'], globe threatens coastal ecosystems (1 results) D ['19587236'], seagrasses (118 results) S ['21479244'], globe threatens coastal (1 results) D ['19587236'], loss of seagrasses (9 results) D ['19587236'], loss of seagrasses (9 results) S ['21479244']
JE	TRUE	0	FALSE	7-Day PAR among women (14 results) D ['16579852'], 7-Day PAR among women (14 results) S ['18498623'], physical activity advice (50 results) S ['19364999'], activity advice (54 results) S ['19364999'], consensus sessions between clinicians (24 results) S ['19534832', '18854033']
JE	TRUE	4	TRUE	VAMP3 null mice (2 results) D ['11238894'], VAMP3 null mice display (1 results) D ['11238894'], SNAP-25 (1255 results) S ['21479242'], SNAP-23 (162 results) S ['21479242', '20118925'], role for SNAP-23 (74 results) S ['21479242', '20118925'], neuronal role for SNAP-23 (6 results) S ['20118925'], t-SNARE SNAP-25 (9 results) D ['11753414'], VAMP3 null (3 results) D ['11238894'], adipocyte target SNARE (22 results) D ['9668052'], function of SNAP-25 (1178 results) S ['21479242'], t-SNARE SNAP-25 distinguishes (2 results) D ['11753414'], SNAP-25 distinguishes mechanisms (2 results) D ['11753414'], SNARE (5688 results) S ['21479242']

PMC3066228	5	FALSE	9	FALSE		5	14	FALSE	14	TRUE	12	TRUE	12	TRU
PMC3066227	0	TRUE	8	FALSE		4	7	TRUE	4	TRUE	4	TRUE	4	TRU
PMC3066226	0	TRUE	18	FALSE		2	16	FALSE	16	TRUE	12	TRUE	10	TRU
PMC3066225	2	FALSE	16	FALSE		8	16	TRUE	11	TRUE	11	TRUE	9	TRU
PMC3066224	3	FALSE	9	FALSE		4	12	FALSE	12	TRUE	11	TRUE	10	TRU
PMC3066222	5	FALSE	9	FALSE		6	13	TRUE	11	TRUE	10	TRUE	9	TRU

JE	TRUE	8	TRUE	audiovisual training (9 results) D ['16860741'], audiovisual training (9 results) S ['21479241'], monaural (1582 results) S ['21479241'], monaural conditions (35 results) S ['21479241'], word recognition Joint (32 results) D ['17640266'], auditory practice (1380 results) S ['21479241'], feedback paradigm (27 results) S ['21479241'], visual feedback paradigm (284 results) S ['21479241'], spatial monaural (134 results) D ['20371808', '19539018'], spatial monaural (134 results) S ['21479241'], recognition Joint extraction (38 results) D ['17640266'], audiovisual effect (457 results) S ['21479241'], spatial hearing (111 results) D ['20371808'], spatial hearing (111 results) S ['21479241']
JE	TRUE	2	TRUE	Proteus (15023 results) S ['21479240'], analyze patterns of bias (99 results) S ['21479240'], significant findings (2012 results) S ['21479240'], potential excess (35 results) S ['18779388'], extreme contradictory estimates (3 results) S ['15878467'], contradictory estimates (3 results) S ['15878467'], Proteus phenomenon (3 results) S ['21479240', '15878467'], human genome epidemiology association (1 results) S ['18492284']
JE	TRUE	6	TRUE	sturgeon (1387 results) S ['21483472'], broodstocks (28 results) S ['21483472'], parent pairs (116 results) S ['21483472'], polyploid sturgeon species (11 results) S ['21483472'], possible parent-pairs (8 results) S ['21483472'], tetraploid sturgeon species (7 results) S ['21483472'], polyploid sturgeon (13 results) S ['21483472'], naccarii (25 results) S ['21483472', '14629343'], polyploid species (189 results) S ['21483472'], tetraploid Adriatic (1 results) S ['21483472'], parental allocation (8 results) S ['21483472'], parent-pairs (116 results) S ['21483472'], Adriatic (784 results) S ['21483472'], polyploid (3046 results) S ['21483472'], sturgeon species (39 results) S ['21483472', '14629343'], candidate breeders (45 results) S ['21483472'], Adriatic sturgeon (7 results) S ['21483472', '14629343'], tetraploid Adriatic sturgeon Acipenser (1 results) S ['21483472']
JE	TRUE	8	TRUE	ribosome entry (1486 results) S ['21479239'], inhibitory effect of IRNA (5 results) S ['21479239'], Giardia (5713 results) S ['21479239'], ribosome entry site (1190 results) S ['21479239'], Giardiavirus (41 results) S ['21479239', '19826476', '14615487', '12022227'], internal ribosome entry site (1185 results) S ['21479239'], poliovirus (13738 results) S ['21479239'], GlLa (1 results) S ['21479239'], internal ribosome (1505 results) S ['21479239'], GLV-IRES (3 results) S ['21479239', '19826476'], parasitic protozoan Giardia lamblia (19 results) D ['10613698'], parasitic protozoan Giardia lamblia (19 results) S ['7651405'], Giardiavirus internal ribosome (6 results) S ['21479239', '19826476', '14615487', '12022227'], entry site (2327 results) S ['21479239'], Giardiavirus internal ribosome entry (5 results) S ['21479239', '19826476', '14615487', '12022227'], small yeast RNA (5 results) D ['8627683', '7933102'], GlLa protein (1 results) S ['21479239'], internal ribosome entry (1468 results) S ['21479239']
JE	TRUE	9	TRUE	promising liver specimen (19 results) D ['18201288'], liver specimen (210 results) D ['20677288'], human liver tissue (390 results) D ['20645046'], human liver tissue (390 results) S ['21479238'], human hepatocytes (2900 results) S ['21479238'], Isolation of primary (30305 results) S ['21479238'], normal donor tissue surplus (9 results) S ['21479238'], donor tissue surplus (44 results) S ['21479238'], liver tissue (11724 results) S ['21479238'], donor liver (3309 results) S ['21479238'], primary human hepatocytes (582 results) S ['21479238'], cell isolation from normal (19709 results) S ['21479238']
JE	TRUE	8	TRUE	Tuberculin skin test size (1 results) D ['17705981'], T-cell assay conversions (9 results) D ['19105884'], full-blown tuberculosis disease (18 results) D ['17352104'], skin test (9154 results) S ['21479236'], tuberculin skin test (1952 results) S ['21479236'], Predictive factors for latent (266 results) S ['21333099'], tuberculosis disease (344 results) S ['21479236'], SATVI study (3 results) S ['21333099'], reversions among household (3 results) D ['19105884'], tuberculin skin (2753 results) S ['21479236'], tuberculosis infection among adolescents (6701 results) S ['21333099'], high-burden (950 results) S ['21479236'], prognostic value of IFN-gamma (45 results) D ['20463900'], high-burden area (504 results) S ['21479236', '21333099']

PMC3066221	3	FALSE	9	FALSE		2	12	TRUE	11	TRUE	11	TRUE	8	TRU
PMC3066220	14	FALSE	9	FALSE		9	21	TRUE	15	TRUE	14	TRUE	14	TRU
PMC3066219	2	FALSE	18	FALSE		5	20	TRUE	19	TRUE	15	TRUE	14	TRU
PMC3066218	11	FALSE	1	FALSE		10	11	TRUE	5	TRUE	5	TRUE	3	TRU
PMC3066217	2	FALSE	0	TRUE		2	2	TRUE	1	TRUE	1	TRUE	1	TRU
PMC3066216	6	FALSE	6	FALSE		3	9	FALSE	9	TRUE	7	TRUE	5	TRU

JE	TRUE	5	TRUE	subregional comparisons (15 results) D ['17684557'], Jokiel (47 results) S ['21479235'], coral adaptation capabilities (5 results) S ['21479235'], examine rates of coral (31 results) S ['21479235'], mass coral (561 results) S ['21479235'], acidification (12091 results) S ['21479235'], surface temperature (1991 results) S ['21479235'], coral cover (104 results) S ['21479235'], coral growth (30 results) S ['21479235'], Regional decline (15 results) D ['17684557'], decline of coral (153 results) S ['21479235'], Regional decline of coral (11 results) D ['17684557']
JE	TRUE	8	TRUE	BRAF (2198 results) S ['21479234'], V600E (527 results) S ['21479234'], pleomorphic xanthoastrocytoma with anaplastic (61 results) D ['20582976'], pleomorphic xanthoastrocytoma with anaplastic (61 results) S ['19164434'], Pediatric cerebellar pleomorphic (3 results) D ['16570197'], pleomorphic (10856 results) S ['21479234'], meningocerebral glioma (9 results) D ['498051'], Pediatric cerebellar pleomorphic xanthoastrocytoma (2 results) D ['16570197'], clinical platform (21 results) S ['20432502'], TSC1 mRNA expression (2 results) D ['16909113'], pleomorphic xanthoastrocytoma (260 results) D ['20582976'], pleomorphic xanthoastrocytoma (260 results) S ['21479234'], distinctive meningocerebral (2 results) D ['498051'], BRAF mutation (488 results) S ['21479234'], BRAF-selective manner (1 results) D ['20668238'], xanthoastrocytoma (270 results) D ['20582976'], xanthoastrocytoma (270 results) S ['21479234'], distinctive meningocerebral glioma (2 results) D ['498051'], Oncogenic RAF1 rearrangement (2 results) D ['19363522'], V600E BRAF-selective manner (1 results) D ['20668238'], Oncogenic RAF1 (29 results) D ['19363522'], xanthoastrocytoma with anaplastic (62 results) D ['20582976'], xanthoastrocytoma with anaplastic (62 results) S ['19164434']
JE	TRUE	10	TRUE	Dimerization of tetherin (4 results) S ['19742323'], activity against Lassa (23 results) S ['19742323'], Vpu (637 results) D ['20980512'], Vpu (637 results) S ['21479233'], RD-114 virus (50 results) S ['20631117'], antiviral activity against Lassa (18 results) S ['19742323'], tetherin (107 results) S ['21479233'], feline against RD-114 (9 results) S ['21479233'], CRFK (194 results) S ['20631117'], antiviral activity (8309 results) S ['21479233'], interaction with alpha-adaptin (133 results) D ['19359243'], RD-114 virus release (7 results) S ['21479233'], glycosylation of feline (107 results) S ['21479233'], RD-114 release (7 results) S ['21479233'], RD-114 from cells (81 results) S ['20631117'], antiviral activity of feline (140 results) S ['21479233'], feline homolog (8 results) S ['21479233'], activity of feline (18399 results) S ['21479233'], RD-114 (132 results) S ['21479233', '20631117'], RD-114 infectious (22 results) S ['21479233', '20631117']
JE	TRUE	1	TRUE	Optimal Anatomic Site (5 results) D ['17955432'], papillomavirus gene (22 results) D ['8174743'], Human papillomavirus gene sequences (3 results) D ['8174743'], human sperm (3675 results) S ['21479232'], Human papillomavirus gene (13 results) D ['8174743'], papillomavirus gene sequences (3 results) D ['8174743'], HPV seminal (17 results) D ['17442614', '15537460', '10560983', '8612829'], papillomavirus DNA from sperm (37 results) D ['17433312', '15537460', '12051883', '11349159', '10560983'], human sperm deoxyribonucleic (5 results) D ['11349159', '8174743'], asymptomatic male infertility (2 results) D ['17433312'], human sperm deoxyribonucleic acid (5 results) D ['11349159', '8174743'], asymptomatic male infertility patients (1 results) D ['17433312']
JE	TRUE	0	FALSE	MMP-2 cleavage of vitronectin (2 results) D ['18340378'], different histotypes of epithelial (34 results) D ['16144910']
JE	TRUE	4	TRUE	impairs alveolar differentiation (18 results) D ['17145767'], luminal MECs (23 results) S ['21479230'], mammary epithelial (5321 results) S ['21479230'], primary MECs (9 results) S ['21479230'], correct three-dimensional heterotypic (1 results) D ['12798139'], three-dimensional heterotypic collagen assays (1 results) D ['12798139'], MECs (427 results) S ['21479230'], three-dimensional heterotypic collagen (11 results) D ['12798139'], mammary epithelial basement membrane (518 results) S ['21479230'], heterotypic collagen assays (7 results) D ['12798139'], correct three-dimensional heterotypic collagen (1 results) D ['12798139'], mammary epithelial basement (549 results) S ['21479230']

PMC3066215	8	FALSE	5	FALSE		5	10	TRUE	8	TRUE	6	TRUE	4	TRU
PMC3066214	6	FALSE	8	FALSE		11	12	TRUE	10	TRUE	10	TRUE	10	TRU
PMC3066213	7	FALSE	8	FALSE		7	14	TRUE	10	TRUE	7	TRUE	7	TRU
PMC3066212	8	FALSE	14	FALSE		12	17	TRUE	11	TRUE	10	TRUE	9	TRU
PMC3066210	8	FALSE	2	FALSE		5	7	TRUE	6	TRUE	5	TRUE	4	TRU
PMC3066208	0	TRUE	11	FALSE		2	11	TRUE	10	TRUE	10	TRUE	10	TRU

JE	TRUE	2	TRUE	substrate vibrations (21 results) D ['18032004'], Papaj DR Patterns (4 results) D ['19302028'], Hollis (1892 results) S ['21479229'], sedentary insect antlions (2 results) S ['21479229'], prey for Random (168 results) S ['21479229'], antlion larva (9 results) D ['18032004', '16943491'], Random antlions (1 results) S ['21479229'], antlions (12 results) D ['16943491'], antlions (12 results) S ['21479229'], larva Euroleon (1 results) D ['18032004'], antlion larva Euroleon (1 results) D ['18032004'], fitness costs of learning (33 results) D ['19549495', '19302028'], pit-building (7 results) D ['18032004']
JE	TRUE	9	TRUE	spinocerebellar (4372 results) S ['21479228'], SCA2 trinucleotide repeat (201 results) D ['19676102', '19672991'], spinocerebellar ataxia type (1112 results) S ['21479228'], AG-09215 AG-10124 (28 results) S ['20740007'], CAA interruptions (11 results) D ['19676102', '18990604', '18043721', '17149720', '15533937', '11689490', '8896555'], CAG (5983 results) S ['21479228'], SCA2 trinucleotide repeat expansion (139 results) D ['19676102', '19672991'], 1R01NS065317-01 (3 results) S ['20740007'], ataxia type (1320 results) S ['21479228'], SCA2 trinucleotide (204 results) D ['19676102', '19672991'], dominant cerebellar ataxia families (1 results) D ['9549522'], CAG repeat (1891 results) S ['21479228'], spinocerebellar ataxia (1851 results) S ['21479228'], cerebellar ataxia families (1 results) D ['9549522']
JE	TRUE	4	TRUE	reaction-diffusion dynamics (23 results) S ['21479227'], Arabidopsis isopentenyltransferases (7 results) D ['17062755'], SAM pattern formation (184 results) S ['21479227'], WUS-CLV dynamics (1 results) S ['21479227'], wus mutant (36 results) S ['21479227'], meristem (3785 results) S ['21479227'], intercellular movement of CLAVATA3 (2 results) D ['12783788'], Arabidopsis shoot (84 results) D ['20169148', '19717465'], feedback loops through cytokinin (4 results) D ['19717465'], reaction-diffusion (1691 results) S ['21479227'], WUS-CLV (3 results) S ['21479227'], apical meristem (1043 results) S ['21479227'], loops through cytokinin (8 results) D ['19717465'], movement of CLAVATA3 (2 results) D ['12783788'], Arabidopsis shoot meristem (10 results) D ['19717465', '12783788', '9865698', '8565856']
JE	TRUE	5	TRUE	moricin family (3 results) D ['16115804'], moricin family (3 results) S ['21479226'], antimicrobial peptide (2854 results) S ['21479226'], family moricin (3 results) D ['16115804'], family moricin (3 results) S ['21479226'], cecropin family moricin (1 results) S ['21479226'], BmcecB6 (1 results) S ['21479226'], gloverin (23 results) D ['20490430', '18524767', '18076111', '12590972'], gloverin (23 results) S ['21479226', '16406194'], gloverin family (3 results) D ['18524767'], gloverin family (3 results) S ['21479226'], inductive expression (26 results) S ['16406194'], silkworm (2768 results) S ['21479226'], moricin (23 results) D ['18265434', '16115804', '11997013'], moricin (23 results) S ['21479226', '16406194'], Drosophila host differential induction (6 results) D ['9405661'], cecropin (551 results) S ['21479226'], BmcecD (1 results) S ['21479226'], major effector genes (1262 results) S ['21479226'], drosomycin gene family (1 results) S ['19888430'], gloverin family of antibacterial (1 results) D ['18524767'], sequences for cecropin (168 results) D ['19107394']
JE	TRUE	2	TRUE	cytosolic chaperone for hamartin (1 results) D ['10585443'], chaperone for hamartin (8 results) D ['10585443'], cytosolic tuberin-hamartin complex (1 results) D ['10585443'], mTORC1 (691 results) S ['21479224'], rapamycin regulates lipid (70 results) D ['19963098'], Tsc1 (761 results) S ['21479224'], rapamycin reduces adiposity (2 results) D ['19496779'], tuberin-hamartin complex (17 results) D ['10585443'], rapamycin regulates lipid metabolism (31 results) D ['19963098', '18762023'], cytosolic tuberin-hamartin (1 results) D ['10585443']
JE	TRUE	9	TRUE	direct PKG phosphorylation (49 results) S ['21479273'], cGMP-dependent protein (1740 results) S ['21479273'], ATP-sensitive channel activation (1396 results) S ['21479273'], rabbit ventricular (6060 results) S ['21479273'], ATP channels (2247 results) S ['21479273'], neuronal KATP (106 results) S ['20053925'], cGMP-dependent (2590 results) S ['21479273'], cGMP-dependent protein involvement (158 results) S ['20053925'], neuronal KATP channels (4 results) S ['20053925'], ATP-sensitive potassium (2200 results) S ['21479273'], ATP-sensitive (5391 results) S ['21479273']

PMC3066207	5	FALSE	4	FALSE		3	9	FALSE	9	TRUE	7	TRUE	6	TRU
PMC3066205	6	FALSE	11	FALSE		7	17	TRUE	14	TRUE	13	TRUE	10	TRU
PMC3066204	3	FALSE	6	FALSE		6	7	TRUE	6	TRUE	4	TRUE	3	TRU
PMC3066203	6	FALSE	13	FALSE		13	15	TRUE	12	TRUE	9	TRUE	7	TRU
PMC3066202	13	FALSE	5	FALSE		7	14	TRUE	12	TRUE	10	TRUE	5	TRU

JE	TRUE	4	TRUE	hypertension severity (75 results) S ['21479272'], hypertension among psoriasis (316 results) S ['21479272'], systemic inflammatory potential mechanistic (55 results) D ['20445552'], potential mechanistic links (10 results) D ['20445552'], association between psoriasis (1530 results) S ['21479272'], psoriasis patients (1303 results) S ['21479272'], inflammatory potential mechanistic links (18 results) D ['20445552'], mechanistic links between skin (9 results) D ['20445552'], Italian psoriasis (489 results) D ['19220983']
JE	TRUE	6	TRUE	gld allele (28 results) S ['14764677'], ligand engagement of resident (17 results) S ['11714783'], factor-alpha mediates oligodendrocyte death (5 results) D ['17151265'], factor-alpha mediates oligodendrocyte (11 results) D ['17151265'], soluble Fas ligand (350 results) S ['21479271'], soluble Fas ligand expression (4 results) D ['10611715'], soluble Fas ligand expression (4 results) S ['10748238'], gene causes massive lymphoproliferation (40 results) S ['14764677'], activity of gld (182 results) D ['19794494'], retinal ganglion (10724 results) S ['21479271'], Fas ligand (7935 results) S ['21479271'], necrosis factor-alpha mediates oligodendrocyte (11 results) D ['17151265'], residual activity of gld (6 results) S ['14764677'], mediates oligodendrocyte death (35 results) D ['17151265'], retinal ganglion cell (2533 results) S ['21479271'], causes massive lymphoproliferation (76 results) S ['14764677'], ganglion cell (6929 results) S ['21479271']
JE	TRUE	2	TRUE	yellow super fluorescent proteins (7 results) S ['16716067'], yellow super fluorescent (8 results) S ['16716067'], FRET Forster (638 results) S ['21479270'], mCerulean sequence (1 results) D ['20081836'], cell-biological applications (23 results) D ['11753368'], efficient maturation for cell-biological (3 results) D ['11753368'], DK077140 (1 results) S ['19934346'], Optimization of pairings (14 results) S ['17481360'], FRET between cyan (357 results) S ['21479270']
JE	TRUE	3	TRUE	Novel mutations of MYO15A (11 results) D ['19274735', '17546645', '11735029'], segment of RNO10 (7 results) S ['14629112'], Usher molecular links (13 results) D ['16987892', '11701652'], mutations of MYO15A (23 results) D ['19274735', '17546645', '11735029', '10552926'], deafblind LEW-ci2 (1 results) S ['14629112'], Review Usher molecular links (4 results) D ['16987892', '11701652'], Usher syndrome (582 results) S ['21479269'], human Usher syndrome (22 results) S ['21479269'], deafblind LEW-ci2 rat (1 results) S ['14629112'], ci2 rat (8 results) S ['21479269', '14629112', '11683905'], mutagenesis-driven (2 results) S ['15965244'], LEW-ci2 (1 results) S ['14629112'], model for usher (158 results) S ['21479269'], human Usher (1210 results) S ['21479269'], LEW-ci2 rat (1 results) S ['14629112'], rat model for usher (6 results) S ['21479269', '15965244'], whirlin (28 results) D ['17171570'], Myo15 (34 results) D ['15654330', '15590698', '11735029'], Myo15 (34 results) S ['21479269', '14629112']
JE	TRUE	4	TRUE	ammonium cations exert (19 results) D ['18519726'], ammonium cations exert synergistic (1 results) D ['18519726'], peptide nucleic acid probe (34 results) S ['20453122', '19270117'], quaternary ammonium cations exert (7 results) D ['18519726'], five-colour multiplex (1 results) D ['17446294'], cations exert synergistic (8 results) D ['18519726'], nucleic acid probe (192 results) S ['20453122'], characterization of subject-specific (12 results) D ['16597990'], five-colour multiplex fluorescence (1 results) D ['17446294'], cations exert synergistic bactericidal (1 results) D ['18519726'], peptide nucleic (1904 results) S ['21479268'], vivo dynamics of Streptococcus (15 results) D ['17446294'], acid probe (310 results) S ['20453122'], nontypical Pseudomonas aeruginosa (2 results) D ['16081953'], exert synergistic bactericidal (13 results) D ['18519726'], nontypical Pseudomonas (5 results) D ['16081953'], peptide nucleic acid (1114 results) S ['21479268'], antibiofilm activity against Pseudomonas (15 results) D ['18519726']

PMC3066201	10	FALSE	4	FALSE		6	11	TRUE	9	TRUE	9	TRUE	7	TRU
PMC3066194	1	FALSE	11	FALSE		4	9	TRUE	8	TRUE	8	TRUE	8	TRU
PMC3066192	4	FALSE	9	FALSE		6	13	FALSE	13	TRUE	9	TRUE	9	TRU
PMC3066189	13	FALSE	6	FALSE		7	16	TRUE	11	TRUE	10	TRUE	8	TRU
PMC3066176	1	FALSE	11	FALSE		2	11	TRUE	10	TRUE	9	TRUE	8	TRU
PMC3066171	4	FALSE	11	FALSE		8	13	TRUE	12	TRUE	11	TRUE	10	TRU

JE	TRUE	5	TRUE	gastropod mollusc Pleurobranchaea japonica (3 results) D ['10805085'], statocyst (176 results) S ['21479267'], gastropod mollusc Pleurobranchaea (42 results) D ['10805085'], reflex of tadpoles (152 results) D ['18666444'], histamine-like immunoreactive sensory hair (1 results) D ['10805085'], arrangement of FMRFamide- (22 results) D ['10805085'], Mosaic arrangement of FMRFamide- (1 results) D ['10805085'], mollusc Pleurobranchaea japonica (1 results) D ['10805085'], histamine-like immunoreactive sensory (2 results) D ['10805085'], return from space (1706 results) S ['21479267'], vestibulo-ocular reflex of tadpoles (20 results) D ['18666444', '16857868', '9792238', '8951390'], postflight (606 results) S ['21479267'], hair cells (9707 results) S ['21479267'], histamine-like immunoreactive (17 results) D ['10805085']
JE	TRUE	5	TRUE	potassium channel KCNC3 (35 results) S ['21479265', '19953606'], substitutions of Ser-906 (1 results) D ['12898257'], Spinocerebellar ataxia (1851 results) S ['21479265'], Spinocerebellar (4372 results) S ['21479265'], Gly263Asp (1 results) S ['21479265'], channel KCNC3 (28 results) S ['21479265', '19953606', '16501573'], CAG (5983 results) S ['21479265'], KCNC3 Gly263Asp (1 results) S ['21479265'], KCNC3 (38 results) S ['21479265', '19953606'], potassium channel (16035 results) S ['21479265'], central nervous system phenotypes (3 results) S ['16501573'], CAG repeat (1891 results) S ['21479265']
JE	TRUE	2	TRUE	Widespread underrecognition (6 results) D ['17288506'], proportion of overtreatment (55 results) S ['21479264'], Netherlands Study of Depression (3072 results) S ['21479264'], correlates of non-response (23 results) S ['20027601'], undertreatment of anxiety (40 results) D ['17288506'], undertreatment of anxiety (40 results) S ['21479264'], Life-Chart method manual (9 results) S ['11311838'], Scottish primary-care setting (71 results) D ['19761665'], overtreatment (1413 results) S ['21479264'], Antidepressant utilisation patterns (8 results) D ['18276016'], focus on overtreatment (38 results) S ['21479264'], overtreatment with antidepressants (9 results) S ['21479264'], Netherlands Study (94 results) S ['21479264']
JE	TRUE	4	TRUE	Janzen-Connell effects (6 results) D ['18831160'], alien Ammophila arenaria (2 results) D ['15309610'], widespread plant-mycorrhizal pines (1 results) D ['17465924'], Janzen-Connell (23 results) D ['18831160'], widespread plant-mycorrhizal (1 results) D ['17465924'], plant-mycorrhizal pines (1 results) D ['17465924'], false truffles (4 results) D ['17465924'], soil biota (87 results) S ['21479262'], Plant-soil (321 results) S ['21479262'], local adaptation (833 results) S ['21479262'], alien Ammophila (2 results) D ['15309610'], Plant pathogens drive density-dependent (3 results) D ['16643302'], soil pathogens (19 results) D ['18831160', '15309610', '10749209'], biota (3073 results) S ['21479262'], soil biota effects (221 results) S ['21479262'], native root herbivores (12 results) D ['15309610', '10749209'], Negative Soil Biota (25 results) D ['14973484'], Negative Soil Biota (25 results) S ['21479262'], pathogens drive density-dependent (4 results) D ['16643302']
JE	TRUE	6	TRUE	catalytic residues (1408 results) S ['21468322'], prediction model (2043 results) S ['21468322'], 10fold (27 results) S ['21468322'], correlation between residues (5035 results) S ['21468322'], noncatalytic residues (6 results) D ['17189479'], prediction model for catalytic (262 results) S ['21468322'], protein structure network (11 results) S ['21468322'], structurefunction relationship (2 results) S ['21468322'], correlations among catalytic (460 results) S ['21468322'], structure network (36 results) S ['21468322'], 10fold crossvalidation (1 results) S ['21468322'], crossvalidation (134 results) S ['21468322']
JE	TRUE	5	TRUE	T4SS (158 results) S ['21468321'], molecular function (1182 results) S ['21468321'], terminology across bacterial (13 results) S ['21468321'], Protein subassemblies (6 results) D ['18178731'], machine learning (2820 results) S ['21468321'], entity recognition (68 results) D ['19656727'], entity recognition (68 results) S ['21468321'], T4SS documents (1 results) S ['21468321'], entity classes (3 results) D ['17090325'], T4SS literature (1 results) S ['21468321'], T4SSs (31 results) D ['19756009', '17158676', '17122343'], T4SSs (31 results) S ['19279686'], secretion systems (891 results) S ['21468321'], cellular components (4813 results) S ['21468321'], Ananiadou (39 results) S ['21468321']

PMC3066169	0	TRUE	7	FALSE		1	7	FALSE	7	TRUE	6	TRUE	5	TRU
PMC3066165	2	FALSE	14	FALSE		7	16	TRUE	12	TRUE	11	TRUE	10	TRU
PMC3066163	18	FALSE	8	FALSE		8	22	TRUE	21	TRUE	18	TRUE	13	TRU
PMC3072940	7	FALSE	15	FALSE		10	19	TRUE	18	TRUE	15	TRUE	11	TRU

JE	TRUE	4	TRUE	Fura-2AM measurements (125 results) S ['21483471'], rat cortical astrocytes (294 results) S ['21483471'], rat cortical (3260 results) S ['21483471'], Cortical Fura-2AM Measurements (10 results) S ['21483471'], cortical astrocytes (886 results) S ['21483471'], experimental Fura-2AM measurements (13 results) S ['21483471'], Rat Cortical Fura-2AM (64 results) S ['21483471']
JE	TRUE	4	TRUE	German Research (1245 results) S ['21468319'], Neuropathic Pain somatosensory (422 results) S ['21468319'], Neuropathic Pain somatosensory abnormalities (31 results) S ['21468319', '20627413'], colocalization with trk (21 results) D ['16304633'], different neuropathic pain syndromes (5 results) S ['20627413'], Network on Neuropathic (100 results) S ['21468319', '20627413'], Pain somatosensory abnormalities (167 results) S ['21468319', '20627413'], QST parameters (7 results) S ['16697110'], supraspinal integration of temperature (5 results) S ['8931593'], postherpetic Differences (72 results) S ['19592166'], German Research Network (53 results) S ['21468319', '20627413'], neuropathic pain patients (89 results) S ['21468319'], novel receptors TRPV3 (5 results) D ['17521436'], different neuropathic pain (11 results) S ['20627413'], pain patients (4213 results) S ['21468319'], supraspinal integration (3 results) S ['8931593']
JE	TRUE	4	TRUE	cellular traction (38 results) S ['21468318', '20018765'], Exert 3-Dimensional (20 results) D ['19779633'], traction force microscopy (44 results) D ['20717570'], traction force microscopy (44 results) S ['21468318'], three-dimensional traction force vector (3 results) D ['15908579'], Cell Traction Image (38 results) D ['19779633', '17827246'], Cells Exert 3-Dimensional (10 results) D ['19779633'], Exert 3-Dimensional Traction Forces (1 results) D ['19779633'], three-dimensional traction vector (7 results) D ['15908579'], 3-Dimensional Traction Forces (6 results) D ['19779633'], locomotion of fibroblasts (4575 results) S ['21468318'], High resolution traction force (12 results) D ['17827246'], traction force microscopy technique (18 results) D ['19779633', '17660320'], traction force microscopy technique (18 results) S ['21468318'], three-dimensional traction force (59 results) D ['21076420', '20717570', '19779633'], three-dimensional traction force (59 results) S ['21468318', '20018765'], Live Cells Exert 3-Dimensional (1 results) D ['19779633'], traction force (318 results) S ['21468318'], three-dimensional traction (205 results) D ['21076420', '20717570'], three-dimensional traction (205 results) S ['21468318'], Cells Exert 3-Dimensional Traction (1 results) D ['19779633'], cellular traction forces (22 results) S ['21468318', '20018765'], traction force vector (29 results) D ['15908579'], 3-Dimensional Traction (41 results) D ['19779633'], resolution traction force microscopy (15 results) D ['17827246', '17660320'], Exert 3-Dimensional Traction (1 results) D ['19779633']
JE	TRUE	6	TRUE	marker genes MATER (31 results) D ['15189828'], early embryonic (4791 results) S ['21447182'], nucleoplasmin (239 results) S ['21447182', '19420384'], bovine NPM2 (1 results) S ['21447182'], oocyte-specific gene (24 results) S ['17978182'], importin alpha8 (1 results) S ['19420384'], NPM2 (20 results) D ['17594605', '17044029', '16075464', '12714744'], NPM2 (20 results) S ['21447182'], intracellular localization throughout folliculogenesis (7 results) D ['16753072'], early embryogenesis (2409 results) S ['21447182'], miRNA-181a (7 results) D ['20591824'], miRNA-181a (7 results) S ['21447182'], NPM2 protein (17 results) D ['17594605', '17044029', '16075464', '12714744'], NPM2 protein (17 results) S ['21447182'], NPM2 cDNA (2 results) S ['21447182'], early embryonic development (1796 results) S ['21447182'], cell marker genes MATER (28 results) D ['15189828'], bovine NPM2 protein (1 results) S ['21447182'], oocyte-specific (327 results) S ['21447182'], maternal effect (1028 results) S ['21447182'], localization throughout folliculogenesis (24 results) D ['16753072'], miR-181a (63 results) S ['21447182']

PMC3077333	8	FALSE	17	FALSE		15	24	FALSE	24	TRUE	21	TRUE	18	TRU
PMC3072136	0	TRUE	12	FALSE		3	10	FALSE	10	TRUE	7	TRUE	6	TRU
PMC3073940	4	FALSE	10	FALSE		10	14	TRUE	11	TRUE	8	TRUE	5	TRU
PMC3078833	0	TRUE	21	FALSE		4	18	TRUE	13	TRUE	9	TRUE	9	TRU
PMC3073923	3	FALSE	7	FALSE		3	8	TRUE	7	TRUE	7	TRUE	7	TRU

JE	TRUE	10	TRUE	XMRV infection with PCa (1 results) S ['21447170'], MLV-related (21 results) D ['21171978', '20878949', '20798047', '18823818', '10196331'], MLV-related (21 results) S ['21447170'], xenotropic (676 results) S ['21447170'], xenotropic murine (198 results) S ['21447170'], XMRV infection (30 results) D ['21203514', '21171978', '20878949', '20836869', '20716359'], XMRV infection (30 results) S ['21447170', '21084477'], infection with PCa (288 results) S ['21447170'], virus gene sequences (15 results) D ['20798047'], murine leukemia (12139 results) S ['21447170'], fatigue syndrome (4554 results) S ['21447170'], XMRV (109 results) S ['21447170'], RNASEL (107 results) D ['20371060'], RNASEL (107 results) S ['21447170'], RNASEL R462Q mutation (7 results) D ['19835577'], RNASEL R462Q mutation (7 results) S ['21447170'], malignant prostatic epithelium (39 results) D ['19805305'], malignant prostatic epithelium (39 results) S ['21447170'], XMRV antigens (11 results) S ['21447170', '21084477'], xenotropic murine leukemia (167 results) S ['21447170'], R462Q (23 results) D ['20371060', '19835577', '18823818', '16609730'], R462Q (23 results) S ['21447170'], prevalence of XMRV (28 results) D ['21171966', '20878949', '20716359'], prevalence of XMRV (28 results) S ['21447170'], chronic fatigue syndrome (4406 results) S ['21447170']
JE	TRUE	4	TRUE	microendoscopy (106 results) S ['21483619'], novel Peptide probe (6 results) S ['21408169'], target peptide QPIHPNNM (1 results) S ['21408169'], endoscopic detection (142 results) S ['21408169'], Kras mouse model (133 results) S ['21408169'], microendoscope (90 results) S ['21483619', '21136184'], mucosa on microendoscopy (10 results) S ['21483619'], endoscopic detection of colon (936 results) S ['21408169'], Peptide probe (74 results) S ['21408169'], Kras mice (8 results) S ['21408169'], QPIHPNNM (2 results) S ['21483619', '21408169'], colon dysplasia (13 results) S ['21408169']
JE	TRUE	4	TRUE	anti-tumor activity (2526 results) S ['21443770'], Sindbis (2589 results) S ['21443770'], mixture of PA63 (2 results) S ['18194342'], virus nsP4 (8 results) D ['19036396', '15197279'], resultant anti-tumor (39 results) S ['19013656', '18094613'], Sindbis virus nsP4 (7 results) D ['19036396', '15197279'], resultant anti-tumor activity (19 results) S ['19013656', '18094613'], synthetic dsRNA (123 results) S ['21443770'], replicase-based (13 results) D ['15063579'], replicase-based (13 results) S ['21443770', '16988457'], dsRNA into tumors (993 results) S ['21443770'], polyinosinic-polycytidylic liposome (8 results) D ['17109465', '10485480'], Tumor chemo-immunotherapy (3 results) S ['18094613'], CA135274 (2 results) S ['19013656']
JE	TRUE	3	TRUE	slavemaker nests (1 results) S ['11308089'], slavemaker densities (1 results) S ['21443778'], slavemakers (5 results) S ['21443778', '11308089'], slavemaker demography (3 results) S ['21443778', '11308089'], host colonies (24 results) S ['21443778', '19537541', '16499708'], parasitism risk (10 results) S ['21443778'], relative influence of parasitism (52 results) S ['21443778'], Bavarian community (42 results) S ['21443778'], nest sites (166 results) S ['21443778'], colony demography (10 results) S ['21443778', '19537541'], slavemaker colonies (6 results) S ['21443778', '11308089'], spatial pattern of colonies (69 results) S ['21443778'], slavemaking (6 results) S ['21443778', '11308089'], slavemaker (7 results) S ['21443778', '11308089'], parasitism (4711 results) S ['21443778'], brood production (32 results) S ['21443778'], slave-raids (8 results) S ['16499708', '11308089'], nest site size (82 results) S ['21443778'], spatial pattern (2225 results) S ['21443778'], slavemaking-host (1 results) S ['21443778'], host on slavemaker (5 results) S ['21443778', '11308089']
JE	TRUE	2	TRUE	non-viral transfection of PC12 (5 results) D ['15763169'], On-chip transfection of PC12 (1 results) D ['15763169'], multi-parametric phenotypic readouts (1 results) S ['21443765'], cell microarrays (84 results) S ['21443765'], functional genome-wide RNAi screen (37 results) D ['18684994'], cell spot (7155 results) S ['21443765'], CSMA cell (33 results) S ['21443765'], CSMA method (26 results) S ['21443765'], CSMA (89 results) S ['21443765'], microarray method (171 results) S ['21443765']

PMC3078842	5	FALSE	13	FALSE		3	18	TRUE	9	TRUE	9	TRUE	7	TRU
PMC3074541	2	FALSE	7	FALSE		3	7	FALSE	7	FALSE	7	FALSE	7	TRU
PMC3073876	1	FALSE	6	FALSE		2	7	TRUE	5	TRUE	5	TRUE	4	TRU
PMC3072303	0	TRUE	12	FALSE		4	12	TRUE	10	TRUE	9	TRUE	8	TRU
PMC3078097	2	FALSE	9	FALSE		2	11	FALSE	11	TRUE	10	TRUE	10	TRU
PMC3073903	0	TRUE	15	FALSE		1	15	FALSE	15	TRUE	14	TRUE	12	TRU

JE	TRUE	7	TRUE	man-labour (3 results) S ['21443801'], number of discharges (2221 results) S ['21443801'], sales of antidepressants (204 results) S ['21443801'], man-labour years (2 results) S ['21443801'], female suicide mortality (5 results) S ['21443801'], density of psychotherapists (4 results) D ['19076117'], Norwegian mental health (13 results) D ['20035623'], Norwegian mental health (13 results) S ['21443801'], Norwegian mental health institutions (3 results) D ['20035623'], Norwegian mental (1732 results) S ['21443801'], Norwegian mental health services (3 results) D ['20035623'], Norwegian mental health services (3 results) S ['21443801'], suicide mortality (369 results) S ['21443801'], trans-institutionalisation (3 results) D ['20035623'], suicide risk (1836 results) S ['21443801'], ecological study (1112 results) S ['21443801'], excess suicide risk (5 results) S ['21443801'], outpatient consultations (204 results) S ['21443801']
JE	TRUE	6	TRUE	Hsp70 decreases with aging (28 results) S ['21443787', '17211576'], Hsp70 levels (342 results) S ['21443787'], inflammatory geriatric patients (401 results) S ['21443787'], serum levels of Hsp70 (341 results) S ['21443787'], possible link between endocrinosenescence (1 results) D ['9626133'], Hsp70 decreases (279 results) S ['21443787'], link between endocrinosenescence (1 results) D ['9626133'], Hsp70 serum (717 results) S ['21443787'], levels of Hsp70 (3875 results) S ['21443787']
JE	TRUE	2	TRUE	sample of Nepalese (48 results) S ['21443802'], Nepalese migrant workers (11 results) S ['21443802'], Nepalese (594 results) S ['21443802'], Gulf countries (91 results) S ['21443802'], Government of Nepal (254 results) S ['21443802'], Nepalese migrants (3 results) D ['18500980'], Nepalese migrants (3 results) S ['21443802']
JE	TRUE	5	TRUE	obstructive coronary artery (640 results) S ['21443790'], expression test (37 results) S ['20921541'], CardioDx (6 results) S ['21443790', '20921541', '20031539'], meta-gene (24 results) S ['21443790'], gene expression test (13 results) S ['20921541'], PCR algorithm development (213 results) S ['21443790'], assessment of obstructive (6214 results) S ['21443790'], obstructive coronary artery disease (615 results) S ['21443790'], meta-genes (2 results) S ['21443790'], CATHGEN (9 results) S ['21443790', '17357071'], blood-based (468 results) S ['21443790'], Multicenter validation (84 results) S ['20921541']
JE	TRUE	5	TRUE	detention centre between May (102 results) S ['21443761'], chronic health problems (444 results) S ['21443761'], detention centre (50 results) S ['21443761'], geographical origin versus (50 results) D ['19582582'], care-seeking (1142 results) S ['21443761'], Asian migrants (73 results) S ['21443761'], health care among migrants (2647 results) S ['21443761'], origin versus legal status (10 results) D ['19582582'], undocumented (1293 results) S ['21443761'], denial of care (36 results) S ['21443761'], care seekers (39 results) S ['21443761']
JE	TRUE	9	TRUE	analysis approach (1564 results) S ['21443803'], Iranian women (190 results) S ['21443803'], random multistage cluster (8 results) S ['21443803'], sectors of Tehran (12 results) S ['21443803'], conventional qualitative content analysis (93 results) S ['21443803'], technique among experts (1463 results) S ['21443803'], qualitative content analysis approach (12 results) S ['21443803'], Iranian women of reproductive (198 results) S ['21443803'], strategies for Iranian (103 results) S ['21443803'], health-promoting (2494 results) S ['21443803'], quantitative results (2373 results) S ['21443803'], sequential explanatory (47 results) S ['21443803'], conventional qualitative content (118 results) S ['21443803'], group technique among experts (437 results) S ['21443803'], content analysis approach (74 results) S ['21443803']

PMC3072332	6	FALSE	30	FALSE		15	33	TRUE	30	TRUE	27	TRUE	20	TRU
PMC3076226	7	FALSE	9	FALSE		11	14	TRUE	12	TRUE	9	TRUE	7	TRU
PMC3072309	17	FALSE	11	FALSE		7	23	TRUE	19	TRUE	16	TRUE	9	TRU

JE	TRUE	15	TRUE	unisexual (347 results) D ['20687905', '20682056'], unisexual (347 results) S ['21439093', '20596906'], modes of unisexual (15 results) D ['18508756', '17546077'], modes of unisexual (15 results) S ['21439093', '20596906'], novel clone (58 results) S ['21439093'], various ploidy (59705 results) S ['21439093'], gibel carp (49 results) S ['21439093', '20596906'], reproduction modes (10 results) S ['21439093', '20596906', '11080373'], polyploid gibel (6 results) S ['21439093', '20596906', '17115331', '14566938', '12403177'], gynogenesis (156 results) S ['21439093', '20596906'], polyploid Carassius (32 results) S ['20596906', '17115331', '15014154'], novel clone strain (387 results) S ['21439093'], allogynogenesis (2 results) S ['14606628'], polyploid Carassius auratus gibelio (13 results) S ['20596906', '17115331', '15014154', '14566938', '12403177'], gonochoristic sexual reproduction (17 results) S ['21439093'], species-specific reproduction isolation (117 results) D ['20738851'], ploidy (8485 results) S ['21439093'], several unisexual (347 results) D ['20687905', '20682056'], several unisexual (347 results) S ['21439093', '20596906'], gynogenetic ability (13 results) D ['1570013'], gynogenetic ability (13 results) S ['21439093'], unisexual reproduction ability (9 results) S ['21439093'], polyploid Carassius auratus (2 results) S ['20596906', '15014154'], nucleo-cytoplasmic (600 results) S ['21439093'], nucleo-cytoplasmic hybrid (28 results) S ['21439093'], clonal diversity (462 results) S ['21439093'], gibel (169 results) S ['21439093', '20596906'], dual reproduction modes (1 results) S ['20596906'], dual reproduction (2315 results) S ['21439093'], gonochoristic sexual (34 results) S ['21439093'], gynogenetic (274 results) S ['21439093'], dual modes of unisexual (2 results) S ['21439093', '20596906'], sexual reproduction (1620 results) S ['21439093'], unisexual gynogenesis (14 results) D ['18508756', '17892466', '17546077'], unisexual gynogenesis (14 results) S ['21439093', '20596906'], nucleo-cytoplasmic hybrid female (5 results) S ['21439093']
JE	TRUE	3	TRUE	inhibitor-resistant TEM-type mutations (5 results) D ['8592985'], CCP4 programs for protein (7 results) D ['15299374'], solvent-water applications (17 results) D ['9613235'], fluorescent biosensor for beta-lactam (5 results) S ['18429614', '15053574'], PenP-E166Cf (1 results) S ['21443768'], beta-lactamase mutant for high-throughput (9 results) S ['16097768'], complex with cefotaxime (143 results) S ['21443768'], AmpC implications (24 results) D ['11478888'], PenP (35 results) S ['21443768', '16097768'], beta-lactam antibiotics (5121 results) S ['21443768'], extended-spectrum class (25 results) D ['12221102', '11371184', '10441119'], Fluorescein-labeled (2109 results) S ['21443768'], biosensor for beta-lactam (58 results) S ['21443768', '18429614'], Acyl-intermediate structures (1 results) D ['12221102'], AmpC implications for resistance (16 results) D ['11478888'], novel fluorescent biosensor (8 results) S ['15053574']
JE	TRUE	4	TRUE	non-conventional hemolysin (3 results) D ['20854656'], tuberculosis TlyA (22 results) D ['20854656', '17496129', '16857584', '15673735'], tuberculosis TlyA (22 results) S ['21443791'], Rv1694 of Mycobacterium (2 results) D ['20854656', '15673735'], Rv1694 (2 results) D ['20854656', '15673735'], ribosomal RNA methyl transferase (130 results) D ['20854656'], Serpulina hyodysenteriae (141 results) D ['11159958'], ribosomal RNA methyl (885 results) D ['20854656'], factor of Serpulina (15 results) D ['9611795'], tlyA gene (16 results) D ['20854656', '16857584', '15673735', '9611795'], tlyA gene (16 results) S ['21443791'], characterization of tlyA (6 results) D ['20854656', '9611795'], characterization of tlyA (6 results) S ['21443791'], TlyA protein (11 results) D ['20854656', '16857584', '15673735', '9611795'], TlyA protein (11 results) S ['21443791'], tlyA gene product (1 results) D ['20854656'], TlyA's function (1 results) S ['21443791'], Mycobacterium tlyA (20 results) D ['20854656', '17496129', '16857584', '15673735', '9611795'], Mycobacterium tlyA (20 results) S ['21443791'], Molecular characterization of tlyA (5 results) D ['20854656', '9611795'], Molecular characterization of tlyA (5 results) S ['21443791'], TlyA (36 results) D ['20854656', '17496129', '16857584'], TlyA (36 results) S ['21443791'], hemolysin (10410 results) S ['21443791'], virulence factor of Serpulina (9 results) D ['9611795'], TlyA's (1 results) S ['21443791'], haemolysin from Mycobacterium (42 results) D ['20854656', '15673735'], haemolysin from Mycobacterium (42 results) S ['21443791']

PMC3072154	6	FALSE	17	FALSE		5	23	TRUE	22	TRUE	20	TRUE	18	TRU
PMC3076115	0	TRUE	18	FALSE		2	17	TRUE	16	TRUE	15	TRUE	15	TRU
PMC3077041	5	FALSE	12	FALSE		6	16	TRUE	10	TRUE	6	TRUE	4	TRU
PMC3077038	2	FALSE	13	FALSE		2	14	TRUE	11	TRUE	11	TRUE	10	TRU
PMC3069548	15	FALSE	2	FALSE		2	13	TRUE	12	TRUE	12	TRUE	12	TRU

JE	TRUE	15	TRUE	antimuscarinic MTC (3 results) S ['21487451'], primary MTC analysis (201 results) S ['21487451'], exacerbations (10639 results) S ['21487451'], LABA (485 results) S ['21487451'], MTC (3037 results) S ['21487451'], long-acting (15149 results) S ['21487451'], beta LAMA (70 results) S ['21487451'], obstructive pulmonary ICS (218 results) D ['21272339'], obstructive pulmonary ICS (218 results) S ['21487451'], pulmonary ICS (691 results) D ['21272339'], pulmonary ICS (691 results) S ['21487451'], roflumilast (181 results) D ['21272339'], roflumilast (181 results) S ['21487451'], comparison meta-analysis (31 results) S ['21487451'], combination of roflumilast (20 results) D ['19716961', '19716960'], combination of roflumilast (20 results) S ['21487451'], mixed-treatment comparison (68 results) S ['21487451'], mixed-treatment (138 results) S ['21487451'], multiple treatment comparison meta-analysis (164 results) S ['21487451'], MTC analysis (7 results) D ['19637942'], MTC analysis (7 results) S ['21487451'], chronic obstructive pulmonary ICS (212 results) D ['21272339'], chronic obstructive pulmonary ICS (212 results) S ['21487451']
JE	TRUE	15	TRUE	blunt trauma (5693 results) S ['21499565'], unusual case of RRD (3 results) S ['21499565'], pars plana (3953 results) S ['21499565'], RRD (400 results) S ['21499565'], macula (11109 results) S ['21499565'], vitrectomy (11414 results) S ['21499565'], pars plana vitrectomy (2972 results) S ['21499565'], retinal detachment (18477 results) S ['21499565'], retinal tear (352 results) S ['21499565'], cognitive disorder Takayuki Baba (2 results) S ['21499565', '17552384'], disorder Takayuki Baba (8 results) S ['21499565'], rhegmatogenous retinal (1467 results) S ['21499565'], plana vitrectomy (2979 results) S ['21499565'], giant retinal (278 results) S ['21499565'], giant retinal tear (112 results) S ['21499565'], rhegmatogenous retinal detachment (1295 results) S ['21499565'], silicone oil (2197 results) S ['21499565'], vitreous (25536 results) S ['21499565']
JE	TRUE	2	TRUE	Kinouchi Abe (20 results) S ['16769690', '12671005', '11591475'], Biases of Influenza (132 results) D ['20723216'], genomic sequence fragments (6 results) S ['16769690'], O157 genome (8 results) S ['11591475'], usage diversity (5 results) S ['11591475'], microbe mixtures (25 results) S ['16769690'], BLSOM learning (1 results) S ['21444341'], Usage Biases of Influenza (3 results) D ['20723216'], Kanaya Kinouchi (10 results) S ['16769690', '12671005', '11591475'], coli O157 genome (2 results) S ['11591475'], BLSOM (3 results) S ['21444341'], codon usage diversity (5 results) S ['11591475'], Usage Biases (45 results) D ['20723216'], Kanaya Kinouchi Abe (7 results) S ['16769690', '12671005', '11591475'], Kimihito Ito Research (11 results) D ['20049332'], Kimihito Ito Research Center (7 results) D ['20049332'], DNA Res (669 results) S ['21444341']
JE	TRUE	3	TRUE	Senecioneae (19 results) S ['21444340'], Asteraceae family (65 results) S ['21444340'], Senecioneae clade (3 results) S ['21444340'], intraspecific polymorphic markers (3 results) S ['21444340'], chloroplast genome (896 results) S ['21444340'], complete chloroplast (61 results) D ['19414502'], complete chloroplast (61 results) S ['21444340'], intraspecific polymorphic (423 results) S ['21444340'], chloroplast genomes of individuals (72 results) S ['21444340'], species Jacobaea (29 results) S ['21444340'], invasive individuals (4 results) S ['21444340'], Pest Species Jacobaea (1 results) S ['21444340'], complete chloroplast genome (34 results) D ['19414502'], complete chloroplast genome (34 results) S ['21444340'], Asteraceae (3158 results) S ['21444340']
JE	TRUE	8	TRUE	Brain Potentials Illuminate (26 results) D ['21441983'], Versus Unsuccessful (728 results) D ['21441983'], Illuminate Successful Versus (4 results) D ['21441983'], Potentials Illuminate Successful Versus (1 results) D ['21441983'], Successful Versus Unsuccessful (285 results) D ['21441983'], Illuminate Successful Versus Unsuccessful (1 results) D ['21441983'], Other-Race (426 results) D ['21441983'], Other-Race (426 results) S ['21483762'], Brain Potentials Illuminate Successful (1 results) D ['21441983'], Potentials Illuminate Successful (1 results) D ['21441983'], Other-Race Faces (63 results) D ['21441983'], Same-Race (235 results) D ['21441983'], Illuminate Successful (82 results) D ['21441983'], Brain Potentials (2109 results) D ['21441983'], Front Hum Neurosci (276 results) D ['21441983'], Front Hum Neurosci (276 results) S ['21483762'], Potentials Illuminate (46 results) D ['21441983']

PMC3069547	3	FALSE	9	FALSE		4	11	TRUE	10	TRUE	8	TRUE	8	TRU
PMC3078899	0	TRUE	12	FALSE		1	12	TRUE	11	TRUE	11	TRUE	10	TRU
PMC3072323	0	TRUE	4	FALSE		1	4	FALSE	4	FALSE	4	FALSE	4	TRU
PMC3078832	0	TRUE	12	FALSE		2	11	TRUE	10	TRUE	8	TRUE	8	TRU
PMC3065660	1	FALSE	10	FALSE		6	8	TRUE	2	TRUE	2	TRUE	1	TRU
PMC3065386	17	FALSE	11	FALSE		17	27	TRUE	22	TRUE	20	TRUE	19	TRU

JE	TRUE	6	TRUE	functional transcranial (111 results) S ['21483761'], language lateralization (325 results) S ['21483761'], fTCD task (32 results) S ['21483761'], transcranial Doppler (5419 results) S ['21483761'], fTCD measurements (9 results) D ['9445333'], functional transcranial Doppler (108 results) S ['21483761'], bone window thickness (1 results) D ['2237951'], lateralization (5260 results) S ['21483761'], fTCD experiment (2 results) S ['21483761'], fTCD (82 results) S ['21483761'], fMRI LIs (103 results) S ['21483761'], interference measures of lateralization (10 results) D ['14591263']
JE	TRUE	8	TRUE	sustainable capacity (17 results) S ['21443780'], stakeholders including policy (443 results) S ['21443780'], sustainability of capacity (481 results) S ['21443780'], southern partners (808 results) S ['21443780'], sustainable capacity building (2 results) S ['21443780'], research capacity (440 results) S ['21443780'], capacity building (1236 results) S ['21443780'], collaboration with stakeholders (792 results) S ['21443780'], capacity building need (523 results) S ['21443780'], early stages of capacity (1835 results) S ['21443780'], building activities (99 results) S ['21443780'], capacity building activities (25 results) S ['21443780']
JE	TRUE	3	TRUE	Siddiqi (1055 results) S ['21443766'], macroeconomic (4425 results) S ['21443766'], political stability (95 results) S ['21443766'], broader health (154 results) S ['21443766']
JE	TRUE	4	TRUE	avian influenza case occurrences (2 results) S ['21443769', '19619336'], ecological niche models (49 results) S ['21443769', '19619336'], influenza case occurrence (1543 results) S ['21443769'], model predictions (3181 results) S ['21443769'], ecological niche (801 results) S ['21443769'], northeastern Africa (9 results) S ['19619336'], suitable for H5N1 (59 results) S ['19619336'], vegetation indices (65 results) S ['21443769'], best index product (181 results) S ['21443769'], case occurrences (7 results) S ['21443769', '19619336'], niche models (82 results) S ['21443769'], within-region predictions (2 results) S ['21443769']
JE	TRUE	0	FALSE	ARMYDA-CAMs (2 results) S ['17045888'], MYocardial Damage during Angioplasty-Cell (2 results) S ['17045888'], Angioplasty-Cell Adhesion substudy (2 results) S ['17045888'], ARMYDA-RECAPTURE (2 results) S ['21461336', '19643320'], ARMYDA for Reduction (26 results) S ['21461336', '20520540', '19643320', '17394957', '17045888'], ARMYDA-RECAPTURE for Reduction (2 results) S ['21461336', '19643320'], chronic atorvastatin treatment (5 results) D ['15837263'], Angioplasty-Cell Adhesion (2 results) S ['17045888'], Adhesion substudy (18 results) S ['17045888'], Damage during Angioplasty-Cell (2 results) S ['17045888'], ARMYDA-CAMs for Reduction (1 results) S ['17045888']
JE	TRUE	6	TRUE	LIM1 domain (16 results) D ['19074270', '11078733', '10574708'], ILK recruitment (25 results) D ['19435803', '12781130'], ILK recruitment (25 results) S ['21444757'], PINCH LIM1 (5 results) D ['11078733'], sites of integrin (5398 results) S ['21444757'], ILK recruits (4 results) D ['12670870'], ANKRs of ILK (643 results) S ['21444757'], Velyvis Yang (5 results) D ['12432066', '11078733'], Drosophila MASs (2809 results) S ['21444757'], integrin-linked (669 results) S ['21444757'], Voronov Chang (2 results) D ['12422219'], binds ILK (30 results) D ['19829382', '15976450'], Braun Pfeifer (8 results) D ['12670870'], paxillin (1891 results) S ['21444757'], integrin adhesion (429 results) S ['21444757'], ILK kinase domain (83 results) D ['20005845', '19829382', '19435803', '19074270'], ILK kinase domain (83 results) S ['21444757'], Obberghen-Schilling (61 results) D ['19435803', '16546570'], Rahmani Moerman (1 results) D ['17662976'], recruitment of PINCH (30 results) D ['19435803', '12432066'], recruitment of PINCH (30 results) S ['21444757'], recruitment of ILK (25 results) D ['19435803', '12781130'], recruitment of ILK (25 results) S ['21444757'], Inoue Stewart (7 results) D ['2192598'], PINCH localization (51 results) D ['17662976'], PINCH localization (51 results) S ['21444757'], Ras suppressor (26 results) D ['15878342', '15596544'], PINCH LIM1 domain (3 results) D ['11078733']

PMC3069583	2	FALSE	19	FALSE		3	18	TRUE	17	TRUE	17	TRUE	12	TRU
PMC3069582	14	FALSE	10	FALSE		9	24	TRUE	22	TRUE	19	TRUE	18	TRU
PMC3069578	2	FALSE	17	FALSE		6	18	FALSE	18	FALSE	18	TRUE	17	TRU
PMC3069577	0	TRUE	0	TRUE		0	0	FALSE	0	FALSE	0	FALSE	0	FAL
PMC3069571	4	FALSE	6	FALSE		6	8	TRUE	7	TRUE	7	TRUE	7	TRU

JE	TRUE	6	TRUE	heterogeneous diverse findings (4598 results) S ['21468271'], inflammatory pathophysiology with dystrophinopathy (4 results) S ['21468271'], ligation probe (63 results) S ['21468271'], liver aminotransferase (30 results) S ['21468271'], dystrophin (5459 results) S ['21468271'], multiple ligation probe amplification (18 results) D ['18639760', '15841391'], multiple ligation probe amplification (18 results) S ['21468271'], multiple ligation probe (20 results) D ['18639760', '15841391'], multiple ligation probe (20 results) S ['21468271'], pathophysiology with dystrophinopathy (38 results) S ['21468271'], suspicious perifascicular atrophy (1 results) S ['21468271'], inflammatory myositis (127 results) S ['21468271'], subacute proximal muscle (71 results) S ['21468271'], carrier woman (17 results) S ['21468271'], DMD gene (429 results) S ['21468271'], infiltration of mono-macrophage (1 results) S ['21468271'], DMD (3649 results) S ['21468271'], carrier of DMD (355 results) S ['21468271'], ligation probe amplification (52 results) S ['21468271'], subacute proximal muscle weakness (40 results) S ['21468271'], suspicious perifascicular (1 results) S ['21468271']
JE	TRUE	10	TRUE	lipid-laden (779 results) D ['21165301'], lipid-laden (779 results) S ['21468270'], Xanthogranulomatous inflammation (67 results) D ['20382597', '19881238'], Xanthogranulomatous inflammation (67 results) S ['21468270'], xanthogranulomatous changes (12 results) D ['18392724', '18019685'], urachus (1188 results) S ['21468270'], cases of XGP (52 results) D ['21165301', '18971878'], XGP mimic (2 results) D ['21165301'], histiocytes (7892 results) S ['21468270'], XGP (102 results) D ['21165301', '18971878'], XGP (102 results) S ['21468270'], Xanthogranulomatous pancreatitis (6 results) D ['21165301', '20382597', '18392724', '16120922', '15471123'], Xanthogranulomatous pancreatitis (6 results) S ['21468270'], XGI (7 results) D ['19881238'], XGI (7 results) S ['21468270'], Xanthogranulomatous (1173 results) D ['21165301'], Xanthogranulomatous (1173 results) S ['21468270'], histiocytes aggregation (24 results) D ['18392724'], Xanthogranulomatous mass (199 results) D ['21165301'], Xanthogranulomatous mass (199 results) S ['21468270'], most cases of XGP (52 results) D ['21165301', '18971878'], Xanthogranulomatous mass lesion (51 results) D ['21165301'], Xanthogranulomatous mass lesion (51 results) S ['21468270'], pathogenesis of XGP (41 results) D ['21165301']
JE	TRUE	12	TRUE	BRC for CRPS (1 results) S ['21468266'], CRPS (956 results) S ['21468266'], characteristics of CRPS (46 results) D ['19590474'], characteristics of CRPS (46 results) S ['18955780'], risk ratios (1782 results) S ['21468266'], research population of CRPS (23 results) D ['17084977', '12749974', '12725849'], research population of CRPS (23 results) S ['21468266'], regional pain (1737 results) S ['21468266'], feature of CRPS (13 results) S ['21468266'], complex regional pain (1422 results) S ['21468266'], regional pain syndrome population (123 results) S ['21468266'], regional pain syndrome (1289 results) S ['21468266'], pain syndrome (5595 results) S ['21468266'], BRC (667 results) S ['21468266'], CRPS populations (48 results) S ['21468266'], motor dysfunction (2022 results) S ['21468266'], complex regional pain syndrome (1222 results) S ['21468266'], pain syndrome population (2773 results) S ['21468266'], objective signs (694 results) S ['21468266']
.SE	FALSE	0	FALSE	searches
JE	TRUE	3	TRUE	large single coronary (4098 results) S ['21468259'], stents versus (3288 results) S ['21468259'], Korea Acute Myocardial Infarction (34 results) S ['21468259', '20554156', '19949634'], single culprit vessels (53 results) D ['17643591'], BASKET Long-term benefit-risk (1 results) D ['19033260'], BASKET Long-term benefit-risk balance (1 results) D ['19033260'], systematic glycoprotein inhibitor infusion (5 results) D ['17616297'], bare metal stents (1356 results) S ['21468259'], Infarction Doo Sun Sim (26 results) S ['21468259', '20554156', '19949634'], Korea Acute Myocardial (34 results) S ['21468259', '20554156', '19949634']

PMC3069570	0	TRUE	13	FALSE		2	13	FALSE	13	TRUE	11	TRUE	11	TRU
PMC3069569	5	FALSE	8	FALSE		3	13	TRUE	12	TRUE	10	TRUE	8	TRU
PMC3069568	0	TRUE	10	FALSE		1	9	TRUE	8	TRUE	6	TRUE	2	TRU
PMC3069567	7	FALSE	24	FALSE		5	31	FALSE	31	TRUE	30	TRUE	23	TRU

JE	TRUE	8	TRUE	patients with ESCC (655 results) S ['21468258'], response after CCRT (69 results) S ['21468258'], esophageal squamous cell (2593 results) S ['21468258'], higher expression of VEGF (3189 results) S ['21468258'], ESCC (1094 results) S ['21468258'], COX-2 expression (4151 results) S ['21468258'], rates after CCRT (45 results) S ['21468258'], ESCC after definitive (10 results) S ['21468258'], esophageal squamous cell carcinoma (2222 results) S ['21468258'], complete response after concurrent (880 results) S ['21468258'], CCRT (362 results) S ['21468258'], definitive CCRT (8 results) S ['21468258', '18053639'], Unlike other well-known studies (57 results) S ['21468258']
JE	TRUE	4	TRUE	tertiary component (9 results) D ['15104304'], Cancer Hospital Database (29 results) D ['18778348'], Jin Seon Cho (19 results) S ['21468257'], Changhee Yoo (4 results) S ['21468257'], hazards for biochemical (858 results) S ['21468257'], high grade tertiary component (13 results) D ['15104304'], Eun Sik Lee (8 results) S ['21468257'], Regional Cancer Hospital Database (28 results) D ['18778348'], biopsy cores (539 results) S ['21468257'], grade tertiary component (23 results) D ['15104304'], bGS (364 results) S ['21468257'], pGS (5630 results) S ['21468257'], Han Yong Choi (40 results) S ['21468257']
JE	TRUE	1	TRUE	bilateral lung infiltration (11 results) S ['21468256'], Yee Gyung Kwak (18 results) S ['21468256'], Severe Cases among Adult (53901 results) S ['21468256'], Kyoung-Ho Song (17 results) S ['21468256'], In-Gyu Bae (13 results) S ['21468256'], Sang-Taek Heo (22 results) S ['21468256'], Ae-Chung Hur (1 results) S ['21468256'], Yoon Seon Park (6 results) S ['21468256'], Kkot Sil Lee (7 results) S ['21468256'], Ji-An Hur (3 results) S ['21468256']
JE	TRUE	17	TRUE	guinea pig cochlea (971 results) S ['21468255'], mild hearing recovery (89 results) S ['21468255'], hearing recovery (198 results) S ['21468255'], spiral ganglion (2055 results) S ['21468255'], scala tympani (1028 results) S ['21468255'], auditory-neuropathy animal effects (17 results) D ['18449604', '17632425', '12382099'], auditory-neuropathy animal effects (17 results) S ['21468255'], ouabain (20886 results) S ['21468255'], guinea pig cochlea days (619 results) S ['21468255'], round window (1944 results) S ['21468255'], cochlea days after ouabain (11 results) D ['18449604', '17632425', '12382099'], cochlea days after ouabain (11 results) S ['21468255'], tympani (3109 results) S ['21468255'], application of ouabain (614 results) D ['18449604'], application of ouabain (614 results) S ['21468255'], ouabain injury (289 results) S ['21468255'], auditory neuropathy (334 results) S ['21468255'], pig cochlea days (286 results) S ['21468255'], hearing recovery after transplantation (20 results) D ['17632425', '17591967'], hearing recovery after transplantation (20 results) S ['21468255'], SGNs (105 results) S ['21468255'], nuclear antibody under confocal (94 results) S ['21468255'], cochlea days (1980 results) S ['21468255'], SNHL model (29 results) D ['17632425'], number of SGNs (14 results) D ['17632425'], number of SGNs (14 results) S ['21468255'], days after ouabain (402 results) S ['21468255'], hair cells (9707 results) S ['21468255'], loss of SGNs (54 results) S ['21468255'], auditory-neuropathy animal (37 results) D ['18449604', '17632425'], auditory-neuropathy animal (37 results) S ['21468255']

PMC3069566	0	TRUE	25	FALSE		1	22	TRUE	18	TRUE	16	TRUE	15	TRU
PMC3069564	5	FALSE	14	FALSE		4	19	TRUE	17	TRUE	14	TRUE	13	TRU
PMC3076233	3	FALSE	13	FALSE		3	16	FALSE	16	TRUE	15	TRUE	12	TRU
PMC3073883	5	FALSE	9	FALSE		7	9	TRUE	7	TRUE	3	TRUE	3	TRU

JE	TRUE	13	TRUE	hATMSCs with scaffolds (2 results) S ['21468254'], nude rat bone defect (59 results) S ['21468254'], femoral segmental defect (14 results) S ['21468254'], cell media phosphate (4717 results) S ['21468254'], hATMSC-related (1 results) S ['21468254'], GLP-compliant toxicity study (6 results) S ['21468254'], rat bone defect (5 results) S ['21468254'], defect model (562 results) S ['21468254'], GLP-compliant toxicity (8 results) S ['21468254'], cell-loading (214 results) S ['21468254'], density-dependent (2999 results) S ['21468254'], nude rat bone (537 results) S ['21468254'], hybrid assessment system (327 results) S ['21468254'], bone defect (2281 results) S ['21468254'], new bone formation (3631 results) S ['21468254'], preclinical assessment system (622 results) S ['21468254'], cell therapies (966 results) S ['21468254'], bone defect model (87 results) S ['21468254'], phosphate scaffolds Group (196 results) S ['21468254'], hATMSC-loaded (1 results) S ['21468254'], hATMSCs-loaded (1 results) S ['21468254'], hATMSCs (2 results) S ['21468254'], segmental defects (390 results) S ['21468254'], rat bone defect model (4 results) S ['21468254'], density-dependent manner (196 results) S ['21468254']
JE	TRUE	7	TRUE	ELBWI (21 results) S ['21468252'], rates of VLBWI (10 results) S ['21468252', '14966337'], perinatal care centers (12 results) S ['21468252'], nationwide neonatal (1350 results) S ['21468252'], mortality of LBWI (7 results) S ['14966337'], Perinatal Care Center Network (58 results) D ['20732945'], rates of LBWI (2 results) S ['14966337'], VLBWI (57 results) S ['21468252'], birth of ELBWI (21 results) S ['21468252'], nationwide Neonatal Research (760 results) S ['21468252'], Neonatal Research Network (156 results) D ['20732945'], NICHD Neonatal Research (58 results) D ['20732945'], organization of perinatal (5988 results) S ['21468252'], NICHD Neonatal Research Network (56 results) D ['20732945'], perinatal care (3240 results) S ['21468252'], regionalization of perinatal (170 results) D ['20810377'], regionalization of perinatal (170 results) S ['21468252'], survival rates of VLBWI (4 results) S ['21468252'], mortality rates of VLBWI (8 results) S ['21468252', '14966337']
JE	TRUE	8	TRUE	properties of PABA (649 results) S ['21443763'], PABA (955 results) S ['21443763'], PABA conjugates (26 results) S ['21443763'], acid side chain (953 results) S ['21443763'], different acid side (8490 results) S ['21443763'], acid side (2797 results) S ['21443763'], larval dietary uptake (37 results) D ['19746737'], uptake mechanism (1348 results) S ['21443763'], C-16 side (122 results) S ['21443763'], sub-cellular internalization (7 results) S ['21443763'], PABA nanomaterials (19 results) S ['21443763', '20332564'], energy dependent endocytosis (17 results) S ['21443763'], PABA nanoparticles (20 results) S ['21443763'], C-16 side chain (64 results) S ['21443763'], adult exposure causes locomotor (137 results) D ['19746737'], exposure causes locomotor impairment (42 results) D ['19746737']
JE	TRUE	1	TRUE	anti-neoplastic effects of tetrapropionate (1 results) S ['16822594'], maghemite (284 results) S ['21443799'], F68 nanoparticles after intratumoral (1 results) D ['20016371'], adducts with nicotinate (8 results) S ['16822594'], silicon tribenzonaphthoporphyrazinato (1 results) S ['18681070'], F68 nanoparticles (3 results) D ['20016371'], effects of tetrapropionate (1 results) S ['16822594'], maghemite nanoparticles (70 results) S ['21443799'], tribenzonaphthoporphyrazinato (1 results) S ['18681070'], release of rhodium (22 results) D ['12033599', '10229652'], cyclodextrins from hydroxyapatite (8 results) D ['12033599'], free rhodium citrate (3 results) S ['21443799'], rhodium citrate (9 results) D ['12033599', '10229652', '2804473'], rhodium citrate (9 results) S ['21443799']

PMC3078901	8	FALSE	11	FALSE		6	16	TRUE	14	TRUE	12	TRUE	8	TRU
PMC3077327	0	TRUE	12	FALSE		2	10	TRUE	9	TRUE	8	TRUE	8	TRU
PMC3078873	6	FALSE	11	FALSE		25	17	TRUE	16	TRUE	16	TRUE	16	TRU
PMC3074560	8	FALSE	6	FALSE		4	13	TRUE	8	TRUE	7	TRUE	7	TRU
PMC3065494	9	FALSE	6	FALSE		7	10	TRUE	8	TRUE	6	TRUE	4	TRU

JE	TRUE	4	TRUE	Plasmodium berghei (4400 results) S ['21439091'], NOD STAT5B mutation (3 results) D ['17130555'], yoelii 17XL infection up-regulates (1 results) S ['16359553'], adami infection (13 results) D ['17868677', '1452338'], Plasmodium chabaudi adami infection (7 results) D ['17868677', '1452338'], 17XL infection up-regulates RANTES (1 results) S ['16359553'], NOD STAT5B (5 results) D ['17130555'], cerebral malaria (2243 results) S ['21439091'], Plasmodium yoelii 17XL (35 results) S ['16359553'], berghei ANKA (319 results) S ['21439091'], chabaudi adami infection (12 results) D ['17868677', '1452338'], berghei (4606 results) S ['21439091'], infection up-regulates RANTES (11 results) S ['16359553'], Plasmodium chabaudi adami (56 results) D ['17868677'], interleukin-2 treatment induces accumulation (20 results) D ['11141489'], induces accumulation of gammadelta (10 results) D ['11141489'], CXCL-10 (71 results) S ['21439091'], 17XL infection up-regulates (1 results) S ['16359553'], up-regulates RANTES (29 results) S ['16359553']
JE	TRUE	5	TRUE	si-RNA (340 results) S ['21443795'], IGF-2 polypeptides (8806 results) S ['21443795'], si-InR treatment (1 results) S ['21443795'], FASD (290 results) S ['21443795'], si-RNA treatments (35 results) S ['21443795'], hypofoliation (2 results) S ['21443795'], si-InR (1 results) S ['21443795'], alcohol spectrum potential (1130 results) S ['21443795'], alcohol spectrum potential link (10 results) S ['19393862'], IGF-2R (49 results) S ['21443795'], fetal alcohol spectrum potential (181 results) S ['21443795'], central nervous system neuronal (41 results) S ['19393862']
JE	TRUE	6	TRUE	low-level nociceptive (39 results) S ['16778058'], superficial dorsal horn neurons (73 results) D ['20089138'], secondary hyperalgesia (301 results) D ['20336422', '20336420'], factor-alpha induces long-term potentiation (3 results) S ['20116424', '17084420'], LTP induction (912 results) S ['21443797'], spinal LTP (32 results) D ['20164395', '20089138', '16930406', '16815482', '16427664'], spinal LTP (32 results) S ['21443797', '20116424', '18837052', '17084420', '16950233', '16902997', '16324725'], necrosis factor-alpha induces long-term (132 results) S ['20116424'], initial painful event (509 results) S ['21443797'], secondary pinprick (37 results) D ['16781073'], secondary pinprick (37 results) S ['17084865'], factor-alpha induces long-term (143 results) S ['20116424'], secondary pinprick hyperalgesia (25 results) D ['16781073', '15109518', '11522578', '9422898'], secondary pinprick hyperalgesia (25 results) S ['17084865'], primary afferent C-fibres (28 results) S ['18507818', '9749775', '9697127'], prevents LTP induction (75 results) D ['18448254'], afferent C-fibres (110 results) S ['18507818', '14659520']
JE	TRUE	3	TRUE	high fat diabetic (1695 results) S ['21439094'], high fat diabetic rats (483 results) S ['21439094'], fat diabetic rats (945 results) S ['21439094'], outer dry layers (88 results) D ['17997520'], Dietary quercetin alleviates diabetic (2 results) D ['19496084'], onion peel (24 results) D ['17484375'], onion peel (24 results) S ['21439094'], quercetin alleviates diabetic (2 results) D ['19496084'], potency of OPE (2 results) S ['21439094'], Dietary quercetin alleviates (4 results) D ['19496084'], dry layers of onion (2 results) D ['17997520'], pure quercetin equivalent (7 results) S ['21439094'], quercetin alleviates diabetic symptoms (1 results) D ['19496084'], alleviates diabetic symptoms (52 results) D ['19496084']
JE	TRUE	1	TRUE	adenovirus after intracranial (45 results) S ['19011597'], Ad-RGD (22 results) D ['16626731', '16574766'], Ad-RGD (22 results) S ['21464908'], motifs improves coxsackievirus-adenovirus receptor-independent (1 results) D ['12228019'], improves coxsackievirus-adenovirus receptor-independent (1 results) D ['12228019'], coxsackievirus-adenovirus receptor-independent gene (3 results) D ['12228019'], Oncolytic Adenovirus Suppress (15 results) S ['20588259'], coxsackievirus-adenovirus receptor-independent gene transfer (2 results) D ['12228019'], Ad-RGD viruses (18 results) D ['16626731', '16574766'], oncolytic (2379 results) S ['21464908'], oncolytic adenovirus after intracranial (6 results) S ['19011597'], polylysine motifs improves coxsackievirus-adenovirus (1 results) D ['12228019'], oncolytic Ad-RGD (1 results) S ['21464908'], receptor-independent gene transfer efficiency (11 results) D ['12228019', '9811704'], improves coxsackievirus-adenovirus receptor-independent gene (1 results) D ['12228019']

PMC3065493	6	FALSE	2	FALSE		4	4	FALSE	4	FALSE	4	TRUE	3	TRU
PMC3065491	9	FALSE	5	FALSE		4	12	TRUE	9	TRUE	5	TRUE	5	TRU
PMC3065489	13	FALSE	12	FALSE		16	23	TRUE	20	TRUE	20	TRUE	18	TRU
PMC3065487	1	FALSE	17	FALSE		7	18	TRUE	14	TRUE	13	TRUE	12	TRU
PMC3065484	2	FALSE	4	FALSE		3	6	TRUE	5	TRUE	5	TRUE	5	TRU
PMC3065482	0	TRUE	10	FALSE		2	10	FALSE	10	TRUE	8	TRUE	8	TRU

JE	TRUE	3	TRUE	system elucidates mouse home (1 results) D ['19106295'], elucidates mouse home cage (1 results) D ['19106295'], home cage behavioral structure (13 results) D ['19106295'], mouse home cage behavioral (400 results) S ['21464907'], preproghrelin-deficient (1 results) D ['19939974'], preproghrelin-deficient mice (1 results) D ['19939974'], food anticipatory (116 results) D ['20668550'], food anticipatory (116 results) S ['21464907']
JE	TRUE	4	TRUE	mutation induce rapid neuritic (1 results) D ['16242634'], Small non-fibrillar (38 results) D ['16242634'], Small non-fibrillar assemblies (2 results) D ['16242634'], non-fibrillar assemblies (3 results) D ['16242634', '11750898'], amyloid beta-peptide (2866 results) S ['21464905'], oligomeric amyloid beta-peptide (10 results) S ['19435461'], Arctic mutation induce (4 results) D ['16242634'], non-fibrillar assemblies of amyloid (9 results) D ['16242634', '11750898'], rapid neuritic degeneration (7 results) D ['16242634'], beta-peptide (3518 results) S ['21464905'], species of amyloid-beta (1746 results) S ['21464905'], induce rapid neuritic degeneration (1 results) D ['16242634'], induce rapid neuritic (8 results) D ['16242634'], fibrillar species of amyloid-beta (167 results) S ['21464905']
JE	TRUE	5	TRUE	cone dystrophy (309 results) S ['21464903'], autosomal dominant cone dystrophy (17 results) D ['18706439', '15735604', '9702199', '9425234'], autosomal dominant cone dystrophy (17 results) S ['11484154'], dominant cone (90 results) D ['18706439'], dominant cone (90 results) S ['21464903', '19299492'], Elovl4 knock-in mice (4 results) D ['17003453'], GCAP1 (91 results) D ['19459154', '18706439'], GCAP1 (91 results) S ['21464903', '19941038'], autosomal dominant cone (66 results) D ['18706439', '15790869'], protein rescues cone recovery (1 results) D ['12732716'], Elovl4 knock-in (4 results) D ['17003453'], cyclase-activating (3138 results) S ['21464903'], Guca1a locus (5 results) D ['15735604', '9425234'], rescues cone recovery kinetics (1 results) D ['12732716'], guanylate (10247 results) S ['21464903'], flash sensitivity of rod (196 results) S ['19941038'], protein causes cone (4004 results) S ['21464903'], GUCA1A (69 results) D ['19459154', '18706439', '15790869', '15735604'], GUCA1A (69 results) S ['21464903', '19941038', '16644365', '15953638'], dominant cone dystrophy (24 results) D ['18706439', '15735604', '9702199', '9425234'], dominant cone dystrophy (24 results) S ['11484154'], gene for GCAP1 (42 results) D ['19459154', '18706439', '15790869', '15735604', '15505030'], gene for GCAP1 (42 results) S ['21464903', '19941038', '15953638'], gene GUCA1A (39 results) D ['19459154', '18706439', '15790869', '15735604', '15505030', '15240799'], gene GUCA1A (39 results) S ['21464903', '19941038', '16644365', '15953638']
JE	TRUE	8	TRUE	stop modulate filopodia formation (2 results) S ['19571116'], actin regulators (46 results) D ['19576200'], actin regulators (46 results) S ['21464901'], Drosophila DAAM (5 results) S ['21464901', '20177055', '19052223', '16469972'], filopodia (2585 results) S ['21464901'], profilin (1169 results) S ['21464901'], formin homology domains (20 results) S ['20177055'], nucleators (143 results) S ['21464901'], Drosophila primary neurons (411 results) S ['21464901'], formin (544 results) S ['21464901'], DAAM (30 results) S ['21464901', '20177055', '19052223'], tendon cells represent (108 results) S ['18667532'], Drosophila tendon cells represent (2 results) S ['18667532'], cells represent architectural elements (7 results) S ['18667532'], filopodia formation (251 results) S ['21464901'], tendon cells represent architectural (2 results) S ['18667532'], cells represent architectural (99 results) S ['18667532'], formin homology (151 results) S ['20177055']
JE	TRUE	3	TRUE	medication adherence among inner-city (74 results) D ['19852197'], personality on adherence (1984 results) S ['21464898'], adherence behaviour (51 results) S ['21464898'], medication adherence (4093 results) S ['21464898'], antidepressant medication compliance (3 results) D ['15065744'], significant importance for adherence (402 results) S ['21464898']
JE	TRUE	2	TRUE	new cancer treatment (58 results) S ['18362256'], interactions between RCTs (48 results) S ['21464896'], treatment discovery (8 results) S ['21464896'], cooperative oncology groups (31 results) S ['18362256'], Treatment success (2829 results) S ['21464896'], RCT interactions (56 results) S ['21464896'], highest centrality measures (8 results) S ['21464896'], RCT networks (24 results) S ['21464896'], treatment discovery process (1999 results) S ['21464896'], oncology groups (69 results) S ['18362256']

PMC3065481	2	FALSE	7	FALSE		6	8	TRUE	7	TRUE	5	TRUE	5	TRU
PMC3065480	2	FALSE	14	FALSE		4	16	TRUE	15	TRUE	14	TRUE	14	TRU
PMC3065477	5	FALSE	16	FALSE		7	18	TRUE	17	TRUE	13	TRUE	12	TRU
PMC3065476	9	FALSE	10	FALSE		10	18	TRUE	13	TRUE	11	TRUE	6	TRU
PMC3065475	5	FALSE	6	FALSE		5	11	TRUE	8	TRUE	5	TRUE	5	TRU

JE	TRUE	3	TRUE	CD4 lymphocyte (15025 results) S ['21464895'], odds of FIR (4 results) S ['21464895'], large urban clinic (6 results) S ['19182060'], Nurse versus doctor management (209 results) D ['20557927'], Kambugu (47 results) S ['21464895'], Malawi through task (23 results) D ['20958897', '20224782', '18992905'], Task-shifting (111 results) S ['21464895'], pharmacy visits for SOC (1 results) S ['21464895'], Babigumira (11 results) S ['21464895', '19182060']
JE	TRUE	10	TRUE	PC12 dissection (23 results) D ['8649837'], gammaretroviral vectors (36 results) S ['21464894'], leukemia virus (18931 results) S ['21464894'], gammaretroviral (94 results) S ['21464894'], murine leukemia (12139 results) S ['21464894'], dihydropyridine-insensitive calcium channels (6 results) S ['2163753'], nerve growth (27068 results) S ['21464894'], gammaretroviruses (87 results) S ['21464894'], murine leukemia virus (12184 results) S ['21464894'], replication-competent gammaretroviruses (263 results) D ['17498744'], replication-competent gammaretroviruses (263 results) S ['21464894'], nerve growth factor (17246 results) S ['21464894'], lentiviral vectors (1631 results) S ['21464894'], Functional expression of dihydropyridine-insensitive (5 results) S ['2163753'], post-mitotic (1454 results) S ['21464894'], PC12 cells (11586 results) S ['21464894']
JE	TRUE	11	TRUE	Bim mediator of cell (115 results) S ['21464892'], Thrombin inhibits Bim mediator (1 results) D ['12844349'], camptothecin (8513 results) S ['21464892'], ability of KLK6 (7 results) S ['21464892', '18778305'], KLK6 promotes (2 results) S ['21464892'], splenocyte (2361 results) S ['21464892'], Blaber (147 results) S ['21464892'], effects of KLK6 (16 results) S ['21464892', '16800746', '16321973'], whole splenocyte preparations (6 results) S ['21464892'], pro-survival (1251 results) S ['21464892'], whole splenocyte (10 results) S ['21464892'], Bim (1829 results) S ['21464892'], Bim mediator (118 results) S ['21464892'], kallikrein (8563 results) S ['21464892'], pepducins inhibits Akt (1 results) D ['19622769'], PAR1 (729 results) S ['21464892'], inhibits Bim mediator (7 results) D ['12844349'], pro-survival effects (61 results) S ['21464892'], KLK6 (119 results) D ['19707197'], KLK6 (119 results) S ['21464892'], Thrombin inhibits Bim (1 results) D ['12844349']
JE	TRUE	3	TRUE	Leptospermum antibacterial (15 results) D ['18210383', '1687577'], Leptospermum antibacterial (15 results) S ['21464891', '19513768'], multiresistant strains of Burkholderia (10 results) D ['10886608'], medical-grade Leptospermum antibacterial (3 results) S ['19513768'], honey against coagulase-negative (2 results) D ['15941774'], antibacterial activity of honey (109 results) S ['21464891'], Leptospermum antibacterial spectrum (3 results) S ['19513768'], sensory properties of citrus (13 results) D ['18303839'], flora for antimicrobial (5767 results) S ['21464891'], floral source (6 results) D ['15527912'], non-peroxide (28 results) D ['15527912', '8961174', '1687577'], non-peroxide (28 results) S ['21464891'], select raw honeys (1 results) D ['15527912'], honey glucose-oxidase system (2 results) D ['14000328'], non-peroxide activity (18 results) D ['15527912', '8961174', '1687577'], non-peroxide activity (18 results) S ['21464891'], medical-grade Leptospermum antibacterial spectrum (2 results) S ['19513768'], Leptospermum (64 results) S ['21464891', '19513768'], activity of honey (613 results) S ['21464891']
JE	TRUE	2	TRUE	adaptive immunity during epileptogenesis (3 results) D ['17931873'], pilocarpine (7238 results) S ['21464890'], study of hydrocortisone (20745 results) S ['21464890'], immunity during epileptogenesis (7 results) D ['17931873'], Acute induction of epileptiform (25 results) S ['18082973'], anti-T cell treatment (7 results) D ['9806067'], versus deflazacort (33 results) D ['18524542'], requires enhancement of blood-brain (5 results) S ['18082973'], hydrocortisone versus deflazacort (2 results) D ['18524542'], guinea-pig brain requires enhancement (8 results) S ['18082973'], guinea-pig brain requires (97 results) S ['18082973']

PMC3065474	9	FALSE	7	FALSE		6	15	TRUE	13	TRUE	12	TRUE	9	TRU
PMC3065473	13	FALSE	5	FALSE		7	11	TRUE	6	TRUE	6	TRUE	5	TRU
PMC3065472	3	FALSE	4	FALSE		4	4	FALSE	4	TRUE	2	TRUE	2	TRU
PMC3065471	2	FALSE	8	FALSE		4	10	FALSE	10	TRUE	9	TRUE	6	TRU
PMC3065469	2	FALSE	8	FALSE		3	10	FALSE	10	FALSE	10	TRUE	9	TRU
PMC3065468	9	FALSE	7	FALSE		7	16	TRUE	13	TRUE	10	TRUE	9	TRU

JE	TRUE	8	TRUE	Subchronic SSRI (21 results) D ['19545475'], tri-allelic 5-HTTLPR genotype (12 results) S ['21464942'], noxious inhibitory control effect (156 results) D ['19359095'], Subchronic SSRI administration reduces (2 results) D ['19545475'], Intramuscular Injection of Granisetron (8 results) D ['17575485'], tri-allelic 5-HTTLPR (12 results) S ['21464942'], reappraisal of 5-HTTLPR (1 results) D ['16402131'], serotonin transporter gene (944 results) S ['21464942'], Diffuse noxious inhibitory controls (157 results) D ['20080425'], noxious inhibitory controls (160 results) D ['20080425'], serotonin transporter (3505 results) S ['21464942'], 5-HTTLPR (922 results) S ['21464942'], CPM-test (1071 results) S ['21464942'], SSRI administration reduces insula (4 results) D ['19545475'], Subchronic SSRI administration (15 results) D ['19545475'], transporter gene (2770 results) S ['21464942']
JE	TRUE	4	TRUE	bladder miR-129 (1 results) D ['19487295'], deep sequencing (543 results) S ['21464941'], suppressor microRNA-101 (4 results) D ['19258506'], utility of LNA (16 results) D ['18295505'], putative tumor suppressor microRNA-101 (1 results) D ['19258506'], simple urothelial hyperplasias (3 results) D ['10079249'], tumor suppressor microRNA-101 (3 results) D ['19258506'], normal urothelium (526 results) S ['21464941'], microRNA-101 modulates (1 results) D ['19258506'], loop between ZEB1-SIP1 (1 results) D ['18829540'], suppressor microRNA-101 modulates (1 results) D ['19258506'], prognostic implications of microRNA (28 results) D ['19676045'], urothelium (4865 results) S ['21464941'], feedback loop between ZEB1-SIP1 (1 results) D ['18829540'], bladder urothelial carcinoma (120 results) S ['21464941'], tumor suppressor microRNA-101 modulates (1 results) D ['19258506'], bladder urothelial carcinoma patients (3 results) S ['21464941'], microRNA-200 family regulates epithelial-mesenchymal (2 results) D ['18829540']
JE	TRUE	0	FALSE	total influenza vaccine failure (28 results) D ['12136133'], helper-dependent adenoviral vaccines (6 results) S ['19333387'], major intrasubtypic antigenic (1 results) D ['12136133'], HA1-con (1 results) S ['21464940'], major intrasubtypic (1 results) D ['12136133'], glycol modification of first-generation (8 results) S ['15585407'], Comparison of replication-competent (91 results) S ['19333387']
JE	TRUE	6	TRUE	extract of huanglian (19 results) D ['15958519'], CMAP (922 results) S ['21464939'], expression profiles of MCF-7 (140 results) S ['21464939'], CMAP gene expression (19 results) D ['20133050'], CMAP gene expression (19 results) S ['21464939'], CMAP profile (10 results) S ['21464939'], SWT (298 results) S ['21464939', '21219992'], ferulic acid (2005 results) S ['21464939'], profiles of MCF-7 (210 results) S ['21464939'], SWT treatment (135 results) S ['21464939']
JE	TRUE	5	TRUE	Rwandan (255 results) S ['21464937'], Baseline renal insufficiency (42 results) D ['18753939'], HIV infection versus diabetes (54 results) D ['17942954'], African HIV populations (3063 results) S ['21464937'], cohort of Rwandan (18 results) S ['21464937'], antiretroviral therapy on kidney (488 results) S ['21464937'], East African HIV populations (131 results) S ['21464937'], few estimates of kidney (70 results) S ['21464937'], Modification of Diet (5896 results) S ['21464937'], Rwandan women (39 results) S ['21464937']
JE	TRUE	4	TRUE	noradrenaline during sympathetic (17586 results) S ['21464936'], presynaptic alpha-2A adrenoceptors (5 results) D ['12817185'], multiple adenosine receptor subtypes (6 results) D ['15452191'], capadenoson (4 results) S ['21464936'], neuronal stunning (2 results) S ['11230970'], hypertensive role of presynaptic (44 results) D ['12817185'], norepinephrine release (1534 results) S ['21464936'], cardiac nerve terminals (10 results) D ['15644943'], Restraint stress differential (255 results) S ['21464936'], partial agonist of adenosine (419 results) S ['21464936'], presynaptic alpha-2A (78 results) D ['12817185'], Adenosine inhibits norepinephrine release (80 results) S ['11230970'], potential partial agonists (7 results) D ['7589213'], cardiac norepinephrine release (39 results) D ['12817185'], GTP shift (22 results) D ['7589213'], Restraint stress differential cardiovascular (13 results) D ['10642286']

PMC3065466	0	TRUE	15	FALSE		1	15	FALSE	15	TRUE	13	TRUE	9	TRU
PMC3065462	10	FALSE	8	FALSE		13	18	TRUE	17	TRUE	17	TRUE	13	TRU
PMC3065461	4	FALSE	6	FALSE		8	10	FALSE	10	TRUE	9	TRUE	8	TRU
PMC3065460	1	FALSE	5	FALSE		2	6	FALSE	6	FALSE	6	TRUE	4	TRU
PMC3065459	12	FALSE	5	FALSE		15	14	FALSE	14	TRUE	11	TRUE	7	TRU
PMC3065458	11	FALSE	3	FALSE		7	14	TRUE	10	TRUE	9	TRUE	6	TRU

JE	TRUE	8	TRUE	household cases (12 results) S ['21464934'], doses of antiviral (2694 results) S ['21464934'], primary household cases (1037 results) S ['21464934'], reproduction number (502 results) S ['21464934'], primary household (17 results) S ['21464934'], household outbreaks (19 results) S ['21464934'], few antiviral doses (86 results) S ['21464934'], initial reproduction number (7 results) S ['21464934'], antiviral doses (7 results) S ['21464934'], antiviral drugs (2613 results) S ['21464934'], influenza pandemic (1643 results) S ['21464934'], containment of transmission (474 results) S ['21464934'], few doses of antivirals (262 results) S ['21464934'], initial reproduction (13 results) S ['21464934'], stockpile (300 results) S ['21464934']
JE	TRUE	2	TRUE	Review Streptococcus suis (49 results) D ['17692141'], Review Streptococcus suis (49 results) S ['19191650'], Streptococcus suis suilysin gene (19 results) D ['19596529', '16431041', '10596804'], Streptococcus suis suilysin gene (19 results) S ['19413493'], Streptococcus suis suilysin (47 results) D ['19596529'], Streptococcus suis suilysin (47 results) S ['19413493'], Streptococcus suis type isolates (73 results) D ['19642376', '17186962', '16965698'], suis type isolates (99 results) D ['19642376'], 136-kilodalton (3 results) D ['1587602'], field strains of Streptococcus (590 results) S ['21464930', '21208459'], Streptococcus suis (851 results) S ['21464930'], Review Streptococcus suis infections (42 results) D ['17692141'], Review Streptococcus suis infections (42 results) S ['19191650'], Streptococcus suis infections (22 results) D ['17692141', '16707046'], suis suilysin (47 results) D ['19596529'], suis suilysin (47 results) S ['19413493'], suis suilysin gene (19 results) D ['19596529', '16431041', '10596804'], suis suilysin gene (19 results) S ['19413493']
JE	TRUE	6	TRUE	NS1 protein (720 results) S ['21464929'], virus NS1 protein (125 results) D ['20702615'], virus NS1 protein (125 results) S ['21464929', '20444891', '20133840'], virus NS1 (183 results) D ['20702615'], virus NS1 (183 results) S ['21464929', '20444891'], host antiviral response (70 results) D ['18725644'], NS1 (1913 results) S ['21464929'], influenza virus NS1 (61 results) D ['16715094', '14645908'], short heterologous dimerization domains (7 results) S ['12438621'], short heterologous dimerization (16 results) S ['12438621']
JE	TRUE	4	TRUE	recombination similarities (1323 results) S ['21464928'], evolution of recombination (12345 results) S ['21464928'], recombination rates (781 results) S ['21464928'], recombination landscape (14 results) D ['15723063'], recombination landscape (14 results) S ['21464928'], recombination rate (1017 results) S ['21464928']
JE	TRUE	1	TRUE	sex hormones on antlerogenic (2 results) D ['11703073', '10368936'], antler growth stage (18 results) D ['11789985'], antler growth stage (18 results) S ['21464927'], mitogenic effect of sex (79 results) D ['11703073'], antler cells (7 results) D ['11735240', '7836891'], antler cells (7 results) S ['21464927', '10607941'], hormones on antlerogenic (3 results) D ['17522075', '11703073', '10368936'], antler growth (101 results) D ['17120051', '16313394', '16191163', '15949530'], antler growth (101 results) S ['21464927', '19124089'], antler cell proliferation (32 results) D ['16191163'], antler cell proliferation (32 results) S ['21464927'], proliferation of antlerogenic (2 results) D ['11703073', '10368936'], antlerogenic cells (8 results) D ['11703073', '10368936'], antlerogenic (27 results) D ['17522075', '15949530', '11789985', '11703073', '10368936'], proliferative potential of ovine (18 results) D ['15332606'], osteoblast-like cells from facial (20 results) D ['19075324'], antler (488 results) S ['21464927']
JE	TRUE	1	TRUE	TWIST1 (436 results) S ['21464926'], TWIST1 overexpression (55 results) D ['20140954'], adjuvant studies C-02 (4 results) D ['10334518'], tyrosine kinase receptor EphA4 (4 results) D ['15313894'], nodal invasion (92 results) D ['19002529'], behavior of TWIST1 (15 results) D ['14601057'], TWIST1 hypermethylation (21 results) D ['20140954'], Project adjuvant Studies C-02 (3 results) D ['10334518'], kinase receptor EphA4 (4 results) D ['15313894'], TWIST1 expression levels (82 results) D ['20818389'], TWIST1 expression levels (82 results) S ['21464926'], studies C-02 (8 results) D ['10334518'], TWIST1 expression (20 results) D ['20140954'], TWIST1 expression (20 results) S ['21464926']

PMC3065457	1	FALSE	15	FALSE		4	14	FALSE	14	TRUE	13	TRUE	13	TRU
PMC3065456	1	FALSE	12	FALSE		5	12	TRUE	11	TRUE	10	TRUE	8	TRU
PMC3065455	16	FALSE	19	FALSE		14	35	TRUE	27	TRUE	23	TRUE	16	TRU

JE	TRUE	8	TRUE	induces salivary gland dysfunction (37 results) S ['19950301'], Sjogren's (11029 results) S ['21464925'], Interleukin-12 induces salivary (1 results) S ['19950301'], dysfunction of Sjogren's (1591 results) S ['21464925'], Ro60 peptide (34 results) S ['21464925'], Interleukin-12 induces salivary gland (1 results) S ['19950301'], salivary gland dysfunction (178 results) S ['21464925'], 60-kDa (3919 results) S ['21464925'], gland dysfunction (536 results) S ['21464925'], gland dysfunction of Sjogren's (458 results) S ['21464925'], Sjogren's syndrome (10912 results) S ['21464925'], model of Sjogren's (401 results) S ['21464925'], new model of Sjogren's (73 results) S ['19950301'], anti-Ro ribonucleoprotein autoimmunity (43 results) D ['10084034'], anti-Ro ribonucleoprotein autoimmunity (43 results) S ['16339583'], Ro60 peptide immunization (7 results) S ['21464925']
JE	TRUE	4	TRUE	deficient acetaminophen glucuronidation (2 results) S ['10862526', '9174118'], UGT1A6 amino (35 results) S ['21464924', '11903872'], UGT1A6 exon sequences (11 results) S ['10862526'], UGT1A1 (1055 results) S ['21464924'], Felidae (367 results) S ['21464924'], UGT1A6 exon (25 results) S ['11903872', '10862526'], Felidae UGT1A6 (3 results) S ['21464924', '10862526'], gene animal-plant molecular (10 results) D ['2196721'], acetaminophen glucuronidation (35 results) S ['10862526', '9174118'], UGT1A6 gene (13 results) S ['11903872'], UGT1A6 amino acid (50 results) S ['21464924'], UGT1A6 (361 results) S ['21464924'], pseudogene (3133 results) S ['21464924']
JE	TRUE	10	TRUE	tail regeneration of Gekko (11 results) S ['21464923', '19712730', '16757806'], Proximodistal identity during vertebrate (27 results) D ['19771161', '17631442', '16107473', '15733667', '12408806', '9082990'], identity during amphibian (1407 results) S ['21464923'], CD59 (1669 results) S ['21464923'], proximodistal identity during amphibian (14 results) D ['19771161', '17631442', '16107473', '15733667', '12408806', '9082990'], proximodistal identity during amphibian (14 results) S ['21464923'], ortholog of CD59 (2 results) D ['12408806'], salamander limb regeneration (10 results) D ['19771161', '17631442'], vertebrate-like thyroid (3 results) D ['19733626'], newt ortholog (2 results) D ['12408806'], japonicus (2160 results) S ['21464923'], regeneration of Gekko (14 results) S ['21464923', '19712730', '16757806'], amphibian limb (133 results) S ['21464923'], Gekko japonicus (15 results) S ['21464923', '19712730', '16757806'], Positional identity of adult (44 results) D ['19771161', '19571878', '17631442'], Positional identity of adult (44 results) S ['21464923'], cord of Gekko (24 results) S ['21464923', '19712730', '16757806'], expression of Pbx4 (14 results) S ['19712730'], blastema cells of urodeles (24 results) D ['9862713', '9082990'], Positional identity (238 results) D ['19771161', '19571878'], spinal cord of Gekko (24 results) S ['21464923', '19712730', '16757806'], limb regeneration (556 results) S ['21464923'], tail regeneration (148 results) S ['21464923', '19712730'], CD59-like gene (4 results) D ['16876248'], proximodistal identity (11 results) D ['17631442', '16107473', '15733667', '12408806', '9082990'], proximodistal identity (11 results) S ['21464923'], pig analogue of relevance (19 results) D ['9558099'], salamander limb (20 results) D ['19771161', '17631442'], spinal cord during tail (2187 results) S ['21464923'], vertebrate-like thyroid hormone (3 results) D ['19733626'], newt ortholog of CD59 (2 results) D ['12408806'], proximodistal (573 results) S ['21464923'], Meis homeodomain proteins (3 results) D ['16107473'], Meis homeodomain proteins (3 results) S ['19712730'], cord during tail (2338 results) S ['21464923']

PMC3065452	14	FALSE	22	FALSE		13	36	TRUE	30	TRUE	25	TRUE	20	TRU
PMC3065451	5	FALSE	4	FALSE		5	8	FALSE	8	FALSE	8	TRUE	7	TRU
PMC3065450	2	FALSE	20	FALSE		4	21	TRUE	19	TRUE	17	TRUE	16	TRU
PMC3070435	4	FALSE	5	FALSE		9	9	TRUE	6	TRUE	6	TRUE	5	TRU

JE	TRUE	7	TRUE	sett density (2 results) D ['17349705'], bovine tuberculosis (2596 results) S ['21464920'], bTB risk (6 results) S ['21464920'], culling (1618 results) S ['21464920'], tuberculosis breakdowns among cattle (34 results) D ['20399521', '16946308'], tuberculosis breakdowns among cattle (34 results) S ['21464920', '17148126'], breakdowns among cattle (49 results) D ['20399521', '16946308'], breakdowns among cattle (49 results) S ['21464920', '17148126'], Herd-level risk factors (43 results) S ['17148126'], Donnelly (5421 results) S ['21464920'], tuberculosis on cattle (2894 results) S ['21464920'], tuberculosis breakdowns (6 results) D ['9234430'], tuberculosis breakdowns (6 results) S ['17148126'], RBCT (37 results) D ['20399521'], RBCT (37 results) S ['21464920', '20161769'], persistent bovine tuberculosis (22 results) D ['17349705'], persistent bovine tuberculosis (22 results) S ['20161769', '19204342'], herd bTB breakdown (10 results) D ['20399521', '17349705', '15979173', '15158568'], herd bTB breakdown (10 results) S ['21464920'], proactive cull (2 results) S ['21464920'], bTB breakdown (4 results) D ['17349705', '15158568'], bTB breakdown (4 results) S ['21464920'], Herd-level (446 results) S ['21464920'], proactive badger cull (2 results) S ['21464920'], cattle herds (561 results) S ['21464920'], herd bTB (50 results) D ['20399521'], herd bTB (50 results) S ['21464920'], badger sett density (2 results) D ['17349705'], bTB breakdown risks (14 results) D ['20399521', '17349705', '15979173', '15158568'], bTB breakdown risks (14 results) S ['21464920'], breeds under field (61 results) D ['17761523'], Herd-level risk (44 results) S ['17148126'], farm management factors (20 results) D ['12322923'], bTB (1013 results) S ['21464920'], proactive badger (14 results) D ['20399521', '15748755'], proactive badger (14 results) S ['21464920']
JE	TRUE	7	TRUE	Lymphocytes Responses against Nef (168 results) D ['20173754'], Lymphocytes Responses against Nef (168 results) S ['21464919'], Depth of Cytotoxic (178 results) S ['21464919'], Mosaic HIV-1 (177 results) D ['20173752'], Mosaic HIV-1 vaccines (17 results) D ['20173752', '17187074'], Inclusion of Epitope (848 results) S ['21464919'], HIV-1 epitopes from diverse (144 results) D ['20173752'], Epitope Variant Sequences (641 results) S ['21464919'], variant HIV-1 epitopes (1 results) D ['15265928']
JE	TRUE	9	TRUE	disease Response outlines (251 results) S ['21464918'], response program effectiveness (6007 results) S ['21464918'], Timpka (161 results) S ['21464918', '19551239'], bioterrorism threat spectrum (9 results) D ['21249234'], information infrastructure for pandemic (23 results) S ['21464918'], infrastructure for Pandemic (115 results) S ['21464918'], analyses of outbreak (1509 results) S ['21464918'], pandemic response (56 results) S ['21464918'], community-level analyses of outbreak (2 results) S ['21464918'], generic health service model (402 results) S ['21464918'], technical components (82 results) S ['21464918'], aberrancy-detection algorithms (2 results) D ['18755992'], outbreak detection (179 results) S ['21464918'], information infrastructure (262 results) S ['21464918'], response program components (1328 results) S ['21464918'], PROSPER Protocol for Implementation (1 results) S ['21464918'], infectious disease Response outlines (30 results) S ['21464918'], response programs (52 results) S ['21464918'], PROSPER Protocol (7 results) S ['21464918'], local pandemic response programs (12 results) S ['21464918'], pandemic evidence levels (74 results) S ['21464918'], pandemic response programs (98 results) S ['21464918']
JE	TRUE	5	TRUE	abdominal pain relation (2608 results) S ['21468324'], young patients with RAP (20 results) D ['11433080', '9627595'], Recurrent abdominal symptom subtypes (5 results) D ['14734882'], psychosomatic symptoms among children (769 results) S ['21468324'], recurrent abdominal (8838 results) S ['21468324'], Huertas-Ceballos (7 results) D ['18254014', '18254012', '11869650'], cognitive-behavioral family intervention (5 results) D ['15944167', '8201068'], recurrent abdominal pain (1275 results) S ['21468324'], abdominal pain prevalence (6399 results) S ['21468324']

PMC3073875	9	FALSE	8	FALSE		5	16	TRUE	11	TRUE	9	TRUE	6	TRU
PMC3072938	0	TRUE	19	FALSE		2	19	TRUE	15	TRUE	15	TRUE	14	TRU
PMC3074558	10	FALSE	7	FALSE		7	17	TRUE	15	TRUE	10	TRUE	9	TRU
PMC3078835	2	FALSE	10	FALSE		2	10	TRUE	9	TRUE	9	TRUE	8	TRU
PMC3072740	6	FALSE	13	FALSE		9	17	TRUE	16	TRUE	16	TRUE	15	TRU

JE	TRUE	4	TRUE	IMRT DQA (1 results) S ['21439096'], Radiat Oncol (368 results) S ['21439096'], irregular IMRT beamlets (4 results) D ['15487721'], narrow irregular IMRT (2 results) D ['15487721'], phantom of acryl (10 results) S ['21439096'], report from AAPM (181 results) D ['19994544'], IMRT multiple institution (12 results) D ['19994544'], narrow irregular IMRT beamlets (2 results) D ['15487721'], ion-chamber (691 results) S ['21439096'], dosimetric gap (62 results) S ['21439096'], same fluence maps (15 results) S ['21439096'], AAPM (612 results) S ['21439096'], cylindrical phantom of acryl (2 results) S ['21439096'], IMRT Subcommittee (4 results) D ['12945975'], irregular IMRT (35 results) D ['18406906', '15487721'], dosimetry comparisons (11 results) D ['19994544'], multiple institution (10 results) D ['19994544']
JE	TRUE	7	TRUE	amongst maternal health policy (29 results) S ['21443794'], maternal health policy makers (408 results) S ['21443794'], local academic obstetricians (4 results) S ['21443794'], academic obstetricians (3 results) S ['21443794'], local academic obstetrics (91 results) S ['21443794'], LMICs (81 results) S ['21443794'], academic obstetrics (37 results) S ['21443794'], particular fields of clinical (723 results) S ['21443794'], practice amongst maternal (127 results) S ['21443794'], evidence base for obstetrics (243 results) S ['21443794'], local researchers (27 results) S ['21443794', '19091083'], amongst maternal health (251 results) S ['21443794'], practice amongst maternal health (62 results) S ['21443794'], senior academic obstetricians (5 results) S ['21443794'], base for obstetrics (3030 results) S ['21443794'], South African academic obstetrics (14 results) S ['21443794'], African academic obstetrics (38 results) S ['21443794'], amongst maternal (787 results) S ['21443794'], African academic obstetricians (2 results) S ['21443794']
JE	TRUE	3	TRUE	trauma team's admission (4 results) D ['17714579'], trauma team (466 results) S ['21439095'], undertriage (90 results) S ['21439095'], pre-hospital trauma triage (6 results) D ['17711567'], trauma team activation guidelines (8 results) D ['19134177'], trauma team activation guidelines (8 results) S ['16691271'], team activation (90 results) D ['20406456'], team activation (90 results) S ['21439095'], trauma team activation (74 results) D ['20406456', '19134177'], trauma team activation (74 results) S ['21439095'], TTA criteria (9 results) D ['17714579'], identify suboptimal criteria (174 results) S ['21439095'], overtriage (108 results) S ['21439095'], international emergency medicine literature (6 results) D ['15795728'], TTA protocol (14 results) D ['17714579'], precision of TTA (5 results) D ['19134177', '17714579'], team activation guidelines (29 results) D ['19134177']
JE	TRUE	6	TRUE	parenteral antithrombotic (92 results) S ['21443789'], fondaparinux (997 results) S ['21443789'], different LMWH agents (355 results) S ['21443789'], biosimilar (134 results) S ['21443789'], parenteral anticoagulant (23 results) S ['21443789'], heparin heparins enoxaparin (532 results) S ['21443789'], parenteral antithrombotic regimens (4 results) S ['21443789'], EXPERT Study Investigators (1 results) D ['18042845'], dalteparin (925 results) S ['21443789'], newer parenteral anticoagulant (12 results) S ['21443789'], deep peripheral nerve (1 results) D ['18042845'], enoxaparin (2974 results) S ['21443789']
JE	TRUE	12	TRUE	clinical use of ALC (268 results) S ['21490942'], Superiority of L-propionylcarnitine (1 results) D ['1555624'], therapeutic use of ALC (272 results) S ['21490942'], joint editorial statement (20 results) D ['10477541'], cerebral ischemia (14979 results) S ['21490942'], cardiovascular effects of ALC (22 results) S ['21490942'], effects of ALC (439 results) S ['21490942'], cardiovascular effects of L-carnitine (904 results) S ['21490942'], carnitine (11149 results) S ['21490942'], TAG liver content (108 results) S ['19545458'], use of PLC (9477 results) S ['21490942'], MHIQ scores (4 results) D ['9070558'], L-carnitine (3040 results) S ['21490942'], effects of PLC (4444 results) S ['21490942'], primed-constant infusion of ALC (1 results) D ['10877193'], use of ALC (1158 results) S ['21490942'], muscular compressions (85 results) D ['18506129'], peripheral arterial disease (4262 results) S ['21490942'], intravenous PLC (69 results) D ['18506129', '18502605', '16518521']

PMC3070138	3	FALSE	25	FALSE		4	23	TRUE	19	TRUE	18	TRUE	17	TRU
PMC3070137	0	TRUE	10	FALSE		1	10	FALSE	10	FALSE	10	FALSE	10	TRU
PMC3070136	9	FALSE	16	FALSE		7	22	TRUE	19	TRUE	15	TRUE	10	TRU
PMC3070135	4	FALSE	12	FALSE		4	16	FALSE	16	FALSE	16	FALSE	16	TRU
PMC3070134	4	FALSE	3	FALSE		9	6	TRUE	5	TRUE	5	TRUE	2	TRU

JE	TRUE	10	TRUE	tracheoesophageal (3996 results) S ['21472133'], inter-pouch (1 results) S ['21472133'], esophageal atresia (3071 results) S ['21472133'], inter-pouch gaps (1 results) S ['21472133'], MPVR (31 results) S ['21472133'], TEF (1106 results) S ['21472133'], TL-VR (1 results) S ['21472133'], upper pouch fistula (3 results) D ['15723217'], multiple planar volume reconstruction (19 results) S ['21472133'], congenital esophageal (2736 results) S ['21472133'], multiple planar volume (138 results) S ['21472133'], transparency lung (45 results) S ['21472133'], three-dimensional transparency lung (4 results) S ['21472133'], TL-VR reconstruction (1 results) S ['21472133'], three-dimensional transparency lung volume (3 results) S ['21472133'], tomography three-dimensional volume (6341 results) S ['21472133'], distal TEF (23 results) D ['15517291'], distal TEF (23 results) S ['21472133'], tracheoesophageal fistula (3197 results) S ['21472133'], congenital esophageal atresia (140 results) S ['21472133'], transparency lung volume (6 results) S ['21472133'], planar volume reconstruction (131 results) S ['21472133'], distal esophageal pouches (3 results) S ['21472133'], distal tracheoesophageal (291 results) S ['21472133'], distal tracheoesophageal fistula (97 results) S ['21472133'], esophageal pouches (21 results) D ['17579871'], esophageal pouches (21 results) S ['21472133'], TL-VR reconstruction of MDCT (1 results) S ['21472133']
JE	TRUE	9	TRUE	colorectal adenocarcinoma (1726 results) S ['21472132'], carcinoembryonic antigen (15779 results) S ['21472132'], serum carcinoembryonic antigen level (95 results) S ['21472132'], carcinoembryonic (15880 results) S ['21472132'], carcinoembryonic antigen level (271 results) S ['21472132'], perioperative serum total protein (193 results) S ['21472132'], colorectal adenocarcinoma after surgical (1297 results) S ['21472132'], antigen level (1929 results) S ['21472132'], short-term outcomes of surgical (3194 results) S ['21472132'], younger group (2109 results) S ['21472132']
JE	TRUE	7	TRUE	factor family genes (7 results) S ['16830362'], TFF mRNA (5 results) S ['21472131'], TFF (271 results) S ['21472131'], proinvasive activity (14 results) D ['12881705'], TFF3 promoter hypomethylation (2 results) D ['20112343'], cholangiocarcinoma via Opisthorchis (18 results) S ['15957169'], TFF2 (203 results) S ['21472131'], progression of CCA (137 results) S ['21472131'], ducts relates (35 results) D ['15101998'], trefoil (1320 results) S ['21472131'], Site-characteristic expression (1 results) D ['15101998'], CCA cell line (9 results) S ['21472131'], Limpaiboon (35 results) S ['21472131', '16830362'], cDNA TFF3 (44 results) D ['12000726'], peptides pS2 (12 results) D ['12881705'], bile ducts relates (14 results) D ['15101998'], trefoil peptides pS2 (12 results) D ['12881705'], TFF3 (491 results) S ['21472131'], TFF gene (8 results) S ['21472131'], Kosriwong (1 results) S ['21472131'], TFF1 (749 results) S ['21472131'], trefoil factor (696 results) S ['21472131'], trefoil factor family genes (1 results) S ['16830362'], intrahepatic bile ducts relates (8 results) D ['15101998'], rhTFF2 (2 results) S ['21472131']
JE	TRUE	11	TRUE	instance allows resolution (36 results) S ['21472130'], abscesses (16636 results) S ['21472130'], percutaneous therapy (216 results) S ['21472130'], radiological drainage (28 results) S ['21472130'], liver abscesses (1212 results) S ['21472130'], pyogenic (5252 results) S ['21472130'], pyogenic liver abscesses (211 results) D ['20569937'], pyogenic liver abscesses (211 results) S ['21472130'], cryptogenic (3984 results) S ['21472130'], initial percutaneous therapy (4859 results) S ['21472130'], coli pyogenic liver abscesses (95 results) D ['20569937', '19826410'], coli pyogenic liver abscesses (95 results) S ['21472130'], cryptogenic origin (27 results) D ['16059789'], pyogenic liver (660 results) S ['21472130'], radiological intervention (165 results) D ['20569937'], Sixty-three patients males (3349 results) S ['21472130']
JE	TRUE	2	TRUE	survivin splice variants (20 results) D ['15856009', '15688031', '12115583'], survivin splice (2 results) D ['15856009'], splice variants (4543 results) S ['21472129'], human anti-apoptopsis gene (1 results) D ['14741722'], expression of survivin (2802 results) S ['21472129'], survivin isoforms (18 results) D ['17656368', '17151952', '16291752', '15809755', '15688031'], survivin isoforms (18 results) S ['21472129']

PMC3070133	10	FALSE	4	FALSE		5	13	TRUE	12	TRUE	11	TRUE	8	TRU
PMC3070132	4	FALSE	12	FALSE		5	16	TRUE	15	TRUE	15	TRUE	15	TRU
PMC3070131	10	FALSE	17	FALSE		7	24	TRUE	15	TRUE	15	TRUE	14	TRU
PMC3070130	0	TRUE	16	FALSE		1	15	FALSE	15	FALSE	15	TRUE	14	TRU

JE	TRUE	4	TRUE	profibrotic chemokines (3 results) D ['19035500'], sclerosis induce fibroblasts (84 results) D ['19035500'], other form of liver (6304 results) S ['21472128'], vascular rarefaction characteristic (17 results) D ['19587802'], rarefaction characteristic (66 results) D ['19587802'], downstream mediator of profibrotic (20 results) D ['18050250'], rarefaction characteristic of systemic (6 results) D ['19587802'], SARI values (203 results) S ['21472128'], splenomegaly (14982 results) S ['21472128'], systemic sclerosis induce fibroblasts (64 results) D ['19035500', '18050250'], splenic artery (3979 results) S ['21472128'], N-acetylcysteine infusion reduces (17 results) D ['19730428'], partial exploitation of toll-like (1 results) D ['19035500'], exploitation of toll-like (25 results) D ['19035500']
JE	TRUE	8	TRUE	acute diverticulitis hospitalizations (101 results) S ['21472127'], hospitalizations for diverticulitis (244 results) S ['21472127'], inpatient sample (1128 results) S ['21472127'], burden of diverticulitis (5 results) D ['19468998', '19212172'], nationwide inpatient (845 results) S ['21472127'], hospital admissions for diverticulitis (36 results) D ['19212172', '16556182', '12752356'], hospital admissions for diverticulitis (36 results) S ['21472127'], admissions for diverticulitis (45 results) D ['19212172', '16556182'], admissions for diverticulitis (45 results) S ['21472127'], diverticulitis (5964 results) S ['21472127'], diverticulitis-associated (23 results) S ['21472127'], nationwide inpatient sample (823 results) S ['21472127'], diverticulitis admissions (45 results) D ['19212172', '16556182'], diverticulitis admissions (45 results) S ['21472127'], rates of diverticulitis (212 results) S ['21472127'], epidemiological trends (338 results) S ['21472127']
JE	TRUE	7	TRUE	motilin (1717 results) S ['21472126'], Motilin effects (4 results) D ['12519743'], expression of motilin (65 results) S ['21472126'], cholecystokinin-8 on antropyloroduodenal (1 results) D ['18957613'], Dose-dependent effects of cholecystokinin-8 (16 results) D ['18957613'], gastrointestinal motility (19274 results) S ['21472126'], intestinal propulsion (122 results) S ['21472126'], psychophysiological stress experiment (45 results) D ['11330489'], capsaicin-sensitive vagal pathway (4 results) D ['3136661'], mosapride (167 results) S ['21472126'], treatment with Simotang (1 results) D ['17005087'], motilin level (36 results) D ['14760770'], motilin level (36 results) S ['21472126'], mosapride group (4 results) S ['21472126'], serum motilin (28 results) S ['21472126'], CCK (9629 results) S ['21472126'], serum motilin level (33 results) S ['21472126'], effect of Simotang (2 results) D ['17005087'], effect of Simotang (2 results) S ['21472126'], stress group mosapride (1 results) S ['21472126'], cholecystokinin (16134 results) S ['21472126'], Simotang (2 results) D ['17005087'], Simotang (2 results) S ['21472126'], Simotang group (2 results) D ['17005087'], Simotang group (2 results) S ['21472126'], group mosapride (37 results) S ['21472126'], CCK expression level (263 results) S ['21472126']
JE	TRUE	14	TRUE	hypobaric (2240 results) S ['21472125'], mucosal injury (2872 results) S ['21472125'], intestinal mucosal (2871 results) S ['21472125'], bacterial translocation (2603 results) S ['21472125'], High-altitude over-starvation (1 results) S ['21472125'], Gln (10107 results) S ['21472125'], hypoxia on intestinal (1406 results) S ['21472125'], oxidase malondialdehyde (9628 results) S ['21472125'], treatment with Gln (1283 results) S ['21472125'], high altitude (6575 results) S ['21472125'], intestinal injury (858 results) S ['21472125'], hypobaric hypoxia (1342 results) S ['21472125'], endotoxin translocation (108 results) S ['21472125'], hypobaric hypoxia on intestinal (13 results) S ['21472125'], intestinal mucosa (57858 results) S ['21472125'], intestinal mucosal injury (187 results) S ['21472125']

PMC3070129	4	FALSE	19	FALSE		9	22	TRUE	18	TRUE	17	TRUE	13	TRU
PMC3070128	4	FALSE	12	FALSE		5	14	TRUE	12	TRUE	9	TRUE	8	TRU
PMC3070125	6	FALSE	2	FALSE		4	7	TRUE	5	TRUE	5	TRUE	4	TRU
PMC3070120	11	FALSE	3	FALSE		5	14	TRUE	13	TRUE	12	TRUE	9	TRU
PMC3070119	13	FALSE	7	FALSE		7	20	TRUE	17	TRUE	14	TRUE	12	TRU

JE	TRUE	7	TRUE	dilution potential technique (768 results) S ['21472124'], ROCK pathways (15 results) S ['19339512', '18353901'], aqueous solutions across Caco-2 (7 results) D ['10907227'], omeprazole (9352 results) S ['21472124'], modulates paracellular (66 results) S ['18353901'], omeprazole on intestinal (508 results) S ['21472124'], Prolactin stimulates transepithelial calcium (3 results) S ['18353901'], Caco-2 epithelium (1149 results) S ['21472124'], paracellular (3726 results) S ['21472124'], Caco-2 (8932 results) S ['21472124'], PKCzeta-dependent transcellular (1 results) S ['19339512'], permeability from claudin-16 (19 results) D ['19538300', '18188451', '16234325'], Prolactin stimulates transepithelial (3 results) S ['18353901'], activation energy for passive (198 results) S ['21472124'], modulates paracellular permselectivity (2 results) S ['18353901'], Caco-2 mediation (17 results) S ['18353901'], Magnesium transport from aqueous (60 results) D ['12609869'], transepithelial calcium (58 results) S ['18353901', '17488805'], stimulates transepithelial calcium transport (51 results) S ['18353901'], paracellular permeability from claudin-16 (17 results) D ['19538300', '18188451', '16234325'], transepithelial calcium transport (40 results) S ['18353901', '17488805'], stimulates transepithelial calcium (67 results) S ['18353901'], paracellular permselectivity (2 results) S ['18353901']
JE	TRUE	5	TRUE	reduces macromolecular hyperpermeability (2 results) S ['10338220'], conversion of glutamine (776 results) S ['21472123'], macromolecular hyperpermeability (32 results) S ['10338220'], Glutamine reduces macromolecular (6 results) S ['10338220'], Differential abilities of phorbol (14 results) D ['11159699'], subsequent uptake of glutamate (248 results) S ['21472123'], extracellular conversion of glutamine (57 results) S ['21472123'], paracellular (3726 results) S ['21472123'], dipetide Ala-Gln (1 results) S ['16115328'], Glutamine reduces macromolecular hyperpermeability (1 results) S ['10338220'], paracellular hyperpermeability (37 results) S ['21472123'], hyperpermeability (947 results) S ['21472123'], cow's milk secrete (7 results) D ['8194697'], effect of glutamine (6667 results) S ['21472123'], milk secrete tumor necrosis (8 results) D ['8194697'], cow's milk secrete tumor (3 results) D ['8194697']
JE	TRUE	3	TRUE	anti-core antibody response (3 results) D ['19919328'], occult HBV infection involves (3 results) D ['10807510'], detection of occult (3386 results) S ['21472120'], Superiority of minipool (1 results) D ['12757502'], occult hepatitis (319 results) S ['21472120'], anti-core (195 results) D ['19919328'], anti-core antibody (37 results) D ['19919328'], role of anti-core (18 results) D ['19919328']
JE	TRUE	4	TRUE	new ultra sensitive real-time (14 results) D ['20226057'], Control of cccDNA (37 results) D ['19616338'], sensitive real-time PCR (88 results) D ['20226057'], ultra sensitive real-time PCR (18 results) D ['20226057'], cccDNA function (2 results) D ['19616338'], complex entity with relevant (81 results) S ['21472115'], sensitive real-time PCR assay (20 results) D ['20226057'], Review Control of cccDNA (9 results) D ['19616338'], Review Occult hepatitis (99 results) D ['20413301', '19615780'], quantification of HBV-DNA (157 results) D ['20226057'], ultra sensitive real-time (55 results) D ['20226057'], infection among hepatopathy (64178 results) S ['21472115'], occult hepatitis (319 results) S ['21472115'], Pollicino (111 results) D ['19616338']
JE	TRUE	7	TRUE	deacetylase inhibitors induce microglial (2 results) D ['17850978'], transporter GLUT1 suggests (93 results) D ['19774643'], defense barrier (59 results) S ['21472114'], role of butyrate (1279 results) S ['21472114'], MUC2 mucin production (3 results) D ['17374366'], effects of butyrate (6124 results) S ['21472114'], glucose transporter GLUT1 suggests (88 results) D ['19774643'], stimulates MUC2 mucin (10 results) D ['17374366'], butyrate production (144 results) D ['20937167'], stimulates MUC2 (10 results) D ['17374366'], stimulates MUC2 mucin production (5 results) D ['17374366'], Short-chain fatty (3154 results) S ['21472114'], metabolic switch from butyrate (6 results) D ['19774643'], Butyrate transport deficiency (14 results) D ['19774643'], butyrate deficiency results (37 results) D ['19774643', '10734024'], butyrate (8722 results) S ['21472114'], visceral perception (160 results) D ['19460106'], inhibitors induce microglial apoptosis (18 results) D ['17850978'], colonic defense barrier (44 results) S ['21472114'], colonic defense (678 results) S ['21472114']

PMC3078896	5	FALSE	1	FALSE		5	4	TRUE	2	TRUE	2	TRUE	2	TRU
PMC3076010	6	FALSE	20	FALSE		8	25	TRUE	17	TRUE	15	TRUE	15	TRU
PMC3066556	1	FALSE	0	TRUE		1	1	FALSE	1	FALSE	1	TRUE	0	TRU
PMC3076039	8	FALSE	3	FALSE		13	10	FALSE	10	FALSE	10	TRUE	8	TRU
PMC3078828	8	FALSE	10	FALSE		8	16	TRUE	15	TRUE	12	TRUE	8	TRU
PMC3074088	4	FALSE	8	FALSE		10	11	TRUE	10	TRUE	9	TRUE	6	TRU

JE	TRUE	1	TRUE	trefoil factor participates (3 results) D ['12034770'], hybrid polar (42 results) D ['19029824'], vivo evidence of metallopanstimulin-1 (1 results) D ['16914586'], evidence of metallopanstimulin-1 (1 results) D ['16914586'], cell cycle-dependent process (5 results) D ['7937935'], CDK2 (4747 results) S ['21439087']
JE	TRUE	12	TRUE	Archimedes procedure (115 results) S ['21494401'], breast volume measurement methods (224 results) S ['21494401'], mastectomy specimen volume (59 results) S ['21494401'], anatomic thermoplastic (55 results) S ['21494401'], half-elliptic cylinder (1 results) D ['10584814'], mammographic (6120 results) S ['21494401'], breast volume determination (3 results) D ['16874195', '7443851'], Archimedes (257 results) S ['21494401'], Grossman-Roudner device (3 results) D ['16411159'], Grossman-Roudner device (3 results) S ['21494401'], Mastectomy Cases Breast (4090 results) S ['21494401'], anthropometric method (73 results) S ['21494401'], mastectomy (24409 results) S ['21494401'], breast volume (330 results) S ['21494401'], mammographic method (4 results) S ['21494401'], different breast volume (837 results) S ['21494401'], Specimen volume after total (232 results) S ['21494401'], volume measurement (1273 results) S ['21494401'], specimen volume (106 results) S ['21494401'], Grossman-Roudner (3 results) D ['16411159'], Grossman-Roudner (3 results) S ['21494401'], different methods (20176 results) S ['21494401'], breast volume measurement (10 results) D ['17122543', '17029808', '9299007'], breast volume measurement (10 results) S ['21494401'], Grossman-Roudner device method (2 results) D ['16411159'], Grossman-Roudner device method (2 results) S ['21494401']
JE	FALSE	0	FALSE	pathological validation (14 results) D ['16824834']
JE	TRUE	4	TRUE	verruciformis human papillomavirus DNA (1 results) D ['10728604'], verruciformis-associated (64 results) D ['15735026', '15023775', '14618345'], relation between non-melanoma (106 results) D ['19551818', '18931088'], detection of epidermodysplasia (63 results) D ['14618345', '12020228'], papillomavirus (23769 results) S ['21499554'], beta-PV (28 results) D ['17412978'], CSCC (146 results) S ['21499554'], human papillomavirus (18127 results) S ['21499554'], beta-PV type (11 results) D ['17412978'], High prevalence of epidermodysplasia (35 results) D ['14618345', '12873884', '12807946'], variety of epidermodysplasia (12 results) D ['10417630', '8876633']
JE	TRUE	3	TRUE	compulsory licences (8 results) D ['16710551'], previous decade of activism (1 results) S ['21439089'], HIV medicines patents (30 results) D ['20309404', '20148090', '17620749'], HIV medicines patents (30 results) S ['21439089'], patents on medicines (160 results) S ['21439089'], Medicines Patent Pool (1 results) S ['21439089'], new ARVs (6 results) S ['21439089'], Patent Pool (15 results) D ['20309404', '20200606', '20148090'], Patent Pool (15 results) S ['21439089'], ARVs (322 results) S ['21439089'], generic versions (95 results) S ['21439089'], patent terms on medicines (17 results) D ['16710551'], health implications of TRIPS (34 results) D ['16710551'], patent holders (23 results) D ['20309404'], major international patent treaty (10 results) D ['11597292'], Medicines Patent (2 results) S ['21439089'], HIV medicines (16 results) D ['20309404', '20148090'], generic medicines (103 results) S ['21439089', '20840741']
JE	TRUE	5	TRUE	chronic HBV (2298 results) S ['21487538'], haplotype of PDCD1 (28 results) D ['20837075', '17468813', '17230193', '17024563'], Hua-Fa Yin (1 results) S ['21487538'], chronic HBV infection (1580 results) S ['21487538'], death-1 gene (19 results) D ['20837075', '17490403', '17024563', '15022318', '12402038'], death-1 gene (19 results) S ['21487538'], new haplotype of PDCD1 (4 results) D ['15818672'], CTLA4 single nucleotide polymorphisms (12 results) D ['18049163'], HBV infection (7473 results) S ['21487538'], genetic susceptibility of chronic (6345 results) S ['21487538'], association analysis method (7 results) S ['21487538'], susceptibility of chronic (12177 results) S ['21487538']

PMC3073936	4	FALSE	12	FALSE		4	16	TRUE	12	TRUE	11	TRUE	11	TRU
PMC3072314	7	FALSE	23	FALSE		4	27	TRUE	25	TRUE	23	TRUE	18	TRU
PMC3072346	2	FALSE	10	FALSE		10	12	FALSE	12	FALSE	12	TRUE	11	TRU
PMC3072306	5	FALSE	10	FALSE		3	12	TRUE	8	TRUE	8	TRUE	7	TRU

JE	TRUE	8	TRUE	subgroups of GERD (157 results) S ['21439078'], conclude GERD (533 results) S ['21439078'], intraesophageal (621 results) S ['21439078'], subtypes of GERD (36 results) S ['21439078'], conclude GERD patients (407 results) S ['21439078'], acid reflux characteristics (4 results) D ['12622762'], negative symptom index (5 results) D ['12622762'], negative symptom index (5 results) S ['21439078'], subtypes of NERD (6 results) S ['21439078'], psychological anticipation (30 results) S ['21439078'], pelvic visceral pain correlates (5 results) D ['18184777'], esophageal acid (747 results) S ['21439078'], visceral pain correlates (36 results) D ['18184777', '15765397'], different patterns of visceral (310 results) S ['21439078'], acid exposure (1751 results) S ['21439078'], GERD patients (621 results) S ['21439078']
JE	TRUE	8	TRUE	Urban Employee Basic Health (9 results) S ['21439077'], Basic Health Insurance scheme (40 results) S ['21439077'], Resident Basic Health (246 results) S ['21439077'], insurance reform on hospital (1641 results) S ['21439077'], cataract surgery numbers (167 results) S ['21439077'], New Cooperative Medical (26 results) D ['20459726', '20180938'], New Cooperative Medical (26 results) S ['21439077'], Employee Basic Health Insurance (87 results) S ['21439077'], number of Chongqing's (1 results) S ['21439077'], Urban Employee (273 results) S ['21439077'], rural group (152 results) S ['21439077'], Urban Resident Basic (2 results) D ['19551750'], Urban Resident Basic (2 results) S ['21439077'], Urban Resident Basic Health (15 results) D ['19551750'], Urban Resident Basic Health (15 results) S ['21439077'], Cooperative Medical Scheme (27 results) D ['20459726', '20180938'], Cooperative Medical Scheme (27 results) S ['21439077'], New Cooperative Medical Scheme (21 results) D ['20459726', '20180938'], New Cooperative Medical Scheme (21 results) S ['21439077'], Medical Scheme (51 results) D ['20459726', '20180938'], Medical Scheme (51 results) S ['21439077'], Basic Health Insurance (41 results) S ['21439077'], insurance reform (223 results) S ['21439077'], Chongqing's cataract surgeries (1 results) S ['21439077'], Basic Health (1447 results) S ['21439077'], number of cataract (2179 results) S ['21439077'], Urban Employee Basic (10 results) S ['21439077'], Resident Basic Health Insurance (13 results) D ['19551750'], Resident Basic Health Insurance (13 results) S ['21439077'], Chongqing's cataract (1 results) S ['21439077']
JE	TRUE	7	TRUE	Lamin suppression (37 results) S ['21439080'], CA099471 (13 results) S ['20174585', '19375154', '16698071'], lamin cells (2385 results) S ['21439080'], chromosomal numerical instability (296 results) S ['21439080'], nuclear morphological deformation (28 results) S ['21439080', '19581290'], lamin expression (38 results) D ['20074078', '18714339', '10579712', '10517909'], ovarian surface (1144 results) S ['21439080'], loss of lamin (266 results) S ['21439080'], nuclear envelope (7700 results) S ['21439080'], lamin gene (30 results) D ['11071918'], lamin (2487 results) S ['21439080'], nuclear morphology (1362 results) S ['21439080']
JE	TRUE	6	TRUE	Kinesin family member variant (13 results) D ['19371834'], member variant Trp719Arg (1 results) D ['19371834'], PTCHD3 (3 results) D ['17904097'], PTCHD3 (3 results) S ['21439084'], calnexin (1136 results) S ['21439084'], homozygous deletion (1720 results) S ['21439084'], PTCHD3 isoforms (2 results) D ['17904097'], PTCHD3 isoforms (2 results) S ['21439084'], fitness disease predisposition (247 results) S ['21439084'], shorter isoform (103 results) S ['21439084'], family member variant Trp719Arg (1 results) D ['19371834'], heterozygous deletions (147 results) S ['21439084'], CNV deletions (111 results) S ['21439084'], HGDP-CEPH (31 results) S ['21439084'], PTCHD3 deletions (1 results) S ['21439084']

PMC3073935	19	FALSE	12	FALSE		7	27	TRUE	21	TRUE	19	TRUE	17	TRU
PMC3078100	0	TRUE	1	FALSE		1	1	FALSE	1	FALSE	1	FALSE	1	FAL
PMC3077816	2	FALSE	3	FALSE		3	5	FALSE	5	TRUE	4	TRUE	4	TRU
PMC3077815	6	FALSE	10	FALSE		7	12	TRUE	10	TRUE	9	TRUE	7	TRU
PMC3077814	13	FALSE	3	FALSE		25	16	TRUE	14	TRUE	13	TRUE	12	TRU

JE	TRUE	13	TRUE	Pars interarticularis fenestration (3 results) D ['9442508'], extraforaminal nerve root (5 results) D ['1750006'], extraforaminal nerve root (5 results) S ['21439083'], posterior decompression (485 results) S ['21439083'], diagnosis of intraforaminal (94 results) D ['20043768'], cranio-dorsolateral (1 results) D ['12021876'], decompression surgery (556 results) S ['21439083'], intraforaminal (123 results) D ['20043768'], intraforaminal (123 results) S ['21439083'], extraforaminal nerve root compression (2 results) D ['1750006'], extraforaminal nerve root compression (2 results) S ['21439083'], cranio-dorsolateral lumbar disc (1 results) D ['12021876'], nerve root compression (664 results) S ['21439083'], treatment of intraforaminal (83 results) D ['20043768'], operative treatment of intraforaminal (11 results) D ['1750006'], decompression surgery for extraforaminal (40 results) D ['20043768'], decompression surgery for extraforaminal (40 results) S ['21439083'], extraforaminal (192 results) D ['20043768'], extraforaminal (192 results) S ['21439083'], translaminar approach (7 results) D ['12021876'], interarticularis fenestration (3 results) D ['9442508'], root compression (937 results) S ['21439083'], lateral disc technical note (19 results) D ['15830977', '12800006'], surgery for extraforaminal (156 results) D ['20043768'], surgery for extraforaminal (156 results) S ['21439083'], cranio-dorsolateral lumbar (1 results) D ['12021876'], extraforaminal nerve (126 results) D ['20043768'], extraforaminal nerve (126 results) S ['21439083'], protruded (1840 results) S ['21439083'], further surgical approach (6 results) D ['9442508'], cranio-dorsolateral lumbar disc herniations (1 results) D ['12021876']
.SE	TRUE	1	TRUE	molecular therapy of melanoma (2230 results) S ['21439082']
JE	TRUE	4	TRUE	coronary flow autoregulation (9 results) D ['1825626'], wide perfusion pressures (403 results) D ['20083726'], J-curve (146 results) S ['21499494'], autoregulation (7257 results) S ['21499494'], coronary pressure autoregulation (5100 results) S ['21499494']
JE	TRUE	6	TRUE	EUCATAX (1 results) S ['20824769'], stent thrombosis (2828 results) S ['21499496'], polymer for coronary (29497 results) S ['21499496'], BIO polymer stent (20 results) S ['21499496'], stent mal-apposition (3 results) S ['21499496'], identical metallic platform (1 results) D ['19670303'], drug-eluting (5646 results) S ['21499496'], clinical comparison of Nobori (4 results) D ['20031715', '19608483'], durable polymer for coronary (107 results) S ['21499496'], NOBORI Circ Cardiovasc Interv (1 results) D ['20031715'], durable polymer (35 results) D ['18765162'], comparison of Nobori (12 results) D ['20031715', '19608483'], EUCATAX trial (1 results) S ['20824769'], Angiographic Test Efficacy (340 results) S ['20824769'], DES with BIO (498 results) S ['21499496'], real-world 18-month clinical (7 results) D ['19463441']
JE	TRUE	6	TRUE	prorenin (941 results) S ['21499495'], direct renin (259 results) S ['21499495'], benefit of aliskiren (39 results) D ['20558431', '20498271', '20376075'], persistent effects of aliskiren (5 results) D ['18308634', '18237679'], renin (48294 results) S ['21499495'], ramipril-based (5 results) D ['19617271'], renin levels through signal (205 results) D ['20075844'], half-life of aliskiren (24 results) D ['20664534', '19617271', '18307734'], inhibitor aliskiren for rat (13 results) D ['20007350', '18653711', '18460596', '18235083', '18212269', '16940215', '16103264'], aliskiren on PRA (30 results) D ['20376075', '19617271', '19066408', '18611061', '18308634'], aliskiren-based (14 results) D ['20033075', '19617271', '17541390'], concentration of aliskiren (47 results) D ['20664534', '20418268', '19917876'], dose of aliskiren (116 results) D ['20664534', '20498271'], oral human renin (1937 results) D ['20409974'], comparable with irbesartan (63 results) D ['20007350', '18343241'], direct renin inhibition (57 results) D ['20409974', '20007350']

PMC3071328	1	FALSE	15	FALSE		2	16	FALSE	16	FALSE	16	FALSE	16	TRU
PMC3073867	1	FALSE	0	TRUE		1	1	FALSE	1	TRUE	0	TRUE	0	TRU
PMC3071336	6	FALSE	15	FALSE		10	21	TRUE	20	TRUE	17	TRUE	15	TRU
PMC3072134	0	TRUE	15	FALSE		4	15	TRUE	14	TRUE	13	TRUE	13	TRU
PMC3072133	5	FALSE	12	FALSE		7	16	TRUE	12	TRUE	10	TRUE	6	TRU

JE	TRUE	12	TRUE	serological test (1279 results) S ['21439029'], test results of rabies (639 results) S ['21439029'], success of rabies (69 results) S ['21439029'], vaccination of dogs (1866 results) S ['21439029'], pre-exposure immunity (148 results) S ['21439029'], antibody response of dogs (2093 results) S ['21439029'], threshold antibody titre (64 results) S ['21439029'], booster vaccination regimen (436 results) S ['21439029'], individual serological test (47907 results) S ['21439029'], serological test for rabies (793 results) S ['21439029'], antibody titres (4931 results) S ['21439029'], rabies vaccination (530 results) S ['21439029'], rabies vaccination of dogs (733 results) S ['21439029'], serological response (1220 results) S ['21439029'], serological response of dogs (91 results) D ['18006120'], serological response of dogs (91 results) S ['21439029']
JE	FALSE	0	FALSE	HIV-seropositive differential associations (8 results) D ['15688282']
JE	TRUE	9	TRUE	frequency-dependence of resistance (260 results) S ['21439045'], Goldman Triangle (50 results) S ['21439045'], upper airway shunt (14 results) S ['21439045'], eRIC model (374 results) S ['21439045'], Reactance Area (8 results) D ['20035146', '19489132', '19070356'], Reactance Area (8 results) S ['21439045', '12205573'], bronchodilator responses (112 results) D ['18331417'], bronchodilator responses (112 results) S ['21439045'], airway shunt (16 results) S ['21439045'], aRIC (659 results) S ['21439045'], eRIC model parameters (29 results) S ['21439045'], IOS data (10 results) S ['21439045'], aRIC model parameters (3 results) S ['21439045'], respiratory reactance (22 results) D ['8726154'], Impulse oscillometry (155 results) S ['21439045'], official American Thoracic Respiratory (24 results) D ['17545458'], reactance (754 results) S ['21439045'], IOS parameters (23 results) D ['18331417', '18076725'], IOS parameters (23 results) S ['21439045'], frequency respiratory reactance (144 results) D ['20394507', '19489132'], frequency respiratory reactance (144 results) S ['21439045']
JE	TRUE	10	TRUE	arterial blood gas levels (128 results) S ['21483617'], brain tissue haemodynamics (93 results) S ['19227488'], Wavelet phase synchronization (8 results) S ['20142164'], NIRS signals (49 results) S ['21483617'], Wavelet phase synchronization analysis (30 results) S ['20142164'], haemodynamics (6339 results) S ['21483617'], ABP (2550 results) S ['21483617'], blood gas levels (304 results) S ['21483617'], phase angle (1270 results) S ['21483617'], scalp flow (476 results) S ['21483617'], haemoglobin flow dynamics (104 results) S ['19629692'], cerebral haemodynamics (286 results) S ['21483617'], relationship between ABP (340 results) S ['21483617'], autoregulation (7257 results) S ['21483617'], tissue haemodynamics (2 results) S ['19227488']
JE	TRUE	4	TRUE	diffuse optical tomography (278 results) S ['21483616'], hyperspectral reconstruction shows (16 results) S ['21483616'], diffuse optical tomographic (19 results) D ['18040425'], diffuse optical tomographic (19 results) S ['17664563', '10795991'], optical tomography (1146 results) S ['21483616'], hyperspectral diffuse optical (10 results) S ['21483616'], hyperspectral reconstructions (2 results) S ['21483616'], parallel plane transmission evaluation (11 results) D ['12607841'], continuous-wave multispectral diffuse (3 results) D ['18059661', '14680175'], regularization parameters (73 results) S ['21483616'], regularization (1987 results) S ['21483616'], hyperspectral (599 results) S ['21483616'], hyperspectral diffuse optical tomography (1 results) S ['21483616'], optimal regularization (32 results) S ['21483616'], continuous-wave multispectral diffuse optical (3 results) D ['18059661', '14680175'], optimal regularization parameters (7 results) S ['21483616'], hybrid frequency wave clinical (2 results) D ['12607841']

PMC3076238	5	FALSE	12	FALSE		5	17	TRUE	14	TRUE	12	TRUE	12	TRU
PMC3073939	4	FALSE	8	FALSE		3	11	TRUE	10	TRUE	10	TRUE	8	TRU
PMC3071341	3	FALSE	8	FALSE		4	10	FALSE	10	FALSE	10	TRUE	7	TRU
PMC3074514	8	FALSE	5	FALSE		6	13	TRUE	8	TRUE	8	TRUE	7	TRU
PMC3070626	0	TRUE	11	FALSE		1	11	FALSE	11	TRUE	10	TRUE	9	TRU
PMC3076225	2	FALSE	10	FALSE		5	11	TRUE	9	TRUE	9	TRUE	6	TRU

JE	TRUE	6	TRUE	histidine-rich C-terminus (2 results) S ['21439037'], non-pathogenic bacterium Mycobacterium smegmatis (60 results) S ['21439037'], GroEL1 (38 results) D ['19717599', '19528065'], GroEL1 (38 results) S ['21439037'], sequence homolog of GroEL (268 results) D ['19528065'], non-pathogenic bacterium Mycobacterium (141 results) S ['21439037'], Hsp60 chaperone GroEL1 (3 results) S ['21439037'], prokaryotic native operon structure (3 results) S ['20085764'], GroEL2 (55 results) D ['19717599'], efficient recombinant protein expression (6264 results) S ['21439037'], oligomerization of mycobacterial (8 results) D ['19717599'], prokaryotic native operon (9 results) S ['20085764'], native operon structure (134 results) S ['20085764'], Mycobacterium smegmatis (2367 results) S ['21439037'], smegmatis (2733 results) S ['21439037'], chaperone GroEL1 (33 results) D ['19717599', '19528065', '16325580'], chaperone GroEL1 (33 results) S ['21439037']
JE	TRUE	7	TRUE	chemotherapy-refractory metastatic colorectal (18 results) D ['20619739'], third-line treatment for KRAS (23 results) S ['21439039'], statistical analysis on BRAF (19 results) D ['19223544'], PIK3CA mutational (147 results) S ['21439039'], additional non-responders (373 results) S ['21439039'], PIK3CA (636 results) S ['21439039'], uniform combination therapy cetuximab-irinotecan (1 results) S ['21439039'], consortium analysis (4 results) D ['20619739'], uniform combination therapy (973 results) S ['21439039'], PIK3CA mutations (179 results) S ['21439039'], retrospective consortium analysis (210 results) D ['20619739'], PIK3CA mutational analysis (128 results) S ['21439039']
JE	TRUE	3	TRUE	PGRP (170 results) S ['21439073'], EvolMAP (1 results) D ['18184685'], peptidoglycan recognition (244 results) S ['21439073'], PGRP evolution (20 results) D ['16930467', '12649138'], PGRP evolution (20 results) S ['21439073'], PGRP-S (19 results) D ['12649138'], vertebrate PGRPs (47 results) S ['21439073'], PGRP genes (53 results) S ['21439073'], PGRP proteins (163 results) S ['21439073'], invertebrate PGRPs (47 results) S ['21439073'], PGRPs (83 results) S ['21439073']
JE	TRUE	3	TRUE	Care Reminder Systems (4 results) D ['8601207'], Preventive Health Care Reminder (2 results) D ['8601207'], preventive care (2815 results) S ['21435277'], view preventive care (521 results) S ['21435277'], Voogdt-Pruis (3 results) D ['20040167'], clinical competences (12 results) S ['21435277'], 6-month lifestyle modification intervention (26 results) D ['20045151'], Galway Consensus international collaboration (3 results) D ['19477858'], Galway Consensus (44 results) D ['19477864', '19477858'], Health Care Reminder Systems (2 results) D ['8601207'], competences of doctors (121 results) S ['21435277'], 6-month lifestyle modification (39 results) D ['20045151'], practice organisation (52 results) S ['21435277']
JE	TRUE	5	TRUE	primary mental health (144 results) S ['21435273'], joint clinical (153 results) S ['21435273'], service linkages (15 results) S ['21435273'], clinical partnership formation (70 results) S ['21435273'], organisational level (67 results) S ['21435273'], expert reference group (6 results) S ['21435273'], primary care services (1033 results) S ['21435273'], primary mental health care (93 results) S ['21435273'], institutional environment of leadership (186 results) S ['21435273'], joint clinical problem (1773 results) S ['21435273'], partnership formation (32 results) S ['21435273']
JE	TRUE	3	TRUE	Illumina Hap550 (1 results) S ['21439053'], pathogenic CNVs (16 results) D ['19015223', '17597782'], pathogenic CNVs (16 results) S ['21439053', '17850622'], hybridization on mental (2754 results) S ['21439053'], AGH platforms (3 results) S ['17850622'], genomic hybridization on mental (2343 results) S ['21439053'], trios on Affymetrix (20 results) S ['21439053'], NimbleGen (75 results) S ['21439053'], X-chromosomal CNVs (4 results) S ['21439053'], Agilent (1081 results) S ['21439053'], autosomal CNVs (28 results) D ['19029900'], autosomal CNVs (28 results) S ['21439053']

PMC3073865	3	FALSE	14	FALSE		4	16	TRUE	13	TRUE	13	TRUE	12	TRU
PMC3074542	17	FALSE	6	FALSE		13	21	TRUE	19	TRUE	16	TRUE	15	TRU
PMC3076265	0	TRUE	14	FALSE		3	14	TRUE	13	TRUE	12	TRUE	9	TRU
PMC3078881	4	FALSE	19	FALSE		3	22	TRUE	20	TRUE	20	TRUE	19	TRU
PMC3078880	0	TRUE	8	FALSE		1	8	TRUE	7	TRUE	7	TRUE	7	TRU

JE	TRUE	8	TRUE	Human leptin forms complexes (5 results) D ['9724081'], RhoGDI2 (32 results) S ['21439033'], between-subject (1641 results) S ['21439033'], between-subject variability (350 results) S ['21439033'], random forests analysis (610 results) S ['21439033'], multiplex immunoassay data (174 results) S ['21439033'], MMP-3 (2469 results) S ['21439033'], PBMC proteome (17 results) S ['21439033', '18489134'], plasma ApoA4 (42 results) S ['21439033'], Proteomic methodological recommendations (2 results) S ['18489134'], 2D-electrophoresis (380 results) S ['21439033'], cola beverages on insulin-like (1 results) D ['19772696'], ApoA4 (85 results) S ['21439033'], obvious biomarkers (2070 results) S ['21439033'], CLIC1 (73 results) S ['21439033'], multiplex immunoassay (120 results) S ['21439033'], leptin forms complexes (5 results) D ['9724081']
JE	TRUE	6	TRUE	reference collection group B2- (22 results) D ['11029456'], non-pathogenic Escherichia coli strains (8 results) D ['11810263'], DksA controls gene (6 results) D ['16824105', '12193624'], ppGpp (918 results) D ['21299642'], ppGpp (918 results) S ['21439067'], island of enterohaemorrhagic (31 results) D ['16824105'], general stress (905 results) D ['21299642'], DksA controls gene expression (6 results) D ['16824105', '12193624'], ppGpp partition gene (1 results) D ['21299642'], virulence regulatory genes (8 results) D ['16824105'], rpoS (1091 results) D ['21299642'], rpoS (1091 results) S ['21439067'], uropathogen-associated (1 results) D ['11029456'], rpoS mutations (22 results) D ['16820496'], rpoS mutations (22 results) S ['21439067', '18757823', '17665171', '14557028', '11790751'], Escherichia sigmaS-dependent (30 results) D ['15716429'], ppGpp levels (73 results) D ['17360576'], ppGpp levels (73 results) S ['21439067'], Escherichia sigmaS-dependent genes (28 results) D ['15716429'], concentrations of ppGpp (107 results) S ['21439067'], general stress resistance (23 results) S ['11790751'], pathogenicity island of enterohaemorrhagic (31 results) D ['16824105'], DksA controls (7 results) D ['16824105', '12193624']
JE	TRUE	6	TRUE	poorer nutritional outcomes (75 results) S ['21439041'], nutritional outcomes (124 results) S ['21439041'], HIV negative counterparts (31 results) S ['21439041'], Agincourt (97 results) S ['21439041', '20338024'], documents poor nutritional status (12 results) S ['21439041'], negative counterparts (167 results) S ['21439041'], poor nutritional outcomes (4 results) S ['21439041'], poor nutritional (965 results) S ['21439041'], documents poor nutritional (20 results) S ['21439041'], HIV positive children (354 results) S ['21439041'], positive children (1112 results) S ['21439041'], people of Mozambican (18 results) S ['15319405'], determinants of nutritional (1699 results) S ['21439041'], study documents poor nutritional (9 results) S ['21439041']
JE	TRUE	11	TRUE	non-injection (185 results) S ['21439069'], median number of casual (31 results) S ['21439069'], National HIV Behavioral (30 results) S ['21439069', '19417579'], fewer casual male partners (42 results) D ['16826162'], fewer casual male partners (42 results) S ['21439069'], more casual male partners (511 results) S ['21439069'], HIV Behavioral Surveillance (36 results) S ['21439069', '19417579'], number of casual (602 results) S ['21439069'], partner number (14 results) S ['21439069'], casual partner (153 results) S ['21439069'], National HIV Behavioral Surveillance (30 results) S ['21439069', '19417579'], casual sex partners (78 results) S ['21439069'], non-injection drug (71 results) S ['21439069'], Behavioral Surveillance men (120727 results) S ['21439069'], HIV Behavioral Surveillance men (11363 results) S ['21439069'], sex partners (2381 results) S ['21439069'], Behavioral Surveillance (139 results) S ['21439069'], casual partner number (107 results) S ['21439069', '19417579'], November 2003-April MMWR (2 results) D ['16826162'], 2003-April MMWR (3 results) D ['16826162'], 2003-April MMWR Surveill Summ (1 results) D ['16826162'], median casual (155 results) S ['21439069'], median of casual (155 results) S ['21439069']
JE	TRUE	6	TRUE	primary level (458 results) S ['21439043'], antenatal HIV (182 results) S ['21439043'], antenatal HIV test (5 results) S ['21439043'], timing of antenatal (347 results) S ['21439043'], PMTCT programmes (44 results) S ['21439043'], PMTCT (398 results) S ['21439043'], HIV test (1629 results) S ['21439043'], ANC (1933 results) S ['21439043']

PMC3078096	3	FALSE	13	FALSE		4	16	FALSE	16	FALSE	16	TRUE	12	TRU
PMC3072952	0	TRUE	13	FALSE		1	13	FALSE	13	FALSE	13	TRUE	12	TRU
PMC3071322	0	TRUE	19	FALSE		1	19	TRUE	16	TRUE	16	TRUE	16	TRU
PMC3071321	1	FALSE	15	FALSE		2	16	FALSE	16	TRUE	14	TRUE	14	TRU
PMC3071320	2	FALSE	12	FALSE		5	10	TRUE	4	TRUE	4	TRUE	3	TRU
PMC3073868	5	FALSE	0	TRUE		4	5	TRUE	4	TRUE	4	TRUE	1	TRU

JE	TRUE	10	TRUE	accelerometry use (896 results) S ['21439040'], accelerometer data (169 results) D ['21104522'], cross-sectional study European Youth (1759 results) S ['21281466'], processing with accelerometers (131 results) S ['21439040'], accelerometers among children (228 results) S ['21439040'], diary data (314 results) D ['21104522'], sedentary behaviour measurement (146 results) S ['21439040'], non-wearing (18 results) D ['21104522'], non-wearing (18 results) S ['21439040'], cross-European (17 results) S ['20500859'], cross-sectional analysis of accelerometer (169 results) S ['21439040', '21281466'], ENERGY-project (18 results) S ['21439040', '21281466', '20500859'], accelerometry (896 results) S ['21439040'], sedentary behaviour (225 results) S ['21439040', '21281466'], accelerometer study protocol (62 results) S ['21439040', '21281466'], data processing with accelerometers (57 results) S ['21439040']
JE	TRUE	9	TRUE	views about body (1734 results) S ['21439062'], interpretive (3561 results) S ['21439062'], interpretive synthesis (15 results) S ['21439062'], quality studies (1081 results) S ['21439062'], aggregative (784 results) S ['21439062'], aggregative synthesis (296 results) S ['21439062'], experiences of body (3697 results) S ['21439062'], overweight people (156 results) S ['21439062'], Children's views about obesity (28 results) S ['21439062'], lowest quality (82 results) S ['21439062'], views about obesity (280 results) S ['21439062'], lowest quality studies (2066 results) S ['21439062'], children's views about body (54 results) S ['21439062']
JE	TRUE	12	TRUE	different understandings of illness (41 results) S ['21439059'], material for migrants (132 results) S ['21439059'], good practice components (492 results) S ['21439059'], migrant patients through experience (45 results) S ['21439059'], language barriers (571 results) S ['21439059'], emergency hospital departments (4 results) S ['21439059'], understandings of illness (217 results) S ['21439059'], information material for migrants (22 results) S ['21439059'], different migrant groups (5 results) S ['21439059'], migrant patients (48 results) S ['21439059'], different migrant (956 results) S ['21439059'], care for migrant (1465 results) S ['21439059'], migrant groups (206 results) S ['21439059'], constitutes good practice (161 results) S ['21439059'], services across Europe (2186 results) S ['21439059'], health services across Europe (3680 results) S ['21439059'], cultural differences (2522 results) S ['21439059'], organisational flexibility (5 results) S ['21439059'], health care for migrant (2594 results) S ['21439059']
JE	TRUE	13	TRUE	healthy dietary (294 results) S ['21439049'], effective parental support programmes (135 results) S ['21439049'], school classes (272 results) S ['21439049'], school health (14021 results) S ['21439049'], school health care (107 results) S ['21439049'], socio-economic status between families (12720 results) S ['21439049'], healthy dietary habits (69 results) S ['21439049'], sedentary behaviours (108 results) S ['21439049'], physical activity habits (207 results) S ['21439049'], parental support programmes (734 results) S ['21439049'], activity habits (254 results) S ['21439049'], cluster-randomised (744 results) S ['21439049'], parental support (486 results) S ['21439049'], dietary habits (4289 results) S ['21439049'], parental component (9 results) D ['15489466'], parental component (9 results) S ['21439049']
JE	TRUE	2	TRUE	Postpolypectomy colonoscopy surveillance predictive (3 results) D ['18347350'], colonoscopy surveillance predictive accuracy (32 results) D ['18347350'], physical activity consultation (5 results) S ['19317825'], BeWEL intervention (1 results) S ['21439044'], colorectal directions for intervention (11 results) S ['18631284'], Type Time2Act (1 results) S ['19317825'], minimal contact lifestyle intervention (2 results) S ['19640325'], colorectal adenomas (1699 results) S ['21439044'], Time2Act (1 results) S ['19317825'], contact lifestyle intervention (2 results) S ['19640325'], follow-up measures of adenoma (201 results) S ['21439044', '19640325'], activity consultation (5 results) S ['19317825'], minimal contact lifestyle (3 results) S ['19640325'], BeWEL (1 results) S ['21439044']
JE	TRUE	0	FALSE	investigator's choice therapy (4 results) D ['19581539'], K562R cells (13 results) D ['20148593'], CLL-1 (61 results) D ['20017722', '17609428'], expression of CLL-1 (19 results) D ['20017722', '17609428'], investigator's choice (20 results) D ['19581539']

PMC3066795	3	FALSE	14	FALSE		2	13	FALSE	13	TRUE	11	TRUE	10	TRU
PMC3072302	14	FALSE	17	FALSE		6	31	TRUE	21	TRUE	18	TRUE	10	TRU
PMC3072349	3	FALSE	6	FALSE		4	9	FALSE	9	FALSE	9	FALSE	9	TRU
PMC3063614	8	FALSE	6	FALSE		4	14	TRUE	8	TRUE	5	TRUE	4	TRU
PMC3072301	0	TRUE	11	FALSE		1	11	TRUE	10	TRUE	9	TRUE	9	TRU

JE	TRUE	8	TRUE	EEGs (3235 results) S ['21472032'], hand paralysis (46 results) S ['21472032'], trigger rates during attempted-movements (1 results) S ['21472032'], recovery of volitional (170 results) D ['20208465'], movement-assist (1 results) S ['21472032'], movement-onset (538 results) S ['21472032'], complete hand paralysis (251 results) S ['21472032'], movement-assist devices (1 results) S ['21472032'], attempted-movements (8 results) S ['21472032'], attempted-movement (15 results) D ['20208465'], movement-assist device (1 results) S ['21472032'], false trigger (130 results) S ['21472032'], finger-extension events (6 results) S ['21472032'], finger-extension (299 results) D ['20208465'], finger-extension (299 results) S ['21472032'], EEG-triggered (21 results) S ['21472032'], motor-planning (496 results) S ['21472032']
JE	TRUE	4	TRUE	Study-Sleep Scale 9-item (3 results) S ['21439051'], Sleep Disturbance subscale (5 results) D ['18411009'], Sleep Disturbance subscale (5 results) S ['21439051', '20236172'], severity levels correspond (101 results) D ['20303665'], 9-item Sleep (11 results) S ['21439051'], MOS-SS Sleep (6 results) D ['18411009'], clinical IMMPACT recommendations (13 results) D ['18055266', '15621359'], painful diabetic peripheral neuropathy (82 results) D ['20303665'], chronic pain clinical IMMPACT (12 results) D ['18055266', '15621359'], pain interference (326 results) S ['21439051'], Scale 9-item Sleep (8 results) S ['21439051'], MOS-SS Sleep Disturbance subscale (2 results) D ['18411009'], Sleep Interference (72 results) S ['21439051', '20236172'], pain severity levels correspond (9 results) D ['20303665'], pain clinical IMMPACT recommendations (12 results) D ['18055266', '15621359'], posttraumatic peripheral neuropathic pain (24 results) S ['21439051'], posttraumatic peripheral neuropathic (15 results) S ['21439051'], pain clinical IMMPACT (16 results) D ['18055266', '15621359'], 9-item Sleep Problems Index (3 results) S ['21439051'], painful diabetic peripheral (83 results) D ['20303665'], Disturbance subscale (14 results) D ['18411009'], Disturbance subscale (14 results) S ['21439051', '20236172'], clinical IMMPACT (26 results) D ['18055266'], 9-item Sleep Problems (3 results) S ['21439051'], pain severity (1826 results) S ['21439051'], MOS-SS Sleep Disturbance (3 results) D ['18411009'], Outcomes Study-Sleep Scale 9-item (3 results) S ['21439051'], Scale 9-item Sleep Problems (2 results) S ['21439051'], Sleep Interference Scale (107 results) S ['21439051'], Interference Scale (52 results) S ['21439051'], Study-Sleep Scale 9-item Sleep (3 results) S ['21439051']
JE	TRUE	5	TRUE	CHF patients (1795 results) S ['21439047'], GP's practice guideline (38 results) D ['16766225'], practice size (224 results) S ['21439047'], PACIC (29 results) D ['19131469'], PACIC (29 results) S ['21439047'], goal attainment (807 results) S ['21439047'], primary care for CHF (316 results) D ['21127097'], primary care for CHF (316 results) S ['21439047'], implementation programme (40 results) S ['21439047']
JE	TRUE	3	TRUE	arteriovenous collective review (7 results) D ['9294664'], contemporary cardiac embryology (24 results) S ['21468248'], Coronary arteriovenous collective review (3 results) D ['9294664'], sinus atresia (7 results) D ['15744464'], sinus atresia (7 results) S ['21468248'], coronary sinus (7114 results) S ['21468248'], atrioventricular canal myocardial connections (2 results) D ['18506001'], canal myocardial connections (2 results) D ['18506001'], accessory atrioventricular canal myocardial (5 results) D ['18506001'], accessory pathways (1324 results) S ['21468248'], accessory atrioventricular canal (20 results) D ['18506001'], Coronary Sinus Atresia (5 results) D ['15744464'], Coronary Sinus Atresia (5 results) S ['21468248'], Review Coronary arteriovenous (595 results) S ['21468248']
JE	TRUE	7	TRUE	physical activity level presents (186 results) S ['21439052'], typical physical activity level (340 results) S ['21439052'], activity counts (315 results) S ['21439052'], valid physical activity guidelines (34 results) S ['21439052'], valid physical activity (1452 results) S ['21439052'], individual's typical baseline physical (3 results) S ['21439052'], accelerometry (896 results) S ['21439052'], typical baseline physical activity (82 results) S ['21439052'], individual's typical baseline (8 results) S ['21439052'], typical baseline physical (340 results) S ['21439052'], typical physical activity (2620 results) S ['21439052']

PMC3065743	8	FALSE	11	FALSE		6	16	TRUE	9	TRUE	8	TRUE	6	TRU
PMC3076235	12	FALSE	9	FALSE		8	20	TRUE	15	TRUE	13	TRUE	10	TRU
PMC3072317	0	TRUE	7	FALSE		1	7	FALSE	7	FALSE	7	FALSE	7	TRU
PMC3071330	8	FALSE	17	FALSE		5	25	TRUE	22	TRUE	19	TRUE	15	TRU
PMC3072310	6	FALSE	4	FALSE		2	9	TRUE	8	TRUE	8	TRUE	6	TRU

JE	TRUE	2	TRUE	annum var angulosum (1 results) S ['15357919'], Tg2576 transgenic (78 results) S ['20682291'], Tg2576 transgenic mice (47 results) S ['20682291'], amyloid beta-peptide accumulation (15 results) S ['20682291'], oral vaccination (815 results) S ['20682291'], oral plant-made measles vaccine (2 results) D ['16154244'], plant-made measles vaccine (2 results) D ['16154244'], angulosum for oral (3 results) S ['15357919'], Capsicum annum var angulosum (1 results) S ['15357919'], Transgenic brown (1143 results) S ['21448341'], beta-peptide accumulation (18 results) S ['20682291'], scutellum tissue with Agrobacterium (3 results) D ['16961734'], S65T-type green fluorescent protein (1 results) D ['10406127'], infection of scutellum (10 results) D ['16961734'], plant-made measles (2 results) D ['16154244'], oral plant-made (21 results) D ['16154244'], oral plant-made measles (2 results) D ['16154244'], Transgenic brown rice (27 results) S ['21448341'], var angulosum for oral (3 results) S ['15357919']
JE	TRUE	6	TRUE	bypass multicenter European experience (10 results) D ['17723822'], multi-factorial retrospective analysis (22 results) D ['19734173'], invasive LAD (442 results) S ['21439055'], impact of port-access (15 results) D ['10536960'], financial impact of port-access (5 results) D ['10536960'], endoscopic coronary (5998 results) S ['21439055'], off-pump TECAB (17 results) D ['16631654'], off-pump TECAB (17 results) S ['21439055'], MIDCAB (318 results) S ['21439055'], invasive direct coronary (1330 results) S ['21439055'], TECAB (52 results) D ['19542085'], TECAB (52 results) S ['21439055'], 1-stop hybrid revascularization (2 results) D ['19147039'], invasive direct coronary artery (407 results) D ['19734173'], PA-CABG (5 results) D ['10086524'], PA-CABG (5 results) S ['21439055'], direct coronary artery (482 results) D ['19734173'], TECAB group (7 results) S ['21439055'], on-pump TECAB (3 results) S ['21439055'], port-access coronary revascularization (1 results) D ['10536960'], multi-factorial retrospective (41 results) D ['19734173']
JE	TRUE	6	TRUE	cardiac tissue (3947 results) S ['21439030'], perioperative nutrition on amino (51 results) S ['21439030'], dimethylarginine (1708 results) S ['21439030'], acid profile (2090 results) S ['21439030'], cardiomyocytes structure (2711 results) S ['21439030'], amino acid profile (425 results) S ['21439030'], cardiac perfusion (320 results) S ['21439030']
JE	TRUE	12	TRUE	positive expression of maspin (102 results) S ['21439064'], nuclear expression of maspin (54 results) S ['21439064'], Various angiogenic regulators (414 results) S ['21439064'], expression of Ets-1 (957 results) S ['21439064'], Maspin overexpression correlates (4 results) D ['18343598'], benign tumors (6858 results) S ['21439064'], Ets-1 expression (133 results) S ['21439064'], maspin (456 results) S ['21439064'], neovascular architecture (5 results) D ['14579791'], maspin expression (185 results) S ['21439064'], angiogenesis cascade (11 results) D ['15173033'], angiogenesis cascade (11 results) S ['21439064'], Ets-1 (1225 results) S ['21439064'], pathological parameters of ovarian (1008 results) S ['21439064'], tumor neovascular architecture (9 results) D ['14579791'], expression of maspin (373 results) S ['21439064'], destabilizes host vasculature (2 results) D ['12810677'], factor up-regulates angiopoietin-2 (12 results) D ['12810677'], correlation with Ang-2 (98 results) S ['21439064'], growth factor up-regulates angiopoietin-2 (13 results) D ['12810677'], Ets-1 target genes (8 results) S ['21439064'], Ang-2 (572 results) S ['21439064'], positive correlation with Ang-2 (35 results) S ['21439064'], factor Ets-1 (111 results) S ['21439064'], temporal-spatial expression of VEGF (6 results) D ['14579791']
JE	TRUE	5	TRUE	copper nanoparticles (114 results) S ['21439072'], apoptosis towards cancer cells (731 results) S ['21439072'], copper metal (106 results) D ['20621582'], degradable copper oxide nanoparticles (1 results) D ['20621582'], degradable copper oxide (2 results) D ['20621582'], apoptosis towards cancer (1258 results) S ['21439072'], degradable copper (15 results) D ['20621582'], Nanoparticle cytotoxicity (12 results) D ['20621582'], copper oxide nanoparticles (23 results) D ['20621582'], DNA degradation (1952 results) S ['21439072']

PMC3072921	1	FALSE	12	FALSE		5	12	FALSE	12	TRUE	11	TRUE	11	TRU
PMC3071312	8	FALSE	17	FALSE		5	23	TRUE	15	TRUE	13	TRUE	12	TRU
PMC3071779	8	FALSE	19	FALSE		12	27	TRUE	24	TRUE	22	TRUE	17	TRU
PMC3070741	3	FALSE	16	FALSE		2	18	FALSE	18	FALSE	18	TRUE	17	TRU

JE	TRUE	8	TRUE	vascular abnormalities (2023 results) S ['21439035'], vascular defects (301 results) S ['21439035'], cardiovascular disease implicate vascular (291 results) S ['21439035'], AG020569 (23 results) S ['20150433', '19103257', '16988487'], vascular-neuronal axis (1 results) S ['21439035'], cardiovascular disease implicate (1105 results) S ['21439035'], brain endothelial (1096 results) S ['21439035'], disease implicate vascular mechanisms (68 results) S ['21439035'], implicate vascular mechanisms (172 results) S ['21439035'], cerebrovascular dysfunction precedes cognitive (6 results) D ['15929050'], disease implicate vascular (296 results) S ['21439035'], broad spectrum of neurodegenerative (144 results) S ['21439035'], atherosclerotic cardiovascular disease implicate (77 results) S ['21439035']
JE	TRUE	6	TRUE	comparative view of metabolite (92 results) D ['20346409'], genotypic adaptation (23 results) S ['21435272'], substrate stress (7 results) D ['20346409'], reveals flexible selection (23 results) S ['19849850'], media reveals flexible selection (1 results) S ['19849850'], resequencing (1086 results) S ['21435272'], Hfq modulates (9 results) D ['17158661'], flexible selection of adaptive (56 results) S ['19849850'], cytoplasmic stress response (11 results) D ['17158661'], exogenous isobutanol (4 results) S ['21435272'], minimal media reveals (41 results) S ['19849850'], lactate minimal media reveals (3 results) S ['19849850'], complex stress tolerance phenotypes (89 results) S ['21435272'], media reveals flexible (5 results) S ['19849850'], complex stress tolerance (752 results) S ['21435272'], isobutanol stress (1 results) S ['21435272'], hfq (340 results) S ['21435272'], isobutanol tolerance (2 results) D ['21179021'], isobutanol tolerance (2 results) S ['21435272'], sigma32-mediated (3 results) D ['17158661'], minimal media reveals flexible (2 results) S ['19849850'], sigmaE-mediated (5 results) D ['17158661'], lactate minimal media (134 results) S ['19849850'], isobutanol (188 results) D ['21179021'], isobutanol (188 results) S ['21435272']
JE	TRUE	8	TRUE	anti-CCP antibodies (295 results) S ['21439056'], anti-CCP antibody isotypes (20 results) D ['17133560'], anti-CCP antibody isotypes (20 results) S ['21439056', '18578976', '18460272'], anti-citrullinated (192 results) S ['21439056'], IgM RF-positive polyarthritis patients (66 results) S ['21439056', '18578976'], isotypes of anti-cyclic (23 results) D ['17133560'], isotypes of anti-cyclic (23 results) S ['21439056', '18578976', '18556446'], Multiple antibody reactivities (213 results) D ['18635594'], IgG anti-CCP antibodies (3 results) S ['21439056'], IgM RF-positive polyarticular JIA (3 results) D ['12672206'], IgM RF-positive polyarticular JIA (3 results) S ['21439056'], fibrinogen antibodies (40 results) D ['18322974'], fibrinogen antibodies (40 results) S ['21439056'], RF-positive polyarthritis (14 results) S ['21439056', '18578976', '15338508'], citrullinated (1313 results) S ['21439056'], IgM RF-positive polyarthritis (74 results) S ['21439056', '18578976'], RF-positive polyarthritis patients (265 results) S ['21439056'], RF-positive polyarticular JIA (19 results) D ['19842993', '16234183', '15547097', '12672206'], RF-positive polyarticular JIA (19 results) S ['21439056'], anti-cyclic (617 results) S ['21439056'], IgM RF-positive (23 results) D ['12672206'], IgM RF-positive (23 results) S ['21439056'], target for citrullination (27 results) S ['21439056'], IgM RF-positive polyarticular (7 results) D ['12672206'], IgM RF-positive polyarticular (7 results) S ['21439056'], significance of isotypes (286 results) S ['21439056'], anti-CCP (635 results) S ['21439056']
JE	TRUE	12	TRUE	non-inferiority trial with allocation (23 results) S ['21483704'], availability of CBT (57 results) S ['21483704'], Cognitive behavioral group therapy (121 results) D ['20194829'], Cognitive behavioral group therapy (121 results) S ['21483704'], ICBT for SAD (1 results) S ['21483704'], CBGT (53 results) D ['20194829'], CBGT (53 results) S ['21483704'], non-inferiority trial (160 results) S ['21483704'], behavioral group (355 results) S ['21483704'], Cognitive behavioral group (265 results) S ['21483704'], behavioral group therapy (138 results) D ['20194829'], behavioral group therapy (138 results) S ['21483704'], social anxiety (2174 results) S ['21483704'], facilitate dissemination of therapeutic (199 results) S ['21483704'], non-inferiority margin (123 results) S ['21483704'], increase availability of CBT (15 results) S ['21483704'], non-inferiority (1006 results) S ['21483704'], ICBT (81 results) S ['21483704'], psychiatric setting (385 results) S ['21483704']

PMC3064677	2	FALSE	15	FALSE		4	17	TRUE	10	TRUE	6	TRUE	5	TRU
PMC3064675	3	FALSE	15	FALSE		10	15	TRUE	11	TRUE	11	TRUE	11	TRU
PMC3064674	4	FALSE	17	FALSE		4	20	TRUE	16	TRUE	15	TRUE	13	TRU
PMC3064673	19	FALSE	2	FALSE		9	21	FALSE	21	TRUE	19	TRUE	16	TRU

JE	TRUE	3	TRUE	panitumumab (481 results) S ['21464917'], pleural mesothelial cells correlates (9 results) D ['11133818'], 86Y for quantitative (10 results) S ['20484421', '20155263'], anti-HER1 monoclonal (5 results) S ['20484421'], chimeric radioimmunotherapy (188 results) S ['21464917'], anti-HER1 (9 results) S ['20484421'], human anti-HER1 monoclonal antibody (3 results) S ['20484421'], monoclonal antibody panitumumab (6 results) S ['20484421'], human anti-HER1 monoclonal (3 results) S ['20484421'], cells correlates with carcinogenicity (33 results) D ['11133818'], human anti-HER1 (5 results) S ['20484421'], quantitative PET of carcinoma (173 results) S ['20484421'], anti-HER1 monoclonal antibody panitumumab (2 results) S ['20484421'], 86Y (82 results) S ['20484421', '20155263'], anti-HER1 monoclonal antibody (5 results) S ['20484421'], antibody panitumumab (16 results) S ['20484421'], HER1-expressing (5 results) S ['20484421', '20155263']
JE	TRUE	8	TRUE	universal application of mycobacterial (2 results) D ['19458183'], repetitive tandem (3840 results) S ['21464915'], major RDRio Sublineage (1 results) D ['18234868'], reference LSPs (1 results) S ['15243038'], repetitive-tandem-repeat (4321 results) S ['21464915'], repetitive tandem repeat analysis (2378 results) S ['21464915'], specific MIRU-VNTR (26 results) S ['21464915', '17898156'], tandem-repeat (9302 results) S ['21464915'], value of 24-locus (2 results) S ['17192416', '17005759'], Prospective universal application (70 results) D ['19458183'], spoligotyping (599 results) S ['21464915'], repetitive tandem repeat (4321 results) S ['21464915'], multifunctional database for online (4 results) S ['18550737'], phylogenetic identification of Mycobacterium (81 results) S ['21464915', '20457747'], tandem repeat (9302 results) S ['21464915'], use of MIRU-VNTRplus (6 results) S ['21464915', '20457747', '18550737', '18234864'], interspersed (6874 results) S ['21464915'], MIRU-VNTRplus (6 results) S ['21464915', '20457747', '18550737', '18234864']
JE	TRUE	9	TRUE	benthic sticklebacks (5 results) S ['21464914'], marine sticklebacks (10 results) S ['21464914'], behavior from marine (3830 results) S ['21464914'], sticklebacks (359 results) S ['21464914'], identical social circumstances (27 results) S ['21464914'], small conspecific fish (24 results) D ['20582314'], benthic sticklebacks exhibit (5 results) S ['21464914'], simultaneous optomotor (5 results) D ['20582314'], marine sticklebacks show (356 results) S ['21464914'], Wark (705 results) S ['21464914'], threespine (171 results) S ['21464914'], experimental stickleback (325 results) S ['21464914'], alternative habitats differ (57 results) S ['21464914'], fish shoals (13 results) D ['18474860', '10675263'], model school (19 results) S ['21464914'], simultaneous optomotor response (4 results) D ['20582314'], threespine stickleback (144 results) S ['21464914'], tendency between marine (183 results) S ['21464914'], stationary shelter options (1 results) S ['21464914'], Peichel (36 results) S ['21464914'], sticklebacks exhibit (119 results) S ['21464914']
JE	TRUE	7	TRUE	regulator Fur reveals (50 results) D ['21208302', '20644138', '19400801'], metal-dependent responses (20 results) D ['20579104', '16541078'], functional metal (61 results) D ['21208302'], ferric uptake regulator Fur (132 results) D ['21208302', '20644138', '20518707'], pylori ferric uptake regulator (33 results) D ['21208302', '20644138', '20518707'], ferric uptake (600 results) D ['21208302', '20644138'], Helicobacter pylori network motifs (6 results) D ['20579104'], Roles of FrxA (13 results) S ['11489869', '11036035'], RdxA (61 results) D ['16547053'], Helicobacter pylori ferric (51 results) D ['21208302', '20644138', '20518707'], pylori network motifs (6 results) D ['20579104'], ferric uptake regulator (225 results) D ['21208302', '20644138', '20518707'], pylori ferric uptake (43 results) D ['21208302', '20644138', '20518707'], uptake regulator (249 results) D ['21208302', '20644138', '20518707'], uptake regulator Fur (139 results) D ['21208302', '20644138', '20518707'], FrxA (26 results) S ['11489869'], regulator Fur (174 results) D ['21208302', '20644138', '20518707'], pylori ferric (52 results) D ['21208302', '20644138', '20518707'], uptake regulator Fur reveals (15 results) D ['21208302', '20644138', '19400801'], Helicobacter pylori ferric uptake (43 results) D ['21208302', '20644138', '20518707'], Helicobacter pylori network (117 results) D ['20579104']

PMC3064671	3	FALSE	8	FALSE		4	8	TRUE	7	TRUE	5	TRUE	2	TRU
PMC3064669	4	FALSE	5	FALSE		4	8	TRUE	6	TRUE	5	TRUE	5	TRU
PMC3064668	0	TRUE	12	FALSE		3	12	TRUE	10	TRUE	9	TRUE	9	TRU
PMC3064667	4	FALSE	3	FALSE		4	6	TRUE	4	TRUE	2	TRUE	2	TRU
PMC3064666	0	TRUE	3	FALSE		1	3	FALSE	3	FALSE	3	TRUE	2	TRU
PMC3064663	5	FALSE	7	FALSE		6	12	TRUE	11	TRUE	9	TRUE	8	TRU
PMC3064662	7	FALSE	11	FALSE		4	14	TRUE	12	TRUE	9	TRUE	8	TRU
PMC3064661	1	FALSE	3	FALSE		2	4	FALSE	4	FALSE	4	FALSE	4	TRU

JE	TRUE	2	TRUE	gp140 protein (19 results) S ['19712773'], AE-specific (10 results) S ['19712773'], Low TRBP levels (4 results) S ['16188979'], Low TRBP (7 results) S ['16188979'], DNA vaccine (3062 results) S ['21464971'], repeat-driven PKR cDNA (1 results) D ['10516008'], repeat-driven PKR cDNA construct (1 results) D ['10516008'], TRBP levels (11 results) S ['16188979'], recombinant gp140 protein (124 results) S ['19712773'], recombinant gp140 (17 results) S ['19712773'], terminal repeat-driven PKR (1 results) D ['10516008']
JE	TRUE	4	TRUE	Schooler (385 results) S ['21464969'], dilation assessment (5 results) D ['14527574'], concurrent pupil dilation (1 results) D ['14527574'], spontaneous cognitive (1958 results) S ['21464969'], Comment on Smallwood (10 results) D ['20192557'], Smallwood (850 results) S ['21464969'], spontaneous cognitive activity (682 results) S ['21464969'], temporal focus (100 results) S ['19121953'], concurrent pupil dilation assessment (2 results) D ['14527574']
JE	TRUE	5	TRUE	stroma regulate tumor (211 results) S ['21464968'], stroma regulate tumor development (60 results) S ['21464968'], hematopoietic tumor stroma supports (8 results) S ['21464968'], tumorigenic cell line supports (38 results) S ['18369099'], tumor stroma regulate (211 results) S ['21464968'], supports tumor outgrowth (45 results) S ['21464968'], hematopoietic stem cells (35921 results) S ['21464968'], liver cell population (40 results) S ['14592820'], tumor stroma (1163 results) S ['21464968'], hematopoietic compartment (192 results) S ['21464968'], stroma supports tumor outgrowth (3 results) S ['21464968'], fetal liver cell population (2 results) S ['14592820']
JE	TRUE	2	TRUE	intestinal IL-10 (2 results) D ['12595590'], Interleukin inhibits (9464 results) S ['21464967'], lactis strain prevents (10 results) D ['17316776'], Autocrine IL-10 impairs dendritic (1 results) D ['11920565'], bovine IL-10 (5 results) S ['21464967'], Interleukin inhibits cytokine (8547 results) S ['21464967'], Lactococcus lactis strain prevents (7 results) D ['17316776']
JE	TRUE	2	TRUE	pure parsimony (11 results) S ['21464966'], haplotyping (633 results) S ['21464966'], parsimony (4132 results) S ['21464966']
JE	TRUE	3	TRUE	coli predispose (201 results) D ['20811584'], pandemic Escherichia (45 results) S ['20118165'], Glycolipid receptors for uropathogenic (16 results) D ['7037645'], virulence mechanisms of UPEC (21 results) D ['18482721'], O25b-ST131 clone (5 results) D ['19474064'], pandemic Escherichia coli (45 results) S ['20118165'], human pandemic Escherichia (32 results) S ['20118165'], uropathogenic (1470 results) S ['21464963'], Uropathogenic Escherichia coli (824 results) S ['21464963'], human pandemic Escherichia coli (32 results) S ['20118165'], O25b-ST131 clone of Escherichia (10 results) D ['19474064'], O25b-ST131 clone of Escherichia (10 results) S ['20118165']
JE	TRUE	2	TRUE	captive subadult (41 results) S ['21464962'], third-party affiliation (7 results) D ['17240341'], mentality of convergent (1 results) D ['15591194'], Corvus frugilegus (36 results) D ['17240341'], frugilegus (38 results) D ['17240341'], same relationship repair (1191 results) S ['21464962'], Postconflict third-party (7 results) D ['17240341'], relationship quality on reconciliation (25 results) S ['20485685'], apparent absence of reconciliation (2 results) S ['21464962'], captive subadult ravens (1 results) S ['21464962'], former opponents (25 results) D ['17240341'], former opponents (25 results) S ['21464962', '20485685'], Postconflict third-party affiliation (3 results) D ['17240341'], post-conflict (215 results) S ['21464962'], subadult ravens (1 results) S ['21464962'], post-conflict distress (13 results) S ['21464962', '20485685'], primate-like valuable relationships (1 results) S ['21464962'], valuable relationships (7 results) S ['21464962']
JE	TRUE	2	TRUE	Species-specific variation of alternative (55 results) D ['18688268'], genomic data (1699 results) S ['21464961'], taeda (419 results) S ['21464961'], exon length (39 results) S ['21464961']
